# Supplementary material for: The Unfolding Space Glove: A Wearable Spatio-Visual to Haptic Sensory Substitution Device for Blind People
Source: Sensors (Basel). 2022 Feb 26;22(5):1859. doi: 10.3390/s22051859 (PMC8914703; doi:10.3390/s22051859)
Supplement: Supplementary file 1 [file sensors-22-01859-s001.zip › S1_GitHub-Release_unfolding-space-hardware-0.2.1-revised.zip!/unfolding-space-hardware-0.2.1-revised/pcb/datasheets/G1040003D.pdf]

产品规格书

PRODUCT SPECIFICATION

客户名称Buyer Name	
客户料号Buyer Part No.	
客户承认签章<br>Buyers Approval & Signatures	

文件编号Spec No.		版本	A/0
品名描述<br>Product Description	LRA Coin Vibration Motor		
型号Part No.	G1040003D		
送样日期Date			
设计Designed by	审核Checked by	批准Approved by	
2019.4.27	2019.4.27	2019.4.27	

Jinlong Machinery & Electronics
www.jinlong-machinery.com
sales@jinlong-machinery.com

Contents of Specification

1. Revision History

2. Application

3. Operating ,Storage Temperature and Humidity Conditions

4. Measurement Conditions

5. Specifications

6. Reliability Test Conditions

7. Cautions for Use

8. Drawing

9. Packing

Preliminary Specification

1. Revision History

Revision No	Date	Content	Approval	
			Draft	Approval
REV.00	2019/04/27	- Release Preliminary Specification	J.H.CHEN	J.K.CHOW

Preliminary Specification

2. Application

This specification provides structure, function and usage condition of Linear Vibrator used in mobile communication devices for silent alert. This Linear Vibrator is designed and manufactured by Jinlong Machinery & Electronics

.

3. Operating , Storage Temperature /Humidity Conditions

No	Item	Condition
3-1	Operating Temperature Range	- 25℃ ~ + 70℃
3-2	Storage Temperature Range	- 40℃ ~ + 85℃
3-3	Operating Humidity Range	Max 65% RH
3-4	Storage Humidity Range	Max 65% RH

4. Measurement Conditions

No	Item	Condition
4-1	Temperature	20 ± 5℃
4-2	Humidity	65 ± 20%RH
4-3	Rated Input Voltage	2.5Vrms AC, Sinewave
4-4	Input Voltage Range	0.1 ~ 2.5 Vrms AC
4-5	Input Frequency	150 ~ 200Hz (f0 : 170±5 Hz)
4-6	Operating Attitude	Refer to Figure 1

Preliminary Specification

※ Measurement Method

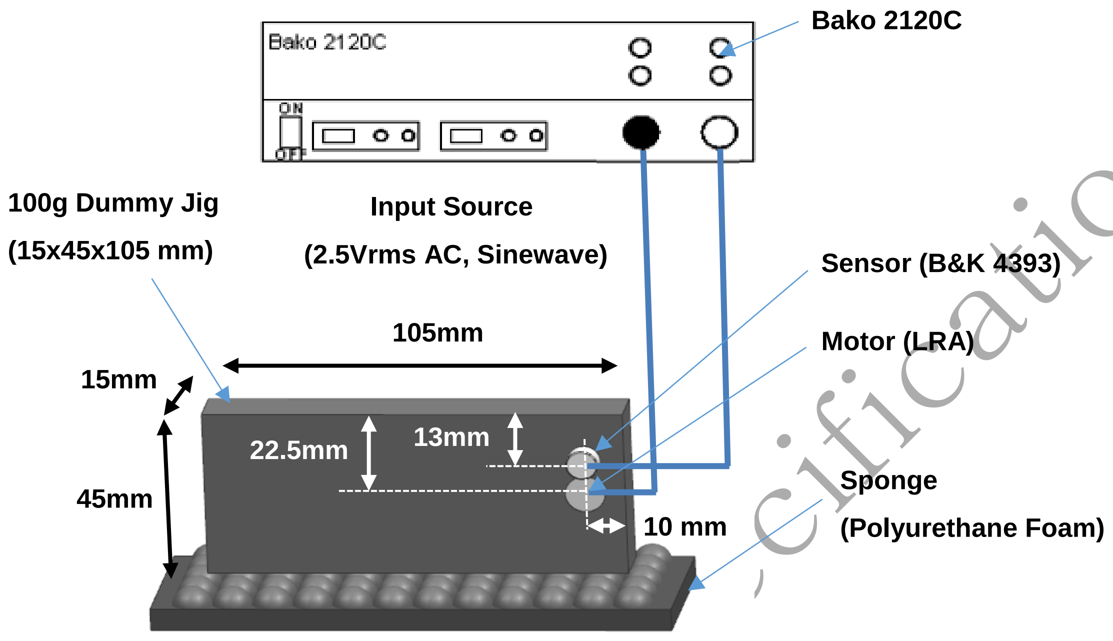

Figure 1. An Example of Measurement Method of Linear Vibrator

Preliminary Specification

☐ Position of Linear Vibrator and Accelerometer (Refer to Figure 1)

- Linear Vibrator should be mounted to vibrate 15mm direction (y-direction) of Jig.
- Accelerometer also should be installed to measure y-direction vibration of Jig

☐ Position of Dummy Jig

- 15mm*105mm plane of Dummy Jig should be located on Sponge
- At measurement of acceleration, Dummy Jig should be stabilized

☐ Measurement of Acceleration

- Acceleration should be measured 2~3 second later after input source is applied
- For the precise measurement, Acceleration should be measured 3 times and adopted average value on each Linear Vibrator

5. Specifications

No	Item	Specification
5-1	Resistance	**13.8Ω ± 15%**
5-2	Rated Current	**Max 170 mArms**<br>(Input Source : 2.5Vrms AC, Sinewave)
5-3	Acceleration	**Min 0.9 Grms @ 150Hz**<br>**Min 1.0 Grms @ 200Hz**<br>**Min 1.8 Grms @ f0**<br>(Input Source : 2.5Vrms AC, Sinewave)
5-4	Frequency Characteristics	**Refer to Graph 1**
5-5	Motor Height	**4.05 ± 0.05mm**<br>- Put the Case of the motor on Jig after zero setting and measure center point of bracket by Height Gauge.
5-6	Noise	**Max. 50 dB(A)**<br>- 10cm distance from microphone, (Input Source : 2.5Vrms AC, Sinewave)
5-7	Noise by mechanical touch (Noise_T)	**Max 35dB**<br>(Input Source : 2.5Vrms AC, Sinewave)<br>- This is full inspection method in the mass production instead of measurement of 5-6 Noise<br>- Measurement method<br>· Equipment : Bako 2120C<br>· It measures Noise touch(Mechanical touch) through vibration signal by acceleration sensor
5-8	Insulation Resistance	**Min 10 MΩ**<br>(Input 100V DC, the insulation resistance between the vibrator case and terminal)

Preliminary Specification

No	Item	Specification
5-9	Rising Time	**Max 10msec**<br>- The time reaching to 50% of normal acceleration from power on<br>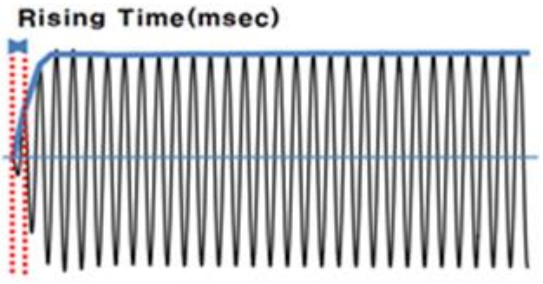
5-10	Falling Time	**Max 50msec**<br>- The time reaching to 10% of normal acceleration from power off<br>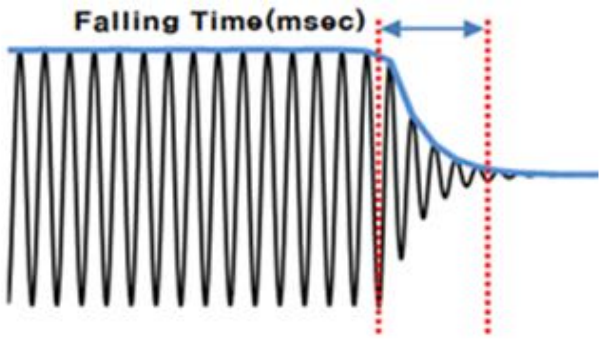

Preliminary Specification

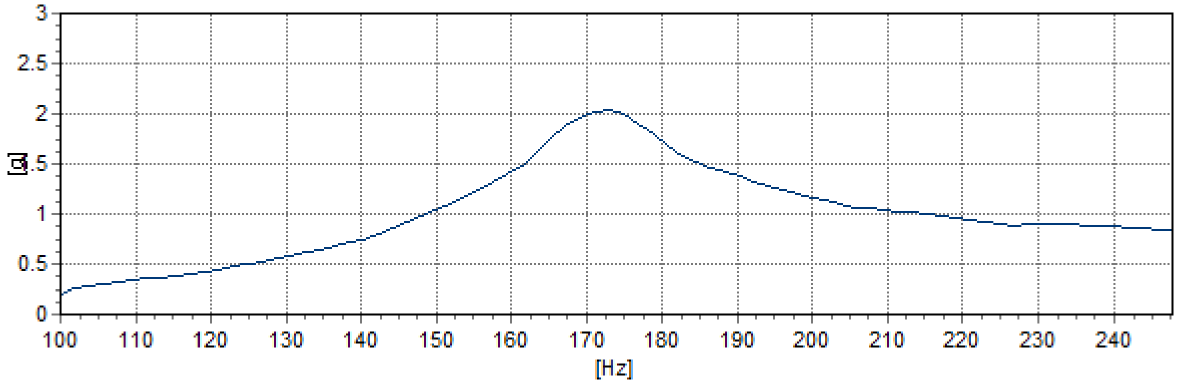

Graph 1. Frequency Characteristics

Preliminary Specification

6. Reliability Test Condition

No	Item	Condition
6-1	Life test	Operating at rated input voltage and input frequency for 1,000,000 cycles. 1 cycle is 2 Sec On, 1 Sec Off.
6-2	Thermal shock test	- 40℃ ~ 85℃ in each of 2Hrs(1cycle), Total 15 cycles. Transition time is 5 minutes max. After the test, the Vibrator should be measured after room-temperature storage for 4Hrs.
6-3	High temperature storage test	+70℃, 168Hrs, After the test, the Vibrator should be measured after room-temperature storage for 4Hrs.
6-4	Low temperature storage test	-30℃, 168Hrs, After the test, the Vibrator should be measured after room-temperature storage for 4Hrs.
6-5	Static humidity test	+50℃, 95%RH, 120Hrs, After the test, the Vibrator should be measured after room-temperature storage for 4Hrs.
6-6	Vibration test	Vibrator that is attached to a 160g dummy jig is vibrated with 2.2G, 10~55Hz/min for 10min in each of X,Y,Z axis..
6-7	Mechanical shock test	The Vibrator that is attached to a 160g dummy jig is dropped to a steel floor 30 times(6 face, 5 times in each of X,Y,Z axis) from 1.5m in height.

Preliminary Specification

Due to this LRA's wide bandwidth , the use of Haptic drivers that make use of "auto-resonance" detection can not be used. Please use the Dongwoon Anatech Part # DW7914A or equivalent.

☐ **Judgement**

① After test, The following specifications must be satisfied**.**

- Acceleration : Within initial Value ± 30%
- Rated Current : Max 170 mA rms
- Noise_T : Max. 35dB

② There should be no abnormalities in appearance and structure.

Preliminary Specification

7. Cautions for Use

(1) Do not press the product with more than 0.5Kgf or drop it.
It can cause the transformation of performance or external appearance.

(2) Do not use under the following conditions. It may cause a decline in performance

- Do not drop into fluid (such as water, alcohol etc.)
- Do not keep at high temperature or high humidity for extended periods of times
- Do not use near gases which cause erosion
- Please refrain from operating the vibrator near magnetic devices.

(3) The vibrator has a strong magnet. So please be aware that it has a magnetic force on the surface of the bracket.

(4) To optimize the vibration force, Rated frequency and voltage could be changed as to assemble condition.

(5) Please refer to the packaging drawing. It can be modified by the request of the user.

(6) If any problems are occurred, Both the user and Mplus Co.,Ltd. shall try to solve the problem by mutual agreement and on reflection of the specification sheet.

(7) The storage condition is 5℃~35℃, 15%~65% RH, 1year about packing.

Preliminary Specification

8. Drawing

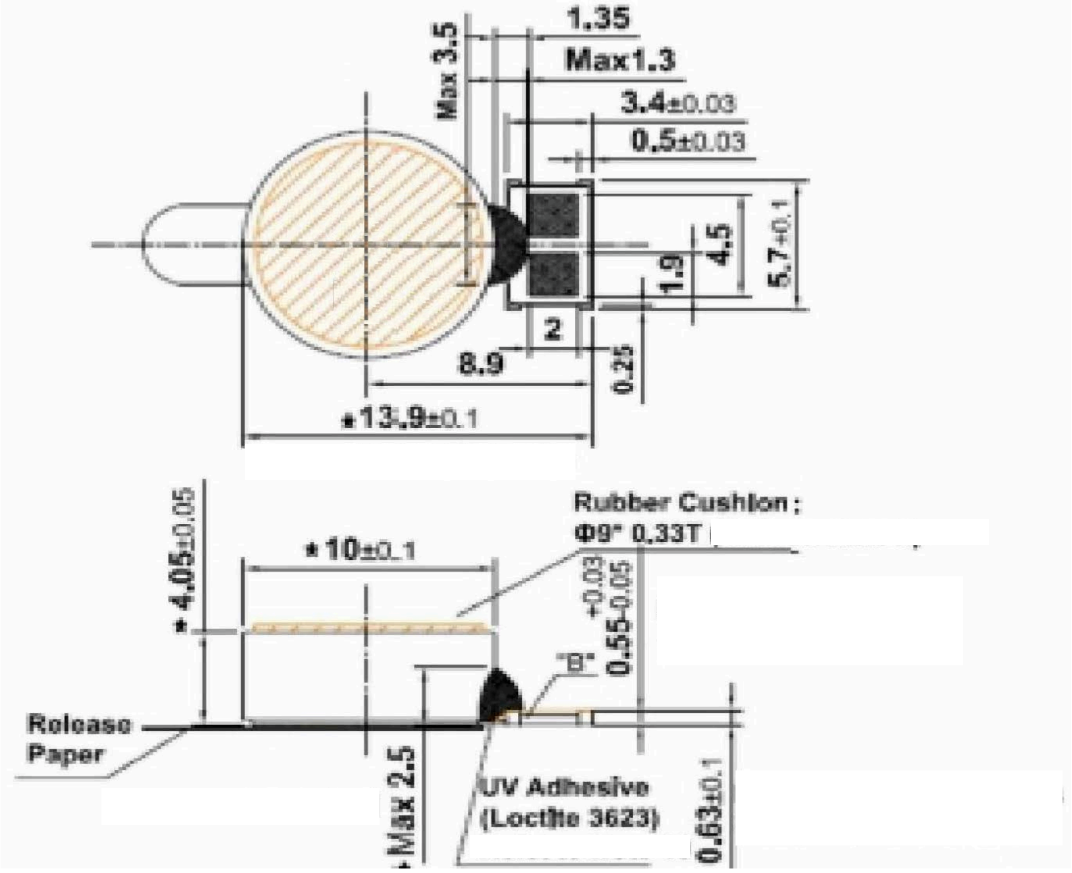

1.35
Max1.3
Max 3.5
3.4±0.03
0.5±0.03
4.5
5.7±0.1
1.9
2
8.9
0.25
★13.9±0.1
Rubber Cushion :
Φ9* 0.33T
★4.05±0.05
★10±0.1
+0.03
0.55-0.05
"B"
Release
Paper
★Max 2.5
UV Adhesive
(Loctite 3623)
0.63±0.1

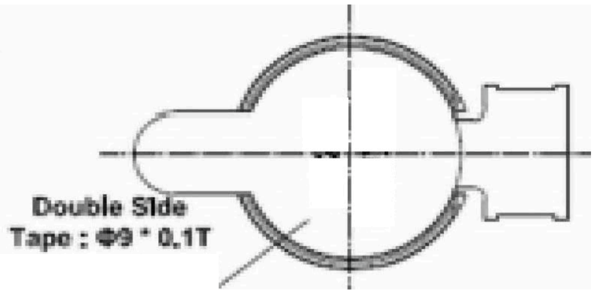

Double Side
Tape : Φ9 * 0.1T
