# Supplementary material for: The Unfolding Space Glove: A Wearable Spatio-Visual to Haptic Sensory Substitution Device for Blind People
Source: Sensors (Basel). 2022 Feb 26;22(5):1859. doi: 10.3390/s22051859 (PMC8914703; doi:10.3390/s22051859)
Supplement: Supplementary file 1 [file sensors-22-01859-s001.zip › S1_GitHub-Release_unfolding-space-hardware-0.2.1-revised.zip!/unfolding-space-hardware-0.2.1-revised/pcb/datasheets/LSM6DSL-acc-gyro.pdf]

LSM6DSL

iNEMO inertial module: always-on 3D accelerometer and 3D gyroscope

Datasheet - production data

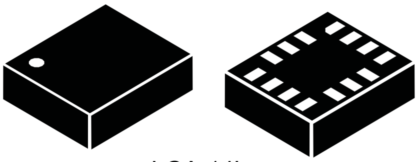

LGA-14L
(2.5 x 3 x 0.83 mm) typ.

Features

- Power consumption: 0.4 mA in combo normal mode and 0.65 mA in combo high-performance mode
- “Always-on” experience with low power consumption for both accelerometer and gyroscope
- Smart FIFO up to 4 kbyte based on features set
- Android M compliant
- Hard, soft ironing for external magnetic sensor corrections
- ±2/±4/±8/±16 *g* full scale
- ±125/±250/±500/±1000/±2000 dps full scale
- Analog supply voltage: 1.71 V to 3.6 V
- Independent IOs supply (1.62 V)
- Compact footprint, 2.5 mm x 3 mm x 0.83 mm
- SPI & I^2C serial interface with main processor data synchronization feature
- Pedometer, step detector and step counter
- Significant motion and tilt function
- Standard interrupts: free-fall, wakeup, 6D/4D orientation, click and double-click
- Embedded temperature sensor
- ECOPACK®, RoHS and “Green” compliant

Applications

- Motion tracking and gesture detection
- Collecting sensor data
- Indoor navigation
- IoT and connected devices
- Intelligent power saving for handheld devices
- Vibration monitoring and compensation

Description

The LSM6DSL is a system-in-package featuring a 3D digital accelerometer and a 3D digital gyroscope performing at 0.65 mA in high-performance mode and enabling always-on low-power features for an optimal motion experience for the consumer.

The LSM6DSL supports main OS requirements, offering real, virtual and batch sensors with 4 kbyte for dynamic data batching.

ST’s family of MEMS sensor modules leverages the robust and mature manufacturing processes already used for the production of micromachined accelerometers and gyroscopes.

The various sensing elements are manufactured using specialized micromachining processes, while the IC interfaces are developed using CMOS technology that allows the design of a dedicated circuit which is trimmed to better match the characteristics of the sensing element.

The LSM6DSL has a full-scale acceleration range of ±2/±4/±8/±16 *g* and an angular rate range of ±125/±250/±500/±1000/±2000 dps.

High robustness to mechanical shock makes the LSM6DSL the preferred choice of system designers for the creation and manufacturing of reliable products.

The LSM6DSL is available in a plastic land grid array (LGA) package.

Table 1. Device summary

Part number	Temp. range [°C]	Package	Packing
LSM6DSL	-40 to +85	LGA-14L (2.5x3x0.83mm)	Tray
LSM6DSLTR	-40 to +85		Tape & Reel

This is information on a product in full production. www.st.com

Contents

1 **Overview** **15**

2 **Embedded low-power features** **16**

2.1 Tilt detection 16

2.2 Absolute wrist tilt 17

3 **Pin description** **18**

3.1 Pin connections 19

4 **Module specifications** **21**

4.1 Mechanical characteristics 21

4.2 Electrical characteristics 24

4.3 Temperature sensor characteristics 25

4.4 Communication interface characteristics 26

4.4.1 SPI - serial peripheral interface 26

4.4.2 I^2C - inter-IC control interface 27

4.5 Absolute maximum ratings 29

4.6 Terminology 30

4.6.1 Sensitivity 30

4.6.2 Zero-g and zero-rate level 30

5 **Functionality** **31**

5.1 Operating modes 31

5.2 Gyroscope power modes 31

5.3 Accelerometer power modes 31

5.4 Block diagram of filters 32

5.4.1 Block diagrams of the gyroscope filters 32

5.4.2 Block diagrams of the accelerometer filters 33

5.5 FIFO 34

5.5.1 Bypass mode 34

5.5.2 FIFO mode 35

5.5.3 Continuous mode 35

5.5.4 Continuous-to-FIFO mode 35

5.5.5 Bypass-to-Continuous mode 35
5.5.6 FIFO reading procedure 36

6 Digital interfaces 37

6.1 I^2C/SPI interface 37
6.2 Master I^2C 37
6.3 I^2C serial interface 38
6.3.1 I^2C operation 38
6.4 SPI bus interface 40
6.4.1 SPI read 41
6.4.2 SPI write 42
6.4.3 SPI read in 3-wire mode 43

7 Application hints 44

7.1 LSM6DSL electrical connections in Mode 1 44
7.2 LSM6DSL electrical connections in Mode 2 45

8 Register mapping 48

9 Register description 52

9.1 FUNC_CFG_ACCESS (01h) 52
9.2 SENSOR_SYNC_TIME_FRAME (04h) 52
9.3 SENSOR_SYNC_RES_RATIO (05h) 53
9.4 FIFO_CTRL1 (06h) 53
9.5 FIFO_CTRL2 (07h) 54
9.6 FIFO_CTRL3 (08h) 55
9.7 FIFO_CTRL4 (09h) 56
9.8 FIFO_CTRL5 (0Ah) 57
9.9 DRDY_PULSE_CFG_G (0Bh) 58
9.10 INT1_CTRL (0Dh) 58
9.11 INT2_CTRL (0Eh) 59
9.12 WHO_AM_I (0Fh) 59
9.13 CTRL1_XL (10h) 60
9.14 CTRL2_G (11h) 61
9.15 CTRL3_C (12h) 62

9.16 CTRL4_C (13h) 63
9.17 CTRL5_C (14h) 63
9.18 CTRL6_C (15h) 65
9.19 CTRL7_G (16h) 66
9.20 CTRL8_XL (17h) 66
9.21 CTRL9_XL (18h) 67
9.22 CTRL10_C (19h) 68
9.23 MASTER_CONFIG (1Ah) 68
9.24 WAKE_UP_SRC (1Bh) 69
9.25 TAP_SRC (1Ch) 70
9.26 D6D_SRC (1Dh) 71
9.27 STATUS_REG (1Eh) 71
9.28 OUT_TEMP_L (20h), OUT_TEMP_H (21h) 72
9.29 OUTX_L_G (22h) 72
9.30 OUTX_H_G (23h) 72
9.31 OUTY_L_G (24h) 73
9.32 OUTY_H_G (25h) 73
9.33 OUTZ_L_G (26h) 73
9.34 OUTZ_H_G (27h) 74
9.35 OUTX_L_XL (28h) 74
9.36 OUTX_H_XL (29h) 74
9.37 OUTY_L_XL (2Ah) 74
9.38 OUTY_H_XL (2Bh) 75
9.39 OUTZ_L_XL (2Ch) 75
9.40 OUTZ_H_XL (2Dh) 75
9.41 SENSORHUB1_REG (2Eh) 75
9.42 SENSORHUB2_REG (2Fh) 76
9.43 SENSORHUB3_REG (30h) 76
9.44 SENSORHUB4_REG (31h) 76
9.45 SENSORHUB5_REG (32h) 76
9.46 SENSORHUB6_REG (33h) 77
9.47 SENSORHUB7_REG (34h) 77
9.48 SENSORHUB8_REG(35h) 77

9.49 SENSORHUB9_REG (36h) 77
9.50 SENSORHUB10_REG (37h) 78
9.51 SENSORHUB11_REG (38h) 78
9.52 SENSORHUB12_REG (39h) 78
9.53 FIFO_STATUS1 (3Ah) 78
9.54 FIFO_STATUS2 (3Bh) 79
9.55 FIFO_STATUS3 (3Ch) 79
9.56 FIFO_STATUS4 (3Dh) 80
9.57 FIFO_DATA_OUT_L (3Eh) 80
9.58 FIFO_DATA_OUT_H (3Fh) 80
9.59 TIMESTAMP0_REG (40h) 81
9.60 TIMESTAMP1_REG (41h) 81
9.61 TIMESTAMP2_REG (42h) 81
9.62 STEP_TIMESTAMP_L (49h) 82
9.63 STEP_TIMESTAMP_H (4Ah) 82
9.64 STEP_COUNTER_L (4Bh) 82
9.65 STEP_COUNTER_H (4Ch) 83
9.66 SENSORHUB13_REG (4Dh) 83
9.67 SENSORHUB14_REG (4Eh) 83
9.68 SENSORHUB15_REG (4Fh) 83
9.69 SENSORHUB16_REG (50h) 84
9.70 SENSORHUB17_REG (51h) 84
9.71 SENSORHUB18_REG (52h) 84
9.72 FUNC_SRC1 (53h) 85
9.73 FUNC_SRC2 (54h) 85
9.74 WRIST_TILT_IA (55h) 86
9.75 TAP_CFG (58h) 87
9.76 TAP_THS_6D (59h) 88
9.77 INT_DUR2 (5Ah) 88
9.78 WAKE_UP_THS (5Bh) 89
9.79 WAKE_UP_DUR (5Ch) 89
9.80 FREE_FALL (5Dh) 90
9.81 MD1_CFG (5Eh) 91

9.82 MD2_CFG (5Fh) 92
9.83 MASTER_CMD_CODE (60h) 93
9.84 SENS_SYNC_SPI_ERROR_CODE (61h) 93
9.85 OUT_MAG_RAW_X_L (66h) 93
9.86 OUT_MAG_RAW_X_H (67h) 93
9.87 OUT_MAG_RAW_Y_L (68h) 94
9.88 OUT_MAG_RAW_Y_H (69h) 94
9.89 OUT_MAG_RAW_Z_L (6Ah) 94
9.90 OUT_MAG_RAW_Z_H (6Bh) 94
9.91 X_OFS_USR (73h) 95
9.92 Y_OFS_USR (74h) 95
9.93 Z_OFS_USR (75h) 95

10 Embedded functions register mapping 96

11 Embedded functions registers description - Bank A 98

11.1 SLV0_ADD (02h) 98
11.2 SLV0_SUBADD (03h) 98
11.3 SLAVE0_CONFIG (04h) 98
11.4 SLV1_ADD (05h) 99
11.5 SLV1_SUBADD (06h) 99
11.6 SLAVE1_CONFIG (07h) 100
11.7 SLV2_ADD (08h) 100
11.8 SLV2_SUBADD (09h) 100
11.9 SLAVE2_CONFIG (0Ah) 101
11.10 SLV3_ADD (0Bh) 101
11.11 SLV3_SUBADD (0Ch) 101
11.12 SLAVE3_CONFIG (0Dh) 102
11.13 DATAWRITE_SRC_MODE_SUB_SLV0 (0Eh) 102
11.14 CONFIG_PEDO_THS_MIN (0Fh) 102
11.15 SM_THS (13h) 103
11.16 PEDO_DEB_REG (14h) 103
11.17 STEP_COUNT_DELTA (15h) 103
11.18 MAG_SI_XX (24h) 104

11.19 MAG_SI_XY (25h) 104
11.20 MAG_SI_XZ (26h) 104
11.21 MAG_SI_YX (27h) 104
11.22 MAG_SI_YY (28h) 105
11.23 MAG_SI_YZ (29h) 105
11.24 MAG_SI_ZX (2Ah) 105
11.25 MAG_SI_ZY (2Bh) 105
11.26 MAG_SI_ZZ (2Ch) 106
11.27 MAG_OFFX_L (2Dh) 106
11.28 MAG_OFFX_H (2Eh) 106
11.29 MAG_OFFY_L (2Fh) 106
11.30 MAG_OFFY_H (30h) 107
11.31 MAG_OFFZ_L (31h) 107
11.32 MAG_OFFZ_H (32h) 107

12 Embedded functions registers description - Bank B 108

12.1 A_WRIST_TILT_LAT (50h) 108
12.2 A_WRIST_TILT_THS (54h) 108
12.3 A_WRIST_TILT_Mask (59h) 108

13 Soldering information 109

14 Package information 110

14.1 LGA-14 package information 110
14.2 LGA-14 packing information 111

15 Revision history 113

List of tables

Table 1. Device summary 1
Table 2. Pin description 20
Table 3. Mechanical characteristics 21
Table 4. Electrical characteristics 24
Table 5. Temperature sensor characteristics 25
Table 6. SPI slave timing values (in mode 3) 26
Table 7. I^2C slave timing values 27
Table 8. I^2C master timing values. 28
Table 9. Absolute maximum ratings 29
Table 10. Serial interface pin description 37
Table 11. Master I^2C pin details 37
Table 12. I^2C terminology 38
Table 13. SAD+Read/Write patterns 39
Table 14. Transfer when master is writing one byte to slave 39
Table 15. Transfer when master is writing multiple bytes to slave 39
Table 16. Transfer when master is receiving (reading) one byte of data from slave 39
Table 17. Transfer when master is receiving (reading) multiple bytes of data from slave 39
Table 18. Internal pin status 46
Table 19. Registers address map. 48
Table 20. FUNC_CFG_ACCESS register. 52
Table 21. FUNC_CFG_ACCESS register description 52
Table 22. Configuration of embedded functions register banks 52
Table 23. SENSOR_SYNC_TIME_FRAME register. 52
Table 24. SENSOR_SYNC_TIME_FRAME register description 52
Table 25. SENSOR_SYNC_RES_RATIO register 53
Table 26. SENSOR_SYNC_RES_RATIO register description. 53
Table 27. FIFO_CTRL1 register 53
Table 28. FIFO_CTRL1 register description. 53
Table 29. FIFO_CTRL2 register 54
Table 30. FIFO_CTRL2 register description. 54
Table 31. FIFO_CTRL3 register 55
Table 32. FIFO_CTRL3 register description. 55
Table 33. Gyro FIFO decimation setting. 55
Table 34. Accelerometer FIFO decimation setting 55
Table 35. FIFO_CTRL4 register 56
Table 36. FIFO_CTRL4 register description. 56
Table 37. Fourth FIFO data set decimation setting. 56
Table 38. Third FIFO data set decimation setting. 56
Table 39. FIFO_CTRL5 register 57
Table 40. FIFO_CTRL5 register description. 57
Table 41. FIFO ODR selection 57
Table 42. FIFO mode selection. 57
Table 43. DRDY_PULSE_CFG_G register. 58
Table 44. DRDY_PULSE_CFG_G register description 58
Table 45. INT1_CTRL register 58
Table 46. INT1_CTRL register description 58
Table 47. INT2_CTRL register 59
Table 48. INT2_CTRL register description 59

Table 49. WHO_AM_I register . . . 59
Table 50. CTRL1_XL register . . . 60
Table 51. CTRL1_XL register description . . . 60
Table 52. Accelerometer ODR register setting . . . 60
Table 53. CTRL2_G register . . . 61
Table 54. CTRL2_G register description . . . 61
Table 55. Gyroscope ODR configuration setting . . . 61
Table 56. CTRL3_C register . . . 62
Table 57. CTRL3_C register description . . . 62
Table 58. CTRL4_C register . . . 63
Table 59. CTRL4_C register description . . . 63
Table 60. CTRL5_C register . . . 63
Table 61. CTRL5_C register description . . . 63
Table 62. Output registers rounding pattern . . . 64
Table 63. Angular rate sensor self-test mode selection . . . 64
Table 64. Linear acceleration sensor self-test mode selection . . . 64
Table 65. CTRL6_C register . . . 65
Table 66. CTRL6_C register description . . . 65
Table 67. Trigger mode selection . . . 65
Table 68. Gyroscope LPF1 bandwidth selection . . . 65
Table 69. CTRL7_G register . . . 66
Table 70. CTRL7_G register description . . . 66
Table 71. CTRL8_XL register . . . 66
Table 72. CTRL8_XL register description . . . 66
Table 73. Accelerometer bandwidth selection . . . 67
Table 74. CTRL9_XL register . . . 67
Table 75. CTRL9_XL register description . . . 67
Table 76. CTRL10_C register . . . 68
Table 77. CTRL10_C register description . . . 68
Table 78. MASTER_CONFIG register . . . 68
Table 79. MASTER_CONFIG register description . . . 69
Table 80. WAKE_UP_SRC register . . . 69
Table 81. WAKE_UP_SRC register description . . . 69
Table 82. TAP_SRC register . . . 70
Table 83. TAP_SRC register description . . . 70
Table 84. D6D_SRC register . . . 71
Table 85. D6D_SRC register description . . . 71
Table 86. STATUS_REG register . . . 71
Table 87. STATUS_REG register description . . . 71
Table 88. OUT_TEMP_L register . . . 72
Table 89. OUT_TEMP_H register . . . 72
Table 90. OUT_TEMP register description . . . 72
Table 91. OUTX_L_G register . . . 72
Table 92. OUTX_L_G register description . . . 72
Table 93. OUTX_H_G register . . . 72
Table 94. OUTX_H_G register description . . . 72
Table 95. OUTY_L_G register . . . 73
Table 96. OUTY_L_G register description . . . 73
Table 97. OUTY_H_G register . . . 73
Table 98. OUTY_H_G register description . . . 73
Table 99. OUTZ_L_G register . . . 73
Table 100. OUTZ_L_G register description . . . 73

Table 101. OUTZ_H_G register 74
Table 102. OUTZ_H_G register description 74
Table 103. OUTX_L_XL register. 74
Table 104. OUTX_L_XL register description 74
Table 105. OUTX_H_XL register 74
Table 106. OUTX_H_XL register description 74
Table 107. OUTY_L_XL register. 74
Table 108. OUTY_L_XL register description 74
Table 109. OUTY_H_G register 75
Table 110. OUTY_H_G register description 75
Table 111. OUTZ_L_XL register. 75
Table 112. OUTZ_L_XL register description 75
Table 113. OUTZ_H_XL register 75
Table 114. OUTZ_H_XL register description 75
Table 115. SENSORHUB1_REG register 75
Table 116. SENSORHUB1_REG register description 75
Table 117. SENSORHUB2_REG register 76
Table 118. SENSORHUB2_REG register description 76
Table 119. SENSORHUB3_REG register 76
Table 120. SENSORHUB3_REG register description 76
Table 121. SENSORHUB4_REG register 76
Table 122. SENSORHUB4_REG register description 76
Table 123. SENSORHUB5_REG register 76
Table 124. SENSORHUB5_REG register description 76
Table 125. SENSORHUB6_REG register 77
Table 126. SENSORHUB6_REG register description 77
Table 127. SENSORHUB7_REG register 77
Table 128. SENSORHUB7_REG register description 77
Table 129. SENSORHUB8_REG register 77
Table 130. SENSORHUB8_REG register description 77
Table 131. SENSORHUB9_REG register 77
Table 132. SENSORHUB9_REG register description 77
Table 133. SENSORHUB10_REG register 78
Table 134. SENSORHUB10_REG register description 78
Table 135. SENSORHUB11_REG register 78
Table 136. SENSORHUB11_REG register description 78
Table 137. SENSORHUB12_REG register 78
Table 138. SENSORHUB12_REG register description 78
Table 139. FIFO_STATUS1 register 78
Table 140. FIFO_STATUS1 register description 78
Table 141. FIFO_STATUS2 register 79
Table 142. FIFO_STATUS2 register description 79
Table 143. FIFO_STATUS3 register 79
Table 144. FIFO_STATUS3 register description 79
Table 145. FIFO_STATUS4 register 80
Table 146. FIFO_STATUS4 register description 80
Table 147. FIFO_DATA_OUT_L register 80
Table 148. FIFO_DATA_OUT_L register description 80
Table 149. FIFO_DATA_OUT_H register. 80
Table 150. FIFO_DATA_OUT_H register description. 80
Table 151. TIMESTAMP0_REG register 81
Table 152. TIMESTAMP0_REG register description 81

Table 153. TIMESTAMP1_REG register . . . 81
Table 154. TIMESTAMP1_REG register description . . . 81
Table 155. TIMESTAMP2_REG register . . . 81
Table 156. TIMESTAMP2_REG register description . . . 81
Table 157. STEP_TIMESTAMP_L register. . . . 82
Table 158. STEP_TIMESTAMP_L register description . . . 82
Table 159. STEP_TIMESTAMP_H register . . . 82
Table 160. STEP_TIMESTAMP_H register description . . . 82
Table 161. STEP_COUNTER_L register . . . 82
Table 162. STEP_COUNTER_L register description . . . 82
Table 163. STEP_COUNTER_H register . . . 83
Table 164. STEP_COUNTER_H register description. . . . 83
Table 165. SENSORHUB13_REG register . . . 83
Table 166. SENSORHUB13_REG register description . . . 83
Table 167. SENSORHUB14_REG register . . . 83
Table 168. SENSORHUB14_REG register description . . . 83
Table 169. SENSORHUB15_REG register . . . 83
Table 170. SENSORHUB15_REG register description . . . 83
Table 171. SENSORHUB16_REG register . . . 84
Table 172. SENSORHUB16_REG register description . . . 84
Table 173. SENSORHUB17_REG register . . . 84
Table 174. SENSORHUB17_REG register description . . . 84
Table 175. SENSORHUB18_REG register . . . 84
Table 176. SENSORHUB18_REG register description . . . 84
Table 177. FUNC_SRC1 register . . . 85
Table 178. FUNC_SRC1 register description. . . . 85
Table 179. FUNC_SRC2 register . . . 85
Table 180. FUNC_SRC2 register description. . . . 85
Table 181. WRIST_TILT_IA register. . . . 86
Table 182. WRIST_TILT_IA register description . . . 86
Table 183. TAP_CFG register . . . 87
Table 184. TAP_CFG register description . . . 87
Table 185. TAP_THS_6D register . . . 88
Table 186. TAP_THS_6D register description . . . 88
Table 187. Threshold for D4D/D6D function. . . . 88
Table 188. INT_DUR2 register . . . 88
Table 189. INT_DUR2 register description. . . . 88
Table 190. WAKE_UP_THS register . . . 89
Table 191. WAKE_UP_THS register description . . . 89
Table 192. WAKE_UP_DUR register . . . 89
Table 193. WAKE_UP_DUR register description . . . 89
Table 194. FREE_FALL register. . . . 90
Table 195. FREE_FALL register description . . . 90
Table 196. Threshold for free-fall function . . . 90
Table 197. MD1_CFG register . . . 91
Table 198. MD1_CFG register description . . . 91
Table 199. MD2_CFG register . . . 92
Table 200. MD2_CFG register description . . . 92
Table 201. MASTER_CMD_CODE register . . . 93
Table 202. MASTER_CMD_CODE register description. . . . 93
Table 203. SENS_SYNC_SPI_ERROR_CODE register . . . 93
Table 204. SENS_SYNC_SPI_ERROR_CODE register description . . . 93

Table 205. OUT_MAG_RAW_X_L register . . . 93
Table 206. OUT_MAG_RAW_X_L register description . . . 93
Table 207. OUT_MAG_RAW_X_H register . . . 93
Table 208. OUT_MAG_RAW_X_H register description . . . 93
Table 209. OUT_MAG_RAW_Y_L register . . . 94
Table 210. OUT_MAG_RAW_Y_L register description . . . 94
Table 211. OUT_MAG_RAW_Y_H register . . . 94
Table 212. OUT_MAG_RAW_Y_H register description . . . 94
Table 213. OUT_MAG_RAW_Z_L register. . . . 94
Table 214. OUT_MAG_RAW_Z_L register description . . . 94
Table 215. OUT_MAG_RAW_Z_H register . . . 94
Table 216. OUT_MAG_RAW_Z_H register description . . . 94
Table 217. X_OFS_USR register . . . 95
Table 218. X_OFS_USR register description . . . 95
Table 219. Y_OFS_USR register . . . 95
Table 220. Y_OFS_USR register description . . . 95
Table 221. Z_OFS_USR register . . . 95
Table 222. Z_OFS_USR register description . . . 95
Table 223. Register address map - Bank A - embedded functions . . . 96
Table 224. Register address map - Bank B - embedded functions . . . 97
Table 225. SLV0_ADD register. . . . 98
Table 226. SLV0_ADD register description . . . 98
Table 227. SLV0_SUBADD register . . . 98
Table 228. SLV0_SUBADD register description. . . . 98
Table 229. SLAVE0_CONFIG register . . . 98
Table 230. SLAVE0_CONFIG register description. . . . 99
Table 231. SLV1_ADD register. . . . 99
Table 232. SLV1_ADD register description . . . 99
Table 233. SLV1_SUBADD register . . . 99
Table 234. SLV1_SUBADD register description. . . . 99
Table 235. SLAVE1_CONFIG register . . . 100
Table 236. SLAVE1_CONFIG register description. . . . 100
Table 237. SLV2_ADD register. . . . 100
Table 238. SLV2_ADD register description . . . 100
Table 239. SLV2_SUBADD register . . . 100
Table 240. SLV2_SUBADD register description. . . . 100
Table 241. SLAVE2_CONFIG register . . . 101
Table 242. SLAVE2_CONFIG register description. . . . 101
Table 243. SLV3_ADD register. . . . 101
Table 244. SLV3_ADD register description . . . 101
Table 245. SLV3_SUBADD register . . . 101
Table 246. SLV3_SUBADD register description. . . . 101
Table 247. SLAVE3_CONFIG register . . . 102
Table 248. SLAVE3_CONFIG register description. . . . 102
Table 249. DATAWRITE_SRC_MODE_SUB_SLV0 register . . . 102
Table 250. DATAWRITE_SRC_MODE_SUB_SLV0 register description. . . . 102
Table 251. CONFIG_PEDO_THS_MIN register. . . . 102
Table 252. CONFIG_PEDO_THS_MIN register description. . . . 102
Table 253. SM_THS register . . . 103
Table 254. SM_THS register description . . . 103
Table 255. PEDO_DEB_REG register default values . . . 103
Table 256. PEDO_DEB_REG register description . . . 103

Table 257. STEP_COUNT_DELTA register 103
Table 258. STEP_COUNT_DELTA register description 103
Table 259. MAG_SI_XX register. 104
Table 260. MAG_SI_XX register description 104
Table 261. MAG_SI_XY register. 104
Table 262. MAG_SI_XY register description 104
Table 263. MAG_SI_XZ register. 104
Table 264. MAG_SI_XZ register description 104
Table 265. MAG_SI_YX register. 104
Table 266. MAG_SI_YX register description 104
Table 267. MAG_SI_YY register. 105
Table 268. MAG_SI_YY register description 105
Table 269. MAG_SI_YZ register. 105
Table 270. MAG_SI_YZ register description 105
Table 271. MAG_SI_ZX register. 105
Table 272. MAG_SI_ZX register description 105
Table 273. MAG_SI_ZY register. 105
Table 274. MAG_SI_ZY register description 105
Table 275. MAG_SI_ZZ register. 106
Table 276. MAG_SI_ZZ register description. 106
Table 277. MAG_OFFX_L register 106
Table 278. MAG_OFFX_L register description. 106
Table 279. MAG_OFFX_H register. 106
Table 280. MAG_OFFX_H register description 106
Table 281. MAG_OFFY_L register 106
Table 282. MAG_OFFY_L register description. 106
Table 283. MAG_OFFY_H register. 107
Table 284. MAG_OFFY_H register description 107
Table 285. MAG_OFFZ_L register 107
Table 286. MAG_OFFZ_L register description. 107
Table 287. MAG_OFFZ_H register. 107
Table 288. MAG_OFFZ_H register description 107
Table 289. A_WRIST_TILT_LAT register. 108
Table 290. A_WRIST_TILT_LAT register description. 108
Table 291. A_WRIST_TILT_THS register 108
Table 292. A_WRIST_TILT_THS register description 108
Table 293. A_WRIST_TILT_Mask register. 108
Table 294. A_WRIST_TILT_Mask register description. 108
Table 295. Reel dimensions for carrier tape of LGA-14 package 112
Table 296. Document revision history. 113

List of figures

Figure 1. Pin connections . . . 18
Figure 2. LSM6DSL connection modes . . . 19
Figure 3. SPI slave timing diagram (in mode 3) . . . 26
Figure 4. I^2C timing diagram . . . 27
Figure 5. Block diagram of filters . . . 32
Figure 6. Gyroscope digital chain - Mode 1 (UI/EIS) and Mode 2 . . . 32
Figure 7. Accelerometer chain . . . 33
Figure 8. Accelerometer composite filter . . . 33
Figure 9. Read and write protocol (in mode 3) . . . 40
Figure 10. SPI read protocol (in mode 3) . . . 41
Figure 11. Multiple byte SPI read protocol (2-byte example) (in mode 3) . . . 41
Figure 12. SPI write protocol (in mode 3) . . . 42
Figure 13. Multiple byte SPI write protocol (2-byte example) (in mode 3) . . . 42
Figure 14. SPI read protocol in 3-wire mode (in mode 3) . . . 43
Figure 15. LSM6DSL electrical connections in Mode 1 . . . 44
Figure 16. LSM6DSL electrical connections in Mode 2 . . . 45
Figure 17. LGA-14 2.5x3x0.86 mm package outline and mechanical data . . . 110
Figure 18. Carrier tape information for LGA-14 package . . . 111
Figure 19. LGA-14 package orientation in carrier tape . . . 111
Figure 20. Reel information for carrier tape of LGA-14 package . . . 112

1 Overview

The LSM6DSL is a system-in-package featuring a high-performance 3-axis digital accelerometer and 3-axis digital gyroscope.

The integrated power-efficient modes are able to reduce the power consumption down to 0.65 mA in high-performance mode, combining always-on low-power features with superior sensing precision for an optimal motion experience for the consumer thanks to ultra-low noise performance for both the gyroscope and accelerometer.

The LSM6DSL delivers best-in-class motion sensing that can detect orientation and gestures in order to empower application developers and consumers with features and capabilities that are more sophisticated than simply orienting their devices to portrait and landscape mode.

The event-detection interrupts enable efficient and reliable motion tracking and contextual awareness, implementing hardware recognition of free-fall events, 6D orientation, click and double-click sensing, activity or inactivity, and wakeup events.

The LSM6DSL supports main OS requirements, offering real, virtual and batch mode sensors. In addition, the LSM6DSL can efficiently run the sensor-related features specified in Android, saving power and enabling faster reaction time. In particular, the LSM6DSL has been designed to implement hardware features such as significant motion detection, tilt, pedometer functions, timestamping and to support the data acquisition of an external magnetometer with ironing correction (hard, soft).

The LSM6DSL offers hardware flexibility to connect the pins with different mode connections to external sensors to expand functionalities such as adding a sensor hub, etc.

Up to 4 kbyte of FIFO with dynamic allocation of significant data (i.e. external sensors, timestamp, etc.) allows overall power saving of the system.

Like the entire portfolio of MEMS sensor modules, the LSM6DSL leverages the robust and mature in-house manufacturing processes already used for the production of micromachined accelerometers and gyroscopes. The various sensing elements are manufactured using specialized micromachining processes, while the IC interfaces are developed using CMOS technology that allows the design of a dedicated circuit which is trimmed to better match the characteristics of the sensing element.

The LSM6DSL is available in a small plastic land grid array (LGA) package of 2.5 x 3.0 x 0.83 mm to address ultra-compact solutions.

2 Embedded low-power features

The LSM6DSL has been designed to be fully compliant with Android, featuring the following on-chip functions:

- 4 kbyte data buffering
  - 100% efficiency with flexible configurations and partitioning
  - Possibility to store timestamp
- Event-detection interrupts (fully configurable):
  - Free-fall
  - Wakeup
  - 6D orientation
  - Click and double-click sensing
  - Activity / inactivity recognition
- Specific IP blocks with negligible power consumption and high-performance:
  - Pedometer functions: step detector and step counters
  - Tilt (refer to *Section 2.1: Tilt detection* for additional information
  - Absolute Wrist Tilt (refer to *Section 2.2: Absolute wrist tilt* for additional information)
  - Significant Motion Detection
- Sensor hub
  - Up to 6 total sensors: 2 internal (accelerometer and gyroscope) and 4 external sensors
- Data rate synchronization with external trigger for reduced sensor access and enhanced fusion

2.1 Tilt detection

The tilt function helps to detect activity change and has been implemented in hardware using only the accelerometer to achieve both the targets of ultra-low power consumption and robustness during the short duration of dynamic accelerations.

It is based on a trigger of an event each time the device's tilt changes. For a more customized user experience, in the LSM6DSL the tilt function is configurable through:

- a programmable average window
- a programmable average threshold

The tilt function can be used with different scenarios, for example:

a) Triggers when phone is in a front pants pocket and the user goes from sitting to standing or standing to sitting;

b) Doesn’t trigger when phone is in a front pants pocket and the user is walking, running or going upstairs.

2.2 Absolute wrist tilt

The LSM6DSL implements in hardware the Absolute Wrist Tilt (AWT) function which allows detecting when the angle between a selectable accelerometer semi-axis and the horizontal plane becomes higher than a specific user-selectable value.

Configurable threshold and latency parameters are associated with the AWT function: the threshold parameter defines the amplitude of the tilt angle; the latency parameter defines the minimum duration of the AWT event to be recognized. The AWT interrupt signal is generated if the tilt angle is higher than the threshold angle for a period of time equal to or greater than the latency period.

The AWT function is based on the accelerometer sensor only and works at 26 Hz, so the accelerometer ODR must be set at a value of 26 Hz or higher.

By default, the AWT algorithm is applied to the positive X-axis.

In order to enable the AWT function it is necessary to set to 1 both the FUNC_EN bit and the WRIST_TILT_EN bit of *CTRL10_C (19h)*.

The AWT interrupt signal can be driven to the INT2 interrupt pin by setting to 1 the INT2_WRIST_TILT bit of the *DRDY_PULSE_CFG_G (0Bh)* register; it can also be checked by reading the WRIST_TILT_IA bit of the *FUNC_SRC2 (54h)* register (it will also clear the interrupt signal if latched).

WRIST_TILT_IA (55h) is the status register to be used to detect which axis has triggered the AWT event (not applicable when using one axis side only).

The full description and an example is given in the dedicated application note.

3 Pin description

Figure 1. Pin connections

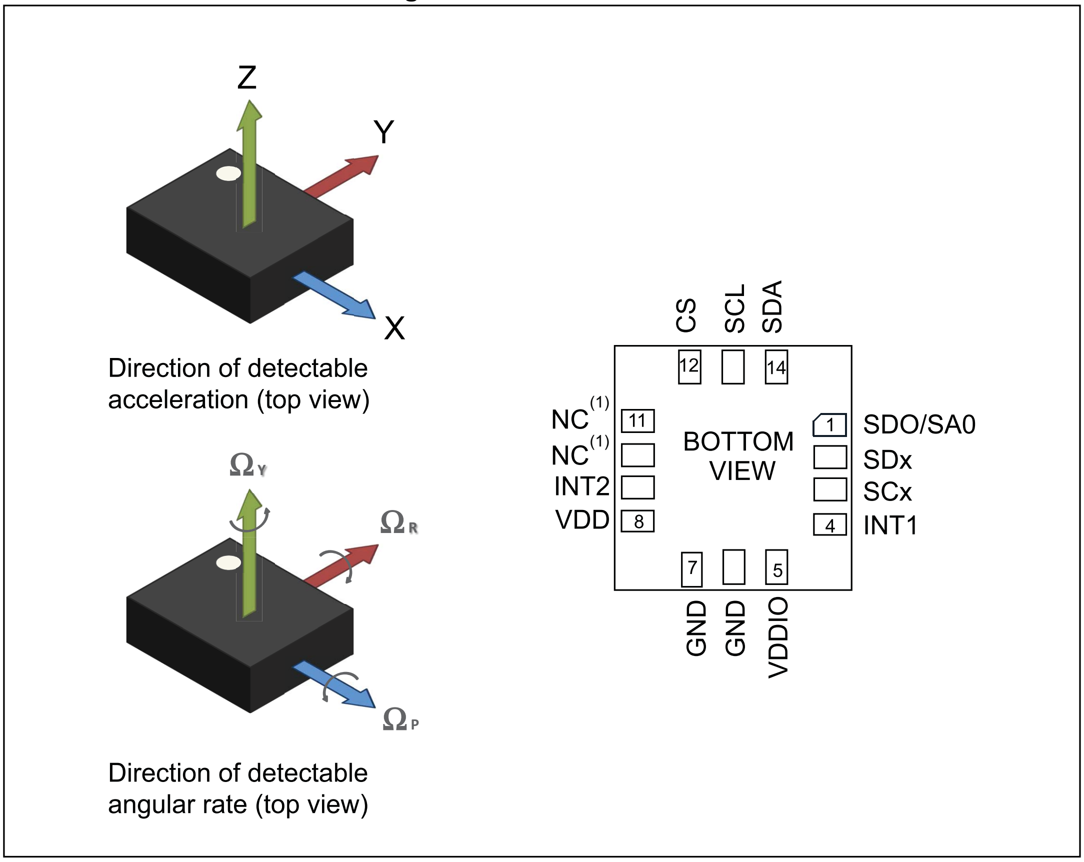

1. Leave pin electrically unconnected and soldered to PCB.

3.1 Pin connections

The LSM6DSL offers flexibility to connect the pins in order to have two different mode connections and functionalities. In detail:

- **Mode 1**: I^2C slave interface or SPI (3- and 4-wire) serial interface is available;
- **Mode 2**: I^2C slave interface or SPI (3- and 4-wire) serial interface and I^2C interface master for external sensor connections are available;

Figure 2. LSM6DSL connection modes

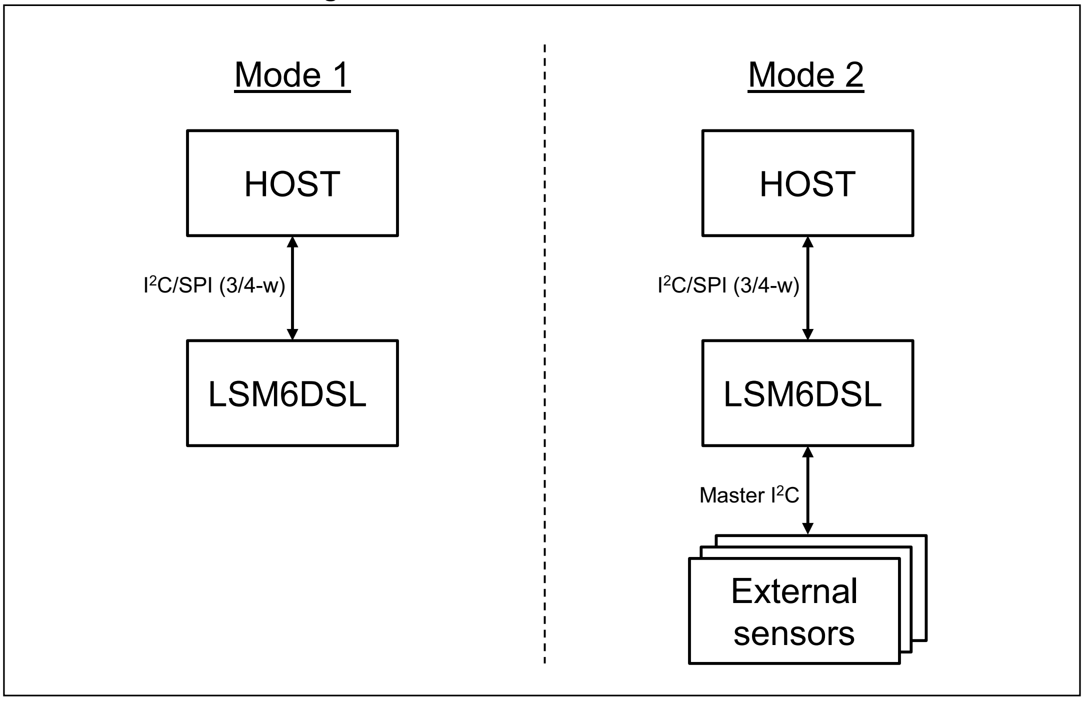

In the following table each mode is described for the pin connections and function.

Table 2. Pin description

Pin#	Name	Mode 1 function	Mode 2 function
1	SDO/SA0	SPI 4-wire interface serial data output (SDO)<br>I^2C least significant bit of the device address (SA0)	SPI 4-wire interface serial data output (SDO)<br>I^2C least significant bit of the device address (SA0)
2	SDx	Connect to VDDIO or GND	I^2C serial data master (MSDA)
3	SCx	Connect to VDDIO or GND	I^2C serial clock master (MSCL)
4	INT1	Programmable interrupt 1	
5	VDDIO(1)	Power supply for I/O pins	
6	GND	0 V supply	
7	GND	0 V supply	
8	VDD(1)	Power supply	
9	INT2	Programmable interrupt 2 (INT2) / Data enable (DEN)	Programmable interrupt 2 (INT2)/ Data enable (DEN)/<br>I^2C master external synchronization signal (MDRDY)
10	NC(2)	Leave unconnected	
11	NC(2)	Leave unconnected	
12	CS	I^2C/SPI mode selection<br>(1: SPI idle mode / I^2C communication enabled; 0: SPI communication mode / I^2C disabled)	I^2C/SPI mode selection<br>(1: SPI idle mode / I^2C communication enabled;<br>0: SPI communication mode / I^2C disabled)
13	SCL	I^2C serial clock (SCL)<br>SPI serial port clock (SPC)	I^2C serial clock (SCL)<br>SPI serial port clock (SPC)
14	SDA	I^2C serial data (SDA)<br>SPI serial data input (SDI)<br>3-wire interface serial data output (SDO)	I^2C serial data (SDA)<br>SPI serial data input (SDI)<br>3-wire interface serial data output (SDO)

1. Recommended 100 nF filter capacitor.
2. Leave pin electrically unconnected and soldered to PCB.

4 Module specifications

4.1 Mechanical characteristics

@ Vdd = 1.8 V, T = 25 °C unless otherwise noted.

Table 3. Mechanical characteristics

Symbol	Parameter	Test conditions	Min.	Typ.(1)	Max.	Unit
LA_FS	Linear acceleration measurement range			±2		*g*
				±4		
				±8		
				±16		
G_FS	Angular rate measurement range			±125		dps
				±250		
				±500		
				±1000		
				±2000		
LA_So	Linear acceleration sensitivity(2)	FS = ±2		0.061		m*g*/LSB
		FS = ±4		0.122		
		FS = ±8		0.244		
		FS = ±16		0.488		
G_So	Angular rate sensitivity(2)	FS = ±125		4.375		mdps/LSB
		FS = ±250		8.75		
		FS = ±500		17.50		
		FS = ±1000		35		
		FS = ±2000		70		
G_So%	Sensitivity tolerance(3)	at component level		±1		%
LA_SoDr	Linear acceleration sensitivity change vs. temperature(4)	from -40° to +85°		±0.01		%/°C
G_SoDr	Angular rate sensitivity change vs. temperature(4)	from -40° to +85°		±0.007		%/°C
LA_TyOff	Linear acceleration zero-*g* level offset accuracy(5)			±40		m*g*
G_TyOff	Angular rate zero-rate level(4)			±3		dps
LA_OffDr	Linear acceleration zero-*g* level change vs. temperature(4)			±0.1		m*g*/ °C
G_OffDr	Angular rate typical zero-rate level change vs. temperature(4)			±0.015		dps/°C
Rn	Rate noise density in high-performance mode(6)			4		mdps/√Hz

Table 3. Mechanical characteristics (continued)

Symbol	Parameter	Test conditions	Min.	Typ.[1]	Max.	Unit
RnRMS	Gyroscope RMS noise in normal/low-power mode[7]			75		mdps
An	Acceleration noise density in high-performance mode[8]	FS = ±2 *g*		80		μ*g*/√Hz
		FS = ±4 *g*		80		
		FS = ±8 *g*		90		
		FS = ±16 *g*		130		
RMS	Acceleration RMS noise in normal/low-power mode[9][10]	FS = ±2 *g*		1.8		m*g*(RMS)
		FS = ±4 *g*		2.0		
		FS = ±8 *g*		2.4		
		FS = ±16 *g*		3.0		
LA_ODR	Linear acceleration output data rate			1.6[11]<br>12.5<br>26<br>52<br>104<br>208<br>416<br>833<br>1666<br>3332<br>6664		Hz
G_ODR	Angular rate output data rate			12.5<br>26<br>52<br>104<br>208<br>416<br>833<br>1666<br>3332<br>6664		
Vst	Linear acceleration self-test output change[12][13][14]		90		1700	m*g*
	Angular rate self-test output change[15][16]	FS = 250 dps	20		80	dps
		FS = 2000 dps	150		700	dps
Top	Operating temperature range		-40		+85	°C

1. Typical specifications are not guaranteed.
2. Sensitivity values after factory calibration test and trimming
3. Subject to change.
4. Measurements are performed in a uniform temperature setup and they are based on characterization data in a limited number of samples. Not measured during final test for production.
5. Values after factory calibration test and trimming.

6. Gyroscope rate noise density in high-performance mode is independent of the ODR and FS setting.
7. Gyroscope RMS noise in normal/low-power mode is independent of the ODR and FS setting.
8. Accelerometer noise density in high-performance mode is independent of the ODR.
9. Accelerometer RMS noise in normal/low-power mode is independent of the ODR.
10. Noise RMS related to BW = ODR /2 (for ODR /9, typ value can be calculated by Typ *0.6).
11. This ODR is available when accelerometer is in low-power mode.
12. The sign of the linear acceleration self-test output change is defined by the STx_XL bits in *CTRL5_C (14h)*, *Table 64* for all axes.
13. The linear acceleration self-test output change is defined with the device in stationary condition as the absolute value of: OUTPUT[LSb] (self-test enabled) - OUTPUT[LSb] (self-test disabled). 1LSb = 0.061 m*g* at ±2 *g* full scale.
14. Accelerometer self-test limits are full-scale independent.
15. The sign of the angular rate self-test output change is defined by the STx_G bits in *CTRL5_C (14h)*, *Table 63* for all axes.
16. The angular rate self-test output change is defined with the device in stationary condition as the absolute value of: OUTPUT[LSb] (self-test enabled) - OUTPUT[LSb] (self-test disabled). 1LSb = 70 mdps at ±2000 dps full scale.

4.2 Electrical characteristics

@ Vdd = 1.8 V, T = 25 °C unless otherwise noted.

Table 4. Electrical characteristics

Symbol	Parameter	Test conditions	Min.	Typ.(1)	Max.	Unit
Vdd	Supply voltage		1.71	1.8	3.6	V
Vdd_IO	Power supply for I/O		1.62		3.6	V
IddHP	Gyroscope and accelerometer current consumption in high-performance mode	ODR = 1.6 kHz		0.65		mA
IddNM	Gyroscope and accelerometer current consumption in normal mode	ODR = 208 Hz		0.45		mA
IddLP	Gyroscope and accelerometer current consumption in low-power mode	ODR = 52 Hz		0.29		mA
LA_IddHP	Accelerometer current consumption in high-performance mode	ODR < 1.6 kHz<br>ODR ≥ 1.6 kHz		150<br>160		µA
LA_IddNM	Accelerometer current consumption in normal mode	ODR = 208 Hz		85		µA
LA_IddLM	Accelerometer current consumption in low-power mode	ODR = 52 Hz<br>ODR = 12.5 Hz<br>ODR = 1.6 Hz		25<br>9<br>4.5		µA
IddPD	Gyroscope and accelerometer current consumption during power-down			3		µA
Ton	Turn-on time			35		ms
V_{IH}	Digital high-level input voltage		0.7 *VDD_IO			V
V_{IL}	Digital low-level input voltage				0.3 *VDD_IO	V
V_{OH}	High-level output voltage	I_{OH} = 4 mA (2)	VDD_IO - 0.2			V
V_{OL}	Low-level output voltage	I_{OL} = 4 mA (2)			0.2	V
Top	Operating temperature range		-40		+85	°C

1. Typical specifications are not guaranteed.
2. 4 mA is the maximum driving capability, i.e. the maximum DC current that can be sourced/sunk by the digital pad in order to guarantee the correct digital output voltage levels V_{OH} and V_{OL}.

4.3 Temperature sensor characteristics

@ Vdd = 1.8 V, T = 25 °C unless otherwise noted.

Table 5. Temperature sensor characteristics

Symbol	Parameter	Test condition	Min.	Typ.(1)	Max.	Unit
TODR(2)	Temperature refresh rate			52		Hz
Toff	Temperature offset(3)		-15		+15	°C
TSen	Temperature sensitivity			256		LSB/°C
TST	Temperature stabilization time(4)				500	µs
T_ADC_res	Temperature ADC resolution			16		bit
Top	Operating temperature range		-40		+85	°C

1. Typical specifications are not guaranteed.
2. When the accelerometer is in Low-Power mode and the gyroscope part is turned off, the TODR value is equal to the accelerometer ODR.
3. The output of the temperature sensor is 0 LSB (typ.) at 25 °C.
4. Time from power ON bit to valid data based on characterization data.

4.4 Communication interface characteristics

4.4.1 SPI - serial peripheral interface

Subject to general operating conditions for Vdd and Top.

Table 6. SPI slave timing values (in mode 3)

Symbol	Parameter	Value(1)		Unit
		Min	Max	
$t_{c(SPC)}$	SPI clock cycle	100		ns
$f_{c(SPC)}$	SPI clock frequency		10	MHz
$t_{su(CS)}$	CS setup time	5		ns
$t_{h(CS)}$	CS hold time	20		
$t_{su(SI)}$	SDI input setup time	5		
$t_{h(SI)}$	SDI input hold time	15		
$t_{v(SO)}$	SDO valid output time		50	
$t_{h(SO)}$	SDO output hold time	5		
$t_{dis(SO)}$	SDO output disable time		50	

1. Values are guaranteed at 10 MHz clock frequency for SPI with both 4 and 3 wires, based on characterization results, not tested in production

Figure 3. SPI slave timing diagram (in mode 3)

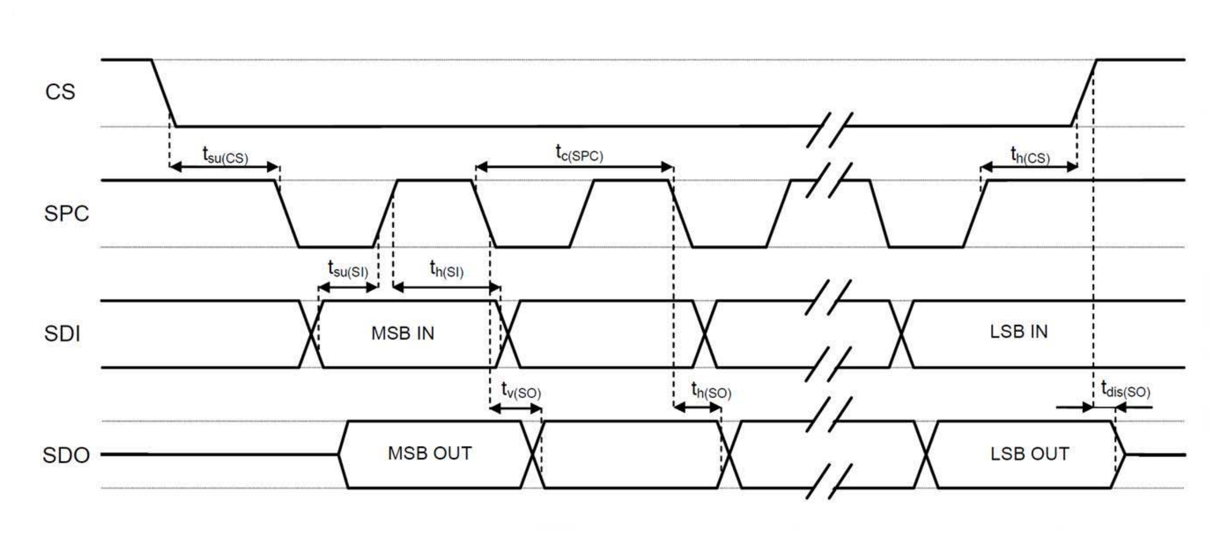

Note: Measurement points are done at 0.2·Vdd_IO and 0.8·Vdd_IO, for both input and output ports.

4.4.2 I^2C - inter-IC control interface

Subject to general operating conditions for Vdd and Top.

Figure 4. I^2C timing diagram

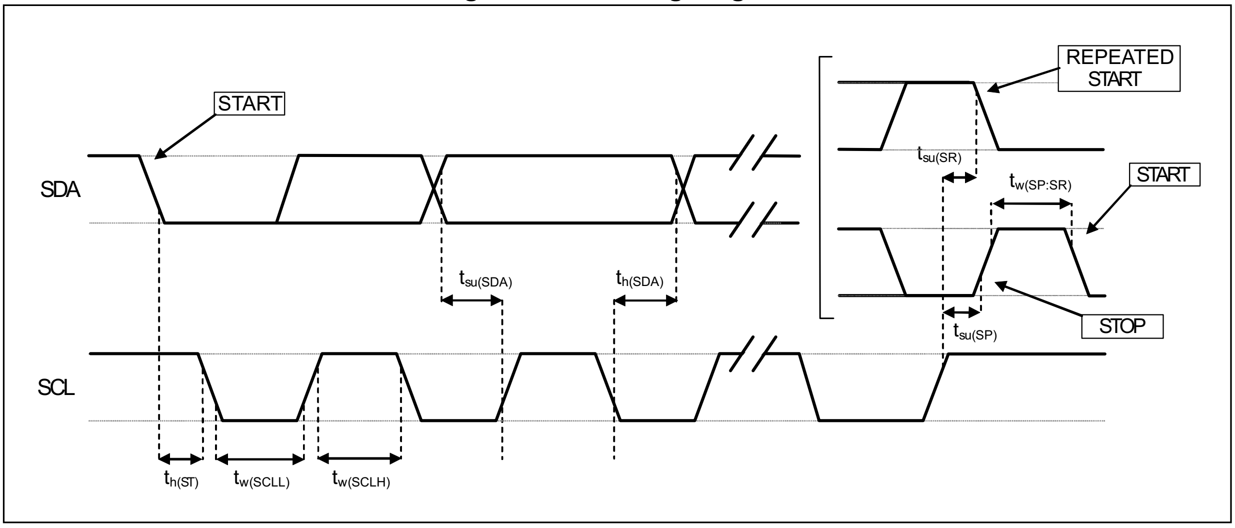

Note: Measurement points are done at 0.2·Vdd_IO and 0.8·Vdd_IO, for both ports.

4.4.2.1 I^2C slave

Table 7. I^2C slave timing values

Symbol	Parameter	I^2C standard mode(1)		I^2C fast mode (1)		Unit
		Min	Max	Min	Max	
$f_{(SCL)}$	SCL clock frequency	0	100	0	400	kHz
$t_{w(SCLL)}$	SCL clock low time	4.7		1.3		µs
$t_{w(SCLH)}$	SCL clock high time	4.0		0.6		
$t_{su(SDA)}$	SDA setup time	250		100		ns
$t_{h(SDA)}$	SDA data hold time	0	3.45	0	0.9	µs
$t_{h(ST)}$	START condition hold time	4		0.6		µs
$t_{su(SR)}$	Repeated START condition setup time	4.7		0.6		
$t_{su(SP)}$	STOP condition setup time	4		0.6		
$t_{w(SP:SR)}$	Bus free time between STOP and START condition	4.7		1.3		

1. Data based on standard I^2C protocol requirement, not tested in production.

4.4.2.2 I^2C master

When in I^2C Master Mode, an external sensor can be connected to LSM6DSL. LSM6DSL supports I^2C Master - Fast Mode only.

Table 8. I^2C master timing values

Symbol	Parameter	I^2C Master	I^2C Fast Mode (min)	Unit
$f_{(SCL)}$	SCL clock frequency	116.3	0 (400 kHz max)	kHz
$t_{w(SCLL)}$	SCL clock low time	5.86	1.3	μs
$t_{w(SCLH)}$	SCL clock high time	2.74	0.6	ns
	Data valid time	3.9	-	μs
	SDA hold time	≥0	0	ns
	SDA setup time	≥100	100	ns
$t_{su(SR)}$	Repeated START condition setup time	1.56	0.6	μs
$t_{su(HD)}$	Repeated START condition hold time	1.56	0.6	μs
$t_{su(SP)}$	STOP condition setup time	2.73	0.6	μs
$t_{w(SP:SR)}$	Bus free time between STOP and START condition	21	1.3	μs

4.5 Absolute maximum ratings

Stresses above those listed as “Absolute maximum ratings” may cause permanent damage to the device. This is a stress rating only and functional operation of the device under these conditions is not implied. Exposure to maximum rating conditions for extended periods may affect device reliability.

Table 9. Absolute maximum ratings

Symbol	Ratings	Maximum value	Unit
Vdd	Supply voltage	-0.3 to 4.8	V
T_{STG}	Storage temperature range	-40 to +125	°C
Sg	Acceleration *g* for 0.2 ms	10,000	*g*
ESD	Electrostatic discharge protection (HBM)	2	kV
Vin	Input voltage on any control pin<br>(including CS, SCL/SPC, SDA/SDI/SDO, SDO/SA0)	-0.3 to Vdd_IO +0.3	V

Note: Supply voltage on any pin should never exceed 4.8 V.

This device is sensitive to mechanical shock, improper handling can cause permanent damage to the part.

This device is sensitive to electrostatic discharge (ESD), improper handling can cause permanent damage to the part.

4.6 Terminology

4.6.1 Sensitivity

Linear acceleration sensitivity can be determined, for example, by applying 1 *g* acceleration to the device. Because the sensor can measure DC accelerations, this can be done easily by pointing the selected axis towards the ground, noting the output value, rotating the sensor 180 degrees (pointing towards the sky) and noting the output value again. By doing so, ±1 *g* acceleration is applied to the sensor. Subtracting the larger output value from the smaller one, and dividing the result by 2, leads to the actual sensitivity of the sensor. This value changes very little over temperature and over time. The sensitivity tolerance describes the range of sensitivities of a large number of sensors (see *Table 3*).

An angular rate gyroscope is a device that produces a positive-going digital output for counterclockwise rotation around the axis considered. Sensitivity describes the gain of the sensor and can be determined by applying a defined angular velocity to it. This value changes very little over temperature and time (see *Table 3*).

4.6.2 Zero-*g* and zero-rate level

Linear acceleration zero-*g* level offset (TyOff) describes the deviation of an actual output signal from the ideal output signal if no acceleration is present. A sensor in a steady state on a horizontal surface will measure 0 *g* on both the X-axis and Y-axis, whereas the Z-axis will measure 1 *g*. Ideally, the output is in the middle of the dynamic range of the sensor (content of OUT registers 00h, data expressed as 2’s complement number). A deviation from the ideal value in this case is called zero-*g* offset.

Offset is to some extent a result of stress to MEMS sensor and therefore the offset can slightly change after mounting the sensor onto a printed circuit board or exposing it to extensive mechanical stress. Offset changes little over temperature, see “Linear acceleration zero-*g* level change vs. temperature” in *Table 3*. The zero-*g* level tolerance (TyOff) describes the standard deviation of the range of zero-*g* levels of a group of sensors.

Zero-rate level describes the actual output signal if there is no angular rate present. The zero-rate level of precise MEMS sensors is, to some extent, a result of stress to the sensor and therefore the zero-rate level can slightly change after mounting the sensor onto a printed circuit board or after exposing it to extensive mechanical stress. This value changes very little over temperature and time (see *Table 3*).

5 Functionality

5.1 Operating modes

In the LSM6DSL, the accelerometer and the gyroscope can be turned on/off independently of each other and are allowed to have different ODRs and power modes.

The LSM6DSL has three operating modes available:

- only accelerometer active and gyroscope in power-down
- only gyroscope active and accelerometer in power-down
- both accelerometer and gyroscope sensors active with independent ODR

The accelerometer is activated from power-down by writing ODR_XL[3:0] in *CTRL1_XL (10h)* while the gyroscope is activated from power-down by writing ODR_G[3:0] in *CTRL2_G (11h)*. For combo-mode the ODRs are totally independent.

5.2 Gyroscope power modes

In the LSM6DSL, the gyroscope can be configured in four different operating modes: power-down, low-power, normal mode and high-performance mode. The operating mode selected depends on the value of the G_HM_MODE bit in *CTRL7_G (16h)*. If G_HM_MODE is set to '0', high-performance mode is valid for all ODRs (from 12.5 Hz up to 6.66 kHz).

To enable the low-power and normal mode, the G_HM_MODE bit has to be set to '1'. Low-power mode is available for lower ODRs (12.5, 26, 52 Hz) while normal mode is available for ODRs equal to 104 and 208 Hz.

5.3 Accelerometer power modes

In the LSM6DSL, the accelerometer can be configured in four different operating modes: power-down, low-power, normal mode and high-performance mode. The operating mode selected depends on the value of the XL_HM_MODE bit in *CTRL6_C (15h)*. If XL_HM_MODE is set to '0', high-performance mode is valid for all ODRs (from 12.5 Hz up to 6.66 kHz).

To enable the low-power and normal mode, the XL_HM_MODE bit has to be set to '1'. Low-power mode is available for lower ODRs (1.6, 12.5, 26, 52 Hz) while normal mode is available for ODRs equal to 104 and 208 Hz.

5.4 Block diagram of filters

Figure 5. Block diagram of filters

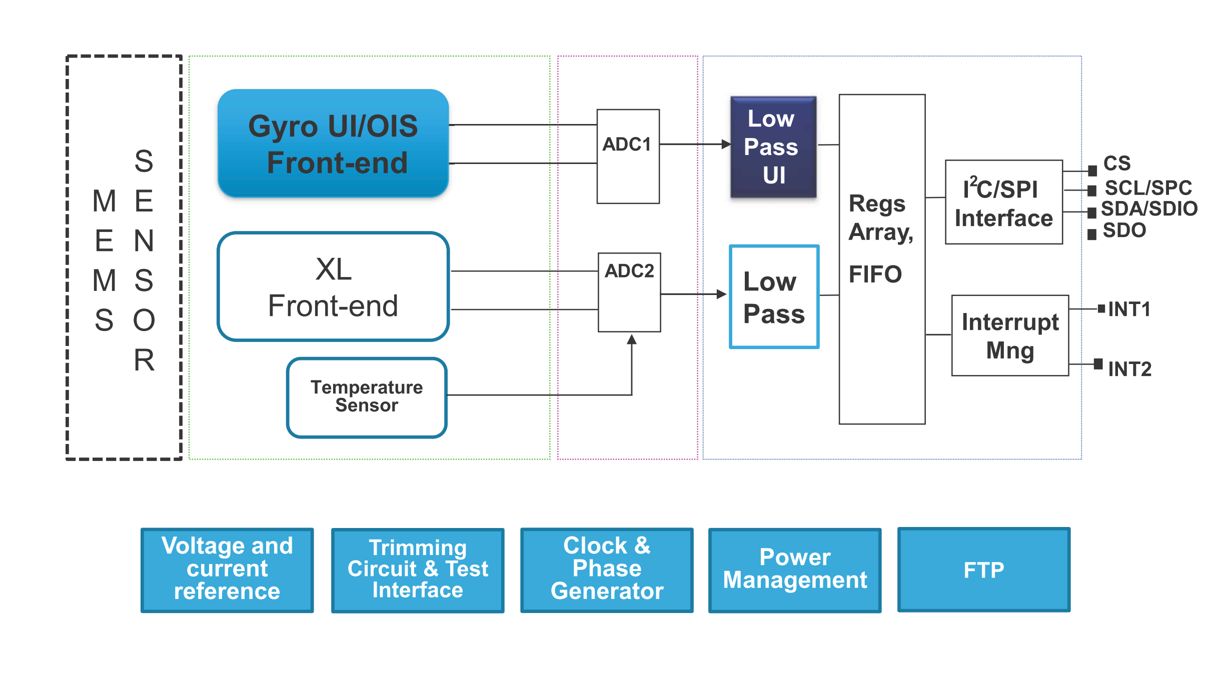

5.4.1 Block diagrams of the gyroscope filters

The gyroscope filtering configuration for both Mode 1 (for User Interface (UI) and Electronic Image Stabilization (EIS) functionality) and Mode 2 is shown in *Figure 6*.

Figure 6. Gyroscope digital chain - Mode 1 (UI/EIS) and Mode 2

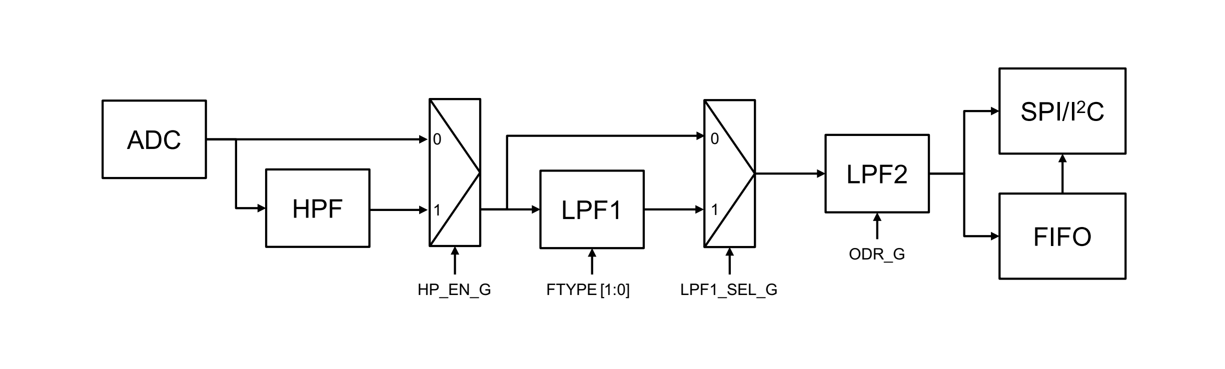

The gyroscope ODR is selectable from 12.5 Hz up to 6.66 kHz. A low-pass filter (LPF1) is available, for more details about the filter characteristics see *Table 68: Gyroscope LPF1 bandwidth selection.*

Data can be acquired from the output registers and FIFO over the I^2C/SPI interface.

5.4.2 Block diagrams of the accelerometer filters

In the LSM6DSL, the filtering chain for the accelerometer part is composed of the following:

- Analog filter (anti-aliasing)
- Digital filter (LPF1)
- Composite filter

Details of the block diagram appear in the following figure.

Figure 7. Accelerometer chain

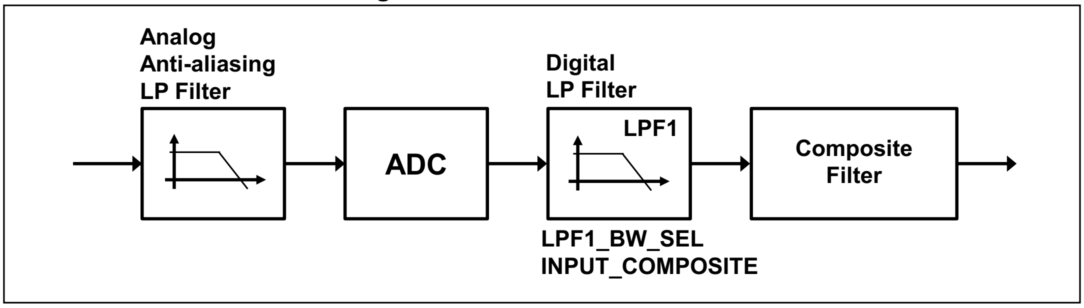

The configuration of the digital filter can be set using the LPF1_BW_SEL bit in *CTRL1_XL (10h)* and the INPUT_COMPOSITE bit in *CTRL8_XL (17h)*.

Figure 8. Accelerometer composite filter

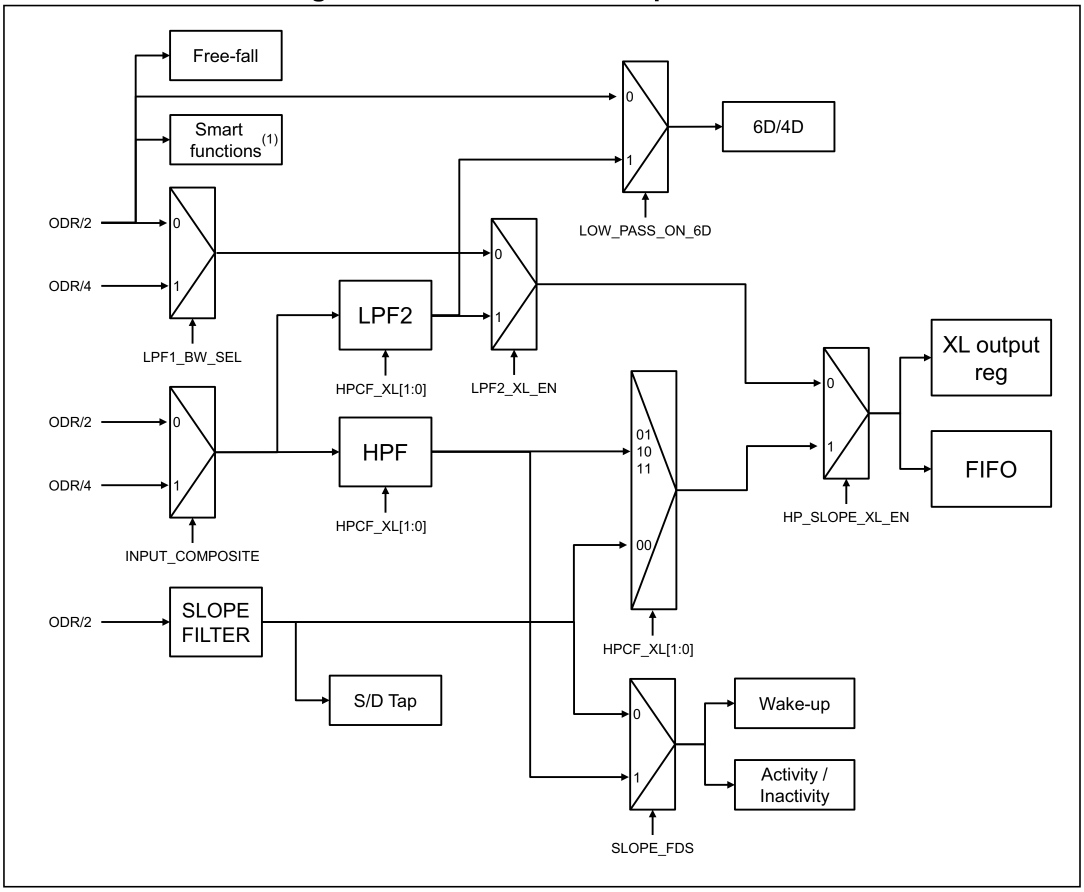

1. Pedometer, step detector and step counter, significant motion and tilt functions.

5.5 FIFO

The presence of a FIFO allows consistent power saving for the system since the host processor does not need continuously poll data from the sensor, but it can wake up only when needed and burst the significant data out from the FIFO.

The LSM6DSL embeds 4 kbytes data FIFO to store the following data:

- gyroscope
- accelerometer
- external sensors
- step counter and timestamp
- temperature

Writing data in the FIFO can be configured to be triggered by the:

- accelerometer/gyroscope data-ready signal; in which case the ODR must be lower than or equal to both the accelerometer and gyroscope ODRs;
- sensor hub data-ready signal;
- step detection signal.

In addition, each data can be stored at a decimated data rate compared to FIFO ODR and it is configurable by the user, setting the *FIFO_CTRL3 (08h)* and *FIFO_CTRL4 (09h)* registers. The available decimation factors are 2, 3, 4, 8, 16, 32.

The programmable FIFO threshold can be set in *FIFO_CTRL1 (06h)* and *FIFO_CTRL2 (07h)* using the FTH [10:0] bits.

To monitor the FIFO status, dedicated registers *FIFO_STATUS1 (3Ah)*, *FIFO_STATUS2 (3Bh)*, *FIFO_STATUS3 (3Ch)*, *FIFO_STATUS4 (3Dh)*) can be read to detect FIFO overrun events, FIFO full status, FIFO empty status, FIFO threshold status and the number of unread samples stored in the FIFO. To generate dedicated interrupts on the INT1 and INT2 pads of these status events, the configuration can be set in *INT1_CTRL (0Dh)* and *INT2_CTRL (0Eh)*.

The FIFO buffer can be configured according to five different modes:

- Bypass mode
- FIFO mode
- Continuous mode
- Continuous-to-FIFO mode
- Bypass-to-continuous mode

Each mode is selected by the FIFO_MODE_[2:0] bits in the *FIFO_CTRL5 (0Ah)* register. To guarantee the correct acquisition of data during the switching into and out of FIFO mode, the first sample acquired must be discarded.

5.5.1 Bypass mode

In Bypass mode (*FIFO_CTRL5 (0Ah)* (FIFO_MODE_[2:0] = 000), the FIFO is not operational and it remains empty.

Bypass mode is also used to reset the FIFO when in FIFO mode.

5.5.2 FIFO mode

In FIFO mode (*FIFO_CTRL5 (0Ah)* (FIFO_MODE_[2:0] = 001) data from the output channels are stored in the FIFO until it is full.

To reset FIFO content, Bypass mode should be selected by writing *FIFO_CTRL5 (0Ah)* (FIFO_MODE_[2:0]) to '000' After this reset command, it is possible to restart FIFO mode by writing *FIFO_CTRL5 (0Ah)*(FIFO_MODE_[2:0]) to '001'.

FIFO buffer memorizes up to 4096 samples of 16 bits each but the depth of the FIFO can be resized by setting the FTH [10:0] bits in *FIFO_CTRL1 (06h)* and *FIFO_CTRL2 (07h)*. If the STOP_ON_FTH bit in *FIFO_CTRL4 (09h)* is set to '1', FIFO depth is limited up to FTH [10:0] bits in *FIFO_CTRL1 (06h)* and *FIFO_CTRL2 (07h)*.

5.5.3 Continuous mode

Continuous mode (*FIFO_CTRL5 (0Ah)* (FIFO_MODE_[2:0] = 110) provides a continuous FIFO update: as new data arrives, the older data is discarded.

A FIFO threshold flag *FIFO_STATUS2 (3Bh)*(FTH) is asserted when the number of unread samples in FIFO is greater than or equal to *FIFO_CTRL1 (06h)* and *FIFO_CTRL2 (07h)*(FTH [10:0]).

It is possible to route *FIFO_STATUS2 (3Bh)*(FTH) to the INT1 pin by writing in register *INT1_CTRL (0Dh)*(INT1_FTH) = ‘1’ or to the INT2 pin by writing in register *INT2_CTRL (0Eh)*(INT2_FTH) = ‘1’.

A full-flag interrupt can be enabled, *INT1_CTRL (0Dh)*(INT_ FULL_FLAG) = '1', in order to indicate FIFO saturation and eventually read its content all at once.

If an overrun occurs, at least one of the oldest samples in FIFO has been overwritten and the OVER_RUN flag in *FIFO_STATUS2 (3Bh)* is asserted.

In order to empty the FIFO before it is full, it is also possible to pull from FIFO the number of unread samples available in *FIFO_STATUS1 (3Ah)* and *FIFO_STATUS2 (3Bh)* (DIFF_FIFO [10:0]).

5.5.4 Continuous-to-FIFO mode

In Continuous-to-FIFO mode *FIFO_CTRL5 (0Ah)*(FIFO_MODE_[2:0] = 011), FIFO behavior changes according to the trigger event detected in one of the following interrupt registers *FUNC_SRC1 (53h)*, *TAP_SRC (1Ch)*, *WAKE_UP_SRC (1Bh)* and *D6D_SRC (1Dh)*.

When the selected trigger bit is equal to '1', FIFO operates in FIFO mode.

When the selected trigger bit is equal to '0', FIFO operates in Continuous mode.

5.5.5 Bypass-to-Continuous mode

In Bypass-to-Continuous mode (*FIFO_CTRL5 (0Ah)* (FIFO_MODE_[2:0] = '100'), data measurement storage inside FIFO operates in Continuous mode when selected triggers in one of the following interrupt registers *FUNC_SRC1 (53h)*, *TAP_SRC (1Ch)*, *WAKE_UP_SRC (1Bh)*and *D6D_SRC (1Dh)* are equal to '1', otherwise FIFO content is reset (Bypass mode).

5.5.6 FIFO reading procedure

The data stored in FIFO are accessible from dedicated registers (*FIFO_DATA_OUT_L (3Eh)* and *FIFO_DATA_OUT_H (3Fh)*) and each FIFO sample is composed of 16 bits.

All FIFO status registers (*FIFO_STATUS1 (3Ah)*, *FIFO_STATUS2 (3Bh)*, *FIFO_STATUS3 (3Ch)*, *FIFO_STATUS4 (3Dh)*) can be read at the start of a reading operation, minimizing the intervention of the application processor.

Saving data in the FIFO buffer is organized in four FIFO data sets consisting of 6 bytes each:

The 1st FIFO data set is reserved for gyroscope data;

The 2nd FIFO data set is reserved for accelerometer data;

The 3rd FIFO data set is reserved for the external sensor data stored in the registers from *SENSORHUB1_REG (2Eh)* to *SENSORHUB6_REG (33h)*;

The 4th FIFO data set can be alternately associated to the external sensor data stored in the registers from *SENSORHUB7_REG (34h)* to *SENSORHUB12_REG (39h)*, to the step counter and timestamp info, or to the temperature sensor data.

6 Digital interfaces

6.1 I^2C/SPI interface

The registers embedded inside the LSM6DSL may be accessed through both the I^2C and SPI serial interfaces. The latter may be SW configured to operate either in 3-wire or 4-wire interface mode. The device is compatible with SPI modes 0 and 3.

The serial interfaces are mapped onto the same pins. To select/exploit the I^2C interface, the CS line must be tied high (i.e connected to Vdd_IO).

Table 10. Serial interface pin description

Pin name	Pin description
CS	SPI enable<br>I^2C/SPI mode selection (1: SPI idle mode / I^2C communication enabled;<br>0: SPI communication mode / I^2C disabled)
SCL/SPC	I^2C Serial Clock (SCL)<br>SPI Serial Port Clock (SPC)
SDA/SDI/SDO	I^2C Serial Data (SDA)<br>SPI Serial Data Input (SDI)<br>3-wire Interface Serial Data Output (SDO)
SDO/SA0	SPI Serial Data Output (SDO)<br>I^2C less significant bit of the device address

6.2 Master I^2C

If the LSM6DSL is configured in Mode2, a master I^2C line is available. The master serial interface is mapped in the following dedicated pins.

Table 11. Master I^2C pin details

Pin name	Pin description
MSCL	I^2C serial clock master
MSDA	I^2C serial data master
MDRDY	I^2C master external synchronization signal

6.3 I²C serial interface

The LSM6DSL I²C is a bus slave. The I²C is employed to write the data to the registers, whose content can also be read back.

The relevant I²C terminology is provided in the table below.

Table 12. I²C terminology

Term	Description
Transmitter	The device which sends data to the bus
Receiver	The device which receives data from the bus
Master	The device which initiates a transfer, generates clock signals and terminates a transfer
Slave	The device addressed by the master

There are two signals associated with the I²C bus: the serial clock line (SCL) and the Serial DAta line (SDA). The latter is a bidirectional line used for sending and receiving the data to/from the interface. Both the lines must be connected to Vdd_IO through external pull-up resistors. When the bus is free, both the lines are high.

The I²C interface is implemeted with fast mode (400 kHz) I²C standards as well as with the standard mode.

In order to disable the I²C block, (I2C_disable) = 1 must be written in *CTRL4_C (13h)*.

6.3.1 I²C operation

The transaction on the bus is started through a START (ST) signal. A START condition is defined as a HIGH to LOW transition on the data line while the SCL line is held HIGH. After this has been transmitted by the master, the bus is considered busy. The next byte of data transmitted after the start condition contains the address of the slave in the first 7 bits and the eighth bit tells whether the master is receiving data from the slave or transmitting data to the slave. When an address is sent, each device in the system compares the first seven bits after a start condition with its address. If they match, the device considers itself addressed by the master.

The Slave ADdress (SAD) associated to the LSM6DSL is 110101xb. The SDO/SA0 pin can be used to modify the less significant bit of the device address. If the SDO/SA0 pin is connected to the supply voltage, LSb is ‘1’ (address 1101011b); else if the SDO/SA0 pin is connected to ground, the LSb value is ‘0’ (address 1101010b). This solution permits to connect and address two different inertial modules to the same I²C bus.

Data transfer with acknowledge is mandatory. The transmitter must release the SDA line during the acknowledge pulse. The receiver must then pull the data line LOW so that it remains stable low during the HIGH period of the acknowledge clock pulse. A receiver which has been addressed is obliged to generate an acknowledge after each byte of data received.

The I²C embedded inside the LSM6DSL behaves like a slave device and the following protocol must be adhered to. After the start condition (ST) a slave address is sent, once a slave acknowledge (SAK) has been returned, an 8-bit sub-address (SUB) is transmitted. The increment of the address is configured by the *CTRL3_C (12h)* (IF_INC).

The slave address is completed with a Read/Write bit. If the bit is ‘1’ (Read), a repeated START (SR) condition must be issued after the two sub-address bytes; if the bit is ‘0’ (Write) the master will transmit to the slave with direction unchanged. *Table 12* explains how the SAD+Read/Write bit pattern is composed, listing all the possible configurations.

Table 13. SAD+Read/Write patterns

Command	SAD[6:1]	SAD[0] = SA0	R/W	SAD+R/W
Read	110101	0	1	11010101 (D5h)
Write	110101	0	0	11010100 (D4h)
Read	110101	1	1	11010111 (D7h)
Write	110101	1	0	11010110 (D6h)

Table 14. Transfer when master is writing one byte to slave

Master	ST	SAD + W		SUB		DATA		SP
Slave			SAK		SAK		SAK	

Table 15. Transfer when master is writing multiple bytes to slave

Master	ST	SAD + W		SUB		DATA		DATA		SP
Slave			SAK		SAK		SAK		SAK	

Table 16. Transfer when master is receiving (reading) one byte of data from slave

Master	ST	SAD + W		SUB		SR	SAD + R			NMAK	SP
Slave			SAK		SAK			SAK	DATA		

Table 17. Transfer when master is receiving (reading) multiple bytes of data from slave

Master	ST	SAD+W		SUB		SR	SAD+R			MAK		MAK		NMAK	SP
Slave			SAK		SAK			SAK	DATA		DAT A		DATA		

Data are transmitted in byte format (DATA). Each data transfer contains 8 bits. The number of bytes transferred per transfer is unlimited. Data is transferred with the Most Significant bit (MSb) first. If a receiver can’t receive another complete byte of data until it has performed some other function, it can hold the clock line, SCL LOW to force the transmitter into a wait state. Data transfer only continues when the receiver is ready for another byte and releases the data line. If a slave receiver doesn’t acknowledge the slave address (i.e. it is not able to receive because it is performing some real-time function) the data line must be left HIGH by the slave. The master can then abort the transfer. A LOW to HIGH transition on the SDA line while the SCL line is HIGH is defined as a STOP condition. Each data transfer must be terminated by the generation of a STOP (SP) condition.

In the presented communication format MAK is Master acknowledge and NMAK is No Master Acknowledge.

6.4 SPI bus interface

The LSM6DSL SPI is a bus slave. The SPI allows writing and reading the registers of the device.

The serial interface communicates to the application using 4 wires: **CS**, **SPC**, **SDI** and **SDO**.

Figure 9. Read and write protocol (in mode 3)

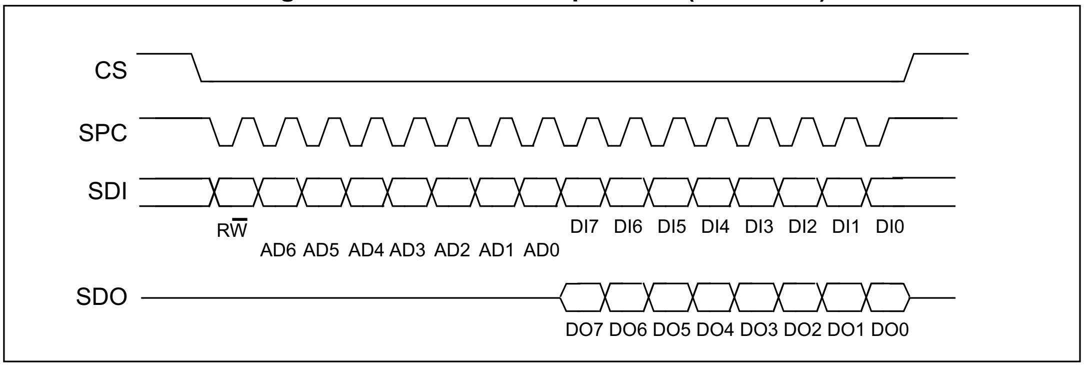

CS is the serial port enable and it is controlled by the SPI master. It goes low at the start of the transmission and goes back high at the end. **SPC** is the serial port clock and it is controlled by the SPI master. It is stopped high when **CS** is high (no transmission). **SDI** and **SDO** are, respectively, the serial port data input and output. Those lines are driven at the falling edge of **SPC** and should be captured at the rising edge of **SPC**.

Both the read register and write register commands are completed in 16 clock pulses or in multiples of 8 in case of multiple read/write bytes. Bit duration is the time between two falling edges of **SPC**. The first bit (bit 0) starts at the first falling edge of **SPC** after the falling edge of **CS** while the last bit (bit 15, bit 23, ...) starts at the last falling edge of SPC just before the rising edge of **CS**.

bit 0: $R\overline{W}$ bit. When 0, the data DI(7:0) is written into the device. When 1, the data DO(7:0) from the device is read. In latter case, the chip will drive **SDO** at the start of bit 8.

bit 1-7: address AD(6:0). This is the address field of the indexed register.

bit 8-15: data DI(7:0) (write mode). This is the data that is written into the device (MSb first).

bit 8-15: data DO(7:0) (read mode). This is the data that is read from the device (MSb first).

In multiple read/write commands further blocks of 8 clock periods will be added. When the *CTRL3_C (12h)* (IF_INC) bit is ‘0’, the address used to read/write data remains the same for every block. When the *CTRL3_C (12h)* (IF_INC) bit is ‘1’, the address used to read/write data is increased at every block.

The function and the behavior of **SDI** and **SDO** remain unchanged.

6.4.1 SPI read

Figure 10. SPI read protocol (in mode 3)

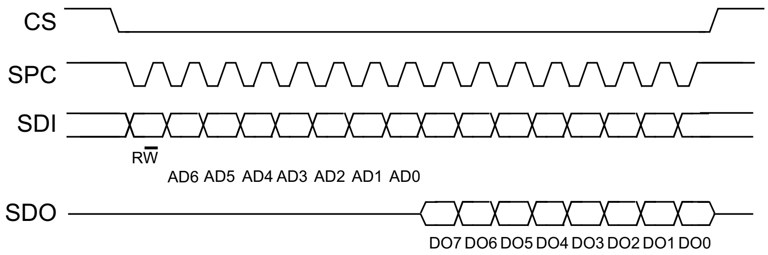

The SPI Read command is performed with 16 clock pulses. A multiple byte read command is performed by adding blocks of 8 clock pulses to the previous one.

bit 0: READ bit. The value is 1.

bit 1-7: address AD(6:0). This is the address field of the indexed register.

bit 8-15: data DO(7:0) (read mode). This is the data that will be read from the device (MSb first).

bit 16-...: data DO(...-8). Further data in multiple byte reads.

Figure 11. Multiple byte SPI read protocol (2-byte example) (in mode 3)

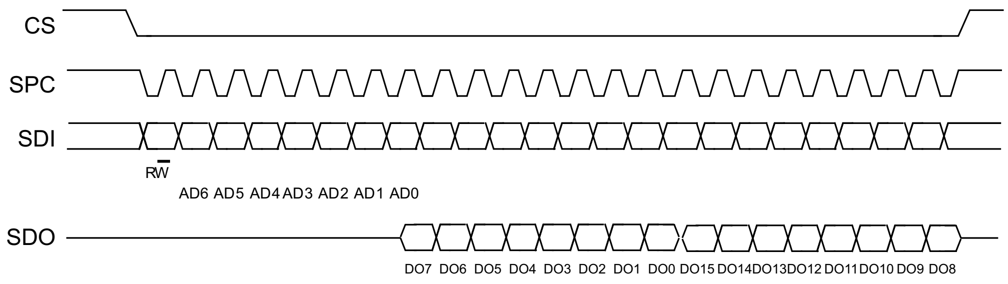

6.4.2 SPI write

Figure 12. SPI write protocol (in mode 3)

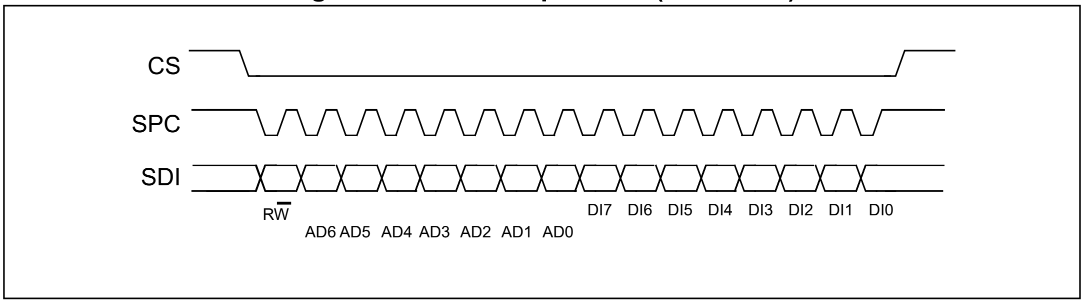

The SPI Write command is performed with 16 clock pulses. A multiple byte write command is performed by adding blocks of 8 clock pulses to the previous one.

bit 0: WRITE bit. The value is 0.

bit 1 -7: address AD(6:0). This is the address field of the indexed register.

bit 8-15: data DI(7:0) (write mode). This is the data that is written inside the device (MSb first).

bit 16-... : data DI(...-8). Further data in multiple byte writes.

Figure 13. Multiple byte SPI write protocol (2-byte example) (in mode 3)

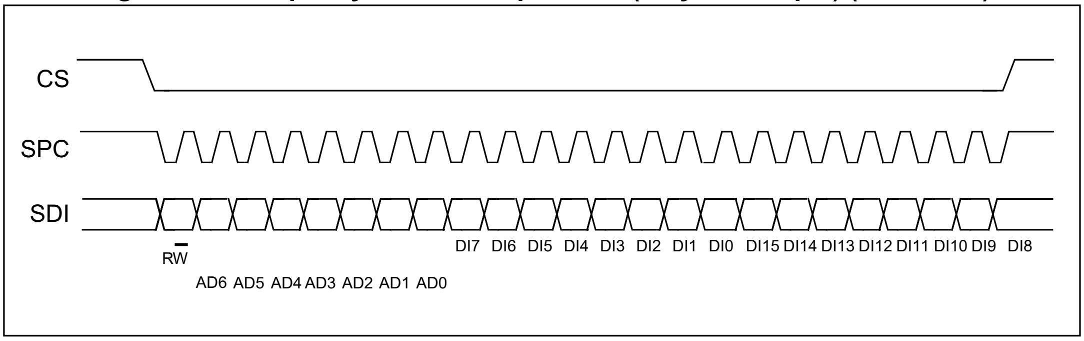

6.4.3 SPI read in 3-wire mode

A 3-wire mode is entered by setting the *CTRL3_C (12h)* (SIM) bit equal to ‘1’ (SPI serial interface mode selection).

Figure 14. SPI read protocol in 3-wire mode (in mode 3)

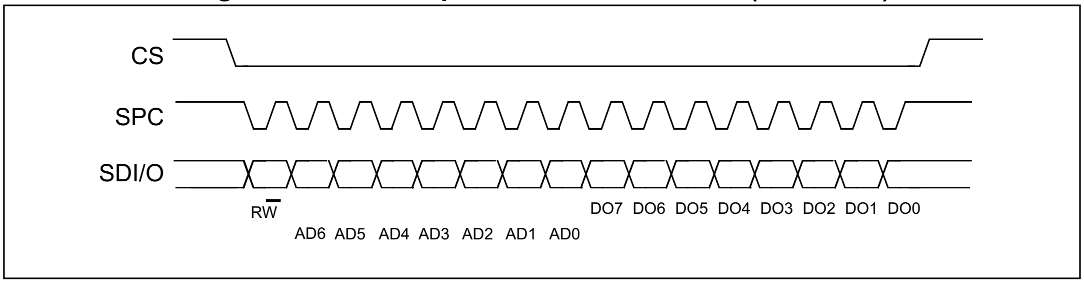

The SPI read command is performed with 16 clock pulses:

bit 0: READ bit. The value is 1.

bit 1-7: address AD(6:0). This is the address field of the indexed register.

bit 8-15: data DO(7:0) (read mode). This is the data that is read from the device (MSb first).

A multiple read command is also available in 3-wire mode.

7 Application hints

7.1 LSM6DSL electrical connections in Mode 1

Figure 15. LSM6DSL electrical connections in Mode 1

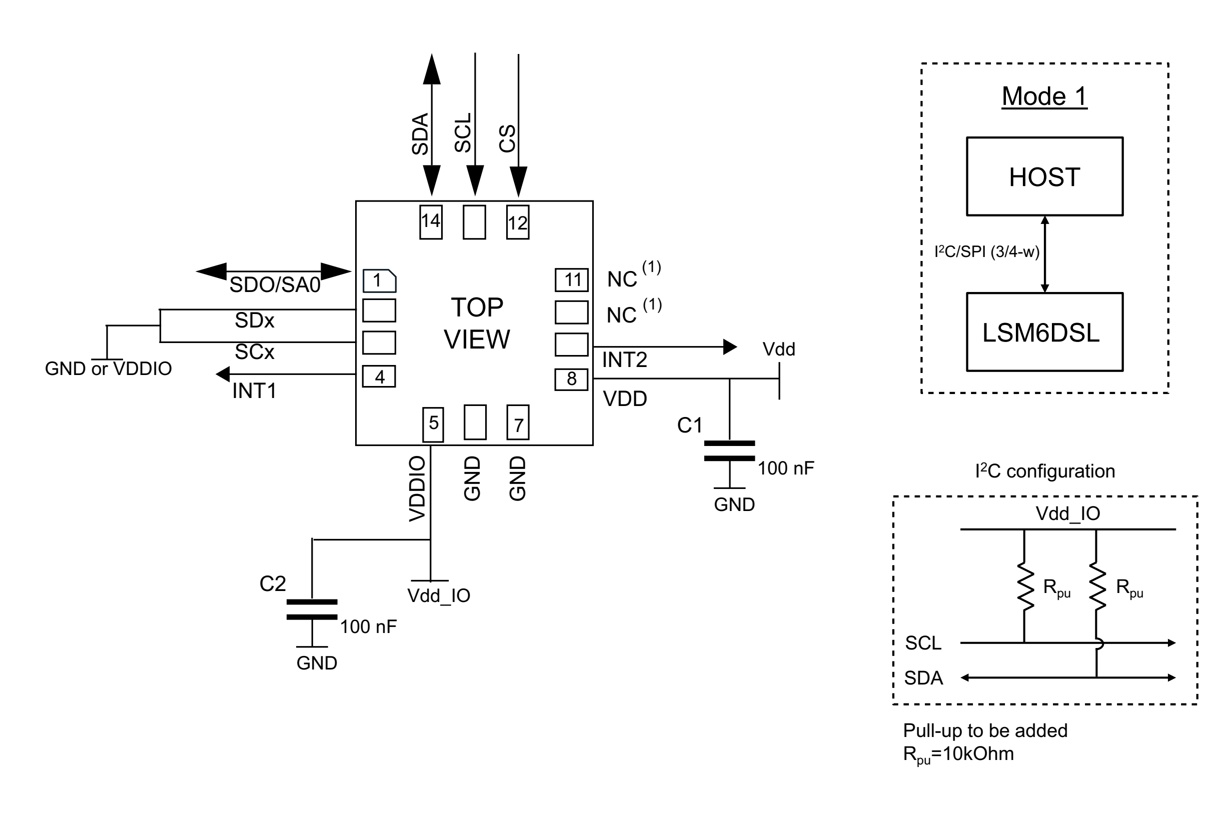

1. Leave pin electrically unconnected and soldered to PCB.

The device core is supplied through the Vdd line. Power supply decoupling capacitors (C1, C2 = 100 *nF* ceramic) should be placed as near as possible to the supply pin of the device (common design practice).

The functionality of the device and the measured acceleration/angular rate data is selectable and accessible through the SPI/I^2C interface.

The functions, the threshold and the timing of the two interrupt pins for each sensor can be completely programmed by the user through the SPI/I^2C interface.

7.2 LSM6DSL electrical connections in Mode 2

Figure 16. LSM6DSL electrical connections in Mode 2

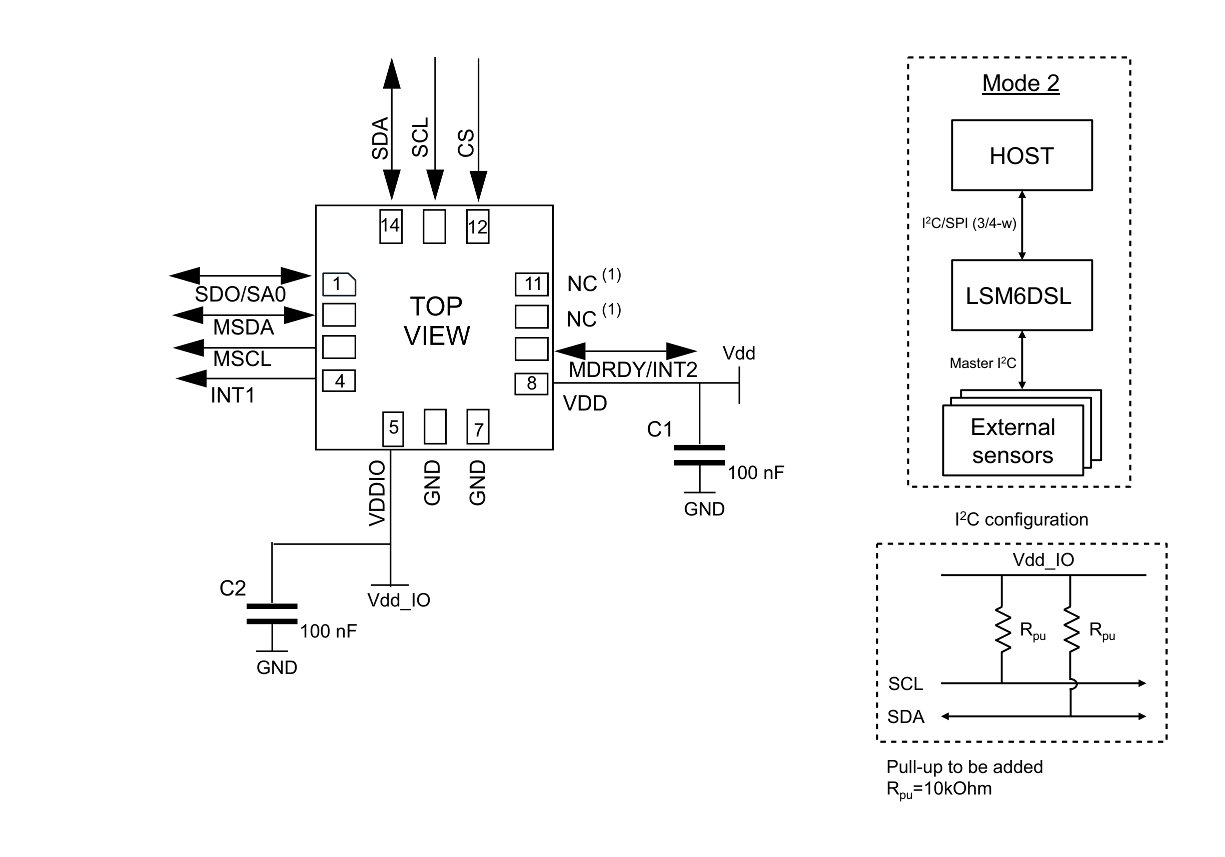

1. Leave pin electrically unconnected and soldered to PCB.

The device core is supplied through the Vdd line. Power supply decoupling capacitors (C1, C2 = 100 *nF* ceramic) should be placed as near as possible to the supply pin of the device (common design practice).

The functionality of the device and the measured acceleration/angular rate data is selectable and accessible through the SPI/I^{2}C interface.

The functions, the threshold and the timing of the two interrupt pins for each sensor can be completely programmed by the user through the SPI/I^{2}C interface.

Table 18. Internal pin status

pin#	Name	Mode 1 function	Mode 2 function	Pin status Mode 1	Pin status Mode 2
1	SDO	SPI 4-wire interface serial data output (SDO)	SPI 4-wire interface serial data output (SDO)	Default: Input without pull-up. Pull-up is enabled if bit SIM = 1 (SPI 3-wire) in reg 12h.	Default: Input without pull-up. Pull-up is enabled if bit SIM = 1 (SPI 3-wire) in reg 12h.
	SA0	I^2C least significant bit of the device address (SA0)	I^2C least significant bit of the device address (SA0)		
2	SDx	Connect to VDDIO or GND	I^2C serial data master (MSDA)	Default: input without pull-up. Pull-up is enabled if bit PULL_UP_EN = 1 in reg 1Ah.	Default: input without pull-up. Pull-up is enabled if bit PULL_UP_EN = 1 in reg 1Ah.
3	SCx	Connect to VDDIO or GND	I^2C serial clock master (MSCL)	Default: input without pull-up. Pull-up is enabled if bit PULL_UP_EN = 1 in reg 1Ah.	Default: input without pull-up. Pull-up is enabled if bit PULL_UP_EN = 1 in reg 1Ah.
4	INT1	Programmable interrupt 1	Programmable interrupt 1	Default: Output forced to ground	Default: Output forced to ground
5	Vdd_IO	Power supply for I/O pins	Power supply for I/O pins		
6	GND	0 V supply	0 V supply		
7	GND	0 V supply	0 V supply		
8	Vdd	Power supply	Power supply		
9	INT2	Programmable interrupt 2 (INT2) / Data enabled (DEN)	Programmable interrupt 2 (INT2) / Data enabled (DEN) / I^2C master external synchronization signal (MDRDY)	Default: Output forced to ground	Default: Output forced to ground
10	NC	Leave unconnected[1]	Leave unconnected[1]	Default: Input with pull-up. (See note below to disable pull-up)	Default: Input with pull-up. (See note below to disable pull-up)
11	NC	Leave unconnected[1]	Leave unconnected[1]	Default: Input with pull-up. (See note below to disable pull-up)	Default: Input with pull-up. (See note below to disable pull-up)
12	CS	I^2C/SPI mode selection (1:SPI idle mode / I^2C communication enabled; 0: SPI communication mode / I^2C disabled)	I^2C/SPI mode selection (1:SPI idle mode / I^2C communication enabled; 0: SPI communication mode / I^2C disabled)	Default: Input with pull-up. Pull-up is disabled if bit I2C_disable = 1 in reg 13h.	Default: Input with pull-up. Pull-up is disabled if bit I2C_disable = 1 in reg 13h.

Table 18. Internal pin status (continued)

pin#	Name	Mode 1 function	Mode 2 function	Pin status Mode 1	Pin status Mode 2
13	SCL	I^2C serial clock (SCL) / SPI serial port clock (SPC)	I^2C serial clock (SCL) / SPI serial port clock (SPC)	Input without pull-up	Input without pull-up
14	SDA	I^2C serial data (SDA) / SPI serial data input (SDI) / 3-wire interface serial data output (SDO)	I^2C serial data (SDA) / SPI serial data input (SDI) / 3-wire interface serial data output (SDO)	Input without pull-up	Input without pull-up

1. Leave pin electrically unconnected and soldered to PCB.

Internal pull-up value is from 30 kΩ to 50 kΩ, depending on VDDIO.

Note: The procedure to disable the pull-up on pins 10-11 is as follows:

1. *AP side: write 80h in register at address 00h*
2. *AP side: write 01h in register at address 05h (disable the pull-up on pins 10 and 11 of LSM6DSL)*
3. *AP side: write 00h in register at address 00h*

8 Register mapping

The table given below provides a list of the 8/16-bit registers embedded in the device and the corresponding addresses.

Table 19. Registers address map

Name	Type	Register address		Default	Comment
		Hex	Binary		
RESERVED	-	00	00000000	-	Reserved
FUNC_CFG_ACCESS	r/w	01	00000001	00000000	Embedded functions configuration register
RESERVED	-	02	00000010	-	Reserved
RESERVED	-	03	00000011	-	Reserved
SENSOR_SYNC_TIME_FRAME	r/w	04	00000100	00000000	Sensor sync configuration register
SENSOR_SYNC_RES_RATIO	r/w	05	00000101	00000000	
FIFO_CTRL1	r/w	06	00000110	00000000	FIFO configuration registers
FIFO_CTRL2	r/w	07	00000111	00000000	
FIFO_CTRL3	r/w	08	00001000	00000000	
FIFO_CTRL4	r/w	09	00001001	00000000	
FIFO_CTRL5	r/w	0A	00001010	00000000	
DRDY_PULSE_CFG_G	r/w	0B	00001011	00000000	
RESERVED	-	0C	00001100	-	Reserved
INT1_CTRL	r/w	0D	00001101	00000000	INT1 pin control
INT2_CTRL	r/w	0E	00001110	00000000	INT2 pin control
WHO_AM_I	r	0F	00001111	01101010	Who I am ID
CTRL1_XL	r/w	10	00010000	00000000	Accelerometer and gyroscope control registers
CTRL2_G	r/w	11	00010001	00000000	
CTRL3_C	r/w	12	00010010	00000100	
CTRL4_C	r/w	13	00010011	00000000	
CTRL5_C	r/w	14	00010100	00000000	
CTRL6_C	r/w	15	00010101	00000000	
CTRL7_G	r/w	16	00010110	00000000	
CTRL8_XL	r/w	17	0001 0111	00000000	
CTRL9_XL	r/w	18	00011000	00000000	
CTRL10_C	r/w	19	00011001	00000000	

Table 19. Registers address map (continued)

Name	Type	Register address		Default	Comment
		Hex	Binary		
MASTER_CONFIG	r/w	1A	00011010	00000000	I^2C master configuration register
WAKE_UP_SRC	r	1B	00011011	output	Interrupt registers
TAP_SRC	r	1C	00011100	output	
D6D_SRC	r	1D	00011101	output	
STATUS_REG	r	1E	00011110	output	Status data register for user interface
RESERVED	-	1F	00011111	-	
OUT_TEMP_L	r	20	00100000	output	Temperature output data registers
OUT_TEMP_H	r	21	00100001	output	
OUTX_L_G	r	22	00100010	output	Gyroscope output registers for user interface
OUTX_H_G	r	23	00100011	output	
OUTY_L_G	r	24	00100100	output	
OUTY_H_G	r	25	00100101	output	
OUTZ_L_G	r	26	00100110	output	
OUTZ_H_G	r	27	00100111	output	
OUTX_L_XL	r	28	00101000	output	Accelerometer output registers
OUTX_H_XL	r	29	00101001	output	
OUTY_L_XL	r	2A	00101010	output	
OUTY_H_XL	r	2B	00101011	output	
OUTZ_L_XL	r	2C	00101100	output	
OUTZ_H_XL	r	2D	00101101	output	
SENSORHUB1_REG	r	2E	00101110	output	Sensor hub output registers
SENSORHUB2_REG	r	2F	00101111	output	
SENSORHUB3_REG	r	30	00110000	output	
SENSORHUB4_REG	r	31	00110001	output	
SENSORHUB5_REG	r	32	00110010	output	
SENSORHUB6_REG	r	33	00110011	output	
SENSORHUB7_REG	r	34	00110100	output	
SENSORHUB8_REG	r	35	00110101	output	
SENSORHUB9_REG	r	36	00110110	output	
SENSORHUB10_REG	r	37	00110111	output	
SENSORHUB11_REG	r	38	00111000	output	
SENSORHUB12_REG	r	39	00111001	output	

Table 19. Registers address map (continued)

Name	Type	Register address		Default	Comment
		Hex	Binary		
FIFO_STATUS1	r	3A	00111010	output	FIFO status registers
FIFO_STATUS2	r	3B	00111011	output	
FIFO_STATUS3	r	3C	00111100	output	
FIFO_STATUS4	r	3D	00111101	output	
FIFO_DATA_OUT_L	r	3E	00111110	output	FIFO data output registers
FIFO_DATA_OUT_H	r	3F	00111111	output	
TIMESTAMP0_REG	r	40	01000000	output	Timestamp output registers
TIMESTAMP1_REG	r	41	01000001	output	
TIMESTAMP2_REG	r/w	42	01000010	output	
RESERVED	-	43-48		-	Reserved
STEP_TIMESTAMP_L	r	49	0100 1001	output	Step counter timestamp registers
STEP_TIMESTAMP_H	r	4A	0100 1010	output	
STEP_COUNTER_L	r	4B	01001011	output	Step counter output registers
STEP_COUNTER_H	r	4C	01001100	output	
SENSORHUB13_REG	r	4D	01001101	output	Sensor hub output registers
SENSORHUB14_REG	r	4E	01001110	output	
SENSORHUB15_REG	r	4F	01001111	output	
SENSORHUB16_REG	r	50	01010000	output	
SENSORHUB17_REG	r	51	01010001	output	
SENSORHUB18_REG	r	52	01010010	output	
FUNC_SRC1	r	53	01010011	output	Interrupt registers
FUNC_SRC2	r	54	01010100	output	
WRIST_TILT_IA	r	55	01010101	output	Interrupt register
RESERVED	-	56-57		-	Reserved
TAP_CFG	r/w	58	01011000	00000000	Interrupt registers
TAP_THS_6D	r/w	59	01011001	00000000	
INT_DUR2	r/w	5A	01011010	00000000	
WAKE_UP_THS	r/w	5B	01011011	00000000	
WAKE_UP_DUR	r/w	5C	01011100	00000000	
FREE_FALL	r/w	5D	01011101	00000000	
MD1_CFG	r/w	5E	01011110	00000000	
MD2_CFG	r/w	5F	01011111	00000000	
MASTER_CMD_CODE	r/w	60	01100000	00000000	

Table 19. Registers address map (continued)

Name	Type	Register address		Default	Comment
		Hex	Binary		
SENS_SYNC_SPI_ ERROR_CODE	r/w	61	0110 0001	00000000	
RESERVED	-	62-65		-	Reserved
OUT_MAG_RAW_X_L	r	66	01100110	output	External magnetometer raw data output registers
OUT_MAG_RAW_X_H	r	67	01100111	output	
OUT_MAG_RAW_Y_L	r	68	01101000	output	
OUT_MAG_RAW_Y_H	r	69	01101001	output	
OUT_MAG_RAW_Z_L	r	6A	01101010	output	
OUT_MAG_RAW_Z_H	r	6B	01101011	output	
RESERVED	-	6C-72		-	Reserved
X_OFS_USR	r/w	73	01110011	00000000	Accelerometer user offset correction
Y_OFS_USR	r/w	74	01110100	00000000	
Z_OFS_USR	r/w	75	01110101	00000000	
RESERVED	-	76-7F		-	Reserved

9 Register description

The device contains a set of registers which are used to control its behavior and to retrieve linear acceleration, angular rate and temperature data. The register addresses, made up of 7 bits, are used to identify them and to write the data through the serial interface.

9.1 FUNC_CFG_ACCESS (01h)

Enable embedded functions register (r/w).

Table 20. FUNC_CFG_ACCESS register

FUNC_ CFG_EN	0(1)	FUNC_ CFG_EN_B	0(1)	0(1)	0(1)	0(1)	0(1)

1. This bit must be set to '0' for the correct operation of the device.

Table 21. FUNC_CFG_ACCESS register description

FUNC_CFG_ EN	Enable access to the embedded functions configuration registers bank A and B(1). Default value: 0. Refer to *Table 22*.
FUNC_CFG_ EN_B	Enable access to the embedded functions configuration register bank B (1). Default value: 0. Refer to *Table 22*.

1. The embedded functions configuration registers details are available in *Section 10: Embedded functions register mapping*, *Section 11: Embedded functions registers description - Bank A*, and *Section 12: Embedded functions registers description - Bank B*.

Table 22. Configuration of embedded functions register banks

FUNC_CFG_EN	FUNC_CFG_EN_B	**Status of embedded register banks**
0	0	Bank A and B disabled (default)
0	1	Forbidden
1	0	Bank A enabled
1	1	Bank B enabled

9.2 SENSOR_SYNC_TIME_FRAME (04h)

Sensor synchronization time frame register (r/w).

Table 23. SENSOR_SYNC_TIME_FRAME register

0(1)	0(1)	0(1)	0(1)	TPH_3	TPH_2	TPH_1	TPH_0

1. This bit must be set to '0' for the correct operation of the device.

Table 24. SENSOR_SYNC_TIME_FRAME register description

TPH_ [3:0]	Sensor synchronization time frame with the step of 500 ms and full range of 5 s. Unsigned 8-bit.<br>Default value: 0000 0000 (sensor sync disabled)

9.3 SENSOR_SYNC_RES_RATIO (05h)

Sensor synchronization resolution ratio (r/w)

Table 25. SENSOR_SYNC_RES_RATIO register

0[1]	0[1]	0[1]	0[1]	0[1]	0[1]	RR_1	RR_0

1. This bit must be set to '0' for the correct operation of the device.

Table 26. SENSOR_SYNC_RES_RATIO register description

RR_[1:0]	Resolution ratio of error code for sensor synchronization:<br>00: SensorSync, Res_Ratio = 2-11<br>01: SensorSync, Res_Ratio = 2-12<br>10: SensorSync, Res_Ratio = 2-13<br>11: SensorSync, Res_Ratio = 2-14

9.4 FIFO_CTRL1 (06h)

FIFO control register (r/w).

Table 27. FIFO_CTRL1 register

FTH_7	FTH_6	FTH_5	FTH_4	FTH_3	FTH_2	FTH_1	FTH_0

Table 28. FIFO_CTRL1 register description

FTH_[7:0]	FIFO threshold level setting[1]. Default value: 0000 0000.<br>Watermark flag rises when the number of bytes written to FIFO after the next write is greater than or equal to the threshold level.<br>Minimum resolution for the FIFO is 1 LSB = 2 bytes (1 word) in FIFO

1. For a complete watermark threshold configuration, consider FTH_[10:8] in *FIFO_CTRL2 (07h)*.

9.5 FIFO_CTRL2 (07h)

FIFO control register (r/w).

Table 29. FIFO_CTRL2 register

TIMER_PEDO _FIFO_EN	TIMER_PEDO _FIFO_DRDY	0(1)	0(1)	FIFO_ TEMP_EN	FTH10	FTH_9	FTH_8

1. This bit must be set to ‘0’ for the correct operation of the device.

Table 30. FIFO_CTRL2 register description

TIMER_PEDO _FIFO_EN	Enable pedometer step counter and timestamp as 4th FIFO data set. Default: 0<br>(0: disable step counter and timestamp data as 4th FIFO data set;<br>1: enable step counter and timestamp data as 4th FIFO data set)
TIMER_PEDO _FIFO_DRDY	FIFO write mode(1). Default: 0<br>(0: enable write in FIFO based on XL/Gyro data-ready;<br>1: enable write in FIFO at every step detected by step counter.)
FIFO_TEMP_EN	Enable the temperature data storage in FIFO. Default: 0.<br>(0: temperature not included in FIFO; 1: temperature included in FIFO)
FTH_[10:8]	FIFO threshold level setting(2). Default value: 0000<br>Watermark flag rises when the number of bytes written to FIFO after the next write is greater than or equal to the threshold level.<br>Minimum resolution for the FIFO is 1LSB = 2 bytes (1 word) in FIFO

1. This bit is effective if the DATA_VALID_SEL_FIFO bit of the *MASTER_CONFIG (1Ah)* register is set to 0.
2. For a complete watermark threshold configuration, consider FTH_[7:0] in *FIFO_CTRL1 (06h)*.

9.6 FIFO_CTRL3 (08h)

FIFO control register (r/w).

Table 31. FIFO_CTRL3 register

0(1)	0(1)	DEC_FIFO _GYRO2	DEC_FIFO _GYRO1	DEC_FIFO _GYRO0	DEC_FIFO _XL2	DEC_FIFO _XL1	DEC_FIFO _XL0

1. This bit must be set to ‘0’ for the correct operation of the device.

Table 32. FIFO_CTRL3 register description

DEC_FIFO_GYRO [2:0]	Gyro FIFO (first data set) decimation setting. Default: 000<br>For the configuration setting, refer to *Table 33*.
DEC_FIFO_XL [2:0]	Accelerometer FIFO (second data set) decimation setting. Default: 000<br>For the configuration setting, refer to *Table 34*.

Table 33. Gyro FIFO decimation setting

DEC_FIFO_GYRO [2:0]	Configuration
000	Gyro sensor not in FIFO
001	No decimation
010	Decimation with factor 2
011	Decimation with factor 3
100	Decimation with factor 4
101	Decimation with factor 8
110	Decimation with factor 16
111	Decimation with factor 32

Table 34. Accelerometer FIFO decimation setting

DEC_FIFO_XL [2:0]	Configuration
000	Accelerometer sensor not in FIFO
001	No decimation
010	Decimation with factor 2
011	Decimation with factor 3
100	Decimation with factor 4
101	Decimation with factor 8
110	Decimation with factor 16
111	Decimation with factor 32

9.7 FIFO_CTRL4 (09h)

FIFO control register (r/w).

Table 35. FIFO_CTRL4 register

STOP_ ON_ FTH	ONLY_HIGH _DATA	DEC_DS4 _FIFO2	DEC_DS4 _FIFO1	DEC_DS4 _FIFO0	DEC_DS3 _FIFO2	DEC_DS3 _FIFO1	DEC_DS3 _FIFO0

Table 36. FIFO_CTRL4 register description

STOP_ON_FTH	Enable FIFO threshold level use. Default value: 0.<br>(0: FIFO depth is not limited; 1: FIFO depth is limited to threshold level)
ONLY_HIGH_DATA	8-bit data storage in FIFO. Default: 0<br>(0: disable MSByte only memorization in FIFO for XL and Gyro;<br>1: enable MSByte only memorization in FIFO for XL and Gyro in FIFO)
DEC_DS4_FIFO[2:0]	Fourth FIFO data set decimation setting. Default: 000<br>For the configuration setting, refer to *Table 37*.
DEC_DS3_FIFO[2:0]	Third FIFO data set decimation setting. Default: 000<br>For the configuration setting, refer to *Table 38*.

Table 37. Fourth FIFO data set decimation setting

DEC_DS4_FIFO[2:0]	Configuration
000	Fourth FIFO data set not in FIFO
001	No decimation
010	Decimation with factor 2
011	Decimation with factor 3
100	Decimation with factor 4
101	Decimation with factor 8
110	Decimation with factor 16
111	Decimation with factor 32

Table 38. Third FIFO data set decimation setting

DEC_DS3_FIFO[2:0]	Configuration
000	Third FIFO data set not in FIFO
001	No decimation
010	Decimation with factor 2
011	Decimation with factor 3
100	Decimation with factor 4
101	Decimation with factor 8
110	Decimation with factor 16
111	Decimation with factor 32

9.8 FIFO_CTRL5 (0Ah)

FIFO control register (r/w).

Table 39. FIFO_CTRL5 register

0(1)	ODR_ FIFO_3	ODR_ FIFO_2	ODR_ FIFO_1	ODR_ FIFO_0	FIFO_ MODE_2	FIFO_ MODE_1	FIFO_ MODE_0

1. This bit must be set to ‘0’ for the correct operation of the device.

Table 40. FIFO_CTRL5 register description

ODR_FIFO_[3:0]	FIFO ODR selection, setting FIFO_MODE also. Default: 0000<br>For the configuration setting, refer to *Table 41*.
FIFO_MODE_[2:0]	FIFO mode selection bits, setting ODR_FIFO also. Default value: 000<br>For the configuration setting, refer to *Table 42*.

Table 41. FIFO ODR selection

ODR_FIFO_[3:0]	Configuration(1)
0000	FIFO disabled
0001	FIFO ODR is set to 12.5 Hz
0010	FIFO ODR is set to 26 Hz
0011	FIFO ODR is set to 52 Hz
0100	FIFO ODR is set to 104 Hz
0101	FIFO ODR is set to 208 Hz
0110	FIFO ODR is set to 416 Hz
0111	FIFO ODR is set to 833 Hz
1000	FIFO ODR is set to 1.66 kHz
1001	FIFO ODR is set to 3.33 kHz
1010	FIFO ODR is set to 6.66 kHz

1. If the device is working at an ODR slower than the one selected, FIFO ODR is limited to that ODR value. Moreover, these bits are effective if both the DATA_VALID_SEL_FIFO bit of *MASTER_CONFIG (1Ah)* and the TIMER_PEDO_FIFO_DRDY bit of *FIFO_CTRL2 (07h)* are set to 0.

Table 42. FIFO mode selection

FIFO_MODE_[2:0]	Configuration mode
000	Bypass mode. FIFO disabled.
001	FIFO mode. Stops collecting data when FIFO is full.
010	Reserved
011	Continuous mode until trigger is deasserted, then FIFO mode.
100	Bypass mode until trigger is deasserted, then Continuous mode.
101	Reserved
110	Continuous mode. If the FIFO is full, the new sample overwrites the older one.
111	Reserved

9.9 DRDY_PULSE_CFG_G (0Bh)

DataReady configuration register (r/w).

Table 43. DRDY_PULSE_CFG_G register

DRDY_ PULSED	0(1)	0(1)	0(1)	0(1)	0(1)	0(1)	INT2_ WRIST_TILT

1. This bit must be set to '0' for the correct operation of the device.

Table 44. DRDY_PULSE_CFG_G register description

DRDY_PULSED	Enable pulsed DataReady mode. Default value: 0<br>(0: DataReady latched mode. Returns to 0 only after output data have been read;<br>1: DataReady pulsed mode. The DataReady pulses are 75 μs long.)
INT2_ WRIST_TILT	Wrist tilt interrupt on INT2 pad. Default value: 0<br>(0: disabled; 1: enabled)

9.10 INT1_CTRL (0Dh)

INT1 pad control register (r/w).

Each bit in this register enables a signal to be carried through INT1. The pad's output will supply the OR combination of the selected signals.

Table 45. INT1_CTRL register

INT1_STEP_ DETECTOR	INT1_SIGN _MOT	INT1_FULL _FLAG	INT1_ FIFO_OVR	INT1_ FTH	INT1_ BOOT	INT1_ DRDY_G	INT1_ DRDY_XL

Table 46. INT1_CTRL register description

INT1_ STEP_ DETECTOR	Pedometer step recognition interrupt enable on INT1 pad. Default value: 0<br>(0: disabled; 1: enabled)
INT1_SIGN_MOT	Significant motion interrupt enable on INT1 pad. Default value: 0<br>(0: disabled; 1: enabled)
INT1_FULL_FLAG	FIFO full flag interrupt enable on INT1 pad. Default value: 0<br>(0: disabled; 1: enabled)
INT1_FIFO_OVR	FIFO overrun interrupt on INT1 pad. Default value: 0<br>(0: disabled; 1: enabled)
INT1_FTH	FIFO threshold interrupt on INT1 pad. Default value: 0<br>(0: disabled; 1: enabled)
INT1_ BOOT	Boot status available on INT1 pad. Default value: 0<br>(0: disabled; 1: enabled)
INT1_DRDY_G	Gyroscope Data Ready on INT1 pad. Default value: 0<br>(0: disabled; 1: enabled)
INT1_DRDY_XL	Accelerometer Data Ready on INT1 pad. Default value: 0<br>(0: disabled; 1: enabled)

9.11 INT2_CTRL (0Eh)

INT2 pad control register (r/w).

Each bit in this register enables a signal to be carried through INT2. The pad's output will supply the OR combination of the selected signals.

Table 47. INT2_CTRL register

INT2_STEP_DELTA	INT2_STEP_COUNT_OV	INT2_FULL_FLAG	INT2_FIFO_OVR	INT2_FTH	INT2_DRDY_TEMP	INT2_DRDY_G	INT2_DRDY_XL

Table 48. INT2_CTRL register description

INT2_STEP_DELTA	Pedometer step recognition interrupt on delta time[(1)] enable on INT2 pad. Default value: 0<br>(0: disabled; 1: enabled)
INT2_STEP_COUNT_OV	Step counter overflow interrupt enable on INT2 pad. Default value: 0<br>(0: disabled; 1: enabled)
INT2_ FULL_FLAG	FIFO full flag interrupt enable on INT2 pad. Default value: 0<br>(0: disabled; 1: enabled)
INT2_FIFO_OVR	FIFO overrun interrupt on INT2 pad. Default value: 0<br>(0: disabled; 1: enabled)
INT2_FTH	FIFO threshold interrupt on INT2 pad. Default value: 0<br>(0: disabled; 1: enabled)
INT2_DRDY_TEMP	Temperature Data Ready on INT2 pad. Default value: 0<br>(0: disabled; 1: enabled)
INT2_DRDY_G	Gyroscope Data Ready on INT2 pad. Default value: 0<br>(0: disabled; 1: enabled)
INT2_DRDY_XL	Accelerometer Data Ready on INT2 pad. Default value: 0<br>(0: disabled; 1: enabled)

1. Delta time value is defined in register *STEP_COUNT_DELTA (15h)*.

9.12 WHO_AM_I (0Fh)

Who_AM_I register (r). This register is a read-only register. Its value is fixed at 6Ah.

Table 49. WHO_AM_I register

0	1	1	0	1	0	1	0

9.13 CTRL1_XL (10h)

Linear acceleration sensor control register 1 (r/w).

Table 50. CTRL1_XL register

ODR_XL3	ODR_XL2	ODR_XL1	ODR_XL0	FS_XL1	FS_XL0	LPF1_BW_ SEL	BW0_XL

Table 51. CTRL1_XL register description

ODR_XL [3:0]	Output data rate and power mode selection. Default value: 0000 (see *Table 52*).
FS_XL [1:0]	Accelerometer full-scale selection. Default value: 00.<br>(00: ±2 *g*; 01: ±16 *g*; 10: ±4 *g*; 11: ±8 *g*)
LPF1_BW_SEL	Accelerometer digital LPF (LPF1) bandwidth selection. For bandwidth selection refer to *CTRL8_XL (17h)*.
BW0_XL	Accelerometer analog chain bandwidth selection (only for accelerometer ODR ≥ 1.67 kHz).<br>(0: BW @ 1.5 kHz;<br>1: BW @ 400 Hz)

Table 52. Accelerometer ODR register setting

ODR_ XL3	ODR_ XL2	ODR_ XL1	ODR_ XL0	ODR selection [Hz] when XL_HM_MODE = 1	ODR selection [Hz] when XL_HM_MODE = 0
0	0	0	0	Power-down	Power-down
1	0	1	1	1.6 Hz (low power only)	12.5 Hz (high performance)
0	0	0	1	12.5 Hz (low power)	12.5 Hz (high performance)
0	0	1	0	26 Hz (low power)	26 Hz (high performance)
0	0	1	1	52 Hz (low power)	52 Hz (high performance)
0	1	0	0	104 Hz (normal mode)	104 Hz (high performance)
0	1	0	1	208 Hz (normal mode)	208 Hz (high performance)
0	1	1	0	416 Hz (high performance)	416 Hz (high performance)
0	1	1	1	833 Hz (high performance)	833 Hz (high performance)
1	0	0	0	1.66 kHz (high performance)	1.66 kHz (high performance)
1	0	0	1	3.33 kHz (high performance)	3.33 kHz (high performance)
1	0	1	0	6.66 kHz (high performance)	6.66 kHz (high performance)
1	1	x	x	Not allowed	Not allowed

9.14 CTRL2_G (11h)

Angular rate sensor control register 2 (r/w).

Table 53. CTRL2_G register

ODR_G3	ODR_G2	ODR_G1	ODR_G0	FS_G1	FS_G0	FS_125	0[(1)]

1. This bit must be set to ‘0’ for the correct operation of the device.

Table 54. CTRL2_G register description

ODR_G [3:0]	Gyroscope output data rate selection. Default value: 0000<br>(Refer to *Table 55*)
FS_G [1:0]	Gyroscope full-scale selection. Default value: 00<br>(00: 250 dps; 01: 500 dps; 10: 1000 dps; 11: 2000 dps)
FS_125	Gyroscope full-scale at 125 dps. Default value: 0<br>(0: disabled; 1: enabled)

Table 55. Gyroscope ODR configuration setting

ODR_G3	ODR_G2	ODR_G1	ODR_G0	ODR [Hz] when G_HM_MODE = 1	ODR [Hz] when G_HM_MODE = 0
0	0	0	0	Power down	Power down
0	0	0	1	12.5 Hz (low power)	12.5 Hz (high performance)
0	0	1	0	26 Hz (low power)	26 Hz (high performance)
0	0	1	1	52 Hz (low power)	52 Hz (high performance)
0	1	0	0	104 Hz (normal mode)	104 Hz (high performance)
0	1	0	1	208 Hz (normal mode)	208 Hz (high performance)
0	1	1	0	416 Hz (high performance)	416 Hz (high performance)
0	1	1	1	833 Hz (high performance)	833 Hz (high performance)
1	0	0	0	1.66 kHz (high performance)	1.66 kHz (high performance)
1	0	0	1	3.33 kHz (high performance	3.33 kHz (high performance)
1	0	1	0	6.66 kHz (high performance	6.66 kHz (high performance)
1	0	1	1	Not available	Not available

9.15 CTRL3_C (12h)

Control register 3 (r/w).

Table 56. CTRL3_C register

BOOT	BDU	H_LACTIVE	PP_OD	SIM	IF_INC	BLE	SW_RESET

Table 57. CTRL3_C register description

BOOT	Reboot memory content. Default value: 0<br>(0: normal mode; 1: reboot memory content)
BDU	Block Data Update. Default value: 0<br>(0: continuous update; 1: output registers not updated until MSB and LSB have been read)
H_LACTIVE	Interrupt activation level. Default value: 0<br>(0: interrupt output pads active high; 1: interrupt output pads active low)
PP_OD	Push-pull/open-drain selection on INT1 and INT2 pads. Default value: 0<br>(0: push-pull mode; 1: open-drain mode)
SIM	SPI Serial Interface Mode selection. Default value: 0<br>(0: 4-wire interface; 1: 3-wire interface).
IF_INC	Register address automatically incremented during a multiple byte access with a serial interface (I^2C or SPI). Default value: 1<br>(0: disabled; 1: enabled)
BLE	Big/Little Endian Data selection. Default value 0<br>(0: data LSB @ lower address; 1: data MSB @ lower address)
SW_RESET	Software reset. Default value: 0<br>(0: normal mode; 1: reset device)<br>This bit is automatically cleared.

9.16 CTRL4_C (13h)

Control register 4 (r/w).

Table 58. CTRL4_C register

DEN_ XL_EN	SLEEP	INT2_on_ INT1	DEN_DRDY _INT1	DRDY_ MASK	I2C_disable	LPF1_SEL_G	0(1)

1. This bit must be set to ‘0’ for the correct operation of the device.

Table 59. CTRL4_C register description

DEN_XL_EN	Extend DEN functionality to accelerometer sensor. Default value: 0<br>(0: disabled; 1: enabled)
SLEEP	Gyroscope sleep mode enable. Default value: 0<br>(0: disabled; 1: enabled)
DEN_DRDY _INT1	DEN DRDY signal on INT1 pad. Default value: 0<br>(0: disabled; 1: enabled)
INT2_on_INT1	All interrupt signals available on INT1 pad enable. Default value: 0<br>(0: interrupt signals divided between INT1 and INT2 pads;<br>1: all interrupt signals in logic or on INT1 pad)
DRDY_MASK	Configuration 1 data available enable bit. Default value: 0<br>(0: DA timer disabled; 1: DA timer enabled)
I2C_disable	Disable I^2C interface. Default value: 0<br>(0: both I^2C and SPI enabled; 1: I^2C disabled, SPI only enabled)
LPF1_SEL_G	Enable gyroscope digital LPF1. The bandwidth can be selected through FTYPE[1:0] in *CTRL6_C (15h)*.<br>(0: disabled; 1: enabled)

9.17 CTRL5_C (14h)

Control register 5 (r/w).

Table 60. CTRL5_C register

ROUNDING2	ROUNDING1	ROUNDING0	DEN _LH	ST1_G	ST0_G	ST1_XL	ST0_XL

Table 61. CTRL5_C register description

ROUNDING[2:0]	Circular burst-mode (rounding) read from the output registers.<br>Default value: 000<br>(000: no rounding; Others: refer to *Table 62*
DEN_LH	DEN active level configuration. Default value: 0<br>(0: active low; 1: active high)
ST_G [1:0]	Angular rate sensor self-test enable. Default value: 00<br>(00: Self-test disabled; Other: refer to *Table 63)*
ST_XL [1:0]	Linear acceleration sensor self-test enable. Default value: 00<br>(00: Self-test disabled; Other: refer to *Table 64*)

Table 62. Output registers rounding pattern

ROUNDING[2:0]	Rounding pattern
000	No rounding
001	Accelerometer only
010	Gyroscope only
011	Gyroscope + accelerometer
100	Registers from *SENSORHUB1_REG (2Eh)* to *SENSORHUB6_REG (33h)* only
101	Accelerometer + registers from *SENSORHUB1_REG (2Eh)* to *SENSORHUB6_REG (33h)*
110	Gyroscope + accelerometer + registers from *SENSORHUB1_REG (2Eh)* to *SENSORHUB6_REG (33h)* and registers from *SENSORHUB7_REG (34h)* to *SENSORHUB12_REG (39h)*
111	Gyroscope + accelerometer + registers from *SENSORHUB1_REG (2Eh)* to *SENSORHUB6_REG (33h)*

Table 63. Angular rate sensor self-test mode selection

ST1_G	ST0_G	Self-test mode
0	0	Normal mode
0	1	Positive sign self-test
1	0	Not allowed
1	1	Negative sign self-test

Table 64. Linear acceleration sensor self-test mode selection

ST1_XL	ST0_XL	Self-test mode
0	0	Normal mode
0	1	Positive sign self-test
1	0	Negative sign self-test
1	1	Not allowed

9.18 CTRL6_C (15h)

Angular rate sensor control register 6 (r/w).

Table 65. CTRL6_C register

TRIG_EN	LVL_EN	LVL2_EN	XL_HM_MODE	USR_ OFF_W	0(1)	FTYPE_1	FTYPE_0

1. This bit must be set to ‘0’ for the correct operation of the device.

Table 66. CTRL6_C register description

TRIG_EN	DEN data edge-sensitive trigger enable. Refer to *Table 67*.
LVL_EN	DEN data level-sensitive trigger enable. Refer to *Table 67*.
LVL2_EN	DEN level-sensitive latched enable. Refer to *Table 67*.
XL_HM_MODE	High-performance operating mode disable for accelerometer. Default value: 0<br>(0: high-performance operating mode enabled;<br>1: high-performance operating mode disabled)
USR_OFF_W	Weight of XL user offset bits of registers *X_OFS_USR (73h)*, *Y_OFS_USR (74h)*, *Z_OFS_USR (75h)*<br>0 = 2^{-10} *g*/LSB<br>1 = 2^{-6} *g*/LSB
FTYPE[1:0]	Gyroscope's low-pass filter (LPF1) bandwidth selection<br>*Table 68* shows the selectable bandwidth values.

Table 67. Trigger mode selection

TRIG_EN, LVL1_EN, LVL2_EN	Trigger mode
100	Edge-sensitive trigger mode is selected
010	Level-sensitive trigger mode is selected
011	Level-sensitive latched mode is selected
110	Level-sensitive FIFO enable mode is selected

Table 68. Gyroscope LPF1 bandwidth selection

FTYPE[1:0]	Bandwidth			
	ODR = 800 Hz	ODR = 1.6 kHz	ODR = 3.3 kHz	ODR = 6.6 kHz
00	245 Hz	315 Hz	343 Hz	351 Hz
01	195 Hz	224 Hz	234 Hz	237 Hz
10	155 Hz	168 Hz	172 Hz	173 Hz
11	293 Hz	505 Hz	925 Hz	937 Hz

9.19 CTRL7_G (16h)

Angular rate sensor control register 7 (r/w).

Table 69. CTRL7_G register

G_HM_MODE	HP_EN_G	HPM1_G	HPM0_G	0[(1)]	ROUNDING_STATUS	0[(1)]	0[(1)]

1. This bit must be set to ‘0’ for the correct operation of the device.

Table 70. CTRL7_G register description

G_HM_MODE	High-performance operating mode disable for gyroscope(1). Default: 0<br>(0: high-performance operating mode enabled;<br>1: high-performance operating mode disabled)
HP_EN_G	Gyroscope digital high-pass filter enable. The filter is enabled only if the gyro is in HP mode. Default value: 0<br>(0: HPF disabled; 1: HPF enabled)
HPM_G[1:0]	Gyroscope digital HP filter cutoff selection. Default: 00<br>(00 = 16 mHz<br>01 = 65 mHz<br>10 = 260 mHz<br>11 = 1.04 Hz)
ROUNDING_STATUS	Source register rounding function on WAKE_UP_SRC (1Bh), TAP_SRC (1Ch), D6D_SRC (1Dh), STATUS_REG (1Eh), and FUNC_SRC (53h).<br>Default value: 0<br>(0: Rounding disabled; 1: Rounding enabled)

9.20 CTRL8_XL (17h)

Linear acceleration sensor control register 8 (r/w).

Table 71. CTRL8_XL register

LPF2_XL_EN	HPCF_XL1	HPCF_XL0	HP_REF_MODE	INPUT_COMPOSITE	HP_SLOPE_XL_EN	0[(1)]	LOW_PASS_ON_6D

1. This bit must be set to ‘0’ for the correct operation of the device.

Table 72. CTRL8_XL register description

LPF2_XL_EN	Accelerometer low-pass filter LPF2 selection. Refer to *Figure 8*.
HPCF_XL[1:0]	Accelerometer LPF2 and high-pass filter configuration and cutoff setting. Refer to *Table 73*.
HP_REF_MODE	Enable HP filter reference mode. Default value: 0<br>(0: disabled; 1: enabled[(1)])
INPUT_COMPOSITE	Composite filter input selection. Default: 0<br>(0: ODR/2 low pass filtered sent to composite filter (default)<br>1: ODR/4 low pass filtered sent to composite filter)
HP_SLOPE_XL_EN	Accelerometer slope filter / high-pass filter selection. Refer to *Figure 8*.
LOW_PASS_ON_6D	LPF2 on 6D function selection. Refer to *Figure 8*.

1. When enabled, the first output data has to be discarded.

Table 73. Accelerometer bandwidth selection

HP_SLOPE_XL_EN	LPF2_XL_EN	LPF1_BW_SEL	HPCF_XL[1:0]	INPUT_COMPOSITE	Bandwidth
0 (low-pass path)[1]	0	0	-	-	ODR/2
		1	-	-	ODR/4
	1	-	00	1 (low noise)<br>0 (low latency)	ODR/50
			01		ODR/100
			10		ODR/9
			11		ODR/400
1 (high-pass path)[2]	-	-	00	0	ODR/4
			01		ODR/100
			10		ODR/9
			11		ODR/400

1. The bandwidth column is related to LPF1 if LPF2_XL_EN = 0 or to LPF2 if LPF2_XL_EN = 1.
2. The bandwidth column is related to the slope filter if HPCF_XL[1:0] = 00 or to the HP filter if HPCF_XL[1:0] = 01/10/11.

9.21 CTRL9_XL (18h)

Linear acceleration sensor control register 9 (r/w).

Table 74. CTRL9_XL register

DEN_X	DEN_Y	DEN_Z	DEN_XL_G	0[1]	SOFT_EN	0[1]	0[1]

1. This bit must be set to ‘0’ for the correct operation of the device.

Table 75. CTRL9_XL register description

DEN_X	DEN value stored in LSB of X-axis. Default value: 1<br>(0: DEN not stored in X-axis LSB; 1: DEN stored in X-axis LSB)
DEN_Y	DEN value stored in LSB of Y-axis. Default value: 1<br>(0: DEN not stored in Y-axis LSB; 1: DEN stored in Y-axis LSB)
DEN_Z	DEN value stored in LSB of Z-axis. Default value: 1<br>(0: DEN not stored in Z-axis LSB; 1: DEN stored in Z-axis LSB)
DEN_XL_G	DEN stamping sensor selection. Default value: 0<br>(0: DEN pin info stamped in the gyroscope axis selected by bits [7:5];<br>1: DEN pin info stamped in the accelerometer axis selected by bits [7:5])
SOFT_EN	Enable soft-iron correction algorithm for magnetometer[1]. Default value: 0<br>(0: soft-iron correction algorithm disabled;<br>1: soft-iron correction algorithm enabled)

1. This bit is effective if the IRON_EN bit of *MASTER_CONFIG (1Ah)* and the FUNC_EN bit of *CTRL10_C (19h)* are set to 1.

9.22 CTRL10_C (19h)

Control register 10 (r/w).

Table 76. CTRL10_C register

WRIST_ TILT_EN	0[(1)]	TIMER_ EN	PEDO_ EN	TILT_ EN	FUNC_EN	PEDO_RST _STEP	SIGN_ MOTION_EN

1. This bit must be set to ‘0’ for the correct operation of the device.

Table 77. CTRL10_C register description

WRIST_TILT_EN	Enable wrist tilt algorithm[(1)(2)]. Default value: 0<br>(0: wrist tilt algorithm disabled;<br>1: wrist tilt algorithm enabled)
TIMER_EN	Enable timestamp count. The count is saved in *TIMESTAMP0_REG (40h)*, *TIMESTAMP1_REG (41h)* and *TIMESTAMP2_REG (42h)*. Default: 0<br>(0: timestamp count disabled; 1: timestamp count enabled)
PEDO_EN	Enable pedometer algorithm[(1)]. Default value: 0<br>(0: pedometer algorithm disabled;<br>1: pedometer algorithm enabled)
TILT_EN	Enable tilt calculation[(1)].
FUNC_EN	Enable embedded functionalities (pedometer, tilt, significant motion detection, sensor hub and ironing). Default value: 0<br>(0: disable functionalities of embedded functions and accelerometer filters;<br>1: enable functionalities of embedded functions and accelerometer filters)
PEDO_RST_ STEP	Reset pedometer step counter. Default value: 0<br>(0: disabled; 1: enabled)
SIGN_MOTION_EN	Enable significant motion detection function[(1)]. Default value: 0<br>(0: disabled; 1: enabled)

1. This is effective if the FUNC_EN bit is set to '1'.
2. By default, the wrist tilt algorithm is applied to the positive X-axis.

9.23 MASTER_CONFIG (1Ah)

Master configuration register (r/w).

Table 78. MASTER_CONFIG register

DRDY_ON _INT1	DATA_VALID _SEL_FIFO	0[(1)]	START_ CONFIG	PULL_UP _EN	PASS_ THROUGH _MODE	IRON_EN	MASTER_ ON

1. This bit must be set to ‘0’ for the correct operation of the device.

Table 79. MASTER_CONFIG register description

DRDY_ON_ INT1	Manage the Master DRDY signal on INT1 pad. Default: 0<br>(0: disable Master DRDY on INT1; 1: enable Master DRDY on INT1)
DATA_VALID_ SEL_FIFO	Selection of FIFO data-valid signal. Default value: 0<br>(0: data-valid signal used to write data in FIFO is the XL/Gyro data-ready or step detection(1);<br>1: data-valid signal used to write data in FIFO is the sensor hub data-ready)
START_ CONFIG	Sensor Hub trigger signal selection. Default value: 0<br>(0: Sensor hub signal is the XL/Gyro data-ready;<br>1: Sensor hub signal external from INT2 pad.)
PULL_UP_EN	Auxiliary I^2C pull-up. Default value: 0<br>(0: internal pull-up on auxiliary I^2C line disabled;<br>1: internal pull-up on auxiliary I^2C line enabled)
PASS_THROUGH _MODE	I^2C interface pass-through. Default value: 0<br>(0: pass-through disabled; 1: pass-through enabled)
IRON_EN	Enable hard-iron correction algorithm for magnetometer(2). Default value: 0<br>(0:hard-iron correction algorithm disabled;<br>1: hard-iron correction algorithm enabled)
MASTER_ON	Sensor hub I^2C master enable(2). Default: 0<br>(0: master I^2C of sensor hub disabled; 1: master I^2C of sensor hub enabled)

1. If the TIMER_PEDO_FIFO_DRDY bit in *FIFO_CTRL2 (07h)* is set to 0, the trigger for writing data in FIFO is XL/Gyro data-ready, otherwise it's the step detection.
2. This is effective if the FUNC_EN bit is set to '1'.

9.24 WAKE_UP_SRC (1Bh)

Wake up interrupt source register (r).

Table 80. WAKE_UP_SRC register

0	0	FF_IA	SLEEP_ STATE_IA	WU_IA	X_WU	Y_WU	Z_WU

Table 81. WAKE_UP_SRC register description

FF_IA	Free-fall event detection status. Default: 0<br>(0: free-fall event not detected; 1: free-fall event detected)
SLEEP_ STATE_IA	Sleep event status. Default value: 0<br>(0: sleep event not detected; 1: sleep event detected)
WU_IA	Wakeup event detection status. Default value: 0<br>(0: wakeup event not detected; 1: wakeup event detected.)
X_WU	Wakeup event detection status on X-axis. Default value: 0<br>(0: wakeup event on X-axis not detected; 1: wakeup event on X-axis detected)
Y_WU	Wakeup event detection status on Y-axis. Default value: 0<br>(0: wakeup event on Y-axis not detected; 1: wakeup event on Y-axis detected)
Z_WU	Wakeup event detection status on Z-axis. Default value: 0<br>(0: wakeup event on Z-axis not detected; 1: wakeup event on Z-axis detected)

9.25 TAP_SRC (1Ch)

Tap source register (r).

Table 82. TAP_SRC register

0	TAP_IA	SINGLE_ TAP	DOUBLE_ TAP	TAP_SIGN	X_TAP	Y_TAP	Z_TAP

Table 83. TAP_SRC register description

TAP_IA	Tap event detection status. Default: 0<br>(0: tap event not detected; 1: tap event detected)
SINGLE_TAP	Single-tap event status. Default value: 0<br>(0: single tap event not detected; 1: single tap event detected)
DOUBLE_TAP	Double-tap event detection status. Default value: 0<br>(0: double-tap event not detected; 1: double-tap event detected.)
TAP_SIGN	Sign of acceleration detected by tap event. Default: 0<br>(0: positive sign of acceleration detected by tap event;<br>1: negative sign of acceleration detected by tap event)
X_TAP	Tap event detection status on X-axis. Default value: 0<br>(0: tap event on X-axis not detected; 1: tap event on X-axis detected)
Y_TAP	Tap event detection status on Y-axis. Default value: 0<br>(0: tap event on Y-axis not detected; 1: tap event on Y-axis detected)
Z_TAP	Tap event detection status on Z-axis. Default value: 0<br>(0: tap event on Z-axis not detected; 1: tap event on Z-axis detected)

9.26 D6D_SRC (1Dh)

Portrait, landscape, face-up and face-down source register (r)

Table 84. D6D_SRC register

DEN_ DRDY	D6D_IA	ZH	ZL	YH	YL	XH	XL

Table 85. D6D_SRC register description

DEN_ DRDY	DEN data-ready signal. It is set high when data output is related to the data coming from a DEN active condition.(1)
D6D_ IA	Interrupt active for change position portrait, landscape, face-up, face-down. Default value: 0<br>(0: change position not detected; 1: change position detected)
ZH	Z-axis high event (over threshold). Default value: 0<br>(0: event not detected; 1: event (over threshold) detected)
ZL	Z-axis low event (under threshold). Default value: 0<br>(0: event not detected; 1: event (under threshold) detected)
YH	Y-axis high event (over threshold). Default value: 0<br>(0: event not detected; 1: event (over-threshold) detected)
YL	Y-axis low event (under threshold). Default value: 0<br>(0: event not detected; 1: event (under threshold) detected)
XH	X-axis high event (over threshold). Default value: 0<br>(0: event not detected; 1: event (over threshold) detected)
XL	X-axis low event (under threshold). Default value: 0<br>(0: event not detected; 1: event (under threshold) detected)

1. The DEN data-ready signal can be latched or pulsed depending on the value of the dataready_pulsed bit of the *DRDY_PULSE_CFG_G (0Bh)* register.

9.27 STATUS_REG (1Eh)

The STATUS_REG register is read by the SPI/I^{2}C interface (r).

Table 86. STATUS_REG register

0	0	0	0	0	TDA	GDA	XLDA

Table 87. STATUS_REG register description

TDA	Temperature new data available. Default: 0<br>(0: no set of data is available at temperature sensor output;<br>1: a new set of data is available at temperature sensor output)
GDA	Gyroscope new data available. Default value: 0<br>(0: no set of data available at gyroscope output;<br>1: a new set of data is available at gyroscope output)
XLDA	Accelerometer new data available. Default value: 0<br>(0: no set of data available at accelerometer output;<br>1: a new set of data is available at accelerometer output)

9.28 OUT_TEMP_L (20h), OUT_TEMP_H (21h)

Temperature data output register (r). L and H registers together express a 16-bit word in two's complement.

Table 88. OUT_TEMP_L register

Temp7	Temp6	Temp5	Temp4	Temp3	Temp2	Temp1	Temp0

Table 89. OUT_TEMP_H register

Temp15	Temp14	Temp13	Temp12	Temp11	Temp10	Temp9	Temp8

Table 90. OUT_TEMP register description

Temp[15:0]	Temperature sensor output data<br>The value is expressed as two's complement sign extended on the MSB.

9.29 OUTX_L_G (22h)

Angular rate sensor pitch axis (X) angular rate output register (r). The value is expressed as a 16-bit word in two's complement.

Data are according to the full-scale and ODR settings (*CTRL2_G (11h)*) of the gyro user interface.

Table 91. OUTX_L_G register

D7	D6	D5	D4	D3	D2	D1	D0

Table 92. OUTX_L_G register description

D[7:0]	Pitch axis (X) angular rate value (LSbyte)<br>D[15:0] expressed in two's complement and its value depends on the interface used:<br>SPI1/I^{2}C: Gyro UI chain pitch axis output

9.30 OUTX_H_G (23h)

Angular rate sensor pitch axis (X) angular rate output register (r). The value is expressed as a 16-bit word in two's complement.

Data are according to the full-scale and ODR settings (*CTRL2_G (11h)*) of the gyro user interface.

Table 93. OUTX_H_G register

D15	D14	D13	D12	D11	D10	D9	D8

Table 94. OUTX_H_G register description

D[15:8]	Pitch axis (X) angular rate value (MSbyte)<br>D[15:0] expressed in two's complement and its value depends on the interface used:<br>SPI1/I^{2}C: Gyro UI chain pitch axis output

9.31 OUTY_L_G (24h)

Angular rate sensor roll axis (Y) angular rate output register (r). The value is expressed as a 16-bit word in two's complement.

Data are according to the full-scale and ODR settings (*CTRL2_G (11h)*) of the gyro user interface.

Table 95. OUTY_L_G register

D7	D6	D5	D4	D3	D2	D1	D0

Table 96. OUTY_L_G register description

D[7:0]	Roll axis (Y) angular rate value (LSbyte)<br>D[15:0] expressed in two's complement and its value depends on the interface used:<br>SPI1/I^{2}C: Gyro UI chain roll axis output

9.32 OUTY_H_G (25h)

Angular rate sensor roll axis (Y) angular rate output register (r). The value is expressed as a 16-bit word in two's complement.

Data are according to the full-scale and ODR settings (*CTRL2_G (11h)*) of the gyro user interface.

Table 97. OUTY_H_G register

D15	D14	D13	D12	D11	D10	D9	D8

Table 98. OUTY_H_G register description

D[15:8]	Roll axis (Y) angular rate value (MSbyte)<br>D[15:0] expressed in two's complement and its value depends on the interface used:<br>SPI1/I^{2}C: Gyro UI chain roll axis output

9.33 OUTZ_L_G (26h)

Angular rate sensor yaw axis (Z) angular rate output register (r). The value is expressed as a 16-bit word in two's complement.

Data are according to the full-scale and ODR settings (*CTRL2_G (11h)*) of the gyro user interface.

Table 99. OUTZ_L_G register

D7	D6	D5	D4	D3	D2	D1	D0

Table 100. OUTZ_L_G register description

D[7:0]	Yaw axis (Z) angular rate value (LSbyte)<br>D[15:0] expressed in two's complement and its value depends on the interface used:<br>SPI1/I^{2}C: Gyro UI chain yaw axis output

9.34 OUTZ_H_G (27h)

Angular rate sensor Yaw axis (Z) angular rate output register (r). The value is expressed as a 16-bit word in two's complement.

Data are according to the full-scale and ODR settings (*CTRL2_G (11h)*) of the gyro user interface.

Table 101. OUTZ_H_G register

D15	D14	D13	D12	D11	D10	D9	D8

Table 102. OUTZ_H_G register description

D[15:8]	Yaw axis (Z) angular rate value (MSbyte)<br>D[15:0] expressed in two's complement and its value depends on the interface used:<br>SPI1/I^{2}C: Gyro UI chain yaw axis output

9.35 OUTX_L_XL (28h)

Linear acceleration sensor X-axis output register (r). The value is expressed as a 16-bit word in two's complement.

Table 103. OUTX_L_XL register

D7	D6	D5	D4	D3	D2	D1	D0

Table 104. OUTX_L_XL register description

D[7:0]	X-axis linear acceleration value (LSbyte)

9.36 OUTX_H_XL (29h)

Linear acceleration sensor X-axis output register (r). The value is expressed as a 16-bit word in two's complement.

Table 105. OUTX_H_XL register

D15	D14	D13	D12	D11	D10	D9	D8

Table 106. OUTX_H_XL register description

D[15:8]	X-axis linear acceleration value (MSbyte)

9.37 OUTY_L_XL (2Ah)

Linear acceleration sensor Y-axis output register (r). The value is expressed as a 16-bit word in two's complement.

Table 107. OUTY_L_XL register

D7	D6	D5	D4	D3	D2	D1	D0

Table 108. OUTY_L_XL register description

D[7:0]	Y-axis linear acceleration value (LSbyte)

9.38 OUTY_H_XL (2Bh)

Linear acceleration sensor Y-axis output register (r). The value is expressed as a 16-bit word in two's complement.

Table 109. OUTY_H_G register

D15	D14	D13	D12	D11	D10	D9	D8

Table 110. OUTY_H_G register description

D[15:8]	Y-axis linear acceleration value (MSbyte)

9.39 OUTZ_L_XL (2Ch)

Linear acceleration sensor Z-axis output register (r). The value is expressed as a 16-bit word in two's complement.

Table 111. OUTZ_L_XL register

D7	D6	D5	D4	D3	D2	D1	D0

Table 112. OUTZ_L_XL register description

D[7:0]	Z-axis linear acceleration value (LSbyte)

9.40 OUTZ_H_XL (2Dh)

Linear acceleration sensor Z-axis output register (r). The value is expressed as a 16-bit word in two's complement.

Table 113. OUTZ_H_XL register

D15	D14	D13	D12	D11	D10	D9	D8

Table 114. OUTZ_H_XL register description

D[15:8]	Z-axis linear acceleration value (MSbyte)

9.41 SENSORHUB1_REG (2Eh)

First byte associated to external sensors. The content of the register is consistent with the SLAVEx_CONFIG number of read operation configurations (for external sensors from x = 0 to x = 3).

Table 115. SENSORHUB1_REG register

SHub1_7	SHub1_6	SHub1_5	SHub1_4	SHub1_3	SHub1_2	SHub1_1	SHub1_0

Table 116. SENSORHUB1_REG register description

SHub1_[7:0]	First byte associated to external sensors

9.42 SENSORHUB2_REG (2Fh)

Second byte associated to external sensors. The content of the register is consistent with the SLAVEx_CONFIG number of read operations configurations (for external sensors from x = 0 to x = 3).

Table 117. SENSORHUB2_REG register

SHub2_7	SHub2_6	SHub2_5	SHub2_4	SHub2_3	SHub2_2	SHub2_1	SHub2_0

Table 118. SENSORHUB2_REG register description

SHub2_[7:0]	Second byte associated to external sensors

9.43 SENSORHUB3_REG (30h)

Third byte associated to external sensors. The content of the register is consistent with the SLAVEx_CONFIG number of read operations configurations (for external sensors from x = 0 to x = 3).

Table 119. SENSORHUB3_REG register

SHub3_7	SHub3_6	SHub3_5	SHub3_4	SHub3_3	SHub3_2	SHub3_1	SHub3_0

Table 120. SENSORHUB3_REG register description

SHub3_[7:0]	Third byte associated to external sensors

9.44 SENSORHUB4_REG (31h)

Fourth byte associated to external sensors. The content of the register is consistent with the SLAVEx_CONFIG number of read operation configurations (for external sensors from x = 0 to x = 3).

Table 121. SENSORHUB4_REG register

SHub4_7	SHub4_6	SHub4_5	SHub4_4	SHub4_3	SHub4_2	SHub4_1	SHub4_0

Table 122. SENSORHUB4_REG register description

SHub4_[7:0]	Fourth byte associated to external sensors

9.45 SENSORHUB5_REG (32h)

Fifth byte associated to external sensors. The content of the register is consistent with the SLAVEx_CONFIG number of read operation configurations (for external sensors from x = 0 to x = 3).

Table 123. SENSORHUB5_REG register

SHub5_7	SHub5_6	SHub5_5	SHub5_4	SHub5_3	SHub5_2	SHub5_1	SHub5_0

Table 124. SENSORHUB5_REG register description

SHub5_[7:0]	Fifth byte associated to external sensors

9.46 SENSORHUB6_REG (33h)

Sixth byte associated to external sensors. The content of the register is consistent with the SLAVEx_CONFIG number of read operation configurations (for external sensors from x = 0 to x = 3).

Table 125. SENSORHUB6_REG register

SHub6_7	SHub6_6	SHub6_5	SHub6_4	SHub6_3	SHub6_2	SHub6_1	SHub6_0

Table 126. SENSORHUB6_REG register description

SHub6_[7:0]	Sixth byte associated to external sensors

9.47 SENSORHUB7_REG (34h)

Seventh byte associated to external sensors. The content of the register is consistent with the SLAVEx_CONFIG number of read operation configurations (for external sensors from x = 0 to x = 3).

Table 127. SENSORHUB7_REG register

SHub7_7	SHub7_6	SHub7_5	SHub7_4	SHub7_3	SHub7_2	SHub7_1	SHub7_0

Table 128. SENSORHUB7_REG register description

SHub7_[7:0]	Seventh byte associated to external sensors

9.48 SENSORHUB8_REG(35h)

Eighth byte associated to external sensors. The content of the register is consistent with the SLAVEx_CONFIG number of read operation configurations (for external sensors from x = 0 to x = 3).

Table 129. SENSORHUB8_REG register

SHub8_7	SHub8_6	SHub8_5	SHub8_4	SHub8_3	SHub8_2	SHub8_1	SHub8_0

Table 130. SENSORHUB8_REG register description

SHub8_[7:0]	Eighth byte associated to external sensors

9.49 SENSORHUB9_REG (36h)

Ninth byte associated to external sensors. The content of the register is consistent with the SLAVEx_CONFIG number of read operation configurations (for external sensors from x = 0 to x = 3).

Table 131. SENSORHUB9_REG register

SHub9_7	SHub9_6	SHub9_5	SHub9_4	SHub9_3	SHub9_2	SHub9_1	SHub9_0

Table 132. SENSORHUB9_REG register description

SHub9_[7:0]	Ninth byte associated to external sensors

9.50 SENSORHUB10_REG (37h)

Tenth byte associated to external sensors. The content of the register is consistent with the SLAVEx_CONFIG number of read operation configurations (for external sensors from x = 0 to x = 3).

Table 133. SENSORHUB10_REG register

SHub10_7	SHub10_6	SHub10_5	SHub10_4	SHub10_3	SHub10_2	SHub10_1	SHub10_0

Table 134. SENSORHUB10_REG register description

SHub10_[7:0]	Tenth byte associated to external sensors

9.51 SENSORHUB11_REG (38h)

Eleventh byte associated to external sensors. The content of the register is consistent with the SLAVEx_CONFIG number of read operation configurations (for external sensors from x = 0 to x = 3).

Table 135. SENSORHUB11_REG register

SHub11_7	SHub11_6	SHub11_5	SHub11_4	SHub11_3	SHub11_2	SHub11_1	SHub11_0

Table 136. SENSORHUB11_REG register description

SHub11_[7:0]	Eleventh byte associated to external sensors

9.52 SENSORHUB12_REG (39h)

Twelfth byte associated to external sensors. The content of the register is consistent with the SLAVEx_CONFIG number of read operation configurations (for external sensors from x = 0 to x = 3).

Table 137. SENSORHUB12_REG register

SHub12_7	SHub12_6	SHub12_5	SHub12_4	SHub12_3	SHub12_2	SHub12_1	SHub12_0

Table 138. SENSORHUB12_REG register description

SHub12[7:0]	Twelfth byte associated to external sensors

9.53 FIFO_STATUS1 (3Ah)

FIFO status control register (r). For a proper reading of the register, it is recommended to set the BDU bit in *CTRL3_C (12h)* to 1.

Table 139. FIFO_STATUS1 register

DIFF_ FIFO_7	DIFF_ FIFO_6	DIFF_ FIFO_5	DIFF_ FIFO_4	DIFF_ FIFO_3	DIFF_ FIFO_2	DIFF_ FIFO_1	DIFF_ FIFO_0

Table 140. FIFO_STATUS1 register description

DIFF_FIFO_[7:0]	Number of unread words (16-bit axes) stored in FIFO(1).

1. For a complete number of unread samples, consider DIFF_FIFO [10:8] in *FIFO_STATUS2 (3Bh)*.

9.54 FIFO_STATUS2 (3Bh)

FIFO status control register (r). For a proper reading of the register, it is recommended to set the BDU bit in *CTRL3_C (12h)* to 1.

Table 141. FIFO_STATUS2 register

WaterM	OVER_RUN	FIFO_ FULL_ SMART	FIFO_ EMPTY	0	DIFF_ FIFO_10	DIFF_ FIFO_9	DIFF_ FIFO_8

Table 142. FIFO_STATUS2 register description

WaterM	FIFO watermark status. The watermark is set through bits FTH_[7:0] in *FIFO_CTRL1 (06h)*. Default value: 0<br>(0: FIFO filling is lower than watermark level(1);<br>1: FIFO filling is equal to or higher than the watermark level)
OVER_RUN	FIFO overrun status. Default value: 0<br>(0: FIFO is not completely filled; 1: FIFO is completely filled)
FIFO_FULL_ SMART	Smart FIFO full status. Default value: 0<br>(0: FIFO is not full; 1: FIFO will be full at the next ODR)
FIFO_EMPTY	FIFO empty bit. Default value: 0<br>(0: FIFO contains data; 1: FIFO is empty)
DIFF_FIFO_[10:8]	Number of unread words (16-bit axes) stored in FIFO(2).

1. FIFO watermark level is set in FTH_[10:0] in *FIFO_CTRL1 (06h)* and *FIFO_CTRL2 (07h)*.
2. For a complete number of unread samples, consider DIFF_FIFO [7:0] in *FIFO_STATUS1 (3Ah)*.

9.55 FIFO_STATUS3 (3Ch)

FIFO status control register (r). For a proper reading of the register, it is recommended to set the BDU bit in *CTRL3_C (12h)* to 1.

Table 143. FIFO_STATUS3 register

FIFO_ PATTERN _7	FIFO_ PATTERN _6	FIFO_ PATTERN _5	FIFO_ PATTERN _4	FIFO_ PATTERN _3	FIFO_ PATTERN _2	FIFO_ PATTERN _1	FIFO_ PATTERN _0

Table 144. FIFO_STATUS3 register description

FIFO_ PATTERN_[7:0]	Word of recursive pattern read at the next reading.

9.56 FIFO_STATUS4 (3Dh)

FIFO status control register (r). For a proper reading of the register, it is recommended to set the BDU bit in *CTRL3_C (12h)* to 1.

Table 145. FIFO_STATUS4 register

0	0	0	0	0	0	FIFO_ PATTERN_9	FIFO_ PATTERN_8

Table 146. FIFO_STATUS4 register description

FIFO_ PATTERN_[9:8]	Word of recursive pattern read at the next reading.

9.57 FIFO_DATA_OUT_L (3Eh)

FIFO data output register (r). For a proper reading of the register, it is recommended to set the BDU bit in *CTRL3_C (12h)* to 1.

Table 147. FIFO_DATA_OUT_L register

DATA_ OUT_ FIFO_L_7	DATA_ OUT_ FIFO_L_6	DATA_ OUT_ FIFO_L_5	DATA_ OUT_ FIFO_L_4	DATA_ OUT_ FIFO_L_3	DATA_ OUT_ FIFO_L_2	DATA_ OUT_ FIFO_L_1	DATA_ OUT_ FIFO_L_0

Table 148. FIFO_DATA_OUT_L register description

DATA_OUT_FIFO_L_[7:0]	FIFO data output (first byte)

9.58 FIFO_DATA_OUT_H (3Fh)

FIFO data output register (r). For a proper reading of the register, it is recommended to set the BDU bit in *CTRL3_C (12h)* to 1.

Table 149. FIFO_DATA_OUT_H register

DATA_ OUT_ FIFO_H_7	DATA_ OUT_ FIFO_H_6	DATA_ OUT_ FIFO_H_5	DATA_ OUT_ FIFO_H_4	DATA_ OUT_ FIFO_H_3	DATA_ OUT_ FIFO_H_2	DATA_ OUT_ FIFO_H_1	DATA_ OUT_ FIFO_H_0

Table 150. FIFO_DATA_OUT_H register description

DATA_OUT_FIFO_H_[7:0]	FIFO data output (second byte)

9.59 TIMESTAMP0_REG (40h)

Timestamp first (least significant) byte data output register (r). The value is expressed as a 24-bit word and the bit resolution is defined by setting the value in *WAKE_UP_DUR (5Ch)*.

Table 151. TIMESTAMP0_REG register

TIMESTAMP0_7	TIMESTAMP0_6	TIMESTAMP0_5	TIMESTAMP0_4	TIMESTAMP0_3	TIMESTAMP0_2	TIMESTAMP0_1	TIMESTAMP0_0

Table 152. TIMESTAMP0_REG register description

TIMESTAMP0_[7:0]	TIMESTAMP first byte data output

9.60 TIMESTAMP1_REG (41h)

Timestamp second byte data output register (r). The value is expressed as a 24-bit word and the bit resolution is defined by setting value in *WAKE_UP_DUR (5Ch)*.

Table 153. TIMESTAMP1_REG register

TIMESTAMP1_7	TIMESTAMP1_6	TIMESTAMP1_5	TIMESTAMP1_4	TIMESTAMP1_3	TIMESTAMP1_2	TIMESTAMP1_1	TIMESTAMP1_0

Table 154. TIMESTAMP1_REG register description

TIMESTAMP1_[7:0]	TIMESTAMP second byte data output

9.61 TIMESTAMP2_REG (42h)

Timestamp third (most significant) byte data output register (r/w). The value is expressed as a 24-bit word and the bit resolution is defined by setting the value in *WAKE_UP_DUR (5Ch)*. To reset the timer, the AAh value has to be stored in this register.

Table 155. TIMESTAMP2_REG register

TIMESTAMP2_7	TIMESTAMP2_6	TIMESTAMP2_5	TIMESTAMP2_4	TIMESTAMP2_3	TIMESTAMP2_2	TIMESTAMP2_1	TIMESTAMP2_0

Table 156. TIMESTAMP2_REG register description

TIMESTAMP2_[7:0]	TIMESTAMP third byte data output

9.62 STEP_TIMESTAMP_L (49h)

Step counter timestamp information register (r). When a step is detected, the value of TIMESTAMP_REG1 register is copied in STEP_TIMESTAMP_L.

Table 157. STEP_TIMESTAMP_L register

STEP_ TIMESTA MP_L_7	STEP_ TIMESTA MP_L_6	STEP_ TIMESTA MP_L_5	STEP_ TIMESTA MP_L_4	STEP_ TIMESTA MP_L_3	STEP_ TIMESTA MP_L_2	STEP_ TIMESTA MP_L_1	STEP_ TIMESTA MP_L_0

Table 158. STEP_TIMESTAMP_L register description

STEP_TIMESTAMP_L[7:0]	Timestamp of last step detected.

9.63 STEP_TIMESTAMP_H (4Ah)

Step counter timestamp information register (r). When a step is detected, the value of TIMESTAMP_REG2 register is copied in STEP_TIMESTAMP_H.

Table 159. STEP_TIMESTAMP_H register

STEP_ TIMESTA MP_H_7	STEP_ TIMESTA MP_H_6	STEP_ TIMESTA MP_H_5	STEP_ TIMESTA MP_H_4	STEP_ TIMESTA MP_H_3	STEP_ TIMESTA MP_H_2	STEP_ TIMESTA MP_H_1	STEP_ TIMESTA MP_H_0

Table 160. STEP_TIMESTAMP_H register description

STEP_TIMESTAMP_H[7:0]	Timestamp of last step detected.

9.64 STEP_COUNTER_L (4Bh)

Step counter output register (r).

Table 161. STEP_COUNTER_L register

STEP_CO UNTER_L _7	STEP_CO UNTER_L _6	STEP_CO UNTER_L _5	STEP_CO UNTER_L _4	STEP_CO UNTER_L _3	STEP_CO UNTER_L _2	STEP_CO UNTER_L _1	STEP_CO UNTER_L _0

Table 162. STEP_COUNTER_L register description

STEP_COUNTER_L_[7:0]	Step counter output (LSbyte)

9.65 STEP_COUNTER_H (4Ch)

Step counter output register (r).

Table 163. STEP_COUNTER_H register

STEP_COUNTER_H_7	STEP_COUNTER_H_6	STEP_COUNTER_H_5	STEP_COUNTER_H_4	STEP_COUNTER_H_3	STEP_COUNTER_H_2	STEP_COUNTER_H_1	STEP_COUNTER_H_0

Table 164. STEP_COUNTER_H register description

STEP_COUNTER_H_[7:0]	Step counter output (MSbyte)

9.66 SENSORHUB13_REG (4Dh)

Thirteenth byte associated to external sensors. The content of the register is consistent with the SLAVEx_CONFIG number of read operation configurations (for external sensors from x = 0 to x = 3).

Table 165. SENSORHUB13_REG register

SHub13_7	SHub13_6	SHub13_5	SHub13_4	SHub13_3	SHub13_2	SHub13_1	SHub13_0

Table 166. SENSORHUB13_REG register description

SHub13_[7:0]	Thirteenth byte associated to external sensors

9.67 SENSORHUB14_REG (4Eh)

Fourteenth byte associated to external sensors. The content of the register is consistent with the SLAVEx_CONFIG number of read operation configurations (for external sensors from x = 0 to x = 3).

Table 167. SENSORHUB14_REG register

SHub14_7	SHub14_6	SHub14_5	SHub14_4	SHub14_3	SHub14_2	SHub14_1	SHub14_0

Table 168. SENSORHUB14_REG register description

SHub14_[7:0]	Fourteenth byte associated to external sensors

9.68 SENSORHUB15_REG (4Fh)

Fifteenth byte associated to external sensors. The content of the register is consistent with the SLAVEx_CONFIG number of read operation configurations (for external sensors from x = 0 to x = 3).

Table 169. SENSORHUB15_REG register

SHub15_7	SHub15_6	SHub15_5	SHub15_4	SHub15_3	SHub15_2	SHub15_1	SHub15_0

Table 170. SENSORHUB15_REG register description

SHub15_[7:0]	Fifteenth byte associated to external sensors

9.69 SENSORHUB16_REG (50h)

Sixteenth byte associated to external sensors. The content of the register is consistent with the SLAVEx_CONFIG number of read operation configurations (for external sensors from x = 0 to x = 3).

Table 171. SENSORHUB16_REG register

SHub16_7	SHub16_6	SHub16_5	SHub16_4	SHub16_3	SHub16_2	SHub16_1	SHub16_0

Table 172. SENSORHUB16_REG register description

SHub16_[7:0]	Sixteenth byte associated to external sensors

9.70 SENSORHUB17_REG (51h)

Seventeenth byte associated to external sensors. The content of the register is consistent with the SLAVEx_CONFIG number of read operation configurations (for external sensors from x = 0 to x = 3).

Table 173. SENSORHUB17_REG register

SHub17_7	SHub17_6	SHub17_5	SHub17_4	SHub17_3	SHub17_2	SHub17_1	SHub17_0

Table 174. SENSORHUB17_REG register description

SHub17_[7:0]	Seventeenth byte associated to external sensors

9.71 SENSORHUB18_REG (52h)

Eighteenth byte associated to external sensors. The content of the register is consistent with the SLAVEx_CONFIG number of read operation configurations (for external sensors from x = 0 to x = 3).

Table 175. SENSORHUB18_REG register

SHub18_7	SHub18_6	SHub18_5	SHub18_4	SHub18_3	SHub18_2	SHub18_1	SHub18_0

Table 176. SENSORHUB18_REG register description

SHub18_[7:0]	Eighteenth byte associated to external sensors

9.72 FUNC_SRC1 (53h)

Significant motion, tilt, step detector, hard/soft-iron and sensor hub interrupt source register (r).

Table 177. FUNC_SRC1 register

STEP_COUNT_DELTA_IA	SIGN_MOTION_IA	TILT_IA	STEP_DETECTED	STEP_OVERFLOW	HI_FAIL	SI_END_OP	SENSOR HUB_END_OP

Table 178. FUNC_SRC1 register description

STEP_COUNT_DELTA_IA	Pedometer step recognition on delta time status. Default value: 0<br>(0: no step recognized during delta time;<br>1: at least one step recognized during delta time)
SIGN_MOTION_IA	Significant motion event detection status. Default value: 0<br>(0: significant motion event not detected; 1: significant motion event detected)
TILT_IA	Tilt event detection status. Default value: 0<br>(0: tilt event not detected; 1: tilt event detected)
STEP_DETECTED	Step detector event detection status. Default value: 0<br>(0: step detector event not detected; 1: step detector event detected)
STEP_OVERFLOW	Step counter overflow status. Default value: 0<br>(0: step counter value < 2^{16}; 1: step counter value reached 2^{16})
HI_FAIL	Fail in hard/soft-ironing algorithm.
SI_END_OP	Hard/soft-iron calculation status. Default value: 0<br>(0: Hard/soft-iron calculation not concluded; 1: Hard/soft-iron calculation concluded)
SENSORHUB_END_OP	Sensor hub communication status. Default value: 0<br>(0: sensor hub communication not concluded;<br>1: sensor hub communication concluded)

9.73 FUNC_SRC2 (54h)

Wrist tilt interrupt source register (r).

Table 179. FUNC_SRC2 register

0	SLAVE3_NACK	SLAVE2_NACK	SLAVE1_NACK	SLAVE0_NACK	0	0	WRIST_TILT_IA

Table 180. FUNC_SRC2 register description

SLAVE3_NACK	This bit is set to 1 if Not acknowledge occurs on slave 3 communication. Default value: 0
SLAVE2_NACK	This bit is set to 1 if Not acknowledge occurs on slave 2 communication. Default value: 0
SLAVE1_NACK	This bit is set to 1 if Not acknowledge occurs on slave 1 communication. Default value: 0
SLAVE0_NACK	This bit is set to 1 if Not acknowledge occurs on slave 0 communication. Default value: 0
WRIST_TILT_IA	Wrist tilt event detection status. Default value: 0<br>(0: Wrist tilt event not detected; 1: Wrist tilt event detected)

9.74 WRIST_TILT_IA (55h)

Wrist tilt interrupt source register (r).

Table 181. WRIST_TILT_IA register

WRIST_ TILT_IA_ Xpos	WRIST_ TILT_IA_ Xneg	WRIST_ TILT_IA_ Ypos	WRIST_ TILT_IA_ Yneg	WRIST_ TILT_IA_ Zpos	WRIST_ TILT_IA_ Zneg	0	0

Table 182. WRIST_TILT_IA register description

WRIST_ TILT_IA_ Xpos	Absolute Wrist Tilt event detection status on X-positive axis. Default value: 0<br>(0: Absolute Wrist Tilt event on X-positive axis not detected;<br>1: Absolute Wrist Tilt event on X-positive axis detected)
WRIST_ TILT_IA_ Xneg	Absolute Wrist Tilt event detection status on X-negative axis. Default value: 0<br>(0: Absolute Wrist Tilt event on X-negative axis not detected;<br>1: Absolute Wrist Tilt event on X-negative axis detected)
WRIST_ TILT_IA_ Ypos	Absolute Wrist Tilt event detection status on Y-positive axis. Default value: 0<br>(0: Absolute Wrist Tilt event on Y-positive axis not detected;<br>1: Absolute Wrist Tilt event on Y-positive axis detected)
WRIST_ TILT_IA_ Yneg	Absolute Wrist Tilt event detection status on Y-negative axis. Default value: 0<br>(0: Absolute Wrist Tilt event on Y-negative axis not detected;<br>1: Absolute Wrist Tilt event on Y-negative axis detected)
WRIST_ TILT_IA_ Zpos	Absolute Wrist Tilt event detection status on Z-positive axis. Default value: 0<br>(0: Absolute Wrist Tilt event on Z-positive axis not detected;<br>1: Absolute Wrist Tilt event on Z-positive axis detected)
WRIST_ TILT_IA_ Zneg	Absolute Wrist Tilt event detection status on Z-negative axis. Default value: 0<br>(0: Absolute Wrist Tilt event on Z-negative axis not detected;<br>1: Absolute Wrist Tilt event on Z-negative axis detected)

9.75 TAP_CFG (58h)

Enables interrupt and inactivity functions, configuration of filtering and tap recognition functions (r/w).

Table 183. TAP_CFG register

INTERRUPTS _ENABLE	INACT_EN1	INACT_EN0	SLOPE _FDS	TAP_X_EN	TAP_Y_EN	TAP_Z_EN	LIR

Table 184. TAP_CFG register description

INTERRUPTS _ENABLE	Enable basic interrupts (6D/4D, free-fall, wake-up, tap, inactivity). Default 0.<br>(0: interrupt disabled; 1: interrupt enabled)
INACT_EN[1:0]	Enable inactivity function. Default value: 00<br>(00: disabled<br>01: sets accelerometer ODR to 12.5 Hz (low-power mode), gyro does not change;<br>10: sets accelerometer ODR to 12.5 Hz (low-power mode), gyro to sleep mode;<br>11: sets accelerometer ODR to 12.5 Hz (low-power mode), gyro to power-down mode)
SLOPE_FDS	HPF or SLOPE filter selection on wake-up and Activity/Inactivity functions. Refer to *Figure 8*. Default value: 0<br>(0: SLOPE filter applied; 1: HPF applied)
TAP_X_EN	Enable X direction in tap recognition. Default value: 0<br>(0: X direction disabled; 1: X direction enabled)
TAP_Y_EN	Enable Y direction in tap recognition. Default value: 0<br>(0: Y direction disabled; 1: Y direction enabled)
TAP_Z_EN	Enable Z direction in tap recognition. Default value: 0<br>(0: Z direction disabled; 1: Z direction enabled)
LIR	Latched Interrupt. Default value: 0<br>(0: interrupt request not latched; 1: interrupt request latched)

9.76 TAP_THS_6D (59h)

Portrait/landscape position and tap function threshold register (r/w).

Table 185. TAP_THS_6D register

D4D_EN	SIXD_THS 1	SIXD_THS 0	TAP_THS 4	TAP_THS 3	TAP_THS 2	TAP_THS 1	TAP_THS 0

Table 186. TAP_THS_6D register description

D4D_EN	4D orientation detection enable. Z-axis position detection is disabled.<br>Default value: 0<br>(0: enabled; 1: disabled)
SIXD_THS[1:0]	Threshold for 4D/6D function. Default value: 00<br>For details, refer to *Table 187*.
TAP_THS[4:0]	Threshold for tap recognition. Default value: 00000<br>1 LSb corresponds to FS_XL/2^5

Table 187. Threshold for D4D/D6D function

SIXD_THS[1:0]	Threshold value
00	80 degrees
01	70 degrees
10	60 degrees
11	50 degrees

9.77 INT_DUR2 (5Ah)

Tap recognition function setting register (r/w).

Table 188. INT_DUR2 register

DUR3	DUR2	DUR1	DUR0	QUIET1	QUIET0	SHOCK1	SHOCK0

Table 189. INT_DUR2 register description

DUR[3:0]	Duration of maximum time gap for double tap recognition. Default: 0000<br>When double tap recognition is enabled, this register expresses the maximum time between two consecutive detected taps to determine a double tap event. The default value of these bits is 0000b which corresponds to 16*ODR_XL time. If the DUR[3:0] bits are set to a different value, 1LSB corresponds to 32*ODR_XL time.
QUIET[1:0]	Expected quiet time after a tap detection. Default value: 00<br>Quiet time is the time after the first detected tap in which there must not be any overthreshold event. The default value of these bits is 00b which corresponds to 2*ODR_XL time. If the QUIET[1:0] bits are set to a different value, 1LSB corresponds to 4*ODR_XL time.
SHOCK[1:0]	Maximum duration of overthreshold event. Default value: 00<br>Maximum duration is the maximum time of an overthreshold signal detection to be recognized as a tap event. The default value of these bits is 00b which corresponds to 4*ODR_XL time. If the SHOCK[1:0] bits are set to a different value, 1LSB corresponds to 8*ODR_XL time.

9.78 WAKE_UP_THS (5Bh)

Single and double-tap function threshold register (r/w).

Table 190. WAKE_UP_THS register

SINGLE_ DOUBLE _TAP	0	WK_THS5	WK_THS4	WK_THS3	WK_THS2	WK_THS1	WK_THS0

Table 191. WAKE_UP_THS register description

SINGLE_DOUBLE_TAP	Single/double-tap event enable. Default: 0<br>(0: only single-tap event enabled;<br>1: both single and double-tap events enabled)
WK_THS[5:0]	Threshold for wakeup. Default value: 000000<br>1 LSb corresponds to FS_XL/2^6

9.79 WAKE_UP_DUR (5Ch)

Free-fall, wakeup, timestamp and sleep mode functions duration setting register (r/w).

Table 192. WAKE_UP_DUR register

FF_DUR5	WAKE_ DUR1	WAKE_ DUR0	TIMER_ HR	SLEEP_ DUR3	SLEEP_ DUR2	SLEEP_ DUR1	SLEEP_ DUR0

Table 193. WAKE_UP_DUR register description

FF_DUR5	Free fall duration event. Default: 0<br>For the complete configuration of the free-fall duration, refer to FF_DUR[4:0] in *FREE_FALL (5Dh)* configuration.<br>1 LSB = 1 ODR_time
WAKE_DUR[1:0]	Wake up duration event. Default: 00<br>1LSB = 1 ODR_time
TIMER_HR	Timestamp register resolution setting(1). Default value: 0<br>(0: 1LSB = 6.4 ms; 1: 1LSB = 25 μs)
SLEEP_DUR[3:0]	Duration to go in sleep mode. Default value: 0000 (this corresponds to 16 ODR)<br>1 LSB = 512 ODR

1. Configuration of this bit affects *TIMESTAMP0_REG (40h)*, *TIMESTAMP1_REG (41h)*, *TIMESTAMP2_REG (42h)*, *STEP_TIMESTAMP_L (49h)*, *STEP_TIMESTAMP_H (4Ah)*, and *CTRL6_C (15h)* registers.

9.80 FREE_FALL (5Dh)

Free-fall function duration setting register (r/w).

Table 194. FREE_FALL register

FF_DUR4	FF_DUR3	FF_DUR2	FF_DUR1	FF_DUR0	FF_THS2	FF_THS1	FF_THS0

Table 195. FREE_FALL register description

FF_DUR[4:0]	Free-fall duration event. Default: 0<br>For the complete configuration of the free fall duration, refer to FF_DUR5 in *WAKE_UP_DUR (5Ch)* configuration
FF_THS[2:0]	Free fall threshold setting. Default: 000<br>For details refer to *Table 196*.

Table 196. Threshold for free-fall function

FF_THS[2:0]	Threshold value
000	156 m*g*
001	219 m*g*
010	250 m*g*
011	312 m*g*
100	344 m*g*
101	406 m*g*
110	469 m*g*
111	500 m*g*

9.81 MD1_CFG (5Eh)

Functions routing on INT1 register (r/w).

Table 197. MD1_CFG register

INT1_ INACT_ STATE	INT1_ SINGLE_ TAP	INT1_WU	INT1_FF	INT1_ DOUBLE_ TAP	INT1_6D	INT1_TILT	INT1_ TIMER

Table 198. MD1_CFG register description

INT1_INACT_ STATE	Routing on INT1 of inactivity mode. Default: 0<br>(0: routing on INT1 of inactivity disabled; 1: routing on INT1 of inactivity enabled)
INT1_SINGLE_ TAP	Single-tap recognition routing on INT1. Default: 0<br>(0: routing of single-tap event on INT1 disabled;<br>1: routing of single-tap event on INT1 enabled)
INT1_WU	Routing of wakeup event on INT1. Default value: 0<br>(0: routing of wakeup event on INT1 disabled;<br>1: routing of wakeup event on INT1 enabled)
INT1_FF	Routing of free-fall event on INT1. Default value: 0<br>(0: routing of free-fall event on INT1 disabled;<br>1: routing of free-fall event on INT1 enabled)
INT1_DOUBLE _TAP	Routing of tap event on INT1. Default value: 0<br>(0: routing of double-tap event on INT1 disabled;<br>1: routing of double-tap event on INT1 enabled)
INT1_6D	Routing of 6D event on INT1. Default value: 0<br>(0: routing of 6D event on INT1 disabled; 1: routing of 6D event on INT1 enabled)
INT1_TILT	Routing of tilt event on INT1. Default value: 0<br>(0: routing of tilt event on INT1 disabled; 1: routing of tilt event on INT1 enabled)
INT1_TIMER	Routing of end counter event of timer on INT1. Default value: 0<br>(0: routing of end counter event of timer on INT1 disabled;<br>1: routing of end counter event of timer event on INT1 enabled)

9.82 MD2_CFG (5Fh)

Functions routing on INT2 register (r/w).

Table 199. MD2_CFG register

INT2_ INACT_ STATE	INT2_ SINGLE_ TAP	INT2_WU	INT2_FF	INT2_ DOUBLE_ TAP	INT2_6D	INT2_TILT	INT2_ IRON

Table 200. MD2_CFG register description

INT2_INACT_ STATE	Routing on INT2 of inactivity mode. Default: 0<br>(0: routing on INT2 of inactivity disabled; 1: routing on INT2 of inactivity enabled)
INT2_SINGLE_ TAP	Single-tap recognition routing on INT2. Default: 0<br>(0: routing of single-tap event on INT2 disabled;<br>1: routing of single-tap event on INT2 enabled)
INT2_WU	Routing of wakeup event on INT2. Default value: 0<br>(0: routing of wakeup event on INT2 disabled;<br>1: routing of wake-up event on INT2 enabled)
INT2_FF	Routing of free-fall event on INT2. Default value: 0<br>(0: routing of free-fall event on INT2 disabled;<br>1: routing of free-fall event on INT2 enabled)
INT2_DOUBLE _TAP	Routing of tap event on INT2. Default value: 0<br>(0: routing of double-tap event on INT2 disabled;<br>1: routing of double-tap event on INT2 enabled)
INT2_6D	Routing of 6D event on INT2. Default value: 0<br>(0: routing of 6D event on INT2 disabled; 1: routing of 6D event on INT2 enabled)
INT2_TILT	Routing of tilt event on INT2. Default value: 0<br>(0: routing of tilt event on INT2 disabled; 1: routing of tilt event on INT2 enabled)
INT2_IRON	Routing of soft-iron/hard-iron algorithm end event on INT2. Default value: 0<br>(0: routing of soft-iron/hard-iron algorithm end event on INT2 disabled;<br>1: routing of soft-iron/hard-iron algorithm end event on INT2 enabled)

9.83 MASTER_CMD_CODE (60h)

Table 201. MASTER_CMD_CODE register

MASTER_ CMD_ CODE7	MASTER_ CMD_ CODE6	MASTER_ CMD_ CODE5	MASTER_ CMD_ CODE4	MASTER_ CMD_ CODE3	MASTER_ CMD_ CODE2	MASTER_ CMD_ CODE1	MASTER_ CMD_ CODE0

Table 202. MASTER_CMD_CODE register description

MASTER_CMD_ CODE[7:0]	Master command code used for stamping for sensor sync. Default: 0

9.84 SENS_SYNC_SPI_ERROR_CODE (61h)

Table 203. SENS_SYNC_SPI_ERROR_CODE register

ERROR_ CODE7	ERROR_ CODE6	ERROR_ CODE5	ERROR_ CODE4	ERROR_ CODE3	ERROR_ CODE2	ERROR_ CODE1	ERROR_ CODE0

Table 204. SENS_SYNC_SPI_ERROR_CODE register description

ERROR_CODE[7:0]	Error code used for sensor synchronization. Default: 0)

9.85 OUT_MAG_RAW_X_L (66h)

External magnetometer raw data (r).

Table 205. OUT_MAG_RAW_X_L register

D7	D6	D5	D4	D3	D2	D1	D0

Table 206. OUT_MAG_RAW_X_L register description

D[7:0]	X-axis external magnetometer value (LSbyte)

9.86 OUT_MAG_RAW_X_H (67h)

External magnetometer raw data (r).

Table 207. OUT_MAG_RAW_X_H register

D15	D14	D13	D12	D11	D10	D9	D8

Table 208. OUT_MAG_RAW_X_H register description

D[15:8]	X-axis external magnetometer value (MSbyte)

9.87 OUT_MAG_RAW_Y_L (68h)

External magnetometer raw data (r).

Table 209. OUT_MAG_RAW_Y_L register

D7	D6	D5	D4	D3	D2	D1	D0

Table 210. OUT_MAG_RAW_Y_L register description

D[7:0]	Y-axis external magnetometer value (LSbyte)

9.88 OUT_MAG_RAW_Y_H (69h)

External magnetometer raw data (r).

Table 211. OUT_MAG_RAW_Y_H register

D15	D14	D13	D12	D11	D10	D9	D8

Table 212. OUT_MAG_RAW_Y_H register description

D[15:8]	Y-axis external magnetometer value (MSbyte)

9.89 OUT_MAG_RAW_Z_L (6Ah)

External magnetometer raw data (r).

Table 213. OUT_MAG_RAW_Z_L register

D7	D6	D5	D4	D3	D2	D1	D0

Table 214. OUT_MAG_RAW_Z_L register description

D[7:0]	Z-axis external magnetometer value (LSbyte)

9.90 OUT_MAG_RAW_Z_H (6Bh)

External magnetometer raw data (r).

Table 215. OUT_MAG_RAW_Z_H register

D15	D14	D13	D12	D11	D10	D9	D8

Table 216. OUT_MAG_RAW_Z_H register description

D[15:8]	Z-axis external magnetometer value (MSbyte)

9.91 X_OFS_USR (73h)

Accelerometer X-axis user offset correction (r/w). The offset value set in the X_OFS_USR offset register is internally added to the acceleration value measured on the X-axis.

Table 217. X_OFS_USR register

X_OFS_USR_7	X_OFS_USR_6	X_OFS_USR_5	X_OFS_USR_4	X_OFS_USR_3	X_OFS_USR_2	X_OFS_USR_1	X_OFS_USR_0

Table 218. X_OFS_USR register description

X_OFS_USR_[7:0]	Accelerometer X-axis user offset correction expressed in two's complement, weight depends on CTRL6_C(4) bit. The value must be in the range [-127 127].

9.92 Y_OFS_USR (74h)

Accelerometer Y-axis user offset correction (r/w). The offset value set in the Y_OFS_USR offset register is internally added to the acceleration value measured on the Y-axis.

Table 219. Y_OFS_USR register

Y_OFS_USR_7	Y_OFS_USR_6	Y_OFS_USR_5	Y_OFS_USR_4	Y_OFS_USR_3	Y_OFS_USR_2	Y_OFS_USR_1	Y_OFS_USR_0

Table 220. Y_OFS_USR register description

Y_OFS_USR_[7:0]	Accelerometer Y-axis user offset correction expressed in two's complement, weight depends on CTRL6_C(4) bit. The value must be in the range [-127 127].

9.93 Z_OFS_USR (75h)

Accelerometer Z-axis user offset correction (r/w). The offset value set in the Z_OFS_USR offset register is internally subtracted from the acceleration value measured on the Z-axis.

Table 221. Z_OFS_USR register

Z_OFS_USR_7	Z_OFS_USR_6	Z_OFS_USR_5	Z_OFS_USR_4	Z_OFS_USR_3	Z_OFS_USR_2	Z_OFS_USR_1	Z_OFS_USR_0

Table 222. Z_OFS_USR register description

Z_OFS_USR_[7:0]	Accelerometer Z-axis user offset correction expressed in two's complement, weight depends on CTRL6_C(4) bit. The value must be in the range [-127 127].

10 Embedded functions register mapping

The tables given below provide a list of the first (A) and second (B) bank registers related to the embedded functions available in the device and the corresponding addresses.

The embedded functions registers of bank A are accessible when FUNC_CFG_EN is set to ‘1’ in *FUNC_CFG_ACCESS (01h)*.

The embedded functions registers of bank B are accessible when both FUNC_CFG_EN and FUNC_CFG_EN_B set to ‘1’ in *FUNC_CFG_ACCESS (01h)*.

Note: All modifications of the content of the embedded functions registers have to be performed with the device in power-down mode.

Table 223. Register address map - Bank A - embedded functions

Name	Type	Register address		Default	Comment
		Hex	Binary		
SLV0_ADD	r/w	02	00000010	00000000	
SLV0_SUBADD	r/w	03	00000011	00000000	
SLAVE0_CONFIG	r/w	04	00000100	00000000	
SLV1_ADD	r/w	05	00000101	00000000	
SLV1_SUBADD	r/w	06	00000110	00000000	
SLAVE1_CONFIG	r/w	07	00000111	00000000	
SLV2_ADD	r/w	08	00001000	00000000	
SLV2_SUBADD	r/w	09	00001001	00000000	
SLAVE2_CONFIG	r/w	0A	00001010	00000000	
SLV3_ADD	r/w	0B	00001011	00000000	
SLV3_SUBADD	r/w	0C	00001100	00000000	
SLAVE3_CONFIG	r/w	0D	00001101	00000000	
DATAWRITE_SRC_ MODE_SUB_SLV0	r/w	0E	00001110	00000000	
CONFIG_PEDO_THS_MIN	r/w	0F	00001111	00010000	
RESERVED	-	10-12			Reserved
SM_THS	r/w	13	00010011	00000110	
PEDO_DEB_REG	r/w	14	00010100	01101110	
STEP_COUNT_DELTA	r/w	15	0001 0101	00000000	
MAG_SI_XX	r/w	24	00100100	00001000	
MAG_SI_XY	r/w	25	00100101	00000000	
MAG_SI_XZ	r/w	26	00100110	00000000	
MAG_SI_YX	r/w	27	00100111	00000000	
MAG_SI_YY	r/w	28	00101000	00001000	

Table 223. Register address map - Bank A - embedded functions (continued)

Name	Type	Register address		Default	Comment
		Hex	Binary		
MAG_SI_YZ	r/w	29	00101001	00000000	
MAG_SI_ZX	r/w	2A	00101010	00000000	
MAG_SI_ZY	r/w	2B	00101011	00000000	
MAG_SI_ZZ	r/w	2C	00101100	00001000	
MAG_OFFX_L	r/w	2D	00101101	00000000	
MAG_OFFX_H	r/w	2E	00101110	00000000	
MAG_OFFY_L	r/w	2F	00101111	00000000	
MAG_OFFY_H	r/w	30	00110000	00000000	
MAG_OFFZ_L	r/w	31	00110001	00000000	
MAG_OFFZ_H	r/w	32	00110010	00000000	

Table 224. Register address map - Bank B - embedded functions

Name	Type	Register address		Default	Comment
		Hex	Binary		
A_WRIST_TILT_LAT	r/w	50	01010000	00001111	
RESERVED	-	51-53			Reserved
A_WRIST_TILT_THS	r/w	54	01010100	00100000	
RESERVED	-	55-58			Reserved
A_WRIST_TILT_Mask	r/w	59	01011001	11000000	

Registers marked as *Reserved* must not be changed. Writing to those registers may cause permanent damage to the device.

The content of the registers that are loaded at boot should not be changed. They contain the factory calibration values. Their content is automatically restored when the device is powered up.

11 Embedded functions registers description - Bank A

Note: All modifications of the content of the embedded functions registers have to be performed with the device in power-down mode.

11.1 SLV0_ADD (02h)

I^{2}C slave address of the first external sensor (Sensor1) register (r/w).

Table 225. SLV0_ADD register

Slave0_ add6	Slave0_ add5	Slave0_ add4	Slave0_ add3	Slave0_ add2	Slave0_ add1	Slave0_ add0	rw_0

Table 226. SLV0_ADD register description

Slave0_add[6:0]	I^{2}C slave address of Sensor1 that can be read by sensor hub.<br>Default value: 0000000
rw_0	Read/write operation on Sensor1. Default value: 0<br>(0: write operation; 1: read operation)

11.2 SLV0_SUBADD (03h)

Address of register on the first external sensor (Sensor1) register (r/w).

Table 227. SLV0_SUBADD register

Slave0_ reg7	Slave0_ reg6	Slave0_ reg5	Slave0_ reg4	Slave0_ reg3	Slave0_ reg2	Slave0_ reg1	Slave0_ reg0

Table 228. SLV0_SUBADD register description

Slave0_reg[7:0]	Address of register on Sensor1 that has to be read/write according to the rw_0 bit value in *SLV0_ADD (02h)*. Default value: 00000000

11.3 SLAVE0_CONFIG (04h)

First external sensor (Sensor1) configuration and sensor hub settings register (r/w).

Table 229. SLAVE0_CONFIG register

Slave0_ rate1	Slave0_ rate0	Aux_sens _on1	Aux_sens _on0	Src_mode	Slave0_ numop2	Slave0_ numop1	Slave0_ numop0

Table 230. SLAVE0_CONFIG register description

Slave0_rate[1:0]	Decimation of read operation on Sensor1 starting from the sensor hub trigger. Default value: 00<br>(00: no decimation<br>01: update every 2 samples<br>10: update every 4 samples<br>11: update every 8 samples)
Aux_sens_on[1:0]	Number of external sensors to be read by sensor hub. Default value: 00<br>(00: one sensor<br>01: two sensors<br>10: three sensors<br>11: four sensors)
Src_mode	Source mode conditioned read(1). Default value: 0<br>(0: source mode read disabled; 1: source mode read enabled)
Slave0_numop[2:0]	Number of read operations on Sensor1.

1. Read conditioned by the content of the register at address specified in the *DATAWRITE_SRC_MODE_SUB_SLV0 (0Eh)*register. If the content is non-zero, the operation continues with the reading of the address specified in the *SLV0_SUBADD (03h)* register, else the operation is interrupted.

11.4 SLV1_ADD (05h)

I^2C slave address of the second external sensor (Sensor2) register (r/w).

Table 231. SLV1_ADD register

Slave1_ add6	Slave1_ add5	Slave1_ add4	Slave1_ add3	Slave1_ add2	Slave1_ add1	Slave1_ add0	r_1

Table 232. SLV1_ADD register description

Slave1_add[6:0]	I^2C slave address of Sensor2 that can be read by sensor hub.<br>Default value: 0000000
r_1	Read operation on Sensor2 enable. Default value: 0<br>(0: read operation disabled; 1: read operation enabled)

11.5 SLV1_SUBADD (06h)

Address of register on the second external sensor (Sensor2) register (r/w).

Table 233. SLV1_SUBADD register

Slave1_ reg7	Slave1_ reg6	Slave1_ reg5	Slave1_ reg4	Slave1_ reg3	Slave1_ reg2	Slave1_ reg1	Slave1_ reg0

Table 234. SLV1_SUBADD register description

Slave1_reg[7:0]	Address of register on Sensor2 that has to be read according to the r_1 bit value in *SLV1_ADD (05h)*. Default value: 00000000

11.6 SLAVE1_CONFIG (07h)

Second external sensor (Sensor2) configuration register (r/w).

Table 235. SLAVE1_CONFIG register

Slave1_ rate1	Slave1_ rate0	write_once	0(1)	0(1)	Slave1_ numop2	Slave1_ numop1	Slave1_ numop0

1. This bit must be set to '0' for the correct operation of the device.

Table 236. SLAVE1_CONFIG register description

Slave1_rate[1:0]	Decimation of read operation on Sensor2 starting from the sensor hub trigger. Default value: 00<br>(00: no decimation<br>01: update every 2 samples<br>10: update every 4 samples<br>11: update every 8 samples)
write_once	Slave 0 write operation is performed only at the first sensor hub cycle.(1)<br>Default value: 0<br>0: write operation for each sensor hub cycle<br>1: write operation only for the first sensor hub cycle
Slave1_numop[2:0]	Number of read operations on Sensor2.

1. This is effective if the Aux_sens_on[1:0] field in *SLAVE0_CONFIG (04h)* is set to a value other than 00.

11.7 SLV2_ADD (08h)

I^{2}C slave address of the third external sensor (Sensor3) register (r/w).

Table 237. SLV2_ADD register

Slave2_ add6	Slave2_ add5	Slave2_ add4	Slave2_ add3	Slave2_ add2	Slave2_ add1	Slave2_ add0	r_2

Table 238. SLV2_ADD register description

Slave2_add[6:0]	I^{2}C slave address of Sensor3 that can be read by sensor hub.<br>Default value: 0000000
r_2	Read operation on Sensor3 enable. Default value: 0<br>(0: read operation disabled; 1: read operation enabled)

11.8 SLV2_SUBADD (09h)

Address of register on the third external sensor (Sensor3) register (r/w).

Table 239. SLV2_SUBADD register

Slave2_ reg7	Slave2_ reg6	Slave2_ reg5	Slave2_ reg4	Slave2_ reg3	Slave2_ reg2	Slave2_ reg1	Slave2_ reg0

Table 240. SLV2_SUBADD register description

Slave2_reg[7:0]	Address of register on Sensor3 that has to be read according to the r_2 bit value in *SLV2_ADD (08h)*. Default value: 00000000

11.9 SLAVE2_CONFIG (0Ah)

Third external sensor (Sensor3) configuration register (r/w).

Table 241. SLAVE2_CONFIG register

Slave2_ rate1	Slave2_ rate0	0(1)	0(1)	0(1)	Slave2_ numop2	Slave2_ numop1	Slave2_ numop0

1. This bit must be set to ‘0’ for the correct operation of the device.

Table 242. SLAVE2_CONFIG register description

Slave2_rate[1:0]	Decimation of read operation on Sensor3 starting from the sensor hub trigger.<br>Default value: 00<br>(00: no decimation<br>01: update every 2 samples<br>10: update every 4 samples<br>11: update every 8 samples)
Slave2_numop[2:0]	Number of read operations on Sensor3.

11.10 SLV3_ADD (0Bh)

I^2C slave address of the fourth external sensor (Sensor4) register (r/w).

Table 243. SLV3_ADD register

Slave3_ add6	Slave3_ add5	Slave3_ add4	Slave3_ add3	Slave3_ add2	Slave3_ add1	Slave3_ add0	r_3

Table 244. SLV3_ADD register description

Slave3_add[6:0]	I^2C slave address of Sensor4 that can be read by the sensor hub.<br>Default value: 0000000
r_3	Read operation on Sensor4 enable. Default value: 0<br>(0: read operation disabled; 1: read operation enabled)

11.11 SLV3_SUBADD (0Ch)

Address of register on the fourth external sensor (Sensor4) register (r/w).

Table 245. SLV3_SUBADD register

Slave3_ reg7	Slave3_ reg6	Slave3_ reg5	Slave3_ reg4	Slave3_ reg3	Slave3_ reg2	Slave3_ reg1	Slave3_ reg0

Table 246. SLV3_SUBADD register description

Slave3_reg[7:0]	Address of register on Sensor4 that has to be read according to the r_3 bit value in *SLV3_ADD (0Bh)*. Default value: 00000000

11.12 SLAVE3_CONFIG (0Dh)

Fourth external sensor (Sensor4) configuration register (r/w).

Table 247. SLAVE3_CONFIG register

Slave3_ rate1	Slave3_ rate0	0(1)	0(1)	0(1)	Slave3_ numop2	Slave3_ numop1	Slave3_ numop0

1. This bit must be set to '0' for the correct operation of the device.

Table 248. SLAVE3_CONFIG register description

Slave3_rate[1:0]	Decimation of read operation on Sensor4 starting from the sensor hub trigger. Default value: 00<br>(00: no decimation<br>01: update every 2 samples<br>10: update every 4 samples<br>11: update every 8 samples)
Slave3_numop[2:0]	Number of read operations on Sensor4.

11.13 DATAWRITE_SRC_MODE_SUB_SLV0 (0Eh)

Data to be written into the slave device register (r/w).

Table 249. DATAWRITE_SRC_MODE_SUB_SLV0 register

Slave_ dataw7	Slave_ dataw6	Slave_ dataw5	Slave_ dataw4	Slave_ dataw3	Slave_ dataw2	Slave_ dataw1	Slave_ dataw0

Table 250. DATAWRITE_SRC_MODE_SUB_SLV0 register description

Slave_dataw[7:0]	Data to be written into the slave device according to the rw_0 bit in *SLV0_ADD (02h)* register or address to be read in source mode.<br>Default value: 00000000

11.14 CONFIG_PEDO_THS_MIN (0Fh)

Table 251. CONFIG_PEDO_THS_MIN register

PEDO_FS	0	0	ths_min_4	ths_min_3	ths_min_2	ths_min_1	ths_min_0

Table 252. CONFIG_PEDO_THS_MIN register description

PEDO_FS	Pedometer data elaboration at 4 *g*.<br>(0: elaboration of 2 *g* data;<br>1: elaboration of 4 *g* data)
ths_min_[4:0]	Minimum threshold to detect a peak. Default is 10h.

11.15 SM_THS (13h)

Significant motion configuration register (r/w).

Table 253. SM_THS register

SM_THS_7	SM_THS_6	SM_THS_5	SM_THS_4	SM_THS_3	SM_THS_2	SM_THS_1	SM_THS_0

Table 254. SM_THS register description

SM_THS[7:0]	Significant motion threshold. Default value: 00000110

11.16 PEDO_DEB_REG (14h)

Table 255. PEDO_DEB_REG register default values

DEB_TIME4	DEB_TIME3	DEB_TIME2	DEB_TIME1	DEB_TIME0	DEB_STEP2	DEB_STEP1	DEB_STEP0
0	1	1	0	1	1	1	0

Table 256. PEDO_DEB_REG register description

DEB_ TIME[4:0]	Debounce time. If the time between two consecutive steps is greater than DEB_TIME*80ms, the debouncer is reactivated. Default value: 01101
DEB_ STEP[2:0]	Debounce threshold. Minimum number of steps to increment step counter (debounce). Default value: 110

11.17 STEP_COUNT_DELTA (15h)

Time period register for step detection on delta time (r/w).

Table 257. STEP_COUNT_DELTA register

SC_DELTA_7	SC_DELTA_6	SC_DELTA_5	SC_DELTA_4	SC_DELTA_3	SC_DELTA_2	SC_DELTA_1	SC_DELTA_0

Table 258. STEP_COUNT_DELTA register description

SC_DELTA[7:0]	Time period value(1) (1LSB = 1.6384 s)

1. This value is effective if the TIMER_EN bit of *CTRL10_C (19h)* is set to 1 and the TIMER_HR bit of *WAKE_UP_DUR (5Ch)* register is set to 0.

11.18 MAG_SI_XX (24h)

Soft-iron matrix correction register (r/w).

Table 259. MAG_SI_XX register

MAG_SI_ XX_7	MAG_SI_ XX_6	MAG_SI_ XX_5	MAG_SI_ XX_4	MAG_SI_ XX_3	MAG_SI_ XX_2	MAG_SI_ XX_1	MAG_SI_ XX_0

Table 260. MAG_SI_XX register description

MAG_SI_XX_[7:0]	Soft-iron correction row1 col1 coefficient(1). Default value: 00001000

1. Value is expressed in sign-module format.

11.19 MAG_SI_XY (25h)

Soft-iron matrix correction register (r/w).

Table 261. MAG_SI_XY register

MAG_SI_ XY_7	MAG_SI_ XY_6	MAG_SI_ XY_5	MAG_SI_ XY_4	MAG_SI_ XY_3	MAG_SI_ XY_2	MAG_SI_ XY_1	MAG_SI_ XY_0

Table 262. MAG_SI_XY register description

MAG_SI_XY_[7:0]	Soft-iron correction row1 col2 coefficient(1). Default value: 00000000

1. Value is expressed in sign-module format.

11.20 MAG_SI_XZ (26h)

Soft-iron matrix correction register (r/w).

Table 263. MAG_SI_XZ register

MAG_SI_ XZ_7	MAG_SI_ XZ_6	MAG_SI_ XZ_5	MAG_SI_ XZ_4	MAG_SI_ XZ_3	MAG_SI_ XZ_2	MAG_SI_ XZ_1	MAG_SI_ XZ_0

Table 264. MAG_SI_XZ register description

MAG_SI_XZ_[7:0]	Soft-iron correction row1 col3 coefficient(1). Default value: 00000000

1. Value is expressed in sign-module format.

11.21 MAG_SI_YX (27h)

Soft-iron matrix correction register (r/w).

Table 265. MAG_SI_YX register

MAG_SI_ YX_7	MAG_SI_ YX_6	MAG_SI_ YX_5	MAG_SI_ YX_4	MAG_SI_ YX_3	MAG_SI_ YX_2	MAG_SI_ YX_1	MAG_SI_ YX_0

Table 266. MAG_SI_YX register description

MAG_SI_YX_[7:0]	Soft-iron correction row2 col1 coefficient(1). Default value: 00000000

1. Value is expressed in sign-module format.

11.22 MAG_SI_YY (28h)

Soft-iron matrix correction register (r/w).

Table 267. MAG_SI_YY register

MAG_SI_ YY_7	MAG_SI_ YY_6	MAG_SI_ YY_5	MAG_SI_ YY_4	MAG_SI_ YY_3	MAG_SI_ YY_2	MAG_SI_ YY_1	MAG_SI_ YY_0

Table 268. MAG_SI_YY register description

MAG_SI_YY_[7:0]	Soft-iron correction row2 col2 coefficient(1). Default value: 00001000

1. Value is expressed in sign-module format.

11.23 MAG_SI_YZ (29h)

Soft-iron matrix correction register (r/w).

Table 269. MAG_SI_YZ register

MAG_SI_ YZ_7	MAG_SI_ YZ_6	MAG_SI_ YZ_5	MAG_SI_ YZ_4	MAG_SI_ YZ_3	MAG_SI_ YZ_2	MAG_SI_ YZ_1	MAG_SI_ YZ_0

Table 270. MAG_SI_YZ register description

MAG_SI_YZ_[7:0]	Soft-iron correction row2 col3 coefficient(1). Default value: 00000000

1. Value is expressed in sign-module format.

11.24 MAG_SI_ZX (2Ah)

Soft-iron matrix correction register (r/w).

Table 271. MAG_SI_ZX register

MAG_SI_ ZX_7	MAG_SI_ ZX_6	MAG_SI_ ZX_5	MAG_SI_ ZX_4	MAG_SI_ ZX_3	MAG_SI_ ZX_2	MAG_SI_ ZX_1	MAG_SI_ ZX_0

Table 272. MAG_SI_ZX register description

MAG_SI_ZX_[7:0]	Soft-iron correction row3 col1 coefficient(1). Default value: 00000000

1. Value is expressed in sign-module format.

11.25 MAG_SI_ZY (2Bh)

Soft-iron matrix correction register (r/w).

Table 273. MAG_SI_ZY register

MAG_SI_ ZY_7	MAG_SI_ ZY_6	MAG_SI_ ZY_5	MAG_SI_ ZY_4	MAG_SI_ ZY_3	MAG_SI_ ZY_2	MAG_SI_ ZY_1	MAG_SI_ ZY_0

Table 274. MAG_SI_ZY register description

MAG_SI_ZY_[7:0]	Soft-iron correction row3 col2 coefficient(1). Default value: 00000000

1. Value is expressed in sign-module format.

11.26 MAG_SI_ZZ (2Ch)

Soft-iron matrix correction register (r/w).

Table 275. MAG_SI_ZZ register

MAG_SI_ ZZ_7	MAG_SI_ ZZ_6	MAG_SI_ ZZ_5	MAG_SI_ ZZ_4	MAG_SI_ ZZ_3	MAG_SI_ ZZ_2	MAG_SI_ ZZ_1	MAG_SI_ ZZ_0

Table 276. MAG_SI_ZZ register description

MAG_SI_ZZ_[7:0]	Soft-iron correction row3 col3 coefficient(1). Default value: 00001000

1. Value is expressed in sign-module format.

11.27 MAG_OFFX_L (2Dh)

Offset for X-axis hard-iron compensation register (r/w). The value is expressed as a 16-bit word in two's complement.

Table 277. MAG_OFFX_L register

MAG_OFF X_L_7	MAG_OFF X_L_6	MAG_OFF X_L_5	MAG_OFF X_L_4	MAG_OFF X_L_3	MAG_OFF X_L_2	MAG_OFF X_L_1	MAG_OFF X_L_0

Table 278. MAG_OFFX_L register description

MAG_OFFX_L_[7:0]	Offset for X-axis hard-iron compensation. Default value: 00000000

11.28 MAG_OFFX_H (2Eh)

Offset for X-axis hard-iron compensation register (r/w).The value is expressed as a 16-bit word in two's complement.

Table 279. MAG_OFFX_H register

MAG_OFF X_H_7	MAG_OFF X_H_6	MAG_OFF X_H_5	MAG_OFF X_H_4	MAG_OFF X_H_3	MAG_OFF X_H_2	MAG_OFF X_H_1	MAG_OFF X_H_0

Table 280. MAG_OFFX_H register description

MAG_OFFX_H_[7:0]	Offset for X-axis hard-iron compensation. Default value: 00000000

11.29 MAG_OFFY_L (2Fh)

Offset for Y-axis hard-iron compensation register (r/w). The value is expressed as a 16-bit word in two's complement.

Table 281. MAG_OFFY_L register

MAG_OFF Y_L_7	MAG_OFF Y_L_6	MAG_OFF Y_L_5	MAG_OFF Y_L_4	MAG_OFF Y_L_3	MAG_OFF Y_L_2	MAG_OFF Y_L_1	MAG_OFF Y_L_0

Table 282. MAG_OFFY_L register description

MAG_OFFY_L_[7:0]	Offset for Y-axis hard-iron compensation. Default value: 00000000

11.30 MAG_OFFY_H (30h)

Offset for Y-axis hard-iron compensation register (r/w). The value is expressed as a 16-bit word in two's complement.

Table 283. MAG_OFFY_H register

MAG_OFF Y_H_7	MAG_OFF Y_H_6	MAG_OFF Y_H_5	MAG_OFF Y_H_4	MAG_OFF Y_H_3	MAG_OFF Y_H_2	MAG_OFF Y_H_1	MAG_OFF Y_H_0

Table 284. MAG_OFFY_H register description

MAG_OFFY_H_[7:0]	Offset for Y-axis hard-iron compensation. Default value: 00000000

11.31 MAG_OFFZ_L (31h)

Offset for Z-axis hard-iron compensation register (r/w). The value is expressed as a 16-bit word in two's complement.

Table 285. MAG_OFFZ_L register

MAG_OFF Z_L_7	MAG_OFF Z_L_6	MAG_OFF Z_L_5	MAG_OFF Z_L_4	MAG_OFF Z_L_3	MAG_OFF Z_L_2	MAG_OFF Z_L_1	MAG_OFF Z_L_0

Table 286. MAG_OFFZ_L register description

MAG_OFFZ_L_[7:0]	Offset for Z-axis hard-iron compensation. Default value: 00000000

11.32 MAG_OFFZ_H (32h)

Offset for Z-axis hard-iron compensation register (r/w). The value is expressed as a 16-bit word in two's complement.

Table 287. MAG_OFFZ_H register

MAG_OFF Z_H_7	MAG_OFF Z_H_6	MAG_OFF Z_H_5	MAG_OFF Z_H_4	MAG_OFF Z_H_3	MAG_OFF Z_H_2	MAG_OFF Z_H_1	MAG_OFF Z_H_0

Table 288. MAG_OFFZ_H register description

MAG_OFFZ_H_[7:0]	Offset for Z-axis hard-iron compensation. Default value: 00000000

12 Embedded functions registers description - Bank B

12.1 A_WRIST_TILT_LAT (50h)

Absolute Wrist Tilt latency register (r/w).

Table 289. A_WRIST_TILT_LAT register

WRIST_TILT _ TIMER7	WRIST_TILT _ TIMER6	WRIST_TILT _ TIMER5	WRIST_TILT _ TIMER4	WRIST_TILT _ TIMER3	WRIST_TILT _ TIMER2	WRIST_TILT _ TIMER1	WRIST_TILT _ TIMER0

Table 290. A_WRIST_TILT_LAT register description

WRIST_TILT_TIMER[7:0]	Absolute wrist tilt latency parameters. 1 LSB = 40 ms.<br>Default value: 0Fh (600 ms)

12.2 A_WRIST_TILT_THS (54h)

Absolute Wrist Tilt threshold register (r/w).

Table 291. A_WRIST_TILT_THS register

WRIST_ TILT_ THS7	WRIST_ TILT_ THS6	WRIST_ TILT_ THS5	WRIST_ TILT_ THS4	WRIST_ TILT_ THS3	WRIST_ TILT_ THS2	WRIST_ TILT_ THS1	WRIST_ TILT_ THS0

Table 292. A_WRIST_TILT_THS register description

WRIST_TILT_THS[7:0]	Absolute wrist tilt threshold parameters. 1 LSB = 15.625 m*g*.<br>Default value: 20h (500 m*g*)

12.3 A_WRIST_TILT_Mask (59h)

Absolute Wrist Tilt mask register (r/w).

Table 293. A_WRIST_TILT_Mask register

WRIST_TILT_ MASK_ Xpos	WRIST_TILT_ MASK_ Xneg	WRIST_TILT_ MASK_ Ypos	WRIST_TILT_ MASK_Yneg	WRIST_TILT_ MASK_ Zpos	WRIST_TILT_ MASK_ Zneg	0	0

Table 294. A_WRIST_TILT_Mask register description

WRIST_TILT_MASK_ Xpos	Absolute wrist tilt positive X-axis enable. Default value: 1<br>(0: disable; 1: enable)
WRIST_TILT_MASK_ Xneg	Absolute wrist tilt negative X-axis enable. Default value: 1<br>(0: disable; 1: enable)
WRIST_TILT_MASK_ Ypos	Absolute wrist tilt positive Y-axis enable. Default value: 0<br>(0: disable; 1: enable)
WRIST_TILT_MASK_ Yneg	Absolute wrist tilt negative Y-axis enable. Default value: 0<br>(0: disable; 1: enable)
WRIST_TILT_MASK_ Zpos	Absolute wrist tilt positive Z-axis enable. Default value: 0<br>(0: disable; 1: enable)
WRIST_TILT_MASK_ Zneg	Absolute wrist tilt negative Z-axis enable. Default value:0<br>(0: disable; 1: enable)

13 Soldering information

The LGA package is compliant with the ECOPACK®, RoHS and "Green" standard.
It is qualified for soldering heat resistance according to JEDEC J-STD-020.

Land pattern and soldering recommendations are available at www.st.com/mems.

14 Package information

In order to meet environmental requirements, ST offers these devices in different grades of ECOPACK® packages, depending on their level of environmental compliance. ECOPACK® specifications, grade definitions and product status are available at: www.st.com. ECOPACK is an ST trademark.

14.1 LGA-14 package information

Figure 17. LGA-14 2.5x3x0.86 mm package outline and mechanical data

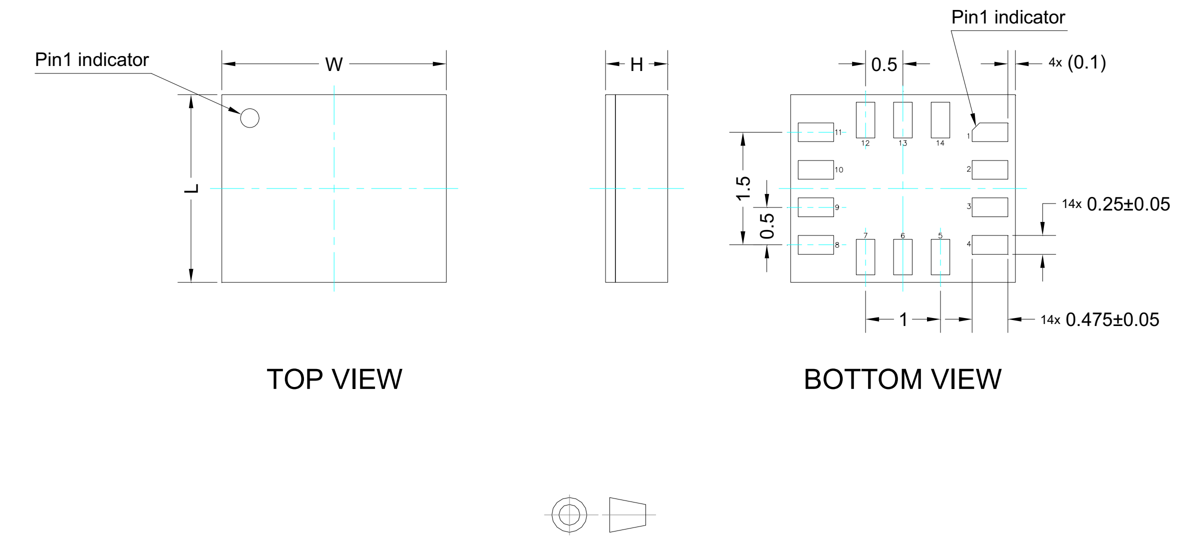

Dimensions are in millimeter unless otherwise specified
General tolerance is +/-0.1mm unless otherwise specified

OUTER DIMENSIONS

ITEM	DIMENSION [mm]	TOLERANCE [mm]
Length [L]	2.50	±0.1
Width [W]	3.00	±0.1
Height [H]	0.86	MAX

DM00249496_1

14.2 LGA-14 packing information

Figure 18. Carrier tape information for LGA-14 package

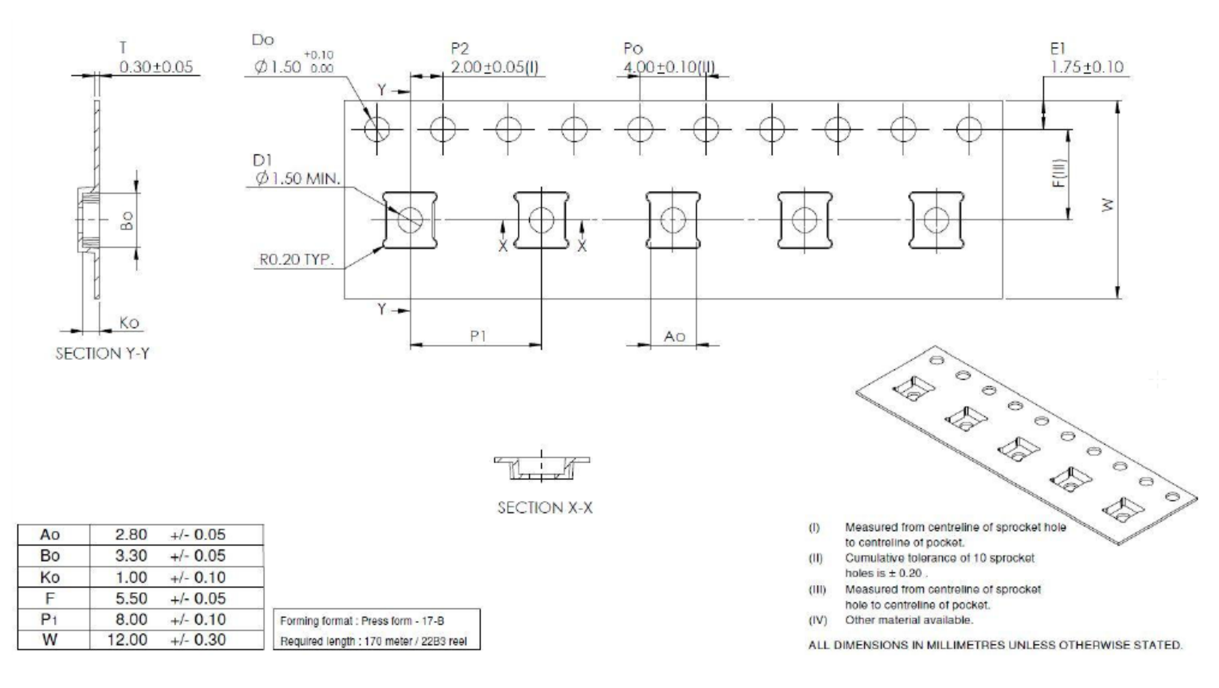

Figure 19. LGA-14 package orientation in carrier tape

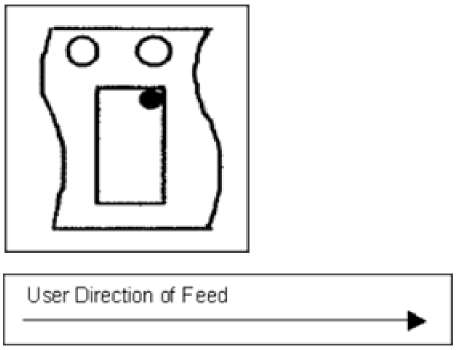

Figure 20. Reel information for carrier tape of LGA-14 package

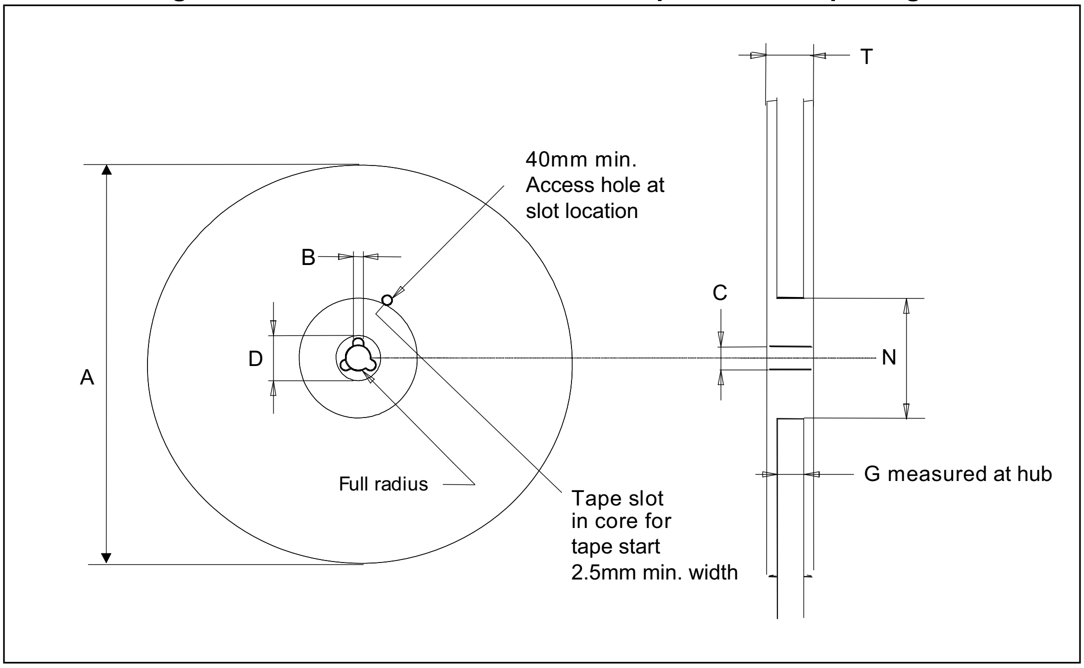

Table 295. Reel dimensions for carrier tape of LGA-14 package

Reel dimensions (mm)	
A (max)	330
B (min)	1.5
C	13 ±0.25
D (min)	20.2
N (min)	60
G	12.4 +2/-0
T (max)	18.4

15 Revision history

Table 296. Document revision history

Date	Revision	Changes
03-May-2017	6	Updated *Section 4.4.2: I²C - inter-IC control interface* (added *Table 8: I²C master timing values*)<br>Updated *Figure 11* and *Figure 13*<br>Updated pin status mode 1 and 2 for pins 10 and 11 as well as adding procedure to disable pull-ups in *Table 18: Internal pin status*<br>Updated bit 0 in *CTRL1_XL (10h)*<br>Updated *CTRL8_XL (17h)*<br>Updated description of SW_RESET bit in *Table 57: CTRL3_C register description*<br>Updated description of *X_OFS_USR (73h)*, *Y_OFS_USR (74h)*, *Z_OFS_USR (75h)*<br>Minor textual updates
29-Sep-2017	7	Updated *Table 3: Mechanical characteristics*<br>Specified SPI mode 3 in *Section 4.4.1: SPI - serial peripheral interface* and throughout *Section 6: Digital interfaces*

IMPORTANT NOTICE – PLEASE READ CAREFULLY

STMicroelectronics NV and its subsidiaries ("ST") reserve the right to make changes, corrections, enhancements, modifications, and improvements to ST products and/or to this document at any time without notice. Purchasers should obtain the latest relevant information on ST products before placing orders. ST products are sold pursuant to ST's terms and conditions of sale in place at the time of order acknowledgement.

Purchasers are solely responsible for the choice, selection, and use of ST products and ST assumes no liability for application assistance or the design of Purchasers' products.

No license, express or implied, to any intellectual property right is granted by ST herein.

Resale of ST products with provisions different from the information set forth herein shall void any warranty granted by ST for such product.

ST and the ST logo are trademarks of ST. All other product or service names are the property of their respective owners.

Information in this document supersedes and replaces information previously supplied in any prior versions of this document.

© 2017 STMicroelectronics – All rights reserved
