# Supplementary material for: The Unfolding Space Glove: A Wearable Spatio-Visual to Haptic Sensory Substitution Device for Blind People
Source: Sensors (Basel). 2022 Feb 26;22(5):1859. doi: 10.3390/s22051859 (PMC8914703; doi:10.3390/s22051859)
Supplement: Supplementary file 1 [file sensors-22-01859-s001.zip › S1_GitHub-Release_unfolding-space-hardware-0.2.1-revised.zip!/unfolding-space-hardware-0.2.1-revised/pcb/datasheets/MMC5633NJL.pdf]

±30 Gauss, Monolithic, High Performance, Low Cost 3-axis Magnetic Sensor

MMC5633NJL

FEATURES

- Monolithic integrated 3-axis AMR magnetic sensor and electronic circuits requiring fewer external components
- Superior Dynamic Range and Accuracy:
  - ✓ ±30 G FSR
  - ✓ 20bits operation mode
  - ✓ 0.0625mG per LSB resolution
  - ✓ 2 mG total RMS noise
  - ✓ Enables heading accuracy of ±1º
- Sensor true frequency response up to 1KHz
- Ultra-Small Wafer Level Package 0.85x0.85x0.4 mm
- On-chip automatic degaussing with built-in SET/RESET function
  - ✓ Eliminates thermal variation induced offset error (Null field output)
  - ✓ Clears the residual magnetization resulting from strong external fields
- On-chip sensitivity compensation
- On-chip temperature sensor
- Selftest signal available
- Data_ready Interrupt (I3C only)
- Low power consumption
- 1 μA power down current
- I^2C slave, FAST (≤400 KHz) mode
- I3C interface, Compatible with MIPI I3C specfication version 1.0
- 1.62V to 3.6V wide range single power supply
- 1.2V compatible I^2C interface
- 1.8V compatible I3C interface
- RoHS compliant

APPLICATIONS

- Electronic Compass & GPS Navigation
- Position Sensing

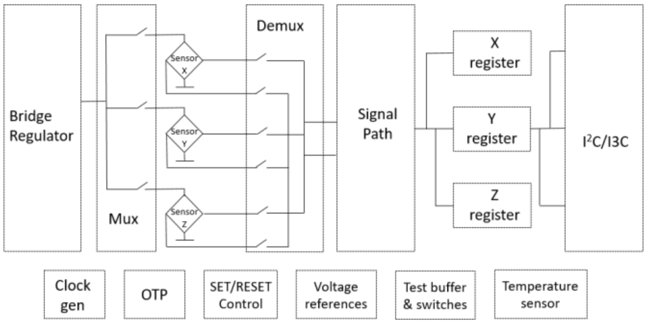

FUNCTIONAL BLOCK DIAGRAM

DESCRIPTION

The MMC5633NJL is a monolithic complete 3-axis AMR magnetic sensor with on-chip signal processing and integrated digital bus (I^2C fast mode and I3C interface), the device can be connected directly to a microprocessor, eliminating the need for A/D converters or timing resources.

It can measure magnetic fields within the full scale range of ±30 Gauss (G), with up to 0.0625mG per LSB resolution at 20bits operation mode and 2mG total RMS noise level, enabling heading accuracy of ±1º in electronic compass applications. Contact MEMSIC for access to advanced calibration and tilt-compensation algorithms.

An integrated SET/RESET function provides for the elimination of error due to Null Field output change with temperature. In addition, it clears the sensors of any residual magnetic polarization resulting from exposure to strong external magnets. The SET/RESET function can be performed for each measurement or periodically as the specific application requires.

The MMC5633NJL is in wafer level package with an ultra-small size of 0.85 x 0.85 x 0.4 mm and with an operating temperature range from -40 °C to +85 °C.

MEMSIC

Information furnished by MEMSIC is believed to be accurate and reliable. However, no responsibility is assumed by MEMSIC for its use, or for any infringements of patents or other rights of third parties which may result from its use. No license is granted by implication or otherwise under any patent or patent rights of MEMSIC.

©MEMSIC, Inc.
One Technology Drive, Suite 325, Andover, MA 01810, USA
Tel: +1 978 738 0900 Fax: +1 978 738 0196
www.memsic.com

SPECIFICATIONS (Measurements @ 25 °C, unless otherwise noted; V_{DD}= 1.8 V, Auto_SR_en=1, unless otherwise specified)

Parameter	Conditions	Min	Typ	Max	Units
Field Range (Each Axis)[1]	Total applied field		±30		G
Supply Voltage	V_{DD}	1.62	1.8	3.6	V
VIO	I^2C interface	1.2	1.8	V_{DD}	V
	I3C interface	1.5	1.8	V_{DD}	V
Supply Voltage rise time				10.0	ms
Supply Current[2,3] (100 measurements/second)	BW=00		3.4	4.0	mA
	BW=01		2.4	3.0	mA
	BW=10		1.3	1.6	mA
	BW=11		0.75	1.0	mA
Power Down Current[3]			1.0	1.2	µA
Operating Temperature		-40		85	°C
Storage Temperature		-55		125	°C
Linearity Error[3] (Best fit straight line)	FS=±30 G $H_{applied}$=±15 G		0.5	0.75	%FS
Hysteresis[3]	3 sweeps across ±30 G		0.02	0.1	%FS
Repeatability Error[3]	3 sweeps across ±30 G		0.02	0.1	%FS
Alignment Error			±1.0	±3.0	Degrees
Transverse Sensitivity			±2.0	±5.0	%
Total RMS Noise[3]	BW=00		1.5	2.5	mG
	BW=01		2.0	4.0	mG
	BW=10		3.0	5.0	mG
	BW=11		4.0	7.0	mG
Output resolution			20		Bits
Max Output data rate[4]	BW=00	75			Hz
	BW=01	150			Hz
	BW=10	255			Hz
	BW=11	255		1000	Hz
Heading accuracy[3,5]			±1.0	±3.0	Degrees
Sensitivity[3,6,8]	±30 G	-5		5	%
	With16bits operation		1024		counts/G
	With18bits operation		4096		counts/G
	With 20bits operation		16384		counts/G
Sensitivity Change Over Temperature[3]	-40~85 °C Delta from 25 °C±30 G			±5	%
Null Field Output[8]		-1.0		1.0	G
	With16bits operation		32768		Counts
	With18bits operation		131072		Counts
	With 20bits operation		524288		Counts
Null Field Output Change Over Temperature[3]			±0.2	±1.0	mG/°C
Temperature Sensor Output[3]		0.6	0.8	1.0	°C/Count
Disturbing Field[7]		32			G
Maximum Exposed Field				10,000	G
Output Repeatability[3]			2.0	3.0	mG

Notes:

1. External magnetic field on each axis not continuously higher than 30G.
2. Supply current is proportional to how many measurements performed per second, 75Hz (maximum) for BW=00.
3. Based on 3lots characterization result.
4. The 1000 Hz ODR is available by writing 255 into Register ODR and setting hpower to 1.
5. MEMSIC product enables users to utilize heading accuracy to be 1.0 degree typical when using MEMSIC’s proprietary software or algorithm.
6. Sensitivity of the orthogonal axes is analytically derived from raw data and is subsequently processed by MEMSIC software drivers.
7. This is the magnitude of external field that can be tolerated without changing the sensor characteristics. If the disturbing field is exceeded, a SET/RESET operation is required to restore proper sensor operation.
8. Based on shipment test result.

MEMSIC

I²C INTERFACE I/O CHARACTERISTICS (VIO=1.8 V)

Parameter	Symbol	Test Condition	Min.	Typ.	Max.	Unit
Logic Input Low Level	V_{IL}		-0.5		0.3* V_{IO}	V
Logic Input High Level	V_{IH}		0.7*V_{IO}		V_{IO}	V
Hysteresis of Schmitt input	V_{hys}		0.2			V
Logic Output Low Level	V_{OL}				0.4	V
Input Leakage Current	I_i	$0.1V_{IO}<V_{in}<0.9V_{IO}$	-10		10	µA
SCL Clock Frequency	f_{SCL}		0		400	kHz
START Hold Time	$t_{HD;STA}$		0.6			µS
START Setup Time	$t_{SU;STA}$		0.6			µS
LOW period of SCL	t_{LOW}		1.3			µS
HIGH period of SCL	t_{HIGH}		0.6			µS
Data Hold Time	$t_{HD;DAT}$		0		0.9	µS
Data Setup Time	$t_{SU;DAT}$		0.1			µS
Rise Time	t_r	From V_{IL} to V_{IH}			0.3	µS
Fall Time	t_f	From V_{IH} to V_{IL}			0.3	µS
Bus Free Time Between STOP and START	t_{BUF}		1.3			µS
STOP Setup Time	$t_{SU;STO}$		0.6			µS

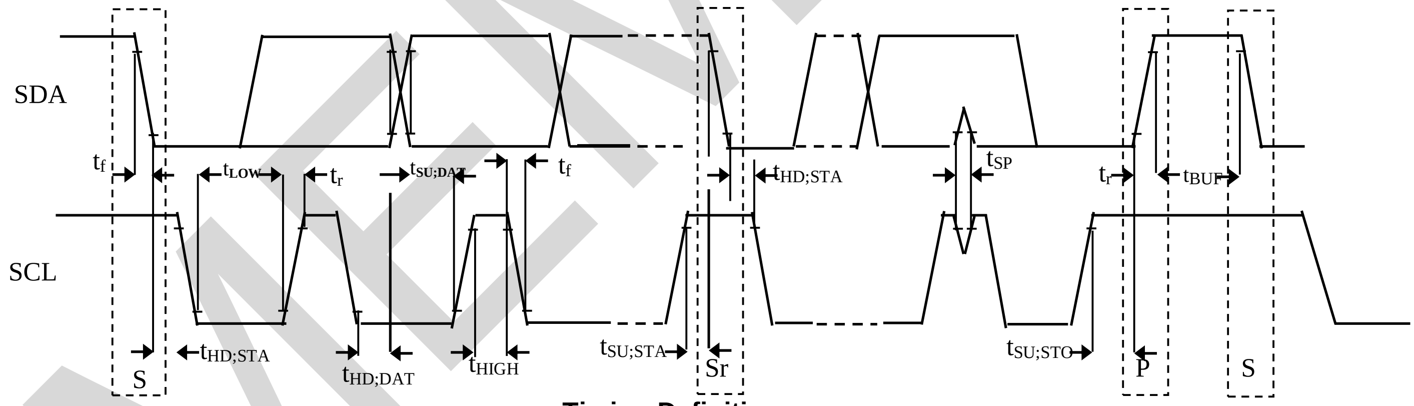

Timing Definition

MEMSIC

I3C INTERFACE I/O CHARACTERISTICS (VIO=1.8 V)

Parameter	Symbol	Test Condition	Min.	Typ.	Max.	Unit
Operating Voltage	V_{DDIO}		1.5		V_{DD}	V
Logic Input Low Level	V_{IL}		-0.5		0.3* V_{IO}	V
Logic Input High Level	V_{IH}		0.7*V_{IO}		V_{IO}	V
Logic Output Low Level	V_{OL}				0.2* V_{IO}	V
Input Leakage Current	I_i	$0.1V_{IO}<V_{in}<0.9V_{IO}$	-10		10	µA
Output High Level. Push-Pull only.	VOH	I_{OH} = -1mA	V_{IO}-0.27			V

Open Drain mode

Parameter	Symbol	Test Condition	Min.	Typ.	Max.	Unit
SCL clock "High" time	t_{HIGH}				41	ns
SCL clock "Low" time	t_{LOW}		200			ns
SDA fall time	$t_{FDA\_OD\_L}$				12.0	ns
Bus available condition	t_{AVAL}			1		µs
Bus idle condition	t_{IDLE}			1		ms

Push Pull mode

Parameter	Symbol	Test Condition	Min.	Typ.	Max.	Unit
SCL Clock Frequency	f_{SCL}		0.01	12.5	12.9	MHz
Clock After START(S) Condition	t_{CAS}		38.4			nS
START Setup Time	$t_{SU;STA}$		19.2			nS
LOW period of SCL	t_{LOW}		24			nS
HIGH period of SCL	t_{HIGH}		24			nS
SDA Signal Data Hold in Push_Pull Mode	$t_{HD;PP}$		t_{CR+3} and t_{CF+3}			
SDA Signal Data Setup in Push_Pull Mode	$t_{SU;PP}$		3			nS
SCL Clock Rise Time	t_{CR}				150*1/fSCL (capped at 60)	nS
SCL Clock Fall Time	t_{CF}				150*1/fSCL (capped at 60)	nS
Clock in to data out for Slaver	tsco				12	nS
Clock Before STOP(P) Condition	T_{CBP}		19.2			nS

MEMSIC

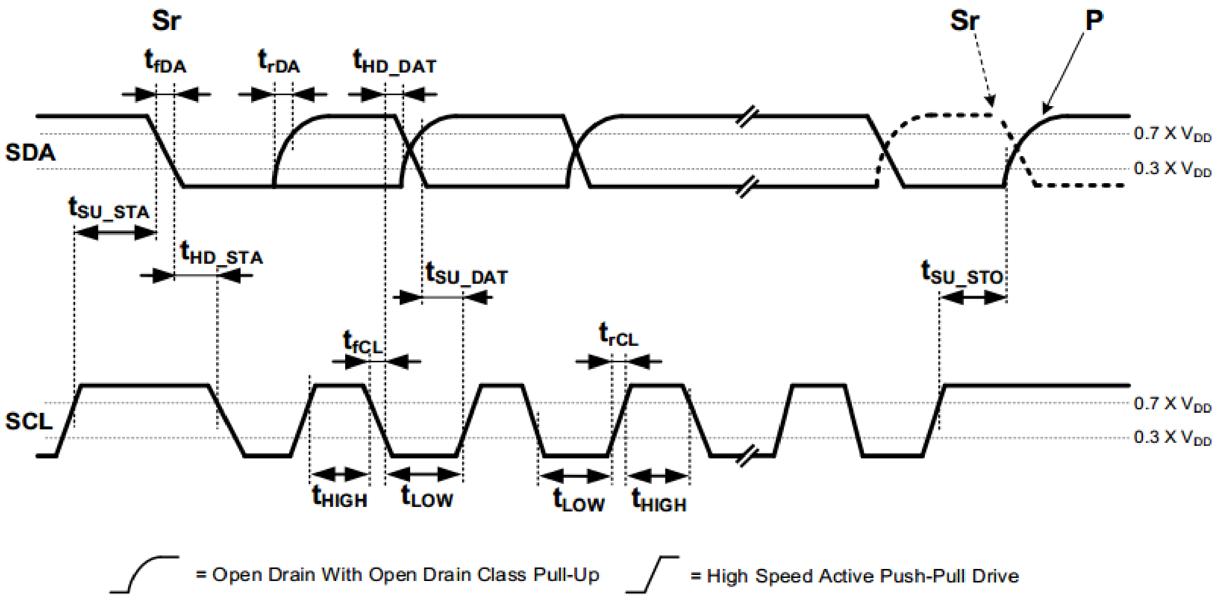

I3C Legacy Mode Timing

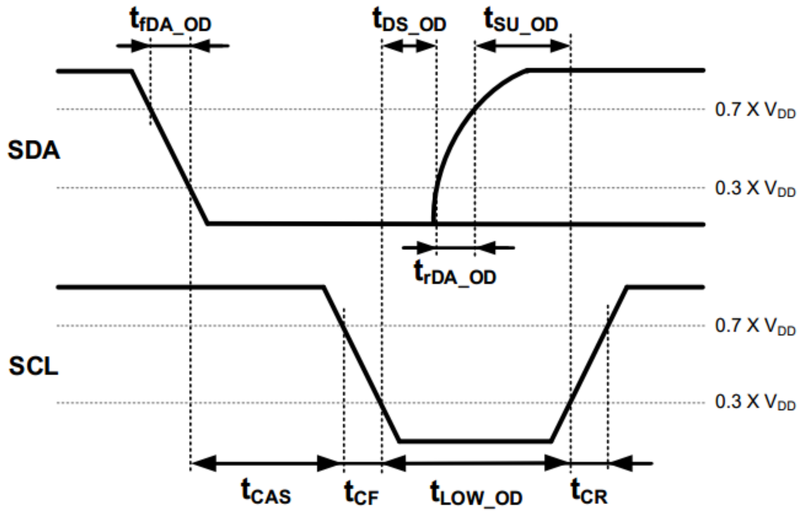

I3C START Timing

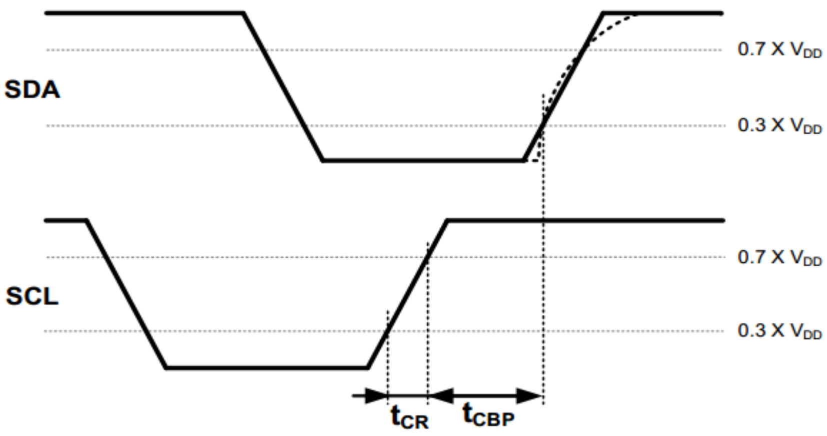

I3C STOP Timing

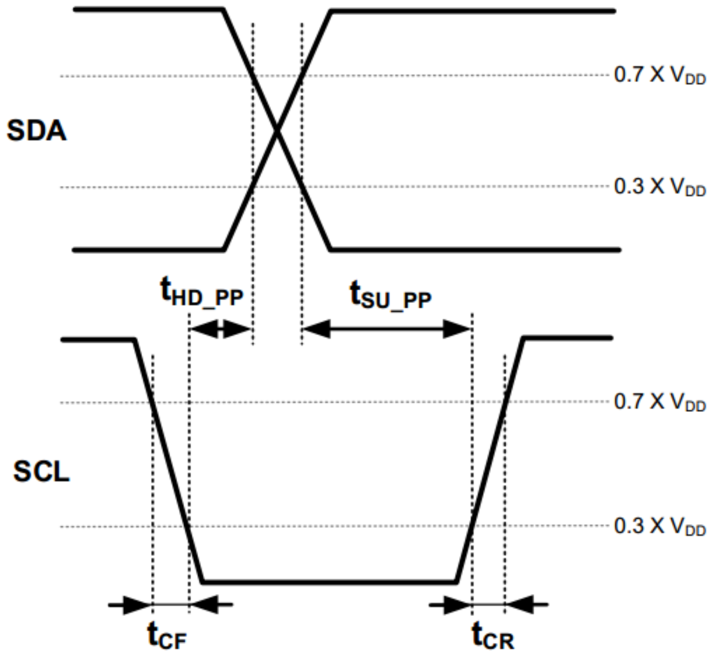

I3C Master Out Timing

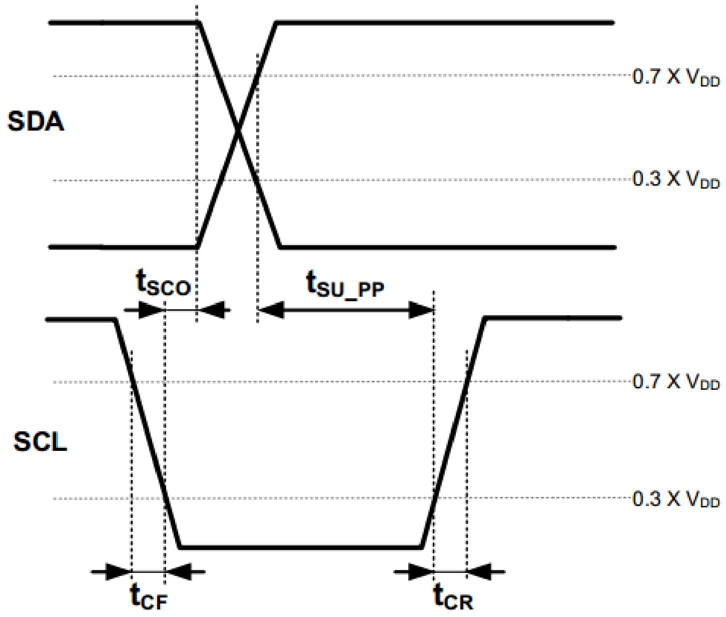

I3C Slave SDR Out Timing

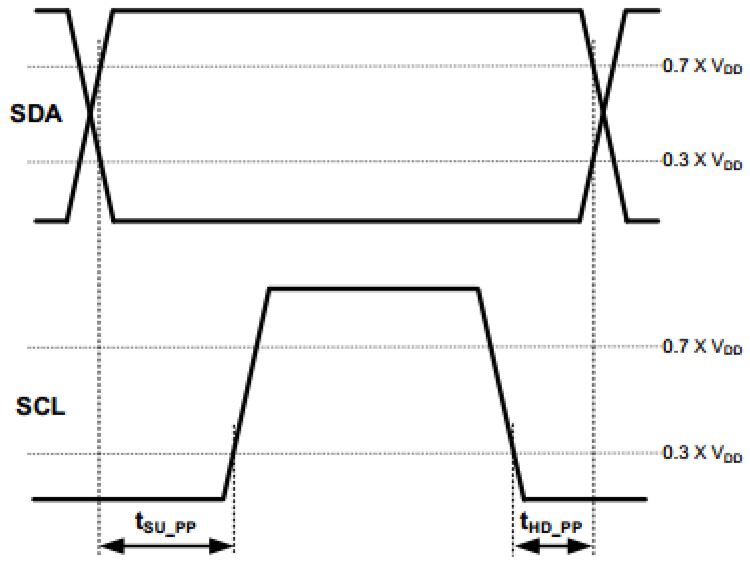

I3C Master SDR Timing

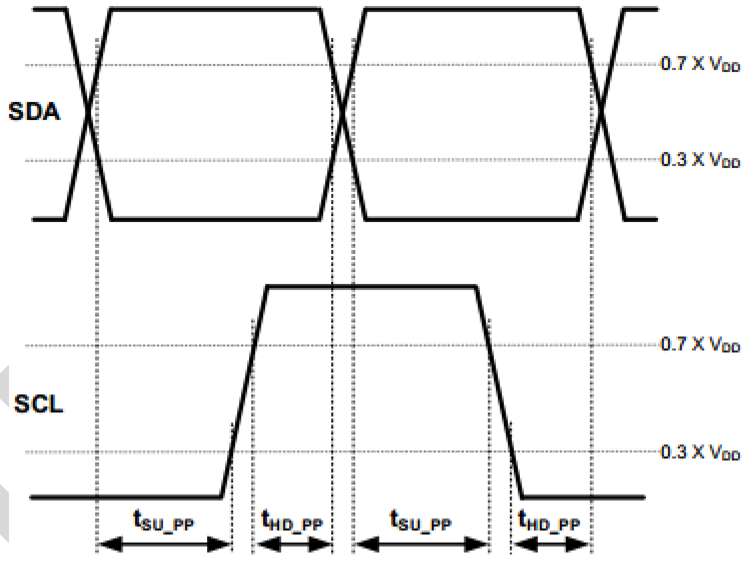

I3C Master HDR-DDR Timing

ABSOLUTE MAXIMUM RATINGS*

Supply Voltage	-0.5 to +5 V
Storage Temperature	-55 °C to +125 °C
Maximum Exposed Field	10000 G

Note: Stresses above those listed under Absolute Maximum Ratings may cause permanent damage to the device. This is a stress rating only; the functional operation of the device at these or any other conditions above those indicated in the operational sections of this specification is not implied. Exposure to absolute maximum rating conditions for extended periods may affect the device's reliability.

Pin Description: WLP Package

Pin	Name	Description	I/O
A1	VSA	Connect to Ground	P
A2	SCL	Serial Clock Line	I
B1	VDD	Power Supply	P
B2	SDA	Serial Data Line	I/O

All parts are shipped in tape and reel packaging with 10000pcs (or 5000pcs per requested) per 7" reel.

Caution:

This is an Electro-static Discharge (ESD) sensitive device.

Ordering Guide:

MMC5633NJL

Package type:

Code	Type
J	WLP package RoHS compliant

Performance Grade:

Code	Performance Grade
N	Temp compensated

MARKING ILLUSTRATION

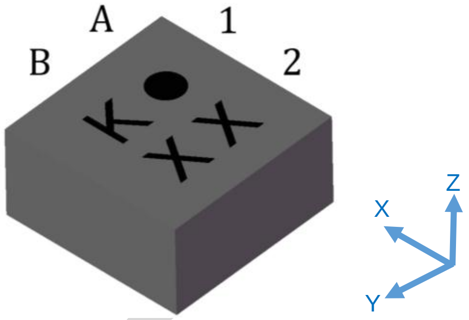

Note: "Number" (top-left character) is used to differentiate between similar devices. The black dot marks the location of pin one (1). The 2nd line represents the device's Lot Number.

THEORY OF OPERATION

The Anisotropic Magneto-Resistive (AMR) sensors are special resistors made of permalloy thin film deposited on a silicon wafer. During manufacturing, a strong magnetic field is applied to the film to orient its magnetic domains in the same direction, establishing a magnetization vector. Subsequently, an external magnetic field applied perpendicularly to the sides of the film causes the magnetization to rotate and change angle. This effect causes the film's resistance to vary with the intensity of the applied magnetic field. The MEMSIC AMR sensor is incorporated into a Wheatstone bridge configuration to maximize Signal to Noise ratio. A change in magnetic field produces a proportional change in differential voltage across the Wheatstone bridge

However, the influence of a strong magnetic field (more than 30 G) in any direction could upset, or flip, the polarity of the film, thus changing the sensor characteristics. A strong restoring magnetic field must be applied momentarily to restore, or set, the sensor characteristics. The MEMSIC magnetic sensor has an on-chip magnetically coupled strap: a SET/RESET strap pulsed with a high current, to provide the restoring magnetic field.

MEMSIC

EXTERNAL CIRCUITRY CONNECTION

The MMC5633NJL can operate from a single 1.62V to 3.6V supply. The circuit connection diagrams below illustrate power supply connection options.

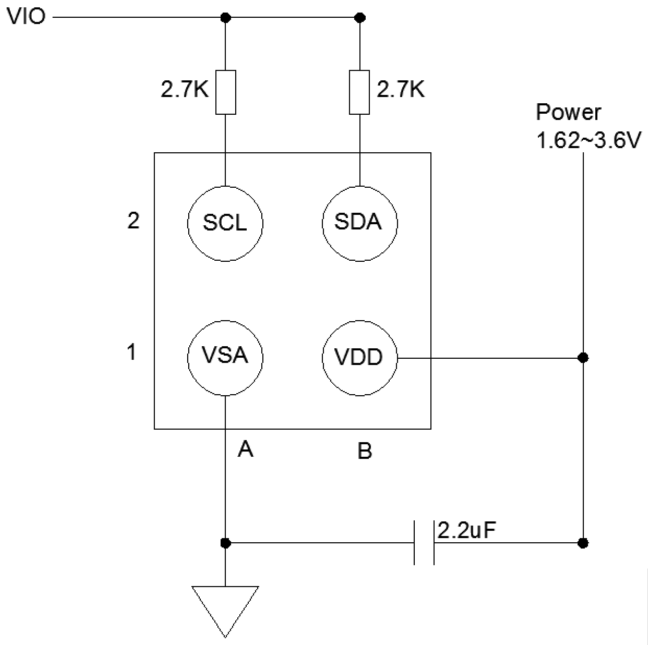

<Top View>
Connection Block Diagram

PIN DESCRIPTIONS

VDD – This is the power supply pin. MEMSIC recommends a minimum bypass capacitor of 2.2 µF placed in close proximity to the VDD pin.

VSA – This is the ground pin for the magnetic sensor.

SDA – This pin is the I3C/I^{2}C serial data line.

SCL– This pin is the I3C/I^{2}C serial clock line.

HARDWARE DESIGN CONSIDERATION

- ✓ Provide adequate separation distance to devices that contain permanent magnets or generate magnetic fields (e.g. speakers, coils, inductors) The combined magnetic field to be measured and interference magnetic field should be less than the full scale range of the MMC5633NJL (±30 G).
- ✓ Provide adequate separation distance to current carrying traces. Do not route current carrying traces under the sensor or on the other side of the PCB opposite the device.
- ✓ Do not cover the sensor with magnetized material or material that may become magnetized, (e.g., shield box, LCD, battery, iron bearing material).
- ✓ Do not place the device opposite magnetized material or material that may become magnetized located on the other side of the PCB.

Details please refer to MEMSIC Magnetic Sensor Hardware Design Layout Guideline for Electronic Device.

POWER CONSUMPTION

The power consumed by the device is proportional to the number of measurements taken per second. For example, when BW<1:0>=10, MMC5633NJL consumes 1.3mA (typical) at 1.8V with 100 measurements per second. If only 1 measurements are performed per second, the current will be 1300*1/100=13µA.

I^{2}C INTERFACE DESCRIPTION

A slave mode I^{2}C circuit has been implemented into the MEMSIC magnetic sensor as a standard interface for customer applications. The A/D converter functionality have been added to the MEMSIC sensor, thereby increasing ease-of-use, and lowering power consumption, footprint and total solution cost.

The I^{2}C (or Inter IC bus) is an industry standard bi-directional two-wire interface bus. A master I^{2}C device can operate READ/WRITE controls to 128 devices by device addressing. The MEMSIC magnetic sensor operates only in a slave mode, i.e. only responding to calls by a master device.

I^{2}C BUS CHARACTERISTICS

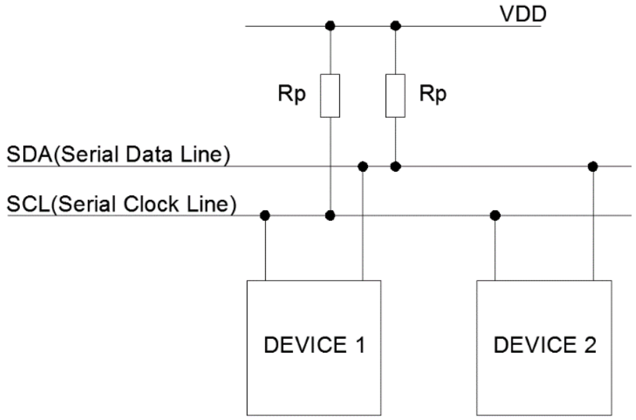

The two wires in the I^{2}C bus are called SDA (serial data line) and SCL (serial clock line). In order for a data transfer to start, the bus has to be free, which is defined by both wires in a HIGH output state. Due to the open-drain/pull-up resistor structure and wired Boolean "AND" operation, any device on the bus can pull lines low and overwrite a HIGH signal. The data on the SDA line has to be stable during the HIGH period of the SCL line. In other words, valid data can only change when the SCL line is LOW.

Note: Rp selection guide: 2.7Kohm for a short I^{2}C bus length (less than 10 cm), and 10Kohm for a bus length less than 5 cm.

MEMSIC

I3C INTERFACE DESCRIPTION

I3C is a two-wire bidirectional serial Bus developed by MIPI Alliance (www.MIPI.org). In order to receive detailed description of I3C interface please contact MIPI Alliance directly.

I3C interface is backward compatible with I^2C, but offers additional features, such faster clock rate, in-band interrupts, and event timing control.

I3C defines several roles for devices connected to the I3C bus. MMC5633NJL is an I3C Slave. It does not have Master capabilities.

MMC5633NJL supports SDR and HDR-DDR modes with clock speed up to 12.5MHz. It does not support High Data Rate Ternary modes.

MMC5633NJL supports in-band interrupts (IBI)

MMC5633NJL does not support Hot Join mechanism.

I3C Spec defines two special 8-bit registers BCR and DCR. MMC5633NJL has BCR= 0x27 and DCR=0x00.

I3C interface defines Common Command Codes (CCC), which can be Broadcast or Direct. MMC5633NJL supports the following CCC's, both Broadcast and Direct:

- Enable Events Command (ENEC)
- Disable Events Command (DISEC)
- Reset Dynamic Address Assignment (RSTDAA)
- Enter Dynamic Address Assignment (ENTDAA)
- Enter HDR Mode 0 (ENTHDR0)
- Exchange Timing Information (SETXTIME)
- Get Exchange Timing Information (GETXTIME)
- Set Dynamic Address from Static Address (SETDASA)
- Set New Dynamic Address (SETNEWDA)
- Get Provisional ID (GETPID)
- Get Bus Characteristics Register (GETBCR)
- Get Device Characteristics Register (GETDCR)
- Get Device Status (GETSTATUS)
- Get Max Data Speed (GETMXDS).
- Get HDR Capability (GETHDRCAP)

INITIATING I3C COMMUNICATION AND DYNAMIC ADDRESS ASSIGNMENT.

MMC5633NJL defaults to I2C mode of communication when power is first applied. Once it detects a READ or WRITE command to address 0x7E MMC5633NJL will switch to I3C mode. It will remain in I3C mode until power is removed.

Since MMC5633NJL has Static I2C address 0x30 (unless ordered in a different configuration), it is recommended to use SETDASA CCC to assign Dynamic Address. MMC5633NJL also supports Minimal Bus Point-To-Point Communication, which is a special case of SETDASA CCC.

The I3C Master may use ENTDAA CCC to assign Dynamic Address. The 48-bit Provisional ID is 0x04_A2_00_00_F0_00.

SDR COMMUNICATION

Once MMC5633NJL is assigned a Dynamic Address, it is ready for SDR communication. The Master can read and write to various registers. List of registers and their definitions are provided later in this document.

Also at this point MMC5633NJL will respond to supported CCC's.

MEMSIC

DEVICE STATUS

I3C defines a 2-byte Status word. The first byte is Status1 register 0x18, and the second byte is the Status0 register 0x19. These registers are defined below.

GETHDRCAP CCC

MMC5633NJL supports HDR-DDR mode. It will return 0x01 in response to GETHDRCAP CCC.

GETMXDS CCC

MMC5633NJL uses Format 1, returning 2-byte. The first byte defines the maximum write speed and is equal to 0x00. The second byte defines the maximum read speed and is set to 0x20. Bits[5:3] define clock-to-data turn-around time Tsco.

IN-BAND INTERRUPT (IBI)

If there is a pending interrupt MMC5633NJL will initiate an IBI request. There is a Mandatory Data Byte which the Master must read if the IBI was accepted. The Mandatory Data Byte is the same as Status1 register 0x18 defined below.

SETXTIME CCC.

MMC5633NJL supports the synchronous timing control as described in the section of SETXTIME of the I3C specification V1.0. This technique provides an approach to synchronize the activities of sensors on I3C bus. In order for the timing control to work properly, five messages are necessary, which include output data rate (ODR), time period and phase (TPH), time unit (TU), synchronization tick (ST) and delay time (DT). ODR, TPH and TU can be defined by either writing to corresponding registers, or through SETXTIME CCC command followed by defining bytes. ST and DT are delivered by SETXTIME CCC command followed by defining bytes. If SETXTIME CCC commands are preferred, users should refer to the following table for the values of the defining bytes.

**Defining byte value**	**Sub-Command Function**	**Description**	**Additional Data Bytes**
0x7F	Sync Tick "ST"	Marks the START condition, which is used as the reference time for the synchronization procedure.	None
0xBF	Delay Time "DT"	Transfers the Delay Time value	One byte: Delay from correct TPH Start to ST marked START.
0x3F	TPH	Specifies the repetition period of synchronization event.	One byte or two bytes
0x9F	TU	Specifies the Time Unit used for DT command.	One byte.
0x8F	ODR	Specifies the output data rate of the sensor.	One byte.

The registers for ODR, TPH and TU will be provided in later sections as well, in case users prefer writing to registers to define such parameters.

MEMSIC

GETXTIME CCC

MMC5633NJL supports the GETXTIME CCC that is defined by the specification of I3C, Version1.0. It provides four data bytes that contain information of supported modes, state, frequency, and inaccuracy information.

HDR-DDR MODE.

MMC5633NJL supports HDR-DDR mode. It is invoked with ENTHDR0 CCC. It does not support other HDR modes. If the Master issues any of the ENTHDR2 – ENTHDR7 CCC's, MMC5633NJL will tristate its SDA output and wait for the HDR-EXIT pattern.

Once MMC5633NJL received an ENTHDR0 CCC it will switch to HDR-DDR mode. It will remain in this mode until it detects HDR-EXIT pattern.

HDR-DDR WRITE commands.

Basic structure of an HDR-DDR command is as following:
2 preamble bits P1 and P0
16 bits of data called Payload.
2 parity bits, also called P1 and P0
The whole frame is 20 bits long.

The MSB of the Payload is the R/W bit. It should be 0 for the Write commands.
The next 7 bits of the Payload are the Command. MMC5633NJL only allows a single Write command value 7'h3B.
The next 7 bits of the Payload are the Dynamic Address
The last (LSB) bit of the Payload is calculated to make parity bit P0 = 1.

A Data Word follows the Command word. Its structure is:
2 preamble bits P1, P0
2 data bytes
2 Parity bits P1 and P0.

The two data bytes of the Data Word define how MMC5633NJL is going to respond. The following six Write commands are defined:
Data Word = 16'h0001 -> Take Measure Magnetic. This has the same effect as writing bit Take_meas_M high.
Data Word = 16'h0003 -> Take Measure Temperature. This has the same effect as writing bit Take_meas_T high.
Data Word = 16'h0004 -> Turn On Continuous Mode. This has the same effect as writing bit Cmm_en high.
Data Word = 16'h0005 -> Do Set. This has the same effect as writing bit "Do Set" high.
Data Word = 16'h0006 -> Turn Off Continuous Mode This has the same effect as writing bit Cmm_en low.
Data Word = 16'h0007 -> Do Reset. This has the same effect as writing bit "Do Reset" high.

A CRC word must follow the Data Word as defined in the MIPI I3C Specification.

HDR-DDR READ commands.

In case of READ command from the Master, the format is a little different. There is no Data Word. The first frame following the HDR Restart has a preamble of 01 and then a Command. The Command has MSB as R/W bit set high, 7-bit Dynamic Address, and a 7-bit Command Code. The last bit of this 16-bit word is picked to make parity bit P0 = 1.

MEMSIC

MMC5633NJL allows the following Command Codes:

7’h21 -> RDSHORT
7’h22 -> RDLONG
7’h23 -> RDSTAT

If MMC5633NJL receives RDSHORT command it will return 3 frames (6 bytes) of data. This data is 16-bit values of X, Y, and Z magnetic field. The first frame payload consists of registers 0x00 followed by register 0x01. The second frame payload consists of registers 0x02 and 0x03. The third frame payload consists of registers 0x04 and 0x05.

If MMC5633NJL receives RDLONG command it will return 5 frames (10 bytes) of data. This allows the user to read 20-bit magnetic field values as well as 8-bit temperature value. The first frame payload consists of registers 0x00 followed by register 0x01. The second frame outputs registers 0x02 and 0x03. The third one registers 0x04 and 0x05. The fourth one outputs registers 0x06 and 0x07, and the fifth one registers 0x08 and 0x09.

If MMC5633NJL receives a RDSTAT command, it will output a single frame (16 bits). The output data consists of Status registers 0x18 and 0x19.

These Data Words are followed by a CRC word as defined in the MIPI I3C Spec.

REGISTER MAP

Register Name	Address (HEX)	Description
Xout0	00H	Xout[19:12]
Xout1	01H	Xout[11:4]
Yout0	02H	Yout[19:12]
Yout1	03H	Yout[11:4]
Zout0	04H	Zout[19:12]
Zout1	05H	Zout[11:4]
Xout2	06H	Xout[3:0]
Yout2	07H	Yout[3:0]
Zout2	08H	Zout[3:0]
Tout	09H	Temperature output
TPH0	0AH	TPH[7:1]
TPH1	0BH	6’h00,TPH[9:8]
TU	0CH	TU[1:0]
Status1	18H	Device status1
Status0	19H	Device status0
ODR	1AH	Output Data Rate
Internal control 0	1BH	Control register 0
Internal control 1	1CH	Control register 1
Internal control 2	1DH	Control register 2
ST_X_TH	1EH	X-axis selftest threshold
ST_Y_TH	1FH	Y-axis selftest threshold
ST_Z_TH	20H	Z-axis selftest threshold
ST_X	27H	X-axis selftest set value
ST_Y	28H	Y-axis selftest set value
ST_Z	29H	Z-axis selftest set value
Product ID	39H	Product ID

MEMSIC

REGISTER DETAILS

Xout0, Xout1, Xout2

**Xout0**	7	6	5	4	3	2	1	0
Addr: 00H	Xout[19:12]							
Mode	Read-only							

**Xout1**	7	6	5	4	3	2	1	0
Addr: 01H	Xout[11:4]							
Mode	Read-only							

**Xout2**	7	6	5	4	3	2	1	0
Addr: 06H	Xout[3:0]				0	0	0	0
Mode	Read-only							

X-axis output, unsigned format.

X-axis output	Data
16bits operation mode	Xout[19:4]
18bits operation mode	Xout[19:2]
20bits operation mode	Xout[19:0]

Yout0, Yout1, Yout2

**Yout0**	7	6	5	4	3	2	1	0
Addr: 02H	Yout[19:12]							
Mode	Read-only							

**Yout1**	7	6	5	4	3	2	1	0
Addr: 03H	Yout[11:4]							
Mode	Read-only							

**Yout2**	7	6	5	4	3	2	1	0
Addr: 07H	Yout[3:0]				0	0	0	0
Mode	Read-only							

Y-axis output, unsigned format.

Y-axis output	Data
16bits operation mode	Yout[19:4]
18bits operation mode	Yout[19:2]
20bits operation mode	Yout[19:0]

Zout0, Zout1, Zout2

**Zout0**	7	6	5	4	3	2	1	0
Addr: 04H	Zout[19:12]							
Mode	Read-only							

**Zout1**	7	6	5	4	3	2	1	0
Addr: 05H	Zout[11:4]							
Mode	Read-only							

**Zout2**	7	6	5	4	3	2	1	0
	Zout[3:0]				0	0	0	0
Addr: 08H								
Mode	Read-only							

MEMSIC

Z-axis output, unsigned format.

Z-axis output	Data
16bits operation mode	Zout[19:4]
18bits operation mode	Zout[19:2]
20bits operation mode	Zout[19:0]

Temperature Out

**Temperature**	7	6	5	4	3	2	1	0
Addr: 09H	Tout[7:0]							
Mode	Read-only							

Temperature output, unsigned format. The range is -75-125°C, about 0.8°C/LSB, 00000000 stands for -75°C

TPH0

**TPH0**	7	6	5	4	3	2	1	0
Addr: 0AH	TPH_Format	TPH[7:1]						
Mode	W							

This byte, together with the next register, TPH1, defines the parameter called TPH for the synchronizations of measurements according to I3C protocol. TPH is the time between two adjacent synchronization events. The master should send a synchronization tick (ST) to slaves, with a period of TPH, as a reference for the internal circuits of the sensor to adjust the period and phase of its measurements. The MSB of this byte defines whether the TPH contains 1 byte (TPH_Format=0), or 2 bytes (TPH_Format=1). If TPH has only 1 byte, its value is defined as {TPH[7:1], 1'b0}, and if TPH has 2 bytes, its value is defined as {TPH[9:1], 1'b0}, where TPH[9:8] comes from the next register, TPH1. The unit of TPH is the number of measurements. For example, TPH=100 means 100 measurements, and the time interval of two adjacent synchronization will be 1 second in case of 100Hz ODR. Alternatively, TPH can be defined by SETXTIME CCC command, with the defining byte of 0x3F, followed by the TPH value. Only one data byte is allowed if TPH is defined by SETXTIME CCC, and the value of that byte is the same as this register.

TPH1

**TPH1**	7	6	5	4	3	2	1	0
Addr: 0BH	0	0	0	0	0	0	TPH[9:8]	
Mode	W							

This byte defines the higher portion of the TPH, and is valid only when TPH_Format=1. MMC5633NJL supports up to 10 bits of TPH, so that only TPH[9:8] are used to define TPH, and the rest of this byte should be set to 0.

TU

**TU**	7	6	5	4	3	2	1	0
Addr: 0CH	x	x	x	x	x	x	TU[1:0]	
Mode	W							

This byte is used to define the time unit of the synchronization events. Only 2 bits are used, and the corresponding time units are 2^(-11), 2^(-12), 2^(-13), and 2^(-14) of the TPH, respectively, for the TU value of 00, 01, 10, and 11. Alternatively, this byte can be defined by I3C CCC command, with the defining byte of 0x9F, followed by one byte of data.

Status1

**Device Status1**	7	6	5	4	3	2	1	0
Addr: 18H	Meas_t_done	Meas_m_done	Sat_sensor	OTP_read_done	ST_Fail	Mdt_flag_int	Meas_t_done_int	Meas_m_done_int
Reset Value	0	0	0	0	0	0	0	0
Mode	Read-only							

**Bit Name**	**Description**
Meas_m_done_int	This bit indicates whether Take Measurement of Magnetic field command has been completed and data ready to be read by the host. This bit is primarily for the I3C in-band interrupt (IBI). This bit is reset if a new Take Measurement command is received or if any of the magnetic data registers is access through I^2C. However, this bit is also reset if either the I3C In-Band Interrupt (IBI) is sent, or this bit is read.

MEMSIC

Meas_t_done_int	This bit indicates whether Take Measurement of Temperature command has been completed and data ready to be read by the host. This bit is also for I3C IBI, it is reset if a new Take Measurement command is received or if the temperature data register is accessed through I^2C. However, this bit is also reset if either the IBI interrupt is sent, or this bit is read.
Mdt_flag_int	This bit indicates whether a motion is detected by the motion detector. This bit is set High when a motion is detected, and it is reset when Start_MDT is received, or an IBI is sent to the master, or this bit is read.
ST_Fail	This bit is specifically for I3C IBI. When the sensor receives the ST and/or DT command via I3C interface, and parity bit is incorrect, this bit will be set High and the sensor will send an IBI to inform the master. This bit is reset after the IBI is sent.
OTP_read_done	This bit is an indicator of successfully reading its OTP memory either as part of its power up sequence, or after an I2C command that reloads the OTP memory, such as resetting the chip and refreshing the OTP registers.
Sat_sensor	This bit is an indicator of the selftest signal, it keeps low once the device PASS selftest.
Meas_m_done	This bit indicates that a measurement of magnetic field is done and the data is ready to be read. This bit is reset only when any of the magnetic data registers is read.
Meas_t_done	This bit indicates that a measurement of temperature is done and the data is ready to be read. This bit is reset only when the temperature register is read.

Status0

**Device Status0**	7	6	5	4	3	2	1	0
Addr: 19H	Activity Mode[1:0]		Protocol error	0	0	Mdt_flag	Pending Interrupt Number[1:0]	
Reset Value	0	0	0	0	0	0	0	0
Mode	R							

**Bit Name**	**Description**
Pending Interrupt Number[1:0]	0b00 – no interrupts pending.<br>0b01 – either of the Meas_m_done_int or Meas_t_done_int is high<br>0b10 – Mdt_flag_int bit is high<br>0b11 – protocol error is detected.
Mdt_flag	This bit becomes high when motion is detected. This bit is cleared when a new Start_MDT bit is written t 1. FACTORY USE ONLY.
Protocol error	I3C Protocol error is detected.
Activity Mode[0]	Always 0
Activity Mode[1]	0 – MMC5633NJL is in power down mode.<br>1 – MMC5633NJL is actively performing a measurement

ODR

**ODR**	7	6	5	4	3	2	1	0
Addr: 1AH	ODR[7:0]							
Reset Value	0	0	0	0	0	0	0	0
Mode	Write-only							

<table>
<tr><th>Bit Name</th><th>Description</th></tr>
<tr><td>ODR[7:0]</td><td>This byte defines the frequency of the continuous-mode measurements. ODR can be defined by either writing the number into this register, or using SETXTIME CCC command, followed by defining byte of 0x8F. In order to enter the continuous mode, this byte should not be zero. The configurable ODR is 1 to 255, with increment of 1. The 1000 Hz ODR is available by writing 255 into this byte and setting hpower to 1. The maximum reachable ODR as below:
<table>
<tr><td>BW</td><td>Automatic SET/RESET</td><td>No SET/RESET</td><td>ODR range</td></tr>
<tr><td>00</td><td>75Hz</td><td>150 Hz</td><td>1~75</td></tr>
<tr><td>01</td><td>150 Hz</td><td>255 Hz</td><td>1~150</td></tr>
<tr><td>10</td><td>255 Hz</td><td>255 Hz</td><td>1~255</td></tr>
<tr><td>11</td><td>255Hz</td><td>hpower=0: 255 Hz;
hpower=1: 1000 Hz</td><td>1~255</td></tr>
</table>
</td></tr>
</table>

Internal Control 0

Control Register 0	7	6	5	4	3	2	1	0
Addr: 1BH	Cmm_freq_en	Auto_st_en	Auto_SR_en	Do Reset	Do Set	Start_MDT	Take_meas_T	Take_meas_M
Reset Value	0	0	0	0	0	0	0	0
Mode	Write-only							

Bit Name	Description
Take_meas_M	Take Measure of Magnetic field, or TM_M bit. Writing a 1 into this location causes the chip to perform a magnetic measurement. This bit is self-clearing at the end of each measurement.
Take_meas_T	Take Measure of Temperature, or TM_T bit. Writing a 1 into this location causes the chip to perform a temperature measurement. This bit is self-clearing at the end of each measurement.
Start_MDT	Factory use only, reset value is 0.
Do Set	Writing a 1 into this location will cause the chip to do the Set operation, which will allow large set current to flow through the sensor coils for 375ns. This bit is self-cleared at the end of Set operation.
Do Reset	Writing a 1 into this location will cause the chip to do the Reset operation, which will allow large reset current to flow through the sensor coils for 375ns. This bit is self-cleared at the end of Reset operation.
Auto_SR_en	Writing a 1 into this location will enable the function of automatic set/reset. This function applies to both on-demand and continuous-time measurements. This bit must be set to 1 in order to activate the feature of periodic set. This bit is recommended to set to "1" in the application.
Auto_st_en	Writing a 1 into this location will enable the function of automatic self-test. The threshold in register 1EH, 1FH, 20H should be set before this bit is set to 1. This bit clears itself after the operation is completed.
Cmm_freq_en	Writing a 1 into this location will start the calculation of the measurement period according to the ODR. This bit should be set before continuous-mode measurements are started. This bit is self-cleared after the measurement period is calculated by internal circuits.

Internal Control 1

Control Register 1	7	6	5	4	3	2	1	0
Addr: 1CH	Sw_reset	St_enm	St_enp	Z-inhibit	Y-inhibit	X-inhibit	BW1	BW0
Reset Value	0	0	0	0	0	0	0	0
Mode	Write-only							

<table>
<tr><th>Bit Name</th><th>Description</th></tr>
<tr><td>BW0 & BW1</td><td>These bandwidth selection bits adjust the length of the decimation filter. They control the duration of each measurement.
<table>
<tr><td>BW1</td><td>BW0</td><td>Measurement Time</td></tr>
<tr><td>0</td><td>0</td><td>6.6ms</td></tr>
<tr><td>0</td><td>1</td><td>3.5ms</td></tr>
<tr><td>1</td><td>0</td><td>2.0ms</td></tr>
<tr><td>1</td><td>1</td><td>1.2ms</td></tr>
</table>
Note: X/Y/Z channel measurements are taken sequentially. Delay Time among those measurements is 1/3 of the Measurement Time defined in the table.</td></tr>
<tr><td>X-inhibit</td><td rowspan="3">Writing "1" will disable this channel, and reduce Measurement Time and total charge per measurement.When a channel is disabled it is simply skipped during Take Measure routine. Its output register is not reset and will maintain the last value written to it when this channel was active. Note: Y/Z needs to be inhibited the same time in case needed.</td></tr>
<tr><td>Y-inhibit</td></tr>
<tr><td>Z-inhibit</td></tr>
<tr><td>St_enp</td><td>Writing 1 into this location will bring a DC current through the self-test coil of the sensor. This current will cause an offset of the magnetic field. This function is used to check whether the sensor has been saturated.</td></tr>
</table>

MEMSIC

St_enm	The function of this bit is similar to ST_ENP, but the offset of the magnetic field is of opposite polarity.
Sw_reset	Software Reset. Writing “1”will cause the part to reset, similar to power-up. It will clear all registers and also re-read OTP as part of its startup routine. The power on time is 20mS.

Internal Control 2

**Control Register 2**	7	6	5	4	3	2	1	0
Addr: 1DH	hpower	INT_meas_done_en	INT_mdt_en	Cmm_en	En_prd_set	Prd_set[2:0]		
Reset Value	0	0	0	0	0	0	0	0
Mode	Write-only							

**Bit Name**	Description
Prd_set[2:0]	These bits determine how many measurements are done before a set is executed, when the part is in continuous mode and the automatic set/reset is enabled. From 000 to 111, the sensor will do one set for every 1, 25, 75, 100, 250, 500, 1000, and 2000 samples. In order to enable this feature, both En_prd_set and Auto_SR must be set to 1, and the part should work in continuous mode. Please note that during this operation, the sensor will not be reset.
En_prd_set	Writing 1 into this location will enable the function of periodical set.
Cmm_en	The device will enter continuous mode, if ODR has been set to a non-zero value and a 1 has been written into Cmm_freq_en. The internal counter will start counting as well since this bit is set.
INT_mdt_en	Factory use only, reset value is 0.
INT_meas_done_en	Factory use only, reset value is 0.
hpower	If this bit is set to 1 to achieve 1000Hz ODR.

ST_X_TH

ST_X_TH	7	6	5	4	3	2	1	0
Addr: 1EH	ST_X_TH[7:0]							
Reset Value	0	0	0	0	0	0	0	0
Mode	Write-only							

X-axis selftest threshold

ST_Y_TH

ST_Y_TH	7	6	5	4	3	2	1	0
Addr: 1FH	ST_Y_TH[7:0]							
Reset Value	0	0	0	0	0	0	0	0
Mode	Write-only							

Y-axis selftest threshold

ST_Z_TH

ST_Z_TH	7	6	5	4	3	2	1	0
Addr: 20H	ST_Z_TH[7:0]							
Reset Value	0	0	0	0	0	0	0	0
Mode	Write-only							

Z-axis selftest threshold

ST_X

ST_X	7	6	5	4	3	2	1	0
Addr: 27H	ST_X[7:0]							
Reset Value	Factory stored value							
Mode	Read/Write							

X-axis selftest set value

MEMSIC

ST_Y

ST_Y	7	6	5	4	3	2	1	0
Addr: 28H	ST_Y[7:0]							
Reset Value	Factory stored value							
Mode	Read/Write							

Y-axis selftest set value

ST_Z

ST_Z	7	6	5	4	3	2	1	0
Addr: 29H	ST_Z[7:0]							
Reset Value	Factory stored value							
Mode	Read/Write							

Z-axis selftest set value

Product ID 1

Product ID 1	7	6	5	4	3	2	1	0
Addr: 39H	Product ID1[7:0]							
Reset Value	0	0	0	1	0	0	0	0
Mode	Read-only							

Product ID, used to recognize device.

MEMSIC

I^{2}C Interface operation:

DATA TRANSFER

A data transfer is started with a "START" condition and ended with a "STOP" condition. A "START" condition is defined by a HIGH to LOW transition on the SDA line while SCL line is HIGH. A "STOP" condition is defined by a LOW to HIGH transition on the SDA line while the SCL line is held HIGH. All data transfer in I^{2}C system are 8-bits long. Each byte has to be followed by an acknowledge bit. Each data transfer involves a total of 9 clock cycles. Data is transferred starting with the most significant bit (MSB).

After a START condition, the master device calls a specific slave device by sending its 7-bit address with the 8th bit (LSB) indicating that either a READ or WRITE operation will follow, [1] for READ and [0] for WRITE. The MEMSIC device 7-bit device address is [**0110000]** where the three LSB's are pre-programmed into the MMC5633NJL by the factory.

Note: A total of 8 different addresses can be pre-programmed into MEMSIC device by the factory. This variation of I^{2}C address avoids a potential address conflict, either by ICs from other manufacturers or by other MEMSIC devices on the same bus.

The initial addressing of the slave is always followed by the master writing the number of the slave register to be read or written, so this initial addressing always indicates a WRITE operation by sending **[01100000]**. After being addressed, the MEMSIC device being called should respond by an "Acknowledge" signal by pulling SDA line LOW. Subsequent communication bytes can either be

a) The data to be written to the device register, or

b) Another START condition followed by the device address indicating a READ operation **[01100001]**, and then the master reads the register data.

Multiple data bytes can be written or read to numerically sequential registers without the need of another START condition. Data transfer is terminated by a STOP condition or another START condition. Two detailed examples of communicating with the MEMSIC device are listed below for the actions of acquiring a magnetic field measurement and magnetizing the sensor.

EXAMPLE OF MEASUREMENT

1st cycle: A START condition is established by the Master Device followed by a call to the slave address [0110000] with the eighth bit held low to indicate a WRITE request.

2nd cycle: After an acknowledge signal is received by the master device (MEMSIC device pulls SDA line low during 9th SCL pulse), the master device sends the address of Control Register 0 as the target register to be written. The MEMSIC device should acknowledge receipt of the address (9th SCL pulse, SDA pulled low).

3rd cycle: The Master device writes to the Internal Control Register 0 the code [00100001] (TM_M and Auto_SR_en high) to initiate data acquisition. The MEMSIC device should send an Acknowledge and internally initiate a measurement (collect x, y and z data). A STOP condition indicates the end of the write operation.

4th cycle: The Master device sends a START command followed by the MEMSIC device's seven bit address, and finally the eighth bit set low to indicate a WRITE. An Acknowledge should be send by the MEMSIC device in response.

5th cycle: The Master device sends the MEMSIC Device Status Register1 as the address to read.

6th cycle: The Master device sends a START command followed by the MEMSIC device's seven bit address, and finally the eighth bit set high to indicate a READ. An Acknowledge should be send by the MEMSIC device in response.

7th cycle: The Master device cycles the SCL line. This causes the Status Register data to appear on SDA line. Continuously read the Device Status Register1 until the Meas_M_Done bit (bit 1) is set to '1'. This indicates that data for the x, y, and z sensors is available to be read.

8th cycle: The Master device sends a START command followed by the MEMSIC device's seven bit address, and finally the eighth bit set low to indicate a WRITE. An Acknowledge should be send by the MEMSIC device in response.

9th cycle: The Master device sends a [00000000] (Xout LSB register address) as the register address to read.

10th cycle: The Master device calls the MEMSIC device's address with a READ (8th SCL cycle SDA line high). An Acknowledge should be send by the MEMSIC device in response.

11th cycle: Master device continues to cycle the SCL line, and each consecutive byte of data from the X, Y and Z registers should appear on the SDA line. The internal memory address pointer automatically moves to the next byte. The Master device acknowledges each. Thus:

12th cycle: Xout[19:12].

13th cycle: Xout[11:4].

14th cycle: Yout[19:12].

15th cycle: Yout[11:4].

16th cycle: Zout[19:12].

17th cycle: Zout[11:4].

18th cycle: Xout[3:2] for 18bits mode. Xout[3:0] for 20bits mode

MEMSIC

19th cycle: Yout[3:2] for 18bits mode. Yout[3:0] for 20bits mode

20th cycle: Zout[3:2] for 18bits mode. Zout[3:0] for 20bits mode:0].

Master ends communications by NOT sending an 'Acknowledge' and also follows with a 'STOP' command.

EXAMPLE OF CONTINUOUS MODE

The MMC5633NJL is designed with an on-chip continuous mode, or CMM. When enabled, the part will periodically take a measurement and store the results in I^2C register. The frequency of these measurements is controlled by a setting in I^2C register. The results of the last measurement can be read by the host. This mode, while it consumes more current, eliminates the need for the host to request measurements every time.

First the user needs to write the desired number into ODR[7:0]. It should be a non-zero integer, otherwise the continuous mode will not be activated. Then Cmm_freq_en is set to 1 to let the internal circuitry to calculate the target number for the counter. After that Cmm_en is set to 1 and the continuous mode is started and the internal counter starts to count at the same time.

EXAMPLE OF SET

1st cycle: A START condition is established by the Master Device followed by a call to the slave address [0110000] with the eighth bit held low to indicate a WRITE request.

2nd cycle: After an acknowledge signal is received by the master device (The MEMSIC device pulls the SDA line low during the 9th SCL pulse), the master device sends [00011011] as the target address (Internal Control Register 0). The MEMSIC device should acknowledge receipt of the address (9th SCL pulse).

3rd cycle: The Master device writes to the MEMSIC device's Internal Control 0 register the code [00001000] (SET bit) to initiate a SET action. The MEMSIC device should send an Acknowledge.

EXAMPLE OF RESET

1st cycle: A START condition is established by the Master Device followed by a call to the slave address [0110000] with the eighth bit held low to indicate a WRITE request.

2nd cycle: After an acknowledge signal is received by the master device (The MEMSIC device pulls the SDA line low during the 9th SCL pulse), the master device sends [00011011] as the target address (Internal Control Register 0). The MEMSIC device should acknowledge receipt of the address (9th SCL pulse).

3rd cycle: The Master device writes to the MEMSIC device's Internal Control 0 register the code [00010000] (RESET bit) to initiate a RESET action. The MEMSIC device should send an Acknowledge.

At this point, the MEMSIC AMR sensors have been conditioned for optimum performance and data measurements can commence.

Note:

- The RESET action can be skipped for most applications

USING SET AND RESET TO REMOVE BRIDGE OFFSET

The integrated SET and RESET functions of the MMC5633NJL enables the user to remove the error associated with bridge Offset change as a function of temperature, thereby enabling more precise heading measurements over a wider temperature than competitive technologies. The SET and RESET functions effectively alternately flip the magnetic sensing polarity of the sensing elements of the device.

1) The most accurate magnetic field measurements can be obtained by using the protocol described as follows: Perform SET. This sets the internal magnetization of the sensing resistors in the direction of the SET field.

2) Perform MEASUREMENT. This measurement will contain not only the sensors response to the external magnetic field, H, but also the Offset; in other words,

   Output1 = +H + Offset.

3) Perform RESET. This resets the internal magnetization of the sensing resistors in the direction of the RESET field, which is opposite to the SET field (180° opposed).

4) Perform MEASUREMENT. This measurement will contain both the sensors response to the external field and also the Offset. In other words,
   Output2 = -H + Offset.

5) Finally, calculate H by subtracting the two measurements and dividing by 2. This procedure effectively eliminates the Offset from the measurement and therefore any changes in the Offset over temperature.
   H = (Output1-Ouput2)/2.

Note:

- To calculate and store the offset; add the two measurements and divide by 2. This calculated offset value can be subtracted from subsequent measurements to obtain H directly from each measurement.

MEMSIC

EXAMPLE OF SELFTEST

The MMC5633NJL is designed with an on-chip selftest signal to do self-diagnose of the sensor:

1) Read out the selftest signal stored at register 27H, 28H, and 29H.
2) Calculate the selftest signal threshold with 80% of the data readout from above registers.
3) Write the threshold in to the register 1EH, 1FH, and 20H.
4) Write [01000001] (TM_M and auto_st_en high) to Internal Control Register 1BH to initiate a selftest.
5) Read out value of Sat_sensor bit at the Device Status register 18H.
6) Sat_sensor=0, PASS selftest.

MEMSIC

OPERATING TIMING

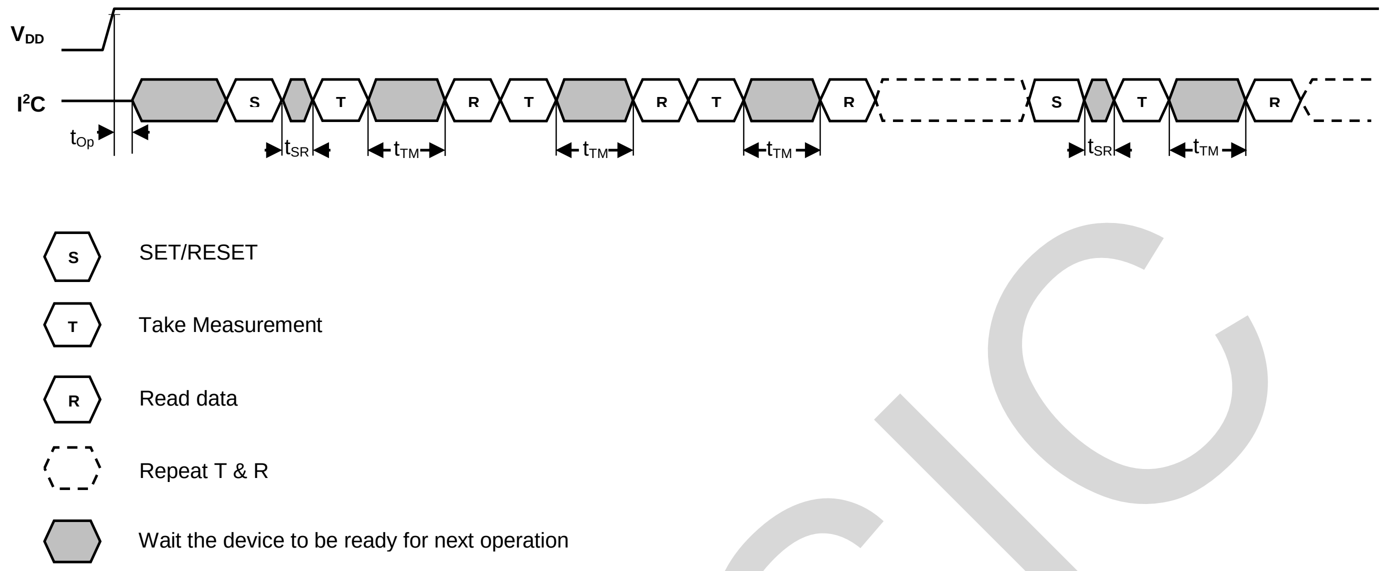

Operating Timing Diagram

Parameter	Symbol	Min.	Max.	Unit
Time to operate device after V_{DD} valid	t_{Op}	5		ms
Minimum time interval between SET or RESET to other operations	t_{SR}	1		ms
	t_{TM} BW=00	6.6		ms
	t_{TM} BW=01	3.5		ms
	t_{TM} BW=10	2.0		ms
	t_{TM} BW=11	1.2		ms

MEMSIC

SOLDERING RECOMMENDATIONS

MEMSIC magnetic sensor is capable of withstanding an MSL1 / 260 ºC solder reflow. Following is the reflow profile:

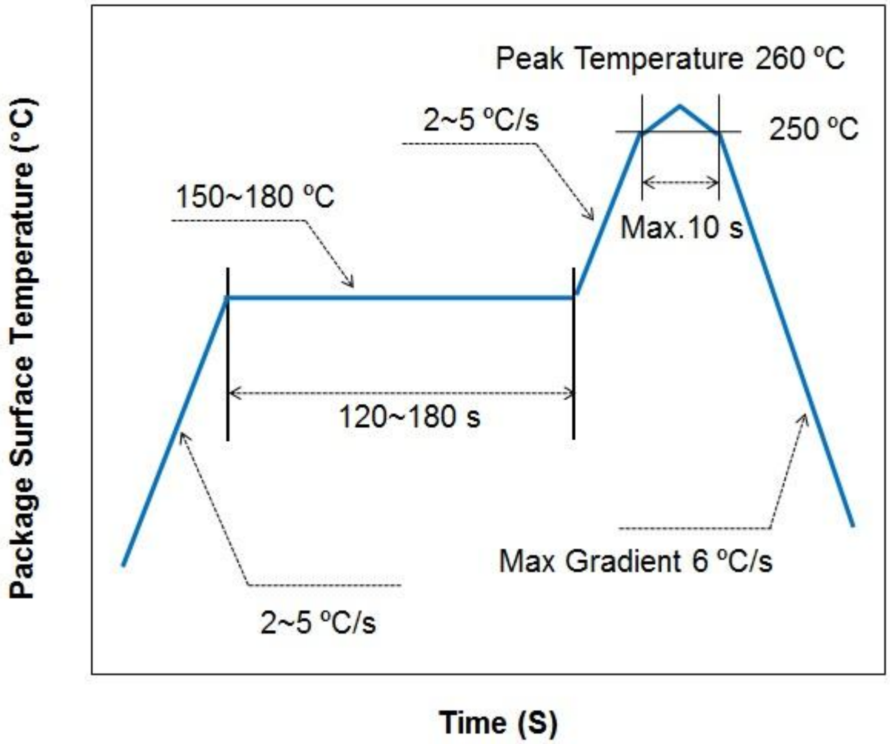

Note:

- The second reflow cycle should be applied after device has cooled down to 25 ℃ (room temperature)
- This is the reflow profile for Pb free process
- The peak temperature on the sensor surface should be limited under 260 ℃ for 10 seconds.
- Solder paste's reflow recommendation should be followed to get the best SMT quality.

If the part is mounted manually, please ensure the temperature could not exceed 260 ℃ for 10 seconds.

PACKAGE DRAWING (WLP package)

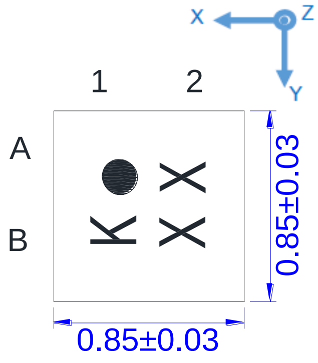

(TOP VIEW)

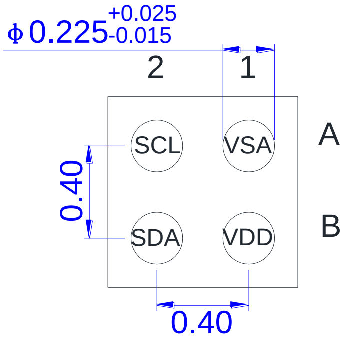

(BOTTOM VIEW)

UNIT: mm

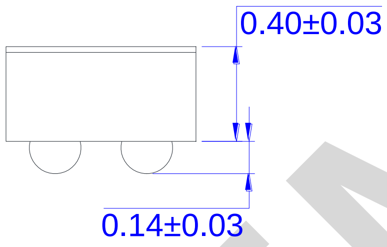

(SIDE VIEW)

MEMSIC

RECOMMENDED LAND PATTERN DESIGN

UNIT: mm

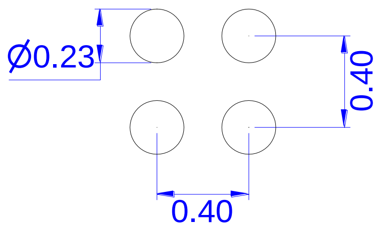

RELATIONSHIP BETWEEN THE MAGNETIC FIELD AND OUTPUT CODE

The measurement data increases as the magnetic flux density increases in the arrow directions.

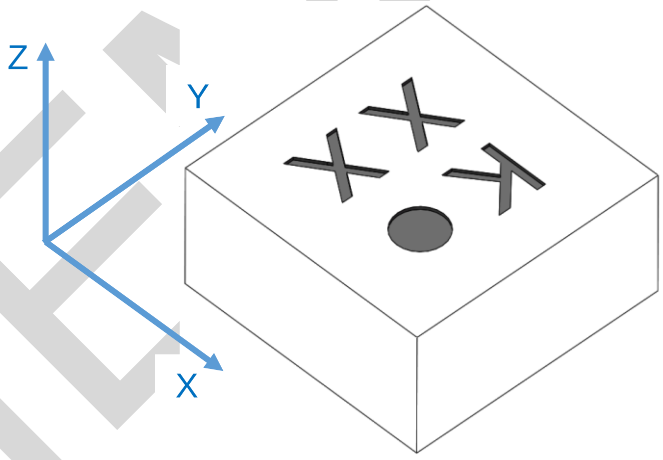

MEMSIC
