# Supplementary material for: The Unfolding Space Glove: A Wearable Spatio-Visual to Haptic Sensory Substitution Device for Blind People
Source: Sensors (Basel). 2022 Feb 26;22(5):1859. doi: 10.3390/s22051859 (PMC8914703; doi:10.3390/s22051859)
Supplement: Supplementary file 1 [file sensors-22-01859-s001.zip › S1_GitHub-Release_unfolding-space-hardware-0.2.1-revised.zip!/unfolding-space-hardware-0.2.1-revised/pcb/datasheets/PCA9635.pdf]

PCA9635

16-bit Fm+ I²C-bus LED driver

Rev. 07 — 16 July 2009 Product data sheet

1. General description

The PCA9635 is an I²C-bus controlled 16-bit LED driver optimized for Red/Green/Blue/Amber (RGBA) color mixing applications. Each LED output has its own 8-bit resolution (256 steps) fixed frequency individual PWM controller that operates at 97 kHz with a duty cycle that is adjustable from 0 % to 99.6 % to allow the LED to be set to a specific brightness value. An additional 8-bit resolution (256 steps) group PWM controller has both a fixed frequency of 190 Hz and an adjustable frequency between 24 Hz to once every 10.73 seconds with a duty cycle that is adjustable from 0 % to 99.6 % that is used to either dim or blink all LEDs with the same value.

Each LED output can be off, on (no PWM control), set at its individual PWM controller value or at both individual and group PWM controller values. The LED output driver is programmed to be either open-drain with a 25 mA current sink capability at 5 V or totem-pole with a 25 mA sink, 10 mA source capability at 5 V. The PCA9635 operates with a supply voltage range of 2.3 V to 5.5 V and the outputs are 5.5 V tolerant. LEDs can be directly connected to the LED output (up to 25 mA, 5.5 V) or controlled with external drivers and a minimum amount of discrete components for larger current or higher voltage LEDs.

The PCA9635 is one of the first LED controller devices in a new Fast-mode Plus (Fm+) family. Fm+ devices offer higher frequency (up to 1 MHz) and more densely populated bus operation (up to 4000 pF).

The active LOW Output Enable input pin ($\overline{OE}$) allows asynchronous control of the LED outputs and can be used to set all the outputs to a defined I²C-bus programmable logic state. The $\overline{OE}$ can also be used to externally PWM the outputs, which is useful when multiple devices need to be dimmed or blinked together using software control.

Software programmable LED Group and three Sub Call I²C-bus addresses allow all or defined groups of PCA9635 devices to respond to a common I²C-bus address, allowing for example, all red LEDs to be turned on or off at the same time or marquee chasing effect, thus minimizing I²C-bus commands. Seven hardware address pins allow up to 126 devices on the same bus.

The Software Reset (SWRST) Call allows the master to perform a reset of the PCA9635 through the I²C-bus, identical to the Power-On Reset (POR) that initializes the registers to their default state causing the outputs to be set HIGH (LED off). This allows an easy and quick way to reconfigure all device registers to the same condition.

founded by Philips

2. Features

- 16 LED drivers. Each output programmable at:
  - Off
  - On
  - Programmable LED brightness
  - Programmable group dimming/blinking mixed with individual LED brightness
- 1 MHz Fast-mode Plus compatible I²C-bus interface with 30 mA high drive capability on SDA output for driving high capacitive buses
- 256-step (8-bit) linear programmable brightness per LED output varying from fully off (default) to maximum brightness using a 97 kHz PWM signal
- 256-step group brightness control allows general dimming (using a 190 Hz PWM signal) from fully off to maximum brightness (default)
- 256-step group blinking with frequency programmable from 24 Hz to 10.73 s and duty cycle from 0 % to 99.6 %
- Sixteen totem-pole outputs (sink 25 mA and source 10 mA at 5 V) with software programmable open-drain LED outputs selection (default at totem-pole). No input function.
- Output state change programmable on the Acknowledge or the STOP Command to update outputs byte-by-byte or all at the same time (default to 'Change on STOP').
- Active LOW Output Enable ($\overline{OE}$) input pin. LED outputs programmable to logic 1, logic 0 or 'high-impedance' (default at power-up) when $\overline{OE}$ is HIGH, thus allowing hardware blinking and dimming of the LEDs.
- 7 hardware address pins allow 126 devices to be connected to the same I²C-bus
- 4 software programmable I²C-bus addresses (one LED Group Call address and three LED Sub Call addresses) allow groups of devices to be addressed at the same time in any combination (for example, one register used for 'All Call' so that all the PCA9635s on the I²C-bus can be addressed at the same time and the second register used for three different addresses so that ⅓ of all devices on the bus can be addressed at the same time in a group). Software enable and disable for I²C-bus address.
- Software Reset feature (SWRST Call) allows the device to be reset through the I²C-bus
- 25 MHz internal oscillator requires no external components
- Internal power-on reset
- Noise filter on SDA/SCL inputs
- Edge rate control on outputs
- No glitch on power-up
- Supports hot insertion
- Low standby current
- Operating power supply voltage range of 2.3 V to 5.5 V
- 5.5 V tolerant inputs
- –40 °C to +85 °C operation
- ESD protection exceeds 2000 V HBM per JESD22-A114, 200 V MM per JESD22-A115 and 1000 V CDM per JESD22-C101
- Latch-up testing is done to JEDEC Standard JESD78 which exceeds 100 mA
- Packages offered: TSSOP28

© NXP B.V. 2009. All rights reserved.

3. Applications

- RGB or RGBA LED drivers
- LED status information
- LED displays
- LCD backlights
- Keypad backlights for cellular phones or handheld devices

4. Ordering information

Table 1. Ordering information

Type number	Topside mark	Package		
		Name	Description	Version
PCA9635PW	PCA9635PW	TSSOP28	plastic thin shrink small outline package; 28 leads; body width 4.4 mm	SOT361-1
PCA9635PW/Q900[1]	PCA9635PW	TSSOP28	plastic thin shrink small outline package; 28 leads; body width 4.4 mm	SOT361-1

[1] PCA9635PW/Q900 is AEC-Q100 compliant. Contact **i2c.support@nxp.com** for PPAP.

5. Block diagram

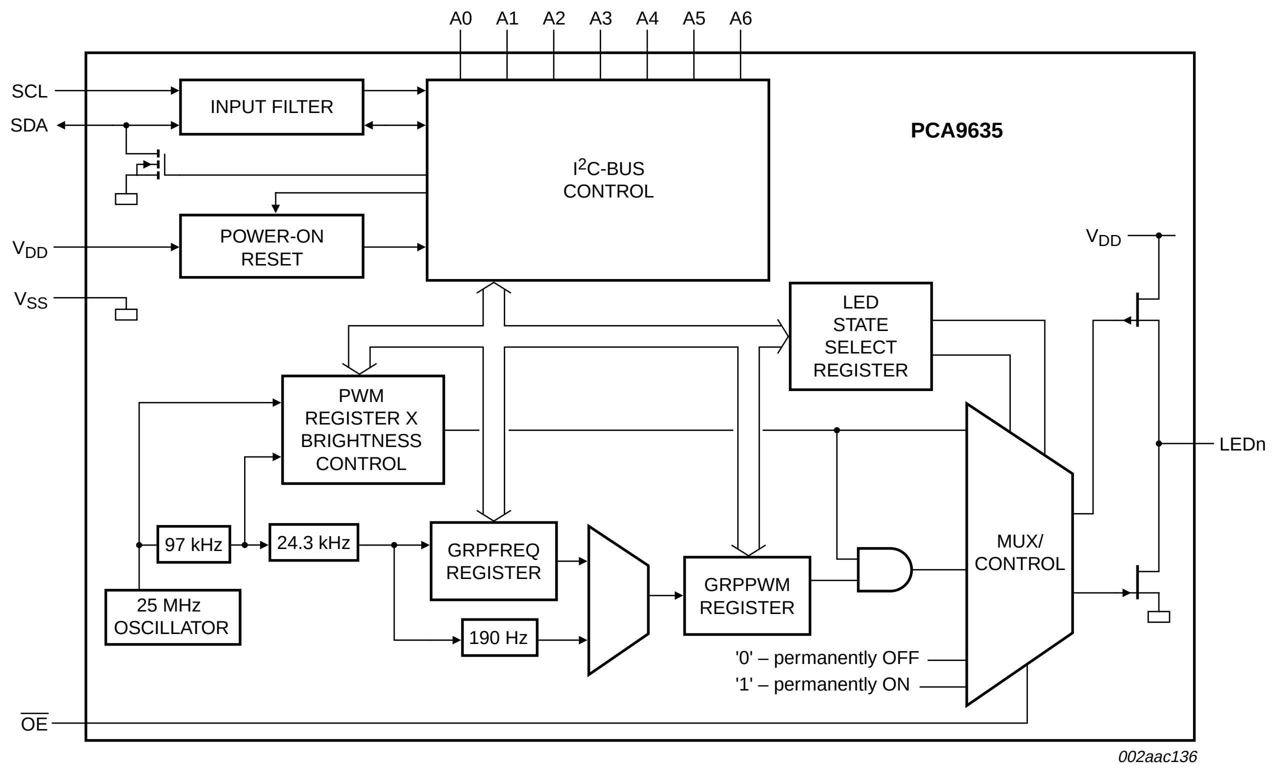

Remark: Only one LED output shown for clarity.

Fig 1. Block diagram of PCA9635

© NXP B.V. 2009. All rights reserved.

6. Pinning information

6.1 Pinning

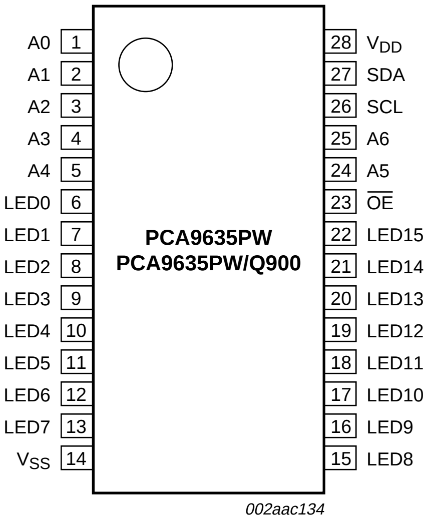

Fig 2. Pin configuration for TSSOP28

6.2 Pin description

Table 2. Pin description

Symbol	Pin	Type	Description
A0	1	I	address input 0
A1	2	I	address input 1
A2	3	I	address input 2
A3	4	I	address input 3
A4	5	I	address input 4
LED0	6	O	LED driver 0
LED1	7	O	LED driver 1
LED2	8	O	LED driver 2
LED3	9	O	LED driver 3
LED4	10	O	LED driver 4
LED5	11	O	LED driver 5
LED6	12	O	LED driver 6
LED7	13	O	LED driver 7
V_{SS}	14	power supply	supply ground
LED8	15	O	LED driver 8
LED9	16	O	LED driver 9
LED10	17	O	LED driver 10
LED11	18	O	LED driver 11
LED12	19	O	LED driver 12

© NXP B.V. 2009. All rights reserved.

Table 2. Pin description *...continued*

Symbol	Pin	Type	Description
LED13	20	O	LED driver 13
LED14	21	O	LED driver 14
LED15	22	O	LED driver 15
$\overline{OE}$	23	I	active LOW output enable
A5	24	I	address input 5
A6	25	I	address input 6
SCL	26	I	serial clock line
SDA	27	I/O	serial data line
V_{DD}	28	power supply	supply voltage

7. Functional description

Refer to Figure 1 “Block diagram of PCA9635”.

7.1 Device addresses

Following a START condition, the bus master must output the address of the slave it is accessing.

There are a maximum of 128 possible programmable addresses using the 7 hardware address pins. Two of these addresses, Software Reset and LED All Call, cannot be used because their default power-up state is ON, leaving a maximum of 126 addresses. Using other reserved addresses, as well as any other Sub Call address, will reduce the total number of possible addresses even further.

7.1.1 Regular I²C-bus slave address

The I²C-bus slave address of the PCA9635 is shown in Figure 3. To conserve power, no internal pull-up resistors are incorporated on the hardware selectable address pins and they must be pulled HIGH or LOW.

Remark: Using reserved I²C-bus addresses will interfere with other devices, but only if the devices are on the bus and/or the bus will be open to other I²C-bus systems at some later date. In a closed system where the designer controls the address assignment these addresses can be used since the PCA9635 treats them like any other address. The LED All Call, Software Rest and PCA9564 or PCA9665 slave address (if on the bus) can never be used for individual device addresses.

- PCA9635 LED All Call address (1110 000) and Software Reset (0000 0110) which are active on start-up
- PCA9564 (0000 000) or PCA9665 (1110 000) slave address which is active on start-up
- ‘reserved for future use’ I²C-bus addresses (0000 011, 1111 1XX)
- slave devices that use the 10-bit addressing scheme (1111 0XX)
- slave devices that are designed to respond to the General Call address (0000 000)
- High-speed mode (Hs-mode) master code (0000 1XX)

© NXP B.V. 2009. All rights reserved.

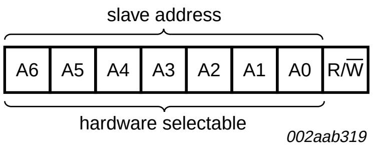

Fig 3. Slave address

The last bit of the address byte defines the operation to be performed. When set to logic 1 a read is selected, while a logic 0 selects a write operation.

7.1.2 LED all call I²C-bus address

- Default power-up value (ALLCALLADR register): E0h or 1110 000X
- Programmable through I²C-bus (volatile programming)
- At power-up, LED All Call I²C-bus address is enabled. PCA9635 sends an ACK when E0h ($R/\overline{W}$ = 0) or E1h ($R/\overline{W}$ = 1) is sent by the master.

See Section 7.3.8 "ALLCALLADR, LED All Call I²C-bus address" for more detail.

Remark: The default LED All Call I²C-bus address (E0h or 1110 000X) must not be used as a regular I²C-bus slave address since this address is enabled at power-up. All the PCA9635s on the I²C-bus will acknowledge the address if sent by the I²C-bus master.

7.1.3 LED sub call I²C-bus addresses

- 3 different I²C-bus addresses can be used
- Default power-up values:
  - SUBADR1 register: E2h or 1110 001X
  - SUBADR2 register: E4h or 1110 010X
  - SUBADR3 register: E8h or 1110 100X
- Programmable through I²C-bus (volatile programming)
- At power-up, Sub Call I²C-bus addresses are disabled. PCA9635 does not send an ACK when E2h ($R/\overline{W}$ = 0) or E3h ($R/\overline{W}$ = 1), E4h ($R/\overline{W}$ = 0) or E5h ($R/\overline{W}$ = 1), or E8h ($R/\overline{W}$ = 0) or E9h ($R/\overline{W}$ = 1) is sent by the master.

See Section 7.3.7 "SUBADR1 to SUBADR3, I²C-bus subaddress 1 to 3" for more detail.

Remark: The default LED Sub Call I²C-bus addresses may be used as regular I²C-bus slave addresses as long as they are disabled.

© NXP B.V. 2009. All rights reserved.

7.1.4 Software reset I²C-bus address

The address shown in Figure 4 is used when a reset of the PCA9635 needs to be performed by the master. The Software Reset address (SWRST Call) must be used with $R/\overline{W}$ = logic 0. If $R/\overline{W}$ = logic 1, the PCA9635 does not acknowledge the SWRST. See Section 7.6 "Software reset" for more detail.

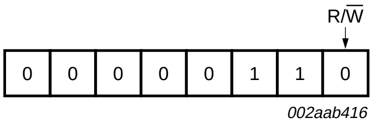

Fig 4. Software Reset address

Remark: The Software Reset I²C-bus address is a reserved address and cannot be used as a regular I²C-bus slave address or as an LED All Call or LED Sub Call address.

7.2 Control register

Following the successful acknowledgement of the slave address, LED All Call address or LED Sub Call address, the bus master will send a byte to the PCA9635, which will be stored in the Control register.

The lowest 5 bits are used as a pointer to determine which register will be accessed (D[4:0]). The highest 3 bits are used as Auto-Increment flag and Auto-Increment options (AI[2:0]).

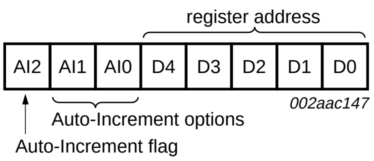

reset state = 80h

Remark: The Control register does not apply to the Software Reset I²C-bus address.

Fig 5. Control register

When the Auto-Increment flag is set (AI2 = logic 1), the five low order bits of the Control register are automatically incremented after a read or write. This allows the user to program the registers sequentially. Four different types of Auto-Increment are possible, depending on AI1 and AI0 values.

© NXP B.V. 2009. All rights reserved.

Table 3. Auto-Increment options

AI2	AI1	AI0	Function
0	0	0	no Auto-Increment
1	0	0	Auto-Increment for all registers. D[4:0] roll over to ‘0 0000’ after the last register (1 1011) is accessed.
1	0	1	Auto-Increment for individual brightness registers only. D[4:0] roll over to ‘0 0010’ after the last register (1 0001) is accessed.
1	1	0	Auto-Increment for global control registers only. D[4:0] roll over to ‘1 0010’ after the last register (1 0011) is accessed.
1	1	1	Auto-Increment for individual and global control registers only. D[4:0] roll over to ‘0 0010’ after the last register (1 0011) is accessed.

Remark: Other combinations not shown in Table 3 (AI[2:0] = 001, 010, and 011) are reserved and must not be used for proper device operation.

AI[2:0] = 000 is used when the same register must be accessed several times during a single I^{2}C-bus communication, for example, changes the brightness of a single LED. Data is overwritten each time the register is accessed during a write operation.

AI[2:0] = 100 is used when all the registers must be sequentially accessed, for example, power-up programming.

AI[2:0] = 101 is used when the four LED drivers must be individually programmed with different values during the same I^{2}C-bus communication, for example, changing color setting to another color setting.

AI[2:0] = 110 is used when the LED drivers must be globally programmed with different settings during the same I^{2}C-bus communication, for example, global brightness or blinking change.

AI[2:0] = 111 is used when individual and global changes must be performed during the same I^{2}C-bus communication, for example, changing a color and global brightness at the same time.

Only the 5 least significant bits D[4:0] are affected by the AI[2:0] bits.

When the Control register is written, the register entry point determined by D[4:0] is the first register that will be addressed (read or write operation), and can be anywhere between 0 0000 and 1 1011 (as defined in Table 4). When AI[2] = 1, the Auto-Increment flag is set and the rollover value at which the register increment stops and goes to the next one is determined by AI[2:0]. See Table 3 for rollover values. For example, if the Control register = 1111 0100 (F4h), then the register addressing sequence will be (in hex): 14 → … → 1B → 00 → … → 13 → 02 → … → 13 → 02 → … → 13 → 02 → … as long as the master keeps sending or reading data.

© NXP B.V. 2009. All rights reserved.

7.3 Register definitions

Table 4. Register summary[1][2]

Register number (hex)	D4	D3	D2	D1	D0	Name	Type	Function
00	0	0	0	0	0	MODE1	read/write	Mode register 1
01	0	0	0	0	1	MODE2	read/write	Mode register 2
02	0	0	0	1	0	PWM0	read/write	brightness control LED0
03	0	0	0	1	1	PWM1	read/write	brightness control LED1
04	0	0	1	0	0	PWM2	read/write	brightness control LED2
05	0	0	1	0	1	PWM3	read/write	brightness control LED3
06	0	0	1	1	0	PWM4	read/write	brightness control LED4
07	0	0	1	1	1	PWM5	read/write	brightness control LED5
08	0	1	0	0	0	PWM6	read/write	brightness control LED6
09	0	1	0	0	1	PWM7	read/write	brightness control LED7
0A	0	1	0	1	0	PWM8	read/write	brightness control LED8
0B	0	1	0	1	1	PWM9	read/write	brightness control LED9
0C	0	1	1	0	0	PWM10	read/write	brightness control LED10
0D	0	1	1	0	1	PWM11	read/write	brightness control LED11
0E	0	1	1	1	0	PWM12	read/write	brightness control LED12
0F	0	1	1	1	1	PWM13	read/write	brightness control LED13
10	1	0	0	0	0	PWM14	read/write	brightness control LED14
11	1	0	0	0	1	PWM15	read/write	brightness control LED15
12	1	0	0	1	0	GRPPWM	read/write	group duty cycle control
13	1	0	0	1	1	GRPFREQ	read/write	group frequency
14	1	0	1	0	0	LEDOUT0	read/write	LED output state 0
15	1	0	1	0	1	LEDOUT1	read/write	LED output state 1
16	1	0	1	1	0	LEDOUT2	read/write	LED output state 2
17	1	0	1	1	1	LEDOUT3	read/write	LED output state 3
18	1	1	0	0	0	SUBADR1	read/write	I^{2}C-bus subaddress 1
19	1	1	0	0	1	SUBADR2	read/write	I^{2}C-bus subaddress 2
1A	1	1	0	1	0	SUBADR3	read/write	I^{2}C-bus subaddress 3
1B	1	1	0	1	1	ALLCALLADR	read/write	LED All Call I^{2}C-bus address

[1] Only D[4:0] = 0 0000 to 1 1011 are allowed and will be acknowledged. D[4:0] = 1 1100 to 1 1111 are reserved and will not be acknowledged.

[2] When writing to the Control register, bit 4 must be programmed with logic 0 for proper device operation.

© NXP B.V. 2009. All rights reserved.

7.3.1 Mode register 1, MODE1

Table 5. MODE1 - Mode register 1 (address 00h) bit description

*Legend: * default value.*

Bit	Symbol	Access	Value	Description
7	AI2	read only	0	Register Auto-Increment disabled.
			1*	Register Auto-Increment enabled.
6	AI1	read only	0*	Auto-Increment bit 1 = 0.
			1	Auto-Increment bit 1 = 1.
5	AI0	read only	0*	Auto-Increment bit 0 = 0.
			1	Auto-Increment bit 0 = 1.
4	SLEEP	R/W	0	Normal mode[1].
			1*	Low power mode. Oscillator off[2].
3	SUB1	R/W	0*	PCA9635 does not respond to I^2C-bus subaddress 1.
			1	PCA9635 responds to I^2C-bus subaddress 1.
2	SUB2	R/W	0*	PCA9635 does not respond to I^2C-bus subaddress 2.
			1	PCA9635 responds to I^2C-bus subaddress 2.
1	SUB3	R/W	0*	PCA9635 does not respond to I^2C-bus subaddress 3.
			1	PCA9635 responds to I^2C-bus subaddress 3.
0	ALLCALL	R/W	0	PCA9635 does not respond to LED All Call I^2C-bus address.
			1*	PCA9635 responds to LED All Call I^2C-bus address.

[1] It takes 500 μs max. for the oscillator to be up and running once SLEEP bit has been set to logic 0. Timings on LEDn outputs are not guaranteed if PWMx, GRPPWM or GRPFREQ registers are accessed within the 500 μs window.

[2] When the oscillator is off (Sleep mode) the LED outputs cannot be turned on, off or dimmed/blinked.

7.3.2 Mode register 2, MODE2

Table 6. MODE2 - Mode register 2 (address 01h) bit description

*Legend: * default value.*

Bit	Symbol	Access	Value	Description
7	-	read only	0*	reserved
6	-	read only	0*	reserved
5	DMBLNK	R/W	0*	group control = dimming.
			1	group control = blinking.
4	INVRT[1]	R/W	0*	Output logic state not inverted. Value to use when no external driver used. Applicable when $\overline{OE}$ = 0.
			1	Output logic state inverted. Value to use when external driver used. Applicable when $\overline{OE}$ = 0.
3	OCH	R/W	0*	Outputs change on STOP command.[2]
			1	Outputs change on ACK.
2	OUTDRV[1]	R/W	0	The 16 LED outputs are configured with an open-drain structure.
			1*	The 16 LED outputs are configured with a totem-pole structure.

© NXP B.V. 2009. All rights reserved.

Table 6. MODE2 - Mode register 2 (address 01h) bit description *...continued*
*Legend: * default value.*

Bit	Symbol	Access	Value	Description
1 to 0	OUTNE[1:0][3]	R/W	00	When $\overline{OE}$ = 1 (output drivers not enabled), LEDn = 0.
			01*	When $\overline{OE}$ = 1 (output drivers not enabled):<br>LEDn = 1 when OUTDRV = 1<br>LEDn = high-impedance when OUTDRV = 0 (same as OUTNE[1:0] = 10)
			10	When $\overline{OE}$ = 1 (output drivers not enabled), LEDn = high-impedance.
			11	reserved

[1] See Section 7.7 "Using the PCA9635 with and without external drivers" for more details. Normal LEDs can be driven directly in either mode. Some newer LEDs include integrated Zener diodes to limit voltage transients, reduce EMI and protect the LEDs, and these must be driven only in the open-drain mode to prevent overheating the IC.

[2] Change of the outputs at the STOP command allows synchronizing outputs of more than one PCA9635. Applicable to registers from 02h (PWM0) to 17h (LEDOUT) only.

[3] See Section 7.4 "Active LOW output enable input" for more details.

7.3.3 PWM0 to PWM15, individual brightness control

Table 7. PWM0 to PWM15 - PWM registers 0 to 15 (address 02h to 11h) bit description
*Legend: * default value.*

Address	Register	Bit	Symbol	Access	Value	Description
02h	PWM0	7:0	IDC0[7:0]	R/W	0000 0000*	PWM0 Individual Duty Cycle
03h	PWM1	7:0	IDC1[7:0]	R/W	0000 0000*	PWM1 Individual Duty Cycle
04h	PWM2	7:0	IDC2[7:0]	R/W	0000 0000*	PWM2 Individual Duty Cycle
05h	PWM3	7:0	IDC3[7:0]	R/W	0000 0000*	PWM3 Individual Duty Cycle
06h	PWM4	7:0	IDC4[7:0]	R/W	0000 0000*	PWM4 Individual Duty Cycle
07h	PWM5	7:0	IDC5[7:0]	R/W	0000 0000*	PWM5 Individual Duty Cycle
08h	PWM6	7:0	IDC6[7:0]	R/W	0000 0000*	PWM6 Individual Duty Cycle
09h	PWM7	7:0	IDC7[7:0]	R/W	0000 0000*	PWM7 Individual Duty Cycle
0Ah	PWM8	7:0	IDC8[7:0]	R/W	0000 0000*	PWM8 Individual Duty Cycle
0Bh	PWM9	7:0	IDC9[7:0]	R/W	0000 0000*	PWM9 Individual Duty Cycle
0Ch	PWM10	7:0	IDC10[7:0]	R/W	0000 0000*	PWM10 Individual Duty Cycle
0Dh	PWM11	7:0	IDC11[7:0]	R/W	0000 0000*	PWM11 Individual Duty Cycle
0Eh	PWM12	7:0	IDC12[7:0]	R/W	0000 0000*	PWM12 Individual Duty Cycle
0Fh	PWM13	7:0	IDC13[7:0]	R/W	0000 0000*	PWM13 Individual Duty Cycle
10h	PWM14	7:0	IDC14[7:0]	R/W	0000 0000*	PWM14 Individual Duty Cycle
11h	PWM15	7:0	IDC15[7:0]	R/W	0000 0000*	PWM15 Individual Duty Cycle

A 97 kHz fixed frequency signal is used for each output. Duty cycle is controlled through 256 linear steps from 00h (0 % duty cycle = LED output off) to FFh (99.6 % duty cycle = LED output at maximum brightness). Applicable to LED outputs programmed with LDRx = 10 or 11 (LEDOUT0 to LEDOUT3 registers).

$$duty\ cycle = \frac{IDCx[7:0]}{256} \quad (1)$$

© NXP B.V. 2009. All rights reserved.

7.3.4 GRPPWM, group duty cycle control

Table 8. GRPPWM - Group brightness control register (address 12h) bit description
*Legend: * default value*

Address	Register	Bit	Symbol	Access	Value	Description
12h	GRPPWM	7:0	GDC[7:0]	R/W	1111 1111	GRPPWM register

When DMBLNK bit (MODE2 register) is programmed with logic 0, a 190 Hz fixed frequency signal is superimposed with the 97 kHz individual brightness control signal. GRPPWM is then used as a global brightness control allowing the LED outputs to be dimmed with the same value. The value in GRPFREQ is then a ‘Don’t care’.

General brightness for the 16 outputs is controlled through 256 linear steps from 00h (0 % duty cycle = LED output off) to FFh (99.6 % duty cycle = maximum brightness). Applicable to LED outputs programmed with LDRx = 11 (LEDOUT0 to LEDOUT3 registers).

When DMBLNK bit is programmed with logic 1, GRPPWM and GRPFREQ registers define a global blinking pattern, where GRPFREQ contains the blinking period (from 24 Hz to 10.73 s) and GRPPWM the duty cycle (ON/OFF ratio in %).

$$duty\ cycle = \frac{GDC[7:0]}{256} \qquad (2)$$

7.3.5 GRPFREQ, group frequency

Table 9. GRPFREQ - Group Frequency register (address 13h) bit description
*Legend: * default value.*

Address	Register	Bit	Symbol	Access	Value	Description
13h	GRPFREQ	7:0	GFRQ[7:0]	R/W	0000 0000*	GRPFREQ register

GRPFREQ is used to program the global blinking period when DMBLNK bit (MODE2 register) is equal to 1. Value in this register is a ‘Don’t care’ when DMBLNK = 0. Applicable to LED outputs programmed with LDRx = 11 (LEDOUT0 to LEDOUT3 registers).

Blinking period is controlled through 256 linear steps from 00h (41 ms, frequency 24 Hz) to FFh (10.73 s).

$$global\ blinking\ period = \frac{GFRQ[7:0] + 1}{24}\ (s) \qquad (3)$$

© NXP B.V. 2009. All rights reserved.

7.3.6 LEDOUT0 to LEDOUT3, LED driver output state

Table 10. LEDOUT0 to LEDOUT3 - LED driver output state register (address 14h to 17h) bit description

*Legend: * default value.*

Address	Register	Bit	Symbol	Access	Value	Description
14h	LEDOUT0	7:6	LDR3	R/W	00*	LED3 output state control
		5:4	LDR2	R/W	00*	LED2 output state control
		3:2	LDR1	R/W	00*	LED1 output state control
		1:0	LDR0	R/W	00*	LED0 output state control
15h	LEDOUT1	7:6	LDR7	R/W	00*	LED7 output state control
		5:4	LDR6	R/W	00*	LED6 output state control
		3:2	LDR5	R/W	00*	LED5 output state control
		1:0	LDR4	R/W	00*	LED4 output state control
16h	LEDOUT2	7:6	LDR11	R/W	00*	LED11 output state control
		5:4	LDR10	R/W	00*	LED10 output state control
		3:2	LDR9	R/W	00*	LED9 output state control
		1:0	LDR8	R/W	00*	LED8 output state control
17h	LEDOUT3	7:6	LDR15	R/W	00*	LED15 output state control
		5:4	LDR14	R/W	00*	LED14 output state control
		3:2	LDR13	R/W	00*	LED13 output state control
		1:0	LDR12	R/W	00*	LED12 output state control

LDRx = 00 — LED driver x is off (default power-up state).

LDRx = 01 — LED driver x is fully on (individual brightness and group dimming/blinking not controlled).

LDRx = 10 — LED driver x individual brightness can be controlled through its PWMx register.

LDRx = 11 — LED driver x individual brightness and group dimming/blinking can be controlled through its PWMx register and the GRPPWM registers.

© NXP B.V. 2009. All rights reserved.

7.3.7 SUBADR1 to SUBADR3, I^2C-bus subaddress 1 to 3

Table 11. SUBADR1 to SUBADR3 - I^2C-bus subaddress registers 1 to 3 (address 18h to 1Ah) bit description

*Legend: * default value.*

Address	Register	Bit	Symbol	Access	Value	Description
18h	SUBADR1	7:1	A1[7:1]	R/W	1110 001*	I^2C-bus subaddress 1
		0	A1[0]	R only	0*	reserved
19h	SUBADR2	7:1	A2[7:1]	R/W	1110 010*	I^2C-bus subaddress 2
		0	A2[0]	R only	0*	reserved
1Ah	SUBADR3	7:1	A3[7:1]	R/W	1110 100*	I^2C-bus subaddress 3
		0	A3[0]	R only	0*	reserved

Subaddresses are programmable through the I^2C-bus. Default power-up values are E2h, E4h, E8h, and the device(s) will not acknowledge these addresses right after power-up (the corresponding SUBx bit in MODE1 register is equal to 0).

Once subaddresses have been programmed to their right values, SUBx bits need to be set to logic 1 in order to have the device acknowledging these addresses (MODE1 register).

Only the 7 MSBs representing the I^2C-bus subaddress are valid. The LSB in SUBADRx register is a read-only bit (0).

When SUBx is set to logic 1, the corresponding I^2C-bus subaddress can be used during either an I^2C-bus read or write sequence.

7.3.8 ALLCALLADR, LED All Call I^2C-bus address

Table 12. ALLCALLADR - LED All Call I^2C-bus address register (address 1Bh) bit description

*Legend: * default value.*

Address	Register	Bit	Symbol	Access	Value	Description
1Bh	ALLCALLADR	7:1	AC[7:1]	R/W	1110 000*	ALLCALL I^2C-bus address register
		0	AC[0]	R only	0*	reserved

The LED All Call I^2C-bus address allows all the PCA9635s in the bus to be programmed at the same time (ALLCALL bit in register MODE1 must be equal to 1 (power-up default state)). This address is programmable through the I^2C-bus and can be used during either an I^2C-bus read or write sequence. The register address can also be programmed as a Sub Call.

Only the 7 MSBs representing the All Call I^2C-bus address are valid. The LSB in ALLCALLADR register is a read-only bit (0).

If ALLCALL bit = 0, the device does not acknowledge the address programmed in register ALLCALLADR.

© NXP B.V. 2009. All rights reserved.

7.4 Active LOW output enable input

The active LOW output enable ($\overline{OE}$) pin, allows to enable or disable all the LED outputs at the same time.

- When a LOW level is applied to $\overline{OE}$ pin, all the LED outputs are enabled and follow the output state defined in the LEDOUT register with the polarity defined by INVRT bit (MODE2 register).
- When a HIGH level is applied to $\overline{OE}$ pin, all the LED outputs are programmed to the value that is defined by OUTNE[1:0] in the MODE2 register.

Table 13. LED outputs when $\overline{OE}$ = 1

OUTNE1	OUTNE0	LED outputs
0	0	0
0	1	1 if OUTDRV = 1, high-impedance if OUTDRV = 0
1	0	high-impedance
1	1	reserved

The $\overline{OE}$ pin can be used as a synchronization signal to switch on/off several PCA9635 devices at the same time. This requires an external clock reference that provides blinking period and the duty cycle.

The $\overline{OE}$ pin can also be used as an external dimming control signal. The frequency of the external clock must be high enough not to be seen by the human eye, and the duty cycle value determines the brightness of the LEDs.

Remark: Do not use $\overline{OE}$ as an external blinking control signal when internal global blinking is selected (DMBLNK = 1, MODE2 register) since it will result in an undefined blinking pattern. Do not use $\overline{OE}$ as an external dimming control signal when internal global dimming is selected (DMBLNK = 0, MODE2 register) since it will result in an undefined dimming pattern.

7.5 Power-on reset

When power is applied to V_{DD}, an internal power-on reset holds the PCA9635 in a reset condition until V_{DD} has reached V_{POR}. At this point, the reset condition is released and the PCA9635 registers and I^{2}C-bus state machine are initialized to their default states (all zeroes) causing all the channels to be deselected. Thereafter, V_{DD} must be lowered below 0.2 V to reset the device.

© NXP B.V. 2009. All rights reserved.

7.6 Software reset

The Software Reset Call (SWRST Call) allows all the devices in the I^{2}C-bus to be reset to the power-up state value through a specific formatted I^{2}C-bus command. To be performed correctly, it implies that the I^{2}C-bus is functional and that there is no device hanging the bus.

The SWRST Call function is defined as the following:

1. A START command is sent by the I^{2}C-bus master.
2. The reserved SWRST I^{2}C-bus address ‘0000 011’ with the $R/\overline{W}$ bit set to ‘0’ (write) is sent by the I^{2}C-bus master.
3. The PCA9635 device(s) acknowledge(s) after seeing the SWRST Call address ‘0000 0110’ (06h) only. If the $R/\overline{W}$ bit is set to ‘1’ (read), no acknowledge is returned to the I^{2}C-bus master.
4. Once the SWRST Call address has been sent and acknowledged, the master sends 2 bytes with 2 specific values (SWRST data byte 1 and byte 2):
    a. Byte 1 = A5h: the PCA9635 acknowledges this value only. If byte 1 is not equal to A5h, the PCA9635 does not acknowledge it.
    b. Byte 2 = 5Ah: the PCA9635 acknowledges this value only. If byte 2 is not equal to 5Ah, then the PCA9635 does not acknowledge it.

    If more than 2 bytes of data are sent, the PCA9635 does not acknowledge any more.
5. Once the right 2 bytes (SWRST data byte 1 and byte 2 only) have been sent and correctly acknowledged, the master sends a STOP command to end the SWRST Call: the PCA9635 then resets to the default value (power-up value) and is ready to be addressed again within the specified bus free time (t_{BUF}).

The I^{2}C-bus master must interpret a non-acknowledge from the PCA9635 (at any time) as a ‘SWRST Call Abort’. The PCA9635 does not initiate a reset of its registers. This happens only when the format of the SWRST Call sequence is not correct.

7.7 Using the PCA9635 with and without external drivers

The PCA9635 LED output drivers are 5.5 V only tolerant and can sink up to 25 mA at 5 V.

If the device needs to drive LEDs to a higher voltage and/or higher current, use of an external driver is required.

- INVRT bit (MODE2 register) can be used to keep the LED PWM control firmware the same (PWMx and GRPPWM values directly calculated from their respective formulas and the LED output state determined by LEDOUT register value) independently of the type of external driver. This bit allows LED output polarity inversion/non-inversion only when $\overline{OE}$ = 0.
- OUTDRV bit (MODE2 register) allows minimizing the amount of external components required to control the external driver (N-type or P-type device).

© NXP B.V. 2009. All rights reserved.

Table 14. Use of INVRT and OUTDRV based on connection to the LEDn outputs when $\overline{OE}$ = 0[1]

INVRT	OUTDRV	Direct connection to LEDn		External N-type driver		External P-type driver	
		Firmware	External pull-up resistor	Firmware	External pull-up resistor	Firmware	External pull-up resistor
0	0	formulas and LED output state values apply[2]	LED current limiting R[2]	formulas and LED output state values inverted	required	formulas and LED output state values apply	required
0	1	formulas and LED output state values apply[2]	LED current limiting R[2]	formulas and LED output state values inverted	not required	formulas and LED output state values apply[4]	not required[4]
1	0	formulas and LED output state values inverted	LED current limiting R	formulas and LED output state values apply	required	formulas and LED output state values inverted	required
1	1	formulas and LED output state values inverted	LED current limiting R	formulas and LED output state values apply[3]	not required[3]	formulas and LED output state values inverted	not required

[1] When $\overline{OE}$ = 1, LED output state is controlled only by OUTNE[1:0] bits (MODE2 register).

[2] Correct configuration when LEDs directly connected to the LEDn outputs (connection to V_{DD} through current limiting resistor).

[3] Optimum configuration when external N-type (NPN, NMOS) driver used.

[4] Optimum configuration when external P-type (PNP, PMOS) driver used.

Table 15. Output transistors based on LEDOUT registers, INVRT and OUTDRV bits when $\overline{OE}$ = 0[1]

LEDOUT	INVRT	OUTDRV	Upper transistor (V_{DD} to LEDn)	Lower transistor (LEDn to V_{SS})	LEDn state
00 LED driver off	0	0	off	off	high-Z[2]
	0	1	on	off	V_{DD}
	1	0	off	on	V_{SS}
	1	1	off	on	V_{SS}
01 LED driver on	0	0	off	on	V_{SS}
	0	1	off	on	V_{SS}
	1	0	off	off	high-Z[2]
	1	1	on	off	V_{DD}
10 Individual brightness control	0	0	off	individual PWM (non-inverted)	V_{SS} or high-Z[2] = PWMx value
	0	1	individual PWM (non-inverted)	individual PWM (non-inverted)	V_{SS} or V_{DD} = PWMx value
	1	0	off	individual PWM (inverted)	high-Z[2] or V_{SS} = 1 – PWMx value
	1	1	individual PWM (inverted)	individual PWM (inverted)	V_{DD} or V_{SS} = 1 – PWMx value

© NXP B.V. 2009. All rights reserved.

Table 15. Output transistors based on LEDOUT registers, INVRT and OUTDRV bits when $\overline{OE}$ = 0[1] *…continued*

LEDOUT	INVRT	OUTDRV	Upper transistor (V_{DD} to LEDn)	Lower transistor (LEDn to V_{SS})	LEDn state
11<br>individual + group dimming/blinking	0	0	off	individual + group PWM (non-inverted)	V_{SS} or high-Z[2] = PWMx or GRPPWM values
	0	1	individual PWM (non-inverted)	individual PWM (non-inverted)	V_{SS} or V_{DD} = PWMx or GRPPWM values
	1	0	off	individual + group PWM (inverted)	high-Z[2] or V_{SS} = (1 – PWMx) or (1 – GRPPWM) values
	1	1	individual PWM (inverted)	individual PWM (inverted)	V_{DD} or V_{SS} = (1 – PWMx) or (1 – GRPPWM) values

[1] When $\overline{OE}$ = 1, LED output state is controlled only by OUTNE[1:0] bits (MODE2 register).

[2] External pull-up or LED current limiting resistor connects LEDn to V_{DD}.

7.8 Individual brightness control with group dimming/blinking

A 97 kHz fixed frequency signal with programmable duty cycle (8 bits, 256 steps) is used to control individually the brightness for each LED.

On top of this signal, one of the following signals can be superimposed (this signal can be applied to the 4 LED outputs):

- A lower 190 Hz fixed frequency signal with programmable duty cycle (8 bits, 256 steps) is used to provide a global brightness control.
- A programmable frequency signal from 24 Hz to $\frac{1}{10.73}$ Hz (8 bits, 256 steps) with programmable duty cycle (8 bits, 256 steps) is used to provide a global blinking control.

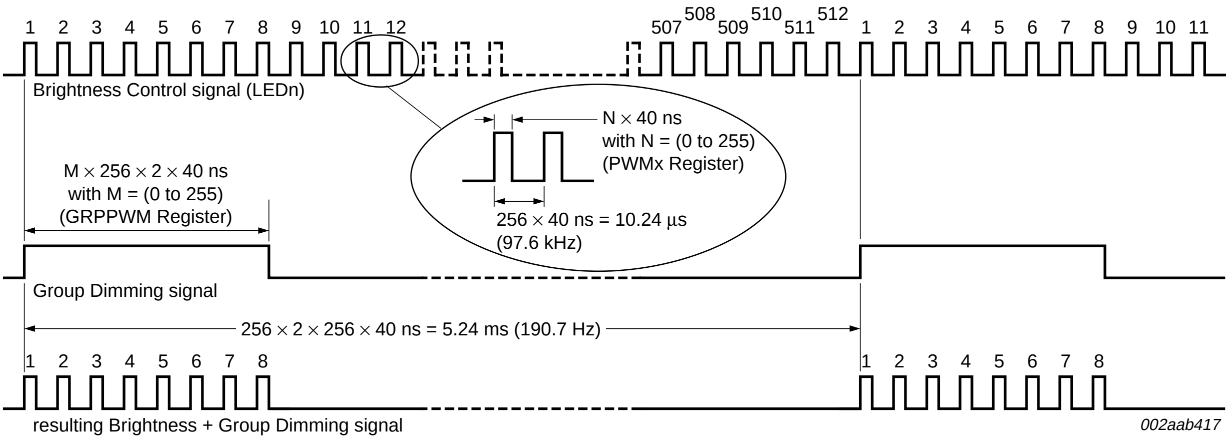

Minimum pulse width for LEDn Brightness Control is 40 ns.

Minimum pulse width for Group Dimming is 20.48 µs.

When M = 1 (GRPPWM register value), the resulting LEDn Brightness Control + Group Dimming signal will have 2 pulses of the LED Brightness Control signal (pulse width = N × 40 ns, with ‘N’ defined in PWMx register).

This resulting Brightness + Group Dimming signal above shows a resulting Control signal with M = 4 (8 pulses).

Fig 6. Brightness + Group Dimming signals

© NXP B.V. 2009. All rights reserved.

8. Characteristics of the I^{2}C-bus

The I^{2}C-bus is for 2-way, 2-line communication between different ICs or modules. The two lines are a serial data line (SDA) and a serial clock line (SCL). Both lines must be connected to a positive supply via a pull-up resistor when connected to the output stages of a device. Data transfer may be initiated only when the bus is not busy.

8.1 Bit transfer

One data bit is transferred during each clock pulse. The data on the SDA line must remain stable during the HIGH period of the clock pulse as changes in the data line at this time will be interpreted as control signals (see Figure 7).

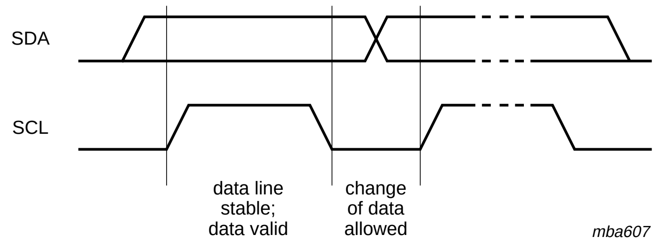

Fig 7. Bit transfer

8.1.1 START and STOP conditions

Both data and clock lines remain HIGH when the bus is not busy. A HIGH-to-LOW transition of the data line while the clock is HIGH is defined as the START condition (S). A LOW-to-HIGH transition of the data line while the clock is HIGH is defined as the STOP condition (P) (see Figure 8).

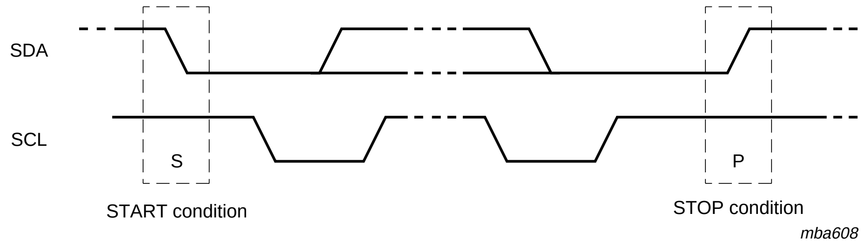

Fig 8. Definition of START and STOP conditions

8.2 System configuration

A device generating a message is a 'transmitter'; a device receiving is the 'receiver'. The device that controls the message is the 'master' and the devices which are controlled by the master are the 'slaves' (see Figure 9).

© NXP B.V. 2009. All rights reserved.

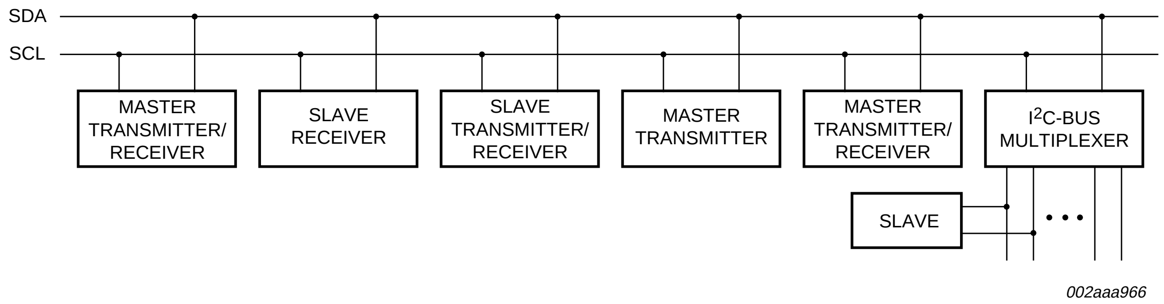

Fig 9. System configuration

8.3 Acknowledge

The number of data bytes transferred between the START and the STOP conditions from transmitter to receiver is not limited. Each byte of eight bits is followed by one acknowledge bit. The acknowledge bit is a HIGH level put on the bus by the transmitter, whereas the master generates an extra acknowledge related clock pulse.

A slave receiver which is addressed must generate an acknowledge after the reception of each byte. Also a master must generate an acknowledge after the reception of each byte that has been clocked out of the slave transmitter. The device that acknowledges has to pull down the SDA line during the acknowledge clock pulse, so that the SDA line is stable LOW during the HIGH period of the acknowledge related clock pulse; set-up time and hold time must be taken into account.

A master receiver must signal an end of data to the transmitter by not generating an acknowledge on the last byte that has been clocked out of the slave. In this event, the transmitter must leave the data line HIGH to enable the master to generate a STOP condition.

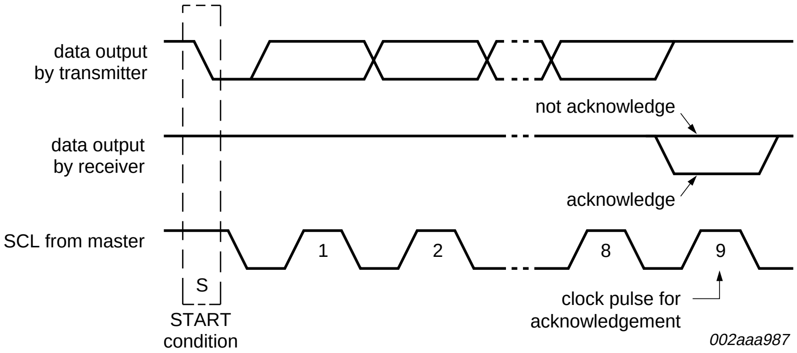

Fig 10. Acknowledgement on the I²C-bus

© NXP B.V. 2009. All rights reserved.

9. Bus transactions

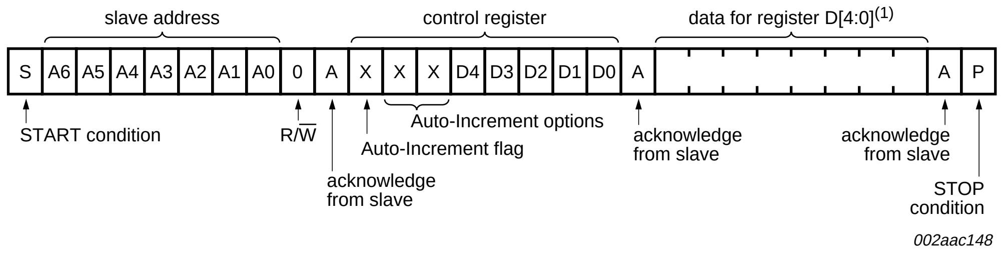

(1) See Table 4 for register definition.

Fig 11. Write to a specific register

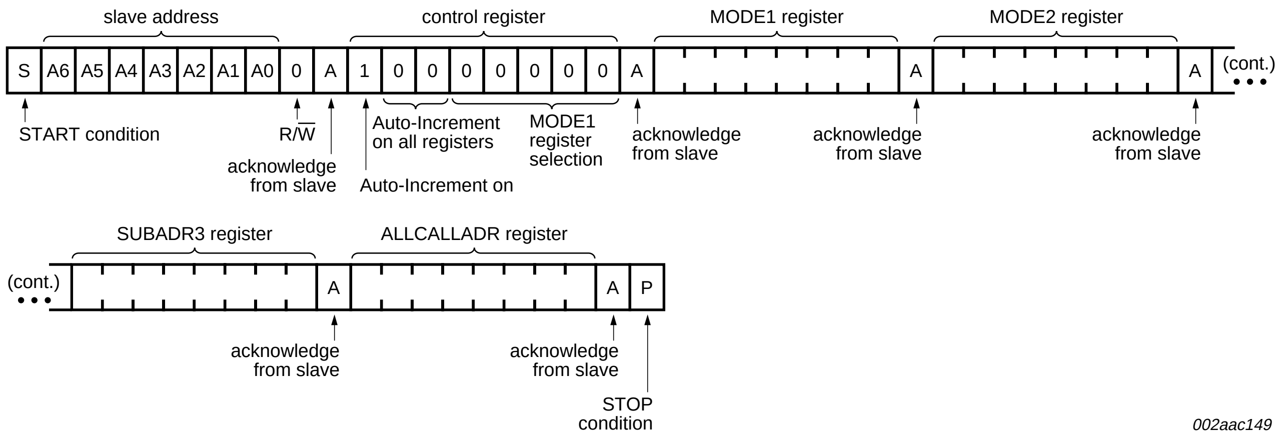

Fig 12. Write to all registers using the Auto-Increment feature

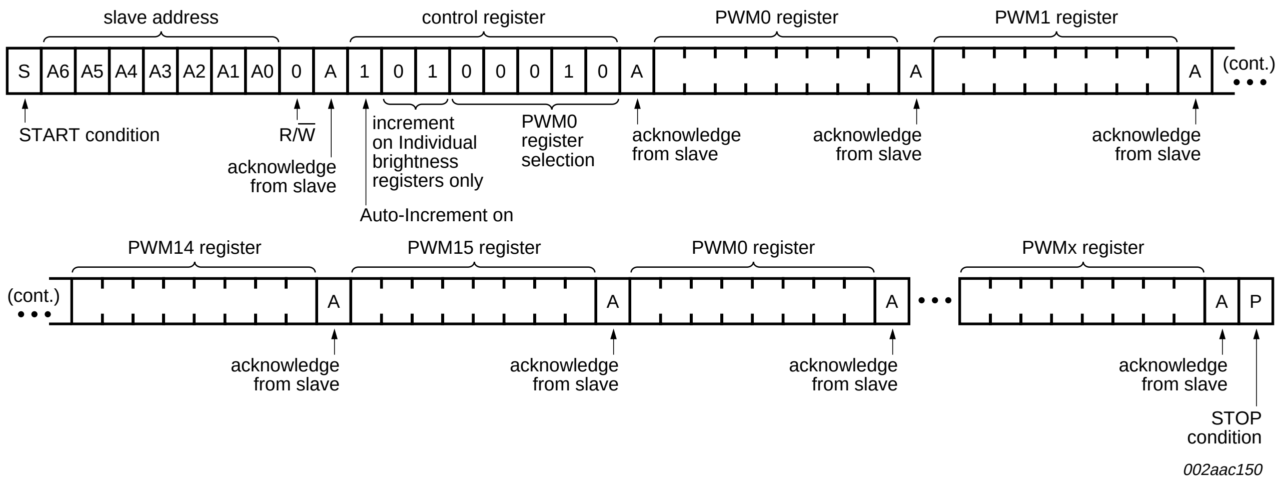

Fig 13. Multiple writes to Individual Brightness registers only using the Auto-Increment feature

© NXP B.V. 2009. All rights reserved.

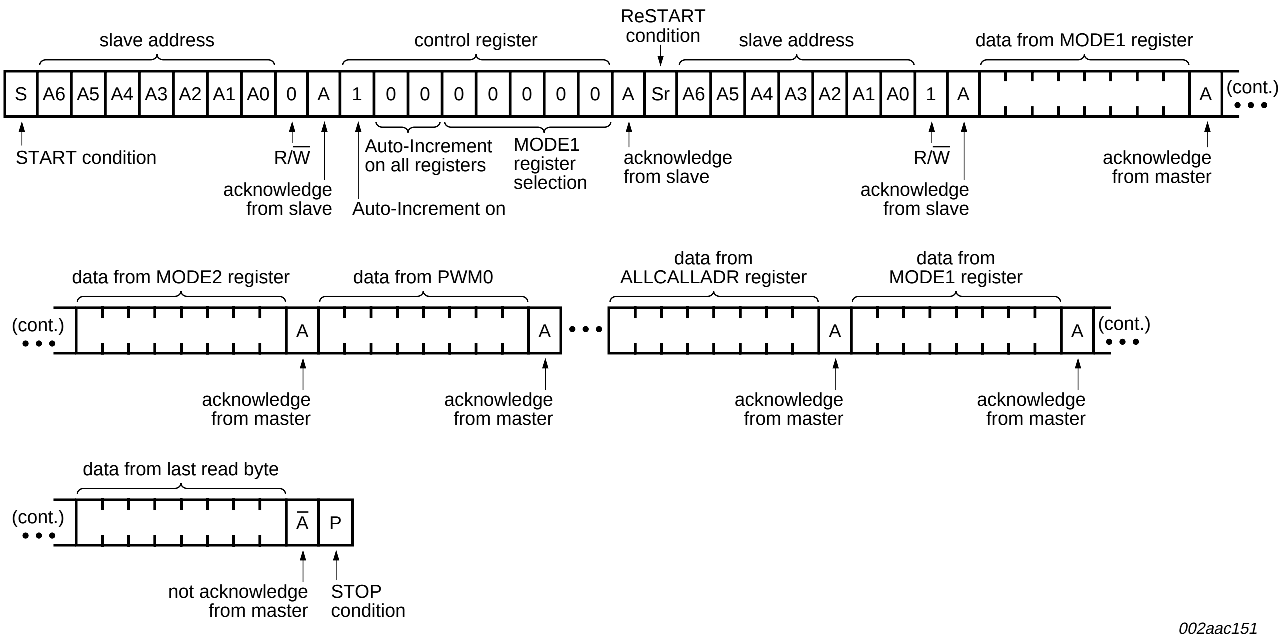

Fig 14. Read all registers using the Auto-Increment feature

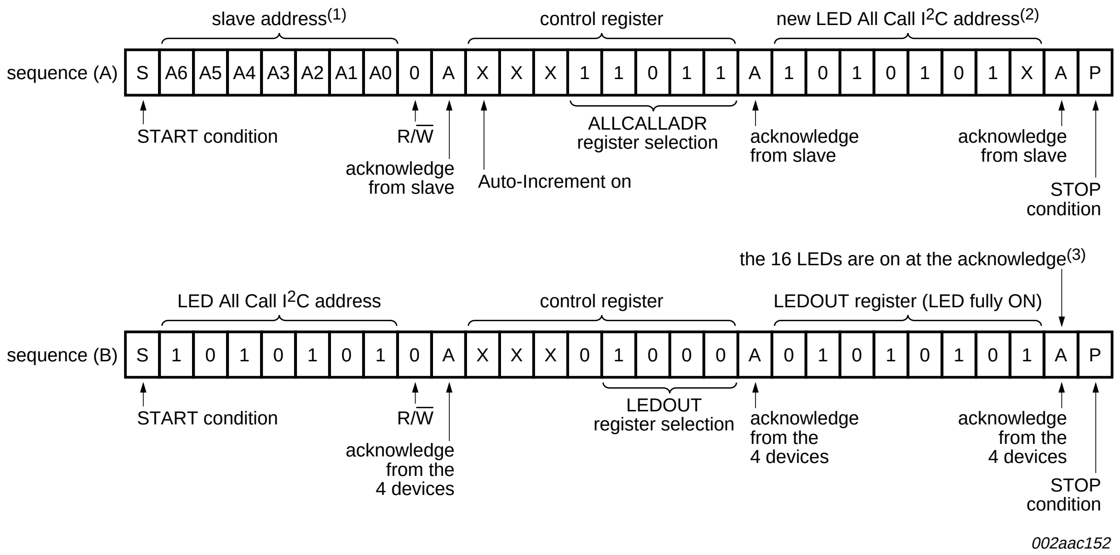

(1) In this example, several PCA9635s are used and the same sequence (A) (above) is sent to each of them.

(2) ALLCALL bit in MODE1 register is equal to 1 for this example.

(3) OCH bit in MODE2 register is equal to 1 for this example.

Fig 15. LED All Call I²C-bus address programming and LED All Call sequence example

© NXP B.V. 2009. All rights reserved.

10. Application design-in information

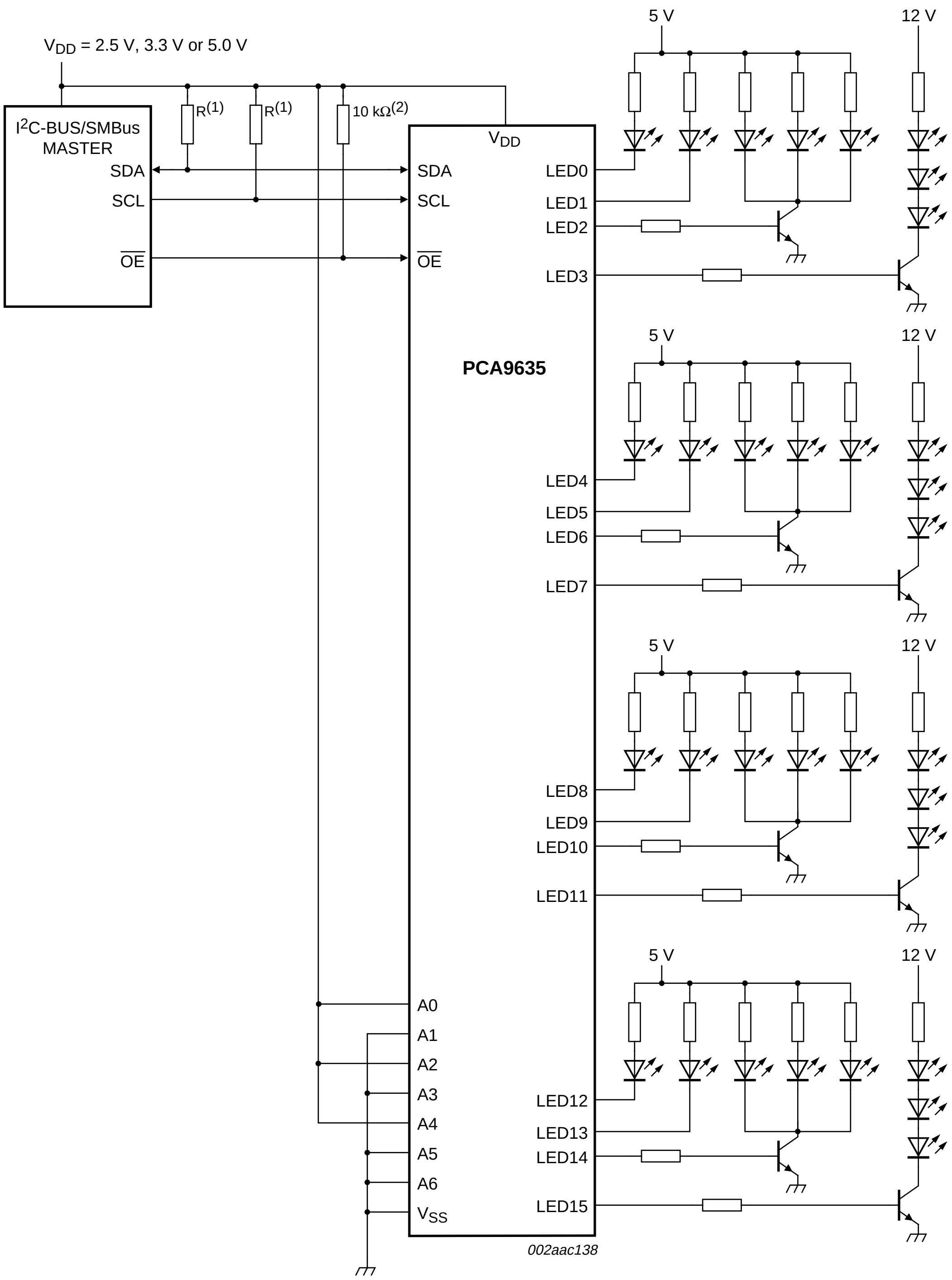

(1) R = 10 kΩ (typical) for SMBus, Standard-mode or Fast-mode I²C-bus. R = 1 kΩ (typical) for Fast-mode Plus I²C-bus.

(2) OE requires pull-up resistor if control signal from the master is open-drain.

I²C-bus address = 0010 101x.

Fig 16. Typical application

© NXP B.V. 2009. All rights reserved.

Question 1: What kind of edge rate control is there on the outputs?

- The typical edge rates depend on the output configuration, supply voltage, and the applied load. The outputs can be configured as either open-drain NMOS or totem-pole outputs. If the customer is using the part to directly drive LEDs, they should be using it in an open-drain NMOS, if they are concerned about the maximum I_{SS} and ground bounce. The edge rate control was designed primarily to slow down the turn-on of the output device; it turns off rather quickly (~1.5 ns). In simulation, the typical turn-on time for the open-drain NMOS was ~14 ns (V_{DD} = 3.6 V; C_L = 50 pF; R_{PU} = 500 Ω).

Question 2: Is ground bounce possible?

- Ground bounce is a possibility, especially if all 16 outputs are changed at full current (25 mA each). There is a fair amount of decoupling capacitance on chip (~50 pF), which is intended to suppress some of the ground bounce. The customer will need to determine if additional decoupling capacitance externally placed as close as physically possible to the device is required.

Question 3: Can I really sink 400 mA through the single ground pin on the package and will this cause any ground bounce problem due to the PWM of the LEDs?

- Yes, you can sink 400 mA through a single ground pin on the **package**. Although the package only has one ground pin, there are two ground pads on the die itself connected to this one pin. Although some ground bounce is likely, it will not disrupt the operation of the part and would be reduced by the external decoupling capacitance.

Question 4: I can’t turn the LEDs on or off, but their registers are set properly. Why?

- Check the Mode Register 1 bit 4 SLEEP setting. The value needs to be 0 so that the OSC is turn on. If the OSC is turned off, the LEDs cannot be turned on or off and also can’t be dimmed or blinked.

Question 5: I’m using LEDs with integrated Zener diodes and the IC is getting very hot. Why?

- The IC outputs can be set to either open-drain or push-pull and default to push-pull outputs. In this application with the Zener diodes, they need to be set to open-drain since in the push-pull architecture there is a low resistance path to GND through the Zener and this is causing the IC to overheat. The PCA9632/33/34/35 ICs all power-up in the push-pull output mode and with the logic state HIGH, so one of the first things that need to be done is to set the outputs to open-drain.

© NXP B.V. 2009. All rights reserved.

11. Limiting values

Table 16. Limiting values

In accordance with the Absolute Maximum Rating System (IEC 60134).

Symbol	Parameter	Conditions	Min	Max	Unit
V_{DD}	supply voltage		–0.5	+6.0	V
$V_{I/O}$	voltage on an input/output pin		V_{SS} – 0.5	5.5	V
$I_{O(LEDn)}$	output current on pin LEDn		-	25	mA
I_{SS}	ground supply current		-	400	mA
P_{tot}	total power dissipation		-	400	mW
T_{stg}	storage temperature		–65	+150	°C
T_{amb}	ambient temperature	operating	–40	+85	°C

12. Static characteristics

Table 17. Static characteristics

V_{DD} = 2.3 V to 5.5 V; V_{SS} = 0 V; T_{amb} = –40 °C to +85 °C; unless otherwise specified.

Symbol	Parameter	Conditions		Min	Typ	Max	Unit
**Supply**							
V_{DD}	supply voltage			2.3	-	5.5	V
I_{DD}	supply current	operating mode; no load; f_{SCL} = 1 MHz					
		V_{DD} = 2.3 V		-	2.5	10	mA
		V_{DD} = 3.3 V		-	2.5	10	mA
		V_{DD} = 5.5 V		-	2.5	10	mA
I_{stb}	standby current	no load; f_{SCL} = 0 Hz; I/O = inputs; V_I = V_{DD}					
		V_{DD} = 2.3 V		-	2.3	11	µA
		V_{DD} = 3.3 V		-	2.9	12	µA
		V_{DD} = 5.5 V		-	3.8	15.5	µA
V_{POR}	power-on reset voltage	no load; V_I = V_{DD} or V_{SS}	[1]	-	1.70	2.0	V
**Input SCL; input/output SDA**							
V_{IL}	LOW-level input voltage			–0.5	-	+0.3V_{DD}	V
V_{IH}	HIGH-level input voltage			0.7V_{DD}	-	5.5	V
I_{OL}	LOW-level output current	V_{OL} = 0.4 V; V_{DD} = 2.3 V		20	-	-	mA
		V_{OL} = 0.4 V; V_{DD} = 5.0 V		30	-	-	mA
I_L	leakage current	V_I = V_{DD} or V_{SS}		–1	-	+1	µA
C_i	input capacitance	V_I = V_{SS}		-	6	10	pF

© NXP B.V. 2009. All rights reserved.

Table 17. Static characteristics *...continued*

V_{DD} = 2.3 V to 5.5 V; V_{SS} = 0 V; T_{amb} = –40 °C to +85 °C; unless otherwise specified.

Symbol	Parameter	Conditions		Min	Typ	Max	Unit
**LED driver outputs**							
I_{OL}	LOW-level output current	V_{OL} = 0.5 V; V_{DD} = 2.3 V	[2]	12	-	-	mA
		V_{OL} = 0.5 V; V_{DD} = 3.0 V	[2]	17	-	-	mA
		V_{OL} = 0.5 V; V_{DD} = 4.5 V	[2]	25	-	-	mA
$I_{OL(tot)}$	total LOW-level output current	V_{OL} = 0.5 V; V_{DD} = 4.5 V	[2]	-	-	400	mA
I_{OH}	HIGH-level output current	open-drain; V_{OH} = V_{DD}		–50	-	+50	µA
V_{OH}	HIGH-level output voltage	I_{OH} = –10 mA; V_{DD} = 2.3 V		1.6	-	-	V
		I_{OH} = –10 mA; V_{DD} = 3.0 V		2.3	-	-	V
		I_{OH} = –10 mA; V_{DD} = 4.5 V		4.0	-	-	V
C_o	output capacitance			-	2.5	5	pF
**$\overline{OE}$ input**							
V_{IL}	LOW-level input voltage			–0.5	-	+0.8	V
V_{IH}	HIGH-level input voltage			2	-	5.5	V
I_{LI}	input leakage current			–1	-	+1	µA
C_i	input capacitance			-	3.7	5	pF
**Address inputs**							
V_{IL}	LOW-level input voltage			–0.5	-	+0.3V_{DD}	V
V_{IH}	HIGH-level input voltage			0.7V_{DD}	-	5.5	V
I_{LI}	input leakage current			–1	-	+1	µA
C_i	input capacitance			-	3.7	5	pF

[1] V_{DD} must be lowered to 0.2 V for at least 5 ns in order to reset part.

[2] Each bit must be limited to a maximum of 25 mA and the total package limited to 400 mA due to internal busing limits.

© NXP B.V. 2009. All rights reserved.

13. Dynamic characteristics

Table 18. Dynamic characteristics

Symbol	Parameter	Conditions	Standard-mode I²C-bus		Fast-mode I²C-bus		Fast-mode Plus I²C-bus		Unit
			Min	Max	Min	Max	Min	Max	
f_{SCL}	SCL clock frequency	[1]	0	100	0	400	0	1000	kHz
t_{BUF}	bus free time between a STOP and START condition		4.7	-	1.3	-	0.5	-	µs
$t_{HD;STA}$	hold time (repeated) START condition		4.0	-	0.6	-	0.26	-	µs
$t_{SU;STA}$	set-up time for a repeated START condition		4.7	-	0.6	-	0.26	-	µs
$t_{SU;STO}$	set-up time for STOP condition		4.0	-	0.6	-	0.26	-	µs
$t_{HD;DAT}$	data hold time		0	-	0	-	0	-	ns
$t_{VD;ACK}$	data valid acknowledge time	[2]	0.3	3.45	0.1	0.9	0.05	0.45	µs
$t_{VD;DAT}$	data valid time	[3]	0.3	3.45	0.1	0.9	0.05	0.45	µs
$t_{SU;DAT}$	data set-up time		250	-	100	-	50	-	ns
t_{LOW}	LOW period of the SCL clock		4.7	-	1.3	-	0.5	-	µs
t_{HIGH}	HIGH period of the SCL clock		4.0	-	0.6	-	0.26	-	µs
t_f	fall time of both SDA and SCL signals	[4][5]	-	300	$20 + 0.1C_b$[6]	300	-	120	ns
t_r	rise time of both SDA and SCL signals		-	1000	$20 + 0.1C_b$[6]	300	-	120	ns
t_{SP}	pulse width of spikes that must be suppressed by the input filter	[7]	-	50	-	50	-	50	ns

[1] Minimum SCL clock frequency is limited by the bus time-out feature, which resets the serial bus interface if either SDA or SCL is held LOW for a minimum of 25 ms. Disable bus time-out feature for DC operation.

[2] $t_{VD;ACK}$ = time for Acknowledgement signal from SCL LOW to SDA (out) LOW.

[3] $t_{VD;DAT}$ = minimum time for SDA data out to be valid following SCL LOW.

[4] A master device must internally provide a hold time of at least 300 ns for the SDA signal (refer to the V_{IL} of the SCL signal) in order to bridge the undefined region of SCL's falling edge.

[5] The maximum t_f for the SDA and SCL bus lines is specified at 300 ns. The maximum fall time (t_f) for the SDA output stage is specified at 250 ns. This allows series protection resistors to be connected between the SDA and the SCL pins and the SDA/SCL bus lines without exceeding the maximum specified t_f.

[6] C_b = total capacitance of one bus line in pF.

[7] Input filters on the SDA and SCL inputs suppress noise spikes less than 50 ns.

© NXP B.V. 2009. All rights reserved.

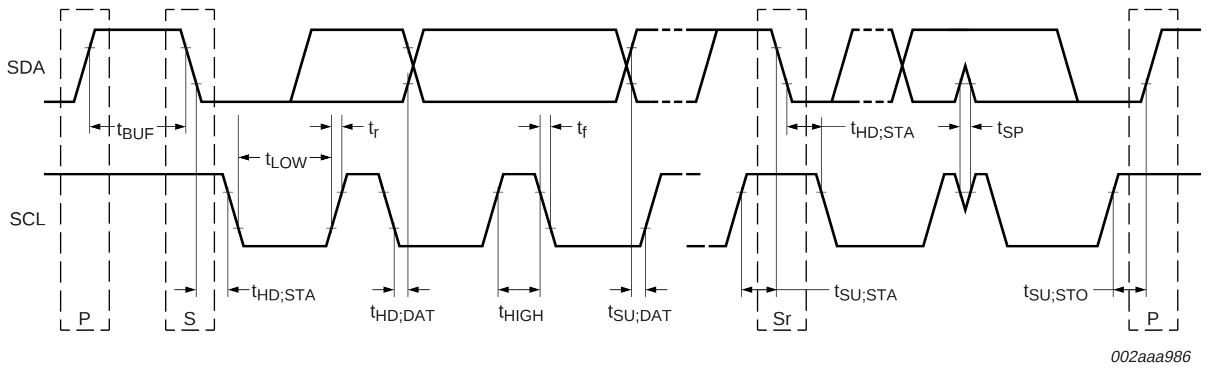

Fig 17. Definition of timing

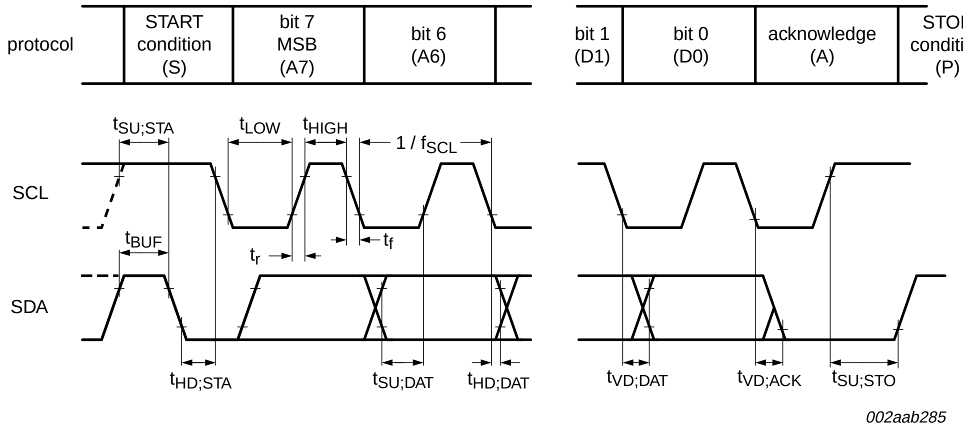

Rise and fall times refer to V_{IL} and V_{IH}.

Fig 18. I²C-bus timing diagram

14. Test information

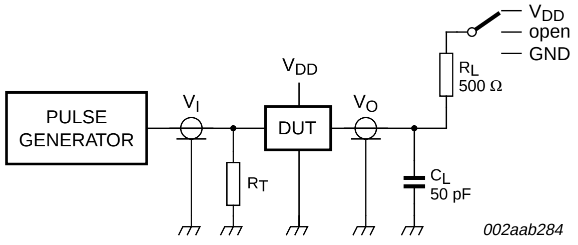

R_L = Load resistor for LEDn. R_L for SDA and SCL > 1 kΩ (3 mA or less current).

C_L = Load capacitance includes jig and probe capacitance.

R_T = Termination resistance should be equal to the output impedance Z_o of the pulse generators.

Fig 19. Test circuitry for switching times

© NXP B.V. 2009. All rights reserved.

15. Package outline

TSSOP28: plastic thin shrink small outline package; 28 leads; body width 4.4 mm **SOT361-1**

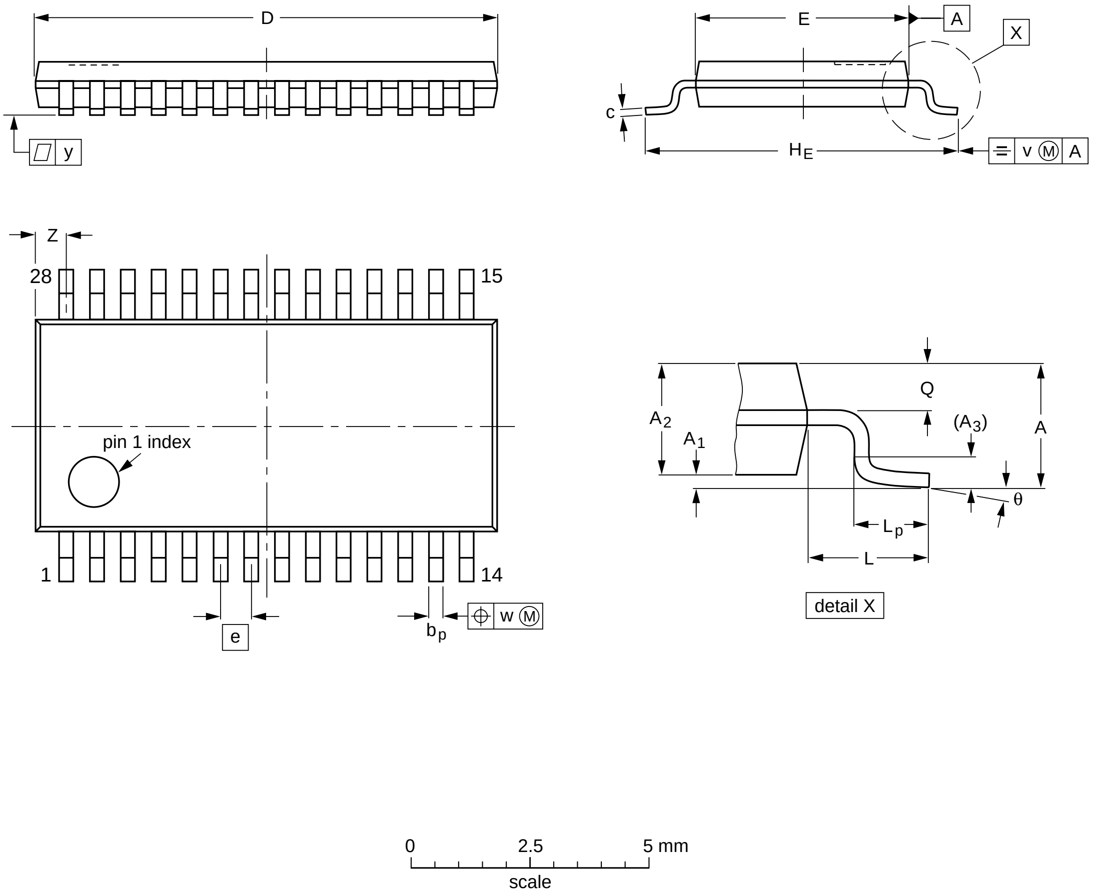

DIMENSIONS (mm are the original dimensions)

UNIT	A max.	A_1	A_2	A_3	b_p	c	D (1)	E (2)	e	H_E	L	L_p	Q	v	w	y	Z (1)	θ
mm	1.1	0.15<br>0.05	0.95<br>0.80	0.25	0.30<br>0.19	0.2<br>0.1	9.8<br>9.6	4.5<br>4.3	0.65	6.6<br>6.2	1	0.75<br>0.50	0.4<br>0.3	0.2	0.13	0.1	0.8<br>0.5	8°<br>0°

Notes

1. Plastic or metal protrusions of 0.15 mm maximum per side are not included.
2. Plastic interlead protrusions of 0.25 mm maximum per side are not included.

OUTLINE VERSION	REFERENCES				EUROPEAN PROJECTION	ISSUE DATE
	IEC	JEDEC	JEITA			
SOT361-1		MO-153				~~99-12-27~~<br>03-02-19

Fig 20. Package outline SOT361-1 (TSSOP28)

© NXP B.V. 2009. All rights reserved.

16. Handling information

All input and output pins are protected against ElectroStatic Discharge (ESD) under normal handling. When handling ensure that the appropriate precautions are taken as described in *JESD625-A* or equivalent standards.

17. Soldering of SMD packages

This text provides a very brief insight into a complex technology. A more in-depth account of soldering ICs can be found in Application Note *AN10365 "Surface mount reflow soldering description"*.

17.1 Introduction to soldering

Soldering is one of the most common methods through which packages are attached to Printed Circuit Boards (PCBs), to form electrical circuits. The soldered joint provides both the mechanical and the electrical connection. There is no single soldering method that is ideal for all IC packages. Wave soldering is often preferred when through-hole and Surface Mount Devices (SMDs) are mixed on one printed wiring board; however, it is not suitable for fine pitch SMDs. Reflow soldering is ideal for the small pitches and high densities that come with increased miniaturization.

17.2 Wave and reflow soldering

Wave soldering is a joining technology in which the joints are made by solder coming from a standing wave of liquid solder. The wave soldering process is suitable for the following:

- Through-hole components
- Leaded or leadless SMDs, which are glued to the surface of the printed circuit board

Not all SMDs can be wave soldered. Packages with solder balls, and some leadless packages which have solder lands underneath the body, cannot be wave soldered. Also, leaded SMDs with leads having a pitch smaller than ~0.6 mm cannot be wave soldered, due to an increased probability of bridging.

The reflow soldering process involves applying solder paste to a board, followed by component placement and exposure to a temperature profile. Leaded packages, packages with solder balls, and leadless packages are all reflow solderable.

Key characteristics in both wave and reflow soldering are:

- Board specifications, including the board finish, solder masks and vias
- Package footprints, including solder thieves and orientation
- The moisture sensitivity level of the packages
- Package placement
- Inspection and repair
- Lead-free soldering versus SnPb soldering

17.3 Wave soldering

Key characteristics in wave soldering are:

© NXP B.V. 2009. All rights reserved.

- Process issues, such as application of adhesive and flux, clinching of leads, board transport, the solder wave parameters, and the time during which components are exposed to the wave
- Solder bath specifications, including temperature and impurities

17.4 Reflow soldering

Key characteristics in reflow soldering are:

- Lead-free versus SnPb soldering; note that a lead-free reflow process usually leads to higher minimum peak temperatures (see Figure 21) than a SnPb process, thus reducing the process window
- Solder paste printing issues including smearing, release, and adjusting the process window for a mix of large and small components on one board
- Reflow temperature profile; this profile includes preheat, reflow (in which the board is heated to the peak temperature) and cooling down. It is imperative that the peak temperature is high enough for the solder to make reliable solder joints (a solder paste characteristic). In addition, the peak temperature must be low enough that the packages and/or boards are not damaged. The peak temperature of the package depends on package thickness and volume and is classified in accordance with Table 19 and 20

Table 19. SnPb eutectic process (from J-STD-020C)

Package thickness (mm)	Package reflow temperature (°C)	
	Volume (mm^3)	
	< 350	≥ 350
< 2.5	235	220
≥ 2.5	220	220

Table 20. Lead-free process (from J-STD-020C)

Package thickness (mm)	Package reflow temperature (°C)		
	Volume (mm^3)		
	< 350	350 to 2000	> 2000
< 1.6	260	260	260
1.6 to 2.5	260	250	245
> 2.5	250	245	245

Moisture sensitivity precautions, as indicated on the packing, must be respected at all times.

Studies have shown that small packages reach higher temperatures during reflow soldering, see Figure 21.

© NXP B.V. 2009. All rights reserved.

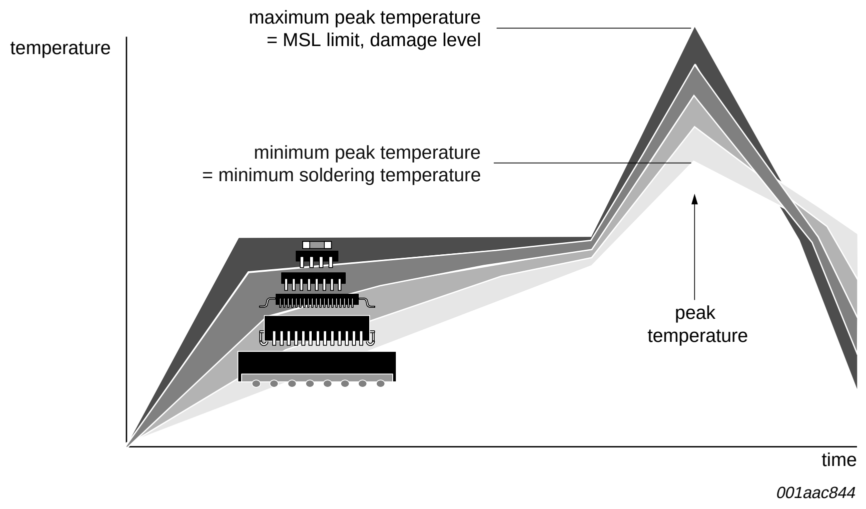

MSL: Moisture Sensitivity Level

Fig 21. Temperature profiles for large and small components

For further information on temperature profiles, refer to Application Note *AN10365 “Surface mount reflow soldering description”*.

18. Abbreviations

Table 21. Abbreviations

Acronym	Description
CDM	Charged Device Model
DUT	Device Under Test
EMI	ElectroMagnetic Interference
ESD	ElectroStatic Discharge
HBM	Human Body Model
I²C-bus	Inter-Integrated Circuit bus
LED	Light Emitting Diode
LSB	Least Significant Bit
MM	Machine Model
MSB	Most Significant Bit
NMOS	Negative-channel Metal Oxide Semiconductor
NPN	bipolar transistor with N-type emitter and collector and a P-type base
PCB	Printed-Circuit Board
PMOS	Positive-channel Metal Oxide Semiconductor
PNP	bipolar transistor with P-type emitter and collector and an N-type base
PWM	Pulse Width Modulation
RGB	Red/Green/Blue
RGBA	Red/Green/Blue/Amber
SMBus	System Management Bus

© NXP B.V. 2009. All rights reserved.

19. Revision history

Table 22. Revision history

Document ID	Release date	Data sheet status	Change notice	Supersedes
PCA9635_7	20090716	Product data sheet	-	PCA9635_6
Modifications:	• Added type number PCA9635PW/Q900 (affects Table 1 “Ordering information” and Figure 2 “Pin configuration for TSSOP28”)			
PCA9635_6	20080911	Product data sheet	-	PCA9635_5
PCA9635_5	20070322	Product data sheet	-	PCA9635_4
PCA9635_4	20061220	Product data sheet	-	PCA9635_3
PCA9635_3	20061116	Product data sheet	-	PCA9635_2
PCA9635_2	20060807	Objective data sheet	-	PCA9635_1
PCA9635_1	20060419	Objective data sheet	-	-

© NXP B.V. 2009. All rights reserved.

20. Legal information

20.1 Data sheet status

Document status[1][2]	Product status[3]	Definition
Objective [short] data sheet	Development	This document contains data from the objective specification for product development.
Preliminary [short] data sheet	Qualification	This document contains data from the preliminary specification.
Product [short] data sheet	Production	This document contains the product specification.

[1] Please consult the most recently issued document before initiating or completing a design.

[2] The term 'short data sheet' is explained in section "Definitions".

[3] The product status of device(s) described in this document may have changed since this document was published and may differ in case of multiple devices. The latest product status information is available on the Internet at URL http://www.nxp.com.

20.2 Definitions

Draft — The document is a draft version only. The content is still under internal review and subject to formal approval, which may result in modifications or additions. NXP Semiconductors does not give any representations or warranties as to the accuracy or completeness of information included herein and shall have no liability for the consequences of use of such information.

Short data sheet — A short data sheet is an extract from a full data sheet with the same product type number(s) and title. A short data sheet is intended for quick reference only and should not be relied upon to contain detailed and full information. For detailed and full information see the relevant full data sheet, which is available on request via the local NXP Semiconductors sales office. In case of any inconsistency or conflict with the short data sheet, the full data sheet shall prevail.

20.3 Disclaimers

General — Information in this document is believed to be accurate and reliable. However, NXP Semiconductors does not give any representations or warranties, expressed or implied, as to the accuracy or completeness of such information and shall have no liability for the consequences of use of such information.

Right to make changes — NXP Semiconductors reserves the right to make changes to information published in this document, including without limitation specifications and product descriptions, at any time and without notice. This document supersedes and replaces all information supplied prior to the publication hereof.

Suitability for use — NXP Semiconductors products are not designed, authorized or warranted to be suitable for use in medical, military, aircraft, space or life support equipment, nor in applications where failure or malfunction of an NXP Semiconductors product can reasonably be expected to result in personal injury, death or severe property or environmental damage. NXP Semiconductors accepts no liability for inclusion and/or use of NXP Semiconductors products in such equipment or applications and therefore such inclusion and/or use is at the customer's own risk.

Applications — Applications that are described herein for any of these products are for illustrative purposes only. NXP Semiconductors makes no representation or warranty that such applications will be suitable for the specified use without further testing or modification.

Limiting values — Stress above one or more limiting values (as defined in the Absolute Maximum Ratings System of IEC 60134) may cause permanent damage to the device. Limiting values are stress ratings only and operation of the device at these or any other conditions above those given in the Characteristics sections of this document is not implied. Exposure to limiting values for extended periods may affect device reliability.

Terms and conditions of sale — NXP Semiconductors products are sold subject to the general terms and conditions of commercial sale, as published at http://www.nxp.com/profile/terms, including those pertaining to warranty, intellectual property rights infringement and limitation of liability, unless explicitly otherwise agreed to in writing by NXP Semiconductors. In case of any inconsistency or conflict between information in this document and such terms and conditions, the latter will prevail.

No offer to sell or license — Nothing in this document may be interpreted or construed as an offer to sell products that is open for acceptance or the grant, conveyance or implication of any license under any copyrights, patents or other industrial or intellectual property rights.

Export control — This document as well as the item(s) described herein may be subject to export control regulations. Export might require a prior authorization from national authorities.

20.4 Trademarks

Notice: All referenced brands, product names, service names and trademarks are the property of their respective owners.

I^{2}C-bus — logo is a trademark of NXP B.V.

21. Contact information

For more information, please visit: **http://www.nxp.com**

For sales office addresses, please send an email to: **salesaddresses@nxp.com**

© NXP B.V. 2009. All rights reserved.

22. Contents

1 General description . . . 1
2 Features . . . 2
3 Applications . . . 3
4 Ordering information . . . 3
5 Block diagram . . . 3
6 Pinning information . . . 4
6.1 Pinning . . . 4
6.2 Pin description . . . 4
7 Functional description . . . 5
7.1 Device addresses . . . 5
7.1.1 Regular I^2C-bus slave address . . . 5
7.1.2 LED all call I^2C-bus address . . . 6
7.1.3 LED sub call I^2C-bus addresses . . . 6
7.1.4 Software reset I^2C-bus address . . . 7
7.2 Control register . . . 7
7.3 Register definitions . . . 9
7.3.1 Mode register 1, MODE1 . . . 10
7.3.2 Mode register 2, MODE2 . . . 10
7.3.3 PWM0 to PWM15, individual brightness control . . . 11
7.3.4 GRPPWM, group duty cycle control . . . 12
7.3.5 GRPFREQ, group frequency . . . 12
7.3.6 LEDOUT0 to LEDOUT3, LED driver output state . . . 13
7.3.7 SUBADR1 to SUBADR3, I^2C-bus subaddress 1 to 3 . . . 14
7.3.8 ALLCALLADR, LED All Call I^2C-bus address . 14
7.4 Active LOW output enable input . . . 15
7.5 Power-on reset . . . 15
7.6 Software reset . . . 16
7.7 Using the PCA9635 with and without external drivers . . . 16
7.8 Individual brightness control with group dimming/blinking . . . 18
8 Characteristics of the I^2C-bus . . . 19
8.1 Bit transfer . . . 19
8.1.1 START and STOP conditions . . . 19
8.2 System configuration . . . 19
8.3 Acknowledge . . . 20
9 Bus transactions . . . 21
10 Application design-in information . . . 23
11 Limiting values . . . 25
12 Static characteristics . . . 25
13 Dynamic characteristics . . . 27
14 Test information . . . 28
15 Package outline . . . 29
16 Handling information . . . 30
17 Soldering of SMD packages . . . 30
17.1 Introduction to soldering . . . 30
17.2 Wave and reflow soldering . . . 30
17.3 Wave soldering . . . 30
17.4 Reflow soldering . . . 31
18 Abbreviations . . . 32
19 Revision history . . . 33
20 Legal information . . . 34
20.1 Data sheet status . . . 34
20.2 Definitions . . . 34
20.3 Disclaimers . . . 34
20.4 Trademarks . . . 34
21 Contact information . . . 34
22 Contents . . . 35

Please be aware that important notices concerning this document and the product(s) described herein, have been included in section ‘Legal information’.

© NXP B.V. 2009. All rights reserved.

For more information, please visit: http://www.nxp.com
For sales office addresses, please send an email to: salesaddresses@nxp.com

Date of release: 16 July 2009

Document identifier: PCA9635_7

founded by PHILIPS
