# Supplementary material for: The Unfolding Space Glove: A Wearable Spatio-Visual to Haptic Sensory Substitution Device for Blind People
Source: Sensors (Basel). 2022 Feb 26;22(5):1859. doi: 10.3390/s22051859 (PMC8914703; doi:10.3390/s22051859)
Supplement: Supplementary file 1 [file sensors-22-01859-s001.zip › S1_GitHub-Release_unfolding-space-hardware-0.2.1-revised.zip!/unfolding-space-hardware-0.2.1-revised/pcb/datasheets/cm4-datasheet.pdf]

Raspberry Pi Compute Module 4

A Raspberry Pi for deeply embedded applications

Colophon

© 2020 Raspberry Pi (Trading) Ltd.

The documentation around the Raspberry Pi Compute Module 4 is licensed under a Creative Commons Attribution-NoDerivatives 4.0 International (CC BY-ND).

build-date: 2021-01-28
build-version: githash: 80b6c48-clean

Legal Disclaimer Notice

TECHNICAL AND RELIABILITY DATA FOR RASPBERRY PI PRODUCTS (INCLUDING DATASHEETS) AS MODIFIED FROM TIME TO TIME (“RESOURCES”) ARE PROVIDED BY RASPBERRY PI (TRADING) LTD (“RPTL) "AS IS" AND ANY EXPRESS OR IMPLIED WARRANTIES, INCLUDING, BUT NOT LIMITED TO, THE IMPLIED WARRANTIES OF MERCHANTABILITY AND FITNESS FOR A PARTICULAR PURPOSE ARE DISCLAIMED. TO THE MAXIMUM EXTENT PERMITTED BY APPLICABLE LAW IN NO EVENT SHALL RPTL BE LIABLE FOR ANY DIRECT, INDIRECT, INCIDENTAL, SPECIAL, EXEMPLARY, OR CONSEQUENTIAL DAMAGES (INCLUDING, BUT NOT LIMITED TO, PROCUREMENT OF SUBSTITUTE GOODS OR SERVICES; LOSS OF USE, DATA, OR PROFITS; OR BUSINESS INTERRUPTION) HOWEVER CAUSED AND ON ANY THEORY OF LIABILITY, WHETHER IN CONTRACT, STRICT LIABILITY, OR TORT (INCLUDING NEGLIGENCE OR OTHERWISE) ARISING IN ANY WAY OUT OF THE USE OF THE RESOURCES, EVEN IF ADVISED OF THE POSSIBILITY OF SUCH DAMAGE.

RPTL reserves the right to make any enhancements, improvements, corrections or any other modifications to the RESOURCES or any products described in them at any time and without further notice.

The RESOURCES are intended for skilled users with suitable levels of design knowledge. Users are solely responsible for their selection and use of the RESOURCES and any application of the products described in them. User agrees to indemnify and hold RPTL harmless against all liabilities, costs, damages or other losses arising out of their use of the RESOURCES.

RPTL grants users permission to use the RESOURCES solely in conjunction with the Raspberry Pi products. All other use of the RESOURCES is prohibited. No licence is granted to any other RPTL or other third party intellectual property right.

HIGH RISK ACTIVITIES. Raspberry Pi products are not designed, manufactured or intended for use in hazardous environments requiring fail safe performance, such as in the operation of nuclear facilities, aircraft navigation or communication systems, air traffic control, weapons systems or safety-critical applications (including life support systems and other medical devices), in which the failure of the products could lead directly to death, personal injury or severe physical or environmental damage (“High Risk Activities”). RPTL specifically disclaims any express or implied warranty of fitness for High Risk Activities and accepts no liability for use or inclusions of Raspberry Pi products in High Risk Activities.

Raspberry Pi products are provided subject to RPTL’s Standard Terms. RPTL’s provision of the RESOURCES does not expand or otherwise modify RPTL’s Standard Terms including but not limited to the disclaimers and warranties expressed in them.

Table of Contents

Colophon . . . 1
Legal Disclaimer Notice . . . 1
1. Introduction . . . 3
1.1. Introduction . . . 3
1.2. Features . . . 3
2. Interfaces . . . 5
2.1. Wireless . . . 5
2.1.1. `WL_nDisable` . . . 5
2.1.2. `BT_nDisable` . . . 5
2.2. Ethernet . . . 6
2.3. PCIe (Gen2 x1) . . . 6
2.4. USB 2.0 (Highspeed) . . . 7
2.5. GPIO . . . 7
2.5.1. Alternative Function Assignments . . . 8
2.6. Dual HDMI 2.0 . . . 10
2.7. CSI-2 (MIPI Serial Camera) . . . 10
2.8. DSI (MIPI Serial Display) . . . 11
2.9. I2C (SDA0 SCL0) . . . 11
2.10. I2C (ID_SD ID_SC) . . . 11
2.11. SDIO/eMMC (CM4Lite only) . . . 11
2.12. Analog IP0/IP1 . . . 12
2.13. `Global_EN` . . . 12
2.14. `RUN_PG` . . . 12
2.15. `nRPI_BOOT` . . . 12
2.16. `LED_nACT` . . . 12
2.17. `LED_nPWR` . . . 13
2.18. `EEPROM_nWP` . . . 13
3. Electrical and Mechanical . . . 14
3.1. Mechanical . . . 14
3.2. Thermal . . . 15
3.3. Electrical Specification . . . 15
4. Pin Out . . . 17
4.1. Differential Pairs . . . 23
4.1.1. 100Ω Differential pairs signal lengths . . . 23
4.1.2. 90Ω Differential Pairs signal lengths . . . 25
5. Power . . . 27
5.1. Power up sequencing . . . 27
5.2. Power down sequencing . . . 27
5.3. Power Consumption . . . 27
5.4. Regulator Outputs . . . 27
Appendix A: Troubleshooting . . . 28
Hardware Checklist . . . 28
Bootloader . . . 28
rpi-eeprom-update . . . 28
EEPROM Write protect . . . 28
Firmware . . . 29
Kernel . . . 29
Appendix B: Availability . . . 30
Support . . . 30
Ordering codes . . . 30
Packaging . . . 31

Chapter 1. Introduction

1.1. Introduction

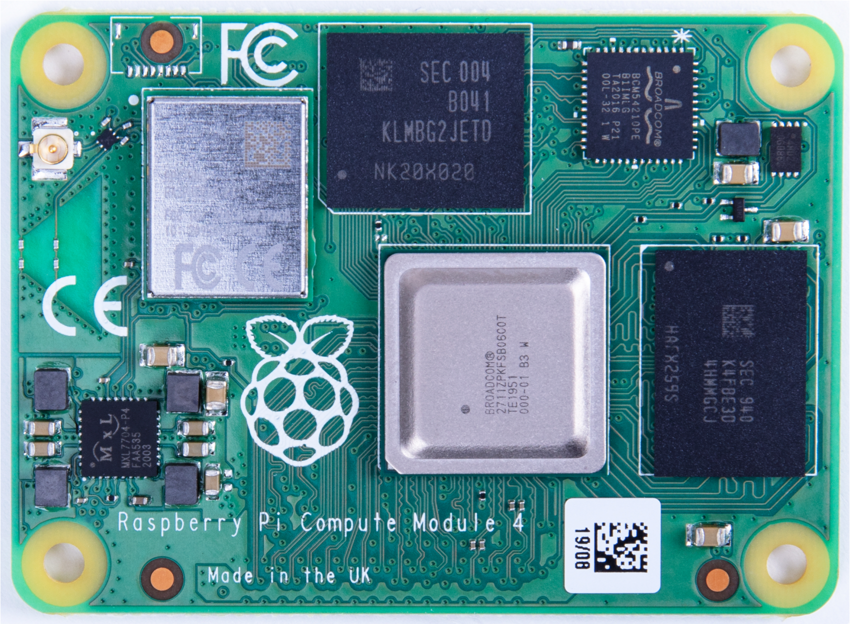

Figure 1. The Raspberry Pi Compute Module 4 (CM4).

The Raspberry Pi Compute Module 4 (CM4) is a System on Module (SoM) containing processor, memory, eMMC Flash and supporting power circuitry. These modules allow a designer to leverage the Raspberry Pi hardware and software stack in their own custom systems and form factors. In addition these modules have extra IO interfaces over and above what is available on the Raspberry Pi boards, opening up more options for the designer.

The design of the CM4 is loosely based on the Raspberry Pi 4, Model B, and for cost sensitive applications it can be supplied without the eMMC fitted; this version is called the Raspberry Pi Compute Module 4 Lite (CM4Lite).

While previous generations of the Compute Module have all shared the same DDR2-SODIMM-mechanically-compatible form factor, the new CM4 and CM4Lite are different. The electrical interface of the CM4 is via two 100-pin high density connectors, and the new physical form factor has a smaller footprint overall when the connectors are taken into account.

This change is due to the addition of new interfaces; an additional second HDMI, PCIe, and Ethernet. The addition of these new interfaces, especially PCIe, would not have been possible while preserving the previous form factor.

NOTE

> Unless otherwise stated, for this document CM4 also refers to CM4Lite.

1.2. Features

Key features of the CM4 are as follows:

- Broadcom BCM2711, Quad core Cortex-A72 (ARM v8) 64-bit SoC @ 1.5GHz
- Small Footprint 55mm × 40mm × 4.7mm module
  - 4 × M2.5 mounting holes
- H.265 (HEVC) (upto 4Kp60 decode), H.264 (upto 1080p60 decode, 1080p30 encode)

- OpenGL ES 3.0 graphics
- Options for 1GB, 2GB, 4GB or 8GB LPDDR4-3200 SDRAM with ECC (see Appendix B)
- Options for 0GB **(CM4Lite)**, 8GB, 16GB, or 32GB eMMC Flash memory (see Appendix B)
    - Peak eMMC bandwidth 100MBytes/s (four times faster than previous Compute modules)
- Option (see Appendix B) for certified radio module with:
    - 2.4 GHz, 5.0 GHz IEEE 802.11 b/g/n/ac wireless
    - Bluetooth 5.0, BLE
    - On board electronic switch to select between PCB trace or external antenna
- Gigabit Ethernet PHY supporting IEEE 1588
- 1 × PCIe 1-lane Host, Gen 2 (5Gbps)
- 1 × USB 2.0 port (highspeed)
- 28 × GPIO supporting either 1.8v or 3.3v signalling and peripheral options:
    - Up to 5 × UART
    - Up to 5 × I2C
    - Up to 5 × SPI
    - 1 × SDIO interface
    - 1 × DPI (Parallel RGB Display)
    - 1 × PCM
    - Up to 2× PWM channels
    - Up to 3× GPCLK outputs
- 2 × HDMI 2.0 ports (up to 4Kp60 supported)
- MIPI DSI:
    - 1 × 2-lane MIPI DSI display port
    - 1 × 4-lane MIPI DSI display port
- MIPI CSI-2:
    - 1 × 2-lane MIPI CSI camera port
    - 1 × 4-lane MIPI CSI camera port
- 1 × SDIO 2.0 **(CM4Lite)**
- Single +5v PSU input.

Chapter 2. Interfaces

2.1. Wireless

The CM4 can be supplied with an onboard wireless module based on the Broadcom BCM43455 supporting both,

- 2.4 GHz, 5.0 GHz IEEE 802.11 b/g/n/ac wireless
- Bluetooth 5.0, BLE.

These wireless interfaces can be individually enabled or disabled as required. For instance, in the case of a kiosk application, a service engineer could enable wireless operation and then disable it once finished.

The CM4 has an onboard antenna. If used it should be positioned in the product such that it is not surrounded by metal, including any ground plane (see Chapter 3 for further details). Alternatively there is a standard U.FL connector on the module, see Figure 1, so that an external antenna can be used.

Raspberry Pi has an antenna kit which is certified to be used with the CM4. If a different antenna is used then separate certification will be required.

WARNING

> Raspberry Pi Trading will not be able to assist with certification for third-party antennas.

The selection of internal or external antenna is done at boot time using the `config.txt` file, and can not be changed during operation. The `config.txt` options are `dtparam=ant1` to select the internal antenna, or `dtparam=ant2` for the external antenna.

2.1.1. `WL_nDisable`

This pin serves a number of functions;

1. It can be used to monitor the enable/disable state of wireless networking. A logic high means the wireless networking module is powered up.
2. When driven or tied low it prevents the wireles network module from powering up. This is useful to reduce power consumption or in applications where it is required to physically ensure the wireless networking is disabled. If the interface is enabled after being disabled, the wireless interface driver needs reinitalised.

NOTE

> On CM4 modules without wireless, this pin is reserved.

2.1.2. `BT_nDisable`

This pin serves a number of functions;

1. It can be used to monitor the enable/disable state of Bluetooth. A logic high means the Bluetooth module is powered up.
2. When driven, or tied low, it prevents the Bluetooth module from powering up. This is useful to reduce power consumption, or in applications where it is required to physically ensure the Bluetooth is disabled. If the interface is enabled after being disabled, the Bluetooth interface driver needs reinitalised.

NOTE

On CM4 modules without wireless, this pin is reserved.

2.2. Ethernet

The CM4 has an onboard Gigabit Ethernet PHY — the Broadcom BCM54210PE — some of the major features of this PHY include;

- IEEE 1588-2008 compliant
- Detection and correction of swapped pairs
- MDI crossover, pair skew and pair polarity correction

A standard 1:1 RJ45 MagJack is all that is necessary to provide an Ethernet connection to the CM4. Typical wiring of a MagJack supporting POE, and with added ESD protection, can be seen in Figure 2.

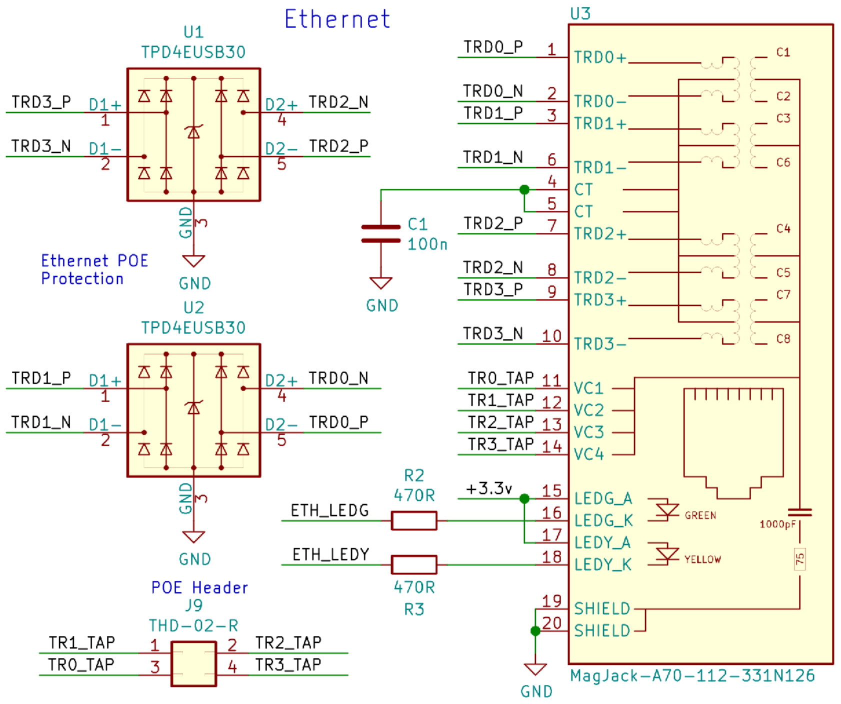

Figure 2. Ethernet schematic interface for the Raspberry Pi Compute Module 4 supporting POE, and with added ESD protection.

The differential Ethernet signals should be routed as 100Ω differential pairs, with suitable clearances. Length matching between pairs should be better than 50mm, so in the typical case no length matching is required. However the signals within a pair need to be length matched, ideally to better than 0.15mm.

The PHY also supports up to 3 LEDs to give user status feedback, these are low active. These LEDs can have a range of functions, and you should consult your OS driver to see which functions are supported by your driver.

The PHY also provides `SYNC_IN` and `SYNC_OUT` at 1.8v signalling to support IEEE 1588-2008.

2.3. PCIe (Gen2 x1)

The CM4 has an internal PCIe 2.0 x1 host controller. While on the Raspberry Pi 4, Model B this has been connected to a USB 3 host controller (using the Via Labs VLI805), on the CM4 the product designer is free to choose how the interface is used.

WARNING

You should ensure that there is a suitable OS driver for any host controller that is chosen before proceeding to a prototype.

NOTE

The onboard PCIe Host controller doesn't support 64bit accesses from the ARM, they must be split up into two 32 bit accesses.

Connecting a PCIe device follows the standard PCIe convention. The CM4 has onboard AC coupling capacitors for `CLK` and `PCIe_TX` signals. However the `PCIe_RX` signals need external coupling capacitors close to the driving source (the device `TX`), if you are using an external PCIe/NVMe cards these capacitors will be onboard. The PCIe conversion is that if you are wiring directly to an IC then the TX and RX pairs need to be swapped (ie. TX->RX , Rx->TX). If you are wiring to a connector then this is typically labeled from the host post of view and so TX RX swaps aren't required. Additionally the `PCIe_CLK_nREQ` must be connected to ensure the CM4 produces a clock signal, and the `PCIe_nRST` should also be connected to ensure the device is correctly reset when required.

The differential PCIe signals should be routed as 90Ω differential pairs, with suitable clearances. There is no need to match the lengths between pairs, only the signals within a Pair need to be length matched ideally to better than 0.1mm.

TIP

5.10 kernels and newer have had support for MSI-X added. There is a limit of upto 32 IRQs available. If the device has problems with interrupts then adding `pci=nomsi` to `cmdline.txt` (and rebooting) often fixes the issue.

2.4. USB 2.0 (Highspeed)

The USB 2.0 interface supports up to 480MBps signalling. The differential pair should be routed as a 90Ω differential pair. The P N signals should ideally be matched to 0.15mm

TIP

The USB interface is disabled to save power by default on the CM4 . To enable it you need to add `dtoverlay=dwc2,dr_mode=host` to the config.txt file

NOTE

The port is capable of being used as a true USB On-The-Go (OTG) port. While there is no official documentation, some users have had success making this work. The `USB_OTG` pin is used to select between USB host and device that is typically wired to the ID pin of a Micro usb connector. To use this functionality it must be enabled in the OS that is used. If using either as a fixed slave or fixed master, please tie the USB `OTGID` pin to ground

2.5. GPIO

There are 28 pins available for general purpose I/O (GPIO), which correspond to the GPIO pins on the Raspberry Pi 4, Model B 40-pin header. These pins have access to internal peripherals; I2C, PWM, SPI, and UART. The BCM2711 ARM Peripherals book describes these features in detail, and the multiplexing options available. The drive strength and slew rate should ideally be set as low as possible to reduce any EMC issues. GPIO2 and GPIO3 have 1.8K pull up resistors.

The BCM2711 GPIO bank is powered by `GPIO_VREF`, this can either be connected to the +1.8v from the CM4 for 1.8v signalling GPIO, or +3.3v from the CM4 for +3.3v signalling. You should keep the load on the 28 GPIO pins to below 50mA

in total. `GPIO_VREF` must be powered for the CM4 to startup correctly

2.5.1. Alternative Function Assignments

Up to 6 alternate functions are available. The BCM2711 ARM Peripherals book describes these features in detail. The table below gives a quick overview.

Table 1. GPIO Pins Alternative Function Assignment

GPIO	Pull	ALT0	ALT1	ALT2	ALT3	ALT4	ALT5
GPIO0	High	SDA0	SA5	PCLK	SPI3_CE0_N	TXD2	SDA6
GPIO1	High	SCL0	SA4	DE	SPI3_MISO	RXD2	SCL6
GPIO2	High	SDA1	SA3	LCD_VSYNC	SPI3_MOSI	CTS2	SDA3
GPIO3	High	SCL1	SA2	LCD_HSYNC	SPI3_SCLK	RTS2	SCL3
GPIO4	High	GPCLK0	SA1	DPI_D0	SPI4_CE0_N	TXD3	SDA3
GPIO5	High	GPCLK1	SA0	DPI_D1	SPI4_MISO	RXD3	SCL3
GPIO6	High	GPCLK2	SOE_N / SE	DPI_D2	SPI4_MOSI	CTS3	SDA4
GPIO7	High	SPI0_CE1_N	SWE_N / SRW_N	DPI_D3	SPI4_SCLK	RTS3	SCL4
GPIO8	High	SPI0_CE0_N	SD0	DPI_D4	BSCSL / CE_N	TXD4	SDA4
GPIO9	Low	SPI0_MISO	SD1	DPI_D5	BSCSL / MISO	RXD4	SCL4
GPIO10	Low	SPI0_MOSI	SD2	DPI_D6	BSCSL SDA / MOSI	CTS4	SDA5
GPIO11	Low	SPI0_SCLK	SD3	DPI_D7	BSCSL SCL / SCLK	RTS4	SCL5
GPIO12	Low	PWM0_0	SD4	DPI_D8	SPI5_CE0_N	TXD5	SDA5
GPIO13	Low	PWM0_1	SD5	DPI_D9	SPI5_MISO	RXD5	SCL5
GPIO14	Low	TXD0	SD6	DPI_D10	SPI5_MOSI	CTS5	TXD1
GPIO15	Low	RXD0	SD7	DPI_D11	SPI5_SCLK	RTS5	RXD1
GPIO16	Low	<reserved>	SD8	DPI_D12	CTS0	SPI1_CE2_N	CTS1
GPIO17	Low	<reserved>	SD9	DPI_D13	RTS0	SPI1_CE1_N	RTS1
GPIO18	Low	PCM_CLK	SD10	DPI_D14	SPI6_CE0_N	SPI1_CE0_N	PWM0_0
GPIO19	Low	PCM_FS	SD11	DPI_D15	SPI6_MISO	SPI1_MISO	PWM0_1
GPIO20	Low	PCM_DIN	SD12	DPI_D16	SPI6_MOSI	SPI1_MOSI	GPCLK0
GPIO21	Low	PCM_DOUT	SD13	DPI_D17	SPI6_SCLK	SPI1_SCLK	GPCLK1
GPIO22	Low	SD0_CLK	SD14	DPI_D18	SD1_CLK	ARM_TRST	SDA6
GPIO23	Low	SD0_CMD	SD15	DPI_D19	SD1_CMD	ARM_RTCK	SCL6
GPIO24	Low	SD0_DAT0	SD16	DPI_D20	SD1_DAT0	ARM_TDO	SPI3_CE1_N
GPIO25	Low	SD0_DAT1	SD17	DPI_D21	SD1_DAT1	ARM_TCK	SPI4_CE1_N
GPIO26	Low	SD0_DAT2	<reserved>	DPI_D22	SD1_DAT2	ARM_TDI	SPI5_CE1_N
GPIO27	Low	SD0_DAT3	<reserved>	DPI_D23	SD1_DAT3	ARM_TMS	SPI6_CE1_N
GPIO44	-	GPCLK1	SDA0	SDA1	<reserved>	SPI0_CE1_N	SD_CARD_VOLT

GPIO	Pull	ALT0	ALT1	ALT2	ALT3	ALT4	ALT5
GPIO45	-	PWM0_1	SCL0	SCL1	<reserved>	SPI0_CE2_N	SD_CARD_PWR0

Special function legend:

Table 2. GPIO Pins Alternative Function Legend

Name	Function
SDA0	BSC master 0 data line[a]
SCL0	BSC master 0 clock line
SDAx	BSC master 1,3,4,5,6 data line[b]
SCLx	BSC master 1,3,4,5,6 clock line
GPCLKx	General purpose Clock 0,1,2
SPIx_CE2_N	SPI 0,3,4,5,6 Chip select 2
SPIx_CE1_N	SPI 0,3,4,5,6 Chip select 1
SPIx_CE0_N	SPI 0,3,4,5,6 Chip select 0
SPIx_MISO	SPI 0,3,4,5,6 MISO
SPIx_MOSI	SPI 0,3,4,5,6 MOSI
SPIx_SCLK	SPI 0,3,4,5,6 Serial clock
PWMx_0	PWM 0,1 channel 0
PWMx_1	PWM 0,1 channel 1
TXDx	UART 0,2,3,4,5 Transmit Data
RXDx	UART 0,2,3,4,5 Receive Data
CTSx	UART 0,2,3,4,5 Clear To Send
RTSx	UART 0,2,3,4,5 Request To Send
PCM_CLK	PCM clock
PCM_FS	PCM Frame Sync
PCM_DIN	PCM Data in
PCM_DOUT	PCM data out
SAx	Secondary mem Address bus
SOE_N / SE	Secondary mem. Controls
SWE_N / SRW_N	Secondary mem. Controls
SDx	Secondary mem. data bus
BSCSL SDA / MOSI	BSC slave Data, SPI slave MOSI
BSCSL SCL / SCLK	BSC slave Clock, SPI slave clock
BSCSL - / MISO	BSC <not used>, SPI MISO
BSCSL - / CE_N	BSC <not used>, SPI CSn
SPI1_CE2_N	SPI 1 Chip select 2 [c]
SPI1_CE1_N	SPI 1 Chip select 1
SPI1_CE0_N	SPI 1 Chip select 0

Name	Function
SPI1_MISO	SPI 1 MISO
SPI1_MOSI	SPI 1 MOSI
SPI1_SCLK	SPI 1 Serial clock
TXD1	UART 1 Transmit Data
RXD1	UART 1 Receive Data
CTS1	UART 1 Clear To Send
RTS1	UART 1 Request To Send
ARM_TRST	ARM JTAG reset
ARM_RTCK	ARM JTAG return clock
ARM_TDO	ARM JTAG Data out
ARM_TCK	ARM JTAG Clock
ARM_TDI	ARM JTAG Data in
ARM_TMS	ARM JTAG Mode select
PCLK	Display Parallel Interface
DE	Display Parallel Interface
LCD_VSYNC	Display Parallel Interface
LCD_HSYNC	Display Parallel Interface
DPI_Dx	Display Parallel Interface

[a] The Broadcom Serial Control bus is a proprietary bus compliant with the Philips® I2C bus/interface.
[b] BSC master 2 & 7 are not user-accessible.
[c] SPI 2 is not user-accessible.

2.6. Dual HDMI 2.0

The CM4 supports two HDMI 2.0 interfaces each one capable of driving 4K images. If both HDMI outputs are used then each can be driven upto 4Kp30, however if only HDMI0 interface is being used then images up to 4Kp60 are possible.

HDMI signals should be routed as 100Ω differential pairs, each signal within a pair should ideally be matched to better than 0.15mm. Pairs don't typically need any extra matching as they only have to be matched to 25mm.

CEC is also supported, an internal 27K pullup resistor is included in the CM4.

Basic onboard ESD protection is provided for the I2C EDID signals and the CEC signals, internal pullup and down resistors are also provided. On the {rpi4} the HDMI signals don't have any extra ESD protection , depending on the application extra ESD protection maybe required.

2.7. CSI-2 (MIPI Serial Camera)

The CM4 supports two camera ports; `CAM0` (2 lanes) and `CAM1` (4 lanes). CSI signals should be routed as 100Ω differential pairs, each signal within a pair should ideally be matched to better than 0.15mm.

The documentation around the CSI interface can be found on the Raspberry Pi website while Linux kernel drivers can be found on Github.

NOTE

Camera sensors supported by the official Raspberry Pi firmware are; the OmniVision OV5647, Sony IMX219 and Sony IMX477, no security device is required on Compute Module devices to use these camera sensors.

2.8. DSI (MIPI Serial Display)

The CM4 supports two display ports; `DISP0` (2 lanes) and `DISP1` (4 lanes). Each lane supports a maximum of data rate per lane of 1Gbit/s.

Although Linux kernel drivers are available, the DSI interface is not currently documented. Only DSI displays supported by the official Raspberry Pi firmware are supported, DSI signals should be routed as 100Ω differential pairs, each signal within a pair should ideally be matched to better than 0.15mm.

NOTE

While only official DSI displays are supported, other displays can be added using the parallel DPI interface which is available as a GPIO alternate function. The CM4 supports up to 3 displays of any type (HDMI, DSI, DPI) at any one time.

2.9. I2C (SDA0 SCL0)

This internal I2C bus is normally allocated to the CSI1 and DSI1 as these devices are controlled by the firmware. It can be used as a general I2C bus if the CSI1 ad DSI1 interfaces aren't being used or are being controlled by the firmware. For example libcamera runs on the ARM and doesn't use the firmware so in this case you may use CSI1 and this I2C bus. SDA0 is connected to GPIO44 on the BCM2711 and SCL0 is connected to GPIO45

2.10. I2C (ID_SD ID_SC)

This I2C bus is normally used for identifying HATs and controlling CSI0 and DSI0 devices. If the firmware isn't using the I2C bus e.g. CSI0 and DSI0 aren't being used then these pins may be used as GPIO 0 and GPIO 1 if required.

Note: If these pins are used as GPIO pins then to prevent the firmware from checking to see if there is a HAT EEPROM available add `force_eeprom_read=0` to the config.txt file.

2.11. SDIO/eMMC (CM4Lite only)

If the CM4Lite is used, which does not have on-board eMMC, then the eMMC signals are available on the connector so that an external eMMC or SDCARD can be used.

The `SD_PWR_ON` signal is used to enable an external power switch to turn on power to the SDCARD, for eMMC it typically isn't used. If booting from SDCARD is required then a pullup resistor must also be fitted to default the power to be on. When `SD_VDD_override` is high, this signal is used to force 1.8v signalling on the SDIO interface. Typically this is used with eMMC memory.

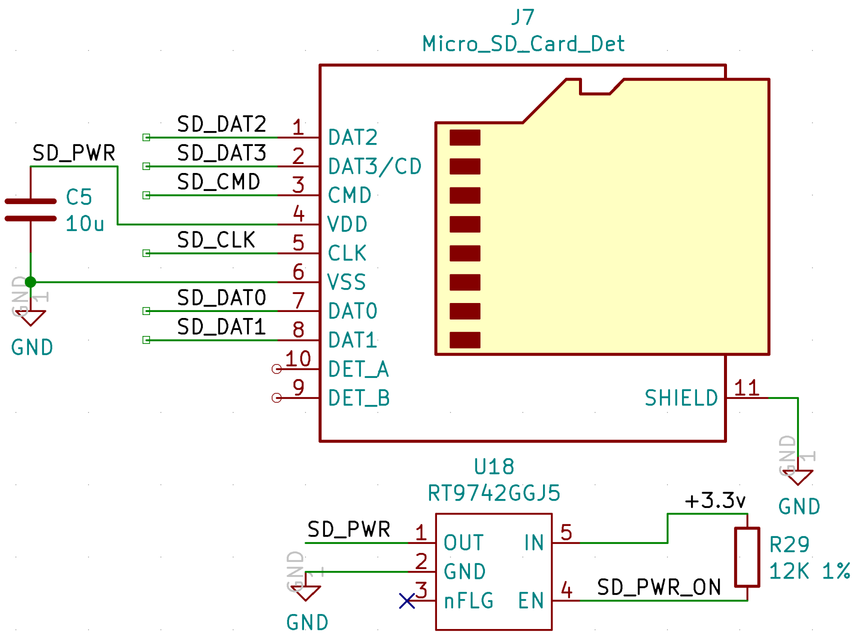

Figure 3. CM4Lite SDCARD interface.

2.12. Analog IP0/IP1

These are the two spare inputs on the MXL7704. The MXL7704 datasheet should be consulted if these pins are to be used. Onboard filtering is provided by a 100nF capacitor to ground for each signal. On the Raspberry Pi 4, Model B these are connected to the USB C connector `CC1` and `CC2` pins.

2.13. `Global_EN`

Pulling this pin low puts the CM4 in the lowest possible power down state. After software shutdown `Global_EN` needs to be pulled low for > 1ms to restart the power system on the CM4.

TIP

> It is recommended to only pull this pin low once the OS has shutdown.

2.14. `RUN_PG`

This pin when high signals that the CM4 has started. Driving this pin low resets the module, this should be done with caution as if files on a filesystem are open they will not be closed.

2.15. `nRPI_BOOT`

During boot if this pin is low booting from eMMC will be stopped and booting will be transferred to rpi boot which is via USB.

2.16. `LED_nACT`

This pin is designed to drive an LED to replicate the green LED on the Raspberry Pi 4, Model B. Under Linux this pin will flash to signify eMMC access, while if there is an error during booting this LED will flash error patterns which can be

decoded using the look up table on the Raspberry Pi website.

2.17. LED_nPWR

This pin needs to be buffered to drive an LED. The signal is designed to replicate the red power LED on the Raspberry Pi 4, Model B.

2.18. EEPROM_nWP

It is recommended that final products pull this pin low to prevent the end users changing the contents of the on board EEPROM. See the Raspberry Pi 4, Model B documentation for instructions on the software settings required to support EEPROM Write protection .

Chapter 3. Electrical and Mechanical

3.1. Mechanical

The CM4 is a compact 40 × 55mm module. The Module is 4.7mm deep, but when connected the height will be 5.078 or 6.578 mm depending on the stacking height chosen.

1. 4 × M2.5 Mounting holes (inset 3.5mm from module edge)
2. PCB thickness 1.2mm ± 10%
3. BCM2711 SOC height including solder balls 2.378 ± 0.11mm
4. Stacking height either:
    a. 1.5mm with mating connector (clearance under CM4 0mm) : DF40C-100DS-0.4v
    b. 3.0mm with mating connector (clearance under CM4 1.5mm): DF40HC(3.0)-100DS-0.4v

If the on board wireless antenna is used (see Section 2.1) it must be orientated towards the edge of the plastic enclosure and any close by metal must have cut outs or the wireless performance will be degraded. It is suggested that there is at least 10mm clearance around the PCB antenna, but the designer must check the performance.

Figure 4. Mechanical specification of the Raspberry Pi Compute Module 4

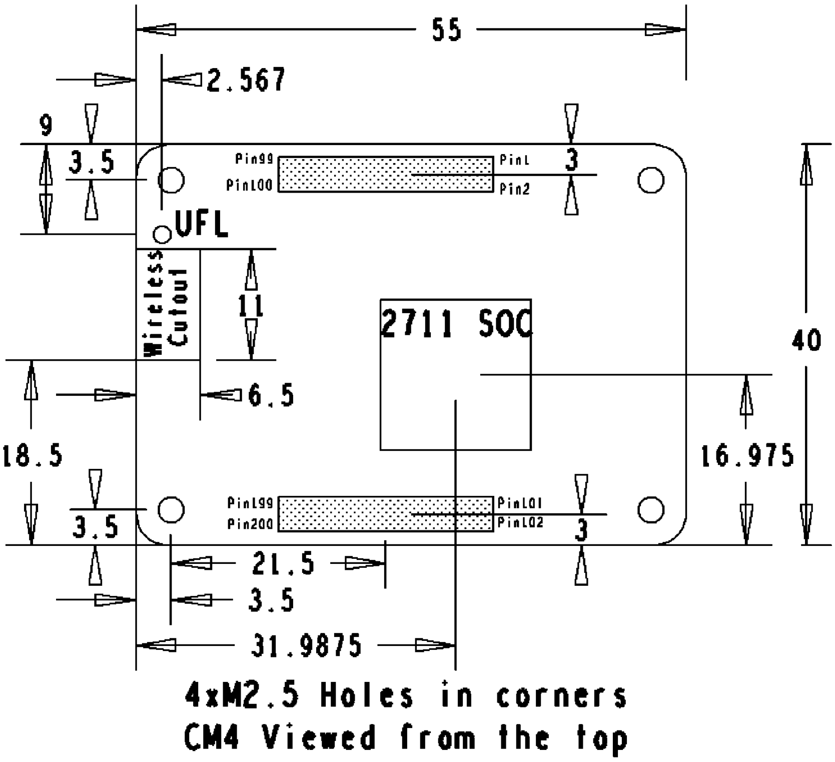

There must not be any metal, including ground planes, under the antenna. The ground plane cutout must be a minimum of 6.5mm × 11mm, but ideally at least 8mm × 15mm. If these requirements can't be met wireless performance may be degraded, especially in the 2.4GHz spectrum. It is recommended that the external antenna is used where possible.

NOTE

> The location and arrangement of components on the Compute Module may change slightly over time due to revisions for cost and manufacturing considerations; however the maximum component heights and PCB thickness will be kept as specified.

A step file of the CM4 is available as part of the CM4 design data package, this is for guidance only and is subject to changes over time due to revisions.

3.2. Thermal

The CM4 dissipates less power than the Raspberry Pi 4, Model B. The CM4 also contains less metal in the PCB and connectors and so it has less passive heat sinking than the Raspberry Pi 4, Model B. Therefore despite it consuming less power it may run warmer than the Raspberry Pi 4, Model B.

The BCM2711 will reduce the clock rate to try and keep its internal temperature below 85°C. So in high ambient temperatures it is possible that the clock will also be automatically throttled back. If the BCM2711 is unable to lower its internal clocks enough to bring the temperature down its case temperature will rise above 85°C. It is important that thermal solution chosen keeps the ambient temperature for the other silcon devices on the CM4 within the operating temperature range.

Operating temperature range: -20°C - +85°C Non-condensing. NB Optimal RF Wireless performance is between -20°C and +75°C .

3.3. Electrical Specification

WARNING

> Stresses above those listed in Table 3 may cause permanent damage to the device. This is a stress rating only; functional operation of the device under these or any other conditions above those listed in the operational sections of this specification is not implied. Exposure to absolute maximum rating conditions for extended periods may affect device reliability.

Table 3. Absolute maximum ratings

Symbol	Parameter	Minimum	Maximum	Unit
V_{IN}	5V Input Voltage	-0.5	6.0	V
V_{GPIO_ref}	GPIO Voltage	-0.5	3.6	V
V_{gpio}	GPIO Input voltage	-0.5	V_{GPIO_ref} + 0.5v	V

Please note that V_{ref} is the GPIO bank voltage which must be tied to either 3.3V or 1.8v rail.

Table 4. DC characteristics

Symbol	Parameter	Conditions	Minimum	Typical	Maximum	Unit
$V_{IL(gpio)}$	Input low voltage	V_{ref} = 3.3V	0	-	0.8	V
$V_{IH(gpio)}$	Input high voltage	V_{ref} = 3.3V	2.0	-	V_{GPIO_ref}	V
$V_{IL(gpio)}$	Input low voltage	V_{ref} = 1.8V	0	-	0.35	V
$V_{IH(gpio)}$	Input high voltage	V_{ref} = 1.8V	0.65	-	V_{GPIO_ref}	V

$I_{IL(gpio)}$	Input leakage current	-	-	-	10	μA
$V_{OL(gpio)}$	Output low voltage	-	-	-	0.4	V
$V_{OH(gpio)}$	Output high voltage	-	V_{GPIO_ref}-0.4	-	-	V
$I_{O(gpio)}$	Output current	1mA	0.87	1.3	-	mA
$I_{O(gpio)}$	Output current	2mA	1.75	2.6	-	mA
$I_{O(gpio)}$	Output current	3mA	2.63	3.9	-	mA
$I_{O(gpio)}$	Output current	4mA **Default**	3.5	5.3	-	mA
$I_{O(gpio)}$	Output current	5mA	4.39	6.6	-	mA
$I_{O(gpio)}$	Output current	6mA	5.27	7.9	-	mA
$I_{O(gpio)}$	Output current	7mA	6.15	9.2	-	mA
$I_{O(gpio)}$	Output current	8mA	7.02	10.5	-	mA
$R_{PU(gpio)}$	Pullup resistor	V_{ref} = 3.3V	33	47	73	kΩ
$R_{PD(gpio)}$	Pulldown resistor	V_{ref} = 3.3V	33	47	73	kΩ
$R_{PU(gpio)}$	Pullup resistor	V_{ref} = 1.8V	18	47	73	kΩ
$R_{PD(gpio)}$	Pulldown resistor	V_{ref} = 1.8V	18	47	73	kΩ

Refer to interface specifications (see Chapter 2) for electrical details of other interfaces.

Table 5. Power Consumption

**Symbol**	**Parameter**	**Conditions**	**Minimum**	**Typical**	**Maximum**	**Unit**
$I_{shutdown}$	Shutdown current	`GLOBAL_EN` = 0v	-	15	-	μA
$I_{shutdown}$	Shutdown current	`GLOBAL_EN` >2v	-	8	-	mA
I_{idle}	Idle current	`GLOBAL_EN` >2v	-	400	-	mA
I_{load}	Operation current	`GLOBAL_EN` >2v	-	1400	-	mA

NOTE

The figures in Table 5 greatly depend on the end application.

Chapter 4. Pin Out

Table 6. Pin out for the Raspberry Pi Compute Module 4

Pin	Signal	Description
1	GND	Ground (0V)
2	GND	Ground (0V)
3	Ethernet_Pair3_P	Ethernet Pair 3 Positive (connect to Transformer or MagJack)
4	Ethernet_Pair1_P	Ethernet Pair 1 Positive (connect to Transformer or MagJack)
5	Ethernet_Pair3_N	Ethernet Pair 3 Negative (connect to Transformer or MagJack)
6	Ethernet_Pair1_N	Ethernet Pair 1 Negative (connect to Transformer or MagJack)
7	GND	Ground (0V)
8	GND	Ground (0V)
9	Ethernet_Pair2_N	Ethernet Pair 2 Negative (connect to Transformer or MagJack)
10	Ethernet_Pair0_N	Ethernet Pair 0 Negative (connect to Transformer or MagJack)
11	Ethernet_Pair2_P	Ethernet Pair 2 Positive (connect to Transformer or MagJack)
12	Ethernet_Pair0_P	Ethernet Pair 0 Positive (connect to Transformer or MagJack)
13	GND	Ground (0V)
14	GND	Ground (0V)
15	Ethernet_nLED3	Low Active Ethernet Activity indicator (3.3V signal) Typically a Green LED is connected to this pin: I_{OL} = 8mA @ $V_{OL} < 0.4V$
16	Ethernet_SYNC_IN	IEEE1588 SYNC Input pin (1.8V signal : I_{OL} = 8mA @ $V_{OL} < 0.4V$)
17	Ethernet_nLED2	Low Active Ethernet speed indicator (3.3V signal) Typically a Yellow LED is connected to this pin. A low State indicates the 1Gbit or 100Mbit Link : I_{OL} = 8mA @ $V_{OL} < 0.4V$
18	Ethernet_SYNC_OUT	IEEE1588 SYNC Output pin (1.8V signal : I_{OL} = 8mA @ $V_{OL} < 0.4V$)
19	Ethernet_nLED1	Low Active Ethernet speed indicator (3.3V signal) Typically a Yellow LED is connected to this pin. A low State indicates the 1Gbit or 10Mbit Link : I_{OL} = 8mA @ $V_{OL} < 0.4V$
20	EEPROM_nWP	Leaving floating NB internally pulled up to `CM4_3.3V` via 100K (V_{IL} <0.8V) but can be grounded to prevent writing to the on board EEPROM which stores the bootcode
21	Pi_nLED_Activity	Low Active Pi Activity LED. 20mA Max 5V tolerant (V_{OL}<0.4V). (this is the signal that drives the Green LED on the Raspberry Pi 4, Model B)
22	GND	Ground (0V)
23	GND	Ground (0V)
24	GPIO26	GPIO Typically a 3.3V signal but can be a 1.8V signal by connecting GPIO_Vref to 1.8V
25	GPIO21	GPIO Typically a 3.3V signal but can be a 1.8V signal by connecting GPIO_Vref to 1.8V
26	GPIO19	GPIO Typically a 3.3V signal but can be a 1.8V signal by connecting GPIO_Vref to 1.8V
27	GPIO20	GPIO Typically a 3.3V signal but can be a 1.8V signal by connecting GPIO_Vref to 1.8V
28	GPIO13	GPIO Typically a 3.3V signal but can be a 1.8V signal by connecting GPIO_Vref to 1.8V
29	GPIO16	GPIO Typically a 3.3V signal but can be a 1.8V signal by connecting GPIO_Vref to 1.8V
30	GPIO6	GPIO Typically a 3.3V signal but can be a 1.8V signal by connecting GPIO_Vref to 1.8V

31	GPIO12	GPIO Typically a 3.3V signal but can be a 1.8V signal by connecting GPIO_Vref to 1.8V
32	GND	Ground (0V)
33	GND	Ground (0V)
34	GPIO5	GPIO Typically a 3.3V signal but can be a 1.8V signal by connecting GPIO_Vref to 1.8V
35	ID_SC	(BCM2711 GPIO 1) GPIO Typically a 3.3V signal but can be a 1.8V signal by connecting GPIO_Vref to 1.8V
36	ID_SD	(BCM2711 GPIO 0) GPIO Typically a 3.3V signal but can be a 1.8V signal by connecting GPIO_Vref to 1.8V
37	GPIO7	GPIO Typically a 3.3V signal but can be a 1.8V signal by connecting GPIO_Vref to 1.8V
38	GPIO11	GPIO Typically a 3.3V signal but can be a 1.8V signal by connecting GPIO_Vref to 1.8V
39	GPIO8	GPIO Typically a 3.3V signal but can be a 1.8V signal by connecting GPIO_Vref to 1.8V
40	GPIO9	GPIO Typically a 3.3V signal but can be a 1.8V signal by connecting GPIO_Vref to 1.8V
41	GPIO25	GPIO Typically a 3.3V signal but can be a 1.8V signal by connecting GPIO_Vref to 1.8V
42	GND	Ground (0V)
43	GND	Ground (0V)
44	GPIO10	GPIO Typically a 3.3V signal but can be a 1.8V signal by connecting GPIO_Vref to 1.8V
45	GPIO24	GPIO Typically a 3.3V signal but can be a 1.8V signal by connecting GPIO_Vref to 1.8V
46	GPIO22	GPIO Typically a 3.3V signal but can be a 1.8V signal by connecting GPIO_Vref to 1.8V
47	GPIO23	GPIO Typically a 3.3V signal but can be a 1.8V signal by connecting GPIO_Vref to 1.8V
48	GPIO27	GPIO Typically a 3.3V signal but can be a 1.8V signal by connecting GPIO_Vref to 1.8V
49	GPIO18	GPIO Typically a 3.3V signal but can be a 1.8V signal by connecting GPIO_Vref to 1.8V
50	GPIO17	GPIO Typically a 3.3V signal but can be a 1.8V signal by connecting GPIO_Vref to 1.8V
51	GPIO15	GPIO Typically a 3.3V signal but can be a 1.8V signal by connecting GPIO_Vref to 1.8V
52	GND	Ground (0V)
53	GND	Ground (0V)
54	GPIO4	GPIO Typically a 3.3V signal but can be a 1.8V signal by connecting GPIO_Vref to 1.8V
55	GPIO14	GPIO Typically a 3.3V signal but can be a 1.8V signal by connecting GPIO_Vref to 1.8V
56	GPIO3	GPIO Typically a 3.3V signal but can be a 1.8V signal by connecting GPIO_Vref to 1.8V. Internal 1.8K pull up to GPIO_Vref
57	SD_CLK	SDCARD Clock signal (only available on CM4Lite)
58	GPIO2	GPIO Typically a 3.3V signal but can be a 1.8V signal by connecting GPIO_Vref to 1.8V. Internal 1.8K pull up to GPIO_Vref
59	GND	Ground (0V)
60	GND	Ground (0V)
61	SD_DAT3	SDCARD/eMMC Data3 signal (only available on CM4Lite)
62	SD_CMD	SDCARD/eMMC Command signal (only available on CM4Lite)
63	SD_DAT0	SDCARD/eMMC Data0 signal (only available on CM4Lite)
64	SD_DAT5	SDCARD/eMMC Data5 signal (only available on CM4Lite)

65	GND	Ground (0V)
66	GND	Ground (0V)
67	SD_DAT1	SDCARD/eMMC Data1 signal (only available on CM4Lite)
68	SD_DAT4	SDCARD/eMMC Data4 signal (only available on CM4Lite)
69	SD_DAT2	SDCARD/eMMC Data2 signal (only available on CM4Lite)
70	SD_DAT7	SDCARD/eMMC Data7 signal (only available on CM4Lite)
71	GND	Ground (0V)
72	SD_DAT6	SDCARD/eMMC Data6 signal (only available on CM4Lite)
73	SD_VDD_Override	Force SDCARD/eMMC interface to 1.8V signalling if set to 3.3V, otherwise leave unconnected. Typically only used if external eMMC is connected
74	GND	Ground (0V)
75	SD_PWR_ON	Output to Power switch for the SDCARD. The CM4 sets this pin High (3.3V) to signal that Power to the SDCARD should be turned on. If booting from the SDCARD is required then a pullup should also be fitted so the power defaults to on. (only available on CM4Lite)
76	Reserved	Do not Connect anything to this pin.
77	+5V (Input)	4.75V-5.25V Main power input
78	GPIO_VREF	Must be connected to `CM4_3.3V` (pins 84 and 86) for 3.3V GPIO or `CM4_1.8V` (pins 88 and 90) for 1.8V GPIO. This pin cannot be floating or connected to ground
79	+5V (Input)	4.75V-5.25V Main power input
80	SCL0	IIC Clock pin (BCM2711 GPIO45) Typically used for Camera and Display Internal 1.8K pull up to `CM4_3.3V`
81	+5V (Input)	4.75V-5.25V Main power input
82	SDA0	IIC Data pin (BCM2711 GPIO44) Typically used for Camera and Display Internal 1.8K pull up to `CM4_3.3V`
83	+5V (Input)	4.75V-5.25V Main power input
84	CM4_3.3V (Output)	3.3V +/-2.5% Power Output max 300mA per pin for a total of 600mA. This will be powered down during power off or GLOBAL_EN being set low
85	+5V (Input)	4.75V-5.25V Main power input
86	CM4_3.3V (Output)	3.3V +/-2.5% Power Output max 300mA per pin for a total of 600mA. This will be powered down during power off or GLOBAL_EN being set low
87	+5V (Input)	4.75V-5.25V Main power input
88	CM4_1.8V (Output)	1.8V +/-2.5% Power Output max 300mA per pin for a total of 600mA. This will be powered down during power off or GLOBAL_EN being set low
89	WL_nDisable	Can be left floating if driven low the wireless interface will be disabled. Internal pulled up via 1.8K to `CM4_3.3V`
90	CM4_1.8V (Output)	1.8V +/-2.5% Power Output max 300mA per pin for a total of 600mA. This will be powered down during power off or GLOBAL_EN being set low
91	BT_nDisable	Can be left floating if driven low the Bluetooth interface will be disabled. Internal pulled up via 1.8K to `CM4_3.3V`
92	RUN_PG	Bidirectional pin. Can be driven low (via a 220R resistor) to Reset the CM4 CPU. As an Output a high signals Power Good and CPU running. Internally pulled up to +3.3V via 10K

93	nRPIBOOT	A low on this pin forces booting from an RPI server (e.g. PC or a Raspberry Pi) if not used leave floating. Internally pulled up via 10K to +3.3V
94	AnalogIP1	Analogue input of the MXL7704. Typically connected to CC pin of Type C power connector
95	PI_LED_nPWR	Low active Output to drive Power On LED. This signal needs to be buffered.
96	AnalogIP0	Analogue input of the MXL7704. Typically connected to CC pin of Type C power connector
97	Camera_GPIO	Typically used to Shutdown the camera to reduce power. Reassigning this pin to another function isn't recommended. `CM4_3.3V` signalling
98	GND	Ground (0V)
99	GLOBAL_EN	Input. Drive low to power off CM4. Internally pulled up with a 100K to +5V
100	nEXTRST	Output Driven low during reset Driven high (`CM4_3.3V`) once CM4 CPU has started to boot
101	USB_OTG_ID	Input (3.3V signal) USB OTG Pin. Internal pulled up. When grounded the CM4 becomes a USB host but the correct OS driver also needs to be used
102	PCIe_CLK_nREQ	Input (3.3V signal) PCIe Clock request pin (low to request PCI clock). Internal pulled up
103	USB_N	USB D-
104	Reserved	Do not Connect anything to this pin.
105	USB_P	USB D+
106	Reserved	Do not Connect anything to this pin.
107	GND	Ground (0V)
108	GND	Ground (0V)
109	PCIe_nRST	Output (+3.3V signal) PCIe Reset Low active
110	PCIe_CLK_P	PCIe Clock Out Positive (100MHz) NB AC coupling Capacitor Included on CM4
111	VDAC_COMP	Video DAC output (TV OUT)
112	PCIe_CLK_N	PCIe Clock Out Negative (100MHz) NB AC coupling Capacitor Included on CM4
113	GND	Ground (0V)
114	GND	Ground (0V)
115	CAM1_D0_N	Input Camera1 D0 Negative
116	PCIe_RX_P	Input PCIe GEN 2 RX Positive NB External AC coupling Capacitor required
117	CAM1_D0_P	Input Camera1 D0 Positive
118	PCIe_RX_N	Input PCIe GEN 2 RX Negative NB External AC coupling Capacitor required
119	GND	Ground (0V)
120	GND	Ground (0V)
121	CAM1_D1_N	Input Camera1 D1 Negative
122	PCIe_TX_P	Output PCIe GEN 2 TX Positive NB AC coupling Capacitor Included on CM4
123	CAM1_D1_P	Input Camera1 D1 Positive
124	PCIe_TX_N	Output PCIe GEN 2 TX Positive NB AC coupling Capacitor Included on CM4
125	GND	Ground (0V)
126	GND	Ground (0V)
127	CAM1_C_N	Input Camera1 Clock Negative

128	CAM0_D0_N	Input Camera0 D0 Negative
129	CAM1_C_P	Input Camera1 Clock Positive
130	CAM0_D0_P	Input Camera0 D0 Positive
131	GND	Ground (0V)
132	GND	Ground (0V)
133	CAM1_D2_N	Input Camera1 D2 Negative
134	CAM0_D1_N	Input Camera0 D1 Negative
135	CAM1_D2_P	Input Camera1 D2 Positive
136	CAM0_D1_P	Input Camera0 D1 Positive
137	GND	Ground (0V)
138	GND	Ground (0V)
139	CAM1_D3_N	Input Camera1 D3 Negative
140	CAM0_C_N	Input Camera0 Clock Negative
141	CAM1_D3_P	Input Camera1 D3 Positive
142	CAM0_C_P	Input Camera0 Clock Positive
143	HDMI1_HOTPLUG	Input HDMI1 Hotplug Internally pulled down with a 100K. 5V tolerant. (It can be connected directly to a HDMI connector a small amount of ESD protection is provided on the CM4 by an on board HDMI05-CL02F3)
144	GND	Ground (0V)
145	HDMI1_SDA	Bidir HDMI1 SDA Internally pulled up with a 1.8K. 5V tolerant. (It can be connected directly to a HDMI connector a small amount of ESD protection is provided on the CM4 by an on board HDMI05-CL02F3)
146	HDMI1_TX2_P	Output HDMI1 TX2 Positive
147	HDMI1_SCL	Input HDMI1 SCL Internally pulled up with a 1.8K. 5V tolerant. (It can be connected directly to a HDMI connector a small amount of ESD protection is provided on the CM4 by an on board HDMI05-CL02F3)
148	HDMI1_TX2_N	Output HDMI1 TX2 Negative
149	HDMI1_CEC	Input HDMI1 CEC Internally pulled up with a 27K. 5V tolerant. (It can be connected directly to a HDMI connector a small amount of ESD protection is provided on the CM4 by an on board HDMI05-CL02F3)
150	GND	Ground (0V)
151	HDMI0_CEC	Input HDMI0 CEC Internally pulled up with a 27K. 5V tolerant (It can be connected directly to a HDMI connector a small amount of ESD protection is provided on the CM4 by an on board HDMI05-CL02F3)
152	HDMI1_TX1_P	Output HDMI1 TX1 Positive
153	HDMI0_HOTPLUG	Input HDMI0 Hotplug Internally pulled down 100K. 5V tolerant. (It can be connected directly to a HDMI connector a small amount of ESD protection is provided on the CM4 by an on board HDMI05-CL02F3)
154	HDMI1_TX1_N	Output HDMI1 TX1 Negative
155	GND	Ground (0V)
156	GND	Ground (0V)

157	DSI0_D0_N	Output Display0 D0 Negative
158	HDMI1_TX0_P	Output HDMI1 TX0 Positive
159	DSI0_D0_P	Output Display0 D0 Positive
160	HDMI1_TX0_N	Output HDMI1 TX0 Negative
161	GND	Ground (0V)
162	GND	Ground (0V)
163	DSI0_D1_N	Output Display0 D1 Negative
164	HDMI1_CLK_P	Output HDMI1 Clock Positive
165	DSI0_D1_P	Output Display0 D1 Positive
166	HDMI1_CLK_N	Output HDMI1 Clock Negative
167	GND	Ground (0V)
168	GND	Ground (0V)
169	DSI0_C_N	Output Display0 Clock Negative
170	HDMI0_TX2_P	Output HDMI0 TX2 Positive
171	DSI0_C_P	Output Display0 Clock Positive
172	HDMI0_TX2_N	Output HDMI0 TX2 Negative
173	GND	Ground (0V)
174	GND	Ground (0V)
175	DSI1_D0_N	Output Display1 D0 Negative
176	HDMI0_TX1_P	Output HDMI0 TX1 Positive
177	DSI1_D0_P	Output Display1 D0 Positive
178	HDMI0_TX1_N	Output HDMI0 TX1 Negative
179	GND	Ground (0V)
180	GND	Ground (0V)
181	DSI1_D1_N	Output Display1 D1 Negative
182	HDMI0_TX0_P	Output HDMI0 TX0 Positive
183	DSI1_D1_P	Output Display1 D1 Positive
184	HDMI0_TX0_N	Output HDMI0 TX0 Negative
185	GND	Ground (0V)
186	GND	Ground (0V)
187	DSI1_C_N	Output Display1 Clock Negative
188	HDMI0_CLK_P	Output HDMI0 Clock Positive
189	DSI1_C_P	Output Display1 Clock Positive
190	HDMI0_CLK_N	Output HDMI0 Clock Negative
191	GND	Ground (0V)
192	GND	Ground (0V)
193	DSI1_D2_N	Output Display1 D2 Negative

194	DSI1_D3_N	Output Display1 D3 Negative
195	DSI1_D2_P	Output Display1 D2 Positive
196	DSI1_D3_P	Output Display1 D3 Positive
197	GND	Ground (0V)
198	GND	Ground (0V)
199	HDMI0_SDA	Bidir HDMI0 SDA Internally pulled up with a 1.8K. 5V tolerant. (It can be connected directly to a HDMI connector a small amount of ESD protection is provided on the CM4 by an on board HDMI05-CL02F3)
200	HDMI0_SCL	Bidir HDMI0 SCL Internally pulled up with a 1.8K. 5V tolerant. (It can be connected directly to a HDMI connector a small amount of ESD protection is provided on the CM4 by an on board HDMI05-CL02F3)

All ground pins should be connected. If none of the signals on the second connector pins 101 to 200 are used then you may not fit the connector to reduce costs, but mechanical stablity needs to be considered.

The voltage on GPIO pins 0-27 must not exceed `CM4_3.3V` if +3.3V signalling is used or `CM4_1.8V` if +1.8V signalling is used. These pins are the same as on the 40-pin connector on the Raspberry Pi 4, Model B.

If the `CM4_1.8V` rail is use to power other devices other than the `GPIO_Vref` then you should ensure that in case of surprise power removal (e.g.the +5V pin goes below +4.5V) from the CM4, the load on the `CM4_1.8V` must go to zero.

Similarly if the `CM4_3.3V` rail is used to power other devices other than the `GPIO_Vref`, then you should ensure that in the case surprise power removal the `CM4_3.3V` rail never fails below the `CM4_1.8V` rail. This is the typical case, but you should check this in your design. In the case where it does fall below the `CM4_1.8V` rail, then extra circuitry is required to disconnect the `CM4_3.3V` load

No reverse voltage must be applied to any pin or power up may be prevented, i.e. during power down/off no pin may have external voltage applied otherwise this may prevent power up.

4.1. Differential Pairs

It is recommended that P/N signals within a pair are matched to better 0.15mm. Often matching between pairs is not so critical, e.g. HDMI pair to pair matching should be better than 25mm so on a typical board no extra matching is required.

4.1.1. 100Ω Differential pairs signal lengths

On the CM4 all differential pairs are matched to better than 0.05mm (P/N signals).

NOTE

It is recommended that pairs are also matched on the interface board.

On the CM4 pair to pairs aren't always matched as many interfaces don't require very accurate matching between pairs. Table 7 documents the CM4 track length difference within each group (a non zero value is how much longer in mm that track is compared to the signal with zero length difference)

Table 7. 100 Ω Differential paris signal length

Signal	Length
CAM0_C_N	0.02
CAM0_C_P	0.02
CAM0_D0_N	0.06

CAM0_D0_P	0.07
CAM0_D1_N	0
CAM0_D1_P	0.01
CAM1_C_N	0.78
CAM1_C_P	0.78
CAM1_D0_N	0.02
CAM1_D0_P	0.01
CAM1_D1_N	0.4
CAM1_D1_P	0.4
CAM1_D2_N	0.05
CAM1_D2_P	0.04
CAM1_D3_N	0.01
CAM1_D3_P	0
DSI0_C_N	0
DSI0_C_P	0
DSI0_D0_N	0
DSI0_D0_P	0
DSI0_D1_N	0.01
DSI0_D1_P	0.01
DSI1_C_N	1.28
DSI1_C_P	1.28
DSI1_D0_N	0
DSI1_D0_P	0.01
DSI1_D1_N	1.06
DSI1_D1_P	1.06
DSI1_D2_N	0.83
DSI1_D2_P	0.84
DSI1_D3_N	3.78
DSI1_D3_P	3.79
HDMI0_CLK_N	3.25
HDMI0_CLK_P	3.24
HDMI0_TX0_N	1.76
HDMI0_TX0_P	1.76
HDMI0_TX1_N	0.62

HDMI0_TX1_P	0.62
HDMI0_TX2_N	0
HDMI0_TX2_P	0
HDMI1_CLK_N	2.47
HDMI1_CLK_P	2.46
HDMI1_TX0_N	1.51
HDMI1_TX0_P	1.51
HDMI1_TX1_N	1
HDMI1_TX1_P	1
HDMI1_TX2_N	0
HDMI1_TX2_P	0.01
Ethernet_Pair0_P	5.23
Ethernet_Pair0_N	5.23
Ethernet_Pair1_P	0
Ethernet_Pair1_N	0
Ethernet_Pair2_P	3.82
Ethernet_Pair2_N	3.82
Ethernet_Pair3_P	4.29
Ethernet_Pair3_N	4.29

4.1.2. 90Ω Differential Pairs signal lengths

On the CM4 all differential pairs are matched to better than 0.05mm (P/N signals).

NOTE

It is recommended that pairs are also matched on the interface board.

However pair to pairs aren't always matched as many interfaces don't require very accurate matching between pairs. Table 8 documents the CM4 track length difference within each group (a non zero value is how much longer in mm that track is compared to the signal with zero length difference)

Table 8. 90 Ω Differential paris signal length

Signal	Length
PCIe_CLK_P	0.65
PCIe_CLK_N	0.65
PCIe_TX_P	0
PCIe_TX_N	0
PCIe_RX_P	0.23
PCIe_RX_N	0.23

USB2_P	0
USB2_N	0

Chapter 5. Power

5.1. Power up sequencing

The CM4 requires a single +5V supply, and can supply up to 600mA at +3.3V and +1.8V to peripherals.

All pins should not have any power applied to them before the +5V rail is applied.

If the EEPROM is to be write protected then the `EEPROM_nWP` should be low before powerup.

If the CM4 is to be booted using USB then `RPI_nBOOT` needs to be low within 2ms of +5V rising.

+5V should rise monotonically to 4.75V and stay above 4.75V for the entire operation of the CM4.

The power up sequence will start when both +5V rall is above 4.75V and `GLOBAL_EN` rises. `GLOBAL_EN` has internal RC delay so that it rises after +5V has risen. The order of events is as follows

1. +5v rises
2. `GLOBAL_EN` rises
3. +3.3V rises
4. +1.8V rises at least 1mS after +3.3v
5. `RUN_PG` rises at least 10mS after +1.8v
6. `EXT_nRESET` rises at least 1second after `RUN_PG`

5.2. Power down sequencing

The OS should be shut down to ensure that the file system remains consistent, before the power is removed. If this can't be achieved, then a filesystem like `btrfs`, `f2fs` or `overlayfs` (use `raspi-config` to enable it) should be considered.

Once the OS has shutdown the +5V rail can be removed or the `GLOBAL_EN` pin can be taken low to put the CM4 into the lowest power mode.

During the shutdown sequence the +1.8v will be discharged before the +3.3v rail.

5.3. Power Consumption

The exact power consumption of the CM4 will greatly depend on the tasks being run on the CM4. The lowest shutdown power consumption mode is with the `GLOBAL_EN` driven low, typically is 15uA. With `GLOBAL_EN` high but software shutdown the typical consumption is 8mA. Idle power cosumption is typically 400mA , but this varys considerable depending on the Operating system. Operating power consuption is typically around 1.4A again this greatly depends on the Operating System and the Tasks being executed.

5.4. Regulator Outputs

To make it easier to interface to the CM4 the on board regulators (+3.3v and +1.8v) can each supply 600mA to devices connected to the CM4. The loads on these outputs isn't taken into account in the power consumption figures.

Appendix A: Troubleshooting

The CM4 has a number of stages of power up before the CPU starts. If there is an error at any of the stages, power up will be halted.

Hardware Checklist

1. Is the +5V supply good? Check this by pulling `GLOBAL_EN` low apply and apply an external 2A load to the +5V supply. Does it stay >+4.75V including noise? Ideally it should remain >+4.9V including any noise.
2. Remove external 2A load, but keep `GLOBAL_EN` pulled low.
3. Check the CM4 +3.3v rail is <200mV. If this is not the case there is an external power path back-feeding the CM4, either directly or indirectly. This could also occur via the digital pins, e.g Ethernet.
4. Still with `GLOBAL_EN` pulled low check the CM4 +1.8v rail is <200mW. Again if the +1.8v rail is above 200mV then there is an external path back feeding the 1.8v rail. (If nothing is connected to these pins you can ignore this check.)
5. Remove the pull down on `GLOBAL_EN`.
6. Check `GLOBAL_EN` now goes high (it internally pulled up on the CM4)
7. Check the +3.3V supply rises to >+3.15V. If it does not, this suggests there is too much load on the +3.3V rail.
8. Check the +1.8V rail gets to >+1.71v. If it does not, this suggests there is much load on the +1.8V rail.
9. Check `RUN_PG` goes high
10. Check `ACT_LED` starts to oscillate to indicate booting check it isn't flashing an error code.

Bootloader

1. Connect a HDMI cable to see if the HDMI diagnostics screen appears.
2. Connect a USB serial cable to GPIO pins 14,15.
   a. See https://www.raspberrypi.org/documentation/configuration/uart.md for details.
3. Short the `nRPIBOOT` pin to ground to force USB boot mode. The CM4IO board has a jumper for `nRPIBOOT` This can be used to enable different boot modes (e.g. network) and enable UART logging.
   a. See https://www.raspberrypi.org/documentation/hardware/computemodule/cm-emmc-flashing.md

rpi-eeprom-update

1. CM4 will not run `recovery.bin` from from the EMMC (or SD Card on CM4Lite). Therefore, the only way to update the bootloader EEPROM is via `usbboot` or self-update.

EEPROM Write protect

The on board EEPROM can be write protected by shorting to ground `EEPROM_nWP`. The CM4IO board has a jumper for `EEPROM_nWP`.

1. See https://www.raspberrypi.org/documentation/hardware/raspberrypi/bcm2711_bootloader_config.md

Firmware

1. A 5.4 or newer kernel and the latest firmware release is required. These can be updated by using usbboot to mount the EMMC as a USB MSD device.
2. Nightly OS images are now available which contain `rpi-update` master firmware + kernel. Bug fixes for CM4 will normally be provided via these images except where a test/patch binary is required.
   a. See http://downloads.raspberrypi.org/nightlies/

Kernel

1. The updated OS images use the new Raspberry Pi Compute Module 4 device tree file. If that is not found then the Raspberry Pi 4, Model B device tree file will be used.
   a. See https://github.com/raspberrypi/linux/blob/rpi-5.4.y/arch/arm/boot/dts/bcm2711-rpi-cm4.dts

Appendix B: Availability

Raspberry Pi guarantees availability of the CM4 until at least **January 2028**.

Support

For support please see the hardware documentation section of the Raspberry Pi website and post questions to the Raspberry Pi forum.

Ordering codes

Table 9. Part Number Options

Model	Wireless	RAM LPDDR4	eMMC Storage
CM4	0 = No	01 = 1GByte	000 = 0GByte (Lite)
	1 = Yes	02 = 2GByte	008 = 8GByte
		04 = 4GByte	016 = 16GByte
		08 = 8GByte	032 = 32GByte
**Example Part Number**			
CM4	1	02	032

Table 10. Ordering Options

Wireless	RAM LPDDR4	Storage eMMC	RPTL #	Part Number	Order Multiple	RRP
-	1GB	Lite	SC0318	CM4001000	1+ / Bulk	$ 25.00
-	1GB	8GB	SC0319	CM4001008	1+ / Bulk	$ 30.00
-	1GB	16GB	SC0320	CM4001016	1+ / Bulk	$ 35.00
-	1GB	32GB	SC0321	CM4001032	1+ / Bulk	$ 40.00
Yes	1GB	Lite	SC0314	CM4101000	Bulk	$ 30.00
Yes	1GB	8GB	SC0315	CM4101008	Bulk	$ 35.00
Yes	1GB	16GB	SC0316	CM4101016	Bulk	$ 40.00
Yes	1GB	32GB	SC0317	CM4101032	Bulk	$ 45.00
-	2GB	Lite	SC0287	CM4002000	1+ / Bulk	$ 30.00
-	2GB	8GB	SC0288	CM4002008	1+ / Bulk	$ 35.00
-	2GB	16GB	SC0289	CM4002016	1+ / Bulk	$ 40.00
-	2GB	32GB	SC0290	CM4002032	1+ / Bulk	$ 45.00
Yes	2GB	Lite	SC0275	CM4102000	1+ / Bulk	$ 35.00
Yes	2GB	8GB	SC0276	CM4102008	1+ / Bulk	$ 40.00
Yes	2GB	16GB	SC0277	CM4102016	1+ / Bulk	$ 45.00
Yes	2GB	32GB	SC0278	CM4102032	1+ / Bulk	$ 50.00
-	4GB	Lite	SC0291	CM4004000	Bulk	$ 45.00
-	4GB	8GB	SC0292	CM4004008	Bulk	$ 50.00

-	4GB	16GB	SC0293	CM4004016	Bulk	$ 55.00
-	4GB	32GB	SC0294	CM4004032	Bulk	$ 60.00
Yes	4GB	Lite	SC0279	CM4104000	1+ / Bulk	$ 50.00
Yes	4GB	8GB	SC0280	CM4104008	1+ / Bulk	$ 55.00
Yes	4GB	16GB	SC0281	CM4104016	1+ / Bulk	$ 60.00
Yes	4GB	32GB	SC0282	CM4104032	1+ / Bulk	$ 65.00
-	8GB	Lite	SC0295	CM4008000	Bulk	$ 70.00
-	8GB	8GB	SC0296	CM4008008	Bulk	$ 75.00
-	8GB	16GB	SC0297	CM4008016	Bulk	$ 80.00
-	8GB	32GB	SC0298	CM4008032	Bulk	$ 85.00
Yes	8GB	Lite	SC0283	CM4108000	Bulk	$ 75.00
Yes	8GB	8GB	SC0284	CM4108008	Bulk	$ 80.00
Yes	8GB	16GB	SC0285	CM4108016	Bulk	$ 85.00
Yes	8GB	32GB	SC0286	CM4108032	Bulk	$ 90.00

NOTE

RRP was correct at time of publication and excludes taxes.

Some options have minimum ordering qualities (MOQ), please check with your supplier.

For prototyping often a higher LPDDR RAM capacity option will exist, without an MOQ. You can use the higher LPDDR RAM option, but limit it to the lower capacity by changing config.txt.

Packaging

Small quantities are supplied in individual cardboard boxes. These have an internal ESD coating so that a separate ESD bag isn't required. This packaging is recyclable and reduces waste.

Raspberry Pi

Raspberry Pi is a trademark of the Raspberry Pi Foundation

Raspberry Pi Trading Ltd
