# Supplementary material for: The Unfolding Space Glove: A Wearable Spatio-Visual to Haptic Sensory Substitution Device for Blind People
Source: Sensors (Basel). 2022 Feb 26;22(5):1859. doi: 10.3390/s22051859 (PMC8914703; doi:10.3390/s22051859)
Supplement: Supplementary file 1 [file sensors-22-01859-s001.zip › S1_GitHub-Release_unfolding-space-hardware-0.2.1-revised.zip!/unfolding-space-hardware-0.2.1-revised/pcb/datasheets/cm4-product-brief.pdf]

Raspberry Pi Compute Module 4

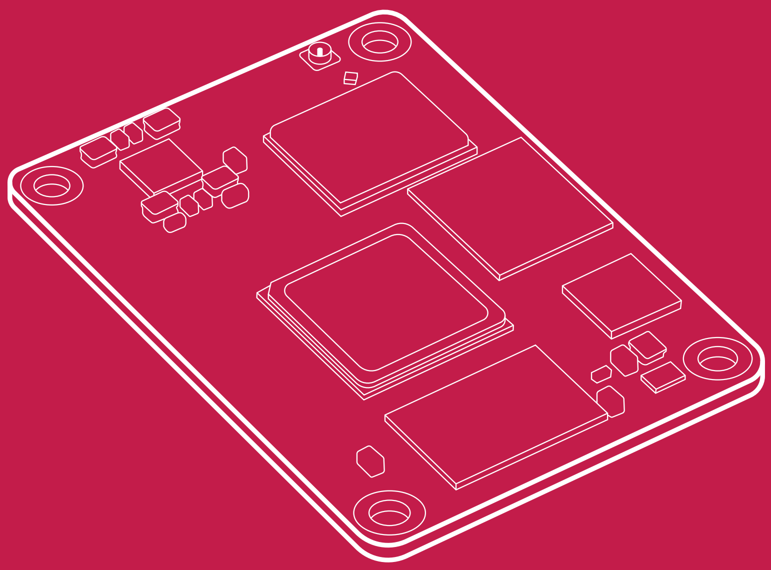

Published in January 2021
by Raspberry Pi Trading Ltd.

www.raspberrypi.org

Raspberry Pi

Overview

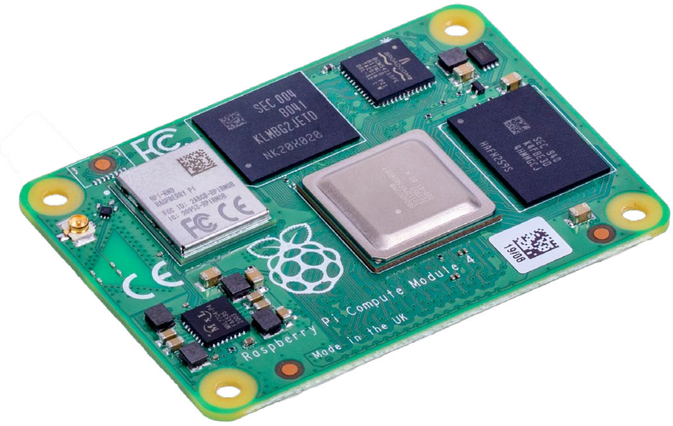

Raspberry Pi Compute Module 4 harnesses the compute power of the popular Raspberry Pi 4 Model B, bringing it to a smaller form factor suitable for integration into products.

Key features include a high-performance 64-bit quad-core processor, dual-display support at resolutions up to 4K, hardware video decode at up to 4Kp60, up to 8GB of RAM, Gigabit Ethernet, USB 2.0, dual camera interfaces, and PCIe Gen 2 x1 interface.

The optional dual-band 2.4/5.0GHz wireless LAN and Bluetooth 5.0 have modular compliance certification. This allows the board to be designed into end products with significantly reduced compliance testing, improving both cost and time to market. Either the onboard antenna or an external antenna kit can be used.

Compute Module 4 has optional onboard eMMC of 8GB, 16GB or 32GB.

Specification

**Form factor:**	55 mm × 40 mm
**Processor:**	Broadcom BCM2711 quad-core Cortex-A72 (ARM v8) 64-bit SoC @ 1.5GHz
**Memory:**	1GB, 2GB, 4GB or 8GB LPDDR4 (depending on model) with on-die ECC
**Connectivity:**	• Optional wireless LAN, 2.4GHz and 5.0GHz IEEE 802.11b/g/n/ac wireless, Bluetooth 5.0, BLE with onboard and external antenna options<br>• Onboard Gigabit Ethernet PHY supporting IEEE1588<br>• 1 × USB 2.0 interface<br>• PCIe Gen 2 x1 interface<br>• 28 GPIO signals<br>• SD card interface for SD card or external eMMC (for use only with Compute Module 4 variants without eMMC)
**Video:**	• Dual HDMI interface (up to 4Kp60 supported)<br>• 2-lane MIPI DSI display interface<br>• 2-lane MIPI CSI camera interface<br>• 4-lane MIPI DSI display interface<br>• 4-lane MIPI CSI camera interface
**Multimedia:**	H.265 (4Kp60 decode); H.264 (1080p60 decode, 1080p30 encode); OpenGL ES 3.0 graphics
**Input power:**	5V DC
**Operating temperature:**	-20°C to +85°C
**Production lifetime:**	Raspberry Pi Compute Module 4 will remain in production until at least January 2028
**Compliance:**	For a full list of local and regional product approvals, please visit<br>www.raspberrypi.org/documentation/hardware/raspberrypi/conformity.md

Physical specifications

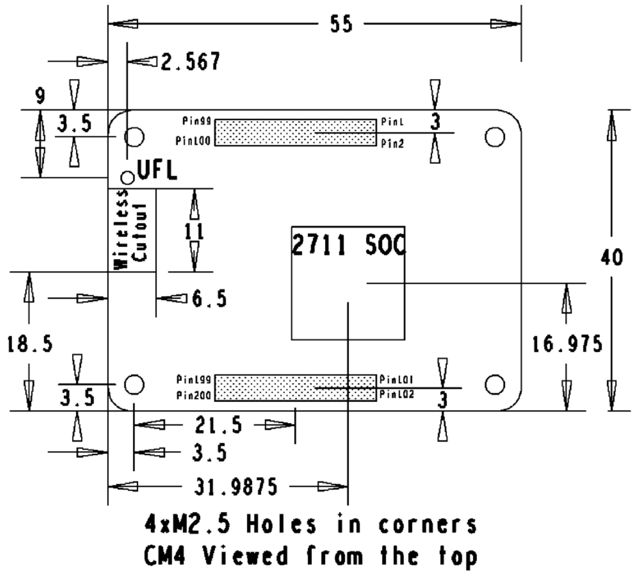

Note: all dimensions in mm

WARNINGS

- Any external power supply used with Raspberry Pi Compute Module 4 shall comply with relevant regulations and standards applicable in the country of intended use.
- This product should be operated in a well-ventilated environment, and if used inside a case, the case should not be covered.
- Whilst in use, this product should be placed on a stable, flat, non-conductive surface, and should not be contacted by conductive items.
- The connection of incompatible devices to Compute Module 4 may affect compliance, result in damage to the unit, and invalidate the warranty.
- All peripherals used with this product should comply with relevant standards for the country of use and be marked accordingly to ensure that safety and performance requirements are met. These articles include but are not limited to keyboards, monitors, and mice when used in conjunction with the Compute Module.
- The cables and connectors of all peripherals used with this product must have adequate insulation so that relevant safety requirements are met.

SAFETY INSTRUCTIONS

To avoid malfunction or damage to this product, please observe the following:

- Do not expose to water or moisture, or place on a conductive surface whilst in operation.
- Do not expose to heat from any source; Raspberry Pi Compute Module 4 is designed for reliable operation at normal ambient temperatures.
- Take care whilst handling to avoid mechanical or electrical damage to the printed circuit board and connectors.
- Whilst it is powered, avoid handling the printed circuit board, or only handle it by the edges to minimise the risk of electrostatic discharge damage.

Pricing

Part Number	Wireless	RAM	eMMC	Price*
**CM4**001000	No	1GB	0GB (Lite)	$25
**CM4**001008			8GB	$30
**CM4**001016			16GB	$35
**CM4**001032			32GB	$40
**CM4**002000		2GB	0GB (Lite)	$30
**CM4**002008			8GB	$35
**CM4**002016			16GB	$40
**CM4**002032			32GB	$45
**CM4**004000		4GB	0GB (Lite)	$45
**CM4**004008			8GB	$50
**CM4**004016			16GB	$55
**CM4**004032			32GB	$60
**CM4**008000		8GB	0GB (Lite)	$70
**CM4**008008			8GB	$75
**CM4**008016			16GB	$80
**CM4**008032			32GB	$85
**CM4**101000	Yes	1GB	0GB (Lite)	$30
**CM4**101008			8GB	$35
**CM4**101016			16GB	$40
**CM4**101032			32GB	$45
**CM4**102000		2GB	0GB (Lite)	$35
**CM4**102008			8GB	$40
**CM4**102016			16GB	$45
**CM4**102032			32GB	$50
**CM4**104000		4GB	0GB (Lite)	$50
**CM4**104008			8GB	$55
**CM4**104016			16GB	$60
**CM4**104032			32GB	$65
**CM4**108000		8GB	0GB (Lite)	$75
**CM4**108008			8GB	$80
**CM4**108016			16GB	$85
**CM4**108032			32GB	$90

* pricing excludes sales tax and any applicable import duties

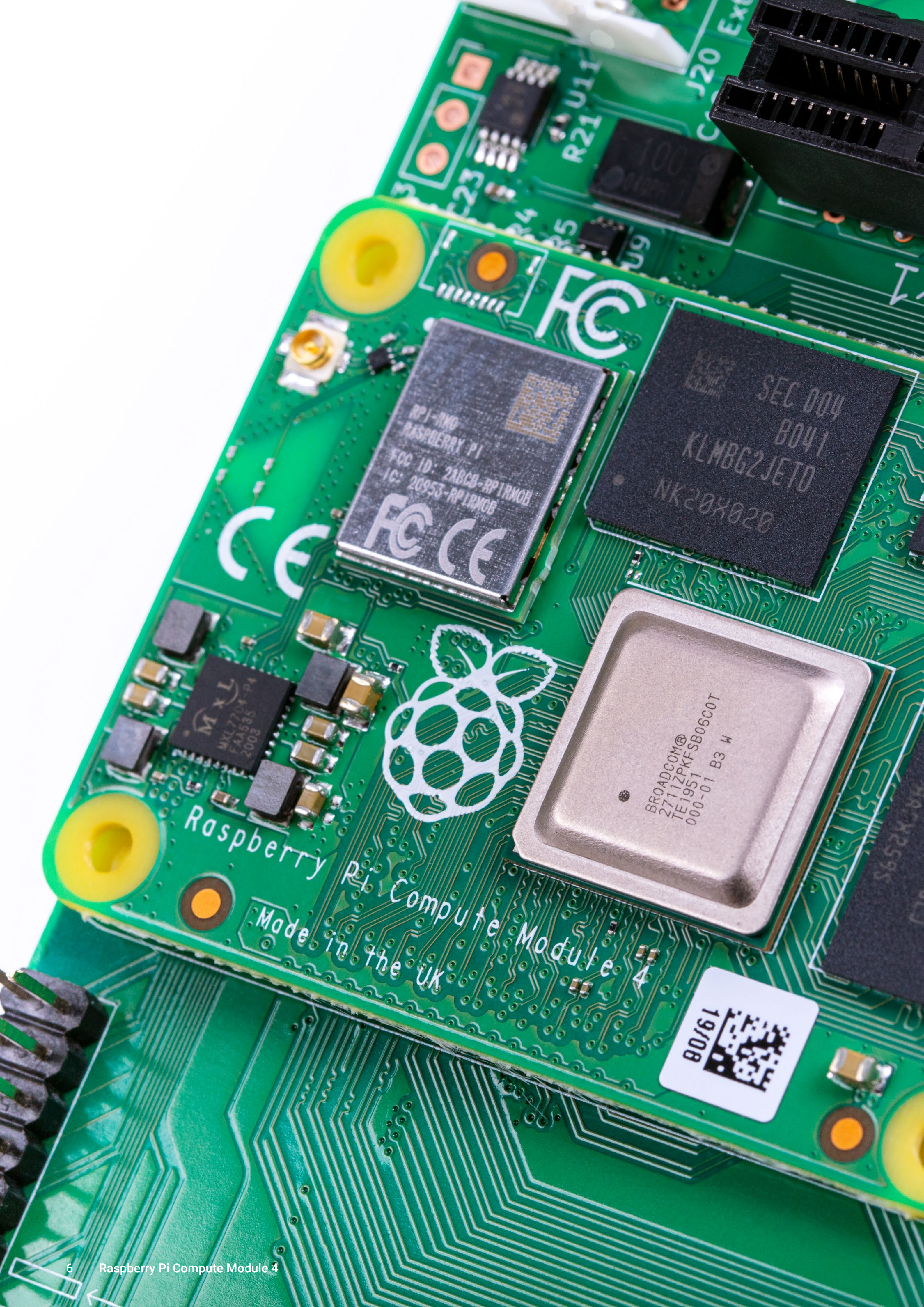
FC
SEC 004
B041
KLMBG2JETD
NK20X020
RPI-RM0
RASPBERRY PI
FCC ID: 2ABCB-RPIRM0B
IC: 20953-RPIRM0B
CE
BROADCOM®
2711ZPKFSB06C0T
TE1951 B3 W
000-01
Raspberry Pi Compute Module 4
Made in the UK
19/08
MXL7704-P4
FAA535
2003
J20
U11
R21
C23
U9

HDMI is a trademark of HDMI Licensing, LLC
Raspberry Pi and the Raspberry Pi logo are trademarks of the Raspberry Pi Foundation

www.raspberrypi.org

Raspberry Pi
