# Supplementary material for: The Unfolding Space Glove: A Wearable Spatio-Visual to Haptic Sensory Substitution Device for Blind People
Source: Sensors (Basel). 2022 Feb 26;22(5):1859. doi: 10.3390/s22051859 (PMC8914703; doi:10.3390/s22051859)
Supplement: Supplementary file 1 [file sensors-22-01859-s001.zip › S1_GitHub-Release_unfolding-space-hardware-0.2.1-revised.zip!/unfolding-space-hardware-0.2.1-revised/pcb/datasheets/drv2605l.pdf]

DRV2605L 2- to 5.2-V Haptic Driver for LRA and ERM with Effect Library and Smart-Loop Architecture

1 Features

- Flexible Haptic and Vibration Driver
  - LRA (Linear Resonance Actuator)
  - ERM (Eccentric Rotating Mass)
- I^2C-Controlled Digital Playback Engine
  - Waveform Sequencer and Trigger
  - Real-Time Playback Mode through I^2C
  - I^2C Dual-Mode Drive (Open and Closed Loop)
- Smart-Loop Architecture (Patent Pending Control Algorithm)
  - Automatic Overdrive and Braking
  - Automatic Resonance Tracking and Reporting (LRA Only)
  - Automatic Actuator Diagnostic
  - Automatic Level Calibration
  - Wide Support for Actuator Models
- Licensed Immersion TouchSense® 2200 Features:
  - Integrated Immersion Effect Library
  - Audio-to-Vibe
- Drive Compensation Over Battery Discharge
- Wide Voltage Operation (2 V to 5.2 V)
- Efficient Differential Switching Output Drive
- PWM Input with 0% to 100% Duty-Cycle Control Range
- Hardware Trigger Input
- Fast Startup Time
- 1.8-V Compatible, V_{DD}-Tolerant Digital Interface

2 Applications

- Mobile Phones and Tablets
- Watches and Wearable Technology
- Remote Controls, Mice, and Peripheral Devices
- Touch-Enabled Devices
- Industrial Human-Machine Interfaces
- Electronic Point of Sale (ePOS)

3 Description

The DRV2605L device is a low-voltage haptic driver that includes a haptic-effect library and provides a closed-loop actuator-control system for high-quality tactile feedback for ERM and LRA. This schema helps improve actuator performance in terms of acceleration consistency, start time, and brake time and is accessible through a shared I^2C compatible bus or PWM input signal.

The DRV2605L device offers a licensed version of TouchSense 2200 software from Immersion which eliminates the requirement to design haptic waveforms because the software includes over 100 licensed effects (6 ERM libraries and 1 LRA library) and audio-to-vibe features.

Additionally, the real-time playback mode allows the host processor to bypass the library playback engine and play waveforms directly from the host through I^2C.

The smart-loop architecture inside the DRV2605L device allows simple auto-resonant drive for the LRA as well as feedback-optimized ERM drive allowing for automatic overdrive and braking. The smart-loop architecture creates a simplified input waveform interface as well as reliable motor control and consistent motor performance. The DRV2605L device also features automatic transition to an open-loop system in the event that an LRA actuator is not generating a valid back-EMF voltage. When the LRA generates a valid back-EMF voltage, the DRV2605L device automatically synchronizes with the LRA. The DRV2605L also allows for open-loop driving through the use of internally-generated PWM. Additionally, the audio-to-vibe mode automatically converts an audio input signal to meaningful tactile effects.

For an important notice regarding Immersion software, see the *Legal Notice* section.

Device Information(1)

PART NUMBER	PACKAGE	BODY SIZE (MAX)
DRV2605L	DSBGA (9)	1.50 mm × 1.50 mm
DRV2605L	VSSOP (10)	3.00 mm × 3.00 mm

(1) For all available packages, see the orderable addendum at the end of the datasheet.

Simplified Schematic

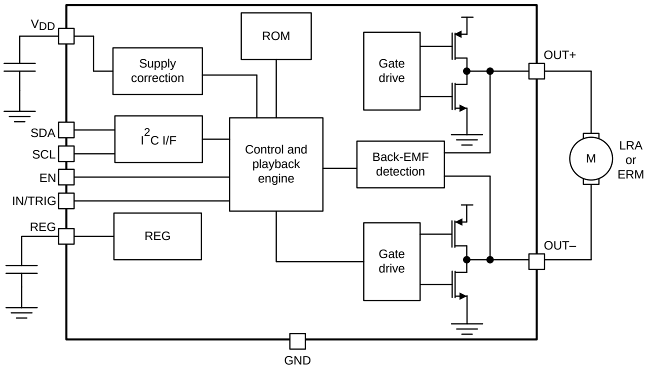

An IMPORTANT NOTICE at the end of this data sheet addresses availability, warranty, changes, use in safety-critical applications, intellectual property matters and other important disclaimers. PRODUCTION DATA.

Table of Contents

1 **Features** ... 1
2 **Applications** ... 1
3 **Description** ... 1
4 **Revision History** ... 2
5 **Pin Configuration and Functions** ... 4
6 **Specifications** ... 6
6.1 Absolute Maximum Ratings ... 6
6.2 ESD Ratings ... 6
6.3 Recommended Operating Conditions ... 6
6.4 Thermal Information ... 6
6.5 Electrical Characteristics ... 7
6.6 Timing Requirements ... 7
6.7 Switching Characteristics ... 7
6.8 Typical Characteristics ... 9
7 **Parameter Measurement Information** ... 10
7.1 Test Setup for Graphs ... 10
8 **Detailed Description** ... 11
8.1 Overview ... 11
8.2 Functional Block Diagram ... 11
8.3 Feature Description ... 12
8.4 Device Functional Modes ... 20
8.5 Programming ... 24
8.6 Register Map ... 35
9 **Application and Implementation** ... 55
9.1 Application Information ... 55
9.2 Typical Application ... 56
9.3 Initialization Setup ... 59
10 **Power Supply Recommendations** ... 60
11 **Layout** ... 61
11.1 Layout Guidelines ... 61
11.2 Layout Example ... 62
12 **Device and Documentation Support** ... 63
12.1 Device Support ... 63
12.2 Documentation Support ... 64
12.3 Receiving Notification of Documentation Updates 64
12.4 Community Resource ... 64
12.5 Trademarks ... 64
12.6 Electrostatic Discharge Caution ... 64
12.7 Glossary ... 64
13 **Mechanical, Packaging, and Orderable Information** ... 65

4 Revision History

NOTE: Page numbers for previous revisions may differ from page numbers in the current version.

Changes from Revision C (September 2014) to Revision D — **Page**

- Changed the *Handling Ratings* table to *ESD Ratings* and moved the storage temperature to the *Absolute Maximum Ratings* table ... 6
- Changed AC couple capacitor recommendation from 0.1 µF to 1 µF to be consistent with Recommended External Components table in the *Waveform Playback Using Audio-to-Vibe Mode* ... 33
- Changed the DEFAULT value for bit 5-4 of Table 25 From: 1 To 3 ... 48
- Changed the DEFAULT value for bit 3-2 of Table 25 From: 2 To 1 ... 49
- Changed the DEFAULT value for bit 1-0 of Table 25 From: 2 To 1 ... 50
- Changed the typical value of $C_{(VDD)}$ in Table 32 From: 0.1 µF To: 1 µF ... 55

Changes from Revision B (June 2014) to Revision C — **Page**

- Added VSSOP package option ... 4
- Added IN/TRIG pin connection to GND required if not used ... 4
- Changed connection terminal of input impedance from GND to $V_{(CM\_ANA)}$ in *Electrical Characteristics* section ... 7
- Added exceptional behavior for I^2C Watchdog Timer ... 19

Changes from Revision A (May 2014) to Revision B — **Page**

- Changed the view listed for the DSBGA package drawing from bottom to top ... 4
- Changed the default value of NG_THRESH[1:0] from 1 to 2 in the *Register Map* ... 51

Copyright © 2014–2018, Texas Instruments Incorporated

Changes from Original (May 2014) to Revision A **Page**

- Changed device status from *Product Preview* to *Production Data* 1
- Changed minimum supported resonant frequency from 50 Hz to 125 Hz 6
- Added digital pulldown resistance parameter to *Electrical Characteristics* 7
- Changed calibration diagram to include DRIVE_TIME into ERM requirements 28
- Changed bitfield name from "LRA_DRIVE_MODE" to "OTP_STATUS" 52
- Changed $C_{(REG)}$ from 0.1 to 1 µF in Figure 60 56

5 Pin Configuration and Functions

YZF Package
9-Pin DSBGA With 0.5-mm Pitch
(Top View)

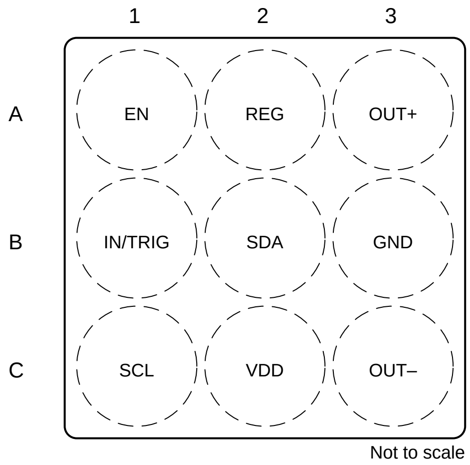

Pin Functions

PIN		TYPE(1)	DESCRIPTION
NO.	NAME		
A1	EN	I	Device enable
A2	REG	O	The REG pin is the 1.8-V regulator output. A 1-µF capacitor is required.
A3	OUT+	O	Positive haptic driver differential output
B1	IN/TRIG	I	Multi-mode Input. I^2C selectable as PWM, analog, or trigger. If not used, this pin should be connected to GND
B2	SDA	I/O	I^2C data
B3	GND	P	Supply ground
C1	SCL	I	I^2C clock
C3	OUT–	O	Negative haptic-driver differential output
C2	V_{DD}	P	Supply input (2 to 5.2 V). A 1-µF capacitor is required.

(1) I = input, O = output, I/O = input and output, P = power

Copyright © 2014–2018, Texas Instruments Incorporated

DGS Package
10-Pin VSSOP
(Top View)

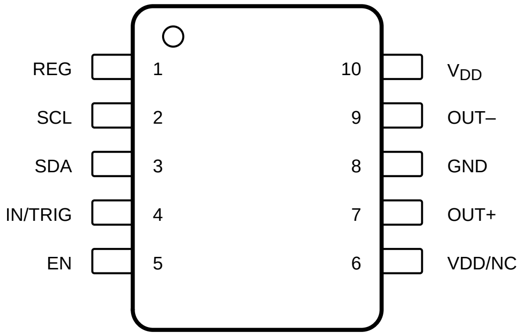

Not to scale

Pin Functions

PIN		TYPE(1)	DESCRIPTION
NO.	NAME		
1	REG	O	The REG pin is the 1.8-V regulator output. A 1-µF capacitor required
2	SCL	I	I^2C clock
3	SDA	I/O	I^2C data
4	IN/TRIG	I	Multi-mode Input. I^2C is selectable as PWM, analog, or trigger. If not used, this pin should be connected to GND
5	EN	I	Device enable
6	V_{DD}/NC	P	Optional supply input. This pin should be tied to V_{DD} or left floating.
7	OUT+	O	Positive haptic driver differential output
8	GND	P	Supply ground
9	OUT–	O	Negative haptic driver differential output
10	V_{DD}	P	Supply Input (2 V to 5.2 V). A 1-µF capacitor is required.

(1) I = input, O = output, I/O = input and output, P = power

6 Specifications

6.1 Absolute Maximum Ratings

over operating free-air temperature range, T_A = 25°C (unless otherwise noted)

		MIN	MAX	UNIT
Input voltage	V_{DD}	–0.3	5.5	V
	EN	–0.3	V_{DD} + 0.3	V
	SDA	–0.3	V_{DD} + 0.3	V
	SCL	–0.3	V_{DD} + 0.3	V
	IN/TRIG	–0.3	V_{DD} + 0.3	V
Operating free-air temperature, T_A		–40	85	°C
Operating junction temperature, T_J		–40	150	°C
Storage temperature, T_{stg}		–65	150	°C

6.2 ESD Ratings

				VALUE	UNIT
**9-PIN DSBGA PACKAGE**					
$V_{(ESD)}$	Electrostatic discharge	Human body model (HBM), per ANSI/ESDA/JEDEC JS-001 (1)		±1000	V
		Charged device model (CDM), per JEDEC specification JESD22-C101, all pins (2)		±250	
**10-PIN VSSOP PACKAGE**					
$V_{(ESD)}$	Electrostatic discharge	Human body model (HBM), per ANSI/ESDA/JEDEC JS-001	OUT+, OUT– pins (3)	±500	V
			Other pins (1)	±1000	
		Charged device model (CDM), per JEDEC specification JESD22-C101, all pins (2)		±250	

(1) JEDEC document JEP155 states that 500-V HBM allows safe manufacturing with a standard ESD control process. Pins listed as ±1000 V may actually have higher performance.
(2) JEDEC document JEP157 states that 250-V CDM allows safe manufacturing with a standard ESD control process. Pins listed as ±250 V may actually have higher performance.
(3) JEDEC document JEP155 states that 500-V HBM allows safe manufacturing with a standard ESD control process.

6.3 Recommended Operating Conditions

over operating free-air temperature range (unless otherwise noted)

			MIN	MAX	UNIT
V_{DD}	Supply voltage	V_{DD}	2	5.2	V
$f_{(PWM)}$	PWM input frequency (1)	IN/TRIG Pin	10	250	kHz
Z_L	Load impedance (1)	V_{DD} = 5.2 V	8		Ω
V_{IL}	Digital low-level input voltage	EN, IN/TRIG, SDA, SCL		0.5	V
V_{IH}	Digital high-level input voltage	EN, IN/TRIG, SDA, SCL	1.3		V
$V_{I(ANA)}$	Input voltage (analog mode)	IN/TRIG	0	1.8	V
$f_{(LRA)}$	LRA Frequency Range (1)		125	300	Hz

(1) Ensured by design. Not production tested.

6.4 Thermal Information

THERMAL METRIC (1)		DRV2605L YZF (DSBGA) (9-PINS)	UNIT
$R_{\theta JA}$	Junction-to-ambient thermal resistance	145.2	°C/W
$R_{\theta JC(top)}$	Junction-to-case (top) thermal resistance	0.9	°C/W
$R_{\theta JB}$	Junction-to-board thermal resistance	105	°C/W
ψ_{JT}	Junction-to-top characterization parameter	5.1	°C/W
ψ_{JB}	Junction-to-board characterization parameter	103.3	°C/W

(1) For more information about traditional and new thermal metrics, see the *Semiconductor and IC Package Thermal Metrics* application report.

 Copyright © 2014–2018, Texas Instruments Incorporated

6.5 Electrical Characteristics

T_A = 25°C, V_{DD} = 3.6 V (unless otherwise noted)

PARAMETER		TEST CONDITIONS	MIN	TYP	MAX	UNIT
$V_{(REG)}$	Voltage at the REG pin			1.83		V
I_{IL}	Digital low-level input current	EN, IN/TRIG, SDA, SCL<br>V_{DD} = 5.2 V , V_I = 0 V			1	µA
I_{IH}	Digital high-level input current	IN/TRIG, SDA, SCL<br>V_{DD} = 5.2 V, V_I = V_{DD}			1	µA
		EN<br>V_{DD} = 5.2 V, V_I = V_{DD}			3.5	
V_{OL}	Digital low-level output voltage	SDAI_{OL}= 4 mA			0.4	V
$R_{(EN\text{-}GND)}$	Digital pull-down resistance	EN<br>V_{DD} = 5.2 V , V_I = V_{DD}		2		MΩ
$I_{(SD)}$	Shutdown current	$V_{(EN)}$ = 0 V		4	7	µA
$I_{I(standby)}$	Standby current	$V_{(EN)}$ = 1.8 V, STANDBY = 1		4.1	7	µA
I_Q	Quiescent current	$V_{(EN)}$ = 1.8 V, STANDBY = 0, no signal		0.5	0.65	mA
Z_I	Input impedance	IN/TRIG to $V_{(CM\_ANA)}$		100		kΩ
$V_{(CM\_ANA)}$	IN/TRIG common-mode voltage (AC-coupled)	AC_COUPLE = 1		0.9		V
$Z_{O(SD)}$	Output impedance in shutdown	OUT+ to GND, OUT– to GND		15		kΩ
$Z_{L(th)}$	Load impedance threshold for over-current detection	OUT+ to GND, OUT– to GND		4		Ω
$I_{(BAT\_AV)}$	Average battery current during operation	Duty cycle = 90%, LRA mode, no load		2.4	3.5	mA
		Duty cycle = 90%, ERM mode, no load		2.3	3.5	

6.6 Timing Requirements

T_A = 25°C, V_{DD} = 3.6 V (unless otherwise noted)

			MIN	NOM	MAX	UNIT
$f_{(SCL)}$	Frequency at the SCL pin with no wait states				400	kHz
$t_{w(H)}$	Pulse duration, SCL high	See Figure 1.	0.6			µs
$t_{w(L)}$	Pulse duration, SCL low		1.3			µs
$t_{su(1)}$	Setup time, SDA to SCL		100			ns
$t_{h(1)}$	Hold time, SCL to SDA		10			ns
$t_{(BUF)}$	Bus free time between stop and start condition	See Figure 2.	1.3			µs
$t_{su(2)}$	Setup time, SCL to start condition		0.6			µs
$t_{h(2)}$	Hold time, start condition to SCL		0.6			µs
$t_{su(3)}$	Setup time, SCL to stop condition		0.6			µs

6.7 Switching Characteristics

T_A = 25°C, V_{DD} = 3.6 V (unless otherwise noted)

PARAMETER		TEST CONDITIONS	MIN	TYP	MAX	UNIT
$t_{(start)}$	Start-up time	Time from the GO bit or external trigger command to output signal		0.7		ms
		Time from EN high to output signal (PWM/Analog Modes)		1.5		
$f_{O(PWM)}$	PWM Output Frequency		19.5	20.5	21.5	kHz

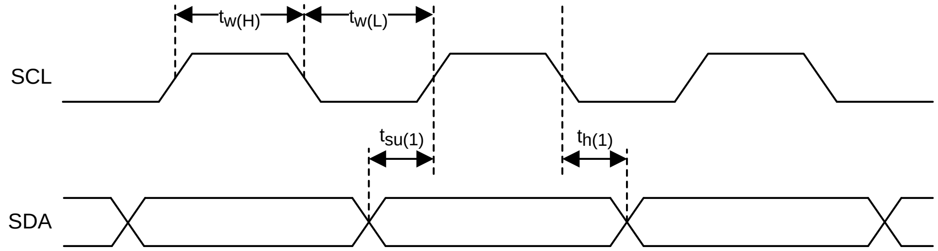

Figure 1. SCL and SDA Timing

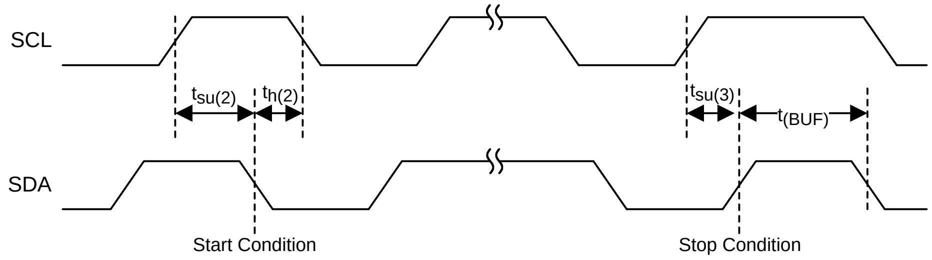

Figure 2. Timing for Start and Stop Conditions

Copyright © 2014–2018, Texas Instruments Incorporated

6.8 Typical Characteristics

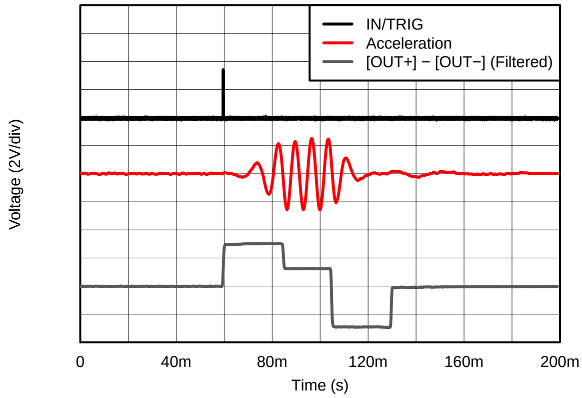

V_{DD} = 3.6 V ERM open loop
Strong click - 60% External edge trigger

Figure 3. ERM Click with and Without Braking (ROM)

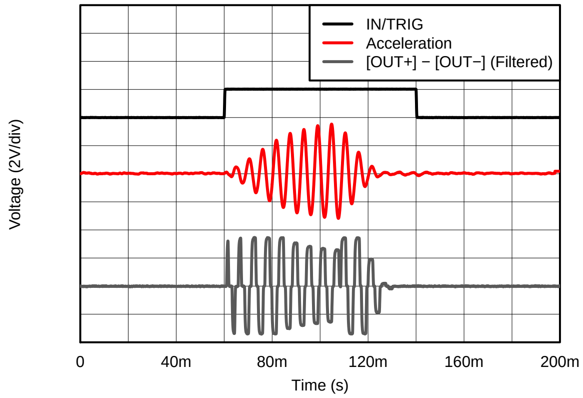

V_{DD} = 3.6 V LRA closed loop
Strong click - 100% External level trigger

Figure 4. LRA Click with Braking (ROM)

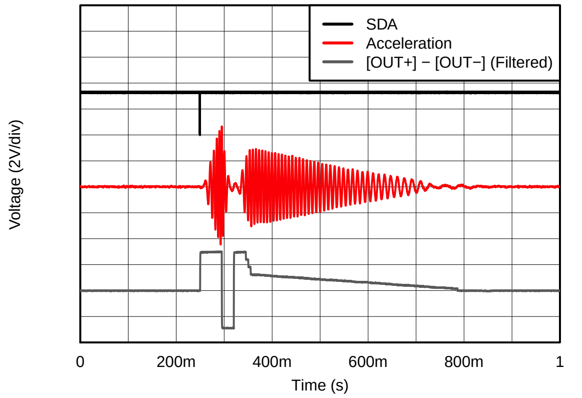

V_{DD} = 3.6 V ERM open loop
Sequence = 0x01, 0x48 Internal trigger

Figure 5. ERM Click-Bounce (ROM)

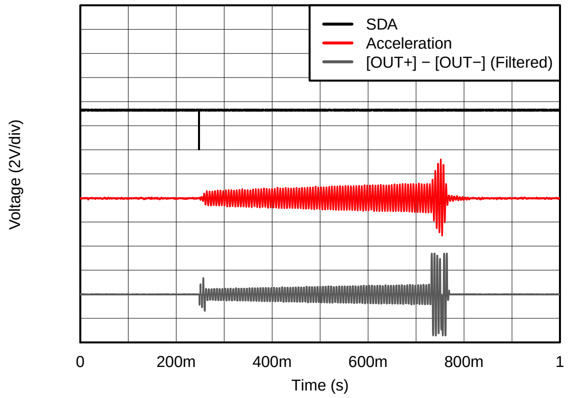

V_{DD} = 3.6 V LRA closed loop
Transition click 1 - 100% Internal trigger

Figure 6. LRA Transition-Click (ROM)

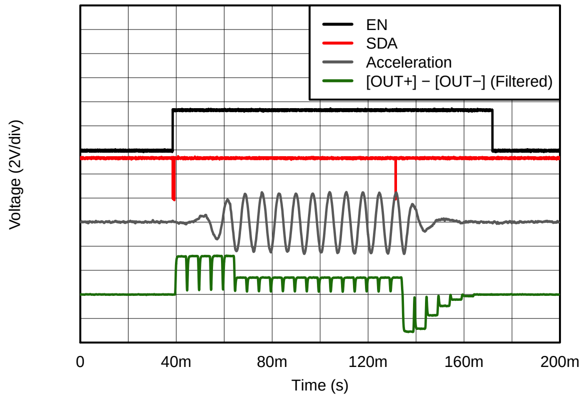

V_{DD} = 3.6 V ERM closed loop RTP Mode

Figure 7. ERM Buzz (RTP)

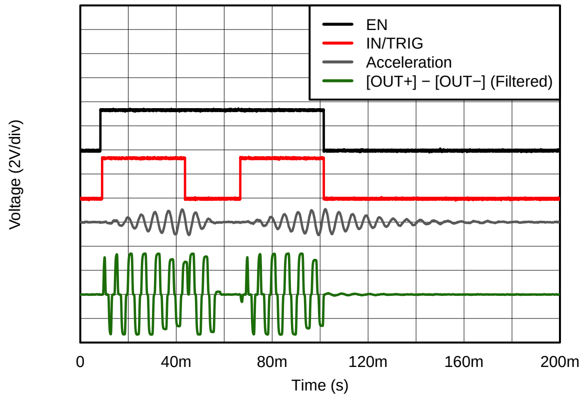

V_{DD} = 3.6 V LRA closed loop PWM Mode

Figure 8. LRA Click With and Without Braking (PWM)

Typical Characteristics (continued)

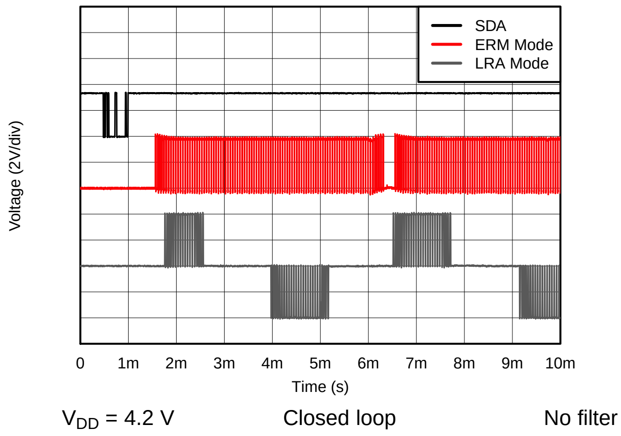

Figure 9. Startup Latency for ERM and LRA

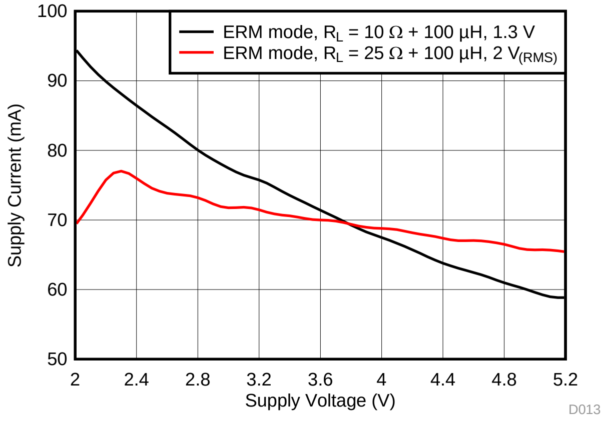

Figure 10. Supply Current vs Supply Voltage (Full Vibration)

7 Parameter Measurement Information

7.1 Test Setup for Graphs

To capture the graphs displayed in the *Typical Characteristics* section, the following first-order RC-filter setup was used with the exception of the waveform in Figure 9 which was captured without any output filter. This filter is recommended when viewing output signals on an oscilloscope because output PWM modulation is present in all modes. Ensure that effective impedance of the filter is not too low because the closed-loop and auto resonance-tracking features can be affected. Therefore, TI recommends that this exact filter be used for output measurement. Most oscilloscopes have an input impedance of 1 MΩ on each channel and therefore have an approximately 1% loss in measured amplitude because of the voltage-divider effect with the filter.

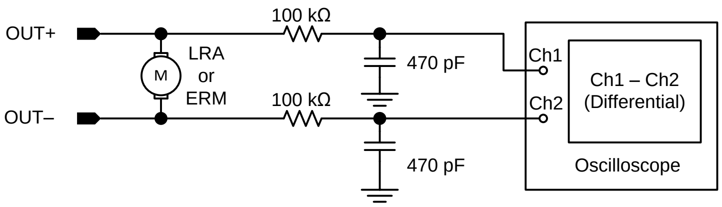

Figure 11. Test Setup

7.1.1 Default Test Conditions

- V_{DD} = 3.6 V, unless otherwise noted.
- Real actuators (as opposed to modeled actuators) were used as loads for both ERM and LRA modes with exception of the *Supply Voltage vs Supply Current (Full Vibration)* waveform in Figure 10, which used passive RL (resistance in series with an inductance) loads for test repeatability. Real actuators vary widely in supply currents because of variation in back-EMF voltages. Because real actuators have back EMF, the real supply current is generally less than what is shown in the waveform because of the reduction in the apparent load impedance. Therefore, the curve shows the worst-case current.
- All ERM library waveforms were taken with Library A in open-loop mode
- All LRA library waveforms were taken with the LRA Library in closed-loop mode
- All traces are 2 V/div except for the accelerometer traces
- All accelerometer traces are 0.87 g/div except for the *LRA Click with and without Braking (PWM)* curve in Figure 8, which is 1.74 g/div.

Copyright © 2014–2018, Texas Instruments Incorporated

8 Detailed Description

8.1 Overview

The DRV2605L device is a low-voltage haptic driver that relies on the back-EMF produced by an actuator to provide a closed-loop system that offers extremely flexible control of LRA and ERM actuators over a shared I²C-compatible bus or PWM input signal. This schema helps improve actuator performance in terms of acceleration consistency, start time, and brake time.

The improved smart-loop architecture inside the DRV2605L device provides effortless auto-resonant drive for LRA, as well as feedback-optimized ERM drive allowing for automatic overdrive and braking. These features create a simplified input waveform paradigm as well as reliable motor control and consistent motor performance. The DRV2605L device also features an automatic transition to open-loop operation in the event that an LRA actuator is not generating a valid back-EMF voltage and automatic synchronization with the LRA when the LRA is generating a valid back-EMF voltage. The DRV2605L device also allows for open-loop driving by using internally-generated PWM. Additionally, the audio-to-vibe mode automatically converts an audio input signal to meaningful haptic effects.

The DRV2605L device offers a licensed version of TouchSense 2200 software from Immersion which eliminates the requirement to design haptic waveforms because the software includes over 100 licensed effects (6 ERM libraries and 1 LRA library) and audio-to-vibe features. The waveforms can be instantly played back through an I²C or can be triggered through a hardware trigger pin. Additionally, the real-time playback mode allows the host processor to bypass the library playback engine and play waveforms directly from the host through the I²C.

The DRV2605L device features a trinary-modulated output stage that provides more efficiency than linear-based output drivers.

8.2 Functional Block Diagram

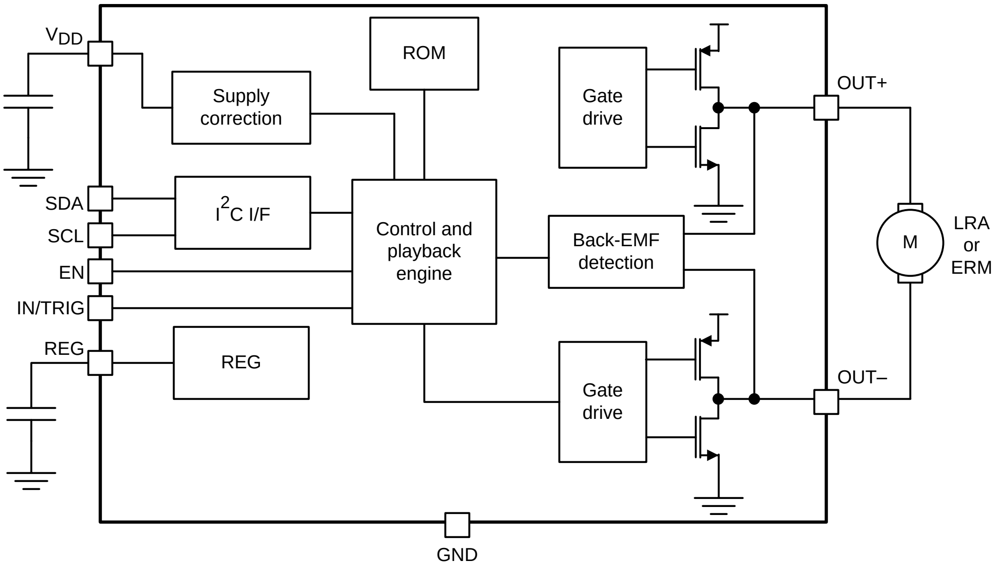

8.3 Feature Description

8.3.1 Support for ERM and LRA Actuators

The DRV2605L device supports both ERM and LRA actuators. The ERM_LRA bit in register 0x1A must be configured to select the type of actuator that the device uses.

8.3.2 Smart-Loop Architecture

The smart-loop architecture is an advanced closed-loop system that optimizes the performance of the actuator and allows for failure detection. The architecture consists of automatic resonance tracking and reporting (for an LRA), automatic level calibration, accelerated startup and braking, diagnostics routines, and other proprietary algorithms.

8.3.2.1 Auto-Resonance Engine for LRA

The DRV2605L auto-resonance engine tracks the resonant frequency of an LRA in real time, effectively locking onto the resonance frequency after half of a cycle. If the resonant frequency shifts in the middle of a waveform for any reason, the engine tracks the frequency from cycle to cycle. The auto-resonance engine accomplishes the tracking by constantly monitoring the back-EMF of the actuator. The auto-resonance engine is not affected by the auto calibration process, which is only used for level calibration. No calibration is required for the auto resonance engine. See the *Auto-Resonance Engine Programming for the LRA* section for auto-resonance engine programming information.

8.3.2.2 Real-Time Resonance-Frequency Reporting for LRA

The smart-loop architecture makes the resonant frequency of the LRA available through I^2C (see the *LRA Resonance Period (Address: 0x22)* section). Because frequency reporting occurs in real time, the frequency must be polled while the DRV2605L device synchronizes with the LRA. The data should not be polled when the actuator is idle or braking.

8.3.2.3 Automatic Switch to Open-Loop for LRA

In the event that an LRA produces a non-valid back-EMF signal, the DRV2605L device automatically switches to open-loop operation and continues to deliver energy to the actuator in overdrive mode at a default and configurable frequency. Use Equation 1 to calculate the default frequency. If the LRA begins to produce a valid back-EMF signal, the auto-resonance engine automatically takes control and continues to track the resonant frequency in real time. When synchronized, the mode enjoys all of the benefits that the smart-loop architecture has to offer.

$$f_{(LRA\_NO\text{-}BEMF)} \approx \frac{1}{2 \times \left(t_{(DRIVE\_TIME[4:0])} - t_{(ZC\_DET\_TIME[1:0])}\right)} \tag{1}$$

The DRV2605L device offers an automatic transition to open-loop mode without the re-synchronization option. The feature is enabled by setting the LRA_AUTO_OPEN_LOOP bit in register 0x1F. The transition to open-loop mode only occurs when the driver fails to synchronize with the LRA. The AUTO_OL_CNT[1:0] bit in register 0x1F can be adjusted to set the amount of non-synchronized cycles allowed before the transition to the open-loop mode. Use Equation 2 to calculate the open-loop frequency. The open-loop mode does not receive benefits from the smart-loop architecture, such as automatic overdrive and braking.

$$f_{(LRA\_OL)} = \frac{1}{OL\_LRA\_PERIOD[6:0] \times 98.49 \times 10^{-6}} \tag{2}$$

8.3.2.4 Automatic Overdrive and Braking

A key feature of the DRV2605L is the smart-loop architecture which employs actuator feedback control for both ERMs and LRAs. The feedback control desensitizes the input waveform from the motor-response behavior by providing automatic overdrive and automatic braking.

Copyright © 2014–2018, Texas Instruments Incorporated

Feature Description (continued)

An open-loop haptic system typically drives an overdrive voltage at startup that is higher than the steady-state rated voltage of the actuator to decrease the startup latency of the actuator. Likewise, a braking algorithm must be employed for effective braking. When using an open-loop driver, these behaviors must be contained in the input waveform data. Figure 12 shows how two different ERMs with different startup behaviors (Motor A and Motor B) can both be driven optimally by the smart-loop architecture with a simple input for both motors. The smart-loop architecture works equally well for LRAs with a combination of feedback control and an auto-resonance engine.

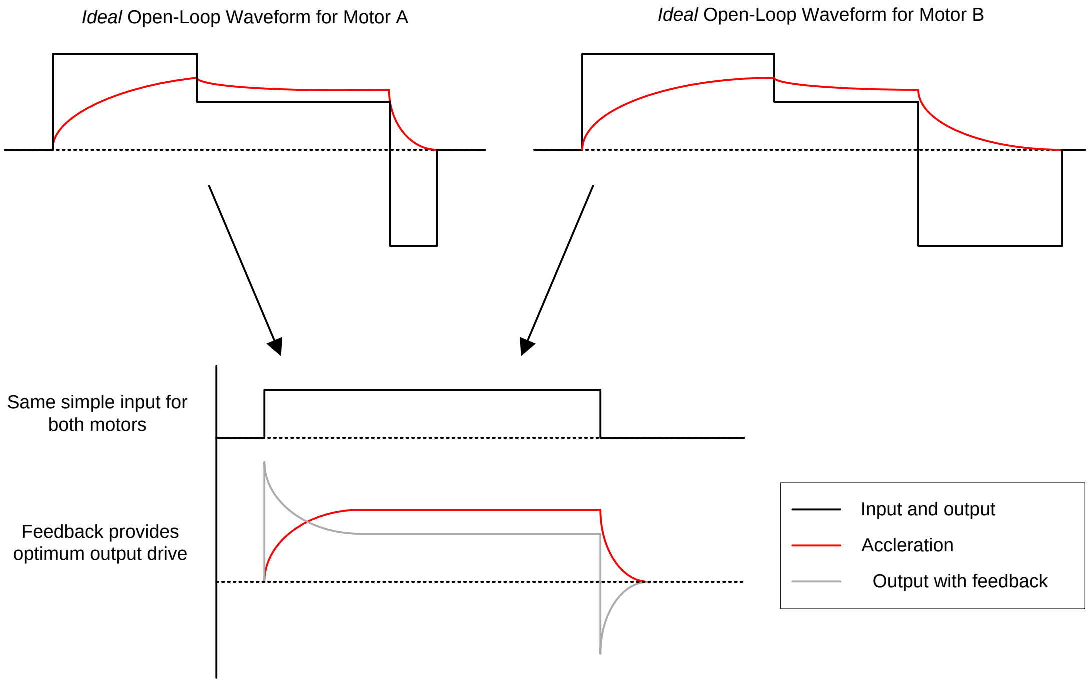

Figure 12. Waveform Simplification With Smart Loop

8.3.2.4.1 Startup Boost

To reduce the actuator start-time performance, the DRV2605L device has an overdrive boost feature that applies higher loop gain to transient response of the actuator. The STARTUP_BOOST bit enables the feature.

8.3.2.4.2 Brake Factor

To reduce the actuator brake-time performance, the DRV2605L device provides a means to increase the gain ratio between braking and driving gain. Higher feedback-gain ratios reduce the brake time, however, the gain ratios also reduce the stability of the closed-loop system. The FB_BRAKE_FACTOR[2:0] bits can be adjusted to set the brake factor.

8.3.2.4.3 Brake Stabilizer

To improve brake stability at high brake-factor gain ratios, the DRV2605L device has a brake-stabilizer mechanism that automatically reduces the loop gain when the braking is near completion. The BRAKE_STABILIZER bit enables the feature.

Feature Description (continued)

8.3.2.5 Automatic Level Calibration

The smart-loop architecture uses actuator feedback by monitoring the back-EMF behavior of the actuator. The level of back-EMF voltage can vary across actuator manufacturers because of the specific actuator construction. Auto calibration compensates for the variation and also performs scaling for the desired actuator according to the specified rated voltage and overdrive clamp-register settings. When auto calibration is performed, a 100% signal level at any of the DRV2605L input interfaces supplies the rated voltage to the actuator at steady-state. The feedback allows the output level to increase above the rated voltage level for automatic overdrive and braking, but without allowing the output level to exceed the programmable overdrive clamp voltage.

In the event where the automatic level-calibration routine fails, the DIAG_RESULT bit in register 0x00 is asserted to flag the problem. Calibration failures are typically fixed by adjusting the registers associated with the automatic level-calibration routine or, for LRA actuators, the registers associated with the automatic-resonance detection engine. See the *Device and Documentation Support* section for automatic-level calibration programming.

8.3.2.5.1 Automatic Compensation for Resistive Losses

The DRV2605L device automatically compensates for resistive losses in the driver. During the automatic level-calibration routine, the impedance of the actuator is checked and the compensation factor is determined and stored in the A_CAL_COMP[7:0] bit.

8.3.2.5.2 Automatic Back-EMF Normalization

The DRV2605L device automatically compensates for differences in back-EMF magnitude between actuators. The compensation factor is determined during the automatic level-calibration routine and the factor is stored in the A_CAL_BEMF[7:0] bit.

8.3.2.5.3 Calibration Time Adjustment

The duration of the automatic level-calibration routine has an impact on accuracy. The impact is highly dependent on the start-time characteristic of the actuator. The auto-calibration routine expects the actuator to have reached a steady acceleration before the calibration factors are calculated. Because the start-time characteristic can be different for each actuator, the AUTO_CAL_TIME[1:0] bit can change the duration of the automatic level-calibration routine to optimize calibration performance.

8.3.2.5.4 Loop-Gain Control

The DRV2605L device allows the user to control how fast the driver attempts to match the back-EMF (and thus motor velocity) and the input signal level. Higher loop-gain (or faster settling) options result in less-stable operation than lower loop gain (or slower settling). The LOOP_GAIN[1:0] bit controls the loop gain.

8.3.2.5.5 Back-EMF Gain Control

The BEMF_GAIN[1:0] bit sets the analog gain for the back-EMF amplifier. The auto-calibration routine automatically populates the bit with the most appropriate value for the actuator.

Modifying the SAMPLE_TIME[1:0] bit also adjusts the back-EMF gain. The higher the sample time, the higher the gain.

By default, the back-EMF is sampled once during a period. In the event that a twice per-period sampling is desired, assert the LRA_DRIVE_MODE bit.

8.3.2.6 Actuator Diagnostics

The DRV2605L device is capable of determining whether the actuator is not present (open) or shorted. If a fault is detected during the diagnostic process, the DIAG_RESULT bit is asserted.

8.3.2.7 Automatic Re-Synchronization

For the LRA, the DRV2605L device features an automatic re-synchronization mode which automatically pushes the actuator in the correct direction when a waveform begins playing while the actuator is moving. If the actuator is at rest when the waveform begins, the DRV2605L device drives in the default direction.

Copyright © 2014–2018, Texas Instruments Incorporated

Feature Description (continued)

8.3.3 Open-Loop Operation for LRA

In the event that open-loop operation is desired (such as for off-resonance driving) the DRV2605L device includes an open-loop LRA drive mode that is available through the PWM input or through the digital interface.

When using the PWM input in open-loop mode, the DRV2605L device employs a fixed divider that observes the PWM signal and commutates the output drive signal at the PWM frequency divided by 128. To accomplish LRA drive, the host should drive the PWM frequency at 128 times the desired operating frequency.

When activated, the digital open-loop mode is available for pre-stored waveforms as well as for RTP mode. The OL_LRA_PERIOD bit in register 0x20 programs the operating frequency, which is derived from the PWM output frequency, $f_{O(PWM)}$. Use Equation 1 to calculate the driving frequency. The open-loop mode does not receive the benefits of the smart-loop architecture.

8.3.4 Open-Loop Operation for ERM

The DRV2605L device offers ERM open-loop operation through the PWM input. The output voltage is based on the duty cycle of the provided PWM signal, where the OD_CLAMP[7:0] bit in register 0x17 sets the full-scale amplitude. For details see the *Rated Voltage Programming* section.

8.3.5 Flexible Front-End Interface

The DRV2605L device offers multiple ways to launch and control haptic effects. The MODE[2:0] bit in register 0x01 is used to select the interface mode.

8.3.5.1 PWM Interface

When the DRV2605L device is in PWM interface mode, the device accepts PWM data at the IN/TRIG pin. The DRV2605L device drives the actuator continuously in PWM interface mode until the user sets the device to standby mode or to enter another interface mode. In standby mode, the strength of vibration is determined by the duty cycle.

For the LRA, the DRV2605L device automatically tracks the resonance frequency unless the LRA_OPEN_LOOP bit in register 0x1D is set. If the LRA_OPEN_LOOP bit is set, the LRA is driven according to the frequency of the PWM input signal. Specifically, the driving frequency is the PWM frequency divided by 128.

8.3.5.2 Internal Memory Interface

The DRV2605L device has seven internal-ROM libraries designed by Immersion called TS2200. The first five libraries and the last library are specifically tuned for six categories of ERMs operated in open-loop mode (see Table 1). Library 6 is a closed-loop library tuned for LRAs. The library selection occurs through register 0x03 (see the *Library Selection (Address: 0x03)* section).

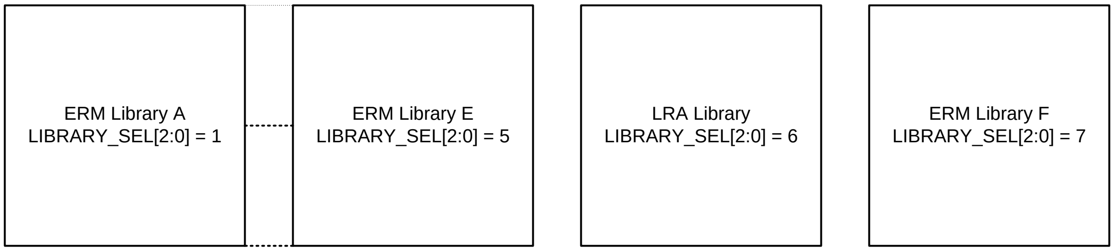

Figure 13. Library Selection

Feature Description (continued)

Table 1. ERM Library Table

LIBRARY	RATED VOLTAGE	OVERDRIVE VOLTAGE	RISE TIME	BRAKE TIME
A	1.3 V	3 V	40 ms to 60 ms	20 ms to 40 ms
B	3 V	3 V	40 ms to 60 ms	5 ms to 15 ms
C	3 V	3 V	60 ms to 80 ms	10 ms to 20 ms
D	3 V	3 V	100 ms to 140 ms	15 ms to 25 ms
E	3 V	3 V	> 140 ms	> 30 ms
F	4.5 V	5 V	35 ms to 45 ms	10 ms to 20 ms

8.3.5.2.1 Waveform Sequencer

The waveform sequencer queues waveform identifiers for playback. Eight sequence registers queue up to eight waveforms for sequential playback. A waveform identifier is an integer value referring to the index position of a waveform in the ROM library. Playback begins at register address 0x04 when the user asserts the GO bit (register 0x0C). When playback of that waveform ends, the waveform sequencer plays the waveform identifier held in register 0x05 if the next waveform is non-zero. The waveform sequencer continues in this way until it reaches an identifier value of zero or until all eight identifiers are played (register addresses 0x04 through 0x0B), whichever scenario is reached first.

The waveform identifier range is 1 to 127. The MSB of each sequence register can implement a delay between sequence waveforms. When the MSB is high, bits [6:0] indicate the length of the wait time. The wait time for that step then becomes WAV_FRM_SEQ[6:0] × 10 ms.

8.3.5.2.2 Library Parameterization

The ROM waveforms are augmented by the time offset registers (registers 0x0D to 0x10). The augmentation occurs only for the ROM waveforms and not for the other interfaces (such as PWM and RTP). The purpose of the functionality is to add *time stretching* (or time shrinking) to the waveform. This functionality is useful for customizing the entire library of waveforms for a specific actuator rise time and fall time.

The time parameters that can be stretched or shrunk include:

ODT Overdrive time

SPT Sustain positive time

SNT Sustain Negative Time

BRT Brake Time

The time values are additive offsets and are 8-bit signed values. The default offset of the time values is 0. Positive values add and negative values subtract from the time value of the effect that is currently played. The most positive value in the waveform is automatically interpreted as the overdrive time, and the most negative value in the waveform is automatically interpreted as the brake time. The time-offset parameters are applied to both voltage-time pairs and linear ramps. For linear ramps, linear interpolation is stretched (or shrunk) over the two operative points for the period (see Equation 3).

$$t + t_{(ofs)} \qquad (3)$$

where

- $t_{(ofs)}$ is the time offset

Changing the playback interval can also manipulate the waveforms stored in memory. Each waveform in memory has a granularity of 5 ms. If the user desires greater granularity, a 1-ms playback interval can be obtained by asserting the PLAYBACK_INTERVAL bit in register 0x1F.

Copyright © 2014–2018, Texas Instruments Incorporated

8.3.5.3 Real-Time Playback (RTP) Interface

The real-time playback mode is a simple, single 8-bit register interface that holds an amplitude value. When real-time playback is enabled, the real-time playback register is sent directly to the playback engine. The amplitude value is played until the user sends the device to standby mode or removes the device from RTP mode. The RTP mode operates exactly like the PWM mode except that the user enters a register value over the I^2C rather than a duty cycle through the input pin. Therefore, any API (application-programming interface) designed for use with a PWM generator in the host processor can write the data values over the I^2C rather than writing the data values to the host timer. This ability frees a timer in the host while retaining compatibility with the original software.

For the LRA, the DRV2605L device automatically tracks the resonance frequency unless the LRA_OPEN_LOOP bit is set (in register 0x1D). If the LRA_OPEN_LOOP bit is set, the LRA is driven according to the open-loop frequency set in the OL_LRA_PERIOD[6:0] bit in register 0x20.

8.3.5.4 Analog Input Interface

When the DRV2605L device is in analog-input interface mode, the device accepts an analog voltage at the IN/TRIG pin. The DRV2605L device drives the actuator continuously in analog-input interface mode until the user sets the device to standby mode or to enter another interface mode. The reference voltage in standby mode is 1.8 V. Therefore, the 1.8-V reference voltage is interpreted as a 100% input value. A reference voltage of 0.9 V is interpreted as a 50% input value and a reference voltage of 0 V is interpreted as a 0% input value. The input value in standby mode is analogous to the duty-cycle percentage in PWM mode.

For the LRA, the DRV2605L automatically tracks the resonance frequency unless the LRA_OPEN_LOOP bit is set (in register 0x1D). If the LRA_OPEN_LOOP bit is set, the LRA is driven according to the open-loop frequency set in OL_LRA_PERIOD[6:0] bit in register 0x20.

8.3.5.5 Audio-to-Vibe Interface

The DRV2605L device features an audio-to-vibe mode that converts an audio input signal into meaningful haptic effects using the Immersion audio-to-vibe technology. Audio-to-Vibe mode adds a vibratory *bass extension* to portable devices which allows users to feel the audio and visual content. Audio-to-Vibe mode is a key feature because it allows for existing applications to include haptic sensations without requiring additional software drivers. Additionally, event-driven audio effects generated within an operating system can be used to automatically provide a product with haptic sensations. See the *Waveform Playback Using Audio-to-Vibe Mode* section for details.

8.3.5.6 Input Trigger Option

The DRV2605L device includes continuous haptic modes (such as PWM and RTP mode) as well as triggered modes (such as the internal memory interface). The haptic effects in the continuous haptic modes begin as soon as the device enters the mode and stop when the device goes into standby mode or exits the continuous haptic mode. For the triggered mode, the DRV2605L device has a variety of trigger options that are explained in this section.

In the continuous haptic modes, the IN/TRIG pin provides external trigger control of the GO bit, which allows GPIO control to fire ROM waveforms. The external trigger control can provide improved latencies in systems where a significant delay exists between the desired effect time and the time a GO command can be sent over the I^2C interface.

NOTE

The triggered effect must already be selected to take advantage of the lower latency. This option works best for accelerating a pre-queued high-priority effect (such as a button press) or for the repeated firing of the same effect (such as scrolling).

8.3.5.6.1 I^2C Trigger

Setting the GO bit (in register 0x0C) launches the waveform. The user can cancel the launching of the waveform by clearing the GO bit.

8.3.5.6.2 Edge Trigger

A low-to-high transition on the IN/TRIG pin sets the GO bit. The playback sequence indicated in the waveform sequencer plays as normal. The user can cancel the transaction by clearing the GO bit. An additional low-to-high transition while the GO bit is high also cancels the transaction which clears and resets the GO bit. Clearing the trigger pin (high-to-low transition) does nothing, therefore the user can send a short pulse without knowing how long the waveform is. The pulse width should be at least 1 µs to ensure detection.

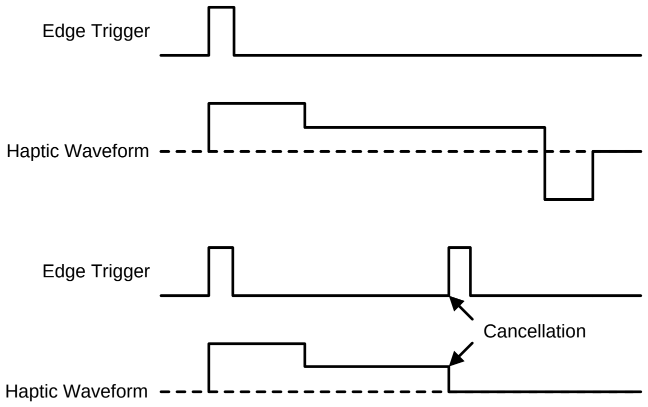

Figure 14. Edge Trigger Mode

8.3.5.6.3 Level Trigger

The actions of the GO bit directly follow the IN/TRIG pin. When the IN/TRIG pin is high, the GO bit is high. When the IN/TRIG pin goes low, the GO bit clears. Therefore, a falling edge cancels the transaction. The level trigger can implement a GPIO-controlled buzz on-off controller if an appropriately long waveform is selected. The user must hold the IN/TRIG high for the entire duration of the waveform to complete the effect.

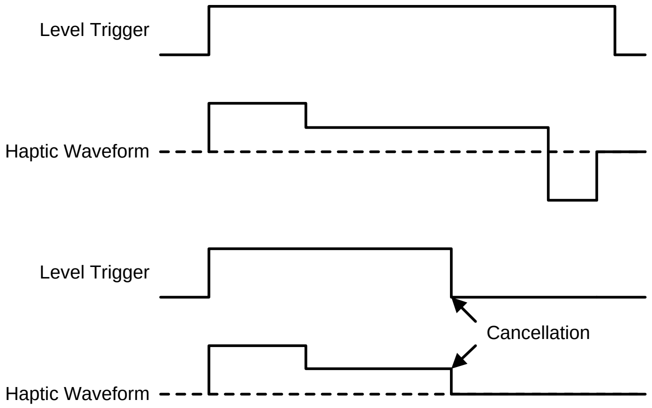

Figure 15. Level Trigger Mode

8.3.5.7 Noise Gate Control

When an actuator is driven with an analog or PWM signal, noise in the line can cause the actuator to vibrate unintentionally. For that reason, the DRV2605L device features a noise gate that filters out any voltage smaller than a particular threshold. The NG_THRESH[1:0] bit in register 0x1D controls the threshold.

8.3.6 Edge Rate Control

The DRV2605L output driver implements edge rate control (ERC). The ERC ensures that the rise and fall characteristics of the output drivers do not emit levels of radiation that could interfere with other circuitry common in mobile and portable platforms. Because of ERC most system do not require external output filters, capacitors, or ferrite beads.

Copyright © 2014–2018, Texas Instruments Incorporated

8.3.7 Constant Vibration Strength

The DRV2605L PWM input uses a digital level-shifter. Therefore, as long as the input voltage meets the V_{IH} and V_{IL} levels, the vibration strength remains the same even if the digital levels vary. The DRV2605L device also features power-supply feedback. If the supply voltage drifts over time (because of battery discharge, for example), the vibration strength remains the same as long as enough supply voltage is available to sustain the required output voltage.

8.3.8 Battery Voltage Reporting

During playback, the DRV2605L device provides real-time voltage measurement of the V_{DD} pin. The VBAT[7:0] bit located in register 0x21 provides this information.

8.3.9 One-Time Programmable (OTP) Memory for Configuration

The DRV2605L device contains nonvolatile, on-chip, OTP memory for specific configuration parameters. When written, the DRV2605L device retains the device settings in registers 0x16 through 0x1A including after power cycling. This retention allows the user to account for small variations in actuator manufacturing from unit to unit as well as to shorten the device-initialization process for device-specific parameters such as actuator type, actuator-rated voltage, and other parameters. An additional benefit of OTP is that the DRV2605L memory can be customized at the device-test level without driving changes in the device software.

8.3.10 Low-Power Standby

Setting the device to standby reduces the idle power consumption without resetting the registers. In Low-Power Standby mode, the DRV2605L device features a fast turnon time when it is requested to play a waveform.

8.3.11 I^2C Watchdog Timer

If an I^2C stops unexpectedly, the possibility exists for the I^2C protocol to remain in a *hanged* state. To allow for the recovery of the communication without having to power cycle the device, the DRV2605L device includes an automatic watchdog timer that resets the I^2C protocol without user intervention after 4.33 ms. This behavior happens in all conditions except in standby mode. If the I^2C stops unexpectedly during standby mode, the only way to recover communication is by power-cycling the device.

8.3.12 Device Protection

8.3.12.1 Thermal Protection

The DRV2605L device has thermal protection that causes the device to shut down if it becomes too hot. In the event where the thermal protection kicks in, the DRV2605L device asserts a flag (bit OVER_TEMP in register 0x00) to notify the host processor.

8.3.12.2 Overcurrent Protection of the Actuator

If the impedance at the output pin of the DRV2605L device is too low, the device latches the over-current flag (OC_DETECT bit in register 0x00) and shuts down. The device periodically monitors the status of the short and remains in this condition until the short is removed. When the short is removed, the DRV2605L device restarts in the default state.

8.3.12.3 Overcurrent Protection of the Regulator

The DRV2605L device has an internal regulator that powers a portion of the system. If a short occurs at the output of the REG pin, an internal overcurrent protection circuit is enabled and limits the current.

During a REG short, the device is not functional. When the short is removed, the DRV2605L device automatically resets to default conditions.

Copyright © 2014–2018, Texas Instruments Incorporated

8.3.12.4 Brownout Protection

The DRV2605L device has on-chip brownout protection. When activated, a reset signal is issued that returns the DRV2605L device to the initial default state. If the regulator voltage $V_{(REG)}$ goes below the brownout protection threshold ($V_{(BOT)}$) the DRV2605L device automatically shuts down. When $V_{(REG)}$ returns to the typical output voltage (1.8 V) the DRV2605L device returns to the initial device state. The brownout protection threshold ($V_{(BOT)}$) is typically at 0.84 V.

The previously described behavior has one exception. The brownout circuit is designed to tolerate fast brownout conditions as shown by Case 1 in Figure 16. If the V_{DD} ramp-up rate is slower than 3.6 kV/s, then the device can fall into an unknown state. In such a situation, to return to the initial default state the device must be power-cycled with a V_{DD} ramp-up rate that is faster than 3.6 kV/s.

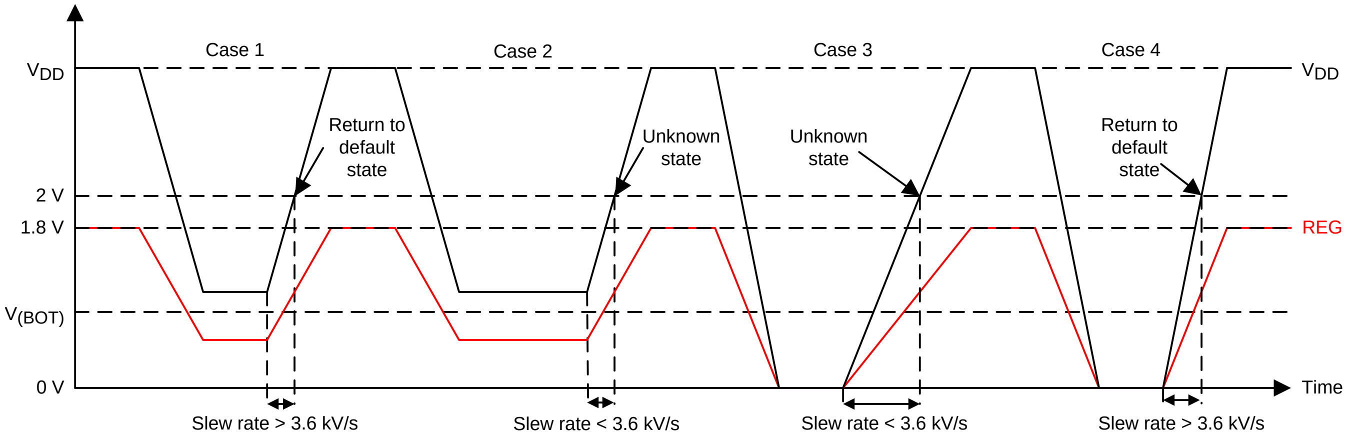

Figure 16. Brownout Behavior

8.4 Device Functional Modes

8.4.1 Power States

The DRV2605L device has three different power states which allow for different power-consumption levels and functions. Figure 17 shows the transition in to and out of each state.

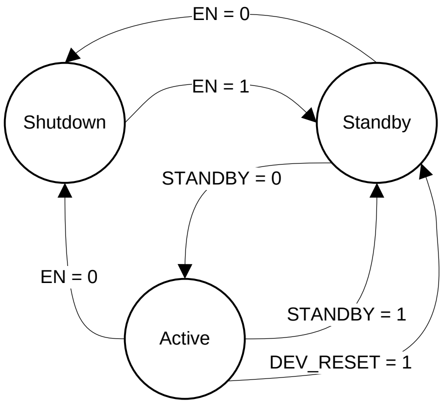

Figure 17. Power-State Transition Diagram

8.4.1.1 Operation With V_{DD} < 2 V (Minimum V_{DD})

Operating the device with a V_{DD} value below 2 V is not recommended.

8.4.1.2 Operation With V_{DD} > 5.5 V (Absolute Maximum V_{DD})

The DRV2605L device is designed to operate at up to 5.2 V, with an absolute maximum voltage of 5.5 V. If exposed to voltages above 5.5 V, the device can suffer permanent damage.

Copyright © 2014–2018, Texas Instruments Incorporated

Device Functional Modes (continued)

8.4.1.3 Operation With EN Control

The EN pin of the DRV2605L device gates the active operation. When the EN pin is logic high, the DRV2605L device is active. When the EN pin is logic low, the device enters the shutdown state, which is the lowest power state of the device. The device registers are not reset. The EN pin operation is particularly useful for constant-source PWM and analog input modes to maintain compatibility with non-I^2C device signaling. The EN pin must be high to write I^2C device registers. However, if the EN pin is low the DRV2605L device can still acknowledge (ACK) during an I^2C transaction, however, no read or write is possible. To completely reset the device to the powerup state, set the DEV_RESET bit in register 0x01.

8.4.1.4 Operation With STANDBY Control

The STANDBY bit in register 0x01 forces the device in an out of the standby state. The STANDBY bit is asserted by default. When the STANDBY bit is asserted, the DRV2605L device goes into a low-power state. In the standby state the device retains register values and the ability to have I^2C communication. The properties of the standby state also feature a fast turn, wake up, and play, on-time. Asserting the STANDBY bit has an immediate effect. For example, if a waveform is played, it immediately stops when the STANDBY bit is asserted.

Clear the STANDBY bit to exit the standby state (and go to the ready state).

8.4.1.5 Operation With DEV_RESET Control

The DEV_RESET bit in register 0x01 performs the equivalent of power cycling the device. Any playback operations are immediately interrupted, and all registers are reset to the default values. The Dev_Reset bit automatically-clears after the reset operation is complete.

8.4.1.6 Operation in the Active State

In the active state, the DRV2605L device has I^2C communication and is capable of playing waveforms, running calibration, and running diagnostics. These operations are referred to as *processes*. Figure 18 shows the flow of starting, or *firing*, a process. Notice that the GO signal fires the processes. Note that the GO signal is not the same as the GO bit. Figure 19 shows a diagram of the GO-signal behavior.

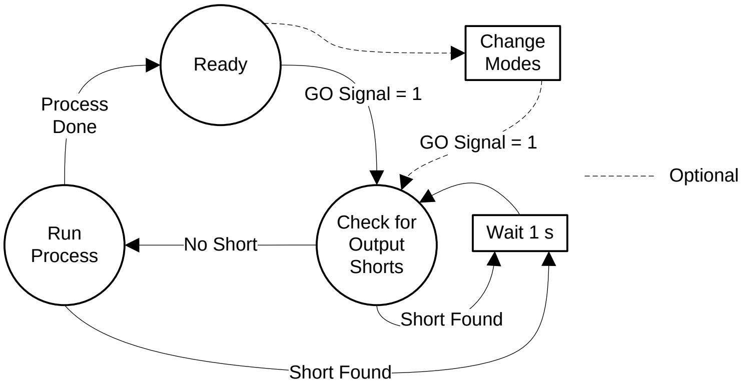

Note: If an output short is present before a waveform is played, changing modes (with the MODE[2:0] bit in register 0x01) is required to resume normal playback.

Figure 18. Diagram of Active States

8.4.2 Changing Modes of Operation

The DRV2605L has multiple modes for playing waveforms, as well as a calibration mode and a diagnostic mode. Table 2 lists the available modes.

Table 2. Mode Selection Table

MODE	MODE[2:0]	N_PWM_ANALOG
Internal trigger mode	0	X
External Trigger mode (edge)	1	X

Table 2. Mode Selection Table (continued)

MODE	MODE[2:0]	N_PWM_ANALOG
External trigger mode (level)	2	X
Analog input mode	3	0
PWM mode	3	1
Audio-to-vibe mode	4	X
RTP mode	5	X
Diagnostics mode	6	X
Calibration mode	7	X

8.4.3 Operation of the GO Bit

The GO bit is the primary way to assert the GO signal, which fires processes in the DRV2605L device. The primary purpose of the GO bit is to fire the playback of the waveform identifiers in the waveform sequencer (registers 0x04 to 0x0B). However, The GO bit can also fire the calibration or diagnostics processes.

When using the GO bit to play waveforms in internal trigger mode, the GO bit is asserted by writing 0x01 to register 0x0C. In this case, the GO bit can be thought of as a *software trigger* for haptic waveforms. The GO bit remains high until the playback of the haptic waveform sequence is complete. Clearing the GO bit during waveform playback cancels the waveform sequence. The GO bit can also be asserted by the external trigger when in external trigger mode. The GO bit in register 0x0C mirrors the state of the external trigger.

Setting RTP mode , PWM mode, or audio-to-vibe mode also sets the GO bit. However, setting the GO bit in this way has no impact on the GO bit located in register 0x0C.

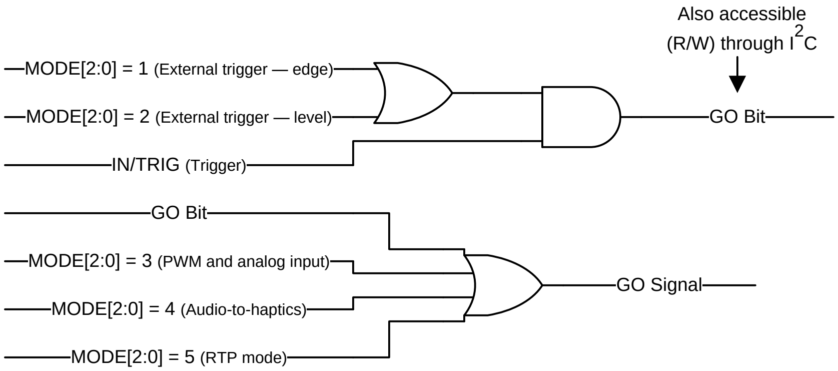

Figure 19. GO-Signal Logic

8.4.4 Operation During Exceptional Conditions

This section lists different exceptional conditions and the ways that the DRV2605L device operates during these conditions. This section also describes how the device goes into and out of these states.

8.4.4.1 Operation With No Actuator Attached

In LRA closed-loop mode, if a waveform is played without an actuator connected to the OUT+ and OUT– pins, the output pins toggle. However, the toggling frequency is not predictable. In LRA open-loop mode, the output pins toggle at the specified open-loop frequency.

8.4.4.2 Operation With a Non-Moving Actuator Attached

The model of a non-moving actuator can be simplified as a resistor. If a resistor (with similar loading as an LRA, such as 25 O) is connected across the OUT+ and OUT– pins, and the DRV2605L device is in LRA closed-loop mode, the output pins toggle at a default frequency calculated with Equation 1. In LRA open-loop mode the output pins toggle at the specified open-loop frequency.

Copyright © 2014–2018, Texas Instruments Incorporated

8.4.4.3 Operation With a Short at REG Pin

If the REG pin is shorted to GND, the device automatically shuts down and an overcurrent-protection circuit is enabled and clamps the maximum current supplied by the regulator. When the short is removed, the device starts in the default condition.

8.4.4.4 Operation With a Short at OUT+, OUT–, or Both

If any of the output pins (OUT+ or OUT–) is shorted to V_{DD}, GND, or to each other while the device is playing a waveform, the OC_DETECT bit is asserted and remains asserted until the short is removed. A current-protection circuit automatically enables to shutdown the current through the short.

If the driver is playing a waveform the DRV2605L device checks for shorts in the output through either a haptic-playback, auto-calibration, or diagnostics process. If the short occurs when the device is idle, the short is not detected until the device attempts to run a waveform.

Copyright © 2014–2018, Texas Instruments Incorporated

8.5 Programming

8.5.1 Auto-Resonance Engine Programming for the LRA

8.5.1.1 Drive-Time Programming

The resonance frequency of each LRA actuator varies based on many factors and is generally dominated by mechanical properties. The auto-resonance engine-tracking system is optimized by providing information about the resonance frequency of the actuator. The DRIVE_TIME[4:0] bit is used as an initial guess for the half-period of the LRA. The drive time is automatically and quickly adjusted for optimum drive. For example, if the LRA has a resonance frequency of 200 Hz, then the drive time should be set to 2.5 ms.

For ERM actuators, the DRIVE_TIME[4:0] bit controls the rate for back-EMF sampling. Lower drive times imply higher back-EMF sampling frequencies which cause higher peak-to-average ratios in the output signal, and requires more supply headroom. Higher drive times imply lower back-EMF sampling frequencies which cause the feedback to react at a slower rate.

8.5.1.2 Current-Dissipation Time Programming

To sense the back-EMF of the actuator, the DRV2605L device goes into high impedance mode. However, before the device enters high impedance mode, the device must dissipate the current in the actuator. The DRV2605L device controls the time allocated for dissipation-current through the IDISS_TIME[3:0] bit.

8.5.1.3 Blanking Time Programming

After the current in the actuator dissipates, the DRV2605L device waits for a blanking time of the signal to settle before the back-EMF analog-to-digital (AD) conversion converts. The BLANKING_TIME[3:0] bit controls this time.

8.5.1.4 Zero-Crossing Detect-Time Programming

When the blanking time expires, the back-EMF AD monitors for zero crossings. The ZC_DET_TIME[1:0] bit controls the minimum time allowed for detecting zero crossings.

8.5.2 Automatic-Level Calibration Programming

8.5.2.1 Rated Voltage Programming

The rated voltage is the driving voltage that the driver will output during steady state. However, in closed-loop drive mode, temporarily having an output voltage that is higher than the rated voltage is possible. See the *Overdrive Voltage-Clamp Programming* section for details.

The RATED_VOLTAGE[7:0] bit in register 0x16 sets the rated voltage for the closed-loop drive modes. For the ERM, Equation 4 calculates the average steady-state voltage when a full-scale input signal is provided. For the LRA, Equation 5 calculates the root-mean-square (RMS) voltage when driven to steady state with a full-scale input signal.

$$V_{(ERM\text{-}CL\_AV)} = 21.18 \times 10^{-3}\ \text{RATED\_VOLTAGE[7:0]} \tag{4}$$

$$V_{(LRA\text{-}CL\_RMS)} = \frac{20.58 \times 10^{-3} \times \text{RATED\_VOLTAGE[7:0]}}{\sqrt{1 - (4 \times t_{(SAMPLE\_TIME)} + 300 \times 10^{-6}) \times f_{(LRA)}}} \tag{5}$$

In open-loop mode, the RATED_VOLTAGE[7:0] bit is ignored. Instead, the OD_CLAMP[7:0] bit (in register 0x17) is used to set the rated voltage for the open-loop drive modes. For the ERM, Equation 6 calculates the rated voltage with a full-scale input signal. For the LRA, Equation 7 calculates the RMS voltage with a full-scale input signal.

$$V_{(ERM\text{-}OL\_AV)} = 21.59 \times 10^{-3}\ \text{OD\_CLAMP[7:0]} \tag{6}$$

$$V_{(LRA\text{-}OL\_RMS)} = 21.32 \times 10^{-3} \times \text{OD\_CLAMP[7:0]} \times \sqrt{1 - f_{(LRA)} \times 800 \times 10^{-6}} \tag{7}$$

The auto-calibration routine uses the RATED_VOLTAGE[7:0] and OD_CLAMP[7:0] bits as inputs and therefore these registers must be written before calibration is performed. Any modification of this register value should be followed by calibration to appropriately set A_CAL_BEMF[7:0].

Copyright © 2014–2018, Texas Instruments Incorporated

Programming (continued)

8.5.2.2 Overdrive Voltage-Clamp Programming

During closed-loop operation, the actuator feedback allows the output voltage go above the rated voltage during the automatic overdrive and automatic braking periods. The OD_CLAMP[7:0] bit (in Register 0x17) sets a clamp so that the automatic overdrive is bounded. The OD_CLAMP[7:0] bit also serves as the full-scale reference voltage for open-loop operation. The OD_CLAMP[7:0] bit always represents the maximum *peak voltage* that is allowed, regardless of the mode.

NOTE

If the supply voltage (V_{DD}) is less than the overdrive clamp voltage, the output driver is unable to reach the clamp voltage value because the output voltage cannot exceed the supply voltage. If the rated voltage exceeds the overdrive clamp voltage, the overdrive clamp voltage has priority over the rated voltage.

In ERM mode, use Equation 8 to calculate the allowed maximum voltage. In LRA mode, use Equation 9 to calculate the maximum peak voltage.

$$V_{(ERM\_clamp)} = \frac{21.64 \times 10^{-3} \times OD\_CLAMP[7:0] \times (t_{(DRIVE\_TIME)} - 300 \times 10^{-6})}{t_{(DRIVE\_TIME)} + t_{(IDISS\_TIME)} + t_{(BLANKING\_TIME)}} \quad (8)$$

$$V_{(LRA\_clamp)} = 21.22 \times 10^{-3} \times OD\_CLAMP[7:0] \quad (9)$$

8.5.3 I²C Interface

8.5.3.1 General I²C Operation

The I²C bus employs two signals, SDA (data) and SCL (clock), to communicate between integrated circuits in a system. The bus transfers data serially, one bit at a time. The 8-bit address and data bytes are transferred with the most-significant bit (MSB) first. In addition, each byte transferred on the bus is acknowledged by the receiving device with an acknowledge bit. Each transfer operation begins with the master device driving a start condition on the bus and ends with the master device driving a stop condition on the bus. The bus uses transitions on the data pin (SDA) while the clock is at logic high to indicate start and stop conditions. A high-to-low transition on the SDA signal indicates a start, and a low-to-high transition indicates a stop. Normal data-bit transitions must occur within the low time of the clock period. Figure 20 shows a typical sequence. The master device generates the 7-bit slave address and the read-write (R/W) bit to start communication with a slave device. The master device then waits for an acknowledge condition. The slave device holds the SDA signal low during the acknowledge clock period to indicate acknowledgment. When this acknowledgment occurs, the master transmits the next byte of the sequence. Each device is addressed by a unique 7-bit slave address plus a R/W bit (1 byte). All compatible devices share the same signals through a bidirectional bus using a wired-AND connection.

The number of bytes that can be transmitted between start and stop conditions is not limited. When the last word transfers, the master generates a stop condition to release the bus. Figure 20 shows a generic data-transfer sequence.

Use external pullup resistors for the SDA and SCL signals to set the logic-high level for the bus. Pullup resistors with values between 660 Ω and 4.7 kΩ are recommended. Do not allow the SDA and SCL voltages to exceed the DRV2605L supply voltage, V_{DD}.

NOTE

The DRV2605L slave address is 0x5A (7-bit), or 1011010 in binary.

Programming (continued)

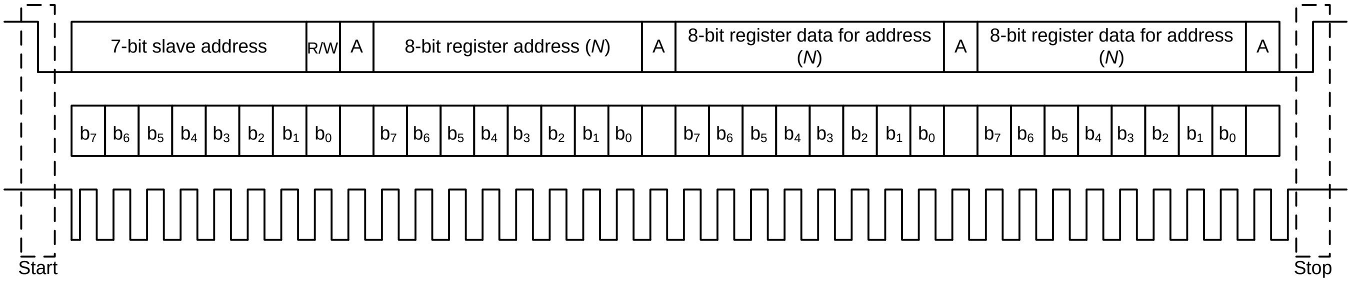

Figure 20. Typical I²C Sequence

The DRV2605L device operates as an I²C-slave 1.8-V logic thresholds, but can operate up to the V_{DD} voltage. The device address is 0x5A (7-bit), or 1011010 in binary which is equivalent to 0xB4 (8-bit) for writing and 0xB5 (8-bit) for reading.

8.5.3.2 *Single-Byte and Multiple-Byte Transfers*

The serial control interface supports both single-byte and multiple-byte R/W operations for all registers.

During multiple-byte read operations, the DRV2605L device responds with data one byte at a time and beginning at the signed register. The device responds as long as the master device continues to respond with acknowledges.

The DRV2605L supports sequential I²C addressing. For write transactions, a sequential I²C write transaction has taken place if a register is issued followed by data for that register as well as the remaining registers that follow. For I²C sequential-write transactions, the register issued then serves as the starting point and the amount of data transmitted subsequently before a stop or start is transmitted determines how many registers are written.

8.5.3.3 *Single-Byte Write*

As shown in Figure 21, a single-byte data-write transfer begins with the master device transmitting a start condition followed by the I²C device address and the read-write bit. The read-write bit determines the direction of the data transfer. For a write-data transfer, the read-write bit must be set to 0. After receiving the correct I²C device address and the read-write bit, the DRV2605L responds with an acknowledge bit. Next, the master transmits the register byte corresponding to the DRV2605L internal-memory address that is accessed. After receiving the register byte, the device responds again with an acknowledge bit. Finally, the master device transmits a stop condition to complete the single-byte data-write transfer.

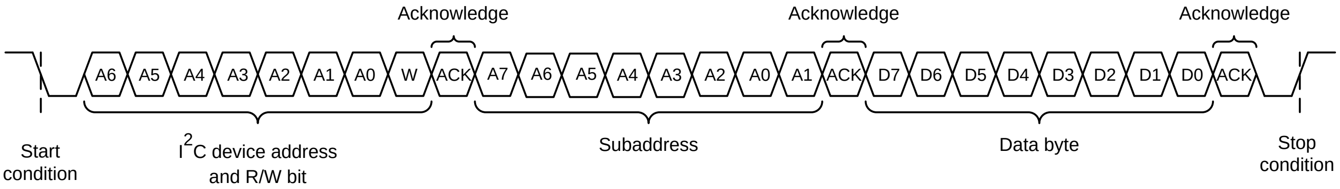

Figure 21. Single-Byte Write Transfer

Copyright © 2014–2018, Texas Instruments Incorporated

Programming (continued)

8.5.3.4 Multiple-Byte Write and Incremental Multiple-Byte Write

A multiple-byte data write transfer is identical to a single-byte data write transfer except that multiple data bytes are transmitted by the master device to the DRV2605L device as shown in Figure 22. After receiving each data byte, the DRV2605L device responds with an acknowledge bit.

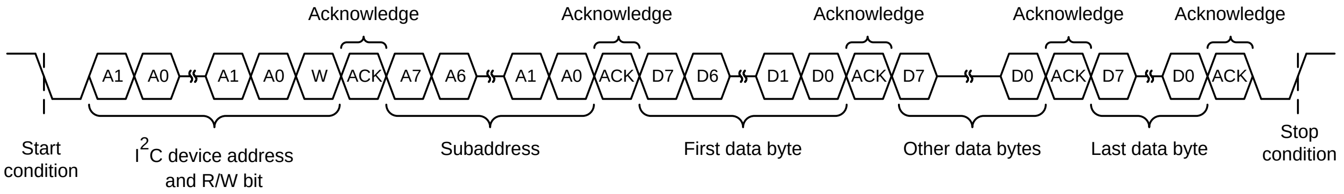

Figure 22. Multiple-Byte Write Transfer

8.5.3.5 Single-Byte Read

Figure 23 shows that a single-byte data-read transfer begins with the master device transmitting a start condition followed by the I^2C device address and the read-write bit. For the data-read transfer, both a write followed by a read actually occur. Initially, a write occurs to transfer the address byte of the internal memory address to be read. As a result, the read-write bit is set to 0.

After receiving the DRV2605L address and the read-write bit, the DRV2605L device responds with an acknowledge bit. The master then sends the internal memory address byte, after which the device issues an acknowledge bit. The master device transmits another start condition followed by the DRV2605L address and the read-write bit again. This time, the read-write bit is set to 1, indicating a read transfer. Next, the DRV2605L device transmits the data byte from the memory address that is read. After receiving the data byte, the master device transmits a not-acknowledge followed by a stop condition to complete the single-byte data read transfer. See the note in the *General I^2C Operation* section.

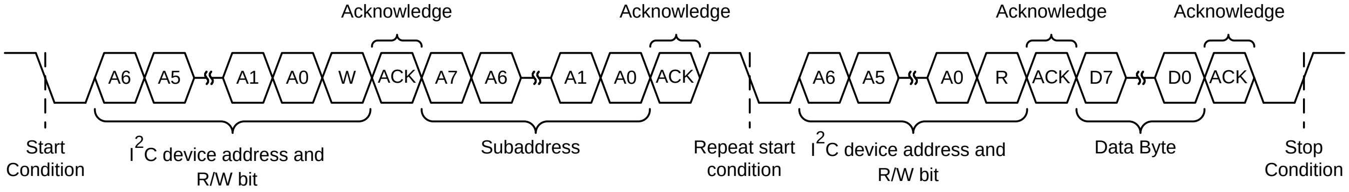

Figure 23. Single-Byte Read Transfer

8.5.3.6 Multiple-Byte Read

A multiple-byte data-read transfer is identical to a single-byte data-read transfer except that multiple data bytes are transmitted by the DRV2605L device to the master device as shown in Figure 24. With the exception of the last data byte, the master device responds with an acknowledge bit after receiving each data byte.

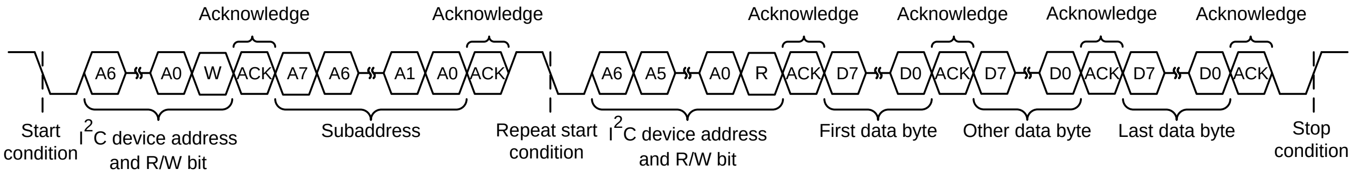

Figure 24. Multiple-Byte Read Transfer

Programming (continued)

8.5.4 Programming for Open-Loop Operation

The DRV2605L device can be used in open-loop mode and closed-loop mode. If open-loop operation is desired, the first step is to determine which actuator type is to use, either ERM or LRA.

8.5.4.1 Programming for ERM Open-Loop Operation

To configure the DRV2605L device in ERM open-loop operation, the ERM must be selected by writing the N_ERM_LRA bit to 0 (in register 0x1A), and the ERM_OPEN_LOOP bit to 1 in register 0x1D.

8.5.4.2 Programming for LRA Open-Loop Operation

To configure the DRV2605L device in LRA open-loop operation, the LRA must be selected by writing the N_ERM_LRA bit to 1 in register 0x1A, and the LRA_OPEN_LOOP bit to 1 in register 0x1D. If PWM interface is used, the open-loop frequency is given by the PWM frequency divided by 128. If PWM interface is not used, the open-loop frequency is given by the OL_LRA_PERIOD[6:0] bit in register 0x20.

8.5.5 Programming for Closed-Loop Operation

For closed-loop operation, the device must be calibrated according to the actuator selection. When calibrated accordingly, the user is only required to provide the desired waveform. The DRV2605L device automatically adjusts the level and, for the LRA, automatically adjusts the driving frequency.

8.5.6 Auto Calibration Procedure

The calibration engine requires a number of bits as inputs before the engine can be executed (see Figure 25). When the inputs are configured, the calibration routine can be executed. After calibration execution occurs, the output parameters are written over the specified register locations. Figure 25 shows all of the required inputs and generated outputs. To ensure proper auto-resonance operation, the LRA actuator type requires more input parameters than the ERM. The LRA parameters are ignored when the device is in ERM mode.

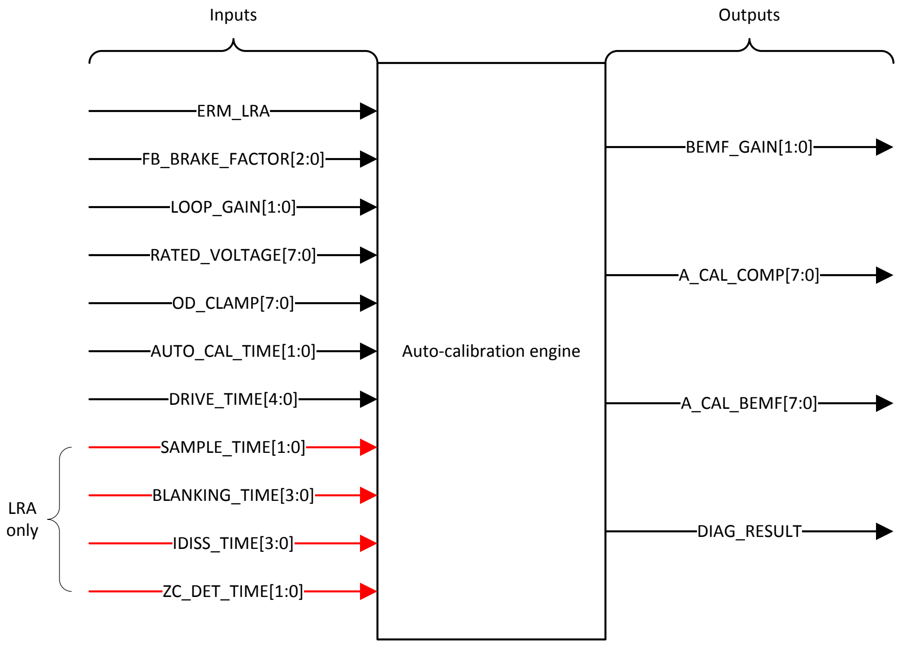

Figure 25. Calibration-Engine Functional Diagram

Variation occurs between different actuators even if the actuators are of the same model. To ensure optimal results, TI recommends that the calibration routine be run at least once for each actuator. The OTP feature of the DRV2605L device can store the calibration values. Because of the stored values, the calibration procedure does not have run every time. Having a single set of calibration register values that can be loaded during the system initialization is possible.

Copyright © 2014–2018, Texas Instruments Incorporated

Programming (continued)

The following instructions list the step-by-step register configuration for auto-calibration. For additional details see the *Register Map* section.

1. Apply the supply voltage to the DRV2605L device, and pull the EN pin high. The supply voltage should allow for adequate drive voltage of the selected actuator.
2. Write a value of 0x07 to register 0x01. This value moves the DRV2605L device out of STANDBY and places the MODE[2:0] bits in auto-calibration mode.
3. Populate the input parameters required by the auto-calibration engine:
   a. ERM_LRA — selection will depend on desired actuator.
   b. FB_BRAKE_FACTOR[2:0] — A value of 2 is valid for most actuators.
   c. LOOP_GAIN[1:0] — A value of 2 is valid for most actuators.
   d. RATED_VOLTAGE[7:0] — See the *Rated Voltage Programming* section for calculating the correct register value.
   e. OD_CLAMP[7:0] — See the *Overdrive Voltage-Clamp Programming* section for calculating the correct register value.
   f. AUTO_CAL_TIME[1:0] — A value of 3 is valid for most actuators.
   g. DRIVE_TIME[3:0] — See the *Drive-Time Programming* for calculating the correct register value.
   h. SAMPLE_TIME[1:0] — A value of 3 is valid for most actuators.
   i. BLANKING_TIME[3:0] — A value of 1 is valid for most actuators.
   j. IDISS_TIME[3:0] — A value of 1 is valid for most actuators.
   k. ZC_DET_TIME[1:0] — A value of 0 is valid for most actuators.
4. Set the GO bit (write 0x01 to register 0x0C) to start the auto-calibration process. When auto calibration is complete, the GO bit automatically clears. The auto-calibration results are written in the respective registers as shown in Figure 25.
5. Check the status of the DIAG_RESULT bit (in register 0x00) to ensure that the auto-calibration routine is complete without faults.
6. Evaluate system performance with the auto-calibrated settings. Note that the evaluation should occur during the final assembly of the device because the auto-calibration process can affect actuator performance and behavior. If any adjustment is required, the inputs can be modified and this sequence can be repeated. If the performance is satisfactory, the user can do any of the following:
   a. Repeat the calibration process upon subsequent power ups.
   b. Store the auto-calibration results in host processor memory and rewrite them to the DRV2605L device upon subsequent power ups. The device retains these settings when in STANDBY mode or when the EN pin is low.
   c. Program the results permanently in nonvolatile, on-chip OTP memory. Even when a device power cycle occurs, the device retains the auto-calibration settings. See the *Programming On-Chip OTP Memory* section for additional information.

8.5.7 Programming On-Chip OTP Memory

The OTP memory can only be written once. To permanently program the OTP memory in registers 0x16 through 0x1A, use the following steps:

1. Write registers 0x16 through 0x1A with the desired configuration and calibration values which provide satisfactory performance.
2. Ensure that the supply voltage (V_{DD}) is between 4 V and 4.4 V. This voltage is required for the nonvolatile memory to program properly.
3. Set the OTP_PROGRAM bit by writing a value of 0x01 to register 0x1E. When the OTP memory is written which can only occur once in the device, the OTP_STATUS bit (in register 0x1E) only reads 1.
4. Reset the device by power cycling the device or setting the DEV_RESET bit in register 0x01, and then read registers 0x16 to 0x1A to ensure that the programmed values were retained.

Programming (continued)

8.5.8 Waveform Playback Programming

8.5.8.1 Data Formats for Waveform Playback

The DRV2605L smart-loop architecture has three modes of operation. Each of the modes can drive either ERM or LRA devices.

1. Open-loop mode
2. Closed-loop mode (unidirectional)
3. Closed-loop mode (bidirectional)

Each mode has different advantages and disadvantages. The DRV2605L device brings new cutting-edge actuator control with closed-loop operation around the back-EMF for automatic overdrive and braking. However, some existing haptic implementations already include overdrive and braking that are embedded in the waveform data. Open-loop mode is used to preserve compatibility with such systems.

The following sections show how the input data for each DRV2605L interface is translated to the output drive signal.

8.5.8.1.1 Open-Loop Mode

In open-loop mode, the reference level for full-scale drive is set by the OD_CLAMP[7:0] bit in Register 0x17. A mid-scale input value gives no drive signal, and a less-than mid-scale gives a negative drive value. For an ERM, a negative drive value results in counter-rotation, or braking. For an LRA, a negative drive value results in a 180-degree phase shift in commutation.

The RTP mode has 8 bits of resolution over the I^2C bus. The RTP data can either be in a signed (2s complement) or unsigned format as defined by the DATA_FORMAT_RTP bit.

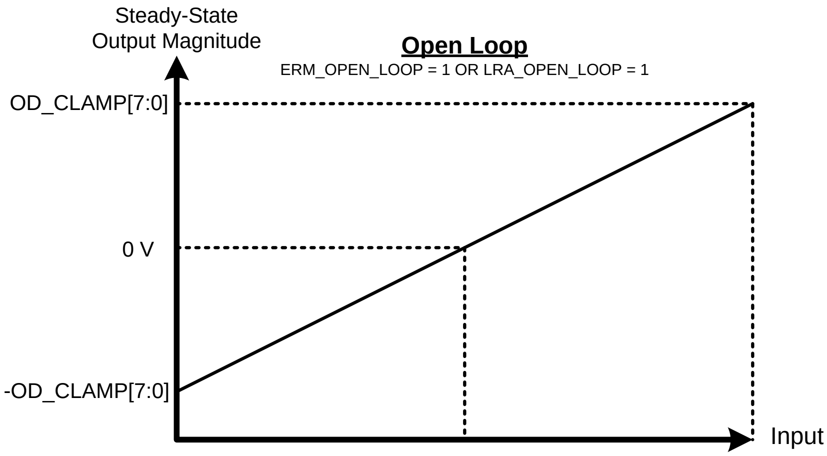

Input Interface			
PWM	0%	50%	100%
RTP (8-bit) DATA_FORMAT_RTP = 0	0x81	0x00	0x7F
RTP (8-bit) DATA_FORMAT_RTP = 1	0x00	0x7F	0xFF

Figure 26.

Copyright © 2014–2018, Texas Instruments Incorporated

Programming (continued)

8.5.8.1.2 Closed-Loop Mode, Unidirectional

In closed-loop unidirectional mode, the DRV2605L device provides automatic overdrive and braking for both ERM and LRA actuators. Closed-loop unidirectional mode is the easiest mode to use and understand. Closed-loop unidirectional mode uses the full 8-bit resolution of the driver. Closed-loop unidirectional mode offers the best performance; however, the data format is not physically compatible with the open-loop mode data that can be used in some existing systems

The reference level for steady-state full-scale drive is set by the RATED_VOLTAGE[7:0] bit (when auto-calibration is performed). The output voltage can momentarily exceed the rated voltage for automatic overdrive and braking, but does not exceed the OD_CLAMP[7:0] voltage. Braking occurs automatically based on the input signal when the back-EMF feedback determines that braking is necessary.

Because the system is unidirectional in closed-loop unidirectional mode, only unsigned data should be used. The RTP mode has 8 bits of resolution over the I^{2}C bus. Setting the DATA_FORMAT_RTP bit to 0 (signed) is not recommended for closed-loop unidirectional mode.

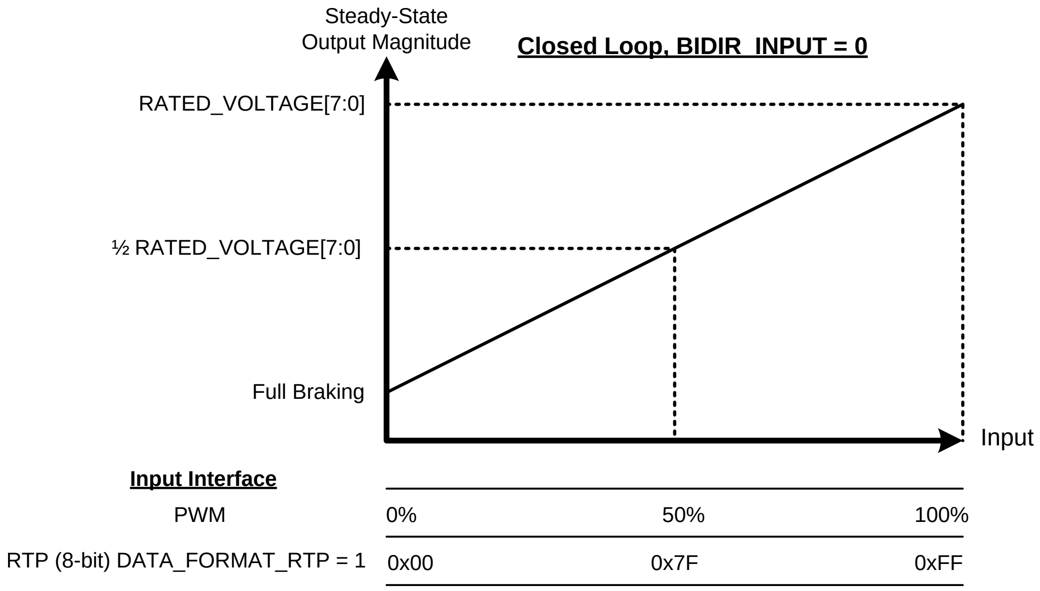

Figure 27.

NOTE

The TS2200 library data is stored in bidirectional format and cannot be used in unidirectional mode.

For the RTP interface, set the DATA_FORMAT_RTP bit to 1 (unsigned).

Programming (continued)

8.5.8.1.3 Closed-Loop Mode, Bidirectional

In closed-loop bidirectional mode, the DRV2605L device provides automatic overdrive and braking for both ERM and LRA devices. Closed-loop bidirectional mode preserves compatibility with data created in open-loop signaling by maintaining zero drive-strength at the mid-scale value. When input values less than the mid-scale value are given, the DRV2605L device interprets them as the same as the mid-scale with zero drive.

The reference level for steady-state full-scale drive is set by the RATED_VOLTAGE[7:0] bit (when auto calibration is performed). The output voltage can momentarily exceed the rated voltage for automatic overdrive and braking, but does not exceed the OD_CLAMP[7:0] voltage. Braking occurs automatically based on the input signal when the back-EMF feedback determines that braking is necessary. Although the Closed-Loop mode preserves compatibility with existing device data formats, it provides closed loop benefits and is the default configuration at power up.

The RTP mode has 8 bits of resolution over the I^2C bus. The RTP data can either be in signed (2s complement) or unsigned format as defined by the DATA_FORMAT_RTP bit.

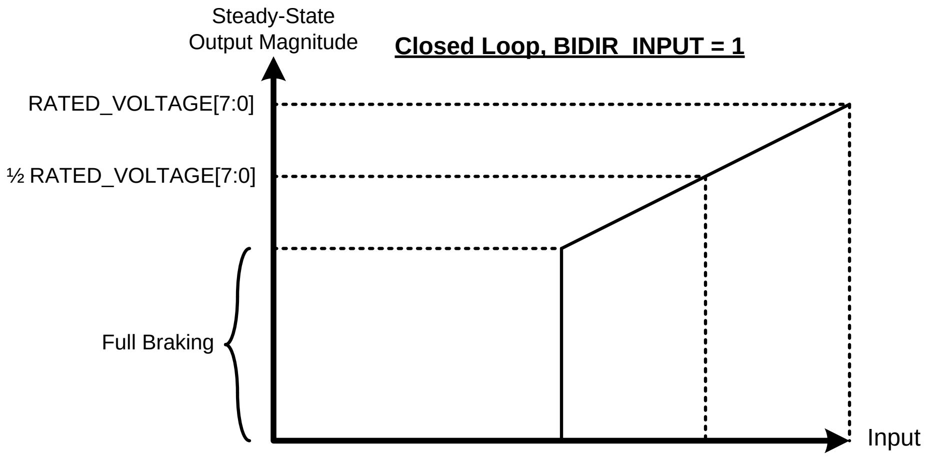

Input Interface				
PWM	0%	50%	75%	100%
RTP (8-bit) DATA_FORMAT_RTP = 0	0x81	0x00	0x3F	0x7F
RTP (8-bit) DATA_FORMAT_RTP = 1	0x00	0x7F	0xBF	0xFF

Figure 28.

NOTE

Closed-loop bidirectional mode is compatible with all DRV2605L interfaces except for TS2200 Library A (with fixed overdrive programming). Library A should only be used in open-loop mode. Libraries B through F (no overdrive) can take advantage of the automatic overdrive and braking of closed-loop bidirectional mode.

Copyright © 2014–2018, Texas Instruments Incorporated

Programming (continued)

8.5.8.2 Waveform Setup and Playback

Playback of a haptic effect can occur in multiple ways. Using the PWM mode, RTP mode, audio-to-vibe mode, and analog-input mode can provide the waveform in real time. The waveforms can also be played from the ROM in which case the waveform playback engine is used and the waveform is either played by an internal GO bit (register 0x0C), or by an external trigger.

8.5.8.2.1 Waveform Playback Using RTP Mode

The user can enter the RTP mode by writing the MODE[2:0] bit to 5 in register 0x01. When in RTP mode, the DRV2605L device drives the actuator continuously with the amplitude specified in the RTP_INPUT[7:0] bit (in register 0x02). Because the amplitude tracks the value specified in the RTP_INPUT[7:0] bit, the I^2C bus can stream waveforms.

8.5.8.2.2 Waveform Playback Using the Analog-Input Mode

The user can enter the analog-input mode by setting the MODE[2:0] bit to 3 in register 0x01 and by setting the N_PWM_ANALOG bit to 1 in register 0x1D. When in analog-input mode, the DRV2605L device accepts an analog voltage at the IN/TRIG pin. The DRV2605L device drives the actuator continuously in analog-input mode until the user sets the device into STANDBY mode or enters another interface mode. The reference voltage in analog-input mode is 1.8 V. Therefore a 1.8-V reference voltage is interpreted as a 100% input value, a 0.9-V reference voltage is interpreted as 50%, and a 0-V reference voltage is interpreted as 0%. The input value is analogous to the duty-cycle percentage in PWM mode. The interpretation of these percentages varies according to the selected mode of operation. See the *Data Formats for Waveform Playback* section for details.

8.5.8.2.3 Waveform Playback Using PWM Mode

The user can enter the PWM mode by setting the MODE[2:0] bit to 3 in register 0x01 and by setting the N_PWM_ANALOG bit to 0 in register 0x1D. When in PWM mode, the DRV2605L device accepts PWM data at the IN/TRIG pin. The DRV2605L device drives the actuator continuously in PWM mode until the user sets the device to STANDBY mode or to enter another interface mode. The interpretation of the duty-cycle information varies according to the selected mode of operation. See the *Data Formats for Waveform Playback* section for details.

8.5.8.2.4 Waveform Playback Using Audio-to-Vibe Mode

To take advantage of the audio-to-vibe feature, connect the DRV2605L device to a line-out source as shown in Figure 59. The full-scale range of the IN/TRIG pin in the audio-to-vibe mode is 1.8 V_{PP}. A 1-µF capacitor is recommended to AC couple the audio source and the IN/TRIG pin. For sources smaller than 1.8 V_{PP}, the ATH_MAX_INPUT bit in register 0x13 can scale down the input range.

The device enters audio-to-vibe mode when the MODE[2:0] bit is set to 4 in register 0x01 and when the AC_COUPLE bit in register 0x1B and the N_PWM_ANALOG bit in register 0x1D are set to 1. See the *Register Map* section for details.

8.5.8.2.5 Waveform Sequencer

If the user uses library effects, the effects must first be loaded into the waveform sequencer, and then the effects can be launched by using any of the trigger options (see the *Waveform Triggers* section for details).

The waveform sequencer (see the *Waveform Sequencer (Address: 0x04 to 0x0B)* section) queues waveform-library identifiers for playback. Eight sequence registers queue up to eight library waveforms for sequential playback. A waveform identifier is an integer value referring to the index position of a waveform in the ROM library. Playback begins at register address 0x04 when the user asserts the GO bit (register 0x0C). When playback of that waveform ends, the waveform sequencer plays the next waveform identifier held in register 0x05, if the next waveform is non-zero. The waveform sequencer continues in this way until the sequencer reaches an identifier value of zero or until all eight identifiers are played (register addresses 0x04 through 0x0B), whichever comes first.

The waveform identifier range is 1 to 123. The MSB of each sequence register can be used to implement a delay between sequence waveforms. When the MSB is high, bits 6-0 indicate the length of the wait time. The wait time for that step then becomes WAV_FRM_SEQ[6:0] × 10 ms.

Programming (continued)

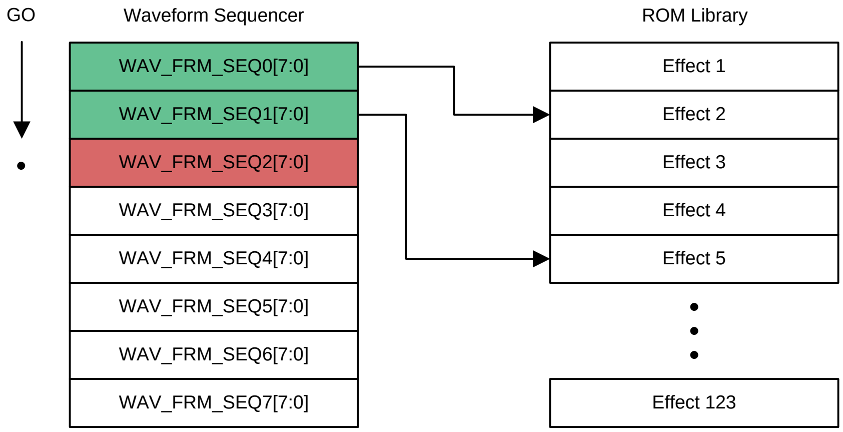

Figure 29. Waveform Sequencer Programming

8.5.8.2.6 Waveform Triggers

When the waveform sequencer has the effect (or effects) loaded, the waveform sequencer can be triggered by an internal trigger, external trigger (edge), or external trigger (level). To trigger using the internal trigger set the MODE[2:0] bit to 0 in register 0x01. To trigger using the external trigger (edge), set the MODE[2:0] bit to 1 and then follow the trigger instructions listed in the *Edge Trigger* section. To trigger using the external trigger (level), set the MODE[2:0] bit to 2 and then follow the trigger instructions listed in the *Level Trigger* section.

Copyright © 2014–2018, Texas Instruments Incorporated

8.6 Register Map

Table 3. Register Map Overview

<table>
<tr><th>REG NO.</th><th>DEFAULT</th><th>BIT 7</th><th>BIT 6</th><th>BIT 5</th><th>BIT 4</th><th>BIT 3</th><th>BIT 2</th><th>BIT 1</th><th>BIT 0</th></tr>
<tr><td>0x00</td><td>0xE0</td><td colspan="3">DEVICE_ID[2:0]</td><td>Reserved</td><td>DIAG_RESULT</td><td>Reserved</td><td>OVER_TEMP</td><td>OC_DETECT</td></tr>
<tr><td>0x01</td><td>0x40</td><td>DEV_RESET</td><td>STANDBY</td><td colspan="3">Reserved</td><td colspan="3">MODE[2:0]</td></tr>
<tr><td>0x02</td><td>0x00</td><td colspan="8">RTP_INPUT[7:0]</td></tr>
<tr><td>0x03</td><td>0x01</td><td colspan="3">Reserved</td><td>HI_Z</td><td>Reserved</td><td>LIBRARY_SEL[2]</td><td>LIBRARY_SEL[1]</td><td>LIBRARY_SEL[0]</td></tr>
<tr><td>0x04</td><td>0x01</td><td>WAIT1</td><td colspan="7">WAV_FRM_SEQ1[6:0]</td></tr>
<tr><td>0x05</td><td>0x00</td><td>WAIT2</td><td colspan="7">WAV_FRM_SEQ2[6:0]</td></tr>
<tr><td>0x06</td><td>0x00</td><td>WAIT3</td><td colspan="7">WAV_FRM_SEQ3[6:0]</td></tr>
<tr><td>0x07</td><td>0x00</td><td>WAIT4</td><td colspan="7">WAV_FRM_SEQ4[6:0]</td></tr>
<tr><td>0x08</td><td>0x00</td><td>WAIT5</td><td colspan="7">WAV_FRM_SEQ5[6:0]</td></tr>
<tr><td>0x09</td><td>0x00</td><td>WAIT6</td><td colspan="7">WAV_FRM_SEQ6[6:0]</td></tr>
<tr><td>0x0A</td><td>0x00</td><td>WAIT7</td><td colspan="7">WAV_FRM_SEQ7[6:0]</td></tr>
<tr><td>0x0B</td><td>0x00</td><td>WAIT8</td><td colspan="7">WAV_FRM_SEQ8[6:0]</td></tr>
<tr><td>0x0C</td><td>0x00</td><td colspan="7">Reserved</td><td>GO</td></tr>
<tr><td>0x0D</td><td>0x00</td><td colspan="8">ODT[7:0]</td></tr>
<tr><td>0x0E</td><td>0x00</td><td colspan="8">SPT[7:0]</td></tr>
<tr><td>0x0F</td><td>0x00</td><td colspan="8">SNT[7:0]</td></tr>
<tr><td>0x10</td><td>0x00</td><td colspan="8">BRT[7:0]</td></tr>
<tr><td>0x11</td><td>0x05</td><td colspan="4">Reserved</td><td colspan="2">ATH_PEAK_TIME[1:0]</td><td colspan="2">ATH_FILTER[1:0]</td></tr>
<tr><td>0x12</td><td>0x19</td><td colspan="8">ATH_MIN_INPUT[7:0]</td></tr>
<tr><td>0x13</td><td>0xFF</td><td colspan="8">ATH_MAX_INPUT[7:0]</td></tr>
<tr><td>0x14</td><td>0x19</td><td colspan="8">ATH_MIN_DRIVE[7:0]</td></tr>
<tr><td>0x15</td><td>0xFF</td><td colspan="8">ATH_MAX_DRIVE[7:0]</td></tr>
<tr><td>0x16</td><td>0x3E</td><td colspan="8">RATED_VOLTAGE[7:0]</td></tr>
<tr><td>0x17</td><td>0x8C</td><td colspan="8">OD_CLAMP[7:0]</td></tr>
<tr><td>0x18</td><td>0x0C</td><td colspan="8">A_CAL_COMP[7:0]</td></tr>
<tr><td>0x19</td><td>0x6C</td><td colspan="8">A_CAL_BEMF[7:0]</td></tr>
<tr><td>0x1A</td><td>0x36</td><td>N_ERM_LRA</td><td colspan="3">FB_BRAKE_FACTOR[2:0]</td><td colspan="2">LOOP_GAIN[1:0]</td><td colspan="2">BEMF_GAIN[1:0]</td></tr>
<tr><td>0x1B</td><td>0x93</td><td>STARTUP_BOOST</td><td>Reserved</td><td>AC_COUPLE</td><td colspan="5">DRIVE_TIME[4:0]</td></tr>
<tr><td>0x1C</td><td>0xF5</td><td>BIDIR_INPUT</td><td>BRAKE_STABILIZER</td><td colspan="2">SAMPLE_TIME[1:0]</td><td colspan="2">BLANKING_TIME[1:0]</td><td colspan="2">IDISS_TIME[1:0]</td></tr>
<tr><td>0x1D</td><td>0xA0</td><td colspan="2">NG_THRESH[1:0]</td><td>ERM_OPEN_LOOP</td><td>SUPPLY_COMP_DIS</td><td>DATA_FORMAT_RTP</td><td>LRA_DRIVE_MODE</td><td>N_PWM_ANALOG</td><td>LRA_OPEN_LOOP</td></tr>
<tr><td>0x1E</td><td>0x20</td><td colspan="2">ZC_DET_TIME[1:0]</td><td colspan="2">AUTO_CAL_TIME[1:0]</td><td>Reserved</td><td>OTP_STATUS</td><td>Reserved</td><td>OTP_PROGRAM</td></tr>
<tr><td>0x1F</td><td>0x80</td><td colspan="2">AUTO_OL_CNT[1:0]</td><td>LRA_AUTO_OPEN_LOOP</td><td>PLAYBACK_INTERVAL</td><td colspan="2">BLANKING_TIME[3:2]</td><td colspan="2">IDISS_TIME[3:2]</td></tr>
<tr><td>0x20</td><td>0x33</td><td>Reserved</td><td colspan="7">OL_LRA_PERIOD[6:0]</td></tr>
<tr><td>0x21</td><td>0x00</td><td colspan="8">VBAT[7:0]</td></tr>
<tr><td>0x22</td><td>0x00</td><td colspan="8">LRA_PERIOD[7:0]</td></tr>
</table>

Copyright © 2014–2018, Texas Instruments Incorporated

8.6.1 Status (Address: 0x00)

Figure 30. Status Register

7	6	5	4	3	2	1	0
DEVICE_ID[2:0]			Reserved	DIAG_RESULT	Reserved	OVER_TEMP	OC_DETECT
RO-1	RO-1	RO-1	RO-0			RO-0	RO-0

Table 4. Status Register Field Descriptions

BIT	FIELD	TYPE	DEFAULT	DESCRIPTION
7-5	DEVICE_ID[2:0]	RO	7	Device identifier. The DEVICE_ID bit indicates the part number to the user. The user software can ascertain the device capabilities by reading this register.<br>3: DRV2605 (contains licensed ROM library, does not contain RAM)<br>4: DRV2604 (contains RAM, does not contain licensed ROM library)<br>6: DRV2604L (low-voltage version of the DRV2604 device)<br>7: DRV2605L (low-voltage version of the DRV2605 device)
4	Reserved			
3	DIAG_RESULT	RO	0	This flag stores the result of the auto-calibration routine and the diagnostic routine. The flag contains the result for whichever routine was executed last. The flag clears upon read. Test result is not valid until the GO bit self-clears at the end of the routine.<br>Auto-calibration mode:<br>0: Auto-calibration passed (optimum result converged)<br>1: Auto-calibration failed (result did not converge)<br>Diagnostic mode:<br>0: Actuator is functioning normally<br>1: Actuator is not present or is shorted, timing out, or giving out–of-range back-EMF
2	Reserved			
1	OVER_TEMP	RO	0	Latching overtemperature detection flag. If the device becomes too hot, it shuts down. This bit clears upon read.<br>0: Device is functioning normally<br>1: Device has exceeded the temperature threshold
0	OC_DETECT	RO	0	Latching overcurrent detection flag. If the load impedance is below the load-impedance threshold, the device shuts down and periodically attempts to restart until the impedance is above the threshold.<br>0: No overcurrent event is detected<br>1: Overcurrent event is detected

Copyright © 2014–2018, Texas Instruments Incorporated

8.6.2 Mode (Address: 0x01)

Figure 31. Mode Register

7	6	5	4	3	2	1	0
DEV_RESET	STANDBY	Reserved			MODE[2:0]		
R/W-0	R/W-1				R/W-0		

Table 5. Mode Register Field Descriptions

BIT	FIELD	TYPE	DEFAULT	DESCRIPTION
7	DEV_RESET	R/W	0	Device reset. Setting this bit performs the equivalent operation of power cycling the device. Any playback operations are immediately interrupted, and all registers are reset to the default values. The DEV_RESET bit self-clears after the reset operation is complete.
6	STANDBY	R/W	1	Software standby mode<br>0: Device ready<br>1: Device in software standby
5-3	Reserved			
2-0	MODE	R/W	0	0: Internal trigger<br>Waveforms are fired by setting the GO bit in register 0x0C.<br>1: External trigger (edge mode)<br>A rising edge on the IN/TRIG pin sets the GO Bit. A second rising edge on the IN/TRIG pin cancels the waveform if the second rising edge occurs before the GO bit has cleared.<br>2: External trigger (level mode)<br>The GO bit follows the state of the external trigger. A rising edge on the IN/TRIG pin sets the GO bit, and a falling edge sends a cancel. If the GO bit is already in the appropriate state, no change occurs.<br>3: PWM input and analog input<br>A PWM or analog signal is accepted at the IN/TRIG pin and used as the driving source. The device actively drives the actuator while in this mode. The PWM or analog input selection occurs by using the N_PWM_ANALOG bit.<br>4: Audio-to-vibe<br>An AC-coupled audio signal is accepted at the IN/TRIG pin. The device converts the audio signal into meaningful haptic vibration. The AC_COUPLE and N_PWM_ANALOG bits should also be set.<br>5: Real-time playback (RTP mode)<br>The device actively drives the actuator with the contents of the RTP_INPUT[7:0] bit in register 0x02.<br>6: Diagnostics<br>Set the device in this mode to perform a diagnostic test on the actuator. The user must set the GO bit to start the test. The test is complete when the GO bit self-clears. Results are stored in the DIAG_RESULT bit in register 0x00.<br>7: Auto calibration<br>Set the device in this mode to auto calibrate the device for the actuator. Before starting the calibration, the user must set the all required input parameters. The user must set the GO bit to start the calibration. Calibration is complete when the GO bit self-clears. For more information see the *Auto Calibration Procedure* section.

8.6.3 Real-Time Playback Input (Address: 0x02)

Figure 32. Real-Time Playback Input Register

7	6	5	4	3	2	1	0
RTP_INPUT[7:0]							
R/W-0							

Table 6. Real-Time Playback Input Register Field Descriptions

BIT	FIELD	TYPE	DEFAULT	DESCRIPTION
7-0	RTP_INPUT[7:0]	R/W	0	This field is the entry point for real-time playback (RTP) data. The DRV2605L playback engine drives the RTP_INPUT[7:0] value to the load when MODE[2:0] = 5 (RTP mode). The RTP_INPUT[7:0] value can be updated in real-time by the host controller to create haptic waveforms. The RTP_INPUT[7:0] value is interpreted as signed by default, but can be set to unsigned by the DATA_FORMAT_RTP bit in register 0x1D. When the haptic waveform is complete, the user can idle the device by setting MODE[2:0] = 0, or alternatively by setting STANDBY = 1.

8.6.4 Library Selection (Address: 0x03)

Figure 33. Library Selection Register

7	6	5	4	3	2	1	0
Reserved			HI_Z	Reserved	LIBRARY_SEL[2:0]		
R/W-0					R/W-0	R/W-0	R/W-1

Table 7. Library Selection Register Field Descriptions

BIT	FIELD	TYPE	DEFAULT	DESCRIPTION
7-5	Reserved			
4	HI_Z	R/W	0	This bit sets the output driver into a true high-impedance state. The device must be enabled to go into the high-impedance state. When in hardware shutdown or standby mode, the output drivers have 15 kO to ground. When the HI_Z bit is asserted, the hi-Z functionality takes effect immediately, even if a transaction is taking place.
3	Reserved			
2-0	LIBRARY_SEL	R/W	1	Waveform library selection value. This bit determines which library the playback engine selects when the GO bit is set. For additional details on the ERM libraries see the *Table 1* section.<br>0: Empty<br>1: TS2200 Library A<br>2: TS2200 Library B<br>3: TS2200 Library C<br>4: TS2200 Library D<br>5: TS2200 Library E<br>6: LRA Library<br>7: TS2200 Library F

Copyright © 2014–2018, Texas Instruments Incorporated

8.6.5 Waveform Sequencer (Address: 0x04 to 0x0B)

Figure 34. Waveform Sequencer Register

7	6	5	4	3	2	1	0
WAIT	WAV_FRM_SEQ[6:0]						
R/W-0	R/W-0						

Table 8. Waveform Sequencer Register Field Descriptions

BIT	FIELD	TYPE	DEFAULT	DESCRIPTION
7	WAIT	R/W	0	When this bit is set, the WAV_FRM_SEQ[6:0] bit is interpreted as a *wait time* in which the playback engine idles. This bit is used to insert timed delays between sequentially played waveforms.<br>Delay time = 10 ms × WAV_FRM_SEQ[6:0]<br>If WAIT = 0, then WAV_FRM_SEQ[6:0] is interpreted as a waveform identifier for sequence playback.
6-0	WAV_FRM_SEQ	R/W	0	Waveform sequence value. This bit holds the waveform identifier of the waveform to be played. A waveform identifier is an integer value referring to the index position of a waveform in a ROM library. Playback begins at register address 0x04 when the user asserts the GO bit (register 0x0C). When playback of that waveform ends, the waveform sequencer plays the next waveform identifier held in register 0x05, if the next waveform identifier is non-zero. The waveform sequencer continues in this way until the sequencer reaches an identifier value of zero, or all eight identifiers are played (register addresses 0x04 through 0x0B), whichever comes first.

8.6.6 GO (Address: 0x0C)

Figure 35. GO Register

7	6	5	4	3	2	1	0
Reserved							GO
							R/W-0

Table 9. GO Register Field Descriptions

BIT	FIELD	TYPE	DEFAULT	DESCRIPTION
7-1	Reserved			
0	GO	R/W	0	This bit is used to fire processes in the DRV2605L device. The process fired by the GO bit is selected by the MODE[2:0] bit (register 0x01). The primary function of this bit is to fire playback of the waveform identifiers in the waveform sequencer (registers 0x04 to 0x0B), in which case, this bit can be thought of a *software trigger* for haptic waveforms. The GO bit remains high until the playback of the haptic waveform sequence is complete. Clearing the GO bit during waveform playback cancels the waveform sequence. Using one of the external trigger modes can cause the GO bit to be set or cleared by the external trigger pin. This bit can also be used to fire the auto-calibration process or the diagnostic process.

8.6.7 Overdrive Time Offset (Address: 0x0D)

Figure 36. Overdrive Time Offset Register

7	6	5	4	3	2	1	0
ODT[7:0]							
R/W-0							

Table 10. Overdrive Time Offset Register Field Descriptions

BIT	FIELD	TYPE	DEFAULT	DESCRIPTION
7-0	ODT	R/W	0	This bit adds a time offset to the overdrive portion of the library waveforms. Some motors require more overdrive time than others, therefore this register allows the user to add or remove overdrive time from the library waveforms. The maximum voltage value in the library waveform is automatically determined to be the overdrive portion. This register is only useful in open-loop mode. Overdrive is automatic for closed-loop mode. The offset is interpreted as 2s complement, therefore the time offset can be positive or negative.<br>Overdrive Time Offset (ms) = ODT[7:0] × PLAYBACK_INTERVAL

8.6.8 Sustain Time Offset, Positive (Address: 0x0E)

Figure 37. Sustain Time Offset, Positive Register

7	6	5	4	3	2	1	0
SPT[7:0]							
R/W-0							

Table 11. Sustain Time Offset, Positive Register Field Descriptions

BIT	FIELD	TYPE	DEFAULT	DESCRIPTION
7-0	SPT	R/W	0	This bit adds a time offset to the positive sustain portion of the library waveforms. Some motors have a faster or slower response time than others, therefore this register allows the user to add or remove positive sustain time from the library waveforms. Any positive voltage value other than the overdrive portion is considered as a sustain positive value. The offset is interpreted as 2s complement, therefore the time offset can positive or negative.<br>Sustain-Time Positive Offset (ms) = SPT[7:0] × PLAYBACK_INTERVAL

Copyright © 2014–2018, Texas Instruments Incorporated

8.6.9 Sustain Time Offset, Negative (Address: 0x0F)

Figure 38. Sustain Time Offset, Negative Register

7	6	5	4	3	2	1	0
SNT[7:0]							
R/W-0							

Table 12. Sustain Time Offset, Negative Register Field Descriptions

BIT	FIELD	TYPE	DEFAULT	DESCRIPTION
7-0	SNT	R/W	0	This bit adds a time offset to the negative sustain portion of the library waveforms. Some motors have a faster or slower response time than others, therefore this register allows the user to add or remove negative sustain time from the library waveforms. Any negative voltage value other than the overdrive portion is considered as a sustaining negative value. The offset is interpreted as two's complement, therefore the time offset can be positive or negative.<br>Sustain-Time Negative Offset (ms) = SNT[7:0] × PLAYBACK_INTERVAL

8.6.10 Brake Time Offset (Address: 0x10)

Figure 39. Brake Time Offset Register

7	6	5	4	3	2	1	0
BRT[7:0]							
R/W-0							

Table 13. Brake Time Offset Register Field Descriptions

BIT	FIELD	TYPE	DEFAULT	DESCRIPTION
7-0	BRT	R/W	0	This bit adds a time offset to the braking portion of the library waveforms. Some motors require more braking time than others, therefore this register allows the user to add or take away brake time from the library waveforms. The most negative voltage value in the library waveform is automatically determined to be the braking portion. This register is only useful in open-loop mode. Braking is automatic for closed-loop mode. The offset is interpreted as 2s complement, therefore the time offset can be positive or negative.<br>Brake Time Offset (ms) = BRT[7:0] × PLAYBACK_INTERVAL

8.6.11 Audio-to-Vibe Control (Address: 0x11)

Figure 40. Audio-to-Vibe Control Register

7	6	5	4	3	2	1	0
Reserved				ATH_PEAK_TIME[1:0]		ATH_FILTER[1:0]	
				R/W-0	R/W-1	R/W-0	R/W-1

Table 14. Audio-to-Vibe Control Register Field Descriptions

BIT	FIELD	TYPE	DEFAULT	DESCRIPTION
7-4	Reserved			
3-2	ATH_PEAK_TIME[1:0]	R/W	1	This bit sets the peak detection time for the audio-to-vibe signal path:<br>0: 10 ms<br>1: 20 ms<br>2: 30 ms<br>3: 40 ms
1-0	ATH_FILTER[1:0]	R/W	1	This bit sets the low-pass filter frequency for the audio-to-vibe signal path:<br>0: 100 Hz<br>1: 125 Hz<br>2: 150 Hz<br>3: 200 Hz

8.6.12 Audio-to-Vibe Minimum Input Level (Address: 0x12)

Figure 41. Audio-to-Vibe Minimum Input Level Register

7	6	5	4	3	2	1	0
ATH_MIN_INPUT[7:0]							
R/W-0	R/W-0	R/W-0	R/W-1	R/W-1	R/W-0	R/W-0	R/W-1

LEGEND: R/W = Read/Write; R = Read only; -n = value after reset

Table 15. Audio-to-Vibe Minimum Input Level Register Field Descriptions

BIT	FIELD	TYPE	DEFAULT	DESCRIPTION
7-0	ATH_MIN_INPUT[7:0]	R/W	0x19	This bit sets the minimum voltage level at the IN/TRIG pin that is detected by the audio-to-vibe engine. Levels below this are ignored.<br>ATH_MIN_INPUT Voltage (V_{PP}) = ATH_MIN_INPUT[7:0] × 1.8 V / 255

8.6.13 Audio-to-Vibe Maximum Input Level (Address: 0x13)

Figure 42. Audio-to-Vibe Maximum Input Level Register

7	6	5	4	3	2	1	0
ATH_MAX_INPUT[7:0]							
R/W-1							

Table 16. Audio-to-Vibe Maximum Input Level Register Field Descriptions

BIT	FIELD	TYPE	DEFAULT	DESCRIPTION
7-0	ATH_MAX_INPUT[7:0]	R/W	0xFF	This bit sets the full-scale voltage level at the IN/TRIG pin for audio-to-vibe mode.<br>ATH_MAX_INPUT Voltage (V_{PP}) = ATH_MAX_INPUT[7:0] × 1.8 V / 255

8.6.14 Audio-to-Vibe Minimum Output Drive (Address: 0x14)

Copyright © 2014–2018, Texas Instruments Incorporated

Figure 43. Audio-to-Vibe Minimum Output Drive Register

7	6	5	4	3	2	1	0
ATH_MIN_DRIVE[7:0]							
R/W-0	R/W-0	R/W-0	R/W-1	R/W-1	R/W-0	R/W-0	R/W-1

Table 17. Audio-to-Vibe Minimum Output Drive Register Field Descriptions

BIT	FIELD	TYPE	DEFAULT	DESCRIPTION
7-0	ATH_MIN_DRIVE[7:0]	R/W	0x19	This bit sets the minimum output level that is applied to the actuator drive engine.<br>ATH_MIN_DRIVE (%) = ATH_MIN_DRIVE[7:0] / 255 × 100%

8.6.15 Audio-to-Vibe Maximum Output Drive (Address: 0x15)

Figure 44. Audio-to-Vibe Maximum Output Drive Register

7	6	5	4	3	2	1	0
ATH_MAX_DRIVE[7:0]							
R/W-1							

Table 18. Audio-to-Vibe Maximum Output Drive Register Field Descriptions

BIT	FIELD	TYPE	DEFAULT	DESCRIPTION
7-0	ATH_MAX_DRIVE[7:0]	R/W	0xFF	This bit sets the maximum output level that is applied to the actuator drive engine.<br>ATH_MAX_DRIVE (%) = ATH_MAX_DRIVE[7:0] / 255 × 100%

Copyright © 2014–2018, Texas Instruments Incorporated

8.6.16 Rated Voltage (Address: 0x16)

Figure 45. Rated Voltage Register

7	6	5	4	3	2	1	0
RATED_VOLTAGE[7:0]							
R/W-0	R/W-0	R/W-1	R/W-1	R/W-1	R/W-1	R/W-1	R/W-0

Table 19. Rated Voltage Register Field Descriptions

BIT	FIELD	TYPE	DEFAULT	DESCRIPTION
7-0	RATED_VOLTAGE[7:0]	R/W	0x3E	This bit sets the reference voltage for full-scale output during closed-loop operation. The auto-calibration routine uses this register as an input, therefore this register must be written with the rated voltage value of the motor before calibration is performed. This register is ignored for open-loop operation because the overdrive voltage sets the reference for that case. Any modification of this register value should be followed by calibration to set A_CAL_BEMF appropriately.<br>See the *Rated Voltage Programming* section for calculating the correct register value.

8.6.17 Overdrive Clamp Voltage (Address: 0x17)

Figure 46. Overdrive Clamp Voltage Register

7	6	5	4	3	2	1	0
OD_CLAMP[7:0]							
R/W-1	R/W-0	R/W-0	R/W-1	R/W-1	R/W-1	R/W-0	R/W-0

Table 20. Overdrive Clamp Voltage Register Field Descriptions

BIT	FIELD	TYPE	DEFAULT	DESCRIPTION
7	OD_CLAMP[7:0]	R/W	0x8C	During closed-loop operation the actuator feedback allows the output voltage to go above the rated voltage during the automatic overdrive and automatic braking periods. This register sets a clamp so that the automatic overdrive is bounded. This bit also serves as the full-scale reference voltage for open-loop operation.<br>See the *Overdrive Voltage-Clamp Programming* section for calculating the correct register value.

8.6.18 Auto-Calibration Compensation Result (Address: 0x18)

Figure 47. Auto-Calibration Compensation-Result Register

7	6	5	4	3	2	1	0
A_CAL_COMP[7:0]							
R/W-0	R/W-0	R/W-0	R/W-0	R/W-1	R/W-1	R/W-0	R/W-0

Table 21. Auto-Calibration Compensation-Result Register Field Descriptions

BIT	FIELD	TYPE	DEFAULT	DESCRIPTION
7-0	A_CAL_COMP[7:0]	R/W	0x0C	This register contains the voltage-compensation result after execution of auto calibration. The value stored in the A_CAL_COMP bit compensates for any resistive losses in the driver. The calibration routine checks the impedance of the actuator to automatically determine an appropriate value. The auto-calibration compensation-result value is multiplied by the drive gain during playback.<br>Auto-calibration compensation coefficient = 1 + A_CAL_COMP[7:0] / 255

Copyright © 2014–2018, Texas Instruments Incorporated

8.6.19 Auto-Calibration Back-EMF Result (Address: 0x19)

Figure 48. Auto-Calibration Back-EMF Result Register

7	6	5	4	3	2	1	0
A_CAL_BEMF[7:0]							
R/W-0	R/W-1	R/W-1	R/W-0	R/W-1	R/W-1	R/W-1	R/W-1

Table 22. Auto-Calibration Back-EMF Result Register Field Descriptions

BIT	FIELD	TYPE	DEFAULT	DESCRIPTION
7-0	A_CAL_BEMF[7:0]	R/W	0x6F	This register contains the rated back-EMF result after execution of auto calibration. The A_CAL_BEMF[7:0] bit is the level of back-EMF voltage that the actuator gives when the actuator is driven at the rated voltage. The DRV2605L playback engine uses this the value stored in this bit to automatically determine the appropriate feedback gain for closed-loop operation.<br>Auto-calibration back-EMF (V) = (A_CAL_BEMF[7:0] / 255) × 1.22 V / BEMF_GAIN[1:0]

Copyright © 2014–2018, Texas Instruments Incorporated

8.6.20 Feedback Control (Address: 0x1A)

Figure 49. Feedback Control Register

7	6	5	4	3	2	1	0
N_ERM_LRA	FB_BRAKE_FACTOR[2:0]			LOOP_GAIN[1:0]		BEMF_GAIN[1:0]	
R/W-0	R/W-0	R/W-1	R/W-1	R/W-0	R/W-1	R/W-1	R/W-0

Table 23. Feedback Control Register Field Descriptions

BIT	FIELD	TYPE	DEFAULT	DESCRIPTION
7	N_ERM_LRA	R/W	0	This bit sets the DRV2605L device in ERM or LRA mode. This bit should be set prior to running auto calibration.<br>0: ERM Mode<br>1: LRA Mode
6-4	FB_BRAKE_FACTOR[2:0]	R/W	3	This bit selects the feedback gain ratio between braking gain and driving gain. In general, adding additional feedback gain while braking is desirable so that the actuator brakes as quickly as possible. Large ratios provide less-stable operation than lower ones. The advanced user can select to optimize this register. Otherwise, the default value should provide good performance for most actuators. This value should be set prior to running auto calibration.<br>0: 1x<br>1: 2x<br>2: 3x<br>3: 4x<br>4: 6x<br>5: 8x<br>6: 16x<br>7: Braking disabled
3-2	LOOP_GAIN[1:0]	R/W	1	This bit selects a loop gain for the feedback control. The LOOP_GAIN[1:0] bit sets how fast the loop attempts to make the back-EMF (and thus motor velocity) match the input signal level. Higher loop-gain (faster settling) options provide less-stable operation than lower loop gain (slower settling). The advanced user can select to optimize this register. Otherwise, the default value should provide good performance for most actuators. This value should be set prior to running auto calibration.<br>0: Low<br>1: Medium (default)<br>2: High<br>3: Very High
1-0	BEMF_GAIN[1:0]	R/W	2	This bit sets the analog gain of the back-EMF amplifier. This value is interpreted differently between ERM mode and LRA mode. Auto calibration automatically populates the BEMF_GAIN bit with the most appropriate value for the actuator.<br>ERM Mode<br>0: 0.255x<br>1: 0.7875x<br>2: 1.365x (default)<br>3: 3.0x<br>LRA Mode<br>0: 3.75x<br>1: 7.5x<br>2: 15x (default)<br>3: 22.5x

Copyright © 2014–2018, Texas Instruments Incorporated

8.6.21 Control1 (Address: 0x1B)

Figure 50. Control1 Register

7	6	5	4	3	2	1	0
STARTUP_BOOST	Reserved	AC_COUPLE	DRIVE_TIME[4:0]				
R/W-1		R/W-0	R/W-1	R/W-0	R/W-0	R/W-1	R/W-1

Table 24. Control1 Register Field Descriptions

BIT	FIELD	TYPE	DEFAULT	DESCRIPTION
7	STARTUP_BOOST	R/W	1	This bit applies higher loop gain during overdrive to enhance actuator transient response.
6	Reserved			
5	AC_COUPLE	R/W	0	This bit applies a 0.9-V common mode voltage to the IN/TRIG pin when an AC-coupling capacitor is used. This bit is only useful for analog input mode. This bit should not be asserted for PWM mode or external trigger mode.<br>0: Common-mode drive disabled for DC-coupling or digital inputs modes<br>1: Common-mode drive enabled for AC coupling
4-0	DRIVE_TIME[4:0]	R/W	0x13	**LRA Mode:** Sets initial *guess* for LRA drive-time in LRA mode. Drive time is automatically adjusted for optimum drive in real time; however, this register should be optimized for the approximate LRA frequency. If the bit is set too low, it can affect the actuator startup time. If the bit is set too high, it can cause instability.<br>Optimum drive time (ms) ≈ 0.5 × LRA Period<br>Drive time (ms) = DRIVE_TIME[4:0] × 0.1 ms + 0.5 ms<br>**ERM Mode:** Sets the sample rate for the back-EMF detection. Lower drive times cause higher peak-to-average ratios in the output signal, requiring more supply headroom. Higher drive times cause the feedback to react at a slower rate.<br>Drive Time (ms) = DRIVE_TIME[4:0] × 0.2 ms + 1 ms

8.6.22 Control2 (Address: 0x1C)

Figure 51. Control2 Register

7	6	5	4	3	2	1	0
BIDIR_INPUT	BRAKE_STABILIZER	SAMPLE_TIME[1:0]		BLANKING_TIME[1:0]		IDISS_TIME[1:0]	
R/W-1	R/W-1	R/W-1		R/W-0	R/W-1	R/W-0	R/W-1

Table 25. Control2 Register Field Descriptions

BIT	FIELD	TYPE	DEFAULT	DESCRIPTION
7	BIDIR_INPUT	R/W	1	The BIDIR_INPUT bit selects how the engine interprets data.<br>0: Unidirectional input mode<br>Braking is automatically determined by the feedback conditions and is applied when required. Use of this mode also recovers an additional bit of vertical resolution. This mode should only be used for closed-loop operation.<br>**Examples:**:<br>0% Input ? No output signal<br>50% Input ? Half-scale output signal<br>100% Input ? Full-scale output signal<br>1: Bidirectional input mode (default)<br>This mode is compatible with traditional open-loop signaling and also works well with closed-loop mode. When operating closed-loop, braking is automatically determined by the feedback conditions and applied when required. When operating open-loop modes, braking is only applied when the input signal is less than 50%.<br>**Open-loop mode (ERM and LRA) examples:**<br>0% Input ? Negative full-scale output signal (braking)<br>25% Input ? Negative half-scale output signal (braking)<br>50% Input ? No output signal<br>75% Input ? Positive half-scale output signal<br>100% Input ? Positive full-scale output signal<br>**Closed-loop mode (ERM and LRA) examples:**<br>0% to 50% Input ? No output signal<br>50% Input ? No output signal<br>75% Input ? Half-scale output signal<br>100% Input ? Full-scale output signal
6	BRAKE_STABILIZER	R/W	1	When this bit is set, loop gain is reduced when braking is almost complete to improve loop stability
5-4	SAMPLE_TIME[1:0]	R/W	3	LRA auto-resonance sampling time (Advanced use only)<br>0: 150 µs<br>1: 200 µs<br>2: 250 µs<br>3: 300 µs

Copyright © 2014–2018, Texas Instruments Incorporated

Table 25. Control2 Register Field Descriptions (continued)

BIT	FIELD	TYPE	DEFAULT	DESCRIPTION
3-2	BLANKING_TIME[1:0]	R/W	1	Blanking time before the back-EMF AD makes a conversion. (Advanced use only)<br>Blanking time for LRA has an additional 2 bits (BLANKING_TIME[3:2]) located in register 0x1F. Depending on the status of N_ERM_LRA the blanking time represents different values.<br>N_ERM_LRA = 0 (ERM mode)<br>0: 45 µs<br>1: 75 µs<br>2: 150 µs<br>3: 225 µs<br>N_ERM_LRA = 1(LRA mode)<br>0: 15 µs<br>1: 25 µs<br>2: 50 µs<br>3: 75 µs<br>4: 90 µs<br>5: 105 µs<br>6: 120 µs<br>7: 135 µs<br>8: 150 µs<br>9: 165 µs<br>10: 180 µs<br>11: 195 µs<br>12: 210 µs<br>13: 235 µs<br>14: 260 µs<br>15: 285 µs

Table 25. Control2 Register Field Descriptions (continued)

BIT	FIELD	TYPE	DEFAULT	DESCRIPTION
1-0	IDISS_TIME[1:0]	R/W	1	Current dissipation time. This bit is the time allowed for the current to dissipate from the actuator between PWM cycles for flyback mitigation. (Advanced use only)<br>the current dissipation time for LRA has an additional 2 bits (IDISS_TIME[3:2]) located in register 0x1F. Depending on the status of N_ERM_LRA the idiss time represents different values<br>N_ERM_LRA = 0 (ERM mode)<br>0: 45 µs<br>1: 75 µs<br>2: 150 µs<br>3: 225 µs<br>N_ERM_LRA = 1(LRA mode)<br>0: 15 µs<br>1: 25 µs<br>2: 50 µs<br>3: 75 µs<br>4: 90 µs<br>5: 105 µs<br>6: 120 µs<br>7: 135 µs<br>8: 150 µs<br>9: 165 µs<br>10: 180 µs<br>11: 195 µs<br>12: 210 µs<br>13: 235 µs<br>14: 260 µs<br>15: 285 µs

Copyright © 2014–2018, Texas Instruments Incorporated

8.6.23 Control3 (Address: 0x1D)

Figure 52. Control3 Register

7	6	5	4	3	2	1	0
NG_THRESH[1:0]		ERM_OPEN_LOOP	SUPPLY_COMP_DIS	DATA_FORMAT_RTP	LRA_DRIVE_MODE	N_PWM_ANALOG	LRA_OPEN_LOOP
R/W-1	R/W-0	R/W-1	R/W-0	R/W-0	R/W-0	R/W-0	R/W-0

Table 26. Control3 Register Field Descriptions

BIT	FIELD	TYPE	DEFAULT	DESCRIPTION
7-6	NG_THRESH[1:0]	R/W	2	This bit is the noise-gate threshold for PWM and analog inputs.<br>0: Disabled<br>1: 2%<br>2: 4% (Default)<br>3: 8%
5	ERM_OPEN_LOOP	R/W	1	This bit selects mode of operation while in ERM mode. Closed-loop operation is usually desired for because of automatic overdrive and braking properties. However, many existing waveform libraries were designed for open-loop operation, therefore open-loop operation can be required for compatibility.<br>0: Closed Loop<br>1: Open Loop
4	SUPPLY_COMP_DIS	R/W	0	This bit disables supply compensation. The DRV2605L device generally provides constant drive output over variation in the power supply input (V_{DD}). In some systems, supply compensation can have already been implemented upstream, therefore disabling the DRV2605L supply compensation can be useful.<br>0: Supply compensation enabled<br>1: Supply compensation disabled
3	DATA_FORMAT_RTP	R/W	0	This bit selects the input data interpretation for RTP (Real-Time Playback) mode.<br>0: Signed<br>1: Unsigned
2	LRA_DRIVE_MODE	R/W	0	This bit selects the drive mode for the LRA algorithm. This bit determines how often the drive amplitude is updated. Updating once per cycle provides a symmetrical output signal, while updating twice per cycle provides more precise control.<br>0: Once per cycle<br>1: Twice per cycle
1	N_PWM_ANALOG	R/W	0	This bit selects the input mode for the IN/TRIG pin when MODE[2:0] = 3. In PWM input mode, the duty cycle of the input signal determines the amplitude of the waveform. In analog input mode, the amplitude of the input determines the amplitude of the waveform.<br>0: PWM Input<br>1: Analog Input
0	LRA_OPEN_LOOP	R/W	0	This bit selects an open-loop drive option for LRA Mode. When asserted, the playback engine drives the LRA at the selected frequency independently of the resonance frequency. In PWM input mode, the playback engine recovers the LRA commutation frequency from the PWM input, dividing the frequency by 128. Therefore the PWM input frequency must be equal to 128 times the resonant frequency of the LRA.<br>In RTP, ROM and audio-to-vibe mode, the frequency is set by the OL_LRA_PERIOD[6:0] bit. Open-loop mode is not supported if analog input mode is selected.<br>0: Auto-resonance mode<br>1: LRA open-loop mode

Copyright © 2014–2018, Texas Instruments Incorporated

8.6.24 Control4 (Address: 0x1E)

Figure 53. Control4 Register

7	6	5	4	3	2	1	0
ZC_DET_TIME[1]	ZC_DET_TIME[0]	AUTO_CAL_TIME[1:0]		Reserved	OTP_STATUS	Reserved	OTP_PROGRAM
R/W-0	R/W-0	R/W-1	R/W-0		R-0		R/W-0

Table 27. Control4 Register Field Descriptions

BIT	FIELD	TYPE	DEFAULT	DESCRIPTION
7-6	ZC_DET_TIME[1:0]	R/W	0	This bit sets the minimum length of time devoted for detecting a zero crossing (advanced use only).<br>0: 100 µs<br>1: 200 µs<br>2: 300 µs<br>3: 390 µs
5-4	AUTO_CAL_TIME[1:0]	R/W	2	This bit sets the length of the auto calibration time. The AUTO_CAL_TIME[1:0] bit should be enough time for the motor acceleration to settle when driven at the RATED_VOLTAGE[7:0] value.<br>0: 150 ms (minimum), 350 ms (maximum)<br>1: 250 ms (minimum), 450 ms (maximum)<br>2: 500 ms (minimum), 700 ms (maximum)<br>3: 1000 ms (minimum), 1200 ms (maximum)
3	Reserved			
2	OTP_STATUS	R	0	OTP Memory status<br>0: OTP Memory has not been programmed<br>1: OTP Memory has been programmed
1	Reserved			
0	OTP_PROGRAM	R/W	0	This bit launches the programming process for one-time programmable (OTP) memory which programs the contents of register 0x16 through 0x1A into nonvolatile memory. This process can only be executed one time per device. See the *Programming On-Chip OTP Memory* section for details.

Copyright © 2014–2018, Texas Instruments Incorporated

8.6.25 Control5 (Address: 0x1F)

Figure 54. Control5 Register

7	6	5	4	3	2	1	0
AUTO_OL_CNT[1:0]		LRA_AUTO_OPEN_LOOP	PLAYBACK_INTERVAL	BLANKING_TIME[3:2]		IDISS_TIME[3:2]	
R/W-1	R/W-0	R/W-0	R/W-0	RW-0	RW-0	RW-0	

Table 28. Control5 Register Field Descriptions

BIT	FIELD	TYPE	DEFAULT	DESCRIPTION
7-6	AUTO_OL_CNT[1:0]	R/W	2	This bit selects number of cycles required to attempt synchronization before transitioning to open loop when the LRA_AUTO_OPEN_LOOP bit is asserted,<br>0: 3 attempts<br>1: 4 attempts<br>2: 5 attempts<br>3: 6 attempts
5	LRA_AUTO_OPEN_LOOP	R/W	0	This bit selects the automatic transition to open-loop drive when a back-EMF signal is not detected (LRA only).<br>0: Never transitions to open loop<br>1: Automatically transitions to open loop
4	PLAYBACK_INTERVAL	R/W	0	This bit selects the memory playback interval.<br>0: 5 ms<br>1: 1 ms
3-2	BLANKING_TIME[3:2]	R/W	0	This bit sets the MSB for the BLANKING_TIME[3:0]. See the BLANKING_TIME[3:0] bit in the *Control2 (Address: 0x1C)* section for details. Advanced use only.
1-0	IDISS_TIME[3:2]	R/W	0	This bit sets the MSB for IDISS_TIME[3:0]. See the IDISS_TIME[1:0] bit in the *Control2 (Address: 0x1C)* section for details. Advanced use only.

8.6.26 LRA Open Loop Period (Address: 0x20)

Figure 55. LRA Open Loop Period Register

7	6	5	4	3	2	1	0
Reserved	OL_LRA_PERIOD[6:0]						
	R/W-0						

Table 29. LRA Open Loop Period Register Field Descriptions

BIT	FIELD	TYPE	DEFAULT	DESCRIPTION
7-0	OL_LRA_PERIOD[6:0]	R/W	0	This bit sets the period to be used for driving an LRA when open-loop mode is selected.<br>LRA open-loop period (µs) = OL_LRA_PERIOD[6:0] × 98.46 µs

8.6.27 $V_{(BAT)}$ Voltage Monitor (Address: 0x21)

Figure 56. $V_{(BAT)}$ Voltage-Monitor Register

7	6	5	4	3	2	1	0
VBAT[7:0]							
R/W-0							

Table 30. $V_{(BAT)}$ Voltage-Monitor Register Field Descriptions

BIT	FIELD	TYPE	DEFAULT	DESCRIPTION
7-0	VBAT[7:0]	R/W	0	This bit provides a real-time reading of the supply voltage at the V_{DD} pin. The device must be actively sending a waveform to take a reading.<br>V_{DD} (V) = VBAT[7:0] × 5.6V / 255

8.6.28 LRA Resonance Period (Address: 0x22)

Figure 57. LRA Resonance-Period Register

7	6	5	4	3	2	1	0
LRA_PERIOD[7:0]							
R/W-0							

Table 31. LRA Resonance-Period Register Field Descriptions

BIT	FIELD	TYPE	DEFAULT	DESCRIPTION
7-0	LRA_PERIOD[7:0]	R/W	0	This bit reports the measurement of the LRA resonance period. The device must be actively sending a waveform to take a reading.<br>LRA period (us) = LRA_Period[7:0] × 98.46 µs

Copyright © 2014–2018, Texas Instruments Incorporated

9 Application and Implementation

NOTE

Information in the following applications sections is not part of the TI component specification, and TI does not warrant its accuracy or completeness. TI's customers are responsible for determining suitability of components for their purposes. Customers should validate and test their design implementation to confirm system functionality.

9.1 Application Information

The typical application for a haptic driver is in a touch-enabled system that already has an application processor which makes the decision on when to execute haptic effects.

The DRV2605L device can be used fully with I^2C communications (either using RTP or the memory interface). A system designer can chose to use external triggers to play low-latency effects (such as from a physical button) or can decide to use the PWM interface. Figure 58 shows a typical haptic system implementation. The system designer should not use the internal regulator (REG) to power any external load.

A system designer can also implement audio-to-vibe. Figure 59 shows a typical haptic system implementation supporting audio-to-vibe.

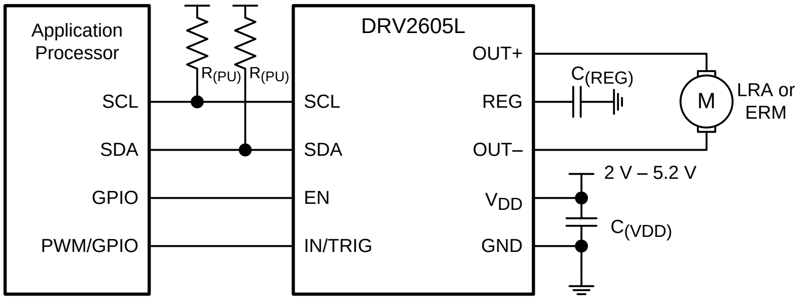

Figure 58. I^2C Control with Optional PWM Input or External Trigger

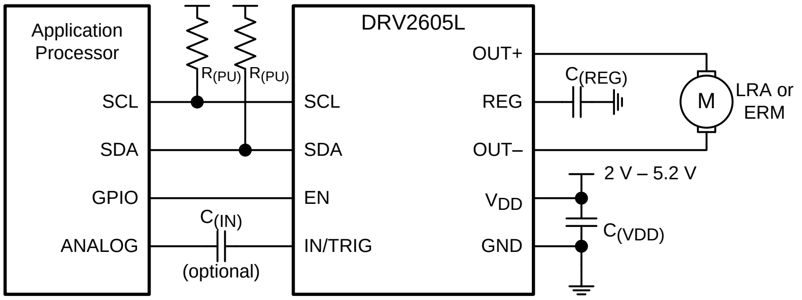

Figure 59. I^2C Control With Audio-to-Vibe Input and Optional AC Coupling

Table 32. Recommended External Components

COMPONENT	DESCRIPTION	SPECIFICATION	TYPICAL VALUE
$C_{(VDD)}$	Input capacitor	Capacitance	1 µF
$C_{(REG)}$	Regulator capacitor	Capacitance	1 µF
$C_{(IN)}$	AC coupling capacitor (optional)	Capacitance	1 µF
$R_{(PU)}$	Pullup resistor	Resistance	2.2 kΩ

Copyright © 2014–2018, Texas Instruments Incorporated

9.2 Typical Application

A typical application of the DRV2605L device is in a system that has external buttons which fire different haptic effects when pressed. Figure 60 shows a typical schematic of such a system. The buttons can be physical buttons, capacitive-touch buttons, or GPIO signals coming from the touch-screen system.

Effects in this type of system are programmable.

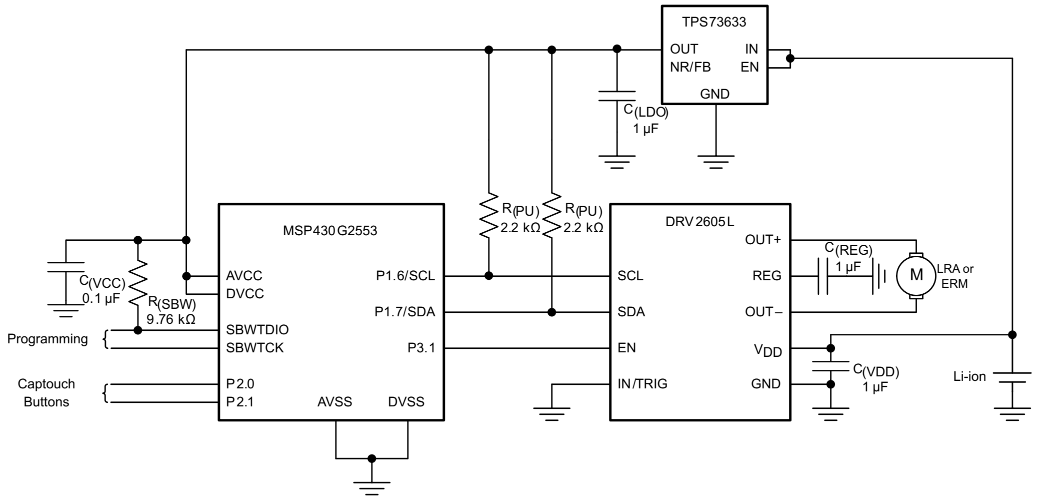

Figure 60. Typical Application Schematic

9.2.1 Design Requirements

For this design example, use the values listed in Table 33 as the input parameters.

Table 33. Design Parameters

DESIGN PARAMETER	EXAMPLE VALUE
Interface	I^2C, external trigger
Actuator type	LRA, ERM
Input power source	Li-ion/Li-polymer, 5-V boost

9.2.2 Detailed Design Procedure

9.2.2.1 Actuator Selection

The actuator decision is based on many factors including cost, form factor, vibration strength, power-consumption requirements, haptic sharpness requirements, reliability, and audible noise performance. The actuator selection is one of the most important design considerations of a haptic system and therefore the actuator should be the first component to consider when designing the system. The following sections list the basics of ERM and LRA actuators.

9.2.2.1.1 Eccentric Rotating-Mass Motors (ERM)

Eccentric rotating-mass motors (ERMs) are typically DC-controlled motors of the bar or coin type. ERMs can be driven in the clockwise direction or counter-clockwise direction depending on the polarity of voltage across the two pins. Bidirectional drive is made possible in a single-supply system by differential outputs that are capable of sourcing and sinking current. The bidirectional drive feature helps eliminate long vibration tails which are undesirable in haptic feedback systems.

Copyright © 2014–2018, Texas Instruments Incorporated

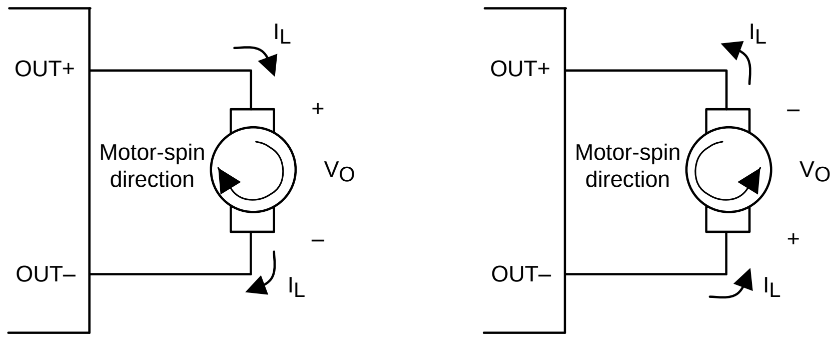

Figure 61. Motor Spin Direction in ERM Motors

Another common approach to driving DC motors is the concept of overdrive voltage. To overcome the inertia of the mass of the motor, the DC motors are often *overdriven* for a short amount of time before returning to the rated voltage of the motor to sustain the rotation of the motor. Overdrive is also used to stop (or brake) a motor quickly. Refer to the data sheet of the particular motor used with the DRV2605L device for safe and reliable overdrive voltage and duration.

9.2.2.1.2 Linear Resonance Actuators (LRA)

Linear resonant actuators (LRAs) vibrate optimally at the resonant frequency. LRAs have a high-Q frequency response because of a rapid drop in vibration performance at the offsets of 3 to 5 Hz from the resonant frequency. Many factors also cause a shift or drift in the resonant frequency of the actuator such as temperature, aging, the mass of the product to which the LRA is mounted, and in the case of a portable product, the manner in which the product is held. Furthermore, as the actuator is driven to the maximum allowed voltage, many LRAs will shift several hertz in frequency because of mechanical compression. All of these factors make a real-time tracking auto-resonant algorithm critical when driving LRA to achieve consistent, optimized performance.

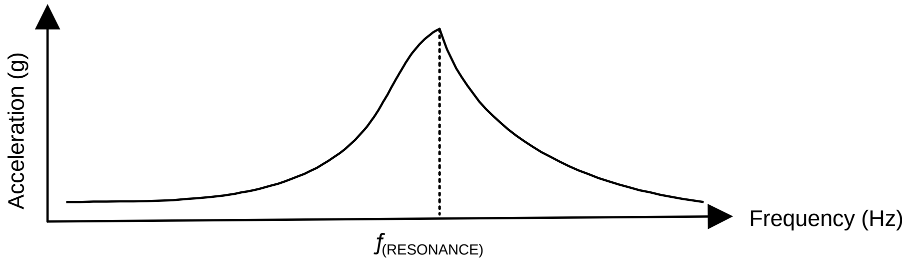

Figure 62. Typical LRA Response

9.2.2.1.2.1 Auto-Resonance Engine for LRA

The DRV2605L auto-resonance engine tracks the resonant frequency of an LRA in real time effectively locking into the resonance frequency after half a cycle. If the resonant frequency shifts in the middle of a waveform for any reason, the engine tracks the frequency from cycle to cycle. The auto resonance engine accomplishes this tracking by constantly monitoring the back-EMF of the actuator. Note that the auto resonance engine is not affected by the auto-calibration process which is only used for level calibration. No calibration is required for the auto resonance engine.

9.2.2.2 Capacitor Selection

The DRV2605L device has a switching output stage which pulls transient currents through the V_{DD} pin. TI recommends placing a 0.1-µF low equivalent-series-resistance (ESR) supply-bypass capacitor of the X5R or X7R type near the V_{DD} supply pin for proper operation of the output driver and the digital portion of the device. Place a 1-µF X5R or X7R-type capacitor from the REG pin to ground.

9.2.2.3 Interface Selection

The I^2C interface is required to configure the device. The device can be used fully with the I^2C interface and with either RTP or internal memory. The advantage of using the I^2C interface is that no additional GPIO (for the IN/TRIG pin) is required for firing effects, and no PWM signal is required to be generated. Therefore the IN/TRIG pin can be connected to GND. Using the external trigger pin has the advantage that no I^2C transaction is required to fire the pre-loaded effect, which is a good choice for interfacing with a button. The PWM interface is available for backward compatibility. If audio-to-vibe is desired, then use $C_{(IN)}$ as shown in Figure 59.

9.2.2.4 Power Supply Selection

The DRV2605L device supports a wide range of voltages in the input. Ensuring that the battery voltage is high enough to support the desired vibration strength with the selected actuator is an important design consideration. The typical application uses Li-ion or Li-polymer batteries which provide enough voltage headroom to drive most common actuators.

If very strong vibrations are desired, a boost converter can be placed between the power supply and the V_{DD} pin to provide a constant voltage with a healthy headroom (5-V rails are common in some systems) which is particularly true if two AA batteries in series are being used to power the system.

9.2.3 Application Curves

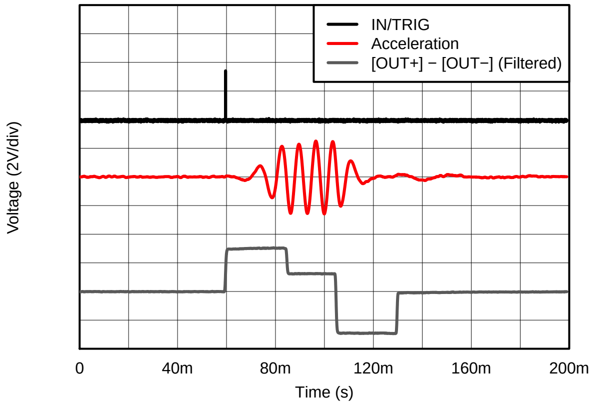

V_{DD} = 3.6 V ERM open loop
Strong click - 60% External edge trigger

Figure 63. ERM Click with and without Braking

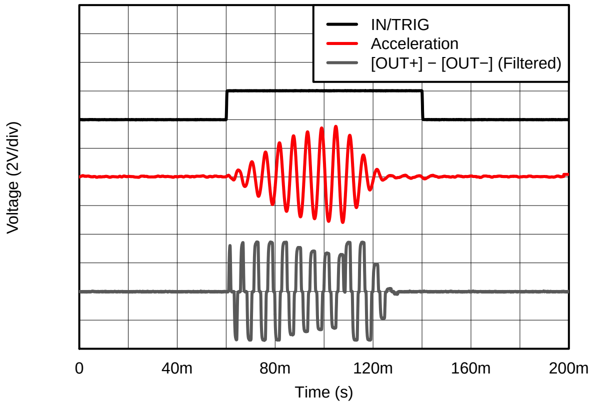

V_{DD} = 3.6 V LRA closed loop
Strong click - 100% External level trigger

Figure 64. LRA Click With and Without Braking

Copyright © 2014–2018, Texas Instruments Incorporated

9.3 Initialization Setup

9.3.1 Initialization Procedure

1. After powerup, wait at least 250 µs before the DRV2605L device accepts I^2C commands.
2. Assert the EN pin (logic high). The EN pin can be asserted any time during or after the 250-µs wait period.
3. Write the MODE register (address 0x01) to value 0x00 to remove the device from standby mode.
4. If the nonvolatile auto-calibration memory has been programmed as described in the *Auto Calibration Procedure* section, skip Step 5 and proceed to Step 6.
5. Perform the steps as described in the *Auto Calibration Procedure* section. Alternatively, rewrite the results from a previous calibration.
6. If using the embedded ROM library, write the library selection register (address 0x03) to select a library.
7. The default setup is closed-loop bidirectional mode. To use other modes and features, write Control1 (0x1B), Control2 (0x1C), and Control3 (0x1D) as required. Open-loop operation is recommended for ERM mode when using the ROM libraries.
8. Put the device in standby mode or deassert the EN pin, whichever is the most convenient. Both settings are low-power modes. The user can select the desired MODE (address 0x01) at the same time the STANDBY bit is set.

9.3.2 Typical Usage Examples

9.3.2.1 Play a Waveform or Waveform Sequence from the ROM Waveform Memory

1. Initialize the device as listed in the *Initialization Procedure* section.
2. Assert the EN pin (active high) if it was previously deasserted.
3. If register 0x01 already holds the desired value and the STANDBY bit is low, the user can skip this step. Select the desired MODE[2:0] value of 0 (internal trigger), 1 (external edge trigger), or 2 (external level trigger) in the MODE register (address 0x01). If the STANDBY bit was previously asserted, this bit should be deasserted (logic low) at this time.
4. Select the waveform index to be played and write it to address 0x04. Alternatively, a sequence of waveform indices can be written to register 0x04 through 0x0B. See the *Waveform Sequencer* section for details.
5. If using the internal trigger mode, set the GO bit (in register 0x0C) to fire the effect or sequence of effects. If using an external trigger mode, send an appropriate trigger pulse to the IN/TRIG pin. See the *Waveform Triggers* section for details.
6. If desired, the user can repeat Step 5 to fire the effect or sequence again.
7. Put the device in low-power mode by deasserting the EN pin or setting the STANDBY bit.

9.3.2.2 Play a Real-Time Playback (RTP) Waveform

1. Initialize the device as shown in the *Initialization Procedure* section.
2. Assert the EN pin (active high) if it was previously deasserted.
3. Set the MODE[2:0] value to 5 (RTP Mode) at address 0x01. If the STANDBY bit was previously asserted, this bit should be deasserted (logic low) at this time. If register 0x01 already holds the desired value and the STANDBY bit is low, the user can skip this step.
4. Write the desired drive amplitude to the real-time playback input register (address 0x02).
5. When the desired sequence of drive amplitudes is complete, put the device in low-power mode by deasserting the EN pin or setting the STANDBY bit.

Copyright © 2014–2018, Texas Instruments Incorporated

Initialization Setup (continued)

9.3.2.3 Play a PWM or Analog Input Waveform

1. Initialize the device as shown in the *Initialization Procedure* section.
2. Assert the EN pin (active high) if it was previously deasserted.
3. If register 0x01 already holds the desired value and the STANDBY bit is low, the user can skip this step. Set the MODE value to 3 (PWM/Analog Mode) at address 0x01. If the STANDBY bit was previously asserted, this bit should be deasserted (logic low) at this time.
4. Select the input mode (PWM or analog) in the Control3 register (address 0x1D). If this mode was selected during the initialization procedure, the user can skip this step.
5. Send the desired PWM or analog input waveform sequence from the external source. See the *Data Formats for Waveform Playback* section for drive amplitude scaling.
6. When the desired drive sequence is complete, put the device in low-power mode by deasserting the EN pin or setting the STANDBY bit.

10 Power Supply Recommendations

The DRV2605L device is designed to operate from an input-voltage supply range between 2 V to 5.2 V. The decoupling capacitor for the power supply should be placed closed to the device pin.

Copyright © 2014–2018, Texas Instruments Incorporated

11 Layout

11.1 Layout Guidelines

Use the following guidelines for the DRV2605L layout:

- The decoupling capacitor for the power supply (V_{DD}) should be placed closed to the device pin.
- The filtering capacitor for the regulator (REG) should be placed close to the device REG pin.
- When creating the pad size for the WCSP pins, TI recommends that the PCB layout use nonsolder mask-defined (NSMD) land. With this method, the solder mask opening is made larger than the desired land area and the opening size is defined by the copper pad width. Figure 65 shows and Table 34 lists appropriate diameters for a wafer-chip scale package (WCSP) layout.

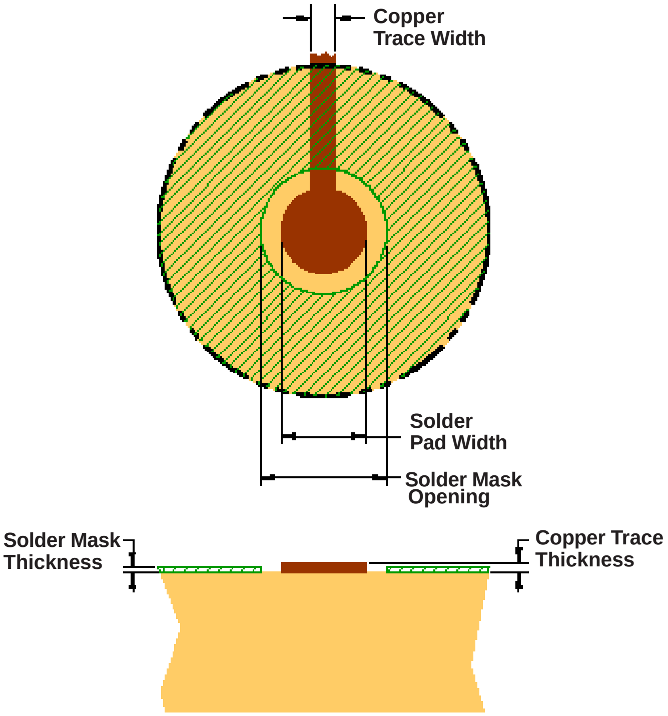

Figure 65. Land Pattern Dimensions

Table 34. Land Pattern Dimensions

SOLDER PAD DEFINITIONS	COPPER PAD	SOLDER MASK OPENING	COPPER THICKNESS	STENCIL OPENING	STENCIL THICKNESS
Nonsolder mask defined (NSMD)	275 µm (0, –25 µm)	375 µm (0, –25 µm)	1-oz maximum (32 µm)	275 µm × 275 µm^2 (rounded corners)	125-µm thick

1. Circuit traces from NSMD defined PWB lands should be 75-µm to 100-µm wide in the exposed area inside the solder mask opening. Wider trace widths reduce device stand-off and impact reliability.
2. The recommended solder paste is Type 3 or Type 4.
3. The best reliability results are achieved when the PWB laminate glass transition temperature is above the operating the range of the intended application.
4. For a PWB using a Ni/Au surface finish, the gold thickness should be less than 0.5 µm to avoid a reduction in thermal fatigue performance.
5. Solder mask thickness should be less than 20 µm on top of the copper circuit pattern.
6. The best solder stencil performance is achieved using laser-cut stencils with electro polishing. Use of chemically-etched stencils results in inferior solder paste volume control.
7. Trace routing away from the WCSP device should be balanced in *X* and *Y* directions to avoid unintentional component movement because of solder-wetting forces.

11.1.1 Trace Width

The recommended trace width at the solder pins is 75 µm to 100 µm to prevent solder wicking onto wider PCB traces. Maintain this trace width until the pin pattern has escaped, then the trace width can be increased for improved current flow. The width and length of the 75-µm to 100-µm traces should be as symmetrical as possible around the device to provide even solder reflow on each of the pins.

11.2 Layout Example

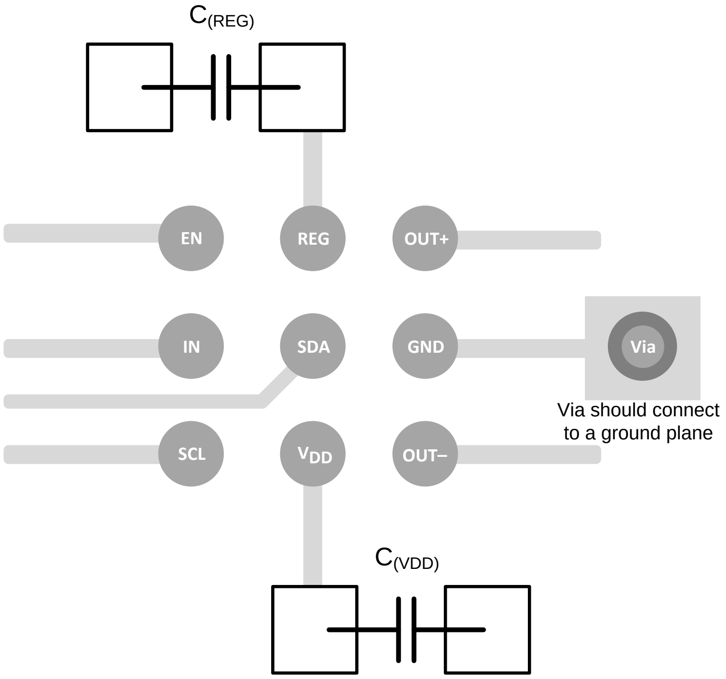

Figure 66. DRV2605L Layout Example DSBGA

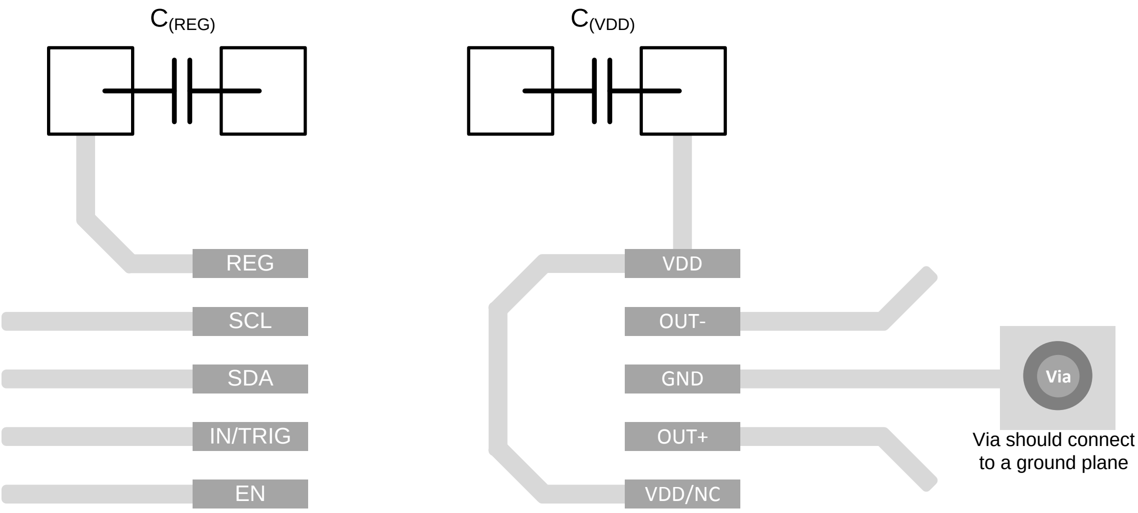

Figure 67. DRV2605L Layout Example VSSOP

Copyright © 2014–2018, Texas Instruments Incorporated

12 Device and Documentation Support

12.1 Device Support

12.1.1 Legal Notice

In order to assist purchasers and users of TI's DRV2605L product, TI has paid a royalty on your behalf to Immersion Corporation to secure your rights to use certain Immersion Corporation software embedded (or designed specifically to be embedded) in TI's DRV2605L product solely as incorporated in TI's DRV2605L product, subject to the terms, conditions and restrictions of TI's license with Immersion Corporation. Subject to the terms, conditions and restrictions of TI's license with Immersion Corporation, you shall not (1) use or distribute any Immersion Corporation software incorporated in TI's DRV2605L product except as incorporated in TI's DRV2605L product in accordance with TI's applicable published specifications and data sheets for the DRV2605L product, (2) modify any Immersion software, (3) change or delete any Immersion proprietary notices, (4) reverse engineer or disassemble any Immersion software or otherwise attempt to discover the internal workings or design of any Immersion software, or (5) distribute Immersion software as a stand-alone basis.

12.1.2 Waveform Library Effects List

EFFECT ID NO.	WAVEFORM NAME	EFFECT ID NO>	WAVEFORM NAME	EFFECT ID NO.	WAVEFORM NAME
1	Strong Click - 100%	42	Long Double Sharp Click Medium 2 – 80%	83	Transition Ramp Up Long Smooth 2 – 0 to 100%
2	Strong Click - 60%	43	Long Double Sharp Click Medium 3 – 60%	84	Transition Ramp Up Medium Smooth 1 – 0 to 100%
3	Strong Click - 30%	44	Long Double Sharp Tick 1 – 100%	85	Transition Ramp Up Medium Smooth 2 – 0 to 100%
4	Sharp Click - 100%	45	Long Double Sharp Tick 2 – 80%	86	Transition Ramp Up Short Smooth 1 – 0 to 100%
5	Sharp Click - 60%	46	Long Double Sharp Tick 3 – 60%	87	Transition Ramp Up Short Smooth 2 – 0 to 100%
6	Sharp Click - 30%	47	Buzz 1 – 100%	88	Transition Ramp Up Long Sharp 1 – 0 to 100%
7	Soft Bump - 100%	48	Buzz 2 – 80%	89	Transition Ramp Up Long Sharp 2 – 0 to 100%
8	Soft Bump - 60%	49	Buzz 3 – 60%	90	Transition Ramp Up Medium Sharp 1 – 0 to 100%
9	Soft Bump - 30%	50	Buzz 4 – 40%	91	Transition Ramp Up Medium Sharp 2 – 0 to 100%
10	Double Click - 100%	51	Buzz 5 – 20%	92	Transition Ramp Up Short Sharp 1 – 0 to 100%
11	Double Click - 60%	52	Pulsing Strong 1 – 100%	93	Transition Ramp Up Short Sharp 2 – 0 to 100%
12	Triple Click - 100%	53	Pulsing Strong 2 – 60%	94	Transition Ramp Down Long Smooth 1 – 50 to 0%
13	Soft Fuzz - 60%	54	Pulsing Medium 1 – 100%	95	Transition Ramp Down Long Smooth 2 – 50 to 0%
14	Strong Buzz - 100%	55	Pulsing Medium 2 – 60%	96	Transition Ramp Down Medium Smooth 1 – 50 to 0%
15	750 ms Alert 100%	56	Pulsing Sharp 1 – 100%	97	Transition Ramp Down Medium Smooth 2 – 50 to 0%
16	1000 ms Alert 100%	57	Pulsing Sharp 2 – 60%	98	Transition Ramp Down Short Smooth 1 – 50 to 0%
17	Strong Click 1 - 100%	58	Transition Click 1 – 100%	99	Transition Ramp Down Short Smooth 2 – 50 to 0%
18	Strong Click 2 - 80%	59	Transition Click 2 – 80%	100	Transition Ramp Down Long Sharp 1 – 50 to 0%
19	Strong Click 3 - 60%	60	Transition Click 3 – 60%	101	Transition Ramp Down Long Sharp 2 – 50 to 0%
20	Strong Click 4 - 30%	61	Transition Click 4 – 40%	102	Transition Ramp Down Medium Sharp 1 – 50 to 0%
21	Medium Click 1 - 100%	62	Transition Click 5 – 20%	103	Transition Ramp Down Medium Sharp 2 – 50 to 0%
22	Medium Click 2 - 80%	63	Transition Click 6 – 10%	104	Transition Ramp Down Short Sharp 1 – 50 to 0%
23	Medium Click 3 - 60%	64	Transition Hum 1 – 100%	105	Transition Ramp Down Short Sharp 2 – 50 to 0%
24	Sharp Tick 1 - 100%	65	Transition Hum 2 – 80%	106	Transition Ramp Up Long Smooth 1 – 0 to 50%
25	Sharp Tick 2 - 80%	66	Transition Hum 3 – 60%	107	Transition Ramp Up Long Smooth 2 – 0 to 50%
26	Sharp Tick 3 – 60%	67	Transition Hum 4 – 40%	108	Transition Ramp Up Medium Smooth 1 – 0 to 50%
27	Short Double Click Strong 1 – 100%	68	Transition Hum 5 – 20%	109	Transition Ramp Up Medium Smooth 2 – 0 to 50%
28	Short Double Click Strong 2 – 80%	69	Transition Hum 6 – 10%	110	Transition Ramp Up Short Smooth 1 – 0 to 50%
29	Short Double Click Strong 3 – 60%	70	Transition Ramp Down Long Smooth 1 – 100 to 0%	111	Transition Ramp Up Short Smooth 2 – 0 to 50%
30	Short Double Click Strong 4 – 30%	71	Transition Ramp Down Long Smooth 2 – 100 to 0%	112	Transition Ramp Up Long Sharp 1 – 0 to 50%
31	Short Double Click Medium 1 – 100%	72	Transition Ramp Down Medium Smooth 1 – 100 to 0%	113	Transition Ramp Up Long Sharp 2 – 0 to 50%
32	Short Double Click Medium 2 – 80%	73	Transition Ramp Down Medium Smooth 2 – 100 to 0%	114	Transition Ramp Up Medium Sharp 1 – 0 to 50%
33	Short Double Click Medium 3 – 60%	74	Transition Ramp Down Short Smooth 1 – 100 to 0%	115	Transition Ramp Up Medium Sharp 2 – 0 to 50%

Copyright © 2014–2018, Texas Instruments Incorporated

Device Support (continued)

EFFECT ID NO.	WAVEFORM NAME	EFFECT ID NO>	WAVEFORM NAME	EFFECT ID NO.	WAVEFORM NAME
34	Short Double Sharp Tick 1 – 100%	75	Transition Ramp Down Short Smooth 2 – 100 to 0%	116	Transition Ramp Up Short Sharp 1 – 0 to 50%
35	Short Double Sharp Tick 2 – 80%	76	Transition Ramp Down Long Sharp 1 – 100 to 0%	117	Transition Ramp Up Short Sharp 2 – 0 to 50%
36	Short Double Sharp Tick 3 – 60%	77	Transition Ramp Down Long Sharp 2 – 100 to 0%	118	Long buzz for programmatic stopping – 100%
37	Long Double Sharp Click Strong 1 – 100%	78	Transition Ramp Down Medium Sharp 1 – 100 to 0%	119	Smooth Hum 1 (No kick or brake pulse) – 50%
38	Long Double Sharp Click Strong 2 – 80%	79	Transition Ramp Down Medium Sharp 2 – 100 to 0%	120	Smooth Hum 2 (No kick or brake pulse) – 40%
39	Long Double Sharp Click Strong 3 – 60%	80	Transition Ramp Down Short Sharp 1 – 100 to 0%	121	Smooth Hum 3 (No kick or brake pulse) – 30%
40	Long Double Sharp Click Strong 4 – 30%	81	Transition Ramp Down Short Sharp 2 – 100 to 0%	122	Smooth Hum 4 (No kick or brake pulse) – 20%
41	Long Double Sharp Click Medium 1 – 100%	82	Transition Ramp Up Long Smooth 1 – 0 to 100%	123	Smooth Hum 5 (No kick or brake pulse) – 10%

12.2 Documentation Support

12.2.1 Related Documentation

For related documentation see the following:

- *Haptic Energy Consumption*, SLOA194
- *Haptic Implementation Considerations for Mobile and Wearable Devices*, SLOA207
- *LRA Actuators: How to Move Them?*, SLOA209

12.3 Receiving Notification of Documentation Updates

To receive notification of documentation updates, navigate to the device product folder on ti.com. In the upper right corner, click on *Alert me* to register and receive a weekly digest of any product information that has changed. For change details, review the revision history included in any revised document.

12.4 Community Resource

The following links connect to TI community resources. Linked contents are provided "AS IS" by the respective contributors. They do not constitute TI specifications and do not necessarily reflect TI's views; see TI's Terms of Use.

TI E2E™ Online Community ***TI's Engineer-to-Engineer (E2E) Community.*** Created to foster collaboration among engineers. At e2e.ti.com, you can ask questions, share knowledge, explore ideas and help solve problems with fellow engineers.

Design Support ***TI's Design Support*** Quickly find helpful E2E forums along with design support tools and contact information for technical support.

12.5 Trademarks

E2E is a trademark of Texas Instruments.
TouchSense is a registered trademark of Immersion Corporation.
All other trademarks are the property of their respective owners.

12.6 Electrostatic Discharge Caution

This integrated circuit can be damaged by ESD. Texas Instruments recommends that all integrated circuits be handled with appropriate precautions. Failure to observe proper handling and installation procedures can cause damage.

ESD damage can range from subtle performance degradation to complete device failure. Precision integrated circuits may be more susceptible to damage because very small parametric changes could cause the device not to meet its published specifications.

12.7 Glossary

SLYZ022 — *TI Glossary*.

This glossary lists and explains terms, acronyms, and definitions.

Copyright © 2014–2018, Texas Instruments Incorporated

13 Mechanical, Packaging, and Orderable Information

The following pages include mechanical packaging and orderable information. This information is the most current data available for the designated devices. This data is subject to change without notice and revision of this document. For browser-based versions of this data sheet, refer to the left-hand navigation.

PACKAGING INFORMATION

Orderable Device	Status (1)	Package Type	Package Drawing	Pins	Package Qty	Eco Plan (2)	Lead finish/ Ball material (6)	MSL Peak Temp (3)	Op Temp (°C)	Device Marking (4/5)	Samples
DRV2605LDGSR	ACTIVE	VSSOP	DGS	10	2500	RoHS & Green	NIPDAUAG	Level-2-260C-1 YEAR	-40 to 85	05L	Samples
DRV2605LDGST	ACTIVE	VSSOP	DGS	10	250	RoHS & Green	NIPDAUAG	Level-2-260C-1 YEAR	-40 to 85	05L	Samples
DRV2605LYZFR	ACTIVE	DSBGA	YZF	9	3000	RoHS & Green	SNAGCU	Level-1-260C-UNLIM	-40 to 85	2605L	Samples
DRV2605LYZFT	ACTIVE	DSBGA	YZF	9	250	RoHS & Green	SNAGCU	Level-1-260C-UNLIM	-40 to 85	2605L	Samples

(1) The marketing status values are defined as follows:
ACTIVE: Product device recommended for new designs.
LIFEBUY: TI has announced that the device will be discontinued, and a lifetime-buy period is in effect.
NRND: Not recommended for new designs. Device is in production to support existing customers, but TI does not recommend using this part in a new design.
PREVIEW: Device has been announced but is not in production. Samples may or may not be available.
OBSOLETE: TI has discontinued the production of the device.

(2) **RoHS:** TI defines "RoHS" to mean semiconductor products that are compliant with the current EU RoHS requirements for all 10 RoHS substances, including the requirement that RoHS substance do not exceed 0.1% by weight in homogeneous materials. Where designed to be soldered at high temperatures, "RoHS" products are suitable for use in specified lead-free processes. TI may reference these types of products as "Pb-Free".
RoHS Exempt: TI defines "RoHS Exempt" to mean products that contain lead but are compliant with EU RoHS pursuant to a specific EU RoHS exemption.
Green: TI defines "Green" to mean the content of Chlorine (Cl) and Bromine (Br) based flame retardants meet JS709B low halogen requirements of <=1000ppm threshold. Antimony trioxide based flame retardants must also meet the <=1000ppm threshold requirement.

(3) MSL, Peak Temp. - The Moisture Sensitivity Level rating according to the JEDEC industry standard classifications, and peak solder temperature.

(4) There may be additional marking, which relates to the logo, the lot trace code information, or the environmental category on the device.

(5) Multiple Device Markings will be inside parentheses. Only one Device Marking contained in parentheses and separated by a "~" will appear on a device. If a line is indented then it is a continuation of the previous line and the two combined represent the entire Device Marking for that device.

(6) Lead finish/Ball material - Orderable Devices may have multiple material finish options. Finish options are separated by a vertical ruled line. Lead finish/Ball material values may wrap to two lines if the finish value exceeds the maximum column width.

Important Information and Disclaimer: The information provided on this page represents TI's knowledge and belief as of the date that it is provided. TI bases its knowledge and belief on information provided by third parties, and makes no representation or warranty as to the accuracy of such information. Efforts are underway to better integrate information from third parties. TI has taken and

continues to take reasonable steps to provide representative and accurate information but may not have conducted destructive testing or chemical analysis on incoming materials and chemicals. TI and TI suppliers consider certain information to be proprietary, and thus CAS numbers and other limited information may not be available for release.

In no event shall TI's liability arising out of such information exceed the total purchase price of the TI part(s) at issue in this document sold by TI to Customer on an annual basis.

OTHER QUALIFIED VERSIONS OF DRV2605L :

- Automotive: DRV2605L-Q1

NOTE: Qualified Version Definitions:

- Automotive - Q100 devices qualified for high-reliability automotive applications targeting zero defects

TAPE AND REEL INFORMATION

REEL DIMENSIONS

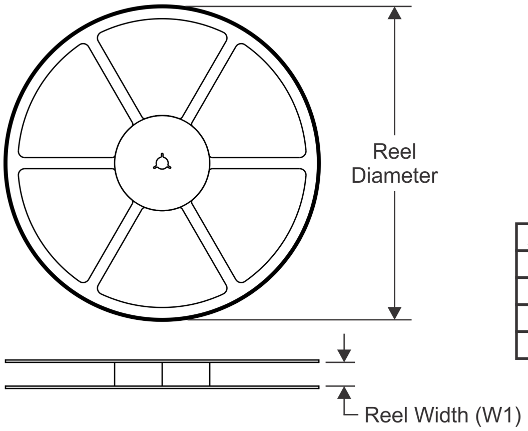

TAPE DIMENSIONS

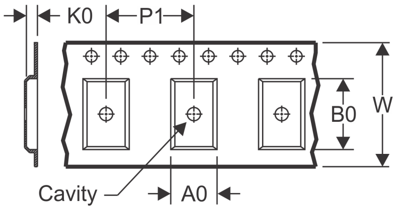

A0	Dimension designed to accommodate the component width
B0	Dimension designed to accommodate the component length
K0	Dimension designed to accommodate the component thickness
W	Overall width of the carrier tape
P1	Pitch between successive cavity centers

QUADRANT ASSIGNMENTS FOR PIN 1 ORIENTATION IN TAPE

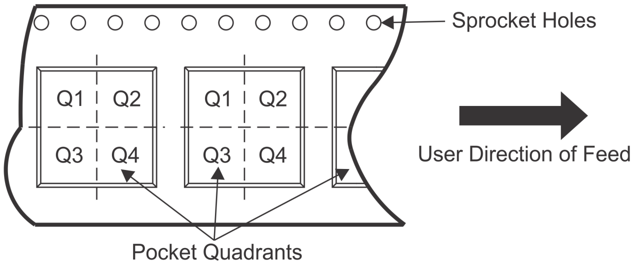

*All dimensions are nominal

Device	Package Type	Package Drawing	Pins	SPQ	Reel Diameter (mm)	Reel Width W1 (mm)	A0 (mm)	B0 (mm)	K0 (mm)	P1 (mm)	W (mm)	Pin1 Quadrant
DRV2605LDGSR	VSSOP	DGS	10	2500	330.0	12.4	5.3	3.4	1.4	8.0	12.0	Q1
DRV2605LDGST	VSSOP	DGS	10	250	330.0	12.4	5.3	3.4	1.4	8.0	12.0	Q1

TAPE AND REEL BOX DIMENSIONS

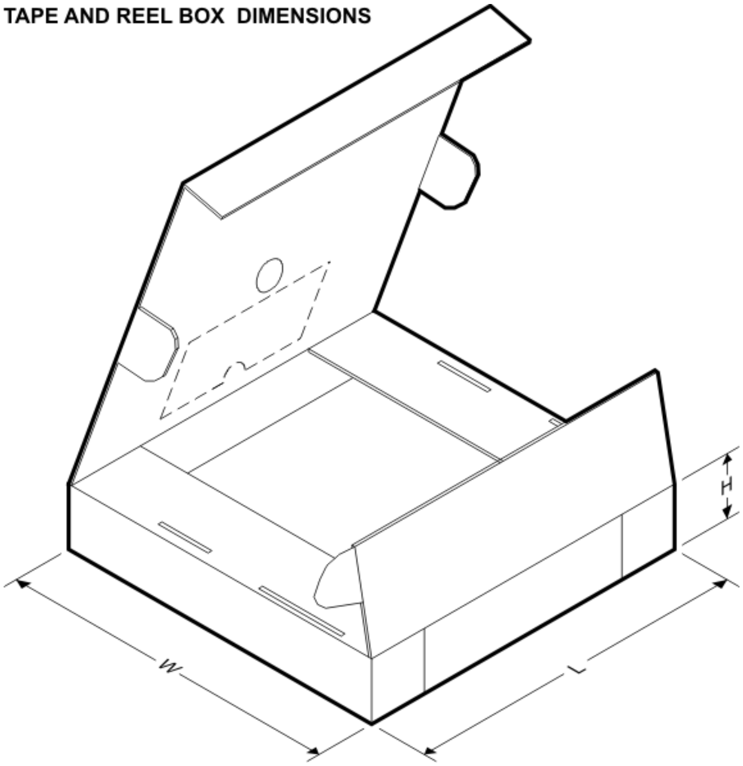

*All dimensions are nominal

Device	Package Type	Package Drawing	Pins	SPQ	Length (mm)	Width (mm)	Height (mm)
DRV2605LDGSR	VSSOP	DGS	10	2500	366.0	364.0	50.0
DRV2605LDGST	VSSOP	DGS	10	250	366.0	364.0	50.0

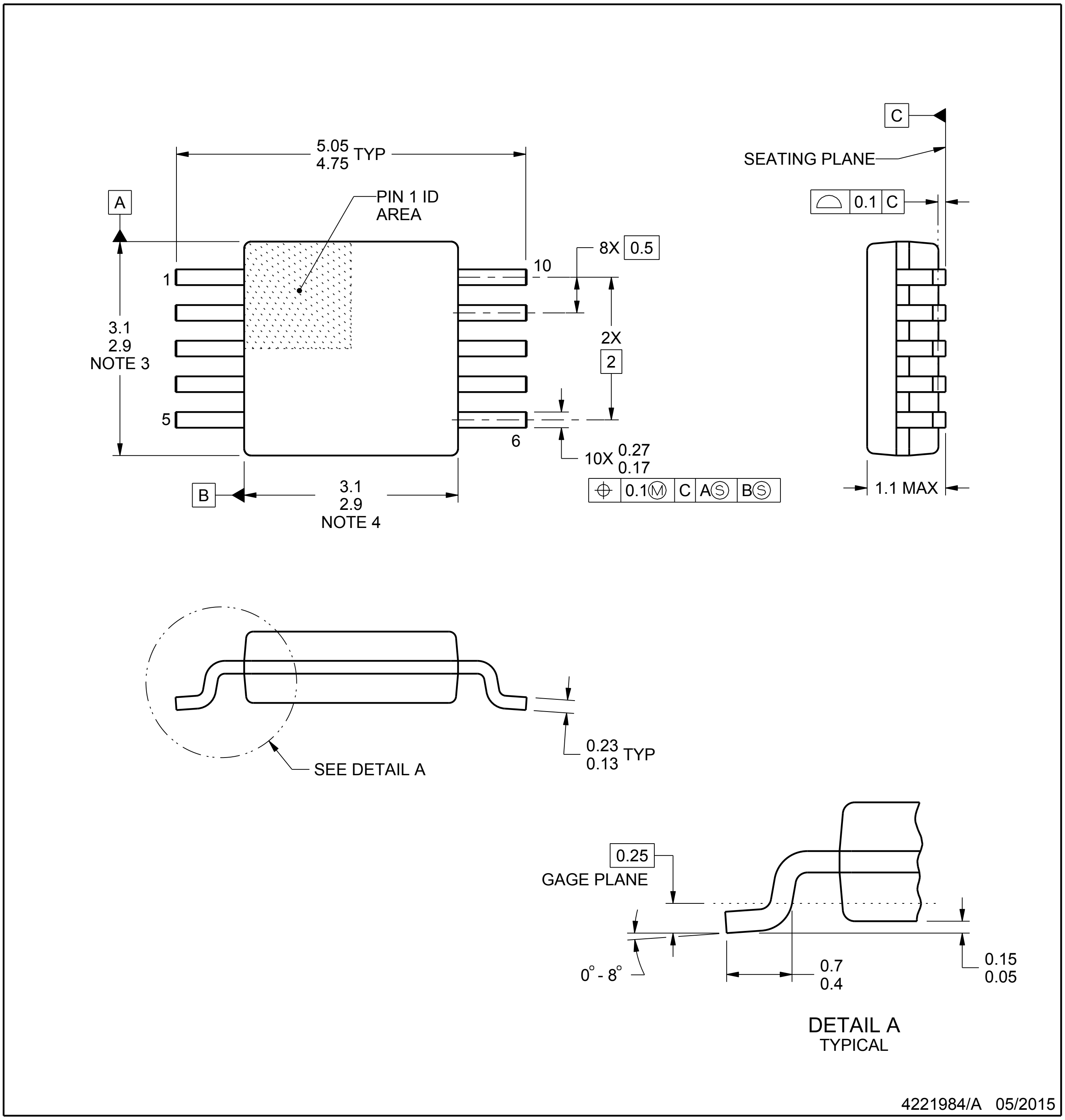

NOTES:

1. All linear dimensions are in millimeters. Any dimensions in parenthesis are for reference only. Dimensioning and tolerancing per ASME Y14.5M.
2. This drawing is subject to change without notice.
3. This dimension does not include mold flash, protrusions, or gate burrs. Mold flash, protrusions, or gate burrs shall not exceed 0.15 mm per side.
4. This dimension does not include interlead flash. Interlead flash shall not exceed 0.25 mm per side.
5. Reference JEDEC registration MO-187, variation BA.

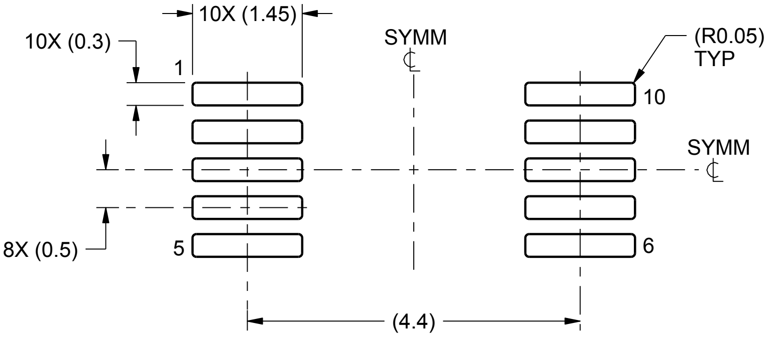

LAND PATTERN EXAMPLE
SCALE:10X

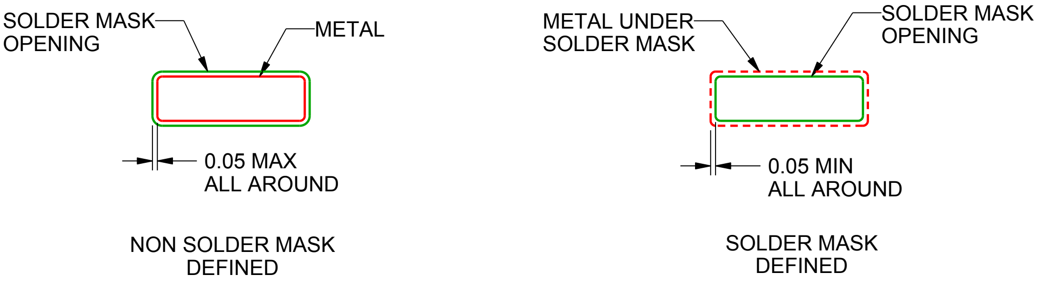

SOLDER MASK DETAILS
NOT TO SCALE

4221984/A 05/2015

NOTES: (continued)

6. Publication IPC-7351 may have alternate designs.
7. Solder mask tolerances between and around signal pads can vary based on board fabrication site.

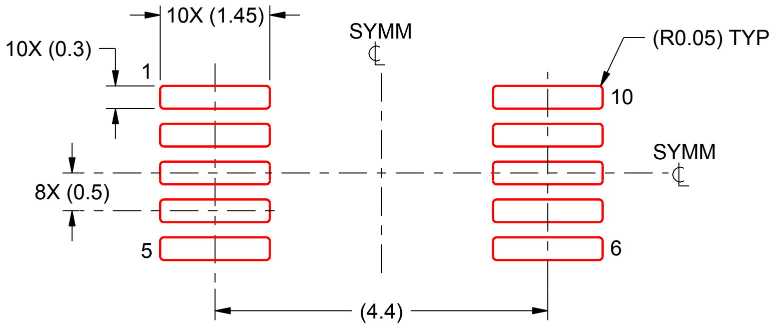

SOLDER PASTE EXAMPLE
BASED ON 0.125 mm THICK STENCIL
SCALE:10X

4221984/A 05/2015

NOTES: (continued)

8. Laser cutting apertures with trapezoidal walls and rounded corners may offer better paste release. IPC-7525 may have alternate design recommendations.
9. Board assembly site may have different recommendations for stencil design.

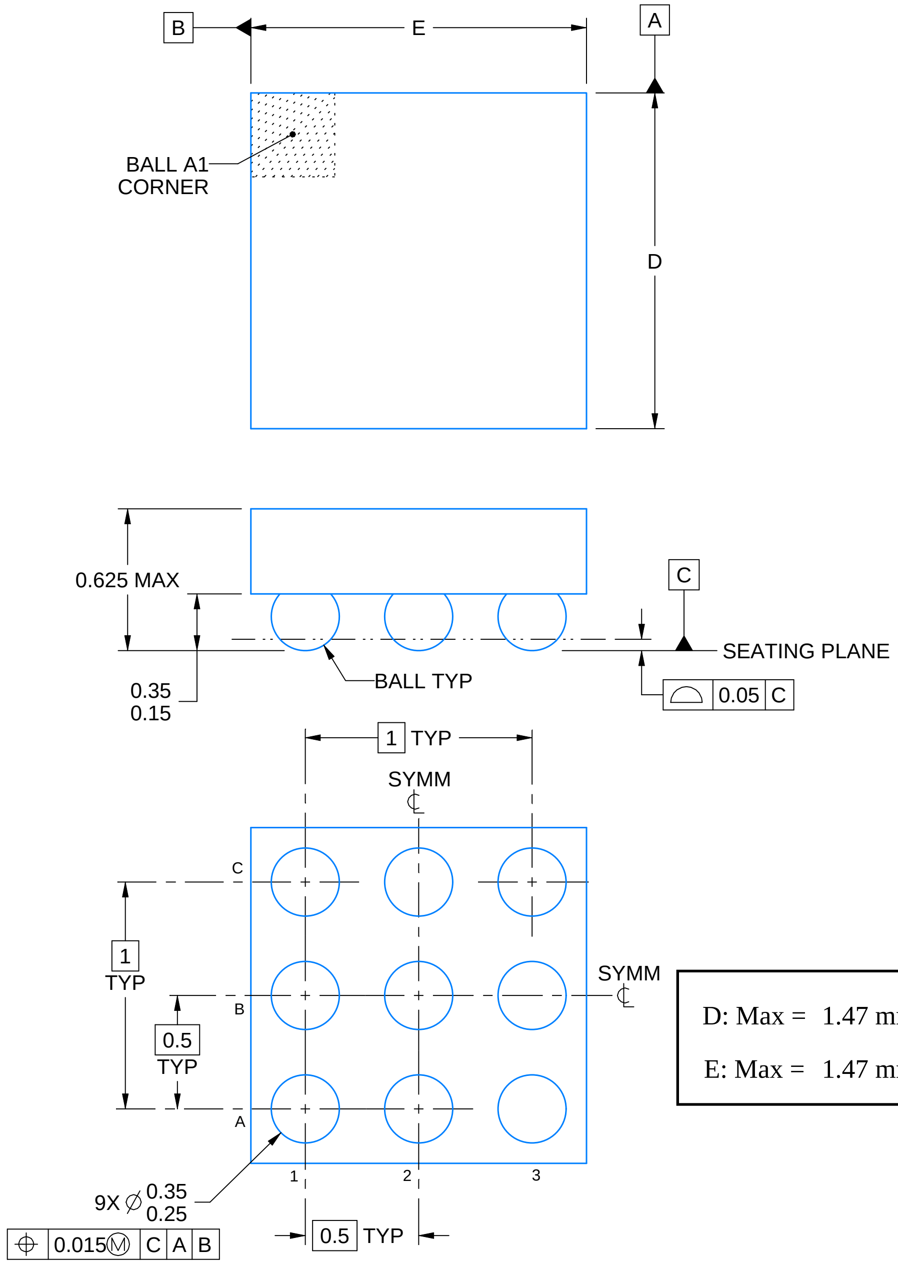

D: Max = 1.47 mm, Min = 1.41 mm	
E: Max = 1.47 mm, Min = 1.41 mm	

4219558/A 10/2018

NOTES:

1. All linear dimensions are in millimeters. Any dimensions in parenthesis are for reference only. Dimensioning and tolerancing per ASME Y14.5M.
2. This drawing is subject to change without notice.

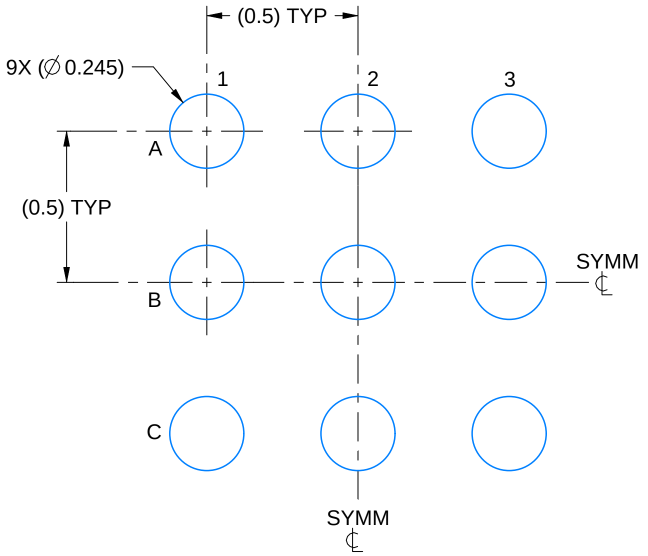

LAND PATTERN EXAMPLE
EXPOSED METAL SHOWN
SCALE: 40X

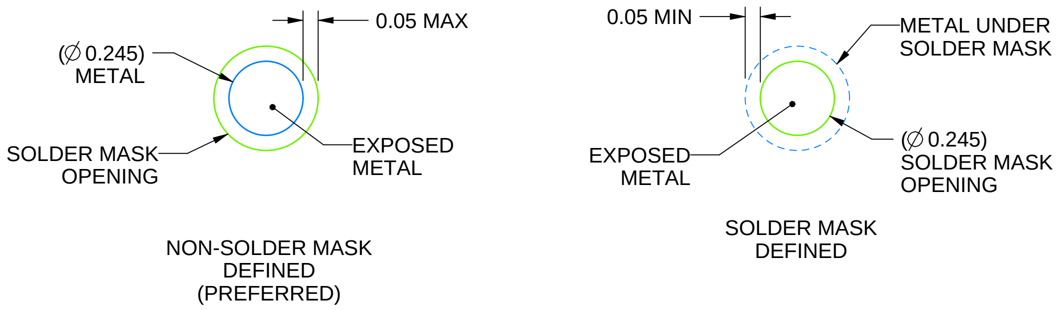

SOLDER MASK DETAILS
NOT TO SCALE

4219558/A 10/2018

NOTES: (continued)

3. Final dimensions may vary due to manufacturing tolerance considerations and also routing constraints. See Texas Instruments Literature No. SNVA009 (www.ti.com/lit/snva009).

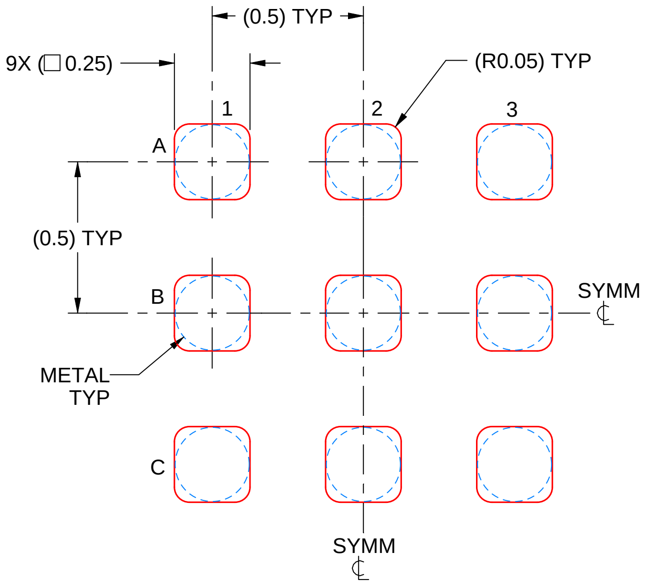

SOLDER PASTE EXAMPLE
BASED ON 0.1 mm THICK STENCIL
SCALE: 40X

4219558/A 10/2018

NOTES: (continued)

4. Laser cutting apertures with trapezoidal walls and rounded corners may offer better paste release.

IMPORTANT NOTICE AND DISCLAIMER

TI PROVIDES TECHNICAL AND RELIABILITY DATA (INCLUDING DATASHEETS), DESIGN RESOURCES (INCLUDING REFERENCE DESIGNS), APPLICATION OR OTHER DESIGN ADVICE, WEB TOOLS, SAFETY INFORMATION, AND OTHER RESOURCES “AS IS” AND WITH ALL FAULTS, AND DISCLAIMS ALL WARRANTIES, EXPRESS AND IMPLIED, INCLUDING WITHOUT LIMITATION ANY IMPLIED WARRANTIES OF MERCHANTABILITY, FITNESS FOR A PARTICULAR PURPOSE OR NON-INFRINGEMENT OF THIRD PARTY INTELLECTUAL PROPERTY RIGHTS.

These resources are intended for skilled developers designing with TI products. You are solely responsible for (1) selecting the appropriate TI products for your application, (2) designing, validating and testing your application, and (3) ensuring your application meets applicable standards, and any other safety, security, or other requirements. These resources are subject to change without notice. TI grants you permission to use these resources only for development of an application that uses the TI products described in the resource. Other reproduction and display of these resources is prohibited. No license is granted to any other TI intellectual property right or to any third party intellectual property right. TI disclaims responsibility for, and you will fully indemnify TI and its representatives against, any claims, damages, costs, losses, and liabilities arising out of your use of these resources.

TI’s products are provided subject to TI’s Terms of Sale (https:www.ti.com/legal/termsofsale.html) or other applicable terms available either on ti.com or provided in conjunction with such TI products. TI’s provision of these resources does not expand or otherwise alter TI’s applicable warranties or warranty disclaimers for TI products.

Mailing Address: Texas Instruments, Post Office Box 655303, Dallas, Texas 75265
Copyright © 2021, Texas Instruments Incorporated
