# Supplementary material for: The Unfolding Space Glove: A Wearable Spatio-Visual to Haptic Sensory Substitution Device for Blind People
Source: Sensors (Basel). 2022 Feb 26;22(5):1859. doi: 10.3390/s22051859 (PMC8914703; doi:10.3390/s22051859)
Supplement: Supplementary file 1 [file sensors-22-01859-s001.zip › S1_GitHub-Release_unfolding-space-hardware-0.2.1-revised.zip!/unfolding-space-hardware-0.2.1-revised/pcb/datasheets/tca9548a.pdf]

TCA9548A Low-Voltage 8-Channel I²C Switch with Reset

1 Features

- 1-to-8 Bidirectional translating switches
- I²C Bus and SMBus compatible
- Active-low reset input
- Three address pins, allowing up to eight TCA9548A devices on the I²C bus
- Channel selection through an I²C Bus, in any combination
- Power up with all switch channels deselected
- Low R_{ON} switches
- Allows voltage-level translation between 1.8-V, 2.5-V, 3.3-V, and 5-V buses
- No glitch on power up
- Supports hot insertion
- Low standby current
- Operating power-supply voltage range of 1.65 V to 5.5 V
- 5-V Tolerant inputs
- 0- to 400-kHz Clock frequency
- Latch-up performance exceeds 100 mA Per JESD 78, class II
- ESD Protection exceeds JESD 22
  - ±2000-V Human-body model (A114-A)
  - 200-V Machine model (A115-A)
  - ±1000-V Charged-device model (C101)

2 Applications

- Servers
- Routers (telecom switching equipment)
- Factory Automation
- Products with I²C slave address conflicts (such as multiple, identical temperature sensors)

3 Description

The TCA9548A device has eight bidirectional translating switches that can be controlled through the I²C bus. The SCL/SDA upstream pair fans out to eight downstream pairs, or channels. Any individual SCn/SDn channel or combination of channels can be selected, determined by the contents of the programmable control register. These downstream channels can be used to resolve I²C slave address conflicts. For example, if eight identical digital temperature sensors are needed in the application, one sensor can be connected at each channel: 0-7.

The system master can reset the TCA9548A in the event of a time-out or other improper operation by asserting a low in the RESET input. Similarly, the power-on reset deselects all channels and initializes the I²C/SMBus state machine. Asserting RESET causes the same reset and initialization to occur without powering down the part. This allows recovery should one of the downstream I²C buses get stuck in a low state.

The pass gates of the switches are constructed so that the VCC pin can be used to limit the maximum high voltage, which is passed by the TCA9548A. Limiting the maximum high voltage allows the use of different bus voltages on each pair, so that 1.8-V, 2.5-V or 3.3-V parts can communicate with 5-V parts, without any additional protection. External pullup resistors pull the bus up to the desired voltage level for each channel. All I/O pins are 5-V tolerant.

Device Information(1)

PART NUMBER	PACKAGE	BODY SIZE (NOM)
TCA9548A	TSSOP (24)	7.80 mm × 4.40 mm
	VQFN (24)	4.00 mm × 4.00 mm

(1) For all available packages, see the orderable addendum at the end of the data sheet.

Simplified Application Diagram

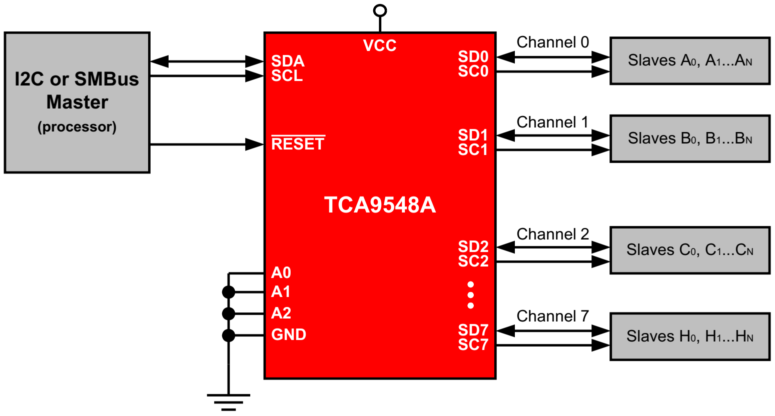

An IMPORTANT NOTICE at the end of this data sheet addresses availability, warranty, changes, use in safety-critical applications, intellectual property matters and other important disclaimers. PRODUCTION DATA.

Table of Contents

1 Features ... 1
2 Applications ... 1
3 Description ... 1
4 Revision History ... 2
5 Pin Configuration and Functions ... 4
6 Specifications ... 5
6.1 Absolute Maximum Ratings ... 5
6.2 ESD Ratings ... 5
6.3 Recommended Operating Conditions ... 5
6.4 Thermal Information ... 5
6.5 Electrical Characteristics ... 6
6.6 I^{2}C Interface Timing Requirements ... 7
6.7 Reset Timing Requirements ... 8
6.8 Switching Characteristics ... 8
6.9 Typical Characteristics ... 9
7 Parameter Measurement Information ... 10
8 Detailed Description ... 12
8.1 Overview ... 12
8.2 Functional Block Diagram ... 13
8.3 Feature Description ... 14
8.4 Device Functional Modes ... 14
8.5 Programming ... 14
9 Application and Implementation ... 20
9.1 Application Information ... 20
9.2 Typical Application ... 20
10 Power Supply Recommendations ... 24
10.1 Power-On Reset Requirements ... 24
11 Layout ... 26
11.1 Layout Guidelines ... 26
11.2 Layout Example ... 26
12 Device and Documentation Support ... 27
12.1 Documentation Support ... 27
12.2 Receiving Notification of Documentation Updates 27
12.3 Support Resources ... 27
12.4 Trademarks ... 27
12.5 Electrostatic Discharge Caution ... 27
12.6 Glossary ... 27
13 Mechanical, Packaging, and Orderable Information ... 27

4 Revision History

NOTE: Page numbers for previous revisions may differ from page numbers in the current version.

Changes from Revision F (November 2016) to Revision G — **Page**

- Changed the appearance of the PW package and the RGE package images ... 4
- Changed T_J from 90 C to 130 C in lower voltage V_{CC} conditions ... 5
- Changed T_A from 85 C to 125C for lower voltage V_{CC} conditions ... 5
- Changed From: V_{CC} = 2.3 V to 3.6 V To: V_{CC} = 1.65 V to 5.5 V in the *Electrical Characteristics* conditions ... 6
- Changed V_O min from 0.9V to 0.6 V ... 6
- Added standby mode specifications for > 85 C T_A ... 6
- Changed R_L = 1 kW To: R_L = 1 KΩ in Figure 6 ... 11

Changes from Revision E (October 2015) to Revision F — **Page**

- Updated the *Description* section ... 1
- Added new orderable part number, TCA9548AMRGER ... 1

Changes from Revision D (January 2015) to Revision E — **Page**

- Updated Pin Functions table. ... 4
- Added new I^{2}C Sections and read/write description ... 16

Changes from Revision C (November 2013) to Revision D — **Page**

- Added *Pin Configuration and Functions* section, *ESD Ratings* table, *Feature Description* section, *Device Functional Modes*, *Application and Implementation* section, *Power Supply Recommendations* section, *Layout* section, *Device and Documentation Support* section, and *Mechanical, Packaging, and Orderable Information* section ... 1
- Updated Typical Application schematic. ... 21

Copyright © 2012–2019, Texas Instruments Incorporated

Changes from Revision B (November 2013) to Revision C	Page
• Updated V_{POR} and I_{CC} standby specification.	6

Changes from Revision A (July 2012) to Revision B	Page
• Updated document formatting.	1
• Removed ordering information.	1

5 Pin Configuration and Functions

PW Package
24-Pin TSSOP
Top View

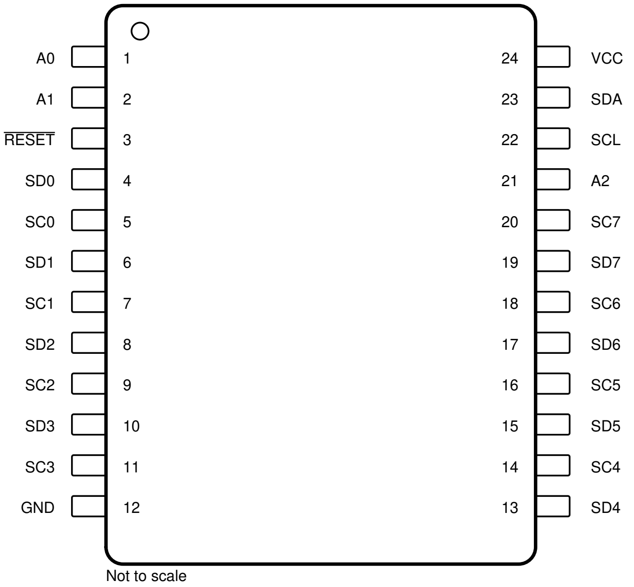

RGE Package
24-Pin VQFN
Top View

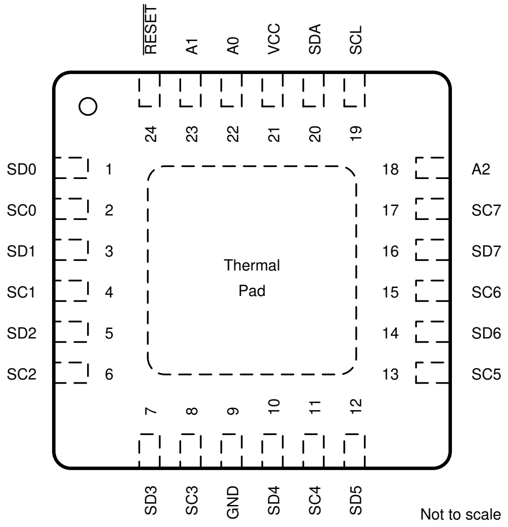

Pin Functions

PIN			TYPE	DESCRIPTION
NAME	TSSOP (PW)	QFN (RGE)		
A0	1	22	I	Address input 0. Connect directly to V_{CC} or ground
A1	2	23	I	Address input 1. Connect directly to V_{CC} or ground
A2	21	18	I	Address input 2. Connect directly to V_{CC} or ground
GND	12	9	—	Ground
RESET	3	24	I	Active-low reset input. Connect to V_{CC} or V_{DPUM} (1) through a pull-up resistor, if not used
SD0	4	1	I/O	Serial data 0. Connect to V_{DPU0} (1) through a pull-up resistor
SC0	5	2	I/O	Serial clock 0. Connect to V_{DPU0} (1) through a pull-up resistor
SD1	6	3	I/O	Serial data 1. Connect to V_{DPU1} (1) through a pull-up resistor
SC1	7	4	I/O	Serial clock 1. Connect to V_{DPU1} (1) through a pull-up resistor
SD2	8	5	I/O	Serial data 2. Connect to V_{DPU2} (1) through a pull-up resistor
SC2	9	6	I/O	Serial clock 2. Connect to V_{DPU2} (1) through a pull-up resistor
SD3	10	7	I/O	Serial data 3. Connect to V_{DPU3} (1) through a pull-up resistor
SC3	11	8	I/O	Serial clock 3. Connect to V_{DPU3} (1) through a pull-up resistor
SD4	13	10	I/O	Serial data 4. Connect to V_{DPU4} (1) through a pull-up resistor
SC4	14	11	I/O	Serial clock 4. Connect to V_{DPU4} (1) through a pull-up resistor
SD5	15	12	I/O	Serial data 5. Connect to V_{DPU5} (1) through a pull-up resistor
SC5	16	13	I/O	Serial clock 5. Connect to V_{DPU5} (1) through a pull-up resistor
SD6	17	14	I/O	Serial data 6. Connect to V_{DPU6} (1) through a pull-up resistor
SC6	18	15	I/O	Serial clock 6. Connect to V_{DPU6} (1) through a pull-up resistor
SD7	19	16	I/O	Serial data 7. Connect to V_{DPU7} (1) through a pull-up resistor
SC7	20	17	I/O	Serial clock 7. Connect to V_{DPU7} (1) through a pull-up resistor
SCL	22	19	I/O	Serial clock bus. Connect to V_{DPUM} (1) through a pull-up resistor
SDA	23	20	I/O	Serial data bus. Connect to V_{DPUM} (1) through a pull-up resistor
VCC	24	21	Power	Supply voltage

(1) V_{DPUX} is the pull-up reference voltage for the associated data line. V_{DPUM} is the master I^2C reference voltage and V_{DPU0}-V_{DPU7} are the slave channel reference voltages.

Copyright © 2012–2019, Texas Instruments Incorporated

6 Specifications

6.1 Absolute Maximum Ratings(1)

over operating free-air temperature range (unless otherwise noted)

			MIN	MAX	UNIT
V_{CC}	Supply voltage		–0.5	7	V
V_I	Input voltage(2)		–0.5	7	V
I_I	Input current		–20	20	mA
I_O	Output current		–25		mA
I_{CC}	Supply current		–100	100	mA
T_{stg}	Storage temperature		–65	150	°C
T_J	Max Junction Temperature	V_{CC} ≤ 3.6 V		130	°C
		V_{CC} ≤ 5.5 V		90	

(1) Stresses beyond those listed under *Absolute Maximum Ratings* may cause permanent damage to the device. These are stress ratings only, which do not imply functional operation of the device at these or any other conditions beyond those indicated under *Recommended Operating Conditions*. Exposure to absolute-maximum-rated conditions for extended periods may affect device reliability.
(2) The input negative-voltage and output voltage ratings may be exceeded if the input and output current ratings are observed.

6.2 ESD Ratings

			VALUE	UNIT
$V_{(ESD)}$	Electrostatic discharge	Human body model (HBM), per ANSI/ESDA/JEDEC JS-001(1)	±2000	V
		Charged-device model (CDM), per JEDEC specification JESD22-C101(2)	±1000	

(1) JEDEC document JEP155 states that 500-V HBM allows safe manufacturing with a standard ESD control process.
(2) JEDEC document JEP157 states that 250-V CDM allows safe manufacturing with a standard ESD control process.

6.3 Recommended Operating Conditions

			MIN	MAX	UNIT
V_{CC}	Supply voltage	-40 °C ≤ T_A ≤ 85 °C	1.65	5.5	V
		85 °C < T_A ≤ 125 °C	1.65	3.6	
V_{IH}	High-level input voltage	SCL, SDA	0.7 × V_{CC}	6	V
		A2–A0, $\overline{RESET}$	0.7 × V_{CC}	V_{CC} + 0.5	
V_{IL}	Low-level input voltage	SCL, SDA	–0.5	0.3 × V_{CC}	V
		A2–A0, $\overline{RESET}$	–0.5	0.3 × V_{CC}	
T_A	Operating free-air temperature	3.6 V < V_{CC} ≤ 5.5 V	–40	85	°C
		1.65 V ≤ V_{CC} ≤ 3.6 V	–40	125	

6.4 Thermal Information

THERMAL METRIC(1)		TCA9548A		UNIT
		PW (TSSOP)	RGE (VQFN)	
		24 PINS	24 PINS	
$R_{\theta JA}$	Junction-to-ambient thermal resistance	108.8	57.2	°C/W
$R_{\theta JC(top)}$	Junction-to-case (top) thermal resistance	54.1	62.5	°C/W
$R_{\theta JB}$	Junction-to-board thermal resistance	62.7	34.4	°C/W
ψ_{JT}	Junction-to-top characterization parameter	10.9	3.8	°C/W
ψ_{JB}	Junction-to-board characterization parameter	62.3	34.4	°C/W
$R_{\theta JC(bot)}$	Junction-to-case (bottom) thermal resistance	N/A	15.5	°C/W

(1) For more information about traditional and new thermal metrics, see the *Semiconductor and IC Package Thermal Metrics* application report.

6.5 Electrical Characteristics[1]

V_{CC} = 1.65 V to 5.5 V, over recommended operating free-air temperature ranges supported by Recommended Operating Conditions (unless otherwise noted)

PARAMETER			TEST CONDITIONS	V_{CC}	MIN	TYP[2]	MAX	UNIT
V_{PORR}	Power-on reset voltage, V_{CC} rising		No load, $V_I = V_{CC}$ or GND[3]			1.2	1.5	V
V_{PORF}	Power-on reset voltage, V_{CC} falling[4]		No load, $V_I = V_{CC}$ or GND[3]		0.8	1		V
$V_{o(sw)}$	Switch output voltage		$V_{i(sw)} = V_{CC}$, I_{SWout} = –100 µA	5 V		3.6		V
				4.5 V to 5.5 V	2.6		4.5	
				3.3 V		1.9		
				3 V to 3.6 V	1.6		2.8	
				2.5 V		1.5		
				2.3 V to 2.7 V	1.1		2	
				1.8 V		1.1		
				1.65 V to 1.95 V	0.6		1.25	
I_{OL}	SDA		V_{OL} = 0.4 V	1.65 V to 5.5 V	3	6		mA
			V_{OL} = 0.6 V		6	9		
I_I	SCL, SDA		$V_I = V_{CC}$ or GND[3]	1.65 V to 5.5 V	–1		1	µA
	SC7–SC0, SD7–SD0				–1		1	
	A2–A0				–1		1	
	$\overline{\text{RESET}}$				–1		1	
I_{CC}	Operating mode	f_{SCL} = 400 kHz	$V_I = V_{CC}$ or GND[3], I_O = 0	5.5 V		50	80	µA
				3.6 V		20	35	
				2.7 V		11	20	
				1.65 V		6	10	
		f_{SCL} = 100 kHz	$V_I = V_{CC}$ or GND[3], I_O = 0	5.5 V		9	30	
				3.6 V		6	15	
				2.7 V		4	8	
				1.65 V		2	4	
	Standby mode	Low inputs	V_I = GND[3], I_O = 0, -40 ℃ ≤ T_A ≤ 85 ℃	5.5 V		0.2	2	
				3.6 V		0.1	2	
				2.7 V		0.1	1	
				1.65 V		0.1	1	
		High inputs	$V_I = V_{CC}$, I_O = 0, -40 ℃ ≤ T_A ≤ 85 ℃	5.5 V		0.2	2	
				3.6 V		0.1	2	
				2.7 V		0.1	1	
				1.65 V		0.1	1	
		Low and High Inputs	$V_I = V_{CC}$ or GND, I_O = 0, 85 ℃ < T_A ≤ 125 ℃	3.6 V		1	2	
				2.7 V		0.7	1.5	
				1.65 V		0.4	1	
ΔI_{CC}	Supply-current change	SCL, SDA	SCL or SDA input at 0.6 V, Other inputs at V_{CC} or GND[3]	1.65 V to 5.5 V		3	20	µA
			SCL or SDA input at V_{CC} – 0.6 V, Other inputs at V_{CC} or GND[3]			3	20	
C_i	A2–A0		$V_I = V_{CC}$ or GND[3]	1.65 V to 5.5 V		4	5	pF
	$\overline{\text{RESET}}$					4	5	
	SCL		$V_I = V_{CC}$ or GND[3], Switch OFF			20	28	

(1) For operation between specified voltage ranges, refer to the worst-case parameter in both applicable ranges.
(2) All typical values are at nominal supply voltage (1.8-, 2.5-, 3.3-, or 5-V V_{CC}), T_A = 25°C.
(3) $\overline{\text{RESET}} = V_{CC}$ (held high) when all other input voltages, V_I = GND.
(4) The power-on reset circuit resets the I^2C bus logic with $V_{CC} < V_{PORF}$.

Copyright © 2012–2019, Texas Instruments Incorporated

Electrical Characteristics(1) (continued)

V_{CC} = 1.65 V to 5.5 V, over recommended operating free-air temperature ranges supported by Recommended Operating Conditions (unless otherwise noted)

PARAMETER		TEST CONDITIONS	V_{CC}	MIN	TYP(2)	MAX	UNIT
$C_{io(off)}$ (5)	SDA	V_I = V_{CC} or GND(3), Switch OFF	1.65 V to 5.5 V		20	28	pF
	SC7–SC0, SD7–SD0				5.5	7.5	
R_{ON}	Switch-on resistance	V_O = 0.4 V, I_O = 15 mA	4.5 V to 5.5 V	4	10	20	Ω
			3 V to 3.6 V	5	12	30	
		V_O = 0.4 V, I_O = 10 mA	2.3 V to 2.7 V	7	15	45	
			1.65 V to 1.95 V	10	25	70	

(5) $C_{io(ON)}$ depends on internal capacitance and external capacitance added to the SCn lines when channels(s) are ON.

6.6 I²C Interface Timing Requirements

over recommended operating free-air temperature range (unless otherwise noted) (see Figure 5)

			MIN	MAX	UNIT
**STANDARD MODE**					
f_{scl}	I²C clock frequency		0	100	kHz
t_{sch}	I²C clock high time		4		µs
t_{scl}	I²C clock low time		4.7		µs
t_{sp}	I²C spike time			50	ns
t_{sds}	I²C serial-data setup time		250		ns
t_{sdh}	I²C serial-data hold time		0(1)		µs
t_{icr}	I²C input rise time			1000	ns
t_{icf}	I²C input fall time			300	ns
t_{ocf}	I²C output (SDn) fall time (10-pF to 400-pF bus)			300	ns
t_{buf}	I²C bus free time between stop and start		4.7		µs
t_{sts}	I²C start or repeated start condition setup		4.7		µs
t_{sth}	I²C start or repeated start condition hold		4		µs
t_{sps}	I²C stop condition setup		4		µs
$t_{vdL(Data)}$	Valid-data time (high to low)(2)	SCL low to SDA output low valid		1	µs
$t_{vdH(Data)}$	Valid-data time (low to high)(2)	SCL low to SDA output high valid		0.6	µs
$t_{vd(ack)}$	Valid-data time of ACK condition	ACK signal from SCL low to SDA output low		1	µs
C_b	I²C bus capacitive load			400	pF
**FAST MODE**					
f_{scl}	I²C clock frequency		0	400	kHz
t_{sch}	I²C clock high time		0.6		µs
t_{scl}	I²C clock low time		1.3		µs
t_{sp}	I²C spike time			50	ns
t_{sds}	I²C serial-data setup time		100		ns
t_{sdh}	I²C serial-data hold time		0(1)		µs
t_{icr}	I²C input rise time		20 + 0.1C_b(3)	300	ns
t_{icf}	I²C input fall time		20 + 0.1C_b(3)	300	ns
t_{ocf}	I²C output (SDn) fall time (10-pF to 400-pF bus)		20 + 0.1C_b(3)	300	ns

(1) A device internally must provide a hold time of at least 300 ns for the SDA signal (referred to the V_{IH} min of the SCL signal), to bridge the undefined region of the falling edge of SCL.
(2) Data taken using a 1-kΩ pull-up resistor and 50-pF load (see Figure 6)
(3) C_b = total bus capacitance of one bus line in pF

Copyright © 2012–2019, Texas Instruments Incorporated

I²C Interface Timing Requirements (continued)

over recommended operating free-air temperature range (unless otherwise noted) (see Figure 5)

			MIN	MAX	UNIT
t_{buf}	I²C bus free time between stop and start		1.3		µs
t_{sts}	I²C start or repeated start condition setup		0.6		µs
t_{sth}	I²C start or repeated start condition hold		0.6		µs
t_{sps}	I²C stop condition setup		0.6		µs
$t_{vdL(Data)}$	Valid-data time (high to low)[(2)]	SCL low to SDA output low valid		1	µs
$t_{vdH(Data)}$	Valid-data time (low to high)[(2)]	SCL low to SDA output high valid		0.6	µs
$t_{vd(ack)}$	Valid-data time of ACK condition	ACK signal from SCL low to SDA output low		1	µs
C_b	I²C bus capacitive load			400	pF

6.7 Reset Timing Requirements

over recommended operating free-air temperature range (unless otherwise noted)

PARAMETER		MIN	MAX	UNIT
$t_{W(L)}$	Pulse duration, $\overline{\text{RESET}}$ low	6		ns
$t_{REC(STA)}$	Recovery time from $\overline{\text{RESET}}$ to start	0		ns

6.8 Switching Characteristics

over recommended operating free-air temperature range, $C_L \le 100$ pF (unless otherwise noted) (see Figure 5)

PARAMETER			FROM (INPUT)	TO (OUTPUT)	MIN	MAX	UNIT
t_{pd} [(1)]	Propagation delay time	$R_{ON} = 20\ \Omega$, $C_L = 15$ pF	SDA or SCL	SDn or SCn		0.3	ns
		$R_{ON} = 20\ \Omega$, $C_L = 50$ pF				1	
t_{rst} [(2)]	$\overline{\text{RESET}}$ time (SDA clear)		$\overline{\text{RESET}}$	SDA		500	ns

(1) The propagation delay is the calculated RC time constant of the typical ON-state resistance of the switch and the specified load capacitance, when driven by an ideal voltage source (zero output impedance).
(2) t_{rst} is the propagation delay measured from the time the $\overline{\text{RESET}}$ pin is first asserted low to the time the SDA pin is asserted high, signaling a stop condition. It must be a minimum of t_{WL}.

Copyright © 2012–2019, Texas Instruments Incorporated

6.9 Typical Characteristics

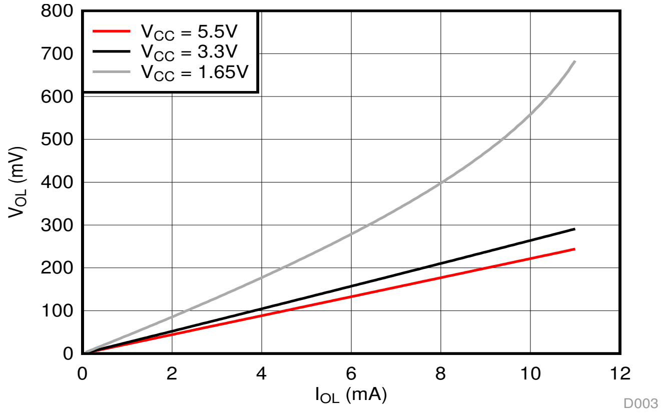

Figure 1. SDA Output Low Voltage (V_{OL}) vs Load Current (I_{OL}) at Three V_{CC} Levels

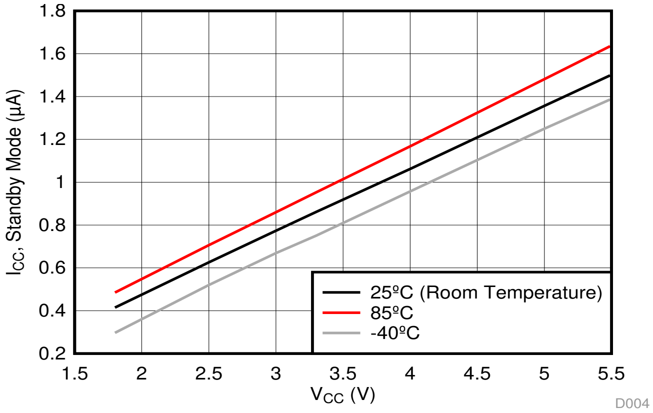

Figure 2. Standby Current (I_{CC}) vs Supply Voltage (V_{CC}) at Three Temperature Points

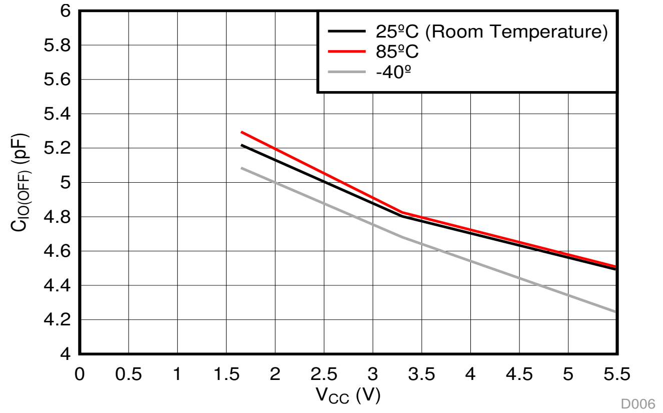

Figure 3. Slave Channel (SCn/SDn) Capacitance ($C_{io(OFF)}$) vs Supply Voltage (V_{CC}) at Three Temperature Points

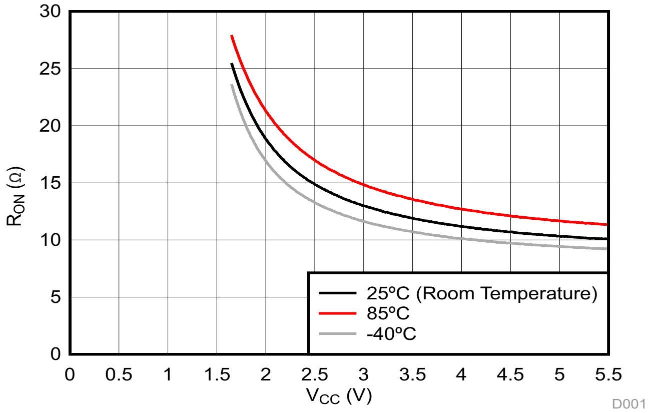

Figure 4. On-Resistance (R_{ON}) vs Supply Voltage (V_{CC}) at Three Temperatures

\

Copyright © 2012–2019, Texas Instruments Incorporated

7 Parameter Measurement Information

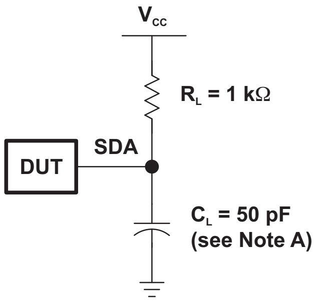

SDA LOAD CONFIGURATION

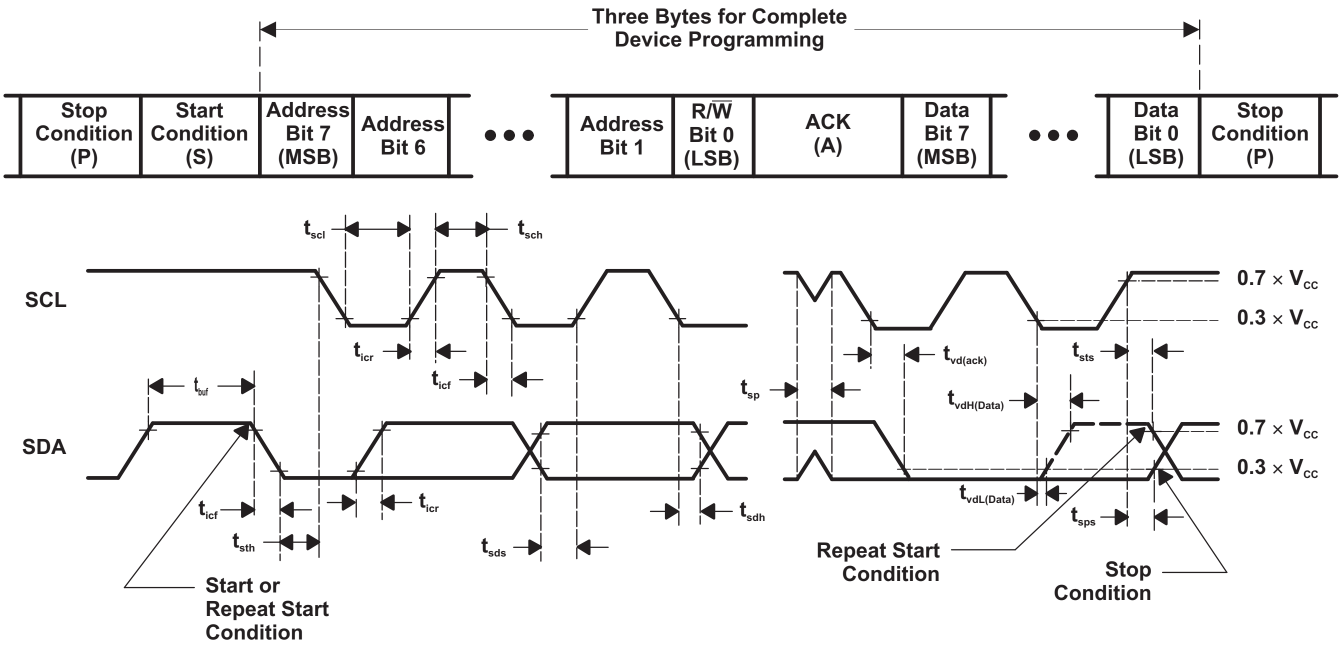

VOLTAGE WAVEFORMS

BYTE	DESCRIPTION
1	I^2C address
2, 3	P-port data

A. C_L includes probe and jig capacitance.

B. All inputs are supplied by generators having the following characteristics: PRR ≤ 10 MHz, Z_O = 50 Ω, t_r/t_f ≤ 30 ns.

C. Not all parameters and waveforms are applicable to all devices.

Figure 5. I^2C Load Circuit and Voltage Waveforms

Copyright © 2012–2019, Texas Instruments Incorporated

Parameter Measurement Information (continued)

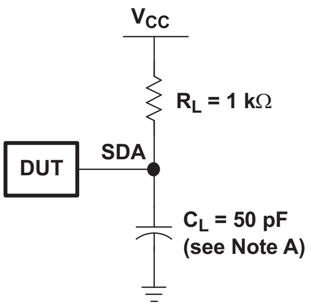

SDA LOAD CONFIGURATION

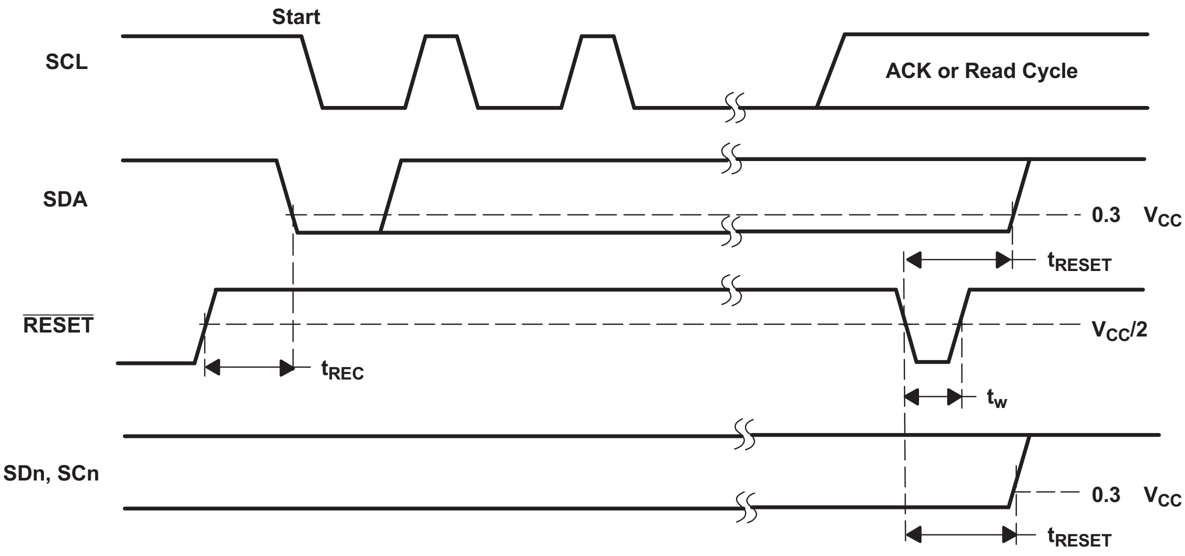

A. C_L includes probe and jig capacitance.

B. All inputs are supplied by generators having the following characteristics: PRR ≤ 10 MHz, Z_O = 50 Ω, t_r/t_f ≤ 30 ns.

C. I/Os are configured as inputs.

D. Not all parameters and waveforms are applicable to all devices.

Figure 6. Reset Load Circuit and Voltage Waveforms

8 Detailed Description

8.1 Overview

The TCA9548A is an 8-channel, bidirectional translating I^2C switch. The master SCL/SDA signal pair is directed to eight channels of slave devices, SC0/SD0-SC7/SD7. Any individual downstream channel can be selected as well as any combination of the eight channels.

The device offers an active-low $\overline{RESET}$ input which resets the state machine and allows the TCA9548A to recover must one of the downstream I^2C buses get stuck in a low state. The state machine of the device can also be reset by cycling the power supply, V_{CC}, also known as a power-on reset (POR). Both the $\overline{RESET}$ function and a POR cause all channels to be deselected.

The connections of the I^2C data path are controlled by the same I^2C master device that is switched to communicate with multiple I^2C slaves. After the successful acknowledgment of the slave address (hardware selectable by A0, A1, and A2 pins), a single 8-bit control register is written to or read from to determine the selected channels.

The TCA9548A may also be used for voltage translation, allowing the use of different bus voltages on each SCn/SDn pair such that 1.8-V, 2.5-V, or 3.3-V parts can communicate with 5-V parts. This is achieved by using external pull-up resistors to pull the bus up to the desired voltage for the master and each slave channel.

Copyright © 2012–2019, Texas Instruments Incorporated

8.2 Functional Block Diagram

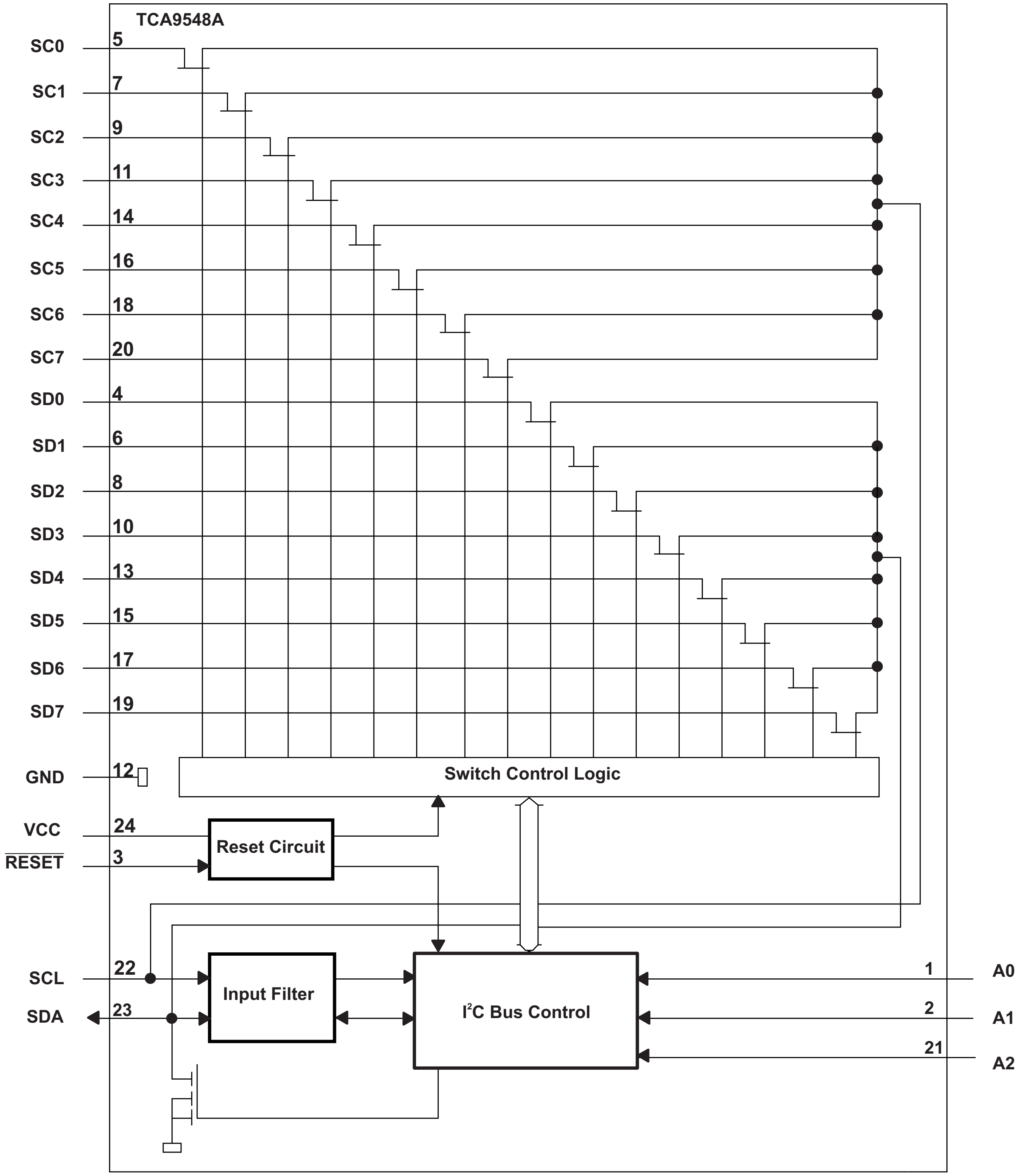

8.3 Feature Description

The TCA9548A is an 8-channel, bidirectional translating switch for I^{2}C buses that supports Standard-Mode (100 kHz) and Fast-Mode (400 kHz) operation. The TCA9548A features I^{2}C control using a single 8-bit control register in which each bit controls the enabling and disabling of one of the corresponding 8 switch channels for I^{2}C data flow. Depending on the application, voltage translation of the I^{2}C bus can also be achieved using the TCA9548A to allow 1.8-V, 2.5-V, or 3.3-V parts to communicate with 5-V parts. Additionally, in the event that communication on the I^{2}C bus enters a fault state, the TCA9548A can be reset to resume normal operation using the $\overline{\text{RESET}}$ pin feature or by a power-on reset which results from cycling power to the device.

8.4 Device Functional Modes

8.4.1 $\overline{\text{RESET}}$ Input

The $\overline{\text{RESET}}$ input is an active-low signal that may be used to recover from a bus-fault condition. When this signal is asserted low for a minimum of t_{WL}, the TCA9548A resets its registers and I^{2}C state machine and deselects all channels. The $\overline{\text{RESET}}$ input must be connected to V_{CC} through a pull-up resistor.

8.4.2 Power-On Reset

When power is applied to the VCC pin, an internal power-on reset holds the TCA9548A in a reset condition until V_{CC} has reached V_{PORR}. At this point, the reset condition is released, and the TCA9548A registers and I^{2}C state machine are initialized to their default states, all zeroes, causing all the channels to be deselected. Thereafter, V_{CC} must be lowered below V_{PORF} to reset the device.

8.5 Programming

8.5.1 I^{2}C Interface

The TCA9548A has a standard bidirectional I^{2}C interface that is controlled by a master device in order to be configured or read the status of this device. Each slave on the I^{2}C bus has a specific device address to differentiate between other slave devices that are on the same I^{2}C bus. Many slave devices require configuration upon startup to set the behavior of the device. This is typically done when the master accesses internal register maps of the slave, which have unique register addresses. A device can have one or multiple registers where data is stored, written, or read.

The physical I^{2}C interface consists of the serial clock (SCL) and serial data (SDA) lines. Both SDA and SCL lines must be connected to V_{CC} through a pull-up resistor. The size of the pull-up resistor is determined by the amount of capacitance on the I^{2}C lines. (For further details, see the *I^{2}C Pull-up Resistor Calculation* application report. Data transfer may be initiated only when the bus is idle. A bus is considered idle if both SDA and SCL lines are high after a STOP condition (See Figure 7 and Figure 8).

The following is the general procedure for a master to access a slave device:

1. If a master wants to send data to a slave:
   – Master-transmitter sends a START condition and addresses the slave-receiver.
   – Master-transmitter sends data to slave-receiver.
   – Master-transmitter terminates the transfer with a STOP condition.
2. If a master wants to receive or read data from a slave:
   – Master-receiver sends a START condition and addresses the slave-transmitter.
   – Master-receiver sends the requested register to read to slave-transmitter.
   – Master-receiver receives data from the slave-transmitter.

Copyright © 2012–2019, Texas Instruments Incorporated

Programming (continued)

– Master-receiver terminates the transfer with a STOP condition.

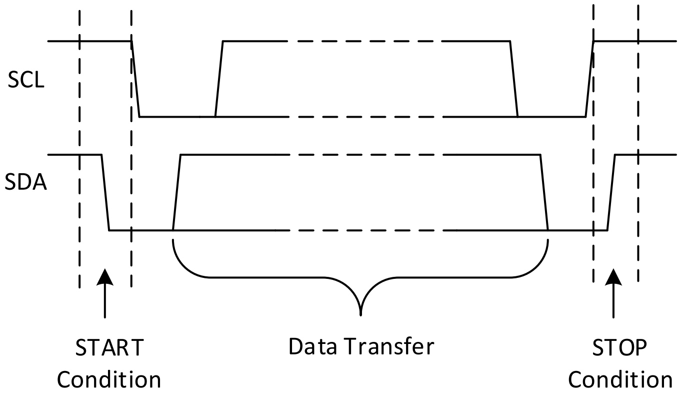

Figure 7. Definition of Start and Stop Conditions

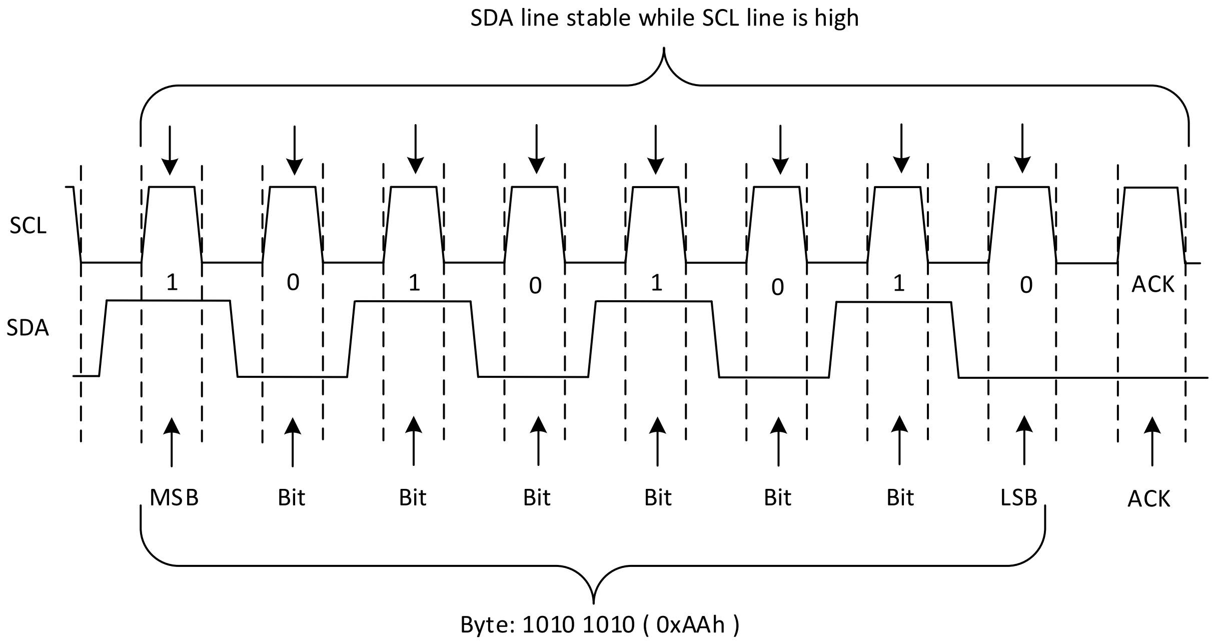

Figure 8. Bit Transfer

8.5.2 Device Address

Figure 9 shows the address byte of the TCA9548A.

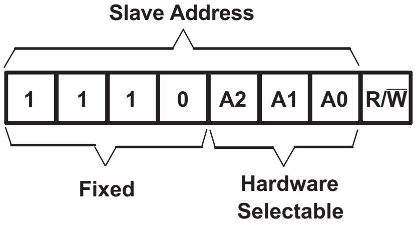

Figure 9. TCA9548A Address

Programming (continued)

The last bit of the slave address defines the operation (read or write) to be performed. When it is high (1), a read is selected, while a low (0) selects a write operation.

Table 1 shows the TCA9548A address reference.

Table 1. Address Reference

INPUTS			I^2C BUS SLAVE ADDRESS
A2	A1	A0	
L	L	L	112 (decimal), 70 (hexadecimal)
L	L	H	113 (decimal), 71 (hexadecimal)
L	H	L	114 (decimal), 72 (hexadecimal)
L	H	H	115 (decimal), 73 (hexadecimal)
H	L	L	116 (decimal), 74 (hexadecimal)
H	L	H	117 (decimal), 75 (hexadecimal)
H	H	L	118 (decimal), 76 (hexadecimal)
H	H	H	119 (decimal), 77 (hexadecimal)

8.5.3 Bus Transactions

Data must be sent to and received from the slave devices, and this is accomplished by reading from or writing to registers in the slave device.

Registers are locations in the memory of the slave which contain information, whether it be the configuration information or some sampled data to send back to the master. The master must write information to these registers in order to instruct the slave device to perform a task.

While it is common to have registers in I^2C slaves, note that not all slave devices have registers. Some devices are simple and contain only 1 register, which may be written to directly by sending the register data immediately after the slave address, instead of addressing a register. The TCA9548A is example of a single-register device, which is controlled via I^2C commands. Since it has 1 bit to enable or disable a channel, there is only 1 register needed, and the master merely writes the register data after the slave address, skipping the register number.

8.5.3.1 Writes

To write on the I^2C bus, the master sends a START condition on the bus with the address of the slave, as well as the last bit (the $R/\overline{W}$ bit) set to 0, which signifies a write. The slave acknowledges, letting the master know it is ready. After this, the master starts sending the control register data to the slave until the master has sent all the data necessary (which is sometimes only a single byte), and the master terminates the transmission with a STOP condition.

There is no limit to the number of bytes sent, but the last byte sent is what is in the register.

Figure 10 shows an example of writing a single byte to a slave register.

Copyright © 2012–2019, Texas Instruments Incorporated

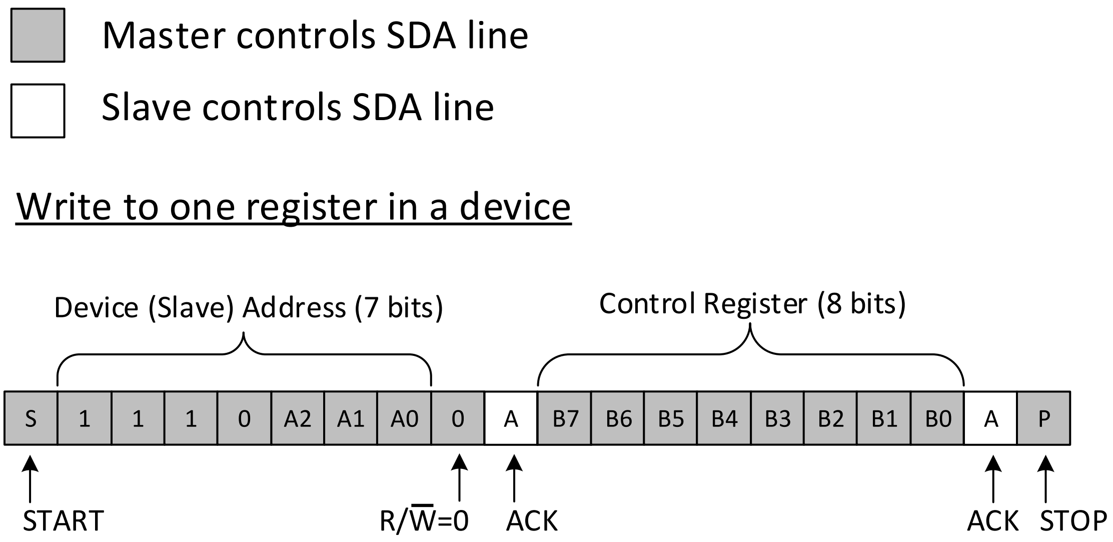

Figure 10. Write to Register

8.5.3.2 Reads

Reading from a slave is very similar to writing, but the master sends a START condition, followed by the slave address with the R/W̄ bit set to 1 (signifying a read). The slave acknowledges the read request, and the master releases the SDA bus but continues supplying the clock to the slave. During this part of the transaction, the master becomes the master-receiver, and the slave becomes the slave-transmitter.

The master continues to send out the clock pulses, but releases the SDA line so that the slave can transmit data. At the end of every byte of data, the master sends an ACK to the slave, letting the slave know that it is ready for more data. Once the master has received the number of bytes it is expecting, it sends a NACK, signaling to the slave to halt communications and release the bus. The master follows this up with a STOP condition.

Figure 11 shows an example of reading a single byte from a slave register.

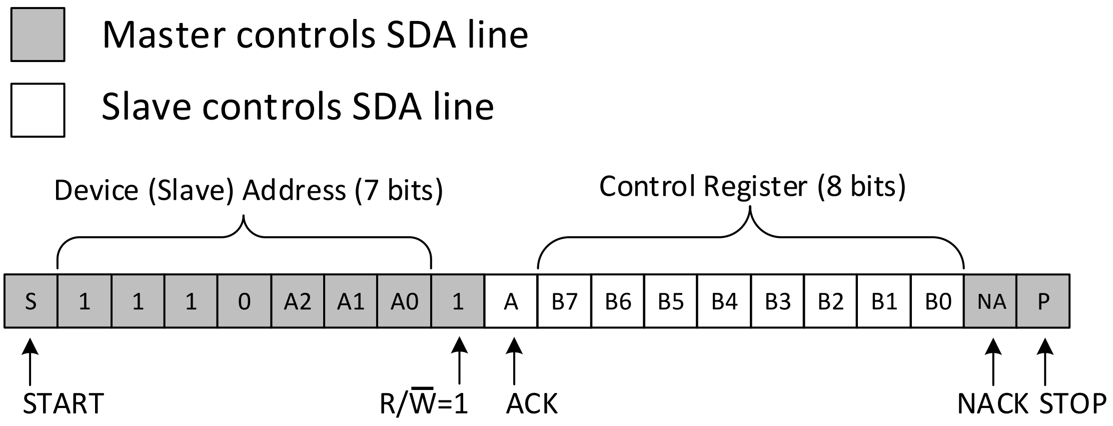

Figure 11. Read from Control Register

8.5.4 Control Register

Following the successful acknowledgment of the address byte, the bus master sends a command byte that is stored in the control register in the TCA9548A (see Figure 12). This register can be written and read via the I^{2}C bus. Each bit in the command byte corresponds to a SCn/SDn channel and a high (or 1) selects this channel. Multiple SCn/SDn channels may be selected at the same time. When a channel is selected, the channel becomes active after a stop condition has been placed on the I^{2}C bus. This ensures that all SCn/SDn lines are in a high state when the channel is made active, so that no false conditions are generated at the time of connection. A stop condition always must occur immediately after the acknowledge cycle. If multiple bytes are received by the TCA9548A, it saves the last byte received.

Channel Selection Bits (Read/Write)

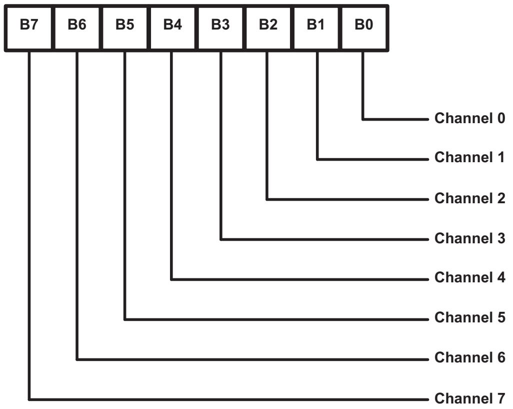

Figure 12. Control Register

Table 2 shows the TCA9548A Command Byte Definition.

Table 2. Command Byte Definition

CONTROL REGISTER BITS								COMMAND
**B7**	**B6**	**B5**	**B4**	**B3**	**B2**	**B1**	**B0**	
X	X	X	X	X	X	X	0	Channel 0 disabled
							1	Channel 0 enabled
X	X	X	X	X	X	0	X	Channel 1 disabled
						1		Channel 1 enabled
X	X	X	X	X	0	X	X	Channel 2 disabled
					1			Channel 2 enabled
X	X	X	X	0	X	X	X	Channel 3 disabled
				1				Channel 3 enabled
X	X	X	0	X	X	X	X	Channel 4 disabled
			1					Channel 4 enabled
X	X	0	X	X	X	X	X	Channel 5 disabled
		1						Channel 5 enabled
X	0	X	X	X	X	X	X	Channel 6 disabled
	1							Channel 6 enabled
0	X	X	X	X	X	X	X	Channel 7 disabled
1								Channel 7 enabled
0	0	0	0	0	0	0	0	No channel selected, power-up/reset default state

Copyright © 2012–2019, Texas Instruments Incorporated

8.5.5 $\overline{\text{RESET}}$ Input

The $\overline{\text{RESET}}$ input is an active-low signal that may be used to recover from a bus-fault condition. When this signal is asserted low for a minimum of t_{WL}, the TCA9548A resets its registers and I^2C state machine and deselects all channels. The $\overline{\text{RESET}}$ input must be connected to V_{CC} through a pull-up resistor.

8.5.6 Power-On Reset

When power (from 0 V) is applied to V_{CC}, an internal power-on reset holds the TCA9548A in a reset condition until V_{CC} has reached V_{POR}. At that point, the reset condition is released and the TCA9548A registers and I^2C state machine initialize to their default states. After that, V_{CC} must be lowered to below V_{POR} and then back up to the operating voltage for a power-reset cycle.

9 Application and Implementation

NOTE

Information in the following applications sections is not part of the TI component specification, and TI does not warrant its accuracy or completeness. TI's customers are responsible for determining suitability of components for their purposes. Customers should validate and test their design implementation to confirm system functionality.

9.1 Application Information

Applications of the TCA9548A contain an I^2C (or SMBus) master device and up to eight I^2C slave devices. The downstream channels are ideally used to resolve I^2C slave address conflicts. For example, if eight identical digital temperature sensors are needed in the application, one sensor can be connected at each channel: 0-7. When the temperature at a specific location needs to be read, the appropriate channel can be enabled and all other channels switched off, the data can be retrieved, and the I^2C master can move on and read the next channel.

In an application where the I^2C bus contains many additional slave devices that do not result in I^2C slave address conflicts, these slave devices can be connected to any desired channel to distribute the total bus capacitance across multiple channels. If multiple switches are enabled simultaneously, additional design requirements must be considered (see the *Design Requirements* section and *Detailed Design Procedure* section).

9.2 Typical Application

Figure 13 shows an application in which the TCA9548A can be used.

Copyright © 2012–2019, Texas Instruments Incorporated

Typical Application (continued)

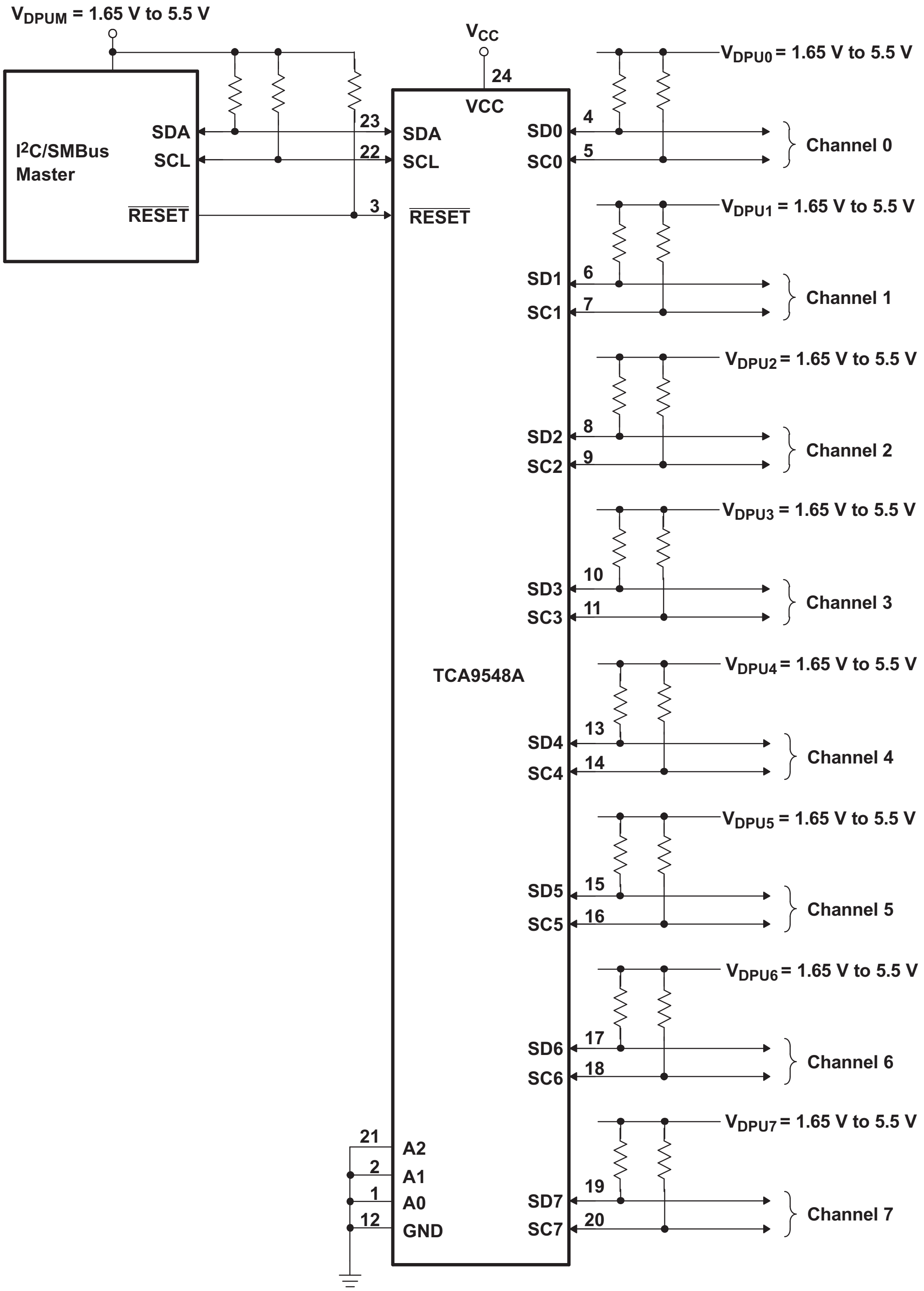

Pin numbers shown are for the PW package.

Figure 13. Typical Application Schematic

Typical Application (continued)

9.2.1 Design Requirements

A typical application of the TCA9548A contains one or more data pull-up voltages, V_{DPUX}, one for the master device (V_{DPUM}) and one for each of the selectable slave channels (V_{DPU0} – V_{DPU7}). In the event where the master device and all slave devices operate at the same voltage, then V_{DPUM} = V_{DPUX} = VCC. In an application where voltage translation is necessary, additional design requirements must be considered to determine an appropriate V_{CC} voltage.

The A0, A1, and A2 pins are hardware selectable to control the slave address of the TCA9548A. These pins may be tied directly to GND or V_{CC} in the application.

If multiple slave channels are activated simultaneously in the application, then the total I_{OL} from SCL/SDA to GND on the master side is the sum of the currents through all pull-up resistors, R_p.

The pass-gate transistors of the TCA9548A are constructed such that the V_{CC} voltage can be used to limit the maximum voltage that is passed from one I^2C bus to another.

Figure 14 shows the voltage characteristics of the pass-gate transistors (note that the graph was generated using data specified in the *Electrical Characteristics* table). In order for the TCA9548A to act as a voltage translator, the V_{pass} voltage must be equal to or lower than the lowest bus voltage. For example, if the main bus is running at 5 V and the downstream buses are 3.3 V and 2.7 V, V_{pass} must be equal to or below 2.7 V to effectively clamp the downstream bus voltages. As shown in Figure 14, $V_{pass(max)}$ is 2.7 V when the TCA9548A supply voltage is 4 V or lower, so the TCA9548A supply voltage could be set to 3.3 V. Pull-up resistors then can be used to bring the bus voltages to their appropriate levels (see Figure 13).

9.2.2 Detailed Design Procedure

Once all the slaves are assigned to the appropriate slave channels and bus voltages are identified, the pull-up resistors, R_p, for each of the buses need to be selected appropriately. The minimum pull-up resistance is a function of V_{DPUX}, $V_{OL,(max)}$, and I_{OL} as shown in Equation 1:

$$R_{p(min)} = \frac{V_{DPUX} - V_{OL(max)}}{I_{OL}} \quad (1)$$

The maximum pull-up resistance is a function of the maximum rise time, t_r (300 ns for fast-mode operation, f_{SCL} = 400 kHz) and bus capacitance, C_b as shown in Equation 2:

$$R_{p(max)} = \frac{t_r}{0.8473 \times C_b} \quad (2)$$

The maximum bus capacitance for an I^2C bus must not exceed 400 pF for fast-mode operation. The bus capacitance can be approximated by adding the capacitance of the TCA9548A, $C_{io(OFF)}$, the capacitance of wires, connections and traces, and the capacitance of each individual slave on a given channel. If multiple channels are activated simultaneously, each of the slaves on all channels contribute to total bus capacitance.

Copyright © 2012–2019, Texas Instruments Incorporated

Typical Application (continued)

9.2.3 Application Curves

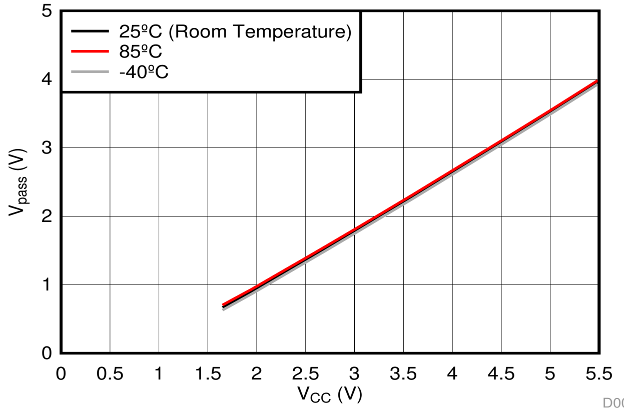

Figure 14. Pass-Gate Voltage (V_{pass}) vs Supply Voltage (V_{CC}) at Three Temperature Points

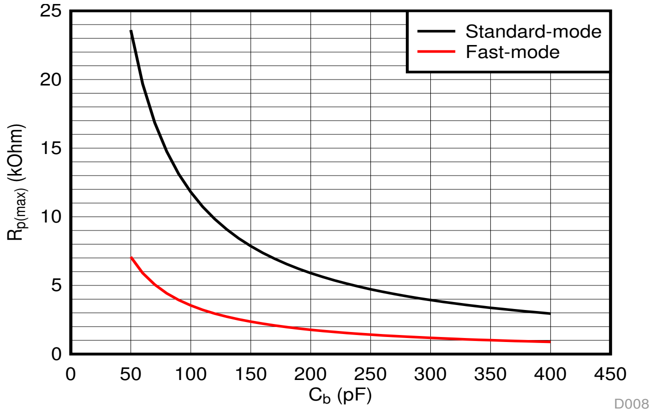

Standard-mode (f_{SCL} = 100 kHz, t_r = 1 µs)

Fast-mode (f_{SCL} = 400 kHz, t_r = 300 ns)

Figure 15. Maximum Pull-Up Resistance ($R_{p(max)}$) vs Bus Capacitance (C_b)

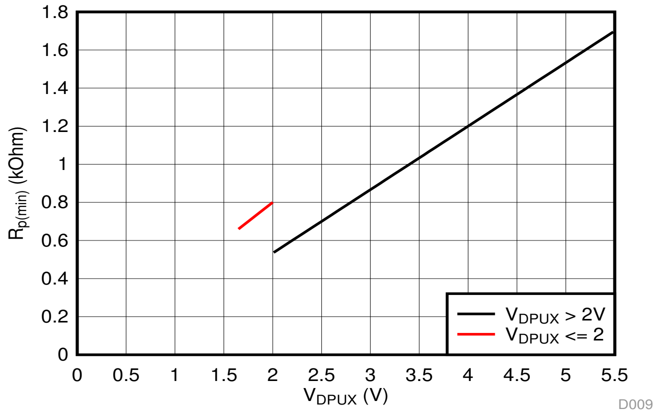

V_{OL} = 0.2*V_{DPUX}, I_{OL} = 2 mA when V_{DPUX} ≤ 2 V

V_{OL} = 0.4 V, I_{OL} = 3 mA when V_{DPUX} > 2 V

Figure 16. Minimum Pullup Resistance ($R_{p(min)}$) vs Pullup Reference Voltage (V_{DPUX})

10 Power Supply Recommendations

The operating power-supply voltage range of the TCA9548A is 1.65 V to 5.5 V applied at the VCC pin. When the TCA9548A is powered on for the first time or anytime the device must be reset by cycling the power supply, the power-on reset requirements must be followed to ensure the I^2C bus logic is initialized properly.

10.1 Power-On Reset Requirements

In the event of a glitch or data corruption, TCA9548A can be reset to its default conditions by using the power-on reset feature. Power-on reset requires that the device go through a power cycle to be completely reset. This reset also happens when the device is powered on for the first time in an application.

A power-on reset is shown in Figure 17.

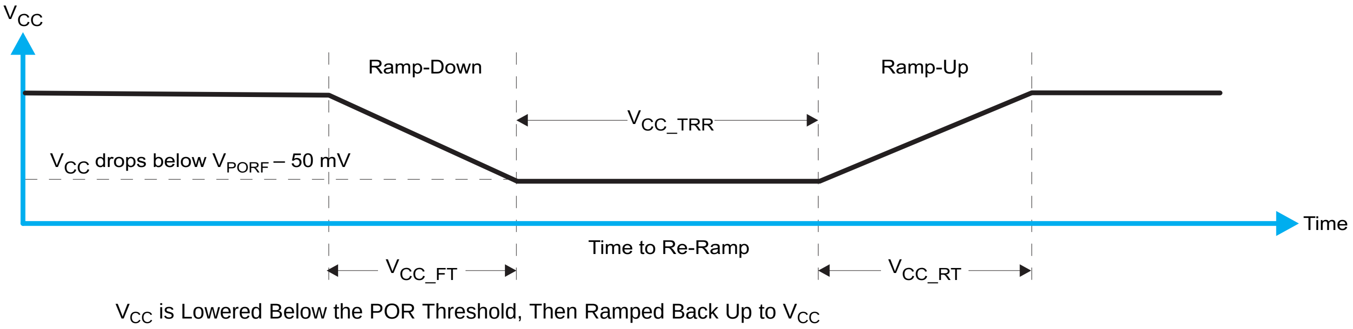

Figure 17. Power-On Reset Waveform

Table 3 specifies the performance of the power-on reset feature for TCA9548A for both types of power-on reset.

Table 3. Recommended Supply Sequencing and Ramp Rates (1)

PARAMETER			MIN	MAX	UNIT
V_{CC_FT}	Fall time	See Figure 17	1	100	ms
V_{CC_RT}	Rise time	See Figure 17	0.1	100	ms
V_{CC_TRR}	Time to re-ramp (when V_{CC} drops below $V_{PORF(min)}$ – 50 mV or when V_{CC} drops to GND)	See Figure 17	40		µs
V_{CC_GH}	Level that V_{CC} can glitch down to, but not cause a functional disruption when V_{CC_GW} = 1 µs	See Figure 18		1.2	V
V_{CC_GW}	Glitch width that does not cause a functional disruption when $V_{CC\_GH} = 0.5 \times V_{CC}$	See Figure 18		10	µs

(1) All supply sequencing and ramp rate values are measured at T_A = 25°C

Glitches in the power supply can also affect the power-on reset performance of this device. The glitch width (V_{CC_GW}) and height (V_{CC_GH}) are dependent on each other. The bypass capacitance, source impedance, and device impedance are factors that affect power-on reset performance. Figure 18 and Table 3 provide more information on how to measure these specifications.

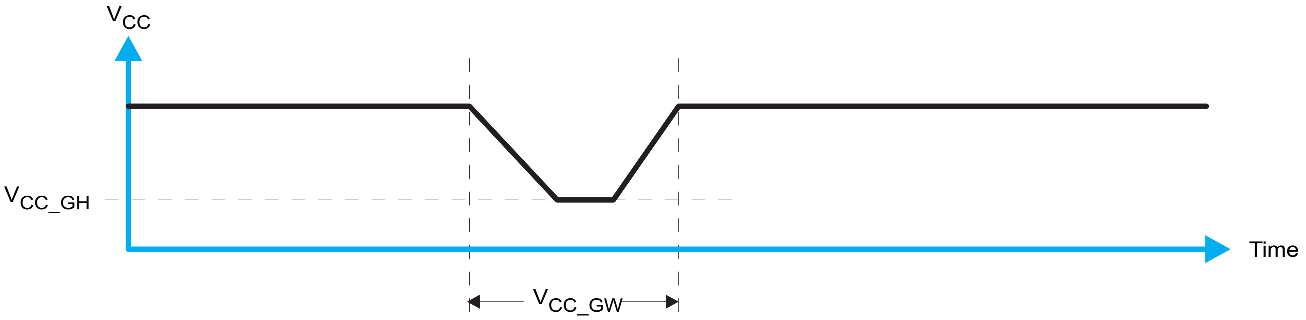

Figure 18. Glitch Width and Glitch Height

Copyright © 2012–2019, Texas Instruments Incorporated

V_{POR} is critical to the power-on reset. V_{POR} is the voltage level at which the reset condition is released and all the registers and the I^{2}C/SMBus state machine are initialized to their default states. The value of V_{POR} differs based on the V_{CC} being lowered to or from 0. Figure 19 and Table 3 provide more details on this specification.

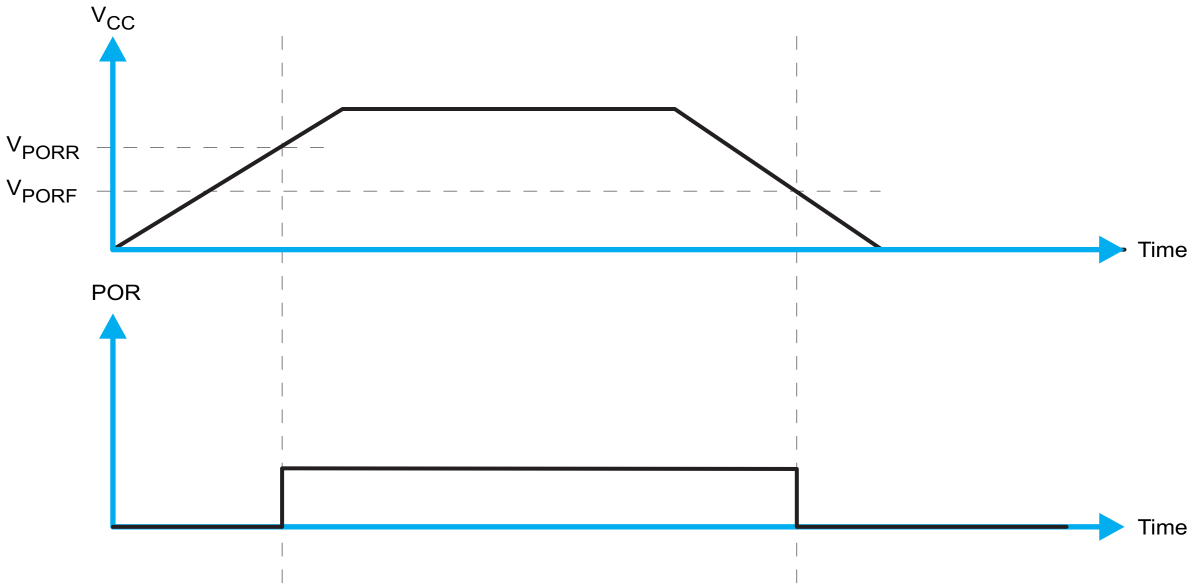

Figure 19. V_{POR}

11 Layout

11.1 Layout Guidelines

For PCB layout of the TCA9548A, common PCB layout practices must be followed but additional concerns related to high-speed data transfer such as matched impedances and differential pairs are not a concern for I^{2}C signal speeds. It is common to have a dedicated ground plane on an inner layer of the board and pins that are connected to ground must have a low-impedance path to the ground plane in the form of wide polygon pours and multiple vias. By-pass and de-coupling capacitors are commonly used to control the voltage on the VCC pin, using a larger capacitor to provide additional power in the event of a short power supply glitch and a smaller capacitor to filter out high-frequency ripple.

In an application where voltage translation is not required, all V_{DPUX} voltages and V_{CC} could be at the same potential and a single copper plane could connect all of pull-up resistors to the appropriate reference voltage. In an application where voltage translation is required, V_{DPUM} and V_{DPU0} – V_{DPU7}, may all be on the same layer of the board with split planes to isolate different voltage potentials.

To reduce the total I^{2}C bus capacitance added by PCB parasitics, data lines (SCn and SDn) must be a short as possible and the widths of the traces must also be minimized (for example, 5-10 mils depending on copper weight).

11.2 Layout Example

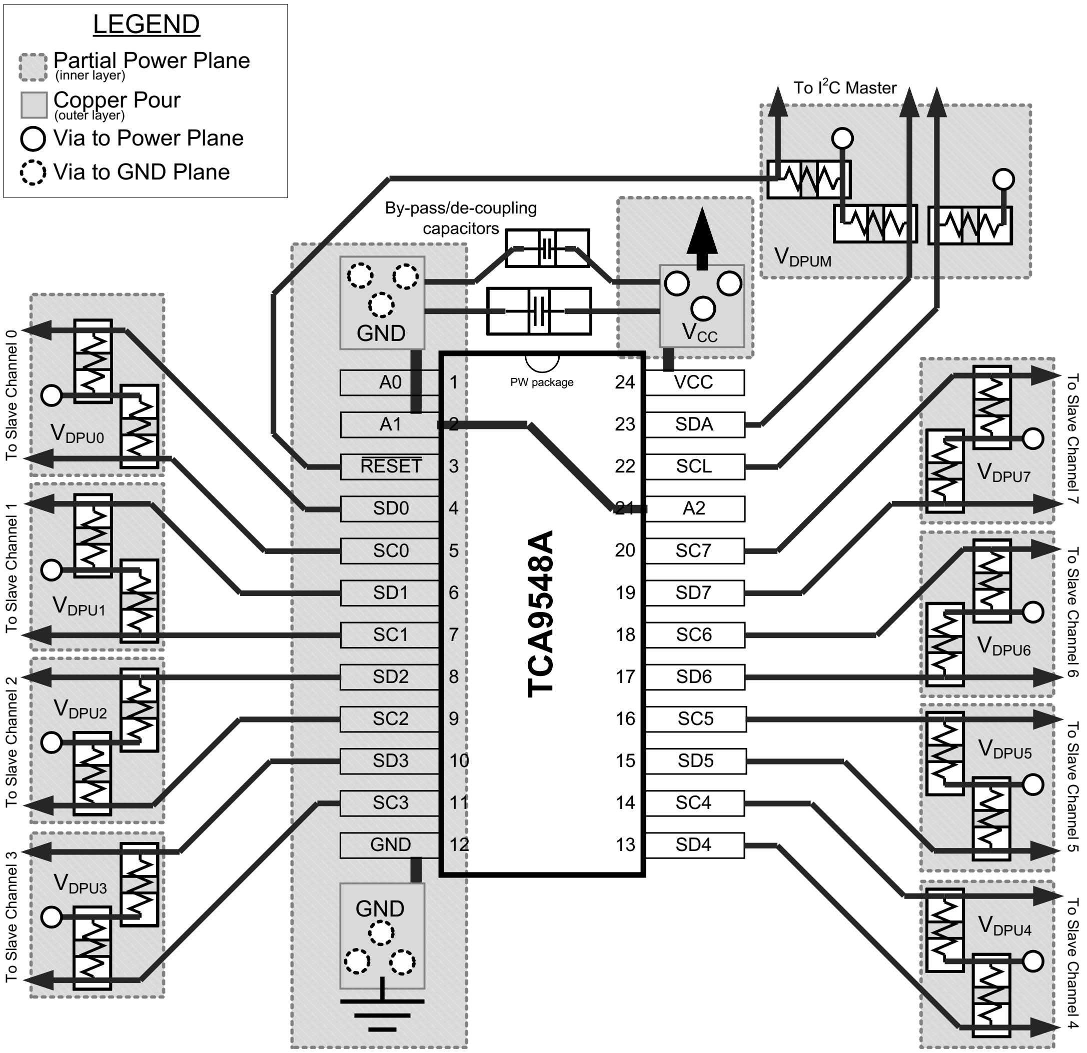

Figure 20. Layout Schematic

Copyright © 2012–2019, Texas Instruments Incorporated

12 Device and Documentation Support

12.1 Documentation Support

12.1.1 Related Documentation

For related documentation see the following:

- *I2C Bus Pull-Up Resistor Calculation*
- *Maximum Clock Frequency of I2C Bus Using Repeaters*
- *Introduction to Logic*
- *Understanding the I2C Bus*
- *Choosing the Correct I2C Device for New Designs*
- *TCA9548AEVM User's Guide*

12.2 Receiving Notification of Documentation Updates

To receive notification of documentation updates, navigate to the device product folder on ti.com. In the upper right corner, click on *Alert me* to register and receive a weekly digest of any product information that has changed. For change details, review the revision history included in any revised document.

12.3 Support Resources

TI E2E™ support forums are an engineer's go-to source for fast, verified answers and design help — straight from the experts. Search existing answers or ask your own question to get the quick design help you need.

Linked content is provided "AS IS" by the respective contributors. They do not constitute TI specifications and do not necessarily reflect TI's views; see TI's Terms of Use.

12.4 Trademarks

E2E is a trademark of Texas Instruments.
All other trademarks are the property of their respective owners.

12.5 Electrostatic Discharge Caution

This integrated circuit can be damaged by ESD. Texas Instruments recommends that all integrated circuits be handled with appropriate precautions. Failure to observe proper handling and installation procedures can cause damage.

ESD damage can range from subtle performance degradation to complete device failure. Precision integrated circuits may be more susceptible to damage because very small parametric changes could cause the device not to meet its published specifications.

12.6 Glossary

SLYZ022 — *TI Glossary*.

This glossary lists and explains terms, acronyms, and definitions.

13 Mechanical, Packaging, and Orderable Information

The following pages include mechanical, packaging, and orderable information. This information is the most current data available for the designated devices. This data is subject to change without notice and revision of this document. For browser-based versions of this data sheet, refer to the left-hand navigation.

PACKAGING INFORMATION

Orderable Device	Status (1)	Package Type	Package Drawing	Pins	Package Qty	Eco Plan (2)	Lead finish/ Ball material (6)	MSL Peak Temp (3)	Op Temp (°C)	Device Marking (4/5)	Samples
TCA9548AMRGER	ACTIVE	VQFN	RGE	24	3000	RoHS & Green	NIPDAU	Level-2-260C-1 YEAR	-40 to 85	PW548A	Samples
TCA9548APWR	ACTIVE	TSSOP	PW	24	2000	RoHS & Green	NIPDAU	Level-1-260C-UNLIM	-40 to 85	PW548A	Samples
TCA9548ARGER	ACTIVE	VQFN	RGE	24	3000	RoHS & Green	NIPDAU	Level-2-260C-1 YEAR	-40 to 85	PW548A	Samples

(1) The marketing status values are defined as follows:
ACTIVE: Product device recommended for new designs.
LIFEBUY: TI has announced that the device will be discontinued, and a lifetime-buy period is in effect.
NRND: Not recommended for new designs. Device is in production to support existing customers, but TI does not recommend using this part in a new design.
PREVIEW: Device has been announced but is not in production. Samples may or may not be available.
OBSOLETE: TI has discontinued the production of the device.

(2) **RoHS:** TI defines "RoHS" to mean semiconductor products that are compliant with the current EU RoHS requirements for all 10 RoHS substances, including the requirement that RoHS substance do not exceed 0.1% by weight in homogeneous materials. Where designed to be soldered at high temperatures, "RoHS" products are suitable for use in specified lead-free processes. TI may reference these types of products as "Pb-Free".
RoHS Exempt: TI defines "RoHS Exempt" to mean products that contain lead but are compliant with EU RoHS pursuant to a specific EU RoHS exemption.
Green: TI defines "Green" to mean the content of Chlorine (Cl) and Bromine (Br) based flame retardants meet JS709B low halogen requirements of <=1000ppm threshold. Antimony trioxide based flame retardants must also meet the <=1000ppm threshold requirement.

(3) MSL, Peak Temp. - The Moisture Sensitivity Level rating according to the JEDEC industry standard classifications, and peak solder temperature.

(4) There may be additional marking, which relates to the logo, the lot trace code information, or the environmental category on the device.

(5) Multiple Device Markings will be inside parentheses. Only one Device Marking contained in parentheses and separated by a "~" will appear on a device. If a line is indented then it is a continuation of the previous line and the two combined represent the entire Device Marking for that device.

(6) Lead finish/Ball material - Orderable Devices may have multiple material finish options. Finish options are separated by a vertical ruled line. Lead finish/Ball material values may wrap to two lines if the finish value exceeds the maximum column width.

Important Information and Disclaimer: The information provided on this page represents TI's knowledge and belief as of the date that it is provided. TI bases its knowledge and belief on information provided by third parties, and makes no representation or warranty as to the accuracy of such information. Efforts are underway to better integrate information from third parties. TI has taken and continues to take reasonable steps to provide representative and accurate information but may not have conducted destructive testing or chemical analysis on incoming materials and chemicals. TI and TI suppliers consider certain information to be proprietary, and thus CAS numbers and other limited information may not be available for release.

In no event shall TI's liability arising out of such information exceed the total purchase price of the TI part(s) at issue in this document sold by TI to Customer on an annual basis.

OTHER QUALIFIED VERSIONS OF TCA9548A :

- Automotive: TCA9548A-Q1

NOTE: Qualified Version Definitions:

- Automotive - Q100 devices qualified for high-reliability automotive applications targeting zero defects

TAPE AND REEL INFORMATION

REEL DIMENSIONS

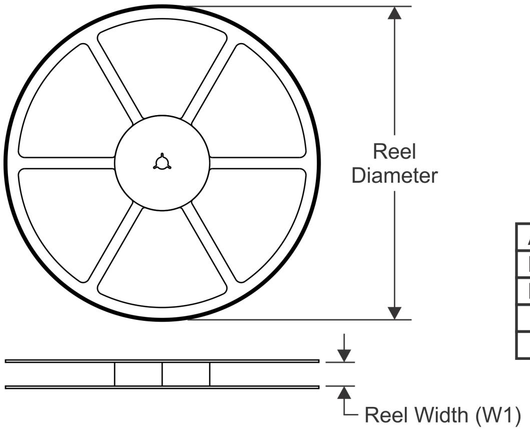

TAPE DIMENSIONS

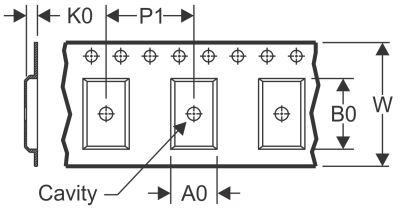

A0	Dimension designed to accommodate the component width
B0	Dimension designed to accommodate the component length
K0	Dimension designed to accommodate the component thickness
W	Overall width of the carrier tape
P1	Pitch between successive cavity centers

QUADRANT ASSIGNMENTS FOR PIN 1 ORIENTATION IN TAPE

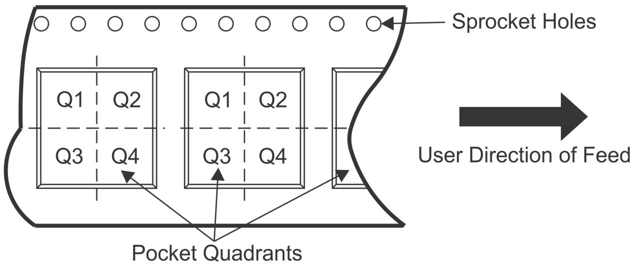

*All dimensions are nominal

Device	Package Type	Package Drawing	Pins	SPQ	Reel Diameter (mm)	Reel Width W1 (mm)	A0 (mm)	B0 (mm)	K0 (mm)	P1 (mm)	W (mm)	Pin1 Quadrant
TCA9548AMRGER	VQFN	RGE	24	3000	330.0	12.4	4.25	4.25	1.15	8.0	12.0	Q1
TCA9548APWR	TSSOP	PW	24	2000	330.0	16.4	6.95	8.3	1.6	8.0	16.0	Q1
TCA9548ARGER	VQFN	RGE	24	3000	330.0	12.4	4.25	4.25	1.15	8.0	12.0	Q2

TAPE AND REEL BOX DIMENSIONS

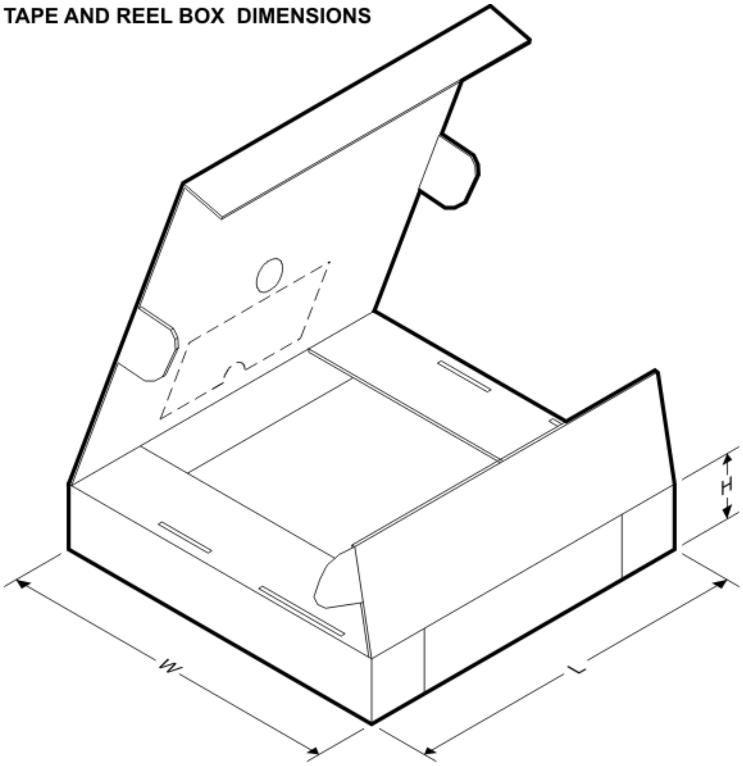

*All dimensions are nominal

Device	Package Type	Package Drawing	Pins	SPQ	Length (mm)	Width (mm)	Height (mm)
TCA9548AMRGER	VQFN	RGE	24	3000	853.0	449.0	35.0
TCA9548APWR	TSSOP	PW	24	2000	853.0	449.0	35.0
TCA9548ARGER	VQFN	RGE	24	3000	853.0	449.0	35.0

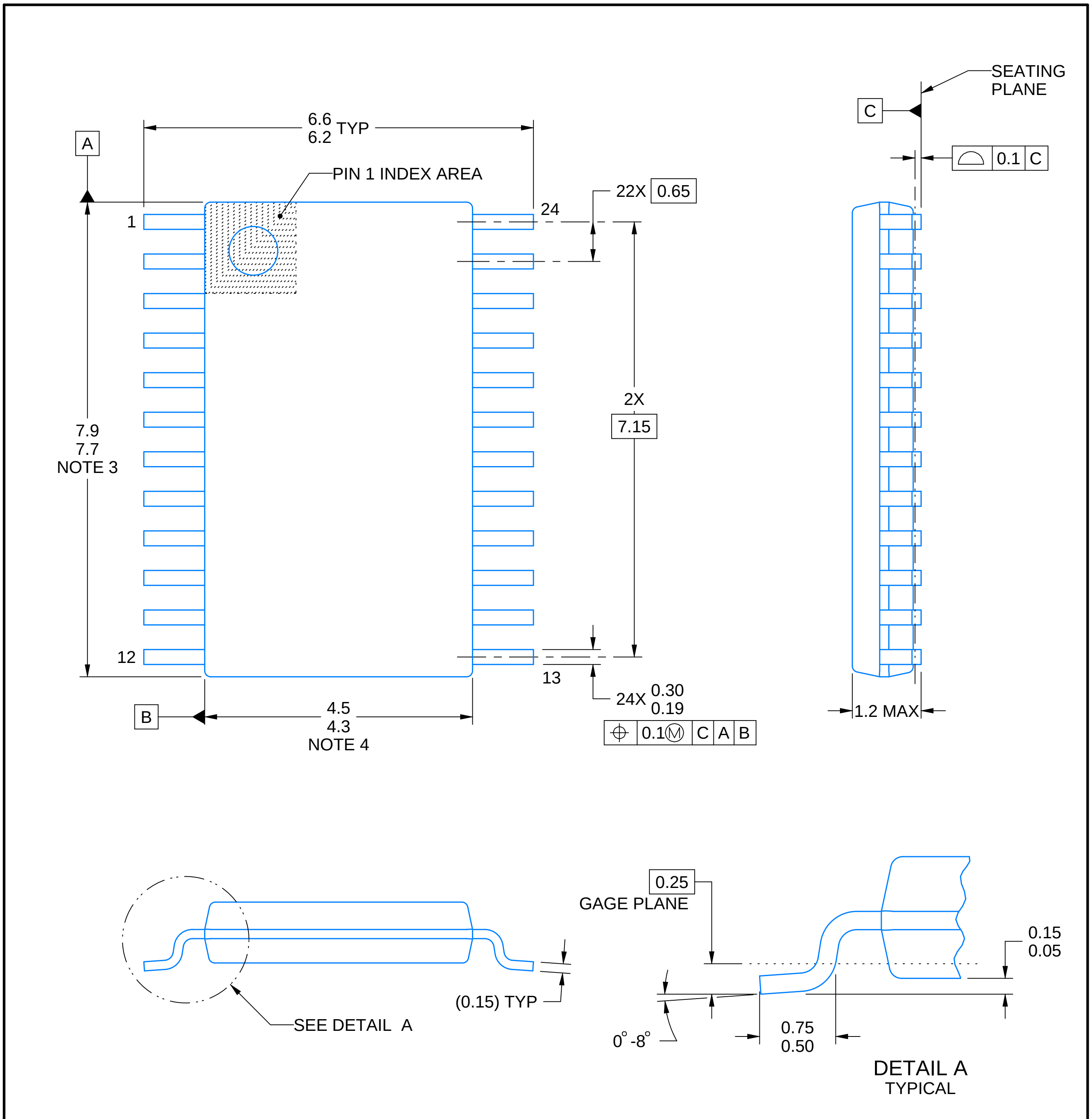

4220208/A 02/2017

NOTES:

1. All linear dimensions are in millimeters. Any dimensions in parenthesis are for reference only. Dimensioning and tolerancing per ASME Y14.5M.
2. This drawing is subject to change without notice.
3. This dimension does not include mold flash, protrusions, or gate burrs. Mold flash, protrusions, or gate burrs shall not exceed 0.15 mm per side.
4. This dimension does not include interlead flash. Interlead flash shall not exceed 0.25 mm per side.
5. Reference JEDEC registration MO-153.

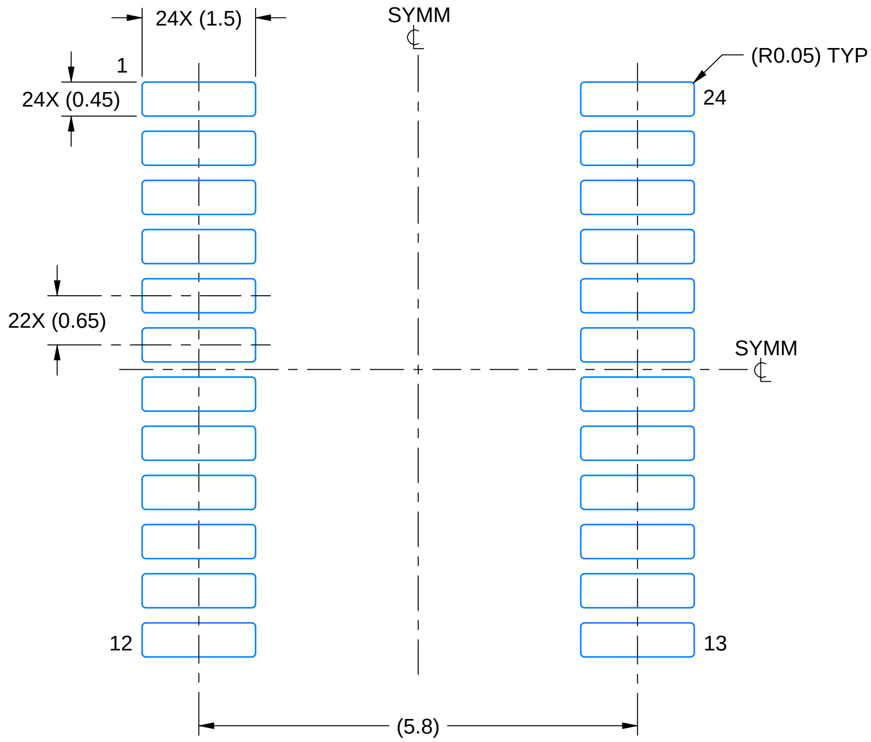

LAND PATTERN EXAMPLE
EXPOSED METAL SHOWN
SCALE: 10X

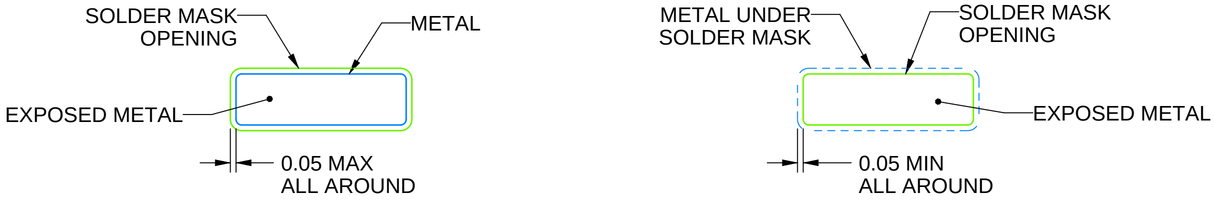

SOLDER MASK DETAILS

4220208/A 02/2017

NOTES: (continued)

6. Publication IPC-7351 may have alternate designs.
7. Solder mask tolerances between and around signal pads can vary based on board fabrication site.

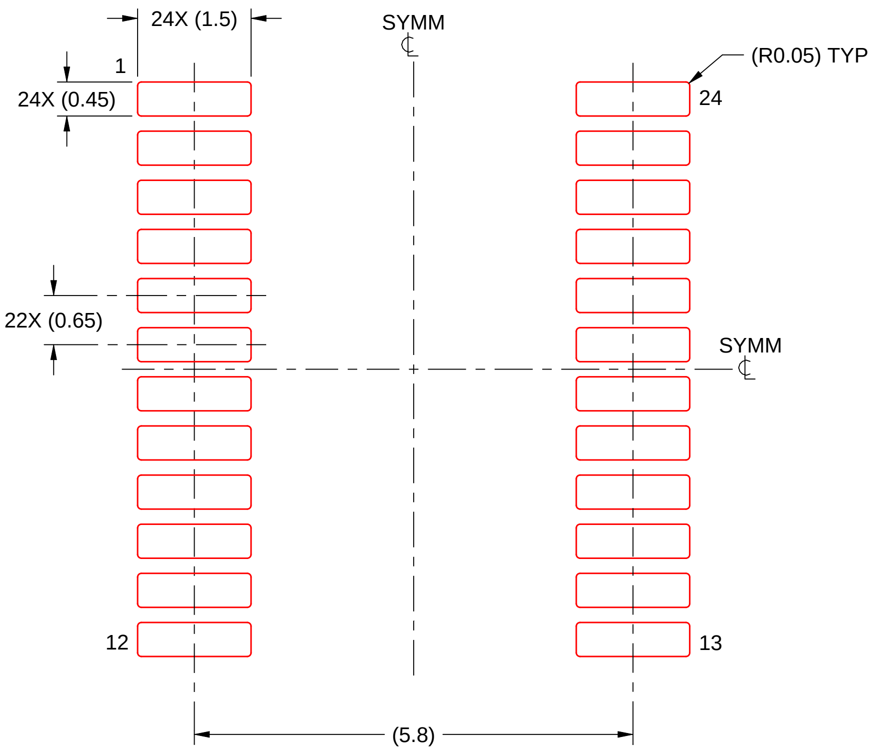

SOLDER PASTE EXAMPLE
BASED ON 0.125 mm THICK STENCIL
SCALE: 10X

4220208/A 02/2017

NOTES: (continued)

8. Laser cutting apertures with trapezoidal walls and rounded corners may offer better paste release. IPC-7525 may have alternate design recommendations.
9. Board assembly site may have different recommendations for stencil design.

RGE 24 VQFN - 1 mm max height

PLASTIC QUAD FLATPACK - NO LEAD

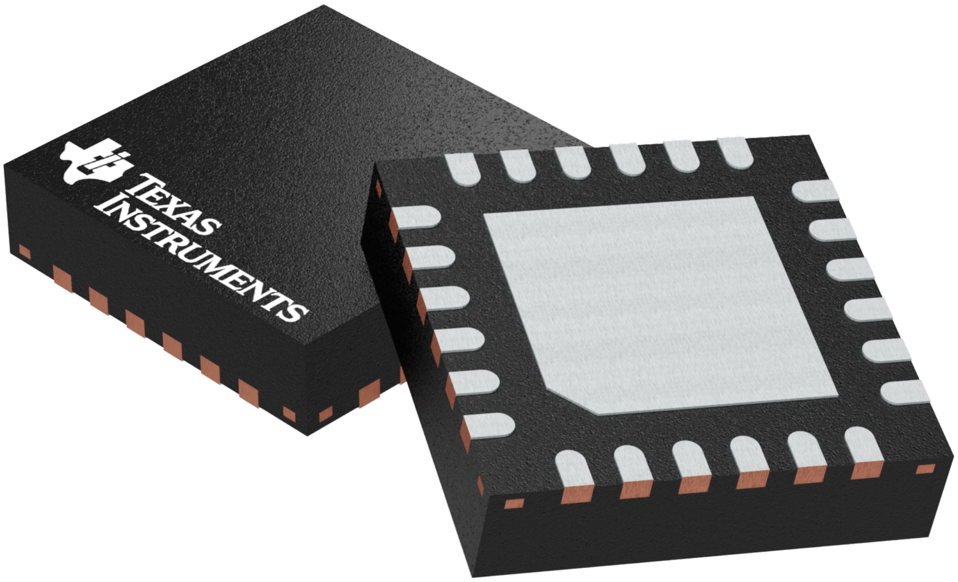

Images above are just a representation of the package family, actual package may vary.
Refer to the product data sheet for package details.

4204104/H

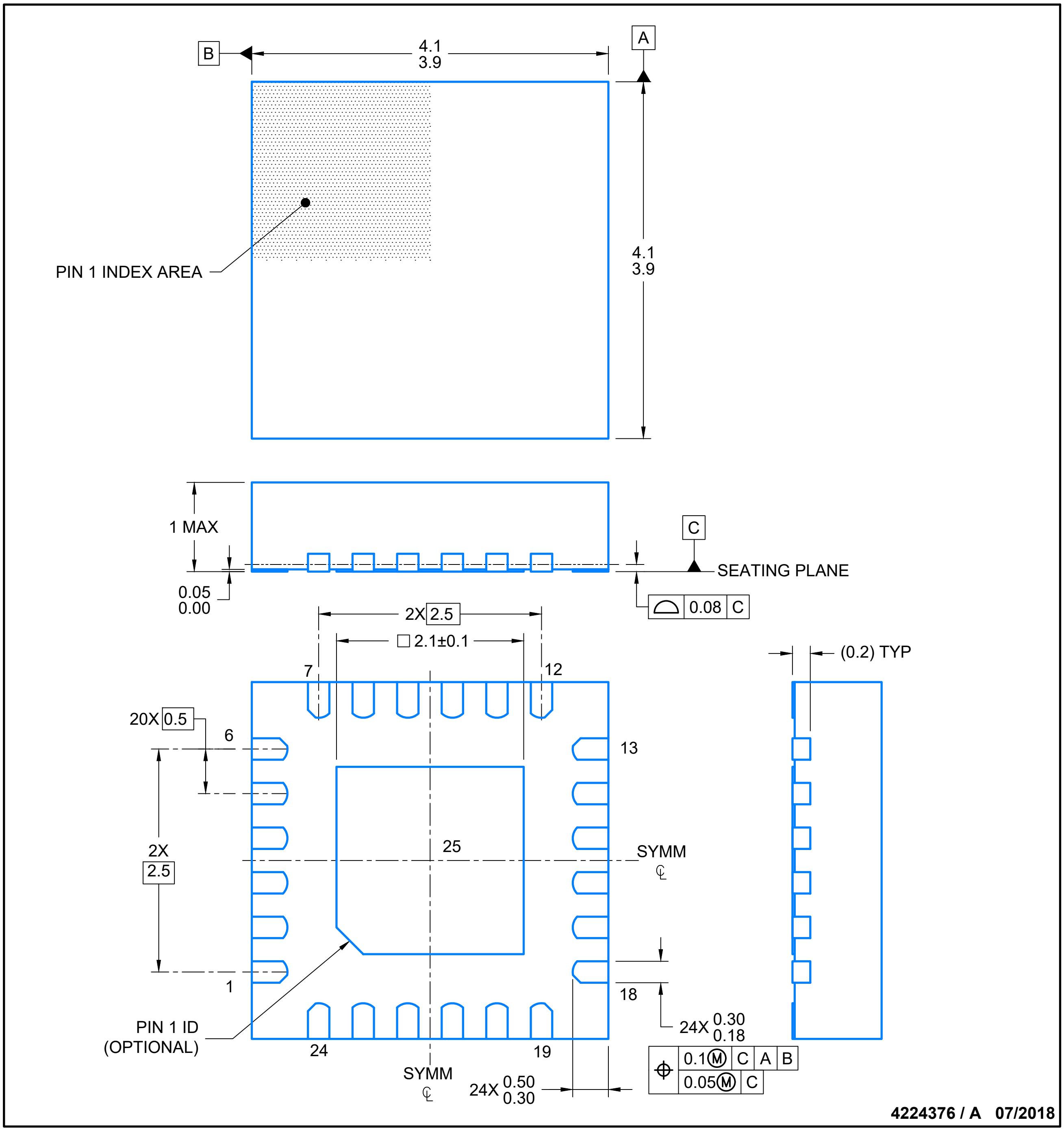

NOTES:

1. All linear dimensions are in millimeters. Any dimensions in parenthesis are for reference only. Dimensioning and tolerancing per ASME Y14.5M.
2. This drawing is subject to change without notice.
3. The package thermal pad must be soldered to the printed circuit board for thermal and mechanical performance.

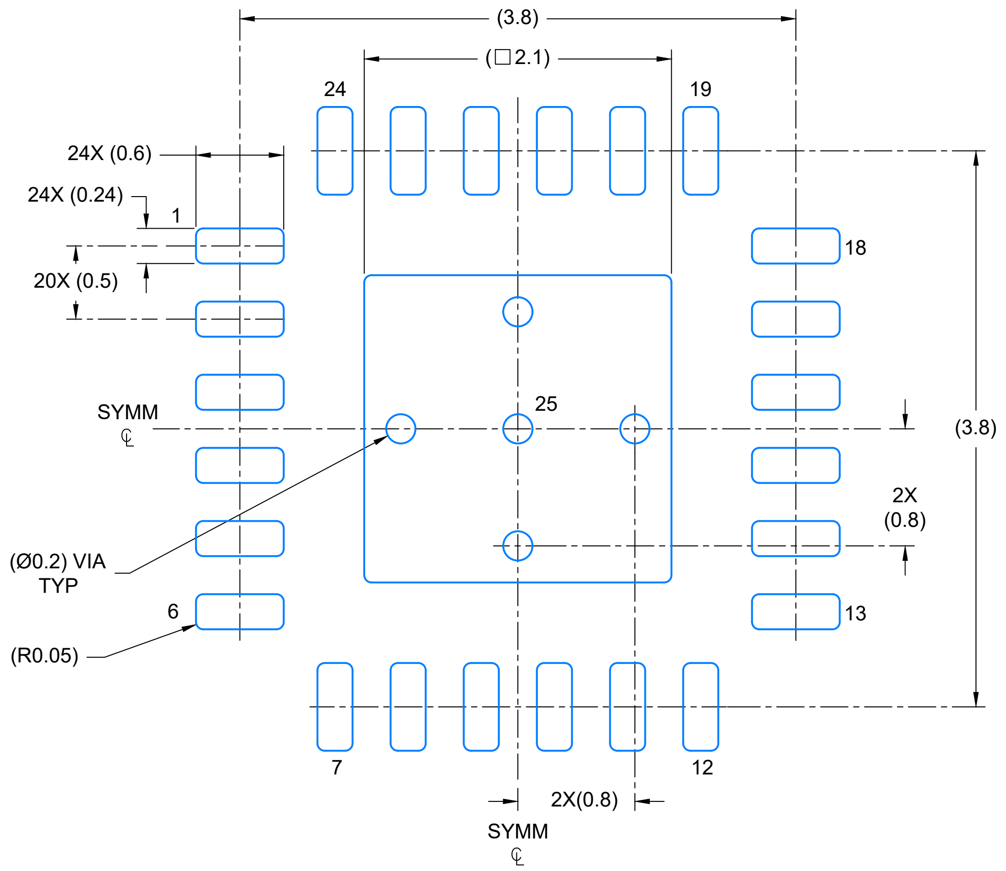

LAND PATTERN EXAMPLE
SCALE: 20X

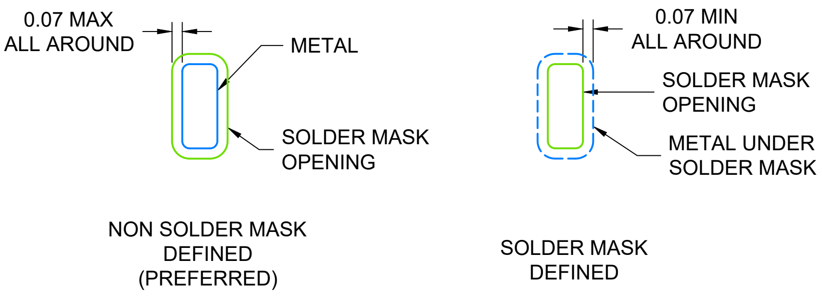

SOLDER MASK DETAILS

4224376 / A 07/2018

NOTES: (continued)

4. This package is designed to be soldered to a thermal pad on the board. For more information, see Texas Instruments literature number SLUA271 (www.ti.com/lit/slua271).
5. Solder mask tolerances between and around signal pads can vary based on board fabrication site.

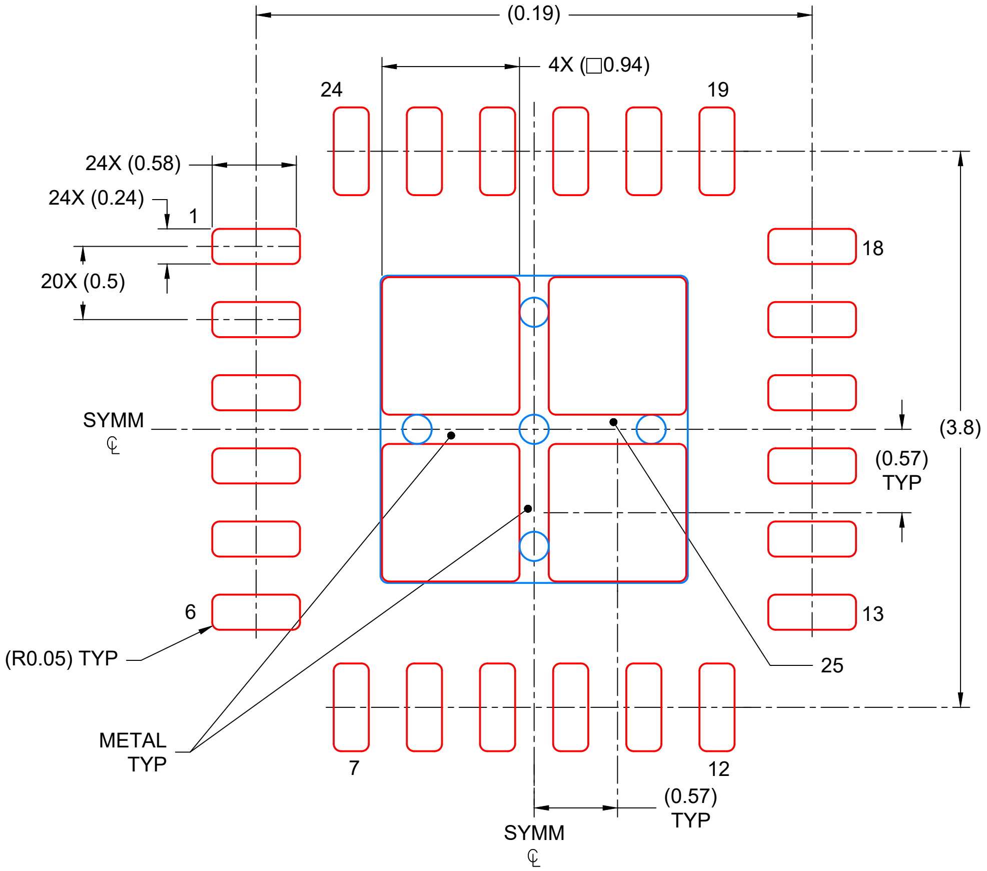

SOLDER PASTE EXAMPLE
BASED ON 0.125 mm THICK STENCIL

EXPOSED PAD
80% PRINTED COVERAGE BY AREA
SCALE: 20X

4224376 / A 07/2018

NOTES: (continued)

6. Laser cutting apertures with trapezoidal walls and rounded corners may offer better paste release. IPC-7525 may have alternate design recommendations..

IMPORTANT NOTICE AND DISCLAIMER

TI PROVIDES TECHNICAL AND RELIABILITY DATA (INCLUDING DATASHEETS), DESIGN RESOURCES (INCLUDING REFERENCE DESIGNS), APPLICATION OR OTHER DESIGN ADVICE, WEB TOOLS, SAFETY INFORMATION, AND OTHER RESOURCES "AS IS" AND WITH ALL FAULTS, AND DISCLAIMS ALL WARRANTIES, EXPRESS AND IMPLIED, INCLUDING WITHOUT LIMITATION ANY IMPLIED WARRANTIES OF MERCHANTABILITY, FITNESS FOR A PARTICULAR PURPOSE OR NON-INFRINGEMENT OF THIRD PARTY INTELLECTUAL PROPERTY RIGHTS.

These resources are intended for skilled developers designing with TI products. You are solely responsible for (1) selecting the appropriate TI products for your application, (2) designing, validating and testing your application, and (3) ensuring your application meets applicable standards, and any other safety, security, or other requirements. These resources are subject to change without notice. TI grants you permission to use these resources only for development of an application that uses the TI products described in the resource. Other reproduction and display of these resources is prohibited. No license is granted to any other TI intellectual property right or to any third party intellectual property right. TI disclaims responsibility for, and you will fully indemnify TI and its representatives against, any claims, damages, costs, losses, and liabilities arising out of your use of these resources.

TI's products are provided subject to TI's Terms of Sale (www.ti.com/legal/termsofsale.html) or other applicable terms available either on ti.com or provided in conjunction with such TI products. TI's provision of these resources does not expand or otherwise alter TI's applicable warranties or warranty disclaimers for TI products.

Mailing Address: Texas Instruments, Post Office Box 655303, Dallas, Texas 75265
Copyright © 2020, Texas Instruments Incorporated
