# Supplementary material for: The Unfolding Space Glove: A Wearable Spatio-Visual to Haptic Sensory Substitution Device for Blind People
Source: Sensors (Basel). 2022 Feb 26;22(5):1859. doi: 10.3390/s22051859 (PMC8914703; doi:10.3390/s22051859)
Supplement: Supplementary file 1 [file sensors-22-01859-s001.zip › S1_GitHub-Release_unfolding-space-hardware-0.2.1-revised.zip!/unfolding-space-hardware-0.2.1-revised/pcb/datasheets/ujc-hp-3-smt-tr.pdf]

CUI DEVICES

MODEL: UJC-HP-3-SMT-TR │ DESCRIPTION: USB RECEPTACLE

FEATURES
- USB Type C receptacle
- mates with standard USB Type C plugs
- designed for power only applications
- 6-pin
- reflow solder compatible
- tape & reel packaging

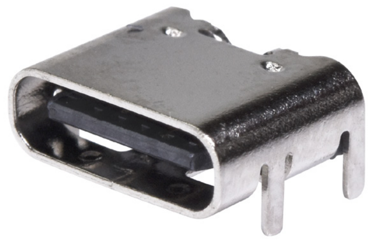

RoHS

SPECIFICATIONS

parameter	conditions/description	min	typ	max	units
rated input voltage				20	Vdc
rated input current	power/GND pins collectively (A9, A12, B9, B12)<br>power/GND pins individually (A9, A12, B9, B12)<br>all other pins			3<br>1.5<br>0.25	A<br>A<br>A
contact resistance[1]				30	mΩ
insulation resistance	at 100 Vdc for 1 minute between adjacent contacts	100			MΩ
voltage withstand	for 1 minute between adjacent contacts			100	Vac
insertion force	at a rate of 30 cycles/min	5		20	N
withdrawal force	at a rate of 30 cycles/min	8		20	N
life	at a rate of 500 max cycles per hour		10,000		cycles
operating temperature		-40		80	°C
flammability rating	UL94V-0				
RoHS	yes				

Note: 1. When measured at 20 mV / 100 mA max.

SOLDERABILITY

parameter	conditions/description	min	typ	max	units
reel storage	at relative humidity <60%			30	°C
reflow soldering[2]	see reflow profile			260	°C

Note: 2. It is recommended to reflow solder within 168 hours from opening vacuum packaging at a temperature <30°C & relative humidity <60%.

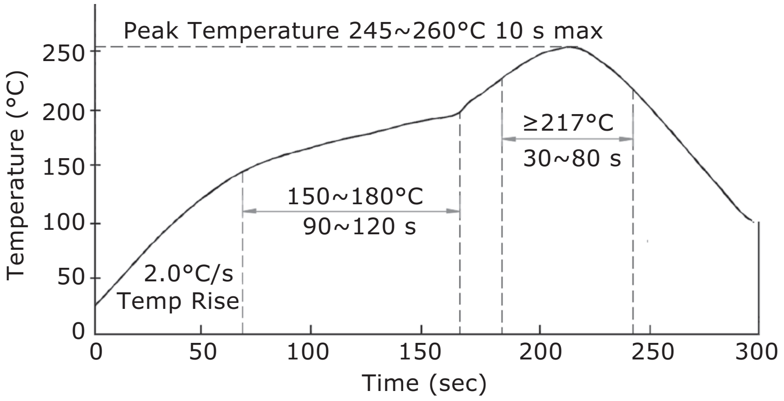

MECHANICAL DRAWING

units: mm
tolerance:
X.X ±0.30 mm
X.XX ±0.20 mm
X.XXX ±0.10 mm
PCB: ±0.05 mm
PCB thickness: 0.8 mm

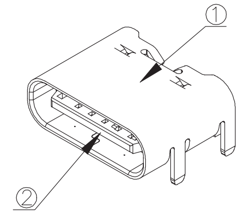

		MATERIAL	PLATING
1	shell	stainless steel	nickel
2	insulator	nylon 46 (UL94V-0)	black
3	contact A	copper alloy	contact: gold<br>solder: nickel
4	contact B	copper alloy	contact: gold<br>solder: nickel
5	mid plate	stainless steel	

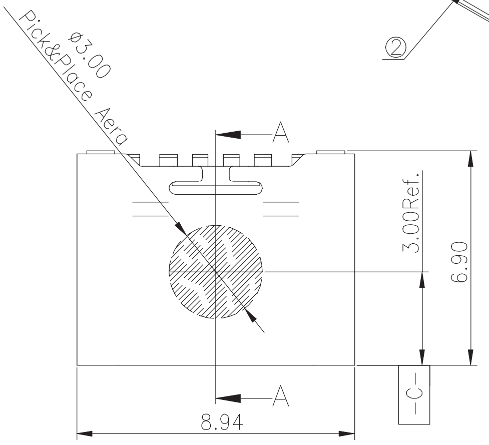

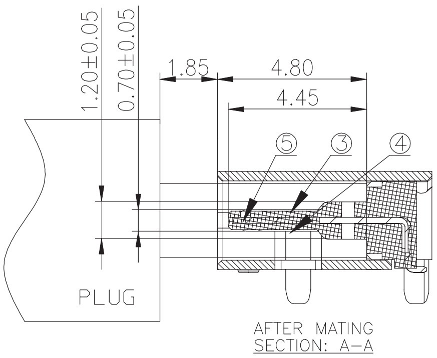

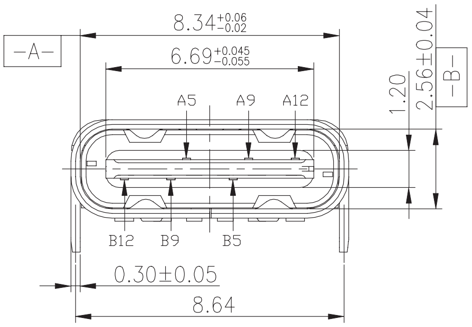

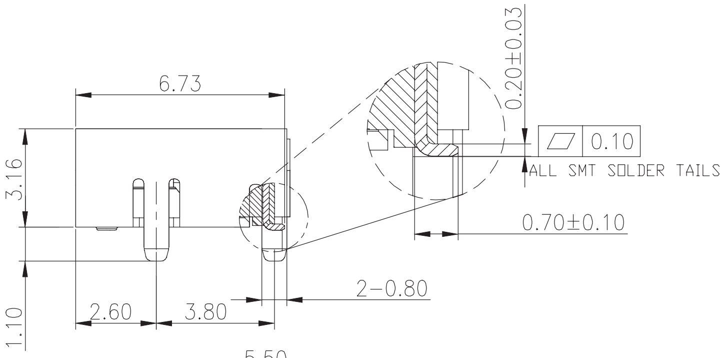

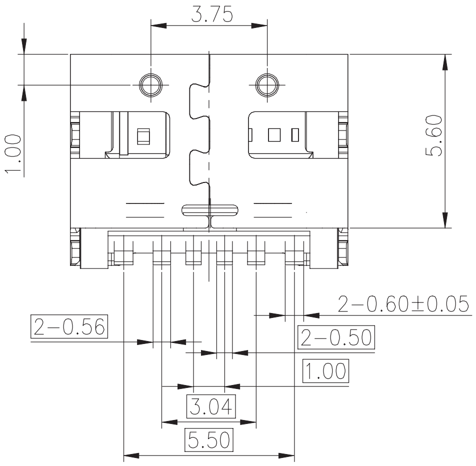

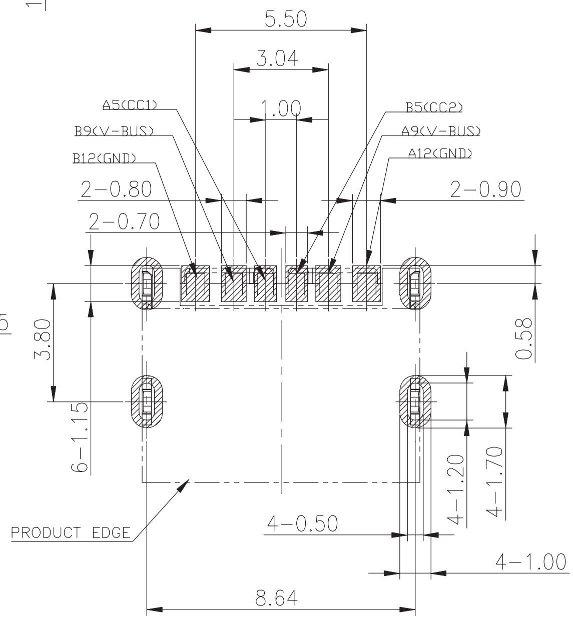

Recommended PCB Layout
Top View

PACKAGING

units: mm

Reel Size: Ø330 mm
Reel QTY: 1,000 pcs per reel

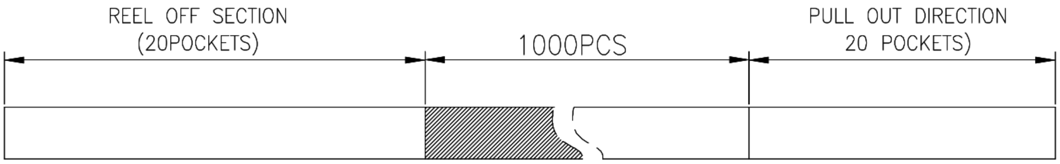

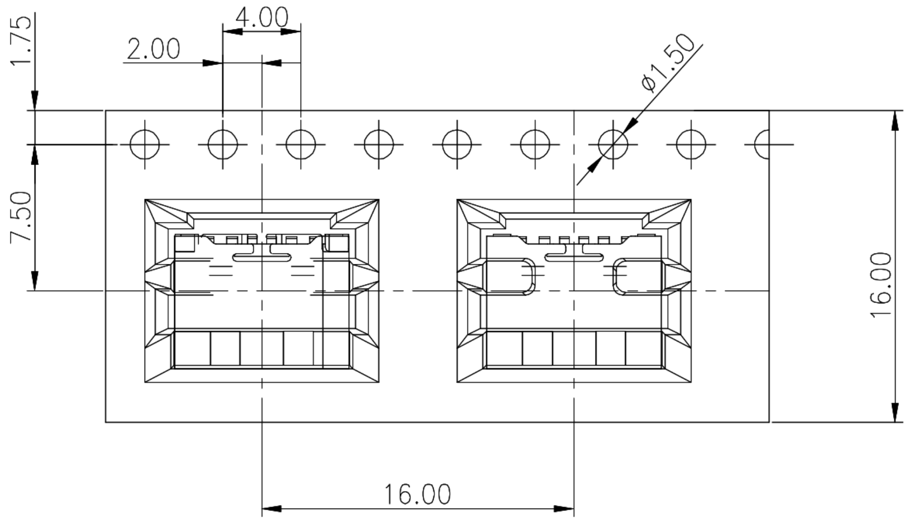

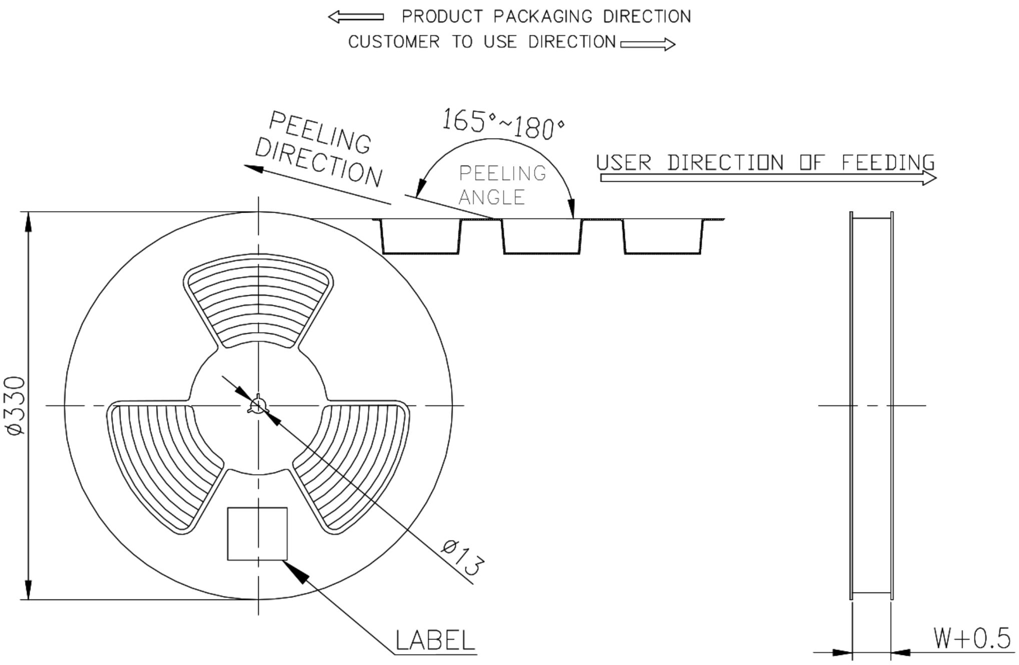

REVISION HISTORY

rev.	description	date
1.0	initial release	04/30/2020

The revision history provided is for informational purposes only and is believed to be accurate.

CUI DEVICES

CUI Devices offers a one (1) year limited warranty. Complete warranty information is listed on our website.

CUI Devices reserves the right to make changes to the product at any time without notice. Information provided by CUI Devices is believed to be accurate and reliable. However, no responsibility is assumed by CUI Devices for its use, nor for any infringements of patents or other rights of third parties which may result from its use.

CUI Devices products are not authorized or warranted for use as critical components in equipment that requires an extremely high level of reliability. A critical component is any component of a life support device or system whose failure to perform can be reasonably expected to cause the failure of the life support device or system, or to affect its safety or effectiveness.

cuidevices.com
