# Supplementary material for: The Unfolding Space Glove: A Wearable Spatio-Visual to Haptic Sensory Substitution Device for Blind People
Source: Sensors (Basel). 2022 Feb 26;22(5):1859. doi: 10.3390/s22051859 (PMC8914703; doi:10.3390/s22051859)
Supplement: Supplementary file 1 [file sensors-22-01859-s001.zip › S1_GitHub-Release_unfolding-space-hardware-0.2.1-revised.zip!/unfolding-space-hardware-0.2.1-revised/pcb/datasheets/usblc6-2.pdf]

Very low capacitance ESD protection

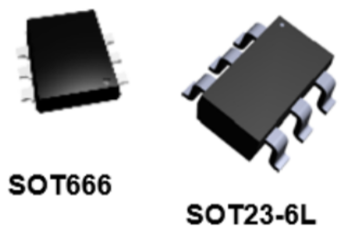

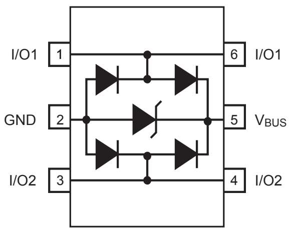

Functional diagram (top view)

Product status link
USBLC6-2

Features

- 2 data-line protection
- Protects VBUS
- Very low capacitance: 3.5 pF max.
- Very low leakage current: 150 nA max.
- SOT-666 and SOT23-6L packages
- RoHS compliant

Benefits

- Very low capacitance between lines to GND for optimized data integrity and speed
- Low PCB space consumption: 2.9 mm² max for SOT-666 and 9 mm² max for SOT23-6L
- Enhanced ESD protection: IEC 61000-4-2 level 4 compliance guaranteed at device level, hence greater immunity at system level
- ESD protection of VBUS
- High reliability offered by monolithic integration
- Low leakage current for longer operation of battery powered devices
- Fast response time
- Consistent D+ / D- signal balance:
  - Very low capacitance matching tolerance I/O to GND = 0.015 pF
  - Compliant with USB 2.0 requirements

Complies with the following standards:

- IEC 61000-4-2 level 4:
  - 15 kV (air discharge)
  - 8 kV (contact discharge)

Applications

- USB 2.0 ports up to 480 Mb/s (high speed)
- Compatible with USB 1.1 low and full speed
- Ethernet port: 10/100 Mb/s
- SIM card protection
- Video line protection
- Portable electronics

Description

The USBLC6-2SC6 and USBLC6-2P6 are monolithic application specific devices dedicated to ESD protection of high speed interfaces, such as USB 2.0, Ethernet links and video lines.

The very low line capacitance secures a high level of signal integrity without compromising in protecting sensitive chips against the most stringently characterized ESD strikes.

1 Characteristics

Table 1. Absolute ratings (T_{amb} = 25 °C)

Symbol	Parameter		Value	Unit
V_{PP}	Peak pulse voltage	IEC 61000-4-2 level 4 standard:<br>Air discharge<br>Contact discharge<br>MIL STD883G-Method 3015-7	 15<br>15<br>25	kV
T_{stg}	Storage temperature range		-55 to +150	°C
T_j	Operating junction temperature range		-40 to +125	°C
T_L	Maximum lead temperature for soldering during 10 s at 5 mm		260	°C

Table 2. Electrical characteristics (T_{amb} = 25 °C)

Symbol	Parameter	Test conditions	Value			Unit
			Min.	Typ.	Max.	
I_{RM}	Leakage current	V_{RM} = 5.25 V		10	150	nA
V_{BR}	Breakdown voltage between V_{BUS} and GND	I_R = 1 mA	6			V
V_F	Forward voltage	I_F = 10 mA			1.1	V
V_{CL}	Clamping voltage	I_{PP} = 1 A, 8/20 µs<br>Any I/O pin to GND			12	V
		I_{PP} = 5 A, 8/20 µs<br>Any I/O pin to GND			17	
$C_{i/o\text{-}GND}$	Capacitance between I/O and GND	V_R = 1.65 V		2.5	3.5	pF
$\Delta C_{i/o\text{-}GND}$				0.015		
$C_{i/o\text{-}i/o}$	Capacitance between I/O	V_R = 1.65 V		1.2	1.7	pF
$\Delta C_{i/o\text{-}i/o}$				0.04		

1.1 Characteristics (curves)

Figure 1. Capacitance versus voltage (typical values)

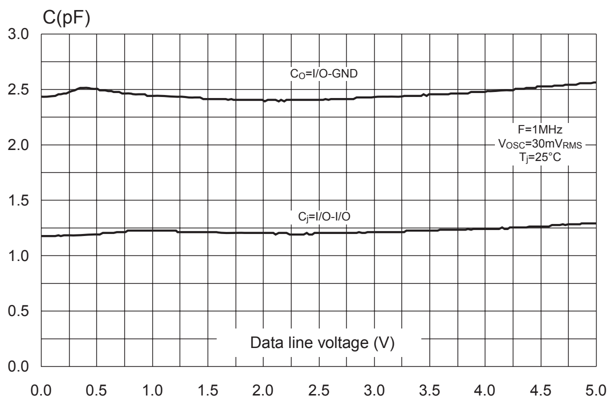

Figure 2. Line capacitance versus frequency (typical values)

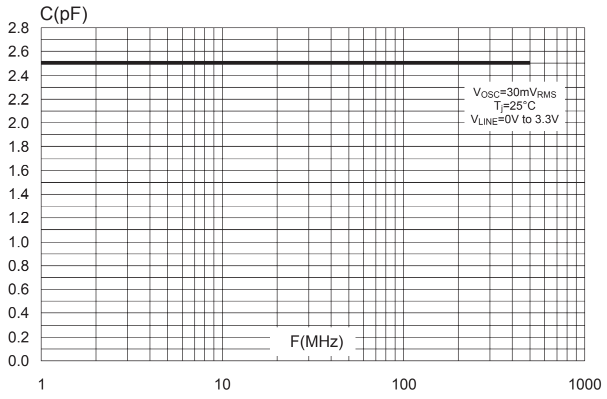

Figure 3. Relative variation of leakage current versus junction temperature (typical values)

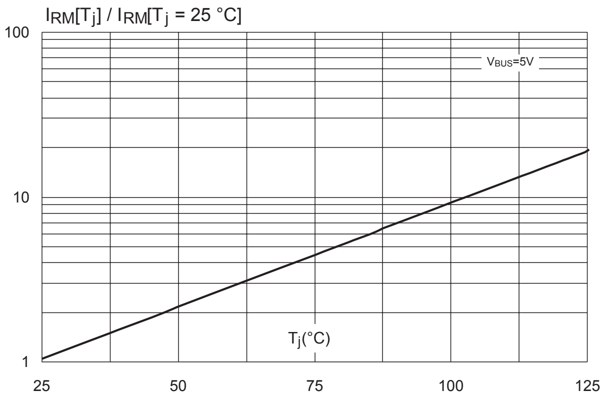

Figure 4. Frequency response

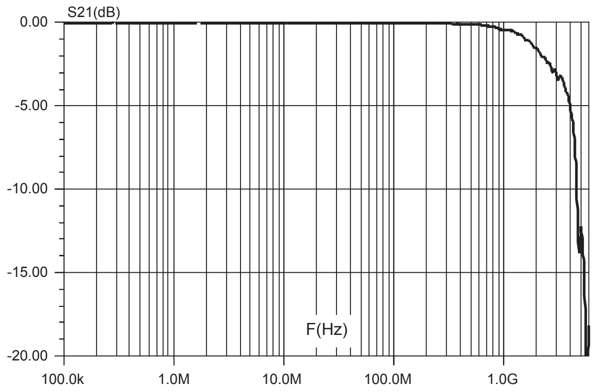

2 Technical information

2.1 Surge protection

The USBLC6-2 is particularly optimized to perform surge protection based on the rail to rail topology.

The clamping voltage V_{CL} can be calculated as follow:

$V_{CL}+ = V_{TRANSIL} + V_F$ for positive surges

$V_{CL}- = - V_F$ for negative surges

with: $V_F = V_T + R_d.Ip$

(V_F forward drop voltage) / (V_T forward drop threshold voltage)

and $V_{TRANSIL} = V_{BR} + R_{d\_TRANSIL}.I_P$

Calculation example

We assume that the value of the dynamic resistance of the clamping diode is typically:

$R_d = 0.5\ \Omega$ and $V_T = 1.1$ V

We assume that the value of the dynamic resistance of the transil diode is typically:

$R_{d\_TRANSIL} = 0.5\ \Omega$ and $V_{BR} = 6.1$ V For an IEC 61000-4-2 surge level 4 (Contact Discharge: $V_g = 8$ kV, $R_g = 330$ Ω), $V_{BUS} = +5$ V, and if in first approximation, we assume that:

$I_p = V_g / R_g = 24$ A.

So, we find:

$V_{CL}+ = +31.2$ V

$V_{CL}- = -13$ V

Note: The calculations do not take into account phenomena due to parasitic inductances.

2.2 Surge protection application example

If we consider that the connections from the pin V_{BUS} to V_{CC}, from I/O to data line and from GND to PCB GND plane are done by tracks of 10 mm long and 0.5 mm large, we assume that the parasitic inductances L_{VBUS}, $L_{I/O}$ and L_{GND} of these tracks are about 6 nH. So when an IEC 61000-4-2 surge occurs on data line, due to the rise time of this spike ($t_r = 1$ ns), the voltage V_{CL} has an extra value equal to $L_{I/O}.dI/dt + L_{GND}.dI/dt$.

The dI/dt is calculated as:

$dI/dt = I_p/t_r = 24$ A/ns

The overvoltage due to the parasitic inductances is:

$L_{I/O}.dI/dt = L_{GND}.dI/dt = 6$ nH x 24 A/ns = 144 V

By taking into account the effect of these parasitic inductances due to unsuitable layout, the clamping voltage will be:

$V_{CL}+ = +31.2 + 144 + 144 = 319.2$ V

$V_{CL}- = -13.1 - 144 - 144 = -301.1$ V

We can significantly reduce this phenomena with simple layout optimization. It is for this reason that some recommendations have to be followed (see).

Figure 5. ESD behavior: parasitic phenomena due to unsuitable layout

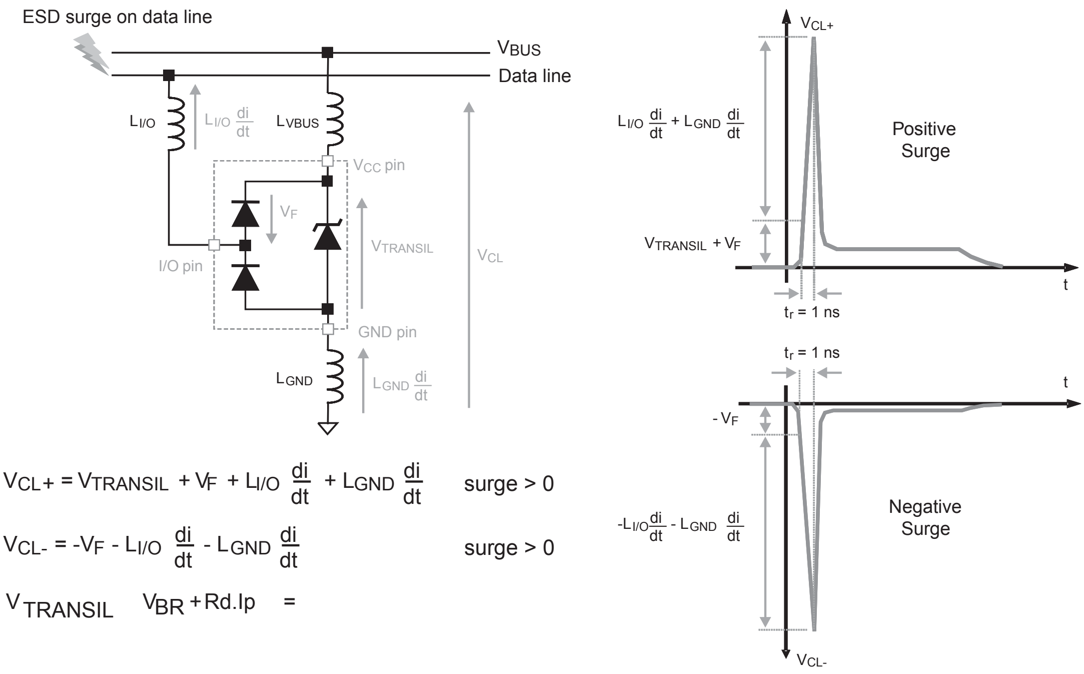

2.3 How to ensure good ESD protection

While the USBLC6-2 provides high immunity to ESD surge, efficient protection depends on the layout of the board. In the same way, with the rail to rail topology, the track from data lines to I/O pins, from VCC to VBUS pin and from GND plane to GND pin must be as short as possible to avoid overvoltages due to parasitic phenomena (see Figure 6. ESD behavior: layout optimization and Figure 5. ESD behavior: parasitic phenomena due to unsuitable layout for layout consideration).

Figure 6. ESD behavior: layout optimization

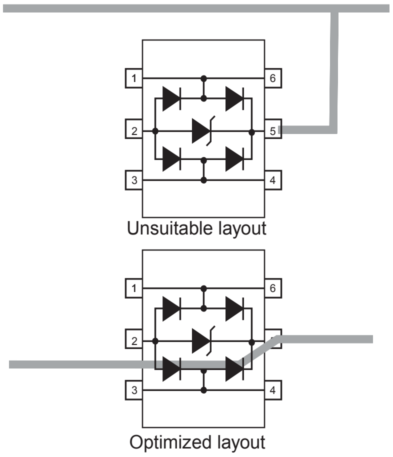

Figure 7. ESD behavior: measurement conditions

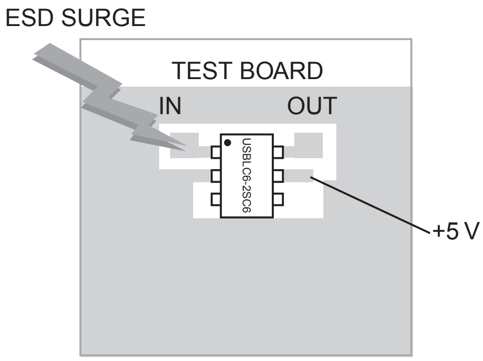

Figure 8. ESD response to IEC 61000-4-2 (+15 kV air discharge)

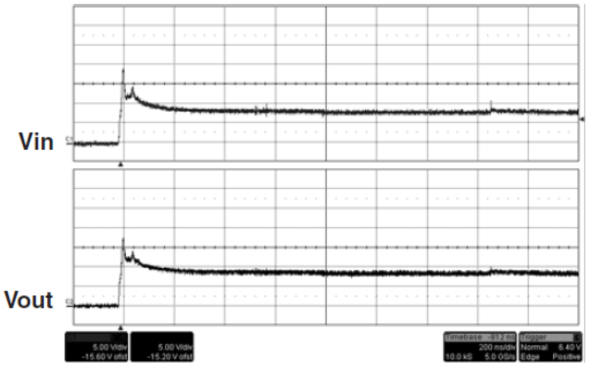

Figure 9. ESD response to IEC 61000-4-2 (-15 kV air discharge)

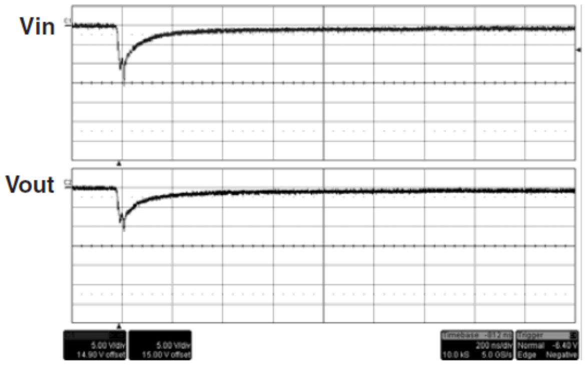

Note: ***Important***: *A good precaution to take is to put the protection device as close as possible to the disturbance source (generally the connector).*

2.4 Crosstalk behavior

2.4.1 Crosstalk phenomenon

Figure 10. **Crosstalk phenomenon**

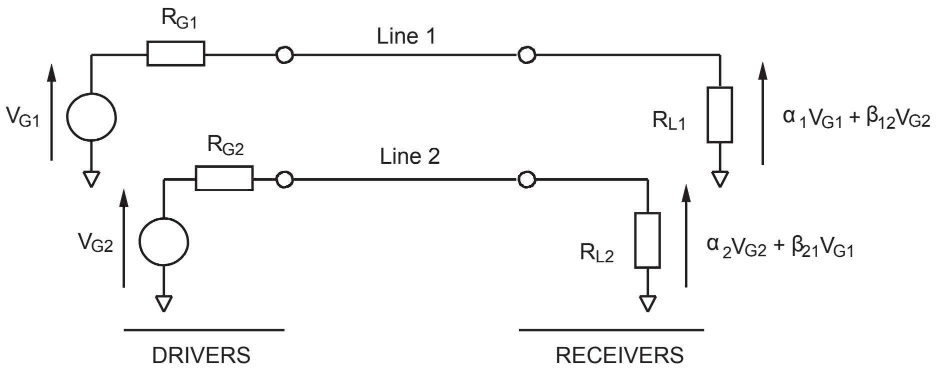

The crosstalk phenomenon is due to the coupling between 2 lines. The coupling factor (β12 or β21) increases when the gap across lines decreases, particularly in silicon dice. In the above example the expected signal on load R_{L2} is $\alpha_2 V_{G2}$, in fact the real voltage at this point has got an extra value $\beta_{21} V_{G1}$. This part of the V_{G1} signal represents the effect of the crosstalk phenomenon of the line 1 on the line 2. This phenomenon has to be taken into account when the drivers impose fast digital data or high frequency analog signals in the disturbing line. The perturbed line will be more affected if it works with low voltage signal or high load impedance (few kΩ).

Figure 11. **Analog crosstalk measurements**

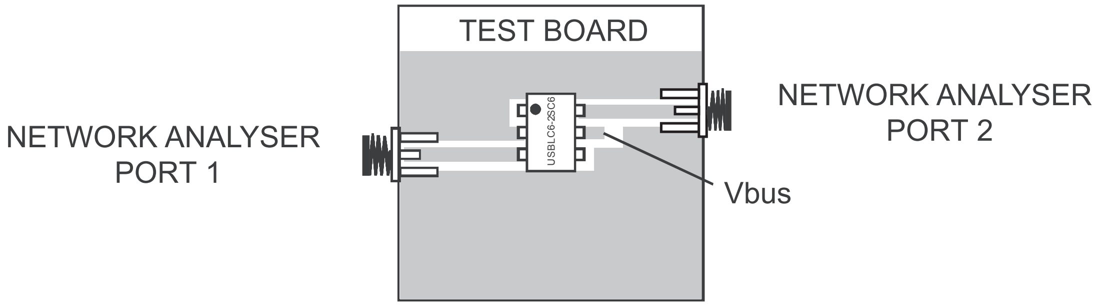

Figure 11. Analog crosstalk measurements shows the measurement circuit for the analog application. In usual frequency range of analog signals (up to 240 MHz) the effect on disturbed line is less than -55 dB (see Figure 12. Analog crosstalk results).

Figure 12. **Analog crosstalk results**

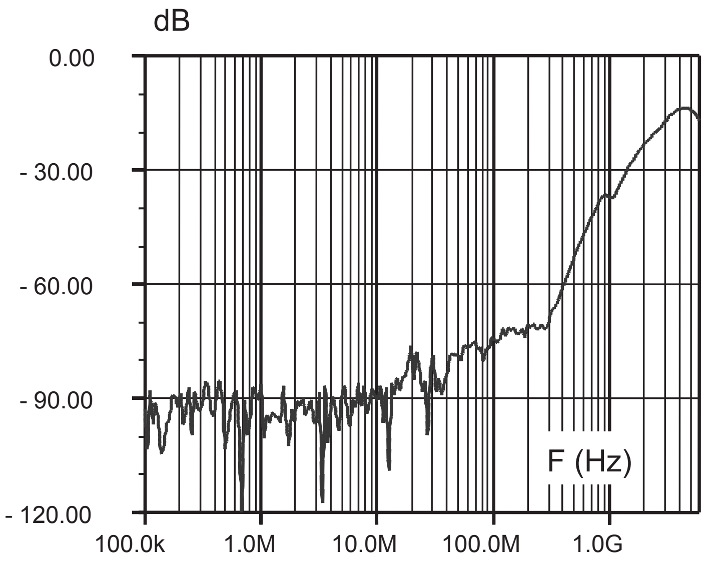

As the USBLC6-2 is designed to protect high speed data lines, it must ensure a good transmission of operating signals. The frequency response (Figure 4. Frequency response) gives attenuation information and shows that the USBLC6-2 is well suitable for data line transmission up to 480 Mbit/s while it works as a filter for undesirable signals like GSM (900 MHz) frequencies, for instance.

2.5 Application examples

Figure 13. USB 2.0 port application diagram using USBLC6-2

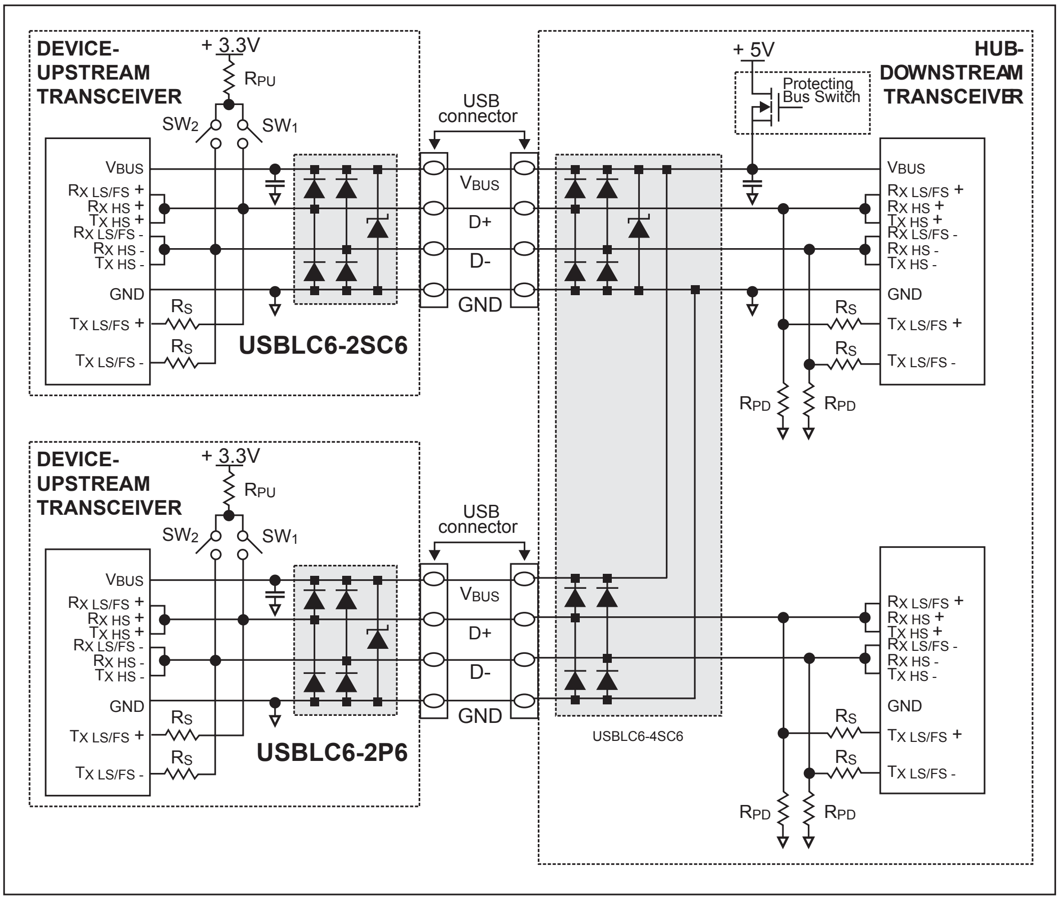

Mode	SW_1	SW_2
Low Speed LS	Open	Closed
Full Speed FS	Closed	Open
High Speed HS	Closed then open	Open

Figure 14. T1/E1/Ethernet protection

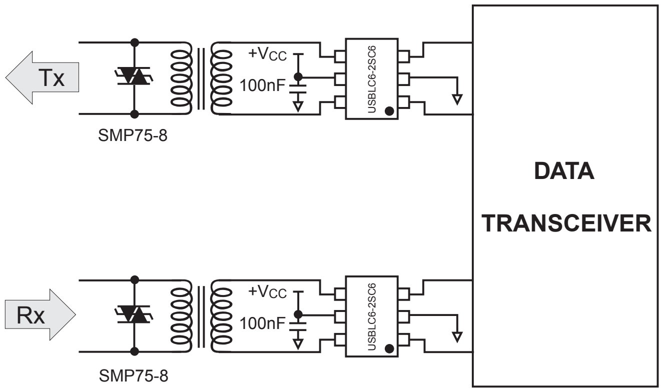

2.6 PSpice model

Figure 15. PSpice model shows the PSpice model of one USBLC6-2 cell. In this model, the diodes are defined by the PSpice parameters given in Figure 16. PSpice parameters.

Figure 15. PSpice model

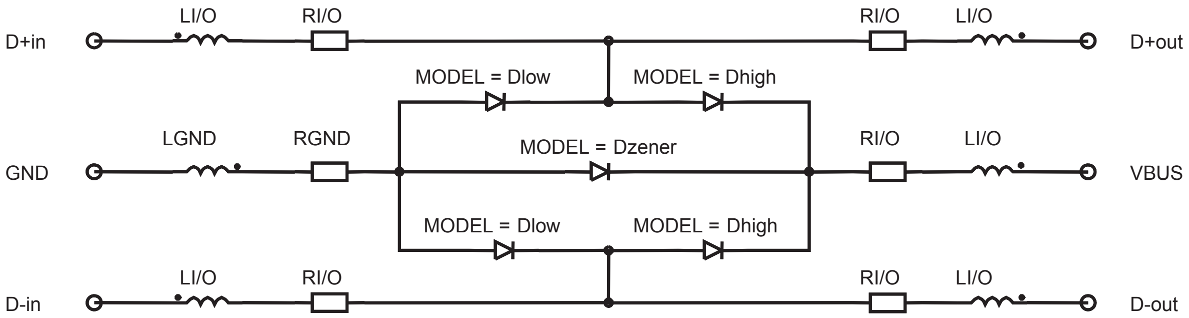

Note: This simulation model is available only for an ambient temperature of 27 °C.

Figure 16. PSpice parameters

	Dlow	Dhigh	Dzener
BV	50	50	7.3
CJ0	0.9p	2.0p	40p
IBV	1m	1m	1m
M	0.3333	0.3333	0.3333
RS	0.2	0.52	0.84
VJ	0.6	0.6	0.6
TT	0.1u	0.1u	0.1u

LI/O	750p
RI/O	110m
LGND	550p
RGND	60m

Figure 17. USBLC6-2 PCB layout considerations

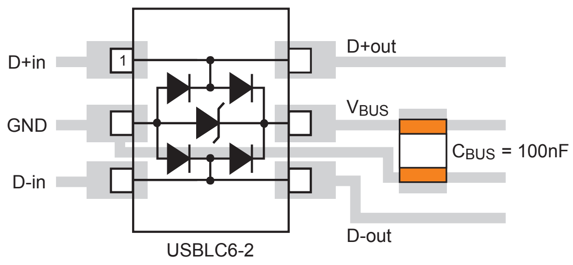

3 Package information

In order to meet environmental requirements, ST offers these devices in different grades of ECOPACK packages, depending on their level of environmental compliance. ECOPACK specifications, grade definitions and product status are available at: www.st.com. ECOPACK is an ST trademark.

3.1 SOT23-6L package information

Figure 18. **SOT23-6L package outline**

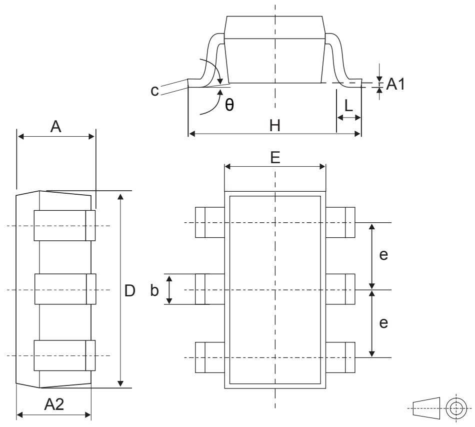

Table 3. **SOT23-6L package mechanical data**

Ref.	Dimensions					
	Millimeters			Inches(1)		
	Min.	Typ.	Max.	Min.	Typ.	Max.
A	0.9		1.45	0.0354		0.0571
A1	0		0.15	0		0.0059
A2	0.9		1.3	0.0354		0.0512
b	0.30		0.5	0.0118		0.0197
c	0.09		0.2	0.0035		0.0079
D	2.8		3.05	0.1102		0.1201
E	1.5		1.75	0.0591		0.0689
e		0.95			0.0374	
H	2.6		3	0.1024		0.1181
L	0.3		0.6	0.0118		0.0236
θ	0		10	0		0.3937

1. *Value in inches are converted from mm and rounded to 4 decimal digits*

Figure 19. Footprint recommendations, dimensions in mm (inches)

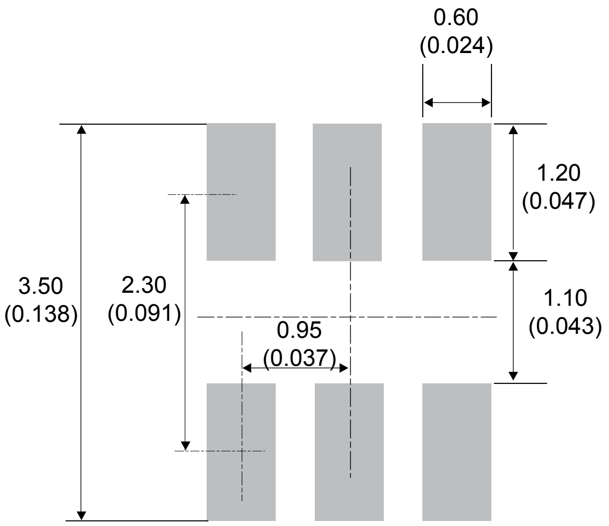

3.2 SOT-666 package information

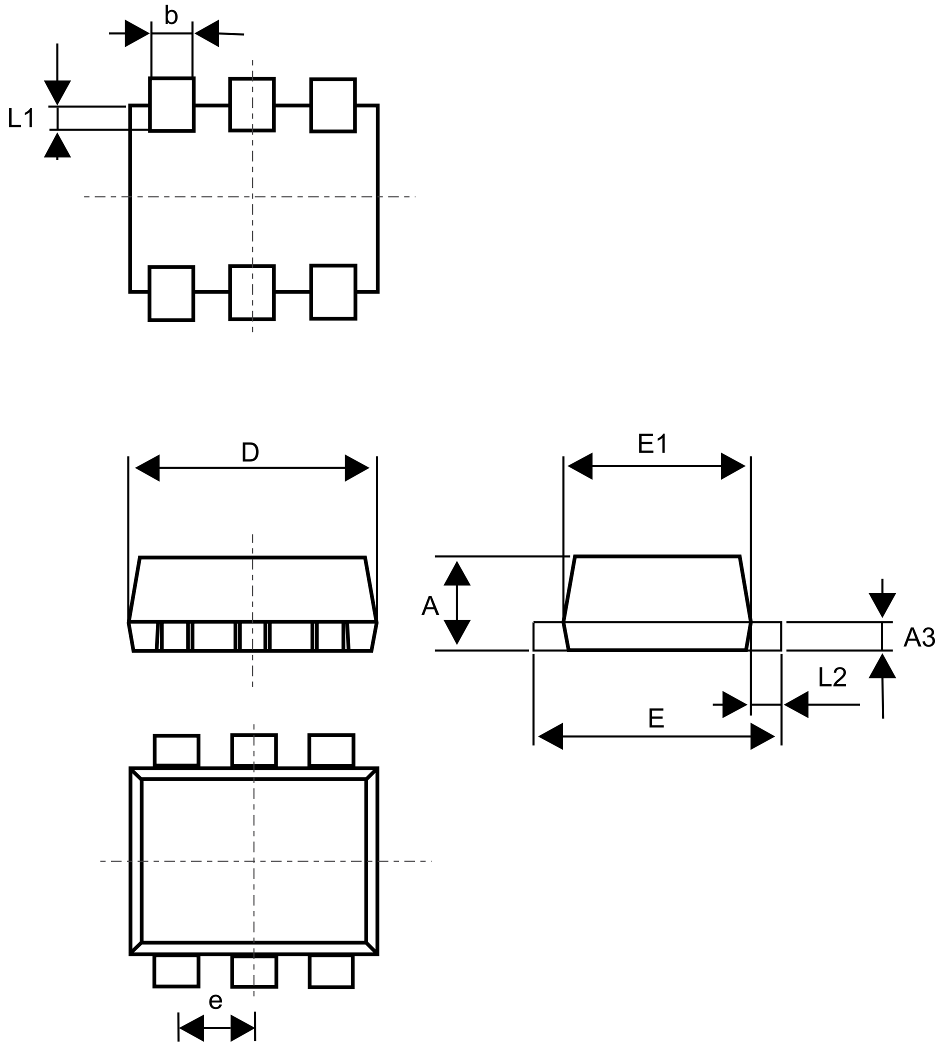

Figure 20. SOT-666 package outline

Table 4. **SOT-666 package mechanical data**

Ref.	Dimensions					
	Millimeters			Inches(1)		
	Min.	Typ.	Max.	Min.	Typ.	Max.
A	0.45		0.62	0.018		0.025
A3	0.08		0.18	0.003		0.007
b	0.17		0.34	0.007		0.013
D	1.50		1.70	0.059		0.067
E	1.50		1.70	0.059		0.067
E1	1.10		1.30	0.043		0.051
e		0.50			0.020	
L1		0.19			0.007	
L2	0.10		0.30	0.004		0.012

1. *Value in inches are converted from mm and rounded to 4 decimal digits*

Figure 21. **Footprint recommendations, dimensions in mm**

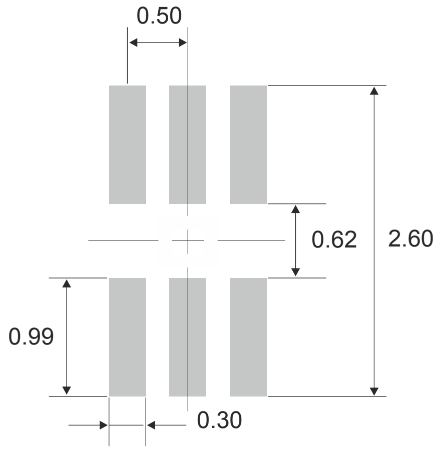

3.3 Packing information

Figure 22. Marking layout (refer to ordering information table for marking)

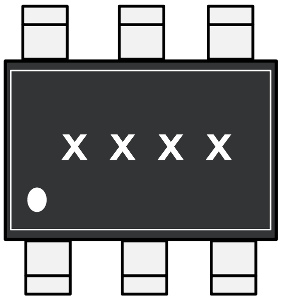

Figure 23. Package orientation in reel

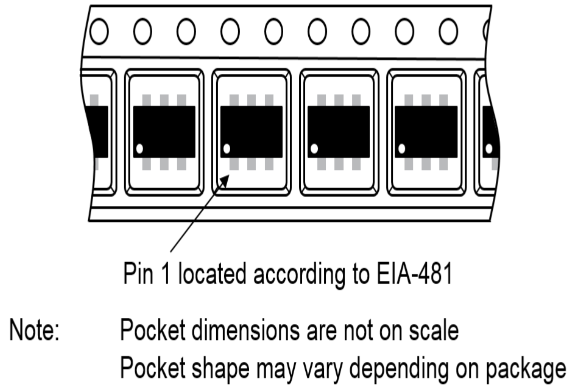

Figure 24. Tape and reel orientation

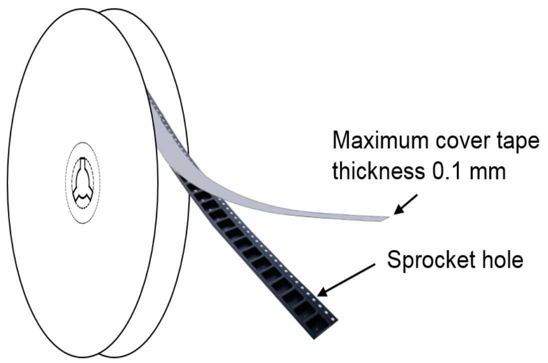

Figure 25. Reel dimensions (mm)

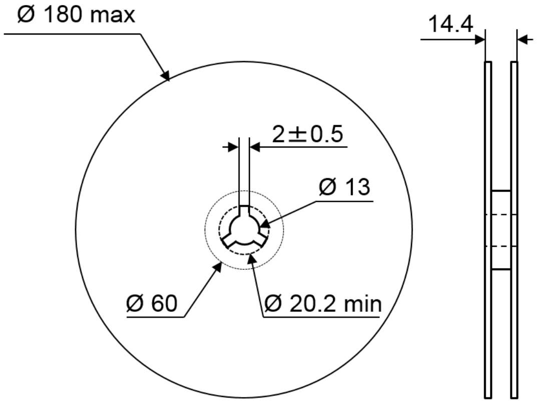

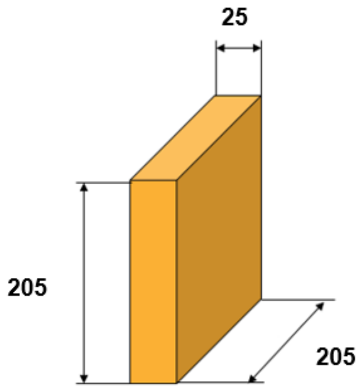

Figure 26. Inner box dimensions (mm)

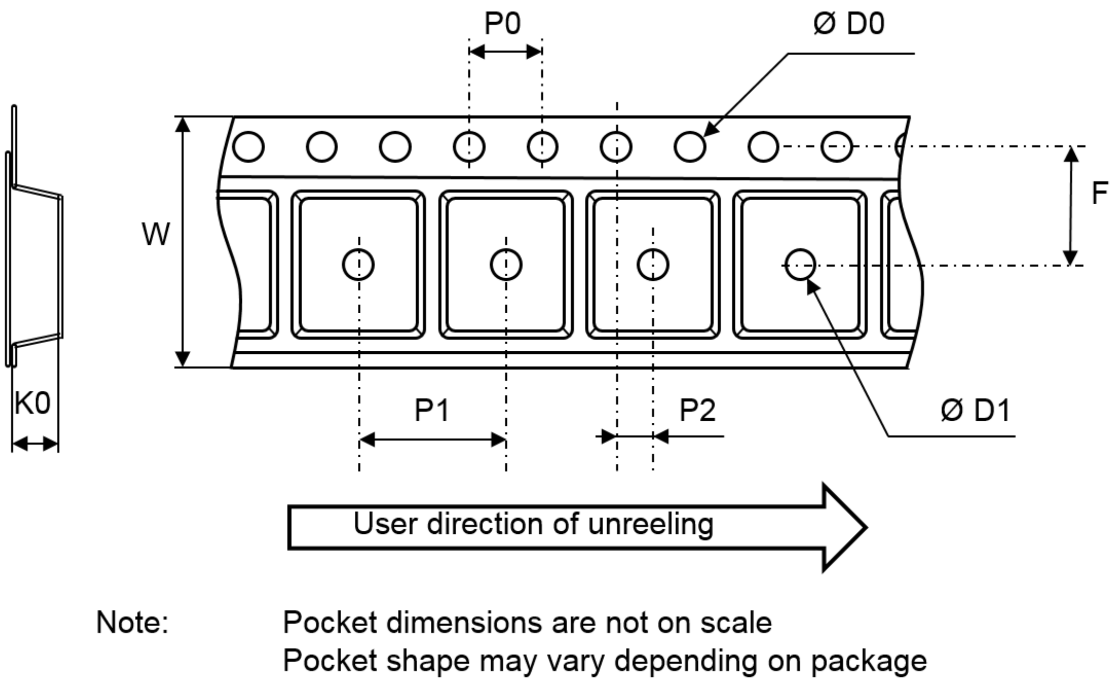

Figure 27. Tape and reel outline

Table 5. Tape and reel mechanical data

Ref.	Dimensions		
	Millimeters		
	Min.	Typ.	Max.
P1	3.9	4	4.1
P0	3.9	4	4.1
D0	1.45	1.5	1.6
D1	1		
F	3.45	3.5	3.55
K0	1.3	1.4	1.6
P2	1.95	2	2.05
W	7.9	8	8.3

4 Ordering information

Figure 28. Ordering information scheme

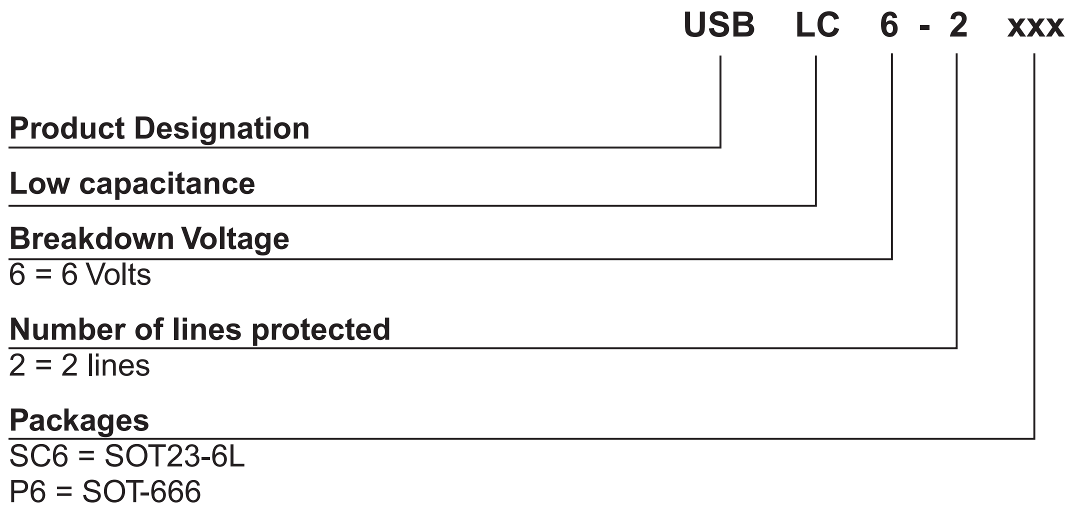

Table 6. Ordering information

Order code	Marking	Package	Weight	Base qty.	Delivery mode
USBLC6-2SC6 (1)	UL26	SOT23-6L	16.7 mg	3000	Tape and reel
USBLC6-2P6(1)	F	SOT-666	2.9 mg	3000	Tape and reel

1. *The marking code can be rotated by 90° to differentiate assembly location.*

Revision history

Table 7. Document revision history

Date	Version	Changes
14-Mar-2005	1	Initial release.
07-Jun-2005	2	Format change to figure 3; no content changed.
20-Mar-2008	3	Added marking illustrations - Figures 21 and 23. Added ECOPACK statement. Updated operating junction temperature range in absolute ratings, page 2. Technical information section updated. Reformatted to current standards.
27-Jun-2011	4	Updated leakage current for VRM = 5.25 V as specified in USB standard. Updated marking illustrations Figure 21 and Figure 23.
24-Oct-2011	5	Updated legal statement.
16-Oct-2020	6	Minor text changes.

IMPORTANT NOTICE – PLEASE READ CAREFULLY

STMicroelectronics NV and its subsidiaries (“ST”) reserve the right to make changes, corrections, enhancements, modifications, and improvements to ST products and/or to this document at any time without notice. Purchasers should obtain the latest relevant information on ST products before placing orders. ST products are sold pursuant to ST’s terms and conditions of sale in place at the time of order acknowledgement.

Purchasers are solely responsible for the choice, selection, and use of ST products and ST assumes no liability for application assistance or the design of Purchasers’ products.

No license, express or implied, to any intellectual property right is granted by ST herein.

Resale of ST products with provisions different from the information set forth herein shall void any warranty granted by ST for such product.

ST and the ST logo are trademarks of ST. For additional information about ST trademarks, please refer to www.st.com/trademarks. All other product or service names are the property of their respective owners.

Information in this document supersedes and replaces information previously supplied in any prior versions of this document.

© 2020 STMicroelectronics – All rights reserved
