# Supplementary material for: The Unfolding Space Glove: A Wearable Spatio-Visual to Haptic Sensory Substitution Device for Blind People
Source: Sensors (Basel). 2022 Feb 26;22(5):1859. doi: 10.3390/s22051859 (PMC8914703; doi:10.3390/s22051859)
Supplement: Supplementary file 1 [file sensors-22-01859-s001.zip › S1_GitHub-Release_unfolding-space-hardware-0.2.1-revised.zip!/unfolding-space-hardware-0.2.1-revised/pcb/exports/Rev2-schematic.pdf]

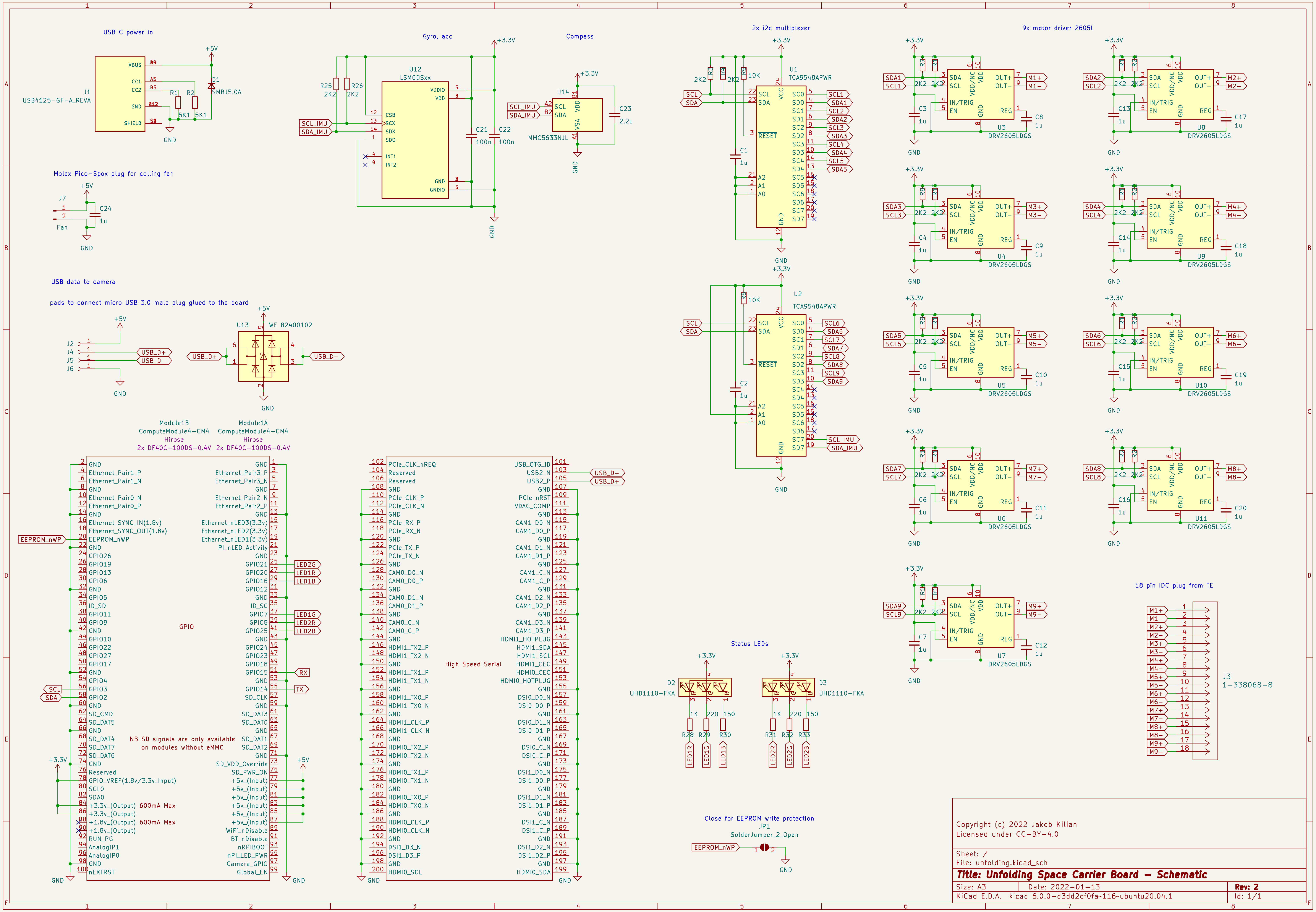

Copyright (c) 2022 Jakob Kilian
Licensed under CC-BY-4.0

Sheet: /
File: unfolding.kicad_sch

Title: Unfolding Space Carrier Board - Schematic

Size: A3 | Date: 2022-01-13 | Rev: 2
KiCad E.D.A. kicad 6.0.0-d3dd2cf0fa-116-ubuntu20.04.1 | Id: 1/1
