# Supplementary material for: The Unfolding Space Glove: A Wearable Spatio-Visual to Haptic Sensory Substitution Device for Blind People
Source: Sensors (Basel). 2022 Feb 26;22(5):1859. doi: 10.3390/s22051859 (PMC8914703; doi:10.3390/s22051859)
Supplement: Supplementary file 1 [file sensors-22-01859-s001.zip › S4_Exemplary_Timetable.zip!/S4_Exemplary_Timetable.pdf]

S2 – Exemplary Timetable for both Groups

Group A

- blind
- experience with the cane

Session 1

Hour	Activity	Duration
0	(Pick Up)	*20 min*
	**Introduction**	*30 min*
	**Tutorial**	*10 min*
1	**TS1**	*30 min*
	**PS1** (SSD)	*15 min*
	**Pause**	*15 min*
2	**TS2**	*30 min*
	(Drop Off)	*20 min*
3		

Session 2

Hour	Activity	Duration
0	(Pick Up)	*20 min*
	**PS2** (SSD)	*15 min*
	**Pause**	*15 min*
1	**TS3**	*30 min*
	(Drop Off)	*20 min*
2		

Session 3

Hour	Activity	Duration
0	(Pick Up)	*20 min*
	**PS3** (SSD)	*15 min*
	**Pause**	*15 min*
1	**TS4**	*30 min*
	**Questionnaire**	*30 min*
2	(Drop Off)	*20 min*

Group B

- no visual impairement
- no prior experience with the cane

Session 1

Aid order (SSD or cane first) being randomized for each Tutorial and Training

Hour	Activity	Duration
0	**Introduction**	*30 min*
	**Tutorial** (Aid 1)	*10 min*
	**Tutorial** (Aid 2)	*10 min*
1	**TS1**	*30 min*
	**PS1** (Aid 1)	*15 min*
	**PS1** (Aid 2)	*15 min*
	**Pause**	*15 min*
2	**TS2**	*30 min*
3		

Session 2

Hour	Activity	Duration
0	**PS2** (Aid 1)	*15 min*
	**PS2** (Aid 2)	*15 min*
	**Pause**	*15 min*
	**TS3**	*30 min*
1		
2		

Session 3

Hour	Activity	Duration
0	**PS3** (Aid 1)	*15 min*
	**PS3** (Aid 2)	*15 min*
	**Pause**	*15 min*
1	**TS4**	*30 min*
	**Questionnaire**	*30 min*
2		
