# Supplementary material for: The Unfolding Space Glove: A Wearable Spatio-Visual to Haptic Sensory Substitution Device for Blind People
Source: Sensors (Basel). 2022 Feb 26;22(5):1859. doi: 10.3390/s22051859 (PMC8914703; doi:10.3390/s22051859)
Supplement: Supplementary file 1 [file sensors-22-01859-s001.zip › S6_GitHub-Release_unfolding-space-monitor-1.4.zip!/unfolding-space-monitor-1.4/Assets/SimplestPlot/ReadMe.pdf]

SIMPLEST PLOT

Description

Add the script to a GameObject containing an image.

There are three types of possible plots. All variable are public and accessible directly, except from the resolution of the texture, which is accessed through *SetResolution(`Vector2 NewResolution`)* and *GetResolution()* Methods.

Once you have finished editing the parameters and passing the values, call `UpdatePlot()` to draw the plot.

GitHub: https://github.com/NeuralDip/SimplestPlot

Global Variables

Some parameters are independent of the type of plot:

`PlotType`	`MyPlotType`	: Select the type of the plot.
`Bool`	AxesVisible	: Show-hide the Min and Max values of the axes.
`int`	FontSize	: The fontsize for the axes and warnings text.
`Color`	`TextColor`	: Set the text color.
`Color`	`BackGroundColor`	: Set the background Color.
`bool`	`ShowWarnings`	: Show warnings in case data are not consistent.

TimeSeries

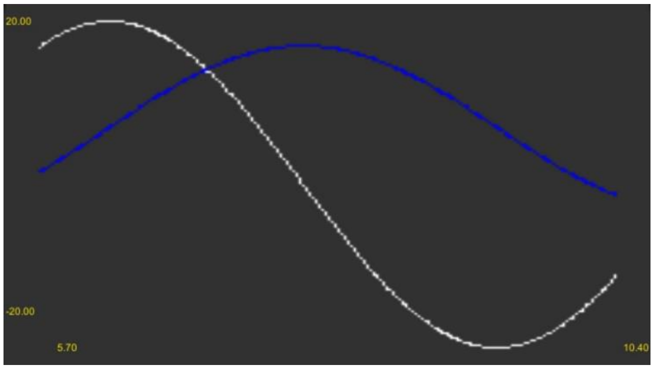

`float[]`	`SeriesPlotX`	: Array of the X values. This is optional.
`List<SeriesClass>`	`SeriesPlotY`	: List of the TimeSeries.
`float[]`	`SeriesPlotY.YValues`	: The actual YValues.

`Color`	`SeriesPlotY.MyColor`	:	The color for this particular TimeSeries.

Distribution

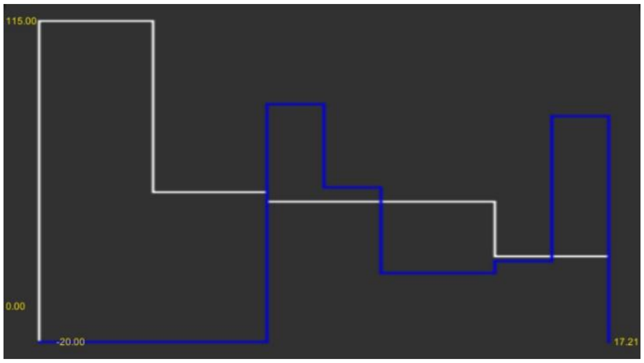

List<DistributionClass>	DistributionPlot	:	List of Distributions
`float[]`	DistributionPlot.`Values`	:	Actual values for the distribution.
`int`	DistributionPlot.NumberOfBins	:	Default = 10.
`Color`	DistributionPlot.MyColor	:	The color for this particulat Distribution.

PhaseSpace

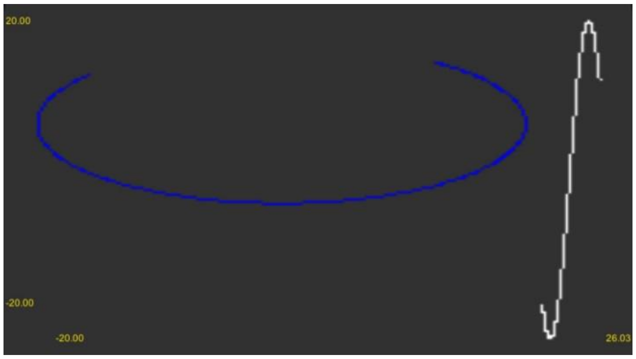

List<PhaseSpaceClass>	PhaseSpacePlot	:	List of Phase Space plots.
float[]	PhaseSpacePlot.XValues	:	XValues for the Plot.
float[]	PhaseSpacePlot.YValues	:	YValues for the Plot.
Color	PhaseSpacePlot.MyColor	:	The color for this particular Phase Space plot.
